# Supplementary material for: Catalytic [4+2]- and [4+4]-cycloaddition using furan-fused cyclobutanone as a privileged C4 synthon
Source: Nat Commun. 2024 Jun 26;15:5407. doi: 10.1038/s41467-024-49664-5 (PMC11208666; doi:10.1038/s41467-024-49664-5)
Supplement: Supplementary file 1 — Supplementary Information [file 41467_2024_49664_MOESM1_ESM.pdf]

SUPPLEMENTARY INFORMATION

Catalytic [4+2]- and [4+4]-cycloaddition using furan-fused cyclobutanone as a privileged C4 synthon

Kemiao Hong[1,§], Mengting Liu[2,§], Lixin Qian[2], Ming Bao[1], Gang Chen[2], Xinyu Jiang[2], Jingjing Huang[2] & Xinfang Xu[1,*]

[1]*School of Chemistry and Chemical Engineering, Zhejiang Sci-Tech University, Hangzhou 310018, China*
[2]*School of Pharmaceutical Sciences, Sun Yat-sen University, Guangzhou, Guangdong 510006, China*
[§]These authors contributed equally: Kemiao Hong, Mengting Liu

Email: xuxinfang@zstu.edu.cn

Table of Contents

1. General Information **S2**

2. Optimization of the Reaction Conditions **S3-S13**

3. General Procedure for the [4+2] Cycloaddition Reaction **S14-S40**

4. General Procedure for the [4+4] Cycloaddition Reaction **S41-S58**

5. General Procedure for Scale Up and Synthetic Applications **S59-S67**

6. 1D-NOE NMR Analysis of 10 and 13 **S68**

7. Control Experiments **S69-S70**

8. NMR Spectra of New Compounds 3, 4, and 6-15 **S71-S160**

9. HPLC Analyses Figures of Compounds 3, 4, and 8-11 **S161-S193**

10. Single-Crystal *X*-ray Diffraction of 4k, 6f, 8, 9, and 14 **S194-S198**

11. General Procedure for the *in vitro* Anti-tumor Activity Study **S199-S204**

12. Supplementary References **S205**

General Information

All reactions were carried out in oven-dried glassware. Solvents were purified and distilled by following the standard methods. Flash column chromatography was performed using silica gel (300-400 mesh). Analytical thin-layer chromatography was performed using glass plates pre-coated with 200-300 mesh silica gel impregnated with a fluorescent indicator (254 nm). The ^{1}H NMR, ^{13}C NMR, and ^{19}F NMR spectra were recorded in $CDCl_3$ on 400 MHz and 500 MHz spectrometer; chemical shifts were reported in ppm with the solvent signal as reference, and coupling constants (J) were given in Hertz. The peak information was described as: s = singlet, d = doublet, t = triplet, q = quartet, m = multiplet, comp = composite. The enantioselectivity was determined on HPLC using Daicel Chiralpak IA and IC column. High-resolution mass spectra (HRMS) were recorded on a commercial apparatus (ESI Source). All substrates whose syntheses were not described herein were either obtained from commercial suppliers or prepared using the referenced literature procedures[1-4]. Unless stated otherwise, all commercially available compounds were used as received.

Optimization of the Reaction Conditions

Supplementary Table 1 | Condition optimization for the [4+4]-cycloaddition[a]

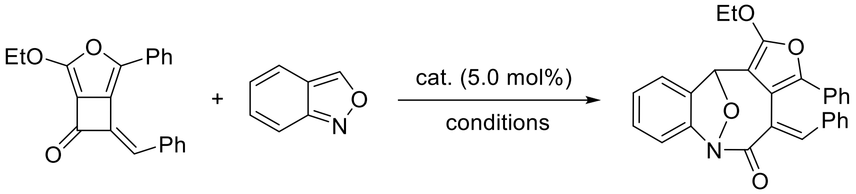

1a **5a** **6a**

Entry	Cat. (5.0 mol%)	Solvent	T (°C)	Yield (%)[b]
1	$Pd_2(dba)_3$	DCE	40	ND[c]
2	$Rh(COD)_2BF_4$	DCE	40	NR[d]
3	$Cu(OTf)_2$	DCE	40	36
4	$Fe(OTf)_3$	DCE	40	21
5	$Sc(OTf)_3$	DCE	40	17
6	$AgSbF_6$	DCE	40	38
7	PPh_3AuNTf_2	DCE	40	62
8	$IPrAuNTf_2$	DCE	40	83
9	$Me_3(OMe)tBuXPhosAuNTf_2$	DCE	40	61
10	$JohnPhosAu(MeCN)SbF_6$	DCE	40	98(93)[e]
11	$JohnPhosAu(MeCN)SbF_6$	DCM	40	86
12	$JohnPhosAu(MeCN)SbF_6$	THF	40	37
13	$JohnPhosAu(MeCN)SbF_6$	EtOAc	40	30
14	$JohnPhosAu(MeCN)SbF_6$	*p*-xylene	40	19[f]
15	$JohnPhosAu(MeCN)SbF_6$	$PhCF_3$	40	78
16	$JohnPhosAu(MeCN)SbF_6$	DCE	60	90
17	$JohnPhosAu(MeCN)SbF_6$	DCE	25	92

[a]The reaction was carried out on a 0.1 mmol scale. To a solution of **1a** (31.6 mg, 0.1 mmol) and **5a** (13.1 mg, 0.11 mmol, 1.1 equiv.) in 1.0 mL solvent, was added a solution of catalyst (5.0 mol%) in 1.0 mL same solvent via syringe under argon atmosphere at indicated temperature, and the reaction mixture was stirred for 18 h under these conditions. [b]Determined by ^1H NMR analysis of the crude reaction mixture based on internal standard (1,3,5-trimethoxybenzene). [c]ND = not detected. [d]NR = not reaction. [e]Isolated yield. [f]Most of the starting material **1a** and **5a** was recovered.

Supplementary Table 2 | Screening of chiral gold catalysts for the [4+2]-cycloaddition[a]

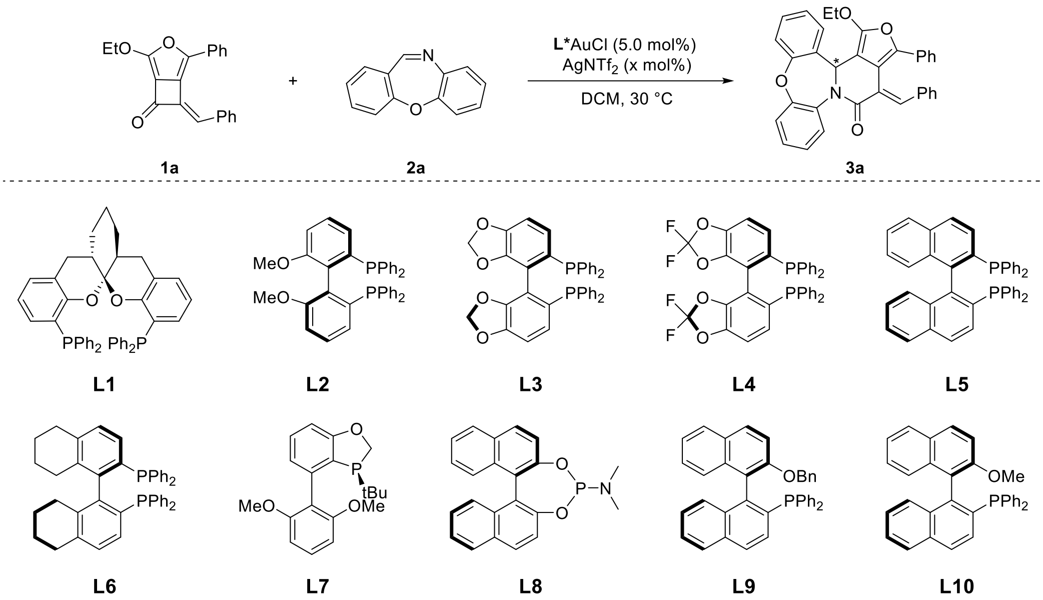

Entry	[Au]	$AgNTf_2$ (x mol%)	Yield (%)[b]	ee (%)[c]
1	**L1**$(AuCl)_2$	10	44	19
2	**L2**$(AuCl)_2$	10	18	5
3	**L3**$(AuCl)_2$	10	54	0
4	**L4**$(AuCl)_2$	10	61	5
5	**L5**$(AuCl)_2$	10	30	5
6	**L6**$(AuCl)_2$	10	39	0
7	**L7**AuCl	5.0	27	5
8	**L8**AuCl	5.0	13	22
9	L9AuCl	5.0	36	40
10	**L10**AuCl	5.0	41	13

[a]The reaction was carried out on a 0.1 mmol scale. To a solution of **1a** (31.6 mg, 0.1 mmol) and **2a** (19.5 mg, 0.11 mmol, 1.1 equiv.) in 1.0 mL DCM, was added a solution of gold catalyst (5.0 mol%) and $AgNTf_2$ (10 or 5.0 mol%) in 1.0 mL DCM via syringe under argon atmosphere at 30 ^oC, and the reaction mixture was stirred for 12 h under these conditions. [b]Determined by ^1H NMR analysis of the crude reaction mixture based on limited reagent **2a** with mesitylene as internal standard. [c]Determined by Chiral HPLC analysis with Chiralpak IC column.

Supplementary Table 3 | Screening of chiral ligands for the Rh-catalyzed enantioselective [4+2]-cycloaddition[a]

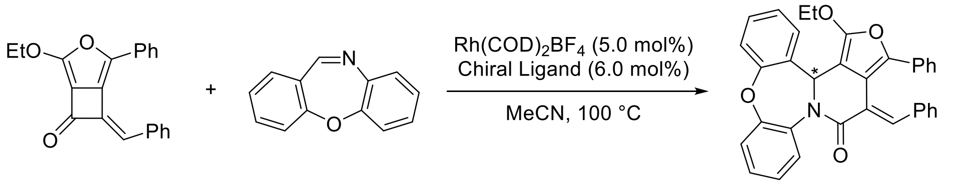

1a **2a** **3a**

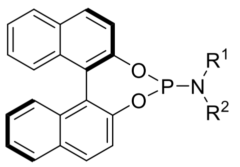

L11, R^1 = R^2 = Me, 91% yield, 73% *ee*
L12, R^1 = R^2 = Et, 89% yield, 20% *ee*
L13, R^1 = R^2 = *i*Pr, 68% yield, 53% *ee*
L14, R^1 = R^2 = *n*Pr, 60% yield, 55% *ee*
L15, R^1 = Ph, R^2 = Me, 57% yield, 0% *ee*
L16, R^1 = Bn, R^2 = Me, 55% yield, 55% *ee*
L17, R^1 = Allyl, R^2 = Me, 66% yield, 23% *ee*

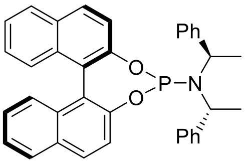

L18, 56% yield, 15% *ee*

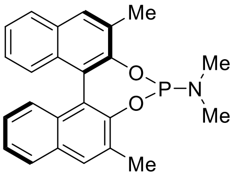

L19, 75% yield, 65% *ee*

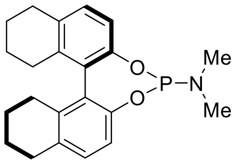

L20, 20% yield, 0% *ee*

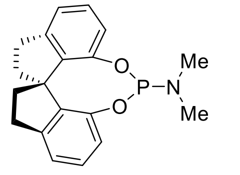

L21, 59% yield, 37% *ee*

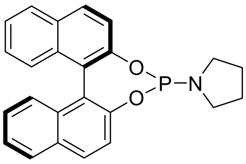

L22, 71% yield, 84% *ee*

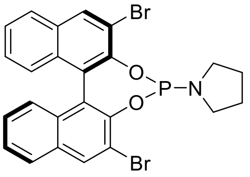

L23, 65% yield, -27% *ee*

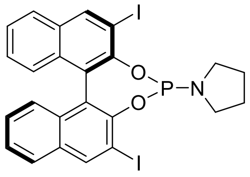

L24, 6% yield, 15% *ee*

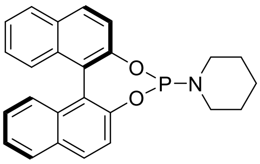

L25, 67% yield, 55% *ee*

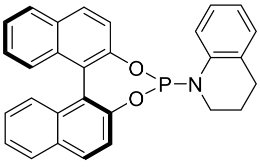

L26, 63% yield, -25% *ee*

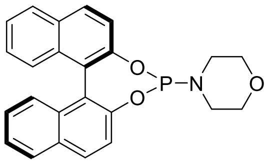

L27, 61% yield, 51% *ee*

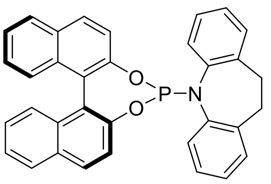

L28, 60% yield, -41% *ee*

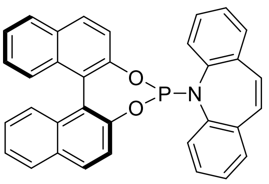

L29, 72% yield, -40% *ee*

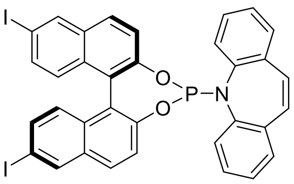

L30, 65% yield, -45% *ee*

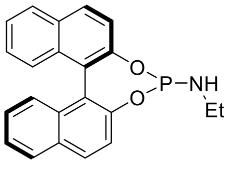

L31, 61% yield, 55% *ee*

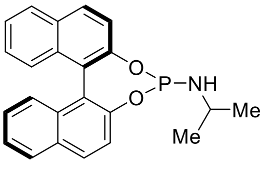

L32, 65% yield, 93% *ee*

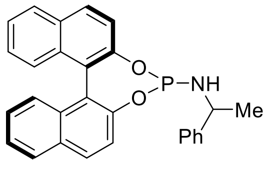

L33, 70% yield, -83% *ee*

Supplementary Table 4 | Condition optimization for the Rh-catalyzed enantioselective [4+2]-cycloaddition[a]

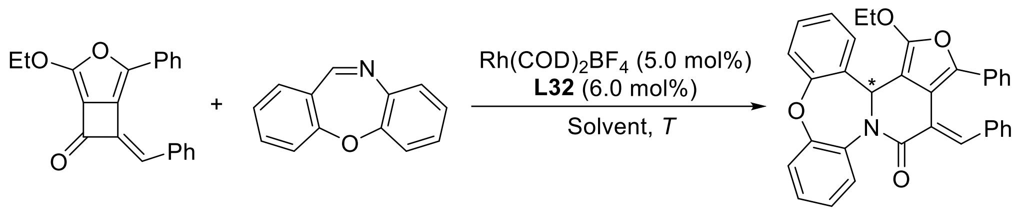

Entry	Solvent	T (°C)	Yield (%)[b]	ee (%)[c]
1	THF	100	56	-75
2	toluene	100	35	-61
3	p-xylene	100	35	-65
4	$MeNO_2$	100	45	0
5	1,4-dioxane	100	36	-75
6	PhCl	100	49	-55
7	$PhCF_3$	100	20	0
8[d]	MeCN	100	50	89
9[e]	MeCN	100	65	93
10[f]	MeCN	100	52	91
11[e]	MeCN	100	51	94
12[e]	MeCN	100	45	95
13[e]	MeCN	80	28	91
14[e]	MeCN	95	39	90
15[e]	MeCN	100	65	93
16[e]	MeCN	105	46	90
17[e]	MeCN	120	17	81
18[e]	MeCN	140	20	71
19[d,e]	MeCN	100	67	93
20[d,e,g]	MeCN	100	70	93
21[d,e,h]	MeCN	100	69	93

[a]The reaction was carried out on a 0.1 mmol scale. To a solution of **1a** (31.6 mg, 0.1 mmol) and **2a** (21.5 mg, 0.11 mmol, 1.1 equiv.) in 1.0 mL solvent, was added a solution of $Rh(COD)_2BF_4$ (2.0 mg, 5.0 mol%) and **L32** (2.3 mg, 6.0 mol%) in 1.0 mL same solvent via syringe at 100 °C under argon atmosphere, and the reaction mixture was stirred for 12 h under these conditions. [b]Determined by ^1H NMR analysis of the crude reaction mixture based on internal standard (1,3,5-trimethoxybenzene). [c]Determined by chiral HPLC analysis with a Chiralpak IC column. [d]$Rh(COD)_2BF_4$ (10 mol%) and **L32** (12 mol%) were added. [e]50.0 mg 4Å MS was used as additive. [f]50.0 mg 5Å MS was used as additive. [g]**1a** (0.12 mmol) and **2a** (0.1 mmol) was added. [h]**1a** (0.2 mmol and **2a** (0.1 mmol) was added.

Supplementary Table 5 | Screening of chiral ligands for the enantioselective [4+4]-cycloaddition[a]

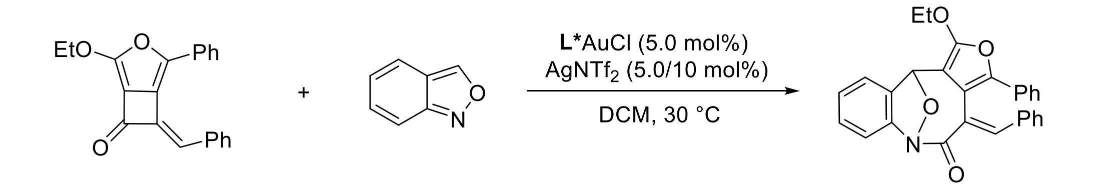

1a **5a** **6a**

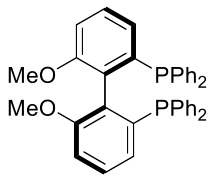

L2, 83% yield, 16 % *ee*

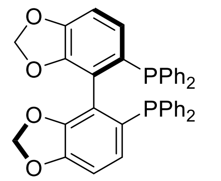

L3, 98% yield, 26% *ee*

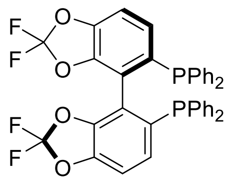

L4, 88% yield, 34% *ee*

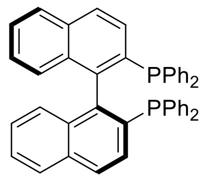

L5, 82% yield, <5% *ee*

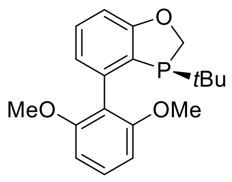

L7, 67% yield, <5% *ee*

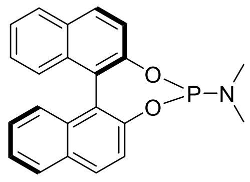

L8, 75% yield, <5% *ee*

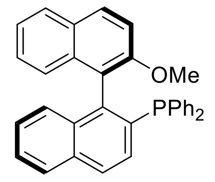

L10, 78% yield, 6% *ee*

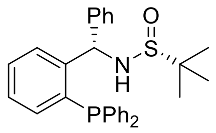

L34, 60% yield, <5% *ee*

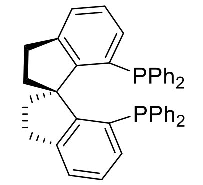

L35, 79% yield, 11% *ee*

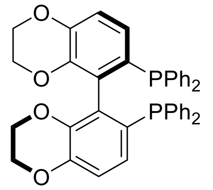

L36, 85% yield, <5% *ee*

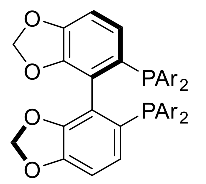

Ar = 3,5-2MeC_6H_3
L37, 94% yield, 20% *ee*

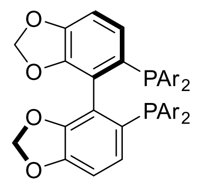

Ar = 3,5-2*t*Bu-4-OMeC_6H_2
L38, 93% yield, 60% *ee*

Supplementary Table 6 | Condition optimization for the enantioselective [4+4]-cycloaddition[a]

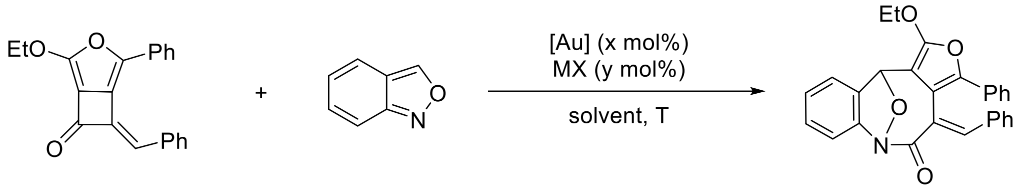

Entry	[Au] (x mol%)	MX (y mol%)	Solvent	Yield (%)[b]	ee (%)[c]
1	**L38**$(AuCl)_2$ (5.0)	AgOTf (10)	DCM	85	49
2	**L38**$(AuCl)_2$ (5.0)	$AgSbF_6$ (10)	DCM	93	64
3	**L38**$(AuCl)_2$ (5.0)	$AgBF_4$ (10)	DCM	82	57
4	**L38**$(AuCl)_2$ (5.0)	NaBArF (10)	DCM	83	57
5	**L38**$(AuCl)_2$ (5.0)	$AgSbF_6$ (5.0)	DCM	80	60
6	**L38**$(AuSbF_6)_2$ (5.0)	-	DCM	91	64
7	**L38**$(AuSbF_6)_2$ (5.0)	-	DCE	87	56
8	**L38**$(AuSbF_6)_2$ (5.0)	-	$PhCF_3$	85	40
9	**L38**$(AuSbF_6)_2$ (5.0)	-	EtOAc	60	49
10[d]	**L38**$(AuSbF_6)_2$ (5.0)	-	DCM	94	60
11[e]	**L38**$(AuSbF_6)_2$ (5.0)	-	DCM	93	62
12[f]	**L38**$(AuSbF_6)_2$ (5.0)	-	DCM	90	59
13[g]	**L38**$(AuSbF_6)_2$ (5.0)	-	DCM	trace	-
14	**L38**$(AuSbF_6)_2$ (10)	-	DCM	91	68

[a]To a solution of gold catalyst (x mol%) in 1.0 mL indicated solvent, was added a solution of **1a** (22.8 mg, 0.072 mmol, 1.2 equiv.) and **5a** (7.1 mg, 0.06 mmol) in the same solvent (1.0 mL) via syringe under argon atmosphere at room temperature, and the reaction mixture was stirred under these conditions. [b]Determined by ^1H NMR analysis of the crude reaction mixture based on limited reagent **5a** with mesitylene as internal standard. [c]Determined by Chiral HPLC analysis with Chiralpak OD-H column. [d]50 mg 3 Å MS was added. [e]50 mg 4 Å MS was added. [f]50 mg 5 Å MS was added. [g]The reaction was conducted at 0 ^oC and most of the starting material **1a** and **5a** was recovered.

Supplementary Table 7 | Condition optimization for the Rh-catalyzed enantioselective [4+2]-cycloaddition with imine 2r[a]

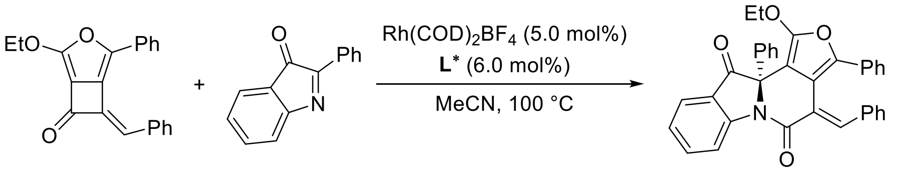

1a **2r** **3r**

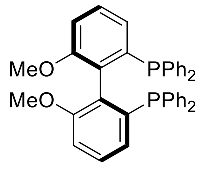

L2, 85%, >99% *ee*

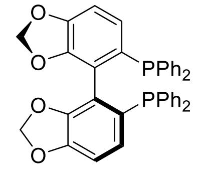

L3, 78%, 99% *ee*

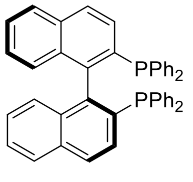

L5, 83%, 99% *ee*

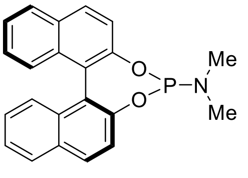

L8, 72%, 38% *ee*

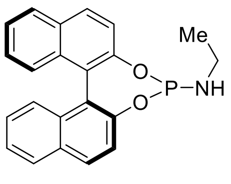

L31, 62%, 30% *ee*

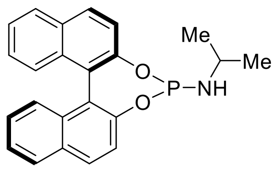

L32, 68%, 52% *ee*

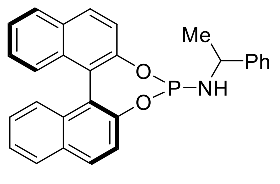

L33, 34%, 18% *ee*

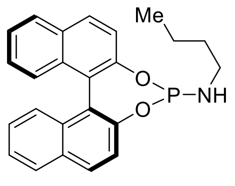

L39, 48%, 10% *ee*

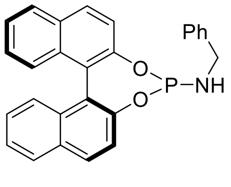

L40, 27%, 20% *ee*

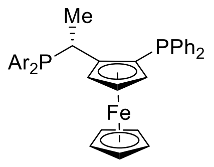

Ar = 3,5-$(Me)_2C_6H_3$
L41, 46%, 93% *ee*

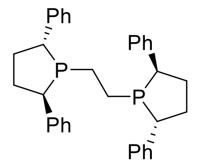

L42, NR

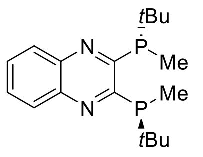

L43, NR

[a]Reaction conditions: **1a** (34.8 mg, 0.11 mmol, 1.1 equiv.), **2r** (20.8 mg, 0.1 mmol), $Rh(COD)_2BF_4$ (2.0 mg, 5.0 mol%), chiral ligand (6.0 mol%) in MeCN (2.0 mL) under argon atmosphere at 100 °C for 12 h. The yields were determined by ^1H NMR analysis of the crude reaction mixture based on internal standard (1,3,5-trimethoxybenzene). The ee values were determined by chiral HPLC analysis with a Chiralpak IC column. NR = no reaction.

Supplementary Table 8 | Condition optimization for the Rh-catalyzed enantioselective [4+2]-cycloaddition with non-cyclic aldimine 2t[a]

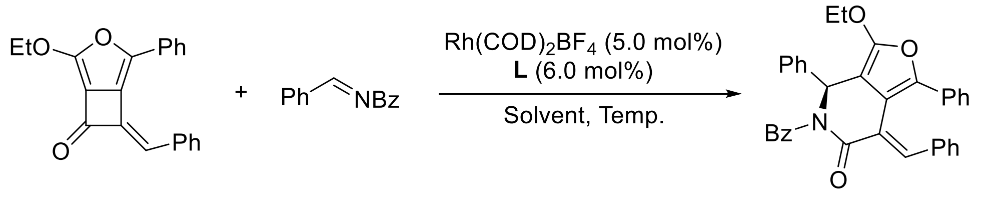

1a **2t** **3t**

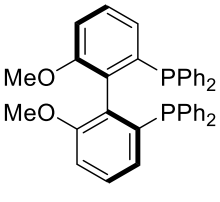

L2

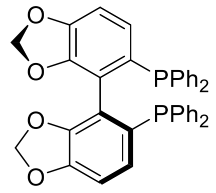

L3

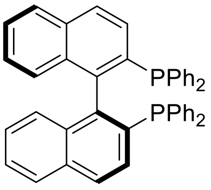

L5

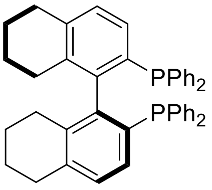

L6

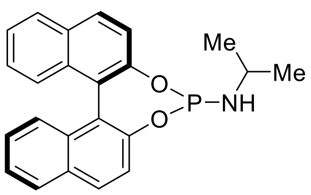

L32

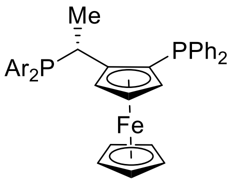

Ar = 3,5-$(Me)_2C_6H_3$

L41

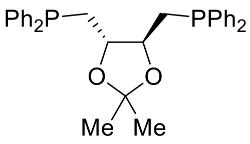

L44

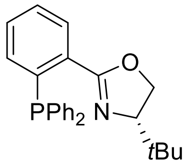

L45

Entry	Ligand (6.0 mol%)	Solvent	T (°C)	Yield (%)[b]	ee (%)[c]
1	**L2**	MeCN	100	60	45
2	**L3**	MeCN	100	68	66
**3**	**L5**	MeCN	100	62	10
4	**L6**	MeCN	100	57	65
5	**L32**	MeCN	100	58	15
6	**L41**	MeCN	100	55	80
7	**L41**	DCE	100	57	50
8	**L41**	PhMe	100	61	4
9	**L41**	MeCN	60	NR[d]	-
10	**L41**	MeCN	80	<5[d]	-
11	**L44**	MeCN	100	43	56
12	**L45**	MeCN	100	NR[d]	-

[a]Reaction conditions: **1a** (34.8 mg, 0.11 mmol, 1.1 equiv.), **2t** (20.8 mg, 0.1 mmol), $Rh(COD)_2BF_4$ (2.0 mg, 5.0 mol%), **L** (6.0 mol%) in 2.0 mL solvent at 100 °C under argon atmosphere for 12 h. [b]Determined by ^1H NMR analysis of the crude reaction mixture based on internal standard (1,3,5-trimethoxybenzene). [c]Determined by chiral HPLC analysis with a Chiralpak IC column. [d]most of starting material **1a** was recovered.

Supplementary Table 9 | Condition optimization for the Au-catalyzed enantioselective [4+2]-cycloaddition with 2p[a]

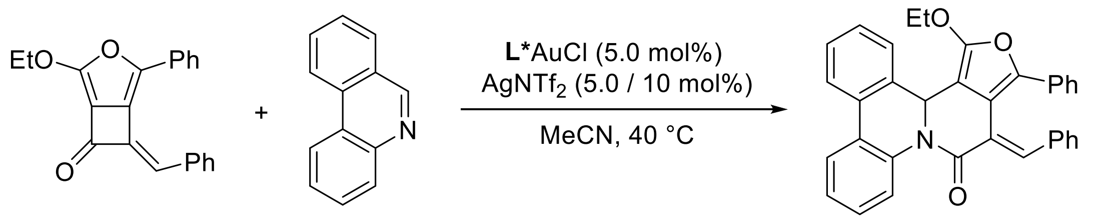

1a **2p** **3p**

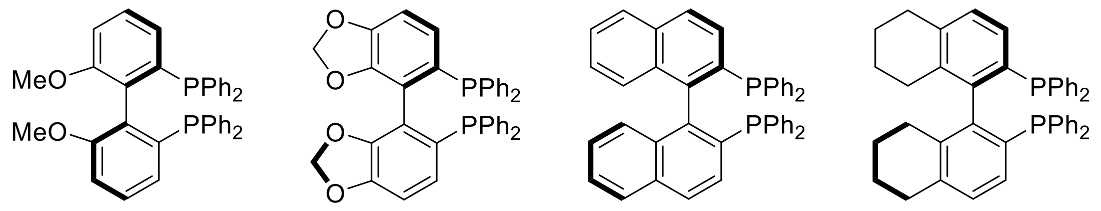

L2 **L3** **L5** **L6**

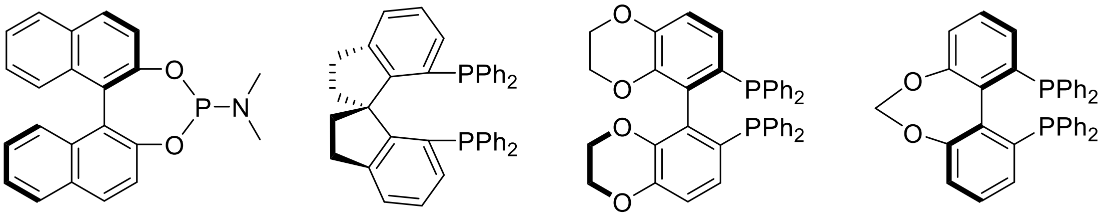

L8 **L35** **L36** **L46**

Entry	[M] (x mol%)	$AgNTf_2$ (y mol%)	Yield (%)[b]	ee (%)[c]
**1**	**L2**[AuCl]$_2$ (5.0)	10	41	40
2	**L3**[AuCl]$_2$ (5.0)	10	36	13
3	**L5**[AuCl]$_2$ (5.0)	10	57	20
4	**L6**[AuCl]$_2$ (5.0)	10	42	16
5	**L8**AuCl (5.0)	5.0	<5[d]	-
6	**L35**[AuCl]$_2$ (5.0)	10	35	32
7	**L36**[AuCl]$_2$ (5.0)	10	59	11
8	**L46**[AuCl]$_2$ (5.0)	10	47	53

[a]Reaction conditions: **1a** (34.8 mg, 0.11 mmol, 1.1 equiv.), **2p** (17.9 mg, 0.1 mmol), chiral gold catalyst (5.0 mol%), $AgNTf_2$ (10 or 5.0 mol%) in MeCN (2.0 mL) under argon atmosphere at 40 ºC for 12 h. [b]Determined by ^1H NMR analysis of the crude reaction mixture using mesitylene as an internal standard. [c]Determined by chiral HPLC analysis with Chiralpak IC column. [d]Most of the starting materials **1a** and **2p** were recovered.

Supplementary Table 10 | Condition optimization for the catalytic [4+2]-cycloaddition with 2q[a]

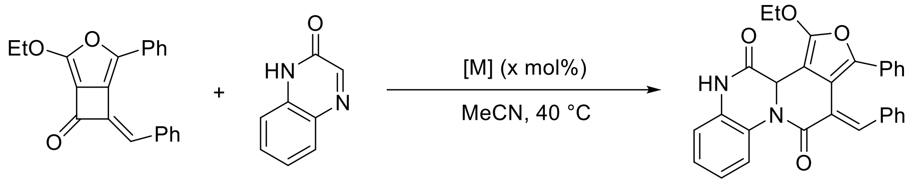

1a **2q** **3q**

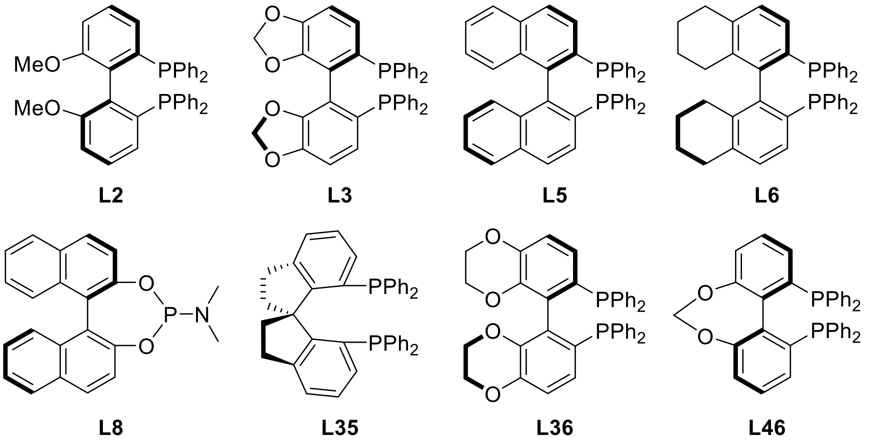

Entry	[M] (x mol%)	$AgNTf_2$ (y mol%)	Yield (%)[b]	ee (%)[c]
1	$Rh(COD)_2BF_4$ (5.0)	-	ND[d]	-
2	$Cu(MeCN)_4PF_6$ (5.0)	-	23	-
3	$[Ir(COD)Cl]_2$ (5.0)	-	12	-
4	AgOTf (5.0)	-	42	-
5	$IPrAuNTf_2$ (5.0)	-	70	-
6	**L2**$[AuCl]_2$ (5.0)	10	39	<5
7	**L3**$[AuCl]_2$ (5.0)	10	42	<5
8	**L35**$[AuCl]_2$ (5.0)	10	12	<5
9	**L5**$[AuCl]_2$ (5.0)	10	37	<5
10	**L6**$[AuCl]_2$ (5.0)	10	31	6
11	**L8**AuCl (5.0)	5.0	16	5
12	**L36**$[AuCl]_2$ (5.0)	10	43	<5
13	**L46**$[AuCl]_2$ (5.0)	10	27	<5

[a]Reaction conditions: **1a** (34.8 mg, 0.11 mmol, 1.1 equiv.), **2q** (14.6 mg, 0.1 mmol), catalyst (x mol%) in MeCN (2.0 mL) under argon atmosphere at 40 °C for 12 h. [b]Determined by ^1H NMR analysis of the crude reaction mixture using mesitylene as an internal standard. [c]Determined by chiral HPLC analysis with Chiralpak IC column. [d]This reaction was carried out in 100 °C. ND = no detected.

Supplementary Table 11 | Condition optimization for the catalytic [4+2]-cycloaddition with 2s[a]

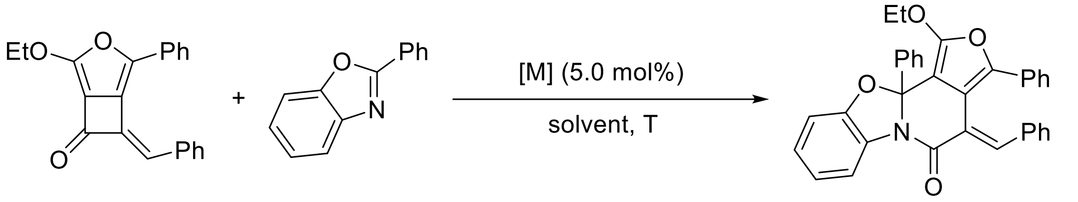

1a **2s** **3s**

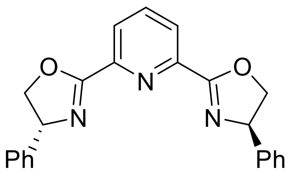

L47

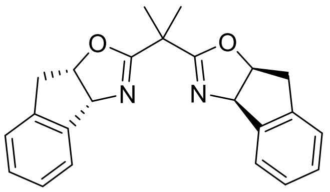

L48

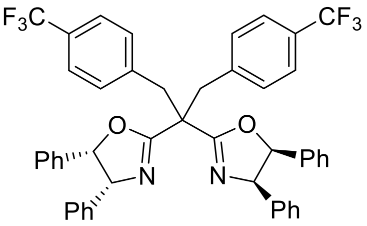

L49

Entry	[M] (5.0 mol%)	Solvent	T (°C)	Yield (%)[b]	ee (%)[c]
1	$Rh(COD)_2BF_4$	MeCN	100	ND[d]	-
2	$IPrAuNTf_2$	MeCN	40	40	-
3	$Cu(MeCN)_4PF_6$	MeCN	40	21	-
4	$Cu(MeCN)_4PF_6$	DCE	40	30	-
5	$Cu(MeCN)_4PF_6$/**L47** (6.0)	DCE	40	20	<5
6	$Cu(MeCN)_4PF_6$/**L48** (6.0)	DCE	40	NR	-
7	$Cu(MeCN)_4PF_6$/**L49** (6.0)	DCE	40	18	<5

[a]Reaction conditions: **1a** (34.8 mg, 0.11 mmol, 1.1 equiv.), **2s** (19.5 mg, 0.1 mmol), catalyst (5.0 mol%) in 2.0 mL solvent under argon atmosphere at 40 °C for 12 h. [b]Determined by ^1H NMR analysis of the crude reaction mixture using mesitylene as an internal standard. [c]Determined by chiral HPLC analysis with Chiralpak IC column. [d]Most of starting material **2s** was recovered. ND = no detected. NR = no reaction.

General Procedure for the [4+2] Cycloaddition Reaction

Condition A:

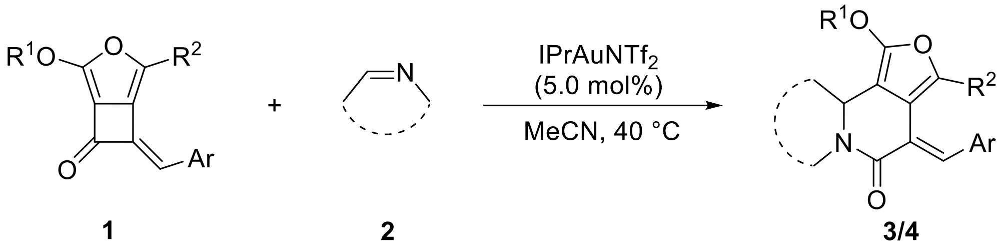

To a 10-mL oven-dried vial containing a magnetic stirring bar, cyclobutanone **1** (0.12 mmol, 1.2 equiv.), imine **2** (0.1 mmol, 1.0 equiv.), $IPrAuNTf_2$ (4.3 mg, 5.0 mol%), and MeCN (2.0 mL) were added sequentially under argon atmosphere. After addition, the reaction mixture was stirred overnight at 40 °C until consumption of the material (monitored by TLC). The solvent was evaporated in vacuo. Then, the residues was purified by column chromatography on silica gel without any additional treatment (Hexanes : EtOAc = 50:1 to 10:1) to give the pure products **3** or **4** in good to high yields.

Condition B:

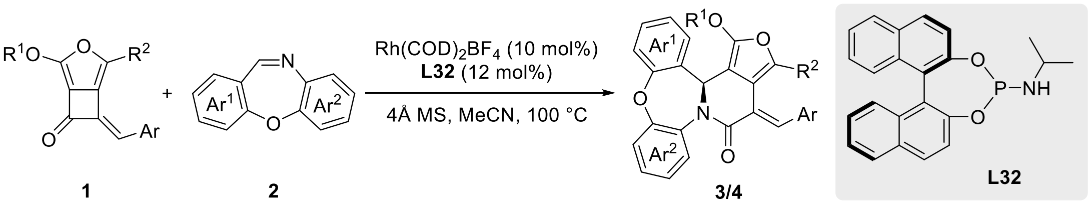

To a 10-mL oven-dried vial containing a magnetic stirring bar, cyclobutanone **1** (0.12 mmol, 1.2 equiv.), imine **2** (0.1 mmol, 1.0 equiv.), $Rh(COD)_2BF_4$ (4.0 mg, 10 mol%), 4Å MS (50 mg), chiral ligand **L32** (4.6 mg, 12 mol%), and MeCN (2.0 mL) were added sequentially under argon atmosphere at 100 °C. After addition, the reaction mixture was stirred overnight under these conditions until consumption of the material (monitored by TLC). The solvent was evaporated in vacuo. Then the residues was purified by column chromatography on silica gel without any additional treatment (Hexanes : EtOAc = 50:1 to 10:1) to give the pure products **3** or **4** in good to high yields with generally excellent enantioselectivity.

Condition C:

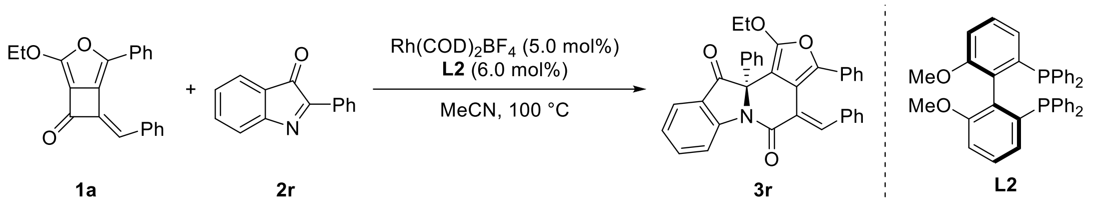

To a 10-mL oven-dried vial containing a magnetic stirring bar, cyclobutanone **1a** (34.8 mg, 0.11 mmol, 1.1 equiv.), imine **2r** (20.8 mg, 0.1 mmol, 1.0 equiv.), Rh(COD)$_2$BF$_4$ (2.0 mg, 5.0 mol%), **L2** (3.5 mg, 6.0 mol%), and MeCN (2.0 mL) were added sequentially under argon atmosphere at 100 ºC. After addition, the reaction mixture was stirred overnight under these conditions until consumption of the material (monitored by TLC). The solvent was evaporated in vacuo. Then the residues were purified by column chromatography on silica gel without any additional treatment (Hexanes: EtOAc = 20:1 to 10:1) to give the pure product **3r**.

Condition D:

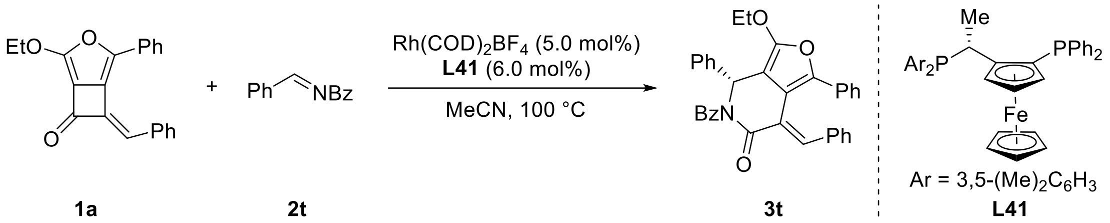

To a 10-mL oven-dried vial containing a magnetic stirring bar, cyclobutanone **1a** (34.8 mg, 0.11 mmol, 1.1 equiv.), imine **2t** (20.9 mg, 0.1 mmol, 1.0 equiv.), Rh(COD)$_2$BF$_4$ (2.0 mg, 5.0 mol%), **L41** (3.8 mg, 6.0 mol%), and MeCN (2.0 mL) were added sequentially under argon atmosphere at 100 ºC. After addition, the reaction mixture was stirred overnight under these conditions until consumption of the material (monitored by TLC). The solvent was evaporated in vacuo. Then the residues were purified by column chromatography on silica gel without any additional treatment (Hexanes: EtOAc = 20:1 to 10:1) to give the pure product **3t**.

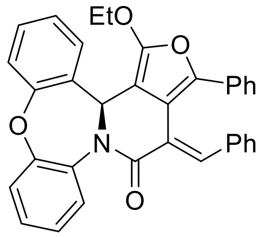

(*S*,*E*)-15-Benzylidene-3-ethoxy-1-phenyl-3Bh-dibenzo[*b*,*f*]furo[3',4':3,4]pyrido[1,2-*d*][1,4]oxazepin-14(15*H*)-one (3a). Condition A: 45.0 mg, 88% yield, **Condition B**: 35.8 mg, 70% yield; 93% *ee*, $[\alpha]_D^{20}$ = 413.0 (c = 0.033, CH_2Cl_2); Yellow solid, m.p. = 105.3 - 107.2 °C; ^{1}H NMR (400 MHz, $CDCl_3$) (δ, ppm) 7.53 (s, 1H), 7.48 (d, *J* = 8.0 Hz, 1H), 7.32 - 7.28 (comp, 3H), 7.25 - 7.17 (comp, 5H), 7.14 - 7.07 (m, 2H), 6.99 - 6.91 (comp, 7H), 6.30 (s, 1H), 4.43 - 4.31 (m, 2H), 1.41 (t, *J* = 7.1 Hz, 3H); ^{13}C NMR (101 MHz, $CDCl_3$) (δ, ppm) 167.9, 154.7, 153.2, 149.9, 139.2, 135.8, 135.6, 131.4, 130.9, 130.8, 129.8, 129.3, 129.0, 128.8, 127.84, 127.80, 127.6, 127.0, 126.0, 125.0, 123.6, 123.1, 122.6, 121.3, 121.0, 115.3, 94.6, 68.7, 54.7, 15.4; HRMS (TOF MS ESI$^+$) calculated for $C_{34}H_{25}NO_4Na[M+Na]^+$: 534.1676, found 534.1675; HPLC conditions for determination of enantiomeric excess: Chiralpak IC, λ = 254 nm, hexane : 2-propanol = 90:10, flow rate = 1.0 mL/min, t_{minor} = 11.7 min, t_{major} = 17.9 min.

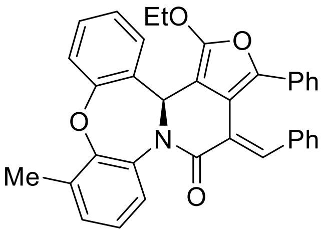

(*S*,*E*)-15-Benzylidene-3-ethoxy-9-methyl-1-phenyl-3b*H*-dibenzo[*b*,*f*]furo[3',4':3,4] pyrido[1,2-*d*][1,4]oxazepin-14(15*H*)-one (3b). Condition A: 47.3 mg, 90% yield, **Condition B**: 32.6 mg, 62% yield; 96% *ee*, $[\alpha]_D^{20}$ = 570.1 (c = 0.033, CH_2Cl_2); Yellow solid, m.p. = 100.6 - 102.5 °C; ^{1}H NMR (400 MHz, $CDCl_3$) (δ, ppm) 7.55 (s, 1H), 7.29 - 7.25 (comp, 3H), 7.21 - 7.16 (comp, 6H), 7.03 (t, *J* = 6.5 Hz, 1H), 6.96 - 6.90 (comp, 7H), 6.16 (s, 1H), 4.47 - 4.35 (m, 2H), 2.46 (s, 3H), 1.43 (t, *J* = 5.6 Hz, 3H); ^{13}C NMR (126 MHz, $CDCl_3$) (δ, ppm) 168.2, 154.4, 153.3, 149.5, 139.2, 135.63, 135.58, 132.4, 130.81, 130.78, 130.5, 129.7, 129.5, 129.0, 128.7, 127.8, 127.5, 126.90,

126.88, 126.7, 125.0, 123.4, 123.1, 122.8, 121.2, 115.3, 95.6, 68.6, 55.1, 17.2, 15.4; HRMS (TOF MS ESI$^+$) calculated for $C_{35}H_{27}NO_4Na[M+Na]^+$: 548.1832, found 548.1829; HPLC conditions for determination of enantiomeric excess: Chiralpak IC, λ = 254 nm, hexane : 2-propanol = 90:10, flow rate = 1.0 mL/min, t_{minor} = 12.1 min, t_{major} = 14.0 min.

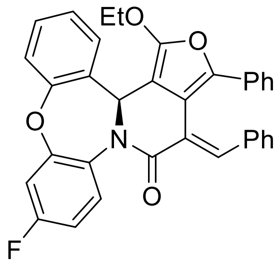

(*S*,*E*)-15-Benzylidene-3-ethoxy-10-fluoro-1-phenyl-3b*H*-dibenzo[*b*,*f*]furo[3',4':3,4]pyrido[1,2-*d*][1,4]oxazepin-14(15*H*)-one (3c). Condition A: 45.5 mg, 86% yield, **Condition B**: 35.5 mg, 67% yield. 95% *ee*, $[\alpha]_D^{20}$ = - 9.0 (c = 0.033, CH_2Cl_2); Yellow oil. ^{1}H NMR (400 MHz, $CDCl_3$) (δ, ppm) 7.53 - 7.52 (m, 1H), 7.45 - 7.42 (m, 1H), 7.31 - 7.29 (m, 2H), 7.26 - 7.12 (comp, 5H), 7.01 - 6.95 (comp, 8H), 6.84 - 6.81 (m, 1H), 6.27 - 6.25 (m, 1H), 4.45 - 4.33 (m, 2H), 1.45 - 1.40 (m, 3H); ^{13}C NMR (126 MHz, $CDCl_3$) (δ, ppm) 168.0, 161.2 (d, *J* = 246.6 Hz), 154.2, 153.2, 150.6 (d, *J* = 12.0 Hz), 139.3, 135.9, 135.5, 131.3, 130.7, 130.4 (d, *J* = 9.8 Hz), 129.9, 129.0, 128.9, 127.9, 127.6, 127.1, 127.0, 126.1, 125.0, 123.9, 122.9, 120.9, 115.2, 109.9 (d, *J* = 22.4 Hz), 108.2 (d, *J* = 25.4 Hz), 94.3, 68.7, 54.6, 15.4; ^{19}F NMR (376 MHz, $CDCl_3$) (δ, ppm) -113.94; HRMS (TOF MS ESI$^+$) calculated for $C_{34}H_{24}FNO_4Na[M+Na]^+$: 552.1582, found 552.1583; HPLC conditions for determination of enantiomeric excess: Chiralpak IC, λ = 254 nm, hexane : 2-propanol = 90:10, flow rate = 1.0 mL/min, t_{minor} = 8.4 min, t_{major} = 10.9 min.

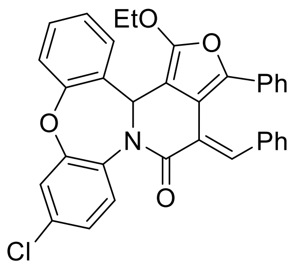

(*E*)-15-Benzylidene-10-chloro-3-ethoxy-1-phenyl-3b*H*-dibenzo[*b,f*]furo[3',4':3,4] pyrido[1,2-*d*][1,4]oxazepin-14(15*H*)-one (3d). Condition A: 42.5 mg, 78% yield; Yellow oil; ^{1}H NMR (500 MHz, $CDCl_3$) (δ, ppm) 7.52 (s, 1H), 7.42 (d, *J* = 10.0 Hz, 1H), 7.31 - 7.30 (comp, 3H), 7.25 - 7.14 (comp, 5H), 7.13 - 7.11 (m, 1H), 7.07 - 7.05 (m, 1H), 6.99 - 6.92 (comp, 6H), 6.28 (s, 1H), 4.44 - 4.31 (m, 2H), 1.42 (t, *J* = 7.0 Hz, 3H); ^{13}C NMR (126 MHz, $CDCl_3$) (δ, ppm) 167.8, 154.3, 153.2, 145.0, 139.4, 136.1, 135.5, 132.5, 131.6, 130.7, 130.2, 123.0, 129.4, 129.0, 128.9, 127.9, 127.6 127.0, 125.9, 125.0, 124.1, 122.9, 122.7, 121.4, 121.0, 115.2, 94.1, 68.7, 54.4, 15.4; HRMS (TOF MS ESI$^+$) calculated for $C_{34}H_{24}ClNO_4Na[M+Na]^+$: 568.1286, found 568.1288.

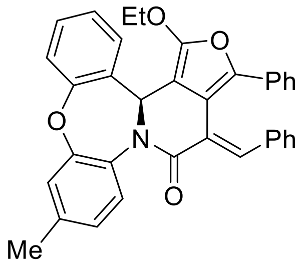

(*S,E*)-15-Benzylidene-10-chloro-3-ethoxy-1-phenyl-3b*H*-dibenzo[*b,f*]furo[3',4':3,4]pyrido[1,2-*d*][1,4]oxazepin-14(15*H*)-one (3e). Condition A: 44.7 mg, 85% yield, **Condition B**: 38.7 mg, 71% yield. 93% *ee*, $[\alpha]_D^{20}$ = 162.0 (c = 0.033, CH_2Cl_2); Yellow oil. ^{1}H NMR (500 MHz, $CDCl_3$) (δ, ppm) 7.53 (s, 1H), 7.35 (d, *J* = 8.1 Hz, 1H), 7.30 (d, *J* = 7.4 Hz, 2H), 7.21 - 7.20 (comp, 3H), 7.17 - 7.10 (comp, 3H), 6.98 - 6.90 (comp, 8H), 6.27 (s, 1H), 4.43 - 4.30 (m, 2H), 2.36 (s, 3H), 1.41 (t, *J* = 7.0 Hz, 3H); ^{13}C NMR (126 MHz, $CDCl_3$) (δ, ppm) 167.8, 154.7, 153.2, 149.5, 139.2, 138.0, 135.7, 135.6, 131.4, 130.8, 129.7, 129.0, 128.8, 128.7, 128.3, 127.8, 127.5, 126.9, 126.1, 125.0, 123.5, 123.2, 121.5, 120.9, 115.4, 94.7, 68.7, 54.7, 21.0, 15.4; HRMS (TOF MS ESI$^+$) calculated for $C_{35}H_{27}NO_4Na[M+Na]^+$: 548.1832, found 548.1828;

HPLC conditions for determination of enantiomeric excess: Chiralpak IC, λ = 254 nm, hexane : 2-propanol = 90:10, flow rate = 1.0 mL/min, t_{minor} = 12.4 min, t_{major} = 20.4 min.

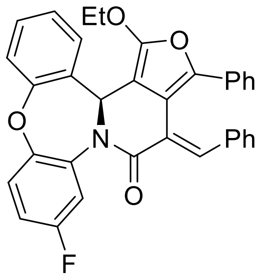

(*S*,*E*)-15-Benzylidene-3-ethoxy-11-fluoro-1-phenyl-3b*H*-dibenzo[*b*,*f*]furo[3',4':3,4]pyrido[1,2-*d*][1,4]oxazepin-14(15*H*)-one (3f). Condition A: 47.1 mg, 89% yield, **Condition B**: 38.7 mg, 70% yield; 90% *ee*, $[\alpha]_D^{20}$ = -3.0 (c = 0.033, CH_2Cl_2); Yellow oil; ^1H NMR (400 MHz, $CDCl_3$) (δ, ppm) 7.54 (s, 1H), 7.32 - 7.30 (m, 2H), 7.26 - 7.11 (comp, 7H), 6.96 - 6.94 (comp, 8H), 6.31 (s, 1H), 4.45 - 4.31 (m, 2H), 1.44 - 1.40 (m, 3H); ^{13}C NMR (126 MHz, $CDCl_3$) (δ, ppm) 167.8, 157.3 (d, J = 242.0 Hz), 154.6, 153.2, 146.14 (d, J = 2.6 Hz), 139.3, 136.1, 135.4, 131.7, 130.7, 129.9, 129.0, 128.9, 127.9, 127.6, 127.0, 126.0, 125.0, 123.9, 122.9, 122.1 (d, J = 8.9 Hz), 120.9, 115.7 (d, J = 25.6 Hz), 115.1, 114.7 (d, J = 23.3 Hz), 94.2, 68.7, 54.3, 15.4; ^{19}F NMR (376 MHz, $CDCl_3$) (δ, ppm) -120.22; HRMS (TOF MS ESI^+) calculated for $C_{34}H_{24}FNO_4Na[M+Na]^+$: 552.1582, found 552.1580; HPLC conditions for determination of enantiomeric excess: Chiralpak IC, λ = 254 nm, hexane : 2-propanol = 90:10, flow rate = 1.0 mL/min, t_{minor} = 11.4 min, t_{major} = 13.7 min.

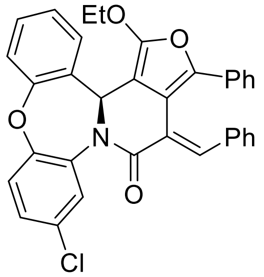

(*S*,*E*)-15-Benzylidene-11-chloro-3-ethoxy-1-phenyl-3b*H*-dibenzo[*b*,*f*]furo[3',4':3,4]pyrido[1,2-*d*][1,4]oxazepin-14(15*H*)-one (3g). Condition A: 45.3 mg, 83% yield,

Condition B: 35.5 mg, 65% yield; 90% *ee*, $[\alpha]_D^{20}$ = 420.0 (c = 0.033, CH_2Cl_2); Yellow oil; ^1H NMR (400 MHz, $CDCl_3$) (δ, ppm) 7.54 (s, 1H), 7.50 (d, *J* = 2.1 Hz, 1H), 7.31 - 7.29 (m, 2H), 7.25 - 7.17 (comp, 6H), 7.15 - 7.11(m, 1H), 7.00 - 6.91 (comp, 7H), 6.28 (s, 1H), 4.45 - 4.30 (m, 2H), 1.42 (t, *J* = 7.1 Hz, 3H); ^{13}C NMR (101 MHz, $CDCl_3$) (δ, ppm) 167.8, 154.4, 153.2, 148.5, 139.4, 136.2, 135.4, 131.5, 131.4, 130.7, 129.9, 129.0, 128.9, 127.9, 127.7, 127.6, 127.3, 127.1, 126.0, 125.0, 123.9, 122.8, 122.4, 120.9, 115.1, 94.0, 68.7, 54.5, 15.4; HRMS (TOF MS ESI^+) calculated for $C_{34}H_{24}ClNO_4Na[M+Na]^+$: 568.1286, found 568.1291; HPLC conditions for determination of enantiomeric excess: Chiralpak IC, λ = 254 nm, hexane : 2-propanol = 90:10, flow rate = 1.0 mL/min, t_{minor} = 9.9 min, t_{major} = 12.5 min.

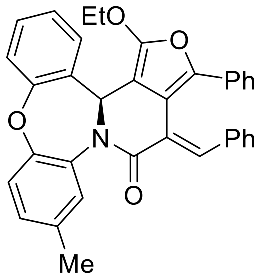

(*S*,*E*)-15-Benzylidene-3-ethoxy-11-methyl-1-phenyl-3b*H*-dibenzo[*b,f*]furo[3',4':3,4]pyrido[1,2-*d*][1,4]oxazepin-14(15*H*)-one (3h). Condition A: 42.0 mg, 80% yield, **Condition B**: 36.3 mg, 69% yield; 91% *ee*, $[\alpha]_D^{20}$ = 123.0 (c = 0.033, CH_2Cl_2); Yellow oil; ^1H NMR (500 MHz, $CDCl_3$) (δ, ppm) 7.55 (s, 1H), 7.30 - 7.29 (comp, 3H), 7.22 - 7.11 (comp, 5H), 7.12 (d, *J* = 7.6 Hz, 1H), 7.03 (d, *J* = 8.3 Hz, 1H), 6.98 - 6.91 (comp, 7H), 6.26 (s, 1H), 4.43 - 4.30 (m, 2H), 2.33 (s, 3H), 1.41 (t, *J* = 7.0 Hz, 3H); ^{13}C NMR (126 MHz, $CDCl_3$) (δ, ppm) 167.8, 154.7, 153.2, 147.9, 139.2, 135.8, 135.6, 132.2, 131.2, 130.8, 130.6, 129.7, 129.3, 129.0, 128.8, 128.6, 127.8, 127.5, 126.9, 126.1, 125.0, 123.4, 123.1, 121.0, 120.9, 115.3, 94.7, 68.7, 54.8, 20.7, 15.4; HRMS (TOF MS ESI^+) calculated for $C_{35}H_{27}NO_4Na[M+Na]^+$: 548.1832, found 548.1824; HPLC conditions for determination of enantiomeric excess: Chiralpak IC, λ = 254 nm, hexane : 2-propanol = 90:10, flow rate = 1.0 mL/min, t_{minor} = 10.3 min, t_{major} = 15.6 min.

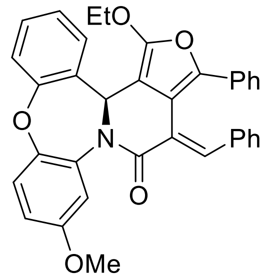

(*S*,*E*)-15-Benzylidene-3-ethoxy-11-methoxy-1-phenyl-3b*H*-dibenzo[*b*,*f*]furo[3',4':3,4]pyrido[1,2-*d*][1,4]oxazepin-14(15*H*)-one (3i). Condition A: 48.2 mg, 89% yield, **Condition B**: 37.9 mg, 70% yield; 90% *ee*, $[\alpha]_D^{20}$ = -6.0 (c = 0.033, CH_2Cl_2); Yellow oil; ^{1}H NMR (400 MHz, $CDCl_3$) (δ, ppm) 7.56 (s, 1H), 7.30 (d, *J* = 7.2 Hz, 2H), 7.22 - 7.18 (comp, 4H), 7.17 - 7.12 (m, 2H), 7.01 (d, *J* = 2.9 Hz, 1H), 6.97 - 6.91 (comp, 7H), 6.81 (dd, *J* = 9.0, 3.0 Hz, 1H), 6.28 (s, 1H), 4.45 - 4.30 (m, 2H), 3.79 (s, 3H), 1.42 (t, *J* = 7.1 Hz, 3H); ^{13}C NMR (101 MHz, $CDCl_3$) (δ, ppm) 168.0, 154.8, 154.6, 153.2, 144.2, 139.2, 135.9, 135.5, 131.4, 131.3, 130.8, 129.7, 129.0, 128.8, 127.8, 127.5, 127.0, 126.1, 125.0, 123.4, 123.0, 121.9, 120.9, 115.2, 115.1, 112.6, 94.7, 68.7, 55.9, 54.7, 15.4; HRMS (TOF MS ESI$^+$) calculated for $C_{35}H_{27}NO_4Na[M+Na]^+$: 564.1781, found 564.1785; HPLC conditions for determination of enantiomeric excess: Chiralpak IC, λ = 254 nm, hexane : 2-propanol = 90:10, flow rate = 1.0 mL/min, t_{minor} = 21.5 min, t_{major} = 33.6 min.

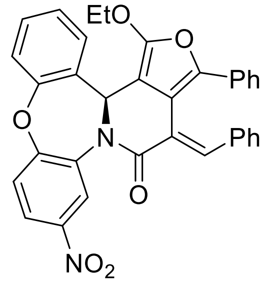

(*S*,*E*)-15-Benzylidene-3-ethoxy-11-nitro-1-phenyl-3b*H*-dibenzo[*b*,*f*]furo[3',4':3,4]pyrido[1,2-*d*][1,4]oxazepin-14(15*H*)-one (3j). Condition A: 45.1 mg, 81% yield, **Condition B**: 45.1 mg, 81% yield; 90% *ee*, $[\alpha]_D^{20}$ = 147.0 (c = 0.033, CH_2Cl_2); Yellow solid, m.p. = 242.3 - 244.2 °C; ^{1}H NMR (500 MHz, $CDCl_3$) (δ, ppm) 8.45 (d, *J* = 2.5 Hz, 1H), 8.09 - 8.07 (m, 1H), 7.55 (s, 1H), 7.35 (d, *J* = 9.1 Hz, 1H), 7.32 - 7.31 (m, 2H), 7.29 - 7.25 (m, 1H), 7.21 - 7.19 (comp, 3H), 7.15 (d, *J* = 7.5 Hz, 1H),

7.03 - 6.93 (comp, 7H), 6.34 (s, 1H), 4.46 - 4.33 (m, 2H), 1.42 (t, J = 7.1 Hz, 3H); ^{13}C NMR (126 MHz, $CDCl_3$) (δ, ppm) 167.8, 154.3, 153.7, 153.2, 142.1, 139.6, 136.6, 135.2, 131.5, 130.6, 130.4, 130.2, 129.1, 129.0, 127.9, 127.6, 127.2, 125.9, 125.8, 125.0, 124.7, 122.7, 122.3, 122.0, 120.9, 114.8, 93.1, 68.7, 54.3, 15.3. HRMS (TOF MS ESI$^+$) calculated for $C_{34}H_{24}N_2O_6Na[M+Na]^+$: 579.1527, found 579.1527; HPLC conditions for determination of enantiomeric excess: Chiralpak IC, λ = 254 nm, hexane : 2-propanol = 80:20, flow rate = 1.0 mL/min, t_{minor} = 25.5 min, t_{major} = 28.9 min.

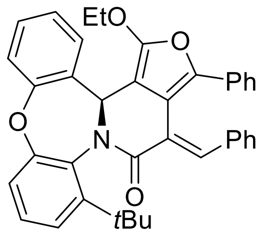

(*S*,*E*)-15-Benzylidene-12-(*tert*-butyl)-3-ethoxy-1-phenyl-3b*H*-dibenzo[*b,f*]furo[3',4':3,4]pyrido[1,2-*d*][1,4]oxazepin-14(15*H*)-one (3k). Condition A: 53.9 mg, 95% yield, **Condition B**: 29.5 mg, 52% yield; 99% *ee*, $[\alpha]_D^{20}$ = 261.1 (c = 0.033, CH_2Cl_2); Yellow oil; ^{1}H NMR (500 MHz, $CDCl_3$) (δ, ppm) 7.54 - 7.49 (m, 2H), 7.30 - 7.27 (m, 2H), 7.24 - 7.10 (comp, 8H), 6.95 - 6.92 (comp, 6H), 6.34 - 6.31 (m, 1H), 4.45 - 4.29 (m, 2H), 1.43 - 1.38 (m, 3H), 1.34 - 1.31 (m, 9H); ^{13}C NMR (101 MHz, $CDCl_3$) (δ, ppm) 167.9, 154.8, 153.2, 147.4, 145.2, 139.2, 135.7, 135.6, 131.6, 130.8, 129.8, 129.7, 128.9, 128.8, 127.8, 127.5, 126.9, 126.2, 125.9, 125.0, 124.9, 123.5, 123.3, 120.9, 120.6, 115.4, 94.5, 68.7, 54.6, 34.5, 31.5, 15.4; HRMS (TOF MS ESI$^+$) calculated for $C_{38}H_{33}N_1O_4Na[M+Na]^+$: 590.2302, found 590.2307; HPLC conditions for determination of enantiomeric excess: Chiralpak IC, λ = 254 nm, hexane : 2-propanol = 80:20, flow rate = 1.0 mL/min, t_{minor} = 8.1 min, t_{major} = 9.3 min.

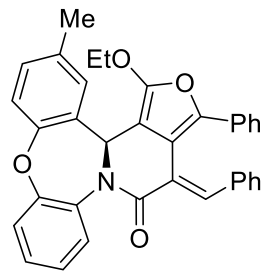

(*S*,*E*)-15-Benzylidene-3-ethoxy-5-methyl-1-phenyl-3b*H*-dibenzo[*b,f*]furo[3',4':3,4] pyrido[1,2-*d*][1,4]oxazepin-14(15*H*)-one (3l). Condition A: 48.4 mg, 92% yield, **Condition B**: 34.7 mg, 66% yield; 93% *ee*, $[\alpha]_D^{20}$ = 432.0 (c = 0.033, CH_2Cl_2); Yellow oil; ^1H NMR (400 MHz, $CDCl_3$) (δ, ppm) 7.51 (s, 1H), 7.47 - 7.45 (m, 1H), 7.33 (d, J = 6.6 Hz, 2H), 7.27 - 7.22 (comp, 4H), 7.09 - 7.05 (m, 2H), 7.00 - 6.90 (comp, 8H), 6.29 (s, 1H), 4.46 - 4.30 (m, 2H), 2.12 (s, 3H), 1.42 (t, J = 7.1 Hz, 3H); ^{13}C NMR (126 MHz, $CDCl_3$) (δ, ppm) 167.9, 153.1, 152.5, 149.8, 139.2, 135.8, 135.7, 133.2, 131.4, 130.7, 130.6, 130.1, 129.3, 128.9, 128.8, 127.9, 127.7, 127.6, 127.0, 126.4, 124.9, 123.5, 122.3, 121.2, 120.6, 115.5, 94.5, 68.8, 54.5, 20.8, 15.4; HRMS (TOF MS ESI^+) calculated for $C_{35}H_{27}NO_4Na[M+Na]^+$: 548.1832, found: 548.1824; HPLC conditions for determination of enantiomeric excess: Chiralpak IC, λ = 254 nm, hexane : 2-propanol = 90:10, flow rate = 1.0 mL/min, t_{minor} = 11.7 min, t_{major} = 18.1 min.

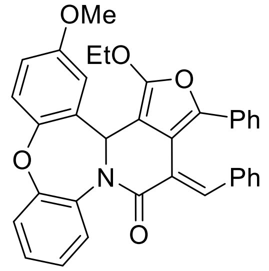

(*E*)-15-Benzylidene-3-ethoxy-5-methoxy-1-phenyl-3b*H*-dibenzo[*b,f*]furo[3',4':3,4] pyrido[1,2-*d*][1,4]oxazepin-14(15*H*)-one (3m). Condition A: 46.6 mg, 86% yield; Yellow oil; ^1H NMR (400 MHz, $CDCl_3$) (δ, ppm) 7.58 -7.57 (m, 1H), 7.49 - 7.47 (m, 1H), 7.36 - 7.34 (m, 2H), 7.26 - 7.21 (comp, 4H), 7.13 - 7.06 (m, 2H), 7.00 - 6.95 (comp, 6H), 6.74 - 6.65 (m, 2H), 6.31 (d, J = 3.5 Hz, 1H), 4.45 - 4.31 (m, 2H), 3.61 - 3.54 (m, 3H), 1.46 - 1.39 (m, 3H); ^{13}C NMR (101 MHz, $CDCl_3$) (δ, ppm) 168.0,

154.8, 154.6, 153.2, 144.2, 139.2, 135.9, 135.5, 131.4, 131.3, 130.8, 129.7, 129.0, 128.8, 127.8, 127.5, 127.0, 126.1, 125.0, 123.4, 123.0, 121.9, 120.9, 115.2, 115.1, 112.6, 94.7, 68.7, 55.9, 54.7, 15.4; HRMS (TOF MS ESI$^+$) calculated for $C_{35}H_{27}NO_5Na[M+Na]^+$: 564.1781, found: 564.1785.

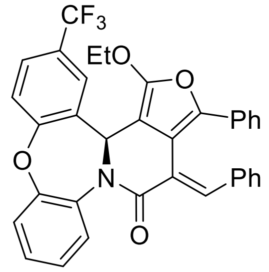

(*S*,*E*)-15-Benzylidene-3-ethoxy-1-phenyl-5-(trifluoromethyl)-3b*H*-dibenzo[*b,f*]furo[3',4':3,4]pyrido[1,2-*d*][1,4]oxazepin-14(15*H*)-one (3n). Condition A: 49.3 mg, 85% yield, **Condition B**: 34.8 mg, 60% yield; 90% *ee*, $[\alpha]_D^{20}$ = 363.0 (c = 0.033, CH_2Cl_2); Yellow oil; ^{1}H NMR (400 MHz, $CDCl_3$) (δ, ppm) 7.53 - 7.52 (m, 1H), 7.49 - 7.46 (m, 2H), 7.39 (s, 1H), 7.28 - 7.22 (comp, 7H), 7.16 - 7.11 (m, 1H), 6.98 - 6.91 (comp, 6H), 6.25 (d, *J* = 2.2 Hz, 1H), 4.50 - 4.33 (m, 2H), 1.45 - 1.40 (m, 3H).; ^{13}C NMR (101 MHz, $CDCl_3$) (δ, ppm) 167.8, 157.1, 153.3, 149.6, 139.5, 136.6, 135.3, 131.7, 131.0, 130.5, 129.4, 129.0, 128.8, 128.2, 127.9, 127.6, 127.1, 127.0 (q, *J* = 3.6 Hz), 125.3, 125.0, 123.8 (q, *J* = 3.4 Hz), 123.3, 122.7, 121.5, 121.3, 120.2 (q, *J* = 306.3 Hz), 114.9, 93.6, 68.6, 54.7, 15.3; ^{19}F NMR (376 MHz, $CDCl_3$) (δ, ppm) -61.83; HRMS (TOF MS ESI$^+$) calculated for $C_{35}H_{24}F_3NO_4Na[M+Na]^+$: 602.1550, found: 602.1552; HPLC conditions for determination of enantiomeric excess: Chiralpak IC, λ = 254 nm, hexane : 2-propanol = 90:10, flow rate = 1.0 mL/min, t_{minor} = 7.9 min, t_{major} = 12.5min.

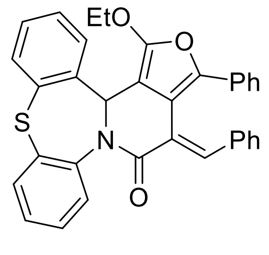

(*E*)-15-Benzylidene-3-ethoxy-1-phenyl-3b*H*-dibenzo[*b,f*]furo[3',4':3,4]pyrido[1,2-

***d*][1,4]thiazepin-14(15*H*)-one (3o). Condition A**: 29.5 mg, 56% yield; Yellow oil; ^{1}H NMR (500 MHz, $CDCl_3$) (δ, ppm) 7.50 (s, 1H), 7.45 - 7.39 (comp, 3H), 7.35 - 7.33 (m, 2H) , 7.34 (d, *J* = 7.8 Hz, 2H), 7.25 - 7.13 (comp, 6H), 7.06 (t, *J* = 7.5 Hz, 1H), 7.00 - 6.93 (comp, 6H), 6.37 (s, 1H), 4.45 - 4.30 (m, 2H), 1.42 (t, *J* = 7.0 Hz, 3H); ^{13}C NMR (126 MHz, $CDCl_3$) (δ, ppm) 169.0, 152.6, 142.0, 141.1, 139.1, 135.54, 135.48, 133.1, 130.7, 130.0, 129.4, 129.2, 129.1, 128.9, 128.7, 128.3, 127.9, 127.6, 127.1, 127.0, 126.9, 126.7, 126.2, 124.9, 124.1, 116.0, 96.3, 68.7, 55.9, 15.4; HRMS (TOF MS ESI^{+}) calculated for $C_{34}H_{25}NO_3SNa[M+Na]^{+}$: 550.1447, found: 550.1448.

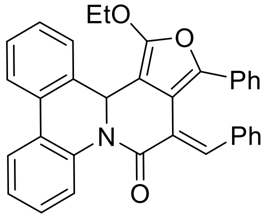

(*E*)-7-Benzylidene-10-ethoxy-8-phenyl-10b*H*-furo[3',4':3,4]pyrido[1,2-*f*]phenanthridin-6(7*H*)-one (3p). Condition A: 39.6 mg, 80% yield; Yellow oil. ^{1}H NMR (500 MHz, $CDCl_3$) (δ, ppm) 7.90 (d, *J* = 7.7 Hz, 1H), 7.81 (d, *J* = 6.5 Hz, 2H), 7.62 (s, 1H), 7.49 - 7.39 (comp, 3H), 7.37 - 7.34 (m, 1H), 7.30 - 7.29 (m, 1H), 7.20 - 7.18 (comp, 4H), 6.96 - 6.90 (comp, 4H), 6.89 - 6.84 (m, 2H), 5.64 (s, 1H), 4.45 - 4.35 (m, 2H), 1.40 (t, *J* = 7.0 Hz, 3H); ^{13}C NMR (126 MHz, $CDCl_3$) (δ, ppm) 166.1, 154.8, 139.4, 138.9, 136.6, 135.8, 135.3, 132.7, 131.2, 129.6, 129.0, 128.6, 128.5, 128.2, 128.1, 127.6, 127.5, 126.9, 126.79, 126.76, 125.3, 124.4, 124.3, 123.8, 122.3, 114.4, 92.3, 68.5, 53.9, 29.9; HRMS (TOF MS ESI^{+}) calculated for $C_{34}H_{25}NO_3Na[M+Na]^{+}$: 550.1447, found: 550.1448.

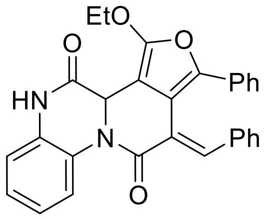

(*E*)-12-Benzylidene-3-ethoxy-1-phenyl-5*H*-furo[3',4':3,4]pyrido[1,2-*a*]quinoxaline-4,11(3b*H*,12*H*)-dione (3q). Condition A: 32.4 mg, 70% yield; Yellow oil; ^{1}H NMR

(400 MHz, $CDCl_3$) (δ, ppm) 8.53 (s, 1H), 7.81 - 7.78 (m, 1H), 7.76 (s, 1H), 7.24 - 7.18 (comp, 4H), 7.12 - 7.10 (m, 2H), 7.00 - 6.97 (m, 1H), 6.96 - 6.90 (comp, 4H), 6.88 - 6.83 (m, 2H), 5.10 (s, 1H), 4.49 - 4.37 (m, 2H), 1.43 (t, J = 7.1 Hz, 3H); ^{13}C NMR (126 MHz, $CDCl_3$) (δ, ppm) 166.7, 166.1, 155.7, 139.2, 136.3, 135.4, 131.2, 131.0, 129.3, 129.2, 128.8, 127.6, 127.5, 127.3, 126.9, 126.6, 125.4, 123.5, 121.2, 116.6, 113.7, 88.5, 68.9, 54.6, 15.4; HRMS (TOF MS ESI^+) calculated for $C_{29}H_{22}N_2O_4Na[M+Na]^+$: 485.1472, found: 485.1468.

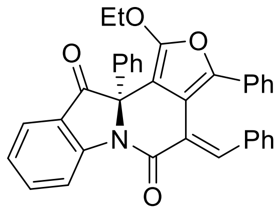

(*R*,*E*)-4-Benzylidene-1-ethoxy-3,11a-diphenylfuro[3',4':3,4]pyrido[1,2-*a*]indole-5,11(4*H*,11*aH*)-dione (3r). Condition A: 41.8 mg, 80% yield, **Condition C**: 44.5 mg, 85% yield; >99% *ee*; Yellow oil; ^1H NMR (400 MHz, $CDCl_3$) (δ, ppm) 8.75 (d, J = 8.3 Hz, 1H), 7.82 - 7.77 (m, 2H), 7.49 (s, 1H), 7.44 - 7.42 (m, 2H), 7.30 - 7.24 (comp, 3H), 7.20 - 7.19 (m, 1H), 7.16 - 7.15 (m, 2H), 7.08 (d, J = 7.4 Hz, 2H), 6.96 - 6.88 (comp, 4H), 6.86 - 6.82 (m, 2H), 4.54 - 4.39 (m, 2H), 1.54 (t, J = 7.1 Hz, 3H); ^{13}C NMR (101 MHz, $CDCl_3$) (δ, ppm) 192.9, 166.8, 153.2, 152.2, 139.6, 138.7, 137.7, 136.2, 135.1, 130.5, 129.1, 128.5, 127.8, 127.6, 127.2, 125.6, 125.5, 125.1, 125.0, 124.3, 122.4, 117.3, 115.2, 97.6, 71.0, 70.0, 29.8, 15.5, 15.4; HRMS (TOF MS ESI^+) calculated for $C_{35}H_{25}NO_4Na[M+Na]^+$: 546.1676, found: 546.1672; HPLC conditions for determination of enantiomeric excess: Chiralpak IC, λ = 254 nm, hexane : 2-propanol = 95:5, flow rate = 1.0 mL/min, t_{minor} = 8.7 min, t_{major} = 7.5 min.

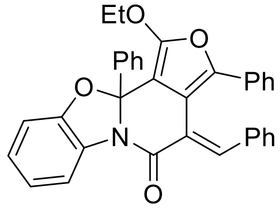

(*E*)-4-Benzylidene-1-ethoxy-3,11a-diphenyl-11a*H*-benzo[4,5]oxazolo[3,2-*a*]furo[3,

4-*c*]pyridin-5(4*H*)-one (3s). Condition A: 20.5 mg, 40% yield; Yellow oil. ^{1}H NMR (500 MHz, $CDCl_3$) (δ, ppm) 8.11 - 8.08 (m, 1H), 7.78 - 7.77 (m, 1H), 7.62 - 7.61 (m, 2H), 7.32 - 7.26 (comp, 3H), 7.19 - 7.18 (m, 2H), 7.11 - 7.10 (m, 2H), 7.07 - 7.04 (m, 1H), 7.02 - 7.00 (m, 1H), 6.98 - 6.95 (comp, 3H), 6.92 - 6.84 (comp, 4H), 4.53 - 4.44 (m, 2H), 1.56 - 1.51 (m, 3H); ^{13}C NMR (126 MHz, $CDCl_3$) (δ, ppm) 164.6, 152.5, 149.7, 141.9, 139.7, 136.3, 135.2, 130.7, 129.8, 129.12, 129.08, 129.0, 128.83, 127.75, 127.5, 127.3, 125.5, 125.4, 124.8, 122.5, 122.1, 116.6, 112.0, 109.7, 100.8, 96.4, 69.8, 15.4; HRMS (TOF MS ESI$^+$) calculated for $C_{34}H_{25}NO_4Na[M+Na]^+$: 534.1676, found: 534.1679.

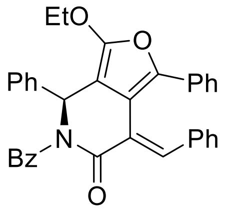

(*R*,*E*)-5-Benzoyl-7-benzylidene-3-ethoxy-1,4-diphenyl-4,5-dihydrofuro[3,4-*c*]pyridin-6(7*H*)-one (3t). **Condition D:** Colourless oil; 28.9 mg, 55% yield; 80% *ee*; ^{1}H NMR (400 MHz, $CDCl_3$) (δ, ppm) 7.62 - 7.61 (comp, 3H), 7.50 - 7.48 (comp, 3H), 7.44 - 7.40 (m, 2H), 7.31 - 7.28 (comp, 3H), 7.23 - 7.19 (m, 2H), 7.18 - 7.15 (comp, 3H), 6.97 - 6.93 (comp, 3H), 6.90 - 6.86 (m, 2H), 6.62 (s, 1H), 4.49 - 4.35 (m, 2H), 1.47 (t, J = 7.1 Hz, 3H); ^{13}C NMR (101 MHz, $CDCl_3$) (δ, ppm) 174.1, 170.2, 153.0, 140.7, 139.7, 138.7, 136.2, 135.0, 131.6, 130.8, 129.5, 129.2, 128.8, 128.4, 128.1, 127.8, 127.6, 127.1, 126.7, 125.0, 124.9, 122.7, 113.9, 98.6, 68.8, 53.3, 15.4; HRMS (TOF MS ESI$^+$) calculated for $C_{35}H_{27}NO_4Na[M+Na]^+$: 548.1832, found: 548.1836; HPLC conditions for determination of enantiomeric excess: Chiralpak IC, λ = 254 nm, hexane : 2-propanol = 95:5, flow rate = 1.0 mL/min, t_{minor} = 11.4 min, t_{major} = 13.7 min.

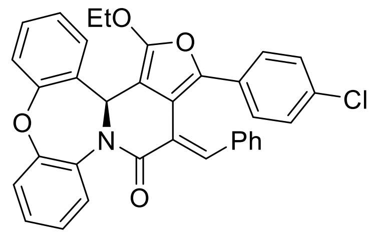

(*S*,*E*)-15-Benzylidene-1-(4-chlorophenyl)-3-ethoxy-3b*H*-dibenzo[*b*,*f*]furo[3',4':3,4]pyrido[1,2-*d*][1,4]oxazepin-14(15*H*)-one (4a). Condition A: 44.8 mg, 82% yield, **Condition B**: 38.7 mg, 71% yield; 90% *ee*, $[\alpha]_D^{20}$ = 33.0 (c = 0.033, CH_2Cl_2); Yellow oil; ^{1}H NMR (400 MHz, $CDCl_3$) (δ, ppm) 7.54 (s, 1H), 7.46 (d, *J* = 8.0 Hz, 1H), 7.30 - 7.28 (comp, 3H), 7.25 - 7.17 (comp, 3H), 7.12 - 7.07 (comp, 4H), 7.02 - 6.95 (comp, 4H), 6.91 - 6.90 (m, 2H), 6.29 (s, 1H), 4.42 - 4.29 (m, 2H), 1.41 (t, *J* = 6.3 Hz, 3H); ^{13}C NMR (101 MHz, $CDCl_3$) (δ, ppm) 167.5, 154.6, 153.2, 149.8, 137.9, 136.0, 135.3, 133.4, 132.5, 131.2, 130.7, 129.7, 129.1, 128.8, 128.0, 127.8, 127.6, 126.0, 125.8, 123.5, 123.0, 122.5, 121.4, 121.2, 120.9, 115.9, 94.6, 68.7, 54.5, 15.2; HRMS (TOF MS ESI$^+$) calculated for $C_{34}H_{24}ClNO_4Na[M+Na]^+$: 568.1286, found: 568.1290; HPLC conditions for determination of enantiomeric excess: Chiralpak IC, λ = 254 nm, hexane : 2-propanol = 90:10, flow rate = 1.0 mL/min, t_{minor} = 12.6 min, t_{major} = 17.9min.

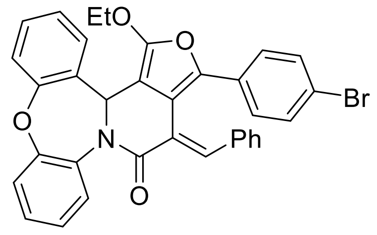

(*E*)-15-Benzylidene-1-(4-bromophenyl)-3-ethoxy-3b*H*-dibenzo[*b*,*f*]furo[3',4':3,4]pyrido[1,2-*d*][1,4]oxazepin-14(15*H*)-one (4b). Condition A: 41.3 mg, 70% yield; Yellow solid, m.p. = 203.2 - 204.6 °C; ^{1}H NMR (500 MHz, $CDCl_3$) (δ, ppm) 7.55 (s, 1H), 7.46 (d, *J* = 7.9 Hz, 1H), 7.30 - 7.28 (comp, 3H), 7.24 - 7.21 (m, 2H), 7.19 - 7.18 (m, 1H), 7.12 - 7.09 (m, 2H), 7.08 - 7.02 (comp, 5H), 7.00 - 6.94 (comp, 3H), 6.29 (s, 1H), 4.44 - 4.30 (m, 2H), 1.41 (t, *J* = 7.1 Hz, 3H); ^{13}C NMR (126 MHz, $CDCl_3$) (δ, ppm) 167.6, 154.7, 153.4, 149.9, 138.0, 136.1, 135.4, 131.3, 130.8, 130.7, 129.8, 129.6, 129.23, 129.20, 128.9, 128.1, 127.9, 126.4, 126.0, 123.6, 123.1, 122.6, 121.4,

121.0, 120.7, 116.1, 94.7, 68.8, 54.7, 15.4; HRMS (TOF MS ESI$^+$) calculated for $C_{34}H_{24}BrNO_4Na[M+Na]^+$: 612.0781, found: 612.0780.

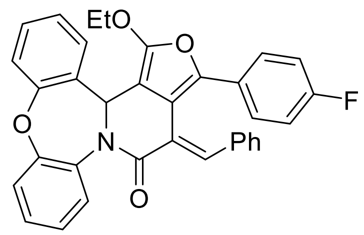

(*E*)-15-Benzylidene-3-ethoxy-1-(4-fluorophenyl)-3b*H*-dibenzo[*b,f*]furo[3',4':3,4]pyrido[1,2-*d*][1,4]oxazepin-14(15*H*)-one (4c). Condition A: 47.1 mg, 89% yield; Yellow solid, m.p. = 251.6 - 252.7 °C; ^{1}H NMR (500 MHz, $CDCl_3$) (δ, ppm) 7.54 (s, 1H), 7.47 (d, *J* = 7.9 Hz, 1H), 7.30 - 7.28 (comp, 3H), 7.25 - 7.21 (m, 2H), 7.19 - 7.15 (comp, 3H), 7.13 - 7.08 (m, 2H), 7.03 - 7.00 (m, 1H), 6.97 - 6.94 (comp, 3H), 6.64 (t, *J* = 8.7 Hz, 2H), 6.30 (s, 1H), 4.43 - 4.29 (m, 2H), 1.41 (t, *J* = 7.1 Hz, 3H); ^{13}C NMR (126 MHz, $CDCl_3$) (δ, ppm) 167.7, 161.8 (d, *J* = 247.8 Hz), 154.7, 153.1, 149.9, 138.4, 135.7, 135.4, 131.4, 130.9, 129.8, 129.2, 129.04, 128.96, 128.0, 127.8, 127.1 (d, *J* = 3.1 Hz), 126.8 (d, *J* = 8.2 Hz), 126.0, 123.6, 123.1, 122.6, 121.3, 121.0, 115.1, 114.6 (d, *J* = 21.9 Hz), 94.6, 68.8, 54.7, 15.4; ^{19}F NMR (376 MHz, $CDCl_3$) (δ, ppm) -114.07; HRMS (TOF MS ESI$^+$) calculated for $C_{34}H_{24}FNO_4Na[M+Na]^+$: 552.1582, found: 552.1582.

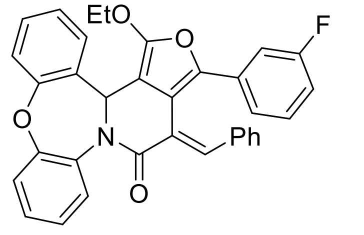

(*E*)-15-Benzylidene-3-ethoxy-1-(3-fluorophenyl)-3b*H*-dibenzo[*b,f*]furo[3',4':3,4]pyrido[1,2-*d*][1,4]oxazepin-14(15*H*)-one (4d). Condition A: 40.2 mg, 76% yield; Yellow solid, m.p. = 236.6 - 237.7 °C; ^{1}H NMR (500 MHz, $CDCl_3$) (δ, ppm) 7.57 (s, 1H), 7.47 (d, *J* = 7.9 Hz, 1H), 7.31 - 7.28 (comp, 3H), 7.26 - 7.18 (comp, 3H), 7.13 - 7.08 (m, 2H), 7.02 - 6.95 (comp, 5H), 6.89 - 6.86 (m, 2H), 6.66 (t, *J* = 8.0 Hz, 1H), 6.30 (s, 1H), 4.45 - 4.31 (m, 2H), 1.42 (t, *J* = 7.0 Hz, 3H); ^{13}C NMR (126 MHz,

$CDCl_3$) (δ, ppm) 167.6, 162.1 (d, *J* = 244.6 Hz), 154.7, 153.4, 149.9, 137.7 (d, *J* = 3.1 Hz), 136.4, 135.6, 132.6 (d, *J* = 8.7 Hz), 131.3, 130.8, 129.8, 129.2, 129.1 (d, *J* = 8.4 Hz), 128.9, 128.8, 128.0, 127.9, 126.0, 123.6, 123.0, 122.6, 121.3, 121.0, 120.7 (d, *J* = 2.7 Hz), 116.4, 113.7 (d, *J* = 21.3 Hz), 111.8 (d, *J* = 23.7 Hz), 94.6, 68.7, 54.7, 15.3; ^{19}F NMR (376 MHz, $CDCl_3$) (δ, ppm) -113.90; HRMS (TOF MS ESI$^+$) calculated for $C_{34}H_{24}FNO_4Na[M+Na]^+$: 552.1582, found: 552.1580.

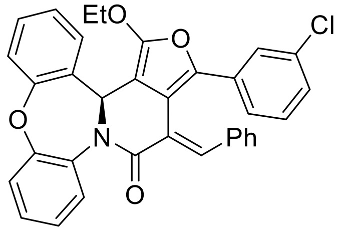

(*S*,*E*)-15-Benzylidene-1-(3-chlorophenyl)-3-ethoxy-3b*H*-dibenzo[*b*,*f*]furo[3',4':3,4]pyrido[1,2-*d*][1,4]oxazepin-14(15*H*)-one (4e). Condition B: 36.5 mg, 67% yield; 90% *ee*, $[\alpha]_D^{20}$ = 33.00 (c = 0.033, CH_2Cl_2); Yellow oil; ^{1}H NMR (400 MHz, $CDCl_3$) (δ, ppm) 7.50 - 7.49 (m, 1H), 7.39 - 7.37 (m, 1H), 7.20 - 7.09 (comp, 7H), 7.02 - 7.00 (comp, 4H), 6.88 - 6.84 (comp, 4H), 6.82 - 6.78 (m, 1H), 6.21 - 6.20 (m, 1H), 4.34 - 4.23 (m, 2H), 1.34 - 1.30 (m, 3H); ^{13}C NMR (101 MHz, $CDCl_3$) (δ, ppm) 167.5, 154.6, 153.5, 149.9, 137.4, 136.5, 135.5, 133.6, 132.3, 131.2, 130.8, 129.8, 129.2, 128.9, 128.71, 128.65, 128.0, 127.9, 126.8, 125.9, 124.9, 123.5, 122.9, 122.8, 122.6, 121.3, 121.0, 116.4, 94.6, 68.7, 54.6, 15.3; HRMS (TOF MS ESI$^+$) calculated for $C_{34}H_{24}ClNO_4Na[M+Na]^+$: 568.1286, found: 568.1285; HPLC conditions for determination of enantiomeric excess: Chiralpak IC, λ = 254 nm, hexane : 2-propanol = 90:10, flow rate = 1.0 mL/min, t_{minor} = 11.9 min, t_{major} = 22.1min.

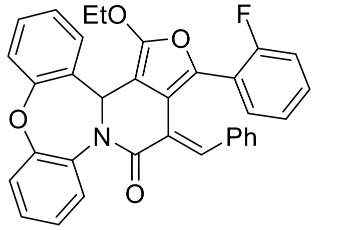

(*E*)-15-Benzylidene-3-ethoxy-1-(2-fluorophenyl)-3b*H*-dibenzo[*b*,*f*]furo[3',4':3,4]pyrido[1,2-*d*][1,4]oxazepin-14(15*H*)-one (4f). Condition A: 45.5 mg, 86% yield;

Yellow solid, m.p. = 230.1 - 232.7 °C; ^{1}H NMR (400 MHz, $CDCl_3$) (δ, ppm) 7.62 (s, 1H), 7.49 (d, *J* = 7.9 Hz, 1H), 7.29 - 7.22 (comp, 4H), 7.19 - 7.18 (comp, 3H), 7.14 (d, *J* = 7.6 Hz, 1H), 7.11 - 7.07 (m, 1H), 6.99 (t, *J* = 6.9 Hz, 2H), 6.90 - 6.88 (comp, 3H), 6.80 (d, *J* = 7.6 Hz, 1H), 6.62 (t, *J* = 9.4 Hz, 1H), 6.32 (s, 1H), 4.43 - 4.28 (m, 2H), 1.40 (t, *J* = 7.0 Hz, 3H); ^{13}C NMR (101 MHz, $CDCl_3$) (δ, ppm) 167.2, 158.2 (d, *J* = 252.4 Hz), 154.7, 153.9, 150.0, 137.2 (d, *J* = 1.7 Hz), 135.5, 134.6, 131.7, 131.1, 129.8, 129.4, 129.3, 128.7, 128.6 (d, *J* = 1.6 Hz), 128.3, 127.8, 127.6, 126.1, 123.6, 123.3, 122.6 (d, *J* = 4.1 Hz), 121.3, 121.0, 120.0 (d, *J* = 13.7 Hz), 117.9, 115.6 (d, *J* = 20.1 Hz), 94.1, 68.6, 54.6, 15.4; ^{19}F NMR (376 MHz, $CDCl_3$) (δ, ppm) -109.36; HRMS (TOF MS ESI^+) calculated for $C_{34}H_{24}FNO_4Na[M+Na]^+$: 552.1582, found: 552.1586.

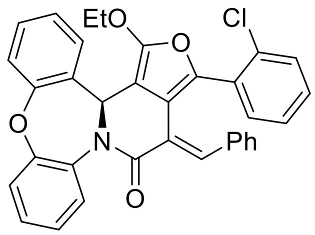

(*S*,*E*)-15-Benzylidene-1-(2-chlorophenyl)-3-ethoxy-3b*H*-dibenzo[*b*,*f*]furo[3',4':3,4]pyrido[1,2-*d*][1,4]oxazepin-14(15*H*)-one (4g). Condition B: 35.4 mg, 65% yield; 90% *ee*, $[\alpha]_D^{20}$ = 33.00 (c = 0.033, CH_2Cl_2); Yellow oil; ^{1}H NMR (500 MHz, $CDCl_3$) (δ, ppm) 7.50 (s, 1H), 7.42 (d, *J* = 8.0 Hz, 1H), 7.22 - 7.15 (comp, 4H), 7.13 - 7.11 (m, 2H), 7.09 - 7.07 (m, 2H), 7.04 - 7.01 (m, 1H), 6.98 - 6.94 (m, 2H), 6.89 - 6.80 (comp, 5H), 6.25 (s, 1H), 4.35 - 4.20 (m, 2H), 1.34 - 1.31 (m, 3H); ^{13}C NMR (126 MHz, $CDCl_3$) (δ, ppm) 167.0, 154.7, 153.7, 149.9, 137.1, 136.9, 135.4, 132.5, 131.7, 131.0, 130.7, 130.5, 129.9, 129.8, 129.3, 129.1, 128.6, 128.2, 127.8, 127.7, 126.08, 126.05, 123.7, 122.6, 121.3, 121.0, 117.9, 93.6, 68.6, 54.7, 15.3; HRMS (TOF MS ESI^+) calculated for $C_{34}H_{24}ClNO_4Na[M+Na]^+$: 568.1286, found: 568.1283; HPLC conditions for determination of enantiomeric excess: Chiralpak IC, λ = 254 nm, hexane : 2-propanol = 90:10, flow rate = 1.0 mL/min, t_{minor} = 12.1 min, t_{major} = 25.4min.

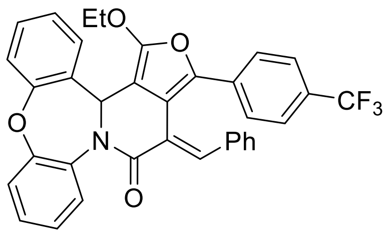

(*E*)-15-Benzylidene-3-ethoxy-1-(4-(trifluoromethyl)phenyl)-3b*H*-dibenzo[*b,f*]furo [3',4':3,4]pyrido[1,2-*d*][1,4]oxazepin-14(15*H*)-one (4h). Condition A: 46.9 mg, 81% yield; Yellow solid, m.p. = 228.2 - 229.7 °C; ^{1}H NMR (400 MHz, $CDCl_3$) (δ, ppm) 7.59 (s, 1H), 7.47 (d, *J* = 8.0 Hz, 1H), 7.30 - 7.21 (comp, 7H), 7.19 - 7.08 (comp, 5H), 6.98 - 6.93 (comp, 4H), 6.31 (s, 1H), 4.48 - 4.33 (m, 2H), 1.43 (t, *J* = 7.0 Hz, 3H); ^{13}C NMR (101 MHz, $CDCl_3$) (δ, ppm) 167.4, 154.7, 153.9, 150.0, 137.5, 136.7, 135.5, 133.9, 131.2, 130.9, 129.9, 129.2, 129.1, 128.8, 128.4 (q, *J* = 32.6 Hz), 128.1, 128.0, 125.9, 124.8, 124.4 (q, *J* = 3.4 Hz), 124.2 (q, *J* = 272.1 Hz), 123.6, 123.0, 122.7, 121.4, 121.1, 117.5, 94.9, 68.7, 54.7, 15.4; ^{19}F NMR (376 MHz, $CDCl_3$) (δ, ppm) -62.55; HRMS (TOF MS ESI$^+$) calculated for $C_{35}H_{24}F_3NO_4Na[M+Na]^+$: 602.1550, found: 602.1545.

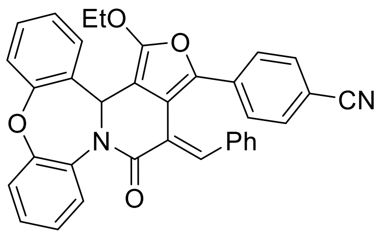

(*S,E*)-4-(15-Benzylidene-3-ethoxy-14-oxo-14,15-dihydro-3b*H*-dibenzo[*b,f*]furo[3', 4':3,4]pyrido[1,2-*d*][1,4]oxazepin-1-yl)benzonitrile (4i). Condition A: 20.9 mg, 39% yield; Yellow oil; ^{1}H NMR (400 MHz, $CDCl_3$) (δ, ppm) 7.59 (s, 1H), 7.45 (d, *J* = 8.0 Hz, 1H), 7.30 - 7.29 (comp, 3H), 7.26 - 7.23 (comp, 5H), 7.21 - 7.19 (comp, 3H), 7.11 - 7.10 (m, 2H), 7.04 - 7.02 (m, 1H), 7.00 - 6.97 (m, 2H), 6.23 (s, 1H), 4.49 - 4.35 (m, 2H), 1.44 (t, *J* = 7.0 Hz, 3H); ^{13}C NMR (101 MHz, $CDCl_3$) (δ, ppm) 167.2, 154.7, 154.3, 149.9, 137.1, 136.7, 135.3, 134.4, 131.3, 131.0, 130.7, 130.0, 129.4, 129.1, 128.8, 128.3, 128.0, 125.8, 124.7, 123.6, 123.0, 122.7, 121.4, 121.1, 119.1, 118.9, 109.3, 95.1, 68.7, 54.6, 15.3; HRMS (TOF MS ESI$^+$) calculated for $C_{35}H_{24}N_2O_4Na[M+Na]^+$: 559.1628, found: 559.1619.

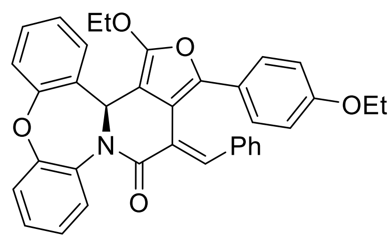

(*S*,*E*)-15-Benzylidene-3-ethoxy-1-(4-ethoxyphenyl)-3b*H*-dibenzo[*b*,*f*]furo[3',4':3,4]pyrido[1,2-*d*][1,4]oxazepin-14(15*H*)-one (4j). Condition A: 37.8 mg, 68% yield, **Condition B**: 40.6 mg, 73% yield; 95% *ee*, $[\alpha]_D^{20}$ = 579.1 (c = 0.033, CH_2Cl_2); Yellow oil; ^1H NMR (400 MHz, $CDCl_3$) (δ, ppm) 7.51 (s, 1H), 7.48 (d, *J* = 7.9 Hz, 1H), 7.31 - 7.27 (comp, 3H), 7.23 - 7.16 (comp, 3H), 7.13 - 7.07 (comp, 4H), 7.00 - 6.94 (comp, 4H), 6.47 (d, *J* = 8.6 Hz, 2H), 6.29 (s, 1H), 4.42 - 4.27 (m, 2H), 3.95 - 3.84 (m, 2H), 1.40 (t, *J* = 7.1 Hz, 3H), 1.35 (t, *J* = 6.9 Hz, 3H); ^{13}C NMR (101 MHz, $CDCl_3$) (δ, ppm) 168.0, 158.2, 154.7, 152.7, 149.9, 139.6, 135.6, 135.1, 131.6, 131.0, 129.7, 129.3, 129.1, 128.8, 127.8, 126.5, 126.0, 123.7, 123.6, 123.3, 122.5, 121.3, 120.9, 113.7, 94.5, 68.8, 63.6, 54.7, 15.4, 14.8; HRMS (TOF MS ESI^+) calculated for $C_{36}H_{29}NO_5Na[M+Na]^+$: 578.1938, found: 578.1945; HPLC conditions for determination of enantiomeric excess: Chiralpak IC, λ = 254 nm, hexane : 2-propanol = 90:10, flow rate = 1.0 mL/min, t_{minor} = 19.9 min, t_{major} = 33.2 min.

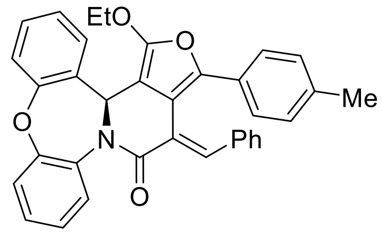

(*S*,*E*)-15-Benzylidene-3-ethoxy-1-(*p*-tolyl)-3b*H*-dibenzo[*b*,*f*]furo[3',4':3,4]pyrido[1,2-*d*][1,4]oxazepin-14(15*H*)-one (4k). Condition A: 45.2 mg, 86% yield, **Condition B**: 40.6 mg, 71% yield; 92% *ee*, $[\alpha]_D^{20}$ = 405.0 (c = 0.033, CH_2Cl_2); Yellow oil; ^1H NMR (400 MHz, $CDCl_3$) (δ, ppm) 7.51 (s, 1H), 7.48 (d, *J* = 8.0 Hz, 1H), 7.31 - 7.27 (comp, 3H), 7.24 - 7.16 (comp, 3H), 7.13 - 7.06 (comp, 4H), 6.98 - 6.91 (comp, 4H), 6.76 - 6.74 (m, 2H), 6.29 (s, 1H), 4.41 - 4.29 (m, 2H), 2.18 (s, 3H), 1.40 (t, *J* = 7.0 Hz, 3H); ^{13}C NMR (101 MHz, $CDCl_3$) (δ, ppm) 168.0, 154.6, 152.9, 149.9, 139.6, 137.0, 135.6, 135.5, 131.5, 130.9, 129.7, 129.3, 129.0, 128.7, 128.2,

128.0, 127.82, 127.76, 126.0, 124.9, 123.6, 123.2, 122.5, 121.3, 120.9, 114.6, 94.5, 68.7, 54.6, 21.3, 15.4; HRMS (TOF MS ESI$^+$) calculated for $C_{35}H_{27}NO_4Na[M+Na]^+$: 548.1832, found: 548.1837; HPLC conditions for determination of enantiomeric excess: Chiralpak IC, λ = 254 nm, hexane : 2-propanol = 90:10, flow rate = 1.0 mL/min, t_{minor} = 12.7 min, t_{major} = 21.3min.

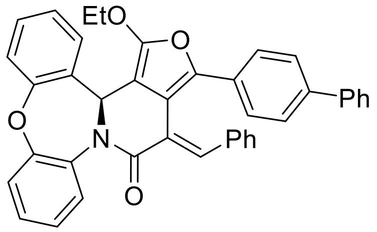

(*S*,*E*)-1-([1,1'-Biphenyl]-4-yl)-15-benzylidene-3-ethoxy-3b*H*-dibenzo[*b*,*f*]furo[3',4':3,4]pyrido[1,2-*d*][1,4]oxazepin-14(15*H*)-one (4l). Condition A: 48.1 mg, 82% yield, **Condition B**: 40.9 mg, 77% yield; 90% *ee*, $[\alpha]_D^{20}$ = 33.0 (c = 0.033, CH_2Cl_2); Yellow oil; ^{1}H NMR (400 MHz, $CDCl_3$) (δ, ppm) 7.59 (s, 1H), 7.51 - 7.49 (m, 1H), 7.46 - 7.44 (m, 2H), 7.40 - 7.37 (m, 2H), 7.33 - 7.30 (comp, 3H), 7.27 - 7.22 (comp, 4H), 7.21 - 7.20 (m, 1H), 7.18 - 7.14 (comp, 4H), 7.10 - 7.07 (m, 1H), 6.96 - 6.92 (comp, 4H), 6.32 (s, 1H), 4.43 - 4.32 (m, 2H), 1.42 - 1.38 (m, 3H); ^{13}C NMR (101 MHz, $CDCl_3$) (δ, ppm) 167.7, 154.6, 153.2, 149.8, 140.7, 139.6, 139.0, 135.8, 135.5, 131.4, 130.9, 129.72, 129.65, 129.2, 128.9, 128.8, 127.82, 127.76, 127.3, 126.9, 126.1, 126.0, 125.3, 123.5, 123.1, 122.5, 121.3, 120.9, 115.5, 94.6, 68.7, 54.6, 15.3; HRMS (TOF MS ESI$^+$) calculated for $C_{40}H_{29}NO_4Na[M+Na]^+$: 610.1989, found 610.1983; HPLC conditions for determination of enantiomeric excess: Chiralpak IC, λ = 254 nm, hexane : 2-propanol = 90:10, flow rate = 1.0 Ml/min, t_{minor} = 20.8 min, t_{major} = 44.3min.

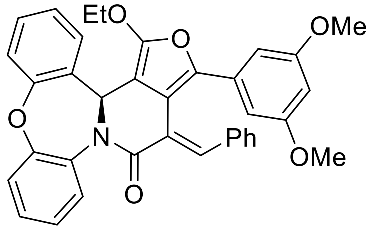

(*S*,*E*)-15-Benzylidene-1-(3,5-dimethoxyphenyl)-3-ethoxy-3b*H*-dibenzo[*b*,*f*]furo[3',

4':3,4]pyrido[1,2-*d*][1,4]oxazepin-14(15*H*)-one (4m). Condition A: 45.2 mg, 79% yield, **Condition B**: 42.3 mg, 74% yield; 91% *ee*, $[\alpha]_D^{20}$ = 414.0 (c = 0.033, CH_2Cl_2); Yellow solid, m.p. = 109.1 - 111.0 °C; ^1H NMR (400 MHz, $CDCl_3$) (δ, ppm) 7.46 (s, 1H), 7.40 - 7.38 (m, 1H), 7.24 (s, 1H), 7.21 - 7.19 (m, 1H), 7.16 - 7.08 (comp, 4H), 7.03 - 7.00 (m, 2H), 6.98 - 6.95 (m, 1H), 6.93 - 6.89 (m, 2H), 6.86 - 6.84 (m, 1H), 6.33 (d, J = 2.2 Hz, 2H), 6.21 (s, 1H), 6.04 (t, J = 2.1 Hz, 1H), 4.34 - 4.22 (m, 2H), 3.51 (s, 6H), 1.33 (t, J = 7.9 Hz, 3H); ^{13}C NMR (101 MHz, $CDCl_3$) (δ, ppm) 167.8, 160.0, 154.6, 153.0, 149.8, 139.1, 135.8, 135.4, 132.5, 131.3, 130.8, 129.7, 129.2, 129.0, 128.9, 127.8, 127.7, 125.9, 123.5, 123.0, 122.4, 121.2, 120.9, 115.8, 103.3, 100.1, 94.5, 68.6, 55.2, 54.5, 15.2. HRMS (TOF MS ESI$^+$) calculated for $C_{36}H_{29}NO_6Na[M+Na]^+$: 594.1887, found: 594.1880; HPLC conditions for determination of enantiomeric excess: Chiralpak IC, λ = 254 nm, hexane : 2-propanol = 85:15, flow rate = 1.0 mL/min, t_{minor} = 14.0 min, t_{major} = 24.8min.

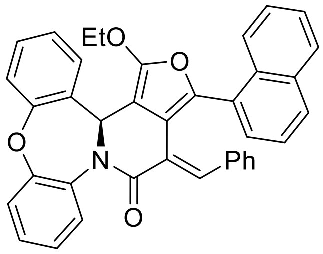

(*S*,*E*)-15-Benzylidene-3-ethoxy-1-(naphthalen-1-yl)-3b*H*-dibenzo[*b*,*f*]furo[3',4':3,4]pyrido[1,2-*d*][1,4]oxazepin-14(15*H*)-one (4n). Condition A: 44.9 mg, 80% yield, **Condition B**: 44.9 mg, 80% yield; 95% *ee*, $[\alpha]_D^{20}$ = 363.0 (c = 0.033, CH_2Cl_2); Yellow solid, m.p. = 135.8 - 137.2 °C; ^1H NMR (400 MHz, $CDCl_3$) (δ, ppm) 7.85 (d, J = 8.3 Hz, 1H), 7.63 (d, J = 7.9 Hz, 1H), 7.57 (d, J = 7.9 Hz, 1H), 7.52 - 7.47 (comp, 3H), 7.43 - 7.41 (m, 2H), 7.32 - 7.23 (comp, 3H), 7.21 - 7.19 (m, 2H), 7.15 - 7.11 (m, 2H), 7.02 (t, J = 6.8 Hz, 1H), 6.72 - 6.71 (m, 2H), 6.54 - 6.51 (m, 1H), 6.41 - 6.39 (comp, 3H), 4.46 - 4.29 (m, 2H), 1.43 - 1.40 (m, 3H); ^{13}C NMR (101 MHz, $CDCl_3$) (δ, ppm) 167.2, 154.7, 153.7, 150.0, 139.0, 137.0, 134.9, 133.4, 131.8, 131.1, 129.8, 129.7, 129.4, 129.3, 128.7, 128.5, 127.83, 127.80, 127.4, 127.2, 126.8, 126.3, 126.1, 125.9, 125.7, 124.5, 123.7, 122.6, 122.5, 121.4, 121.0, 117.4, 94.1, 68.7, 54.7, 15.4;

HRMS (TOF MS ESI+) calculated for $C_{38}H_{27}NO_4Na[M+Na]^+$: 584.1832, found: 584.1833; HPLC conditions for determination of enantiomeric excess: Chiralpak IC, λ = 254 nm, hexane : 2-propanol = 90:10, flow rate = 1.0 mL/min, t_{minor} = 11.3 min, t_{major} = 21.5min.

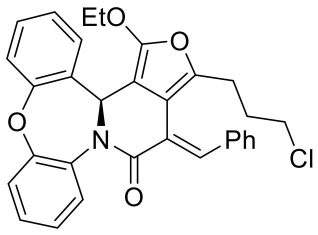

(*S*,*E*)-15-Benzylidene-1-(3-chloropropyl)-3-ethoxy-3b*H*-dibenzo[*b*,*f*]furo[3',4':3,4]pyrido[1,2-*d*][1,4]oxazepin-14(15*H*)-one (4o). Condition A: 42.5 mg, 83% yield, **Condition B**: 40.9 mg, 80% yield; 93% *ee*, $[\alpha]_D^{20}$ = -72.0 (c = 0.033, CH_2Cl_2); Yellow solid, m.p. = 172.4 - 174.2 °C; ^{1}H NMR (400 MHz, $CDCl_3$) (δ, ppm) 7.53 (s, 1H), 7.46 - 7.41 (comp, 3H), 7.36 (t, *J* = 7.5 Hz, 2H), 7.30 - 7.28 (m, 1H), 7.26 - 7.22 (comp, 3H), 7.20 - 7.16 (m, 1H), 7.13 - 7.12 (m, 1H), 7.09 - 7.04 (m, 1H), 7.01 (t, *J* = 7.3 Hz, 1H), 6.20 (s, 1H), 4.28 - 4.17 (m, 2H), 3.29 - 3.19 (m, 2H), 2.16 - 2.08 (m, 1H), 1.98 - 1.90 (m, 1H), 1.88 - 1.71 (m, 2H), 1.34 (t, *J* = 7.1 Hz, 3H); ^{13}C NMR (101 MHz, $CDCl_3$) (δ, ppm) 166.8, 154.5, 152.6, 149.9, 140.6, 136.5, 135.1, 131.4, 131.0, 129.8, 129.2, 128.9, 128.8, 128.5, 127.8, 126.1, 123.7, 123.4, 122.5, 121.3, 120.9, 115.2, 92.9, 68.8, 55.1, 44.2, 29.8, 26.1, 15.3; HRMS (TOF MS ESI+) calculated for $C_{31}H_{26}ClNO_4Na$ $[M+Na]^+$: 534.1443, found: 534.1447; HPLC conditions for determination of enantiomeric excess: Chiralpak IC, λ = 254 nm, hexane : 2-propanol = 90:10, flow rate = 1.0 mL/min, t_{minor} = 15.0 min, t_{major} = 21.6min.

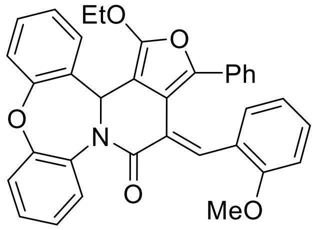

(*E*)-3-Ethoxy-15-(2-methoxybenzylidene)-1-phenyl-3b*H*-dibenzo[*b*,*f*]furo[3',4':3,4]pyrido[1,2-*d*][1,4]oxazepin-14(15*H*)-one (4p). Condition A: 42.2 mg, 78% yield;

Yellow solid, m.p. = 259.6 - 260.8 °C; ^{1}H NMR (500 MHz, $CDCl_3$) (δ, ppm) 7.81 (s, 1H), 7.49 (d, *J* = 8.0 Hz, 1H), 7.28 - 7.15 (comp, 8H), 7.07 (t, *J* = 7.5 Hz, 1H), 6.99 - 6.95 (comp, 5H), 6.57 (d, *J* = 8.2 Hz, 1H), 6.40 (t, *J* = 7.3 Hz, 1H), 6.31 (s, 1H), 4.41 - 4.27 (m, 2H), 3.78 (s, 3H), 1.39 (t, *J* = 7.0 Hz, 3H); ^{13}C NMR (126 MHz, $CDCl_3$) (δ, ppm) 167.6, 157.4, 154.7, 153.0, 149.8, 139.2, 132.3, 131.7, 130.9, 130.8, 130.3, 129.7, 129.4, 128.2, 127.6, 127.3, 126.7, 126.0, 125.1, 125.0, 123.5, 122.9, 122.4, 121.2, 120.9, 119.7, 115.6, 110.2, 94.5, 68.7, 55.3, 54.6, 15.3; HRMS (TOF MS ESI$^+$) calculated for $C_{35}H_{27}NO_5Na$ [M+Na]$^+$: 564.1781, found: 564.1778.

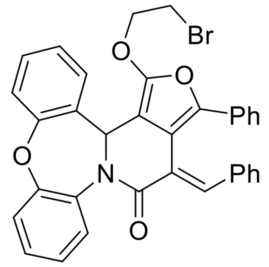

(*E*)-15-Benzylidene-3-(2-bromoethoxy)-1-phenyl-3b*H*-dibenzo[*b*,*f*]furo[3',4':3,4]pyrido[1,2-*d*][1,4]oxazepin-14(15*H*)-one (4q). Condition A: 41.3 mg, 70% yield; Yellow solid, m.p. = 172.4 - 174.2 °C; ^{1}H NMR (500 MHz, $CDCl_3$) (δ, ppm) 7.56 (s, 1H), 7.47 (d, *J* = 7.9 Hz, 1H), 7.29 - 7.28 (comp, 3H), 7.26 - 7.24 (m, 1H), 7.22 - 7.19 (comp, 4H), 7.12 - 7.09 (m, 2H), 7.00 - 6.91 (comp, 7H), 6.33 (s, 1H), 4.61 - 4.55 (m, 2H), 3.61 (t, *J* = 6.2 Hz, 2H); ^{13}C NMR (126 MHz, $CDCl_3$) (δ, ppm) 167.7, 154.7, 152.0, 150.0, 139.8, 136.1, 135.5, 131.1, 131.0, 130.5, 129.8, 129.2, 129.0, 128.9, 127.91, 127.85, 127.6, 127.3, 126.0, 125.1, 123.6, 122.8, 122.7, 121.3, 121.0, 115.3, 96.0, 71.9, 54.5, 28.7; HRMS (TOF MS ESI$^+$) calculated for $C_{34}H_{24}BrNO_4Na$ [M+Na]$^+$: 612.0781, found: 612.0787.

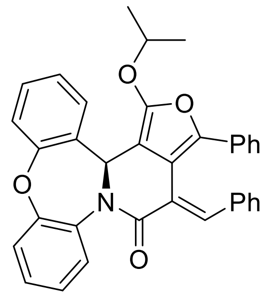

(*S*,*E*)-15-Benzylidene-3-isopropoxy-1-phenyl-3b*H*-dibenzo[*b*,*f*]furo[3',4':3,4]pyrido[1,2-*d*][1,4]oxazepin-14(15*H*)-one (4r). Condition B: 32.6 mg, 62% yield; 91% *ee*, $[\alpha]_D^{20}$ = 210.0 (c = 0.033, CH_2Cl_2); Yellow oil; ^1H NMR (500 MHz, $CDCl_3$) (δ, ppm) 7.46 - 7.45 (m, 1H), 7.41 - 7.39 (m, 1H), 7.23 - 7.20 (comp, 3H), 7.19 - 7.09 (comp, 5H), 7.08 - 7.00 (m, 2H), 6.88 - 6.86 (comp, 7H), 6.18 - 6.17 (m, 1H), 4.72 - 4.69 (m, 1H), 1.35 - 1.33 (m, 3H), 1.30 - 1.29 (m, 3H); ^{13}C NMR (101 MHz, $CDCl_3$) (δ, ppm) 167.9, 154.7, 152.6, 149.9, 139.5, 135.7, 135.6, 131.5, 130.93, 130.85, 129.7, 129.2, 129.0, 128.8, 127.83, 127.77, 127.5, 127.0, 126.0, 125.0, 123.6, 123.2, 122.5, 121.3, 121.0, 115.2, 96.3, 54.7, 22.8, 22.6; HRMS (TOF MS ESI+) calculated for $C_{35}H_{27}NO_4Na$ $[M+Na]^+$: 548.1832, found 548.1824; HPLC conditions for determination of enantiomeric excess: Chiralpak IC, λ = 254 nm, hexane : 2-propanol = 90:10, flow rate = 1.0 mL/min, tminor = 10.2 min, tmajor = 16.2min.

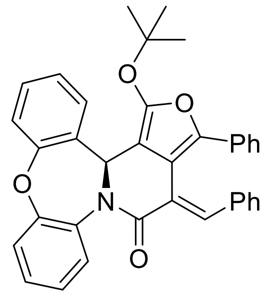

(*S*,*E*)-15-Benzylidene-3-(*tert*-butoxy)-1-phenyl-3b*H*-dibenzo[*b*,*f*]furo[3',4':3,4]pyrido[1,2-*d*][1,4]oxazepin-14(15*H*)-one (4s). Condition B: 32.6 mg, 58% yield; 90% *ee*, $[\alpha]_D^{20}$ = -6.0 (c = 0.033, CH_2Cl_2); Yellow oil; ^1H NMR (400 MHz, $CDCl_3$) (δ, ppm) 7.51 (s, 1H), 7.48 - 7.45 (m , 1H), 7.31 - 7.27 (comp, 3H), 7.23 - 7.18 (comp, 4H), 7.11- 7.06 (m, 2H), 7.00 - 6.90 (comp, 8H), 6.22 (s, 1H), 1.46 (s, 9H); ^{13}C NMR (101 MHz, $CDCl_3$) (δ, ppm) 168.1, 154.7, 151.6, 149.9, 140.4, 135.7, 135.5, 131.7, 131.00,

130.96, 129.7, 129.3, 129.0, 128.8, 127.9, 127.8, 127.6, 127.1, 126.1, 125.2, 123.6, 123.4, 122.6, 121.3, 121.0, 114.8, 100.7, 84.9, 54.8, 29.1. HRMS (TOF MS ESI+) calculated for $C_{36}H_{29}NO_4Na$ [M+Na]$^+$: 562.1989, found: 562.1990; HPLC conditions for determination of enantiomeric excess: Chiralpak IC, λ = 254 nm, hexane : 2-propanol = 90:10, flow rate = 1.0 mL/min, tminor = 10.2 min, tmajor = 16.2min.

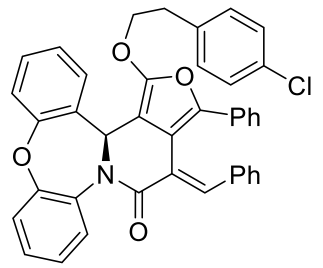

(*S*,*E*)-15-Benzylidene-3-(4-chlorophenethoxy)-1-phenyl-3b*H*-dibenzo[*b,f*]furo[3', 4':3,4]pyrido[1,2-*d*][1,4]oxazepin-14(15*H*)-one (4t). Condition B: 43.5 mg, 70% yield; 90% *ee*, $[\alpha]_D^{20}$ = 237.0 (c = 0.033, CH_2Cl_2); Yellow solid, m.p. = 136.7 - 138.2 °C; ^{1}H NMR (500 MHz, $CDCl_3$) (δ, ppm) 7.48 (s, 1H), 7.39 (d, *J* = 7.9 Hz, 1H), 7.22 - 7.20 (comp, 4H), 7.18 - 7.12 (comp, 7H), 7.04 - 7.02 (m, 2H), 6.92 - 6.83 (comp, 8H), 6.25 (s, 1H), 4.53 - 4.47 (m, 2H), 3.53 (t, *J* = 6.2 Hz, 3H); ^{13}C NMR (126 MHz, $CDCl_3$) (δ, ppm) 167.7, 154.7, 152.0, 150.0, 139.8, 136.1, 135.5, 131.1, 131.0, 130.5, 129.8, 129.2, 129.0, 128.9, 127.91, 127.85, 127.6, 127.3, 126.0, 125.1, 123.6, 122.8, 122.7, 121.3, 121.0, 115.3, 96.0, 71.9, 54.5, 28.7; HRMS (TOF MS ESI+) calculated for $C_{40}H_{28}ClNO_4Na$ [M+Na]+: 644.1599, found: 644.1597; HPLC conditions for determination of enantiomeric excess: Chiralpak IC, λ = 254 nm, hexane : 2-propanol = 90:10, flow rate = 1.0 mL/min, tminor = 15.4 min, tmajor = 24.6min.

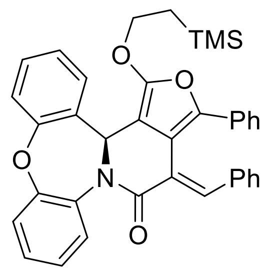

(*S*,*E*)-15-Benzylidene-1-phenyl-3-(2-(trimethylsilyl)ethoxy)-3b*H*-dibenzo[*b*,*f*]furo [3',4':3,4]pyrido[1,2-*d*][1,4]oxazepin-14(15*H*) (4u). Condition B: 39.5 mg, 68% yield; 90% *ee*, $[\alpha]_D^{20}$ = -33.0 (c = 0.033, CH_2Cl_2); Yellow oil; ^{1}H NMR (400 MHz, $CDCl_3$) (δ, ppm) 7.52 (s, 1H), 7.47 - 7.45 (m, 1H), 7.29 - 7.25 (comp, 3H), 7.22 - 7.14 (comp, 5H), 7.11 - 7.04 (m, 2H), 6.96 - 6.88 (comp, 7H), 6.26 (s, 1H), 4.40 - 4.33 (m, 2H), 1.16 - 1.12 (t, *J* = 8.0 Hz, 3H), 0.00 (s, 9H); ^{13}C NMR (101 MHz, $CDCl_3$) (δ, ppm) 167.9, 154.7, 153.3, 150.0, 139.3, 135.8, 135.6, 131.4, 131.1, 130.9, 129.7, 129.3, 129.0, 128.8, 127.8, 127.6, 126.9, 126.1, 125.0, 123.5, 123.2, 122.6, 121.3, 120.9, 115.4, 95.0, 71.4, 71.3, 54.8, 54.7, 18.6, -1.35, -1.37; HRMS (TOF MS ESI+) calculated for $C_{37}H_{33}NO_4SiNa$[M+Na]+: 606.2071, found: 606.2069; HPLC conditions for determination of enantiomeric excess: Chiralpak IC, λ = 254 nm, hexane : 2-propanol = 90:10, flow rate = 1.0 mL/min, tminor = 8.9 min, tmajor = 13.2min.

General Procedure for the [4+4] Cycloaddition Reaction

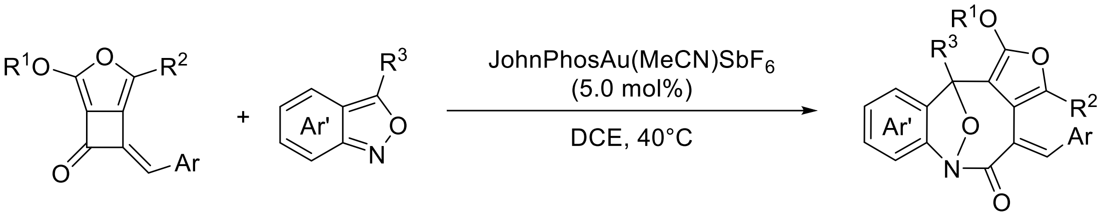

To a 10-mL oven-dried vial containing a magnetic stirring bar, cyclobutanone **1** (0.1 mmol, 1.0 equiv.), and anthranil **5** (0.11 mmol, 1.1 equiv.) in DCE (1.0 mL), was added a solution of JohnPhosAu(MeCN)SbF_6 (3.9 mg, 5.0 mol%) in DCE (1.0 mL) *via* syringe under argon atmosphere at 40 °C. After addition, the reaction mixture was stirred 12 h under these conditions until consumption of the material (monitored by TLC). The solvent was evaporated in vacuo. Then, the residues was purified by column chromatography on silica gel without any additional treatment (Hexanes : EtOAc = 30:1 to 10:1) to give the pure products **6** or **7** in good to high yields.

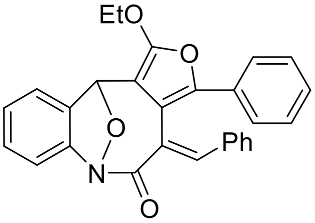

(*E*)-4-Benzylidene-1-ethoxy-3-phenyl-11*H*-6,11-epoxybenzo[*b*]furo[3,4-*e*]azocin-5(4*H*)-one (6a). Yellow solid, m.p. = 185.6 - 186.6 °C; 40.5 mg, 93% yield; ^{1}H NMR (400 MHz, $CDCl_3$) (δ, ppm) 7.52 (d, *J* = 7.8 Hz, 1H), 7.38 (d, *J* = 7.3 Hz, 1H), 7.34 - 7.30 (m, 1H), 7.24 - 7.17 (comp, 5H), 6.95 - 6.93 (comp, 6H), 6.91 (s, 1H), 6.27 (s, 1H), 4.39 - 4.23 (m, 2H), 1.45 (t, *J* = 7.1 Hz, 3H); ^{13}C NMR (101 MHz, $CDCl_3$) (δ, ppm) 179.8, 152.6, 142.4, 141.1, 136.4, 135.2, 133.6, 130.4, 130.0, 128.80, 128.77, 128.2, 127.9, 127.3, 126.7, 125.7, 120.7, 115.8, 110.7, 99.7, 77.4, 68.2, 15.4; HRMS (TOF MS ESI$^+$) calculated for $C_{28}H_{22}NO_4$ [M+H]$^+$: 436.1543, found 436.1546.

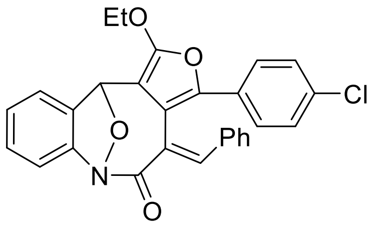

(*E*)-4-Benzylidene-3-(4-chlorophenyl)-1-ethoxy-11*H*-6,11-epoxybenzo[*b*]furo[3,4-*e*]azocin-5(4*H*)-one (6b). Yellow solid, m.p. = 159.7 - 160.7 °C; 41.4 mg, 88% yield; ^{1}H NMR (400 MHz, $CDCl_3$) (δ, ppm) 7.51 (d, *J* = 7.7 Hz, 1H), 7.38 (d, *J* = 7.3 Hz, 1H), 7.33 (t, *J* = 7.6 Hz, 1H), 7.22 (d, *J* = 7.5 Hz, 1H), 7.18 - 7.17 (m, 2H), 7.13 (d, *J* = 8.5 Hz, 2H), 7.00 - 6.99 (comp, 3H), 6.94 - 6.91 (comp, 3H), 6.26 (s, 1H), 4.39 - 4.24 (m, 2H), 1.46 (t, *J* = 7.0 Hz, 3H); ^{13}C NMR (126 MHz, $CDCl_3$) (δ, ppm) 179.6, 152.8, 142.4, 139.8, 136.3, 135.1, 133.9, 132.9, 130.0, 128.9, 128.84, 128.77, 128.6, 128.11, 128.08, 127.0, 126.8, 120.7, 115.8, 111.4, 99.8, 77.3, 68.3, 15.4; HRMS (TOF MS ESI$^+$) calculated for $C_{28}H_{21}ClNO_4$ $[M+H]^+$: 470.1154, found 470.1156.

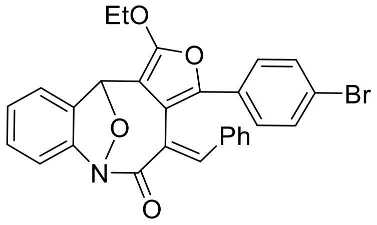

(*E*)-4-Benzylidene-3-(4-bromophenyl)-1-ethoxy-11*H*-6,11-epoxybenzo[*b*]furo[3,4-*e*]azocin-5(4*H*)-one (6c). Yellow solid, m.p. = 179.1 - 180.5 °C; 46.3 mg, 90% yield; ^{1}H NMR (400 MHz, $CDCl_3$) (δ, ppm) 7.51 (d, *J* = 7.7 Hz, 1H), 7.38 (d, *J* = 7.3 Hz, 1H), 7.33 (t, *J* = 7.6 Hz, 1H), 7.22 (d, *J* = 7.4 Hz, 1H), 7.19 - 7.16 (m, 2H), 7.09 - 7.05 (comp, 4H), 7.00 - 6.99 (comp, 3H), 6.93 (s, 1H), 6.26 (s, 1H), 4.39 - 4.23 (m, 2H), 1.46 (t, *J* = 7.1 Hz, 3H); ^{13}C NMR (126 MHz, $CDCl_3$) (δ, ppm) 179.5, 152.8, 142.4, 139.8, 136.3, 135.1, 133.9, 131.0, 130.0, 129.3, 128.9, 128.8, 128.6, 128.1, 127.2, 126.8, 121.1, 120.7, 115.8, 111.5, 99.8, 77.3, 68.2, 15.4; HRMS (TOF MS ESI$^+$) calculated for $C_{28}H_{20}BrNO_4Na$ $[M+Na]^+$: 536.0468, found 536.0464.

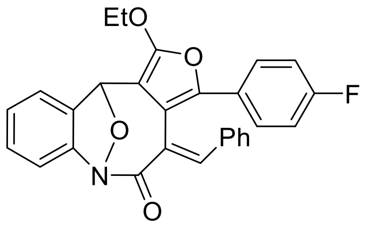

(*E*)-4-Benzylidene-1-ethoxy-3-(4-fluorophenyl)-11*H*-6,11-epoxybenzo[*b*]furo[3,4-*e*]azocin-5(4*H*)-one (6d). Yellow solid, m.p. = 171.7 - 172.9 °C; 38.5 mg, 85% yield; ^{1}H NMR (400 MHz, $CDCl_3$) (δ, ppm) 7.52 (d, *J* = 7.7 Hz, 1H), 7.39 (d, *J* = 7.4 Hz, 1H), 7.33 (t, *J* = 7.6 Hz, 1H), 7.22 (t, *J* = 7.5 Hz, 1H), 7.18 - 7.15 (comp, 4H), 6.99 - 6.98 (comp, 3H), 6.91 (s, 1H), 6.66 (t, *J* = 8.7 Hz, 2H), 6.27 (s, 1H), 4.39 - 4.23 (m, 2H), 1.46 (t, *J* = 7.1 Hz, 3H); ^{13}C NMR (126 MHz, $CDCl_3$) (δ, ppm) 179.7, 162.0 (d, *J* = 247.6 Hz), 152.6, 142.4, 140.2, 136.4, 135.2, 133.7, 130.1, 128.9, 128.8, 128.4, 128.0, 127.8 (d, *J* = 8.2 Hz), 126.8, 126.7 (d, *J* = 3.3 Hz), 120.7, 115.8, 115.0 (d, *J* = 21.9 Hz), 110.7, 99.8, 77.3, 68.3, 15.4; ^{19}F NMR (376 MHz, $CDCl_3$) (δ, ppm) -113.85; HRMS (TOF MS ESI$^+$) calculated for $C_{28}H_{20}FNO_4Na$ [M+Na]$^+$: 476.1269, found 476.1272.

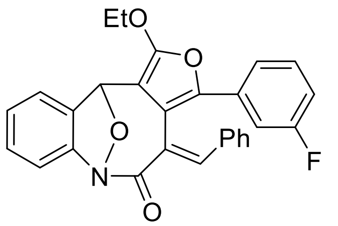

(*E*)-4-Benzylidene-1-ethoxy-3-(3-fluorophenyl)-11*H*-6,11-epoxybenzo[*b*]furo[3,4-*e*]azocin-5(4*H*)-one (6e). Yellow solid, m.p. = 174.5 - 175.6 °C; 41.3 mg, 91% yield; ^{1}H NMR (500 MHz, $CDCl_3$) (δ, ppm) 7.52 (d, *J* = 7.8 Hz, 1H), 7.39 (d, *J* = 7.4 Hz, 1H), 7.33 (t, *J* = 7.6 Hz, 1H), 7.23 - 7.19 (comp, 3H), 7.02 - 6.98 (comp, 4H), 6.96 (s, 1H), 6.93 - 6.86 (m, 2H), 6.64 - 6.60 (m, 1H), 6.27 (s, 1H), 4.40 - 4.26 (m, 2H), 1.46 (t, *J* = 7.1 Hz, 3H); ^{13}C NMR (126 MHz, $CDCl_3$) (δ, ppm) 179.5, 162.3 (d, *J* = 244.5 Hz), 152.8, 142.4, 139.4 (d, *J* = 2.9 Hz), 136.3, 135.2, 134.2, 132.3 (d, *J* = 8.7 Hz), 129.9, 129.4 (d, *J* = 8.5 Hz), 128.9, 128.7, 128.5, 128.0, 126.8, 121.3 (d, *J* = 2.8 Hz), 120.7, 115.8, 113.9 (d, *J* = 21.3 Hz), 112.3 (d, *J* = 23.6 Hz), 111.9, 99.8, 77.3, 68.2, 15.4; ^{19}F NMR (470 MHz, $CDCl_3$) (δ, ppm) -113.65; HRMS (TOF MS ESI$^+$)

calculated for $C_{28}H_{20}FNO_4Na$ $[M+Na]^+$: 476.1269, found 476.1271.

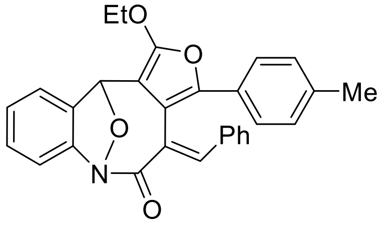

(*E*)-4-Benzylidene-1-ethoxy-3-(*p*-tolyl)-11*H*-6,11-epoxybenzo[*b*]furo[3,4-*e*]azocin-5(4*H*)-one (6f). Yellow solid, m.p. = 188.1 - 182.3 °C; 41.4 mg, 92% yield; ^1H NMR (400 MHz, $CDCl_3$) (δ, ppm) 7.51 (d, *J* = 7.8 Hz, 1H), 7.38 (d, *J* = 7.4 Hz, 1H), 7.34 - 7.30 (m, 1H), 7.23 - 7.17 (comp, 3H), 7.10 (d, *J* = 8.2 Hz, 2H), 6.96 - 6.95 (comp, 3H), 6.89 (s, 1H), 6.76 (d, *J* = 8.0 Hz, 2H), 6.26 (s, 1H), 4.38 - 4.22 (m, 2H), 2.14 (s, 3H), 1.45 (t, *J* = 7.1 Hz, 3H); ^{13}C NMR (101 MHz, $CDCl_3$) (δ, ppm) 179.9, 152.4, 142.5, 141.5, 137.2, 136.5, 135.3, 133.4, 130.2, 128.9, 128.8, 128.6, 128.2, 127.9, 127.6, 126.7, 125.7, 120.7, 115.8, 110.1, 99.7, 77.4, 68.2, 21.3, 15.5; HRMS (TOF MS ESI^+) calculated for $C_{29}H_{23}NO_4Na$ $[M+Na]^+$: 472.1519, found 472.1521.

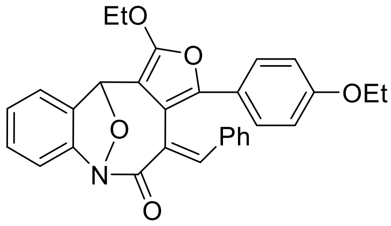

(*E*)-4-Benzylidene-1-ethoxy-3-(4-ethoxyphenyl)-11*H*-6,11-epoxybenzo[*b*]furo[3,4-*e*]azocin-5(4*H*)-one (6g). Yellow solid, m.p. = 196.8 - 197.9 °C; 43.6 mg, 91% yield; ^1H NMR (400 MHz, $CDCl_3$) (δ, ppm) 7.52 (d, *J* = 7.8 Hz, 1H), 7.38 (d, *J* = 7.3 Hz, 1H), 7.34 (d, *J* = 7.6 Hz, 1H), 7.23 - 7.18 (comp, 3H), 7.13 (d, *J* = 8.4 Hz, 2H), 6.98 - 6.97 (comp, 3H), 6.89 (s, 1H), 6.49 (d, *J* = 8.5 Hz, 2H), 6.26 (s, 1H), 4.37 - 4.21 (m, 2H), 3.91 - 3.85 (m, 2H), 1.46 (d, *J* = 7.1 Hz, 3H), 1.32 (t, *J* = 6.9 Hz, 3H); ^{13}C NMR (101 MHz, $CDCl_3$) (δ, ppm) 180.0, 158.3, 152.2, 142.5, 141.4, 136.5, 135.3, 133.2, 130.2, 128.9, 128.8, 128.3, 127.9, 127.3, 126.7, 123.2, 120.7, 115.8, 114.1, 109.3, 99.7, 77.4, 68.3, 63.5, 15.5, 14.8; HRMS (TOF MS ESI^+) calculated for $C_{30}H_{25}NO_5Na$ $[M+Na]^+$: 502.1625, found 502.1629.

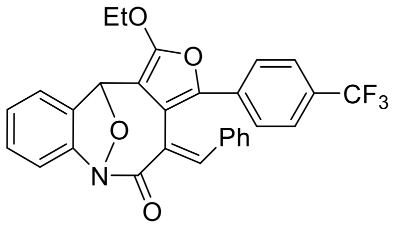

(*E*)-4-Benzylidene-1-ethoxy-3-(4-(trifluoromethyl)phenyl)-11*H*-6,11-epoxybenzo[*b*]furo[3,4-*e*]azocin-5(4*H*)-one (6h). Yellow solid, m.p. = 168.5 - 169.9 °C; 40.8 mg, 81% yield; ^{1}H NMR (500 MHz, $CDCl_3$) (δ, ppm) 7.53 (d, *J* = 7.7 Hz, 1H), 7.40 (d, *J* = 7.4 Hz, 1H), 7.34 (t, *J* = 7.5 Hz, 1H), 7.28 (d, *J* = 8.2 Hz, 2H), 7.23 (t, *J* = 7.5 Hz, 1H), 7.19 (d, *J* = 8.3 Hz, 2H), 7.15 - 7.13 (m, 2H), 6.97 - 6.94 (comp, 4H), 6.28 (s, 1H), 4.42 - 4.28 (m, 2H), 1.47 (t, *J* = 7.1 Hz, 3H); ^{13}C NMR (126 MHz, $CDCl_3$) (δ, ppm) 179.4, 153.3, 142.4, 139.2, 136.2, 135.1, 134.4, 133.7, 129.9, 129.0, 128.70 (q, *J* = 32.4 Hz), 128.68, 128.6, 128.1, 126.8, 125.8, 124.8 (q, *J* = 3.8 Hz), 124.1 (q, *J* = 271.9 Hz), 120.7, 115.9, 112.8, 99.9, 77.3, 68.2, 15.4; ^{19}F NMR (470 MHz, $CDCl_3$) (δ, ppm) -62.78; HRMS (TOF MS ESI$^+$) calculated for $C_{29}H_{20}F_3NO_4Na$ $[M+Na]^+$: 526.1237, found 526.1233.

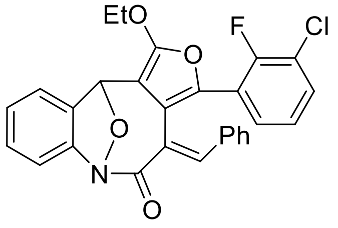

(*E*)-4-Benzylidene-3-(3-chloro-2-fluorophenyl)-1-ethoxy-11*H*-6,11-epoxybenzo[*b*]furo[3,4-*e*]azocin-5(4*H*)-one (6i). Yellow solid, m.p. = 166.8 - 167.9 °C; 38.5 mg, 79% yield; ^{1}H NMR (500 MHz, $CDCl_3$) (δ, ppm) 7.52 (d, *J* = 7.7 Hz, 1H), 7.40 (d, *J* = 7.4 Hz, 1H), 7.34 (t, *J* = 7.7 Hz, 1H), 7.23 (t, *J* = 7.5 Hz, 1H), 7.06 - 6.96 (comp, 7H), 6.95 (s, 1H) 6.71 (t, *J* = 7.9 Hz, 1H), 6.28 (s, 1H), 4.38 - 4.24 (m, 2H), 1.45 (t, *J* = 7.0 Hz, 3H); ^{13}C NMR (126 MHz, $CDCl_3$) (δ, ppm) 179.3, 154.5 (d, *J* = 254.6 Hz), 153.4, 142.4, 136.2, 135.1, 135.0, 134.8, 130.0, 129.9, 128.9, 128.6, 128.5, 128.2, 128.00, 127.97, 126.7, 124.0 (d, *J* = 4.4 Hz), 121.4 (d, *J* = 17.7 Hz), 120.8, 120.3 (d, *J* = 13.8 Hz), 115.8, 114.3, 99.3, 77.3, 68.1, 15.4; ^{19}F NMR (376 MHz, $CDCl_3$) (δ, ppm) -113.36; HRMS (TOF MS ESI$^+$) calculated for $C_{28}H_{19}ClFNO_4Na$ $[M+Na]^+$: 510.0879,

found 510.0882.

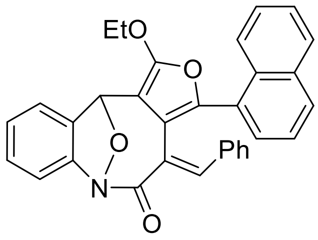

(*E*)-4-Benzylidene-1-ethoxy-3-(naphthalen-1-yl)-11*H*-6,11-epoxybenzo[*b*]furo[3,4-*e*]azocin-5(4*H*)-one (6j). Yellow solid, m.p. = 169.7 - 170.6 °C; 43.2 mg, 89% yield; ^{1}H NMR (400 MHz, $CDCl_3$) (δ, ppm) 7.62 - 7.56 (comp, 3H), 7.51 (d, *J* = 7.9 Hz, 1H), 7.44 (d, *J* = 7.3 Hz, 1H), 7.39 - 7.34 (comp, 3H), 7.27 - 7.23 (m, 1H), 7.19 - 7.17 (m, 1H), 7.14 -7.11 (m, 1H), 6.79 (s, 1H), 6.70 (d, *J* = 7.3 Hz, 2H), 6.53 (t, *J* = 7.5 Hz, 2H), 6.46 (t, *J* = 7.3 Hz, 1H), 6.33 (s, 1H), 4.38 - 4.21 (m, 2H), 1.45 (t, *J* = 7.1 Hz, 3H); ^{13}C NMR (126 MHz, $CDCl_3$) (δ, ppm) 179.7, 153.1, 142.6, 141.0, 136.4, 134.8, 134.5, 133.6, 130.2, 130.1, 128.9, 128.8, 128.3, 128.2, 127.74, 127.65, 127.4, 126.8, 126.7, 126.2, 125.8, 125.6, 124.9, 120.8, 115.9, 113.3, 99.5, 77.5, 68.2, 15.5; HRMS (TOF MS ESI$^+$) calculated for $C_{32}H_{23}NO_4Na$ [M+Na]$^+$: 508.1519, found 508.1520.

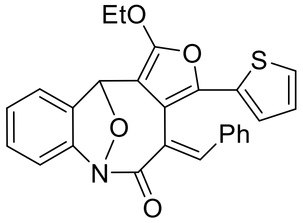

(*E*)-4-Benzylidene-1-ethoxy-3-(thiophen-2-yl)-11*H*-6,11-epoxybenzo[*b*]furo[3,4-*e*] azocin-5(4*H*)-one (6k). Yellow oil; 26.9 mg, 61% yield; ^{1}H NMR (500 MHz, $CDCl_3$) (δ, ppm) 7.50 (d, *J* = 7.7 Hz, 1H), 7.39 (d, *J* = 7.3 Hz, 1H), 7.34 - 7.31 (comp, 3H), 7.21 (t, *J* = 7.4 Hz, 1H), 7.09 - 7.08 (comp, 3H), 6.97 (s, 1H), 6.94 (d, *J* = 5.0 Hz, 1H), 6.74 (d, *J* = 3.0 Hz, 1H), 6.61 - 6.59 (m, 1H), 6.26 (s, 1H), 4.39 - 4.25 (m, 2H), 1.46 (t, *J* = 7.0 Hz, 3H); ^{13}C NMR (126 MHz, $CDCl_3$) (δ, ppm) 179.5, 152.2, 142.4, 136.5, 136.3, 135.4, 133.9, 131.4, 129.5, 128.88, 128.85, 128.7, 128.2, 127.0, 126.7, 125.0, 124.7, 120.6, 115.8, 110.8, 99.7, 77.3, 68.4, 15.4; HRMS (TOF MS ESI$^+$) calculated for $C_{29}H_{19}NO_4SNa$ [M+Na]$^+$: 464.0927, found 464.0931.

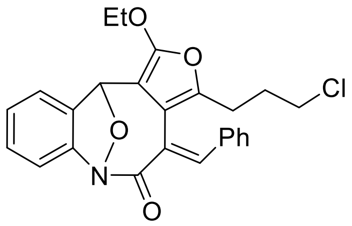

(*E*)-4-Benzylidene-3-(3-chloropropyl)-1-ethoxy-11*H*-6,11-epoxybenzo[*b*]furo[3,4-*e*]azocin-5(4*H*)-one (6l). Yellow solid, m.p. = 132.6 - 133.6 °C; 26.2 mg, 60% yield; ^{1}H NMR (500 MHz, $CDCl_3$) (δ, ppm) 7.46 (d, *J* = 7.7 Hz, 1H), 7.43 (d, *J* = 7.5 Hz, 2H), 7.36 (d, *J* = 7.4 Hz, 1H), 7.33 - 7.29 (comp, 3H), 7.27 - 7.24 (m, 1H), 7.20 (t, *J* = 7.4 Hz, 1H), 7.02 (s, 1H), 6.19 (s, 1H), 4.26 - 4.13 (m, 2H), 3.15 (t, *J* = 6.7 Hz, 2H), 2.04 - 1.98 (m, 1H), 1.94 - 1.88 (m, 1H), 1.67 - 1.61 (m, 1H), 1.47 - 1.43 (m, 1H), 1.41 (t, *J* = 7.1 Hz, 3H); ^{13}C NMR (126 MHz, $CDCl_3$) (δ, ppm) 179.4, 151.9, 142.4, 141.8, 136.4, 135.8, 132.4, 130.9, 129.0, 128.83, 128.75, 128.5, 126.6, 120.6, 115.8, 111.2, 98.6, 77.4, 68.3, 44.3, 29.4, 25.0, 15.4; HRMS (TOF MS ESI$^+$) calculated for $C_{25}H_{22}ClNO_4Na$ $[M+Na]^+$: 458.1130, found 458.1134.

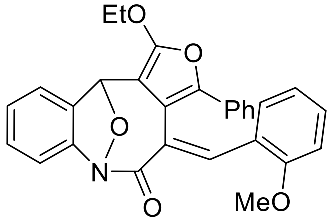

(*E*)-1-Ethoxy-4-(2-methoxybenzylidene)-3-phenyl-11*H*-6,11-epoxybenzo[*b*]furo[3,4-*e*]azocin-5(4*H*)-one (6m). Yellow solid, m.p. = 206.9 - 207.9 °C; 39.6 mg, 85% yield; ^{1}H NMR (400 MHz, $CDCl_3$) (δ, ppm) 7.52 (d, *J* = 7.7 Hz, 1H), 7.37 (d, *J* = 7.4 Hz, 1H), 7.34 - 7.30 (m, 1H), 7.26 - 7.25 (m, 1H), 7.22 - 7.16 (comp, 4H), 6.98 - 6.90 (comp, 4H), 6.52 (t, *J* = 7.5 Hz, 1H), 6.44 (d, *J* = 8.3 Hz, 1H), 6.27 (s, 1H), 4.37 - 4.21 (m, 2H), 3.67 (s, 3H), 1.44 (t, *J* = 7.1 Hz, 3H); ^{13}C NMR (101 MHz, $CDCl_3$) (δ, ppm) 179.8, 157.3, 152.5, 142.6, 141.0, 136.5, 130.6, 130.4, 129.9, 129.7, 128.9, 128.8, 127.7, 126.9, 126.6, 125.8, 124.2, 120.7, 119.7, 115.8, 111.1, 110.0, 100.0, 77.4, 68.2, 54.9, 15.4; HRMS (TOF MS ESI$^+$) calculated for $C_{29}H_{23}NO_5Na$ $[M+Na]^+$: 488.1468, found 488.1469.

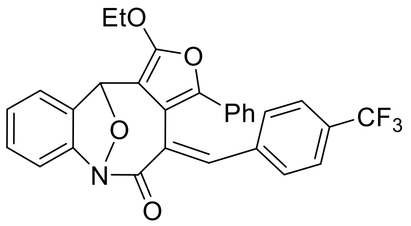

(*E*)-1-Ethoxy-3-phenyl-4-(4-(trifluoromethyl)benzylidene)-11*H*-6,11-epoxybenzo[*b*]furo[3,4-*e*]azocin-5(4*H*)-one (6n). Yellow solid, m.p. = 171.8 - 172.6 °C; 37.3 mg, 74% yield; ^{1}H NMR (400 MHz, $CDCl_3$) (δ, ppm) 7.53 (d, *J* = 7.7 Hz, 1H), 7.40 (d, *J* = 7.4 Hz, 1H), 7.37 - 7.33 (m, 1H), 7.23 (d, *J* = 7.4 Hz, 1H), 7.20 - 7.15 (comp, 4H), 7.09 - 7.07 (m, 2H), 6.98 - 6.95 (comp, 3H), 6.90 (s, 1H), 6.29 (s, 1H), 4.39 - 4.23 (m, 2H), 1.45 (t, *J* = 7.1 Hz, 3H); ^{13}C NMR (101 MHz, $CDCl_3$) (δ, ppm) 179.2, 152.8, 142.3, 141.7, 139.1, 136.2, 132.6, 131.8, 130.2, 129.5 (q, *J* = 32.7 Hz), 129.0, 128.9, 128.2, 127.7, 126.9, 126.4, 124.8 (q, *J* = 3.5 Hz), 121.3 (q, *J* = 274.0 Hz), 120.8, 115.8, 110.3, 99.6, 77.4, 68.3, 15.4; ^{19}F NMR (376 MHz, $CDCl_3$) (δ, ppm) -62.99; HRMS (TOF MS ESI$^+$) calculated for $C_{29}H_{20}F_3NO_4Na$ [M+Na]$^+$: 526.1237, found 526.1240.

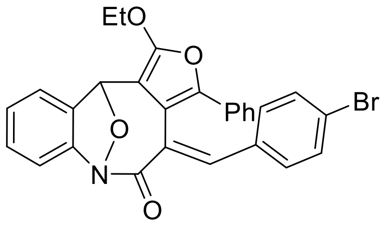

(*E*)-4-(4-Bromobenzylidene)-1-ethoxy-3-phenyl-11*H*-6,11-epoxybenzo[*b*]furo[3,4-*e*]azocin-5(4*H*)-one (6o). Yellow solid, m.p. = 191.6 - 192.8 °C; 45.8 mg, 89% yield; ^{1}H NMR (500 MHz, $CDCl_3$) (δ, ppm) 7.52 (d, *J* = 7.8 Hz, 1H), 7.39 (d, *J* = 7.4 Hz, 1H), 7.35 - 7.32 (m, 1H), 7.24 - 7.21 (m, 1H), 7.18 - 7.16 (m, 2H), 7.07 - 7.05 (m, 2H), 7.03 - 6.99 (comp, 5H), 6.82 (s, 1H), 6.26 (s, 1H), 4.38 - 4.24 (m, 2H), 1.46 (t, *J* = 7.1 Hz, 3H); ^{13}C NMR (126 MHz, $CDCl_3$) (δ, ppm) 179.5, 152.8, 142.4, 141.3, 136.3, 134.2, 132.3, 131.1, 131.0, 130.3, 128.9, 128.1, 127.7, 126.8, 126.0, 122.2, 120.7, 115.8, 110.5, 99.6, 77.4, 68.2, 15.5; HRMS (TOF MS ESI$^+$) calculated for $C_{28}H_{20}BrNO_4Na$ [M+Na]$^+$: 536.0468, found 536.0461.

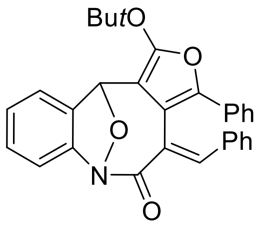

(*E*)-4-Benzylidene-1-(*tert*-butoxy)-3-phenyl-11*H*-6,11-epoxybenzo[*b*]furo[3,4-*e*]azocin-5(4*H*)-one (6p). Yellow solid, m.p. = 162.4 - 163.6 °C; 43.1 mg, 93% yield; ^{1}H NMR (400 MHz, $CDCl_3$) (δ, ppm) 7.52 (d, *J* = 7.7 Hz, 1H), 7.40 (d, *J* = 7.3 Hz, 1H), 7.32 (t, *J* = 7.6 Hz, 1H), 7.22 - 7.15 (comp, 5H), 6.95 - 6.91 (comp, 7H), 6.23 (s, 1H), 1.53 (s, 9H); ^{13}C NMR (101 MHz, $CDCl_3$) (δ, ppm) 179.9, 151.2, 142.4, 142.0, 136.2, 135.3, 133.4, 130.5, 130.2, 128.9, 128.8, 128.2, 127.9, 127.3, 126.6, 125.8, 120.9, 115.8, 110.0, 104.6, 84.5, 77.7, 29.2; HRMS (TOF MS ESI$^+$) calculated for $C_{30}H_{25}NO_4Na$ [M+Na]$^+$: 486.1676, found 486.1678.

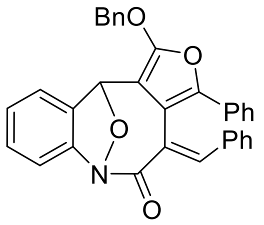

(*E*)-4-Benzylidene-1-(benzyloxy)-3-phenyl-11*H*-6,11-epoxybenzo[*b*]furo[3,4-*e*]azocin-5(4*H*)-one (6q). Yellow oil; 42.3 mg, 85% yield; ^{1}H NMR (500 MHz, $CDCl_3$) (δ, ppm) 7.49 (d, *J* = 7.8 Hz, 1H), 7.46 - 7.43 (comp, 5H), 7.30 (t, *J* = 7.7 Hz, 1H), 7.22 - 7.19 (comp, 3H), 7.15 - 7.12 (m, 3H), 6.99 - 6.93 (comp, 6H), 6.90 (s, 1H), 6.10 (s, 1H), 5.29 (d, *J* = 11.5 Hz, 1H), 5.23 (d, *J* = 11.5 Hz, 1H); ^{13}C NMR (126 MHz, $CDCl_3$) (δ, ppm) 179.8, 152.0, 142.3, 141.6, 136.2, 135.5, 135.2, 133.8, 130.3, 130.0, 129.2, 129.0, 128.8, 128.7, 128.2, 128.0, 127.9, 127.4, 126.7, 125.9, 120.8, 115.7, 110.7, 101.1, 77.3, 74.3; HRMS (TOF MS ESI$^+$) calculated for $C_{33}H_{23}NO_4Na$ [M+Na]$^+$: 520.1519, found 520.1523.

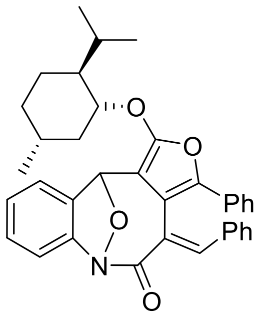

4-((*E*)-Benzylidene)-1-(((*1R,2S,5R*)-2-isopropyl-5-methylcyclohexyl)oxy)-3-phenyl-11*H*-6,11-epoxybenzo[*b*]furo[3,4-*e*]azocin-5(4*H*)-one (6r). Yellow solid, m.p. = 142.4 - 144.1 °C; 47.5 mg, 87% yield, 1:1 *dr*; ^{1}H NMR (400 MHz, $CDCl_3$) (δ, ppm) 7.63 - 7.61 (m, 2H), 7.56 - 7.50 (comp, 4H), 7.43 - 7.37 (m, 2H), 7.35 - 7.28 (comp, 4H), 7.22 - 7.20 (comp, 8H), 6.98 - 6.95 (comp, 8H), 6.92 - 6.91 (m, 2H), 6.26 (d, *J* = 2.7 Hz, 1H), 6.24 (d, *J* = 2.7 Hz, 1H), 4.38 - 4.33 (m, 2H), 2.42 - 2.35 (m, 1H), 2.27 - 2.24 (m, 1H), 2.21 - 2.17 (m, 1H), 1.95 - 1.92 (m, 1H), 1.78 - 1.67 (comp, 5H), 1.60 - 1.55 (m, 3H), 1.38 - 1.34 (m, 2H), 1.29 - 1.27 (m, 2H), 1.14 - 1.09 (m, 2H), 1.06 - 1.01 (comp, 6H), 0.97 (dd, *J* = 6.9, 2.8 Hz, 6H), 0.88 (dd, *J* = 6.3, 2.8 Hz, 3H), 0.81 (dd, *J* = 6.8, 2.9 Hz, 3H); ^{13}C NMR (126 MHz, $CDCl_3$) (δ, ppm) 179.9, 179.8, 156.1, 154.5, 152.3, 152.1, 142.4, 142.3, 140.9, 140.8, 136.2, 135.2, 135.2, 133.4, 130.9, 130.5, 130.4, 130.1, 130.0, 128.8, 128.73, 128.71, 128.7, 128.13, 128.07, 127.83, 127.79, 127.2, 127.1, 126.5, 126.3, 125.8, 125.5, 124.4, 121.1, 120.9, 119.7, 118.2, 115.7, 115.6, 115.1, 110.7, 110.6, 100.4, 100.0, 82.8, 82.7, 77.5, 77.3, 48.07, 48.05, 41.9, 41.6, 34.21, 34.17, 31.7, 31.5, 26.1, 23.5, 23.2, 22.3, 22.0, 21.0, 20.8, 16.3, 16.2; HRMS (TOF MS ESI$^+$) calculated for $C_{36}H_{35}NO_4Na$ [M+Na]$^+$: 568.2458, found 568.2460.

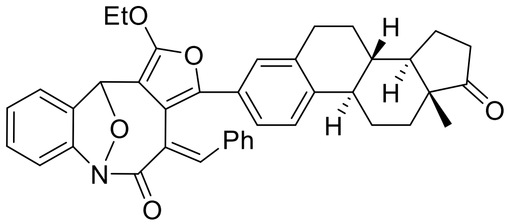

4-((*E*)-Benzylidene)-1-ethoxy-3-((*8S,9R,13R,14R*)-13-methyl-17-*oxo*-7,8,9,11,12,13,14,15,16,17-decahydro-6*H*-cyclopenta[*a*]phenanthren-3-yl)-11*H*-6,11-epoxybenz

o[*b*]furo[3,4-*e*]azocin-5(4*H*)-one (6s). Yellow solid, m.p. = 158.9 - 160.1 °C; 53.8 mg, 88% yield, 1:1 *dr*; ^{1}H NMR (500 MHz, $CDCl_3$) (δ, ppm) 7.52 (d, *J* = 7.7 Hz, 1H), 7.39 (d, *J* = 7.3 Hz, 1H), 7.35 - 7.32 (m, 1H), 7.23 - 7.20 (m, 1H), 7.15 - 7.13 (m, 2H), 6.98 - 6.92 (comp, 4H), 6.89 - 6.88 (d, *J* = 5.9 Hz, 2H), 6.77 (d, *J* = 9.6 Hz, 1H), 6.26 (s, 1H), 4.37 - 4.23 (m, 2H), 2.62 - 2.56 (m, 2H), 2.49 (dd, *J* = 19.1, 8.8 Hz, 1H), 2.28 - 2.25 (m, 1H), 2.16 - 2.09 (m, 2H), 2.05 - 2.00 (m, 1H), 1.93 - 1.88 (m, 2H), 1.64 - 1.56 (m, 2H), 1.48 - 1.39 (comp, 7H), 0.89 (s, 3H); ^{13}C NMR (126 MHz, $CDCl_3$) (δ, ppm) 180.01, 179.97, 152.41, 152.40, 142.5, 141.4, 141.2, 139.1, 138.9, 136.4, 135.7, 135.7, 135.54, 135.49, 133.39, 133.35, 130.2, 130.1, 128.8, 128.7, 128.6, 128.04, 127.99, 127.9, 127.8, 127.69, 127.65, 126.7, 126.6, 126.5, 124.9, 123.7, 123.6, 120.7, 115.8, 110.3, 110.2, 99.68, 99.65, 77.41, 77.36, 68.2, 50.53, 50.49, 48.0, 44.4, 38.1, 36.0, 31.6, 29.4, 26.5, 26.4, 25.8, 25.7, 21.69, 21.67, 15.4, 13.92, 13.90; HRMS (TOF MS ESI^{+}) calculated for $C_{40}H_{37}NO_5Na$ $[M+Na]^{+}$: 634.2564, found 634.2567.

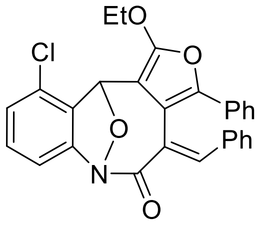

(*E*)-4-Benzylidene-10-chloro-1-ethoxy-3-phenyl-11*H*-6,11-epoxybenzo[*b*]furo[3,4-*e*]azocin-5(4*H*)-one (7a). Yellow solid, m.p. = 209.9 - 211.1 °C; 35.7 mg, 76% yield; ^{1}H NMR (500 MHz, $CDCl_3$) (δ, ppm) 7.42 (d, *J* = 7.7 Hz, 1H), 7.29 - 7.25 (m, 1H), 7.21 - 7.17 (comp, 3H), 7.13 - 7.12 (m, 2H), 6.99 - 6.94 (comp, 6H), 6.90 (s, 1H), 6.41 (s, 1H), 4.43 - 4.36 (m, 1H), 4.28 - 4.22 (m, 1H), 1.45 (t, *J* = 7.1 Hz, 3H); ^{13}C NMR (126 MHz, $CDCl_3$) (δ, ppm) 179.5, 153.3, 144.0, 141.5, 135.2, 134.6, 134.3, 130.31, 130.25, 129.6, 128.7, 128.2, 128.0, 127.9, 127.44, 127.37, 127.2, 126.1, 114.2, 110.7, 97.7, 77.3, 68.3, 15.2; HRMS (TOF MS ESI^{+}) calculated for $C_{28}H_{20}ClNO_4Na$ $[M+Na]^{+}$: 492.0973, found 492.0973.

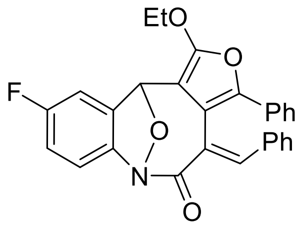

(*E*)-4-Benzylidene-1-ethoxy-9-fluoro-3-phenyl-11*H*-6,11-epoxybenzo[*b*]furo[3,4-*e*] azocin-5(4*H*)-one (7b). Yellow solid, m.p. = 172.6 - 174.1 °C; 37.2 mg, 82% yield; ^{1}H NMR (400 MHz, $CDCl_3$) (δ, ppm) 7.46 (dd, *J* = 8.5, 4.2 Hz, 1H), 7.22 - 7.17 (comp, 4H), 7.09 (dd, *J* = 7.4, 2.4 Hz, 1H), 7.02 (dd, *J* = 8.8, 2.5 Hz, 1H), 6.99 - 6.94 (comp, 6H), 6.91 (s, 1H), 6.24 (s, 1H), 4.42 - 4.27 (m, 2H), 1.47 (t, *J* = 7.1 Hz, 3H); ^{13}C NMR (101 MHz, $CDCl_3$) (δ, ppm) 179.8, 161.4 (d, *J* = 245.3 Hz), 152.8, 141.2, 138.50, 138.45 (d, *J* = 6.2 Hz), 135.1, 133.8, 130.3, 129.7, 128.8, 128.4, 128.0, 127.4, 125.8, 116.9 (d, *J* = 8.9 Hz), 115.3 (d, *J* = 24.2 Hz), 110.6, 108.8, 108.5, 98.6, 77.4, 68.1, 15.5; ^{19}F NMR (376 MHz, $CDCl_3$) (δ, ppm) -115.52; HRMS (TOF MS ESI$^+$) calculated for $C_{28}H_{20}FNO_4Na$ $[M+Na]^+$: 476.1269, found 476.1266.

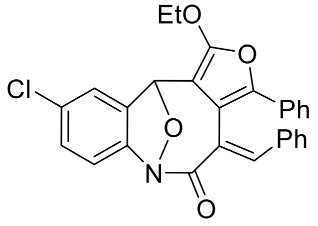

(*E*)-4-Benzylidene-9-chloro-1-ethoxy-3-phenyl-11*H*-6,11-epoxybenzo[*b*]furo[3,4-*e*]azocin-5(4*H*)-one (7c). Yellow solid, m.p. = 175.6 - 176.9 °C; 39.5 mg, 84% yield; ^{1}H NMR (400 MHz, $CDCl_3$) (δ, ppm) 7.52 (d, *J* = 1.5 Hz, 1H), 7.30 (d, *J* = 7.9 Hz, 1H), 7.21 - 7.16 (comp, 5H), 6.98 - 6.94 (comp, 6H), 6.92 (s, 1H), 6.25 (s, 1H), 4.40 - 4.25 (m, 2H), 1.45 (t, *J* = 7.1 Hz, 3H); ^{13}C NMR (101 MHz, $CDCl_3$) (δ, ppm) 179.3, 152.6, 143.6, 141.3, 135.11, 135.09, 134.6, 134.1, 130.3, 129.6, 128.8, 128.4, 128.0, 127.4, 126.8, 125.8, 121.4, 116.5, 110.5, 99.1, 77.3, 68.1, 15.5; HRMS (TOF MS ESI$^+$) calculated for $C_{28}H_{20}ClNO_4Na$ $[M+Na]^+$: 492.0973, found 492.0977.

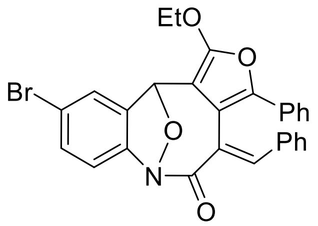

(*E*)-4-Benzylidene-9-bromo-1-ethoxy-3-phenyl-11*H*-6,11-epoxybenzo[*b*]furo[3,4-*e*]azocin-5(4*H*)-one (7d). Yellow oil; 33.9 mg, 66% yield; ^{1}H NMR (400 MHz, $CDCl_3$) (δ, ppm) 7.50 (d, *J* = 1.3 Hz, 1H), 7.46 - 7.44 (m, 1H), 7.40 - 7.38 (m, 1H), 7.21 - 7.16 (comp, 4H), 6.98 - 6.95 (comp, 6H), 6.91 (s, 1H), 6.24 (s, 1H), 4.42 - 4.28 (m, 2H), 1.47 (t, *J* = 7.1 Hz, 3H); ^{13}C NMR (101 MHz, $CDCl_3$) (δ, ppm) 179.4, 152.7, 141.6, 141.2, 138.7, 135.1, 134.0, 131.7, 130.3, 129.5, 128.8, 128.4, 127.9, 127.4, 125.8, 124.1, 119.6, 117.2, 110.5, 98.7, 77.2, 68.1, 15.4; HRMS (TOF MS ESI$^+$) calculated for $C_{28}H_{20}BrNO_4Na$ $[M+Na]^+$: 536.0468, found 536.0465.

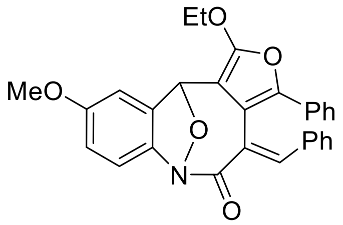

(*E*)-4-Benzylidene-1-ethoxy-9-methoxy-3-phenyl-11*H*-6,11-epoxybenzo[*b*]furo[3,4-*e*]azocin-5(4*H*)-one (7e). Yellow solid, m.p. = 142.1 - 143.6 °C; 36.8 mg, 79% yield; ^{1}H NMR (400 MHz, $CDCl_3$) (δ, ppm) 7.42 (d, *J* = 8.5 Hz, 1H), 7.23 - 7.17 (comp, 4H), 6.98 - 6.93 (comp, 7H), 6.89 (s, 1H), 6.81 (dd, *J* = 8.5, 2.5 Hz, 1H), 6.22 (s, 1H), 4.40 - 4.25 (m, 2H), 3.80 (s, 3H), 1.46 (t, *J* = 7.1 Hz, 3H); ^{13}C NMR (101 MHz, $CDCl_3$) (δ, ppm) 180.3, 158.8, 152.7, 141.0, 138.1, 135.9, 135.3, 133.4, 130.4, 130.1, 128.8, 128.2, 127.9, 127.3, 125.8, 116.4, 112.7, 110.8, 107.7, 99.4, 77.5, 68.1, 56.0, 15.5; HRMS (TOF MS ESI$^+$) calculated for $C_{29}H_{23}NO_5Na$ $[M+Na]^+$: 488.1468, found 488.1470.

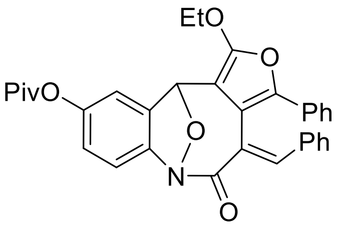

(*E*)-4-Benzylidene-1-ethoxy-5-*oxo*-3-phenyl-4,5-dihydro-11*H*-6,11-epoxybenzo[*b*]furo[3,4-*e*]azocin-9-yl pivalate (7f). Yellow solid, m.p. = 119.1 - 120.5 °C; 47.7 mg, 89% yield; ^{1}H NMR (400 MHz, $CDCl_3$) (δ, ppm) 7.49 (d, *J* = 8.4 Hz, 1H), 7.22 - 7.16 (comp, 4H), 7.11 (d, *J* = 1.6 Hz, 1H), 7.01 - 6.95 (comp, 7H), 6.91 (s, 1H), 6.25 (s, 1H), 4.40 - 4.25 (m, 2H), 1.44 (t, *J* = 7.1 Hz, 3H), 1.35 (s, 9H); ^{13}C NMR (126 MHz, $CDCl_3$) (δ, ppm) 179.7, 177.1, 154.7, 152.8, 149.6, 141.3, 139.8, 137.7, 135.2, 133.8, 130.4, 129.8, 128.8, 128.3, 127.9, 127.4, 125.9, 121.5, 116.3, 114.6, 110.6, 99.3, 77.4, 68.3, 39.3, 27.2, 15.4; HRMS (TOF MS ESI$^+$) calculated for $C_{33}H_{29}NO_6Na$ [M+Na]$^+$: 558.1887, found 558.1889.

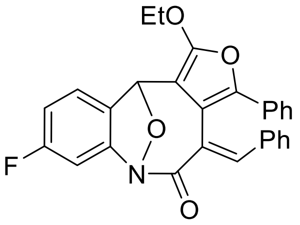

(*E*)-4-Benzylidene-1-ethoxy-8-fluoro-3-phenyl-11*H*-6,11-epoxybenzo[*b*]furo[3,4-*e*]azocin-5(4*H*)-one (7g). Yellow solid, m.p. = 175.4 - 176.4 °C; 38.5 mg, 85% yield; ^{1}H NMR (500 MHz, $CDCl_3$) (δ, ppm) 7.32 - 7.30 (m, 1H), 7.26 - 7.24 (m, 1H), 7.22 - 7.20 (m, 2H), 7.18 - 7.17 (m, 2H), 6.98 - 6.95 (comp, 6H), 6.92 - 6.88 (m, 2H), 6.24 (s, 1H), 4.39 - 4.25 (m, 2H), 1.45 (t, *J* = 7.1 Hz, 3H); ^{13}C NMR (126 MHz, $CDCl_3$) (δ, ppm) 179.3, 163.0 (d, *J* = 247.1 Hz), 152.6, 143.9 (d, *J* = 12.0 Hz), 141.2, 135.1, 134.0, 132.2 (d, *J* = 2.6 Hz), 130.3, 129.7, 128.8, 128.4, 127.9, 127.4, 125.8, 121.4 (d, *J* = 9.7 Hz), 113.3 (d, *J* = 23.1 Hz), 110.5, 104.6 (d, *J* = 27.5 Hz), 99.4, 77.2, 68.1, 15.4; ^{19}F NMR (376 MHz, $CDCl_3$) (δ, ppm) -112.17; HRMS (TOF MS ESI$^+$) calculated for $C_{28}H_{20}FNO_4Na$ [M+Na]$^+$: 476.1269, found 476.1273.

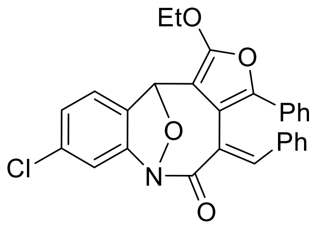

(*E*)-4-Benzylidene-8-chloro-1-ethoxy-3-phenyl-11*H*-6,11-epoxybenzo[*b*]furo[3,4-*e*]azocin-5(4*H*)-one (7h). Yellow solid, m.p. = 198.6 - 199.9 °C; 38.1 mg, 81% yield; ^{1}H NMR (400 MHz, $CDCl_3$) (δ, ppm) 7.44 (d, *J* = 8.2 Hz, 1H), 7.35 (s, 1H), 7.30 - 7.28 (m, 1H), 7.22 - 7.16 (comp, 4H), 6.96 - 6.95 (comp, 6H), 6.91 (s, 1H), 6.24 (s, 1H), 4.41 - 4.27 (m, 2H), 1.47 (t, *J* = 7.1 Hz, 3H); ^{13}C NMR (101 MHz, $CDCl_3$) (δ, ppm) 179.5, 152.7, 141.2, 141.1, 138.3, 135.1, 134.0, 132.0, 130.3, 129.6, 128.8, 128.4, 127.9, 127.4, 125.8, 121.2, 116.8, 110.5, 98.7, 77.3, 68.1, 15.4; HRMS (TOF MS ESI^{+}) calculated for $C_{28}H_{20}ClNO_4Na$ [M+Na]$^{+}$: 492.0973, found 492.0977.

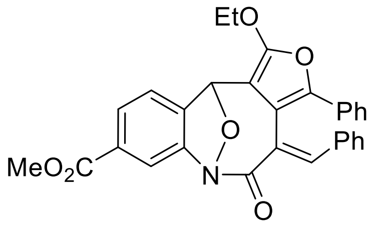

Methyl (*E*)-4-benzylidene-1-ethoxy-5-*oxo*-3-phenyl-4,5-dihydro-11*H*-6,11-epoxy benzo[*b*]furo[3,4-*e*]azocine-8-carboxylate (7i). Yellow solid, m.p. = 163.8 - 164.9 °C; 43.9 mg, 89% yield; ^{1}H NMR (400 MHz, $CDCl_3$) (δ, ppm) 8.15 (s, 1H), 7.97 (dd, *J* = 7.8, 1.2 Hz, 1H), 7.45 (d, *J* = 7.8 Hz, 1H), 7.21 - 7.17 (comp, 4H), 6.97 - 6.94 (comp, 7H), 6.31 (s, 1H), 4.41 - 4.25 (m, 2H), 3.93 (s, 3H), 1.46 (t, *J* = 7.1 Hz, 3H); ^{13}C NMR (101 MHz, $CDCl_3$) (δ, ppm) 179.4, 166.2, 152.7, 142.8, 141.3, 141.1, 135.1, 134.0, 131.3, 130.3, 129.5, 128.9, 128.8, 128.4, 127.9, 127.4, 125.8, 120.6, 116.6, 110.6, 98.6, 77.3, 68.1, 52.5, 15.4; HRMS (TOF MS ESI^{+}) calculated for $C_{30}H_{23}NO_6Na$ [M+Na]$^{+}$: 516.1418, found 516.1413.

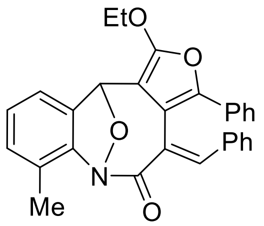

(*E*)-4-Benzylidene-1-ethoxy-7-methyl-3-phenyl-11*H*-6,11-epoxybenzo[*b*]furo[3,4-*e*]azocin-5(4*H*)-one (7j). Yellow solid, m.p. = 169.8 - 171.2 °C; 40.9 mg, 91% yield; ^{1}H NMR (500 MHz, $CDCl_3$) (δ, ppm) 7.25 - 7.23 (comp, 3H), 7.21 - 7.19 (m, 2H), 7.15 - 7.10 (m, 2H), 6.98 - 6.92 (comp, 6H), 6.86 (s, 1H), 6.20 (s, 1H), 4.39 - 4.32 (m, 1H), 4.31 - 4.25 (m, 1H), 2.66 (s, 3H), 1.45 (t, *J* = 7.1 Hz, 3H); ^{13}C NMR (126 MHz, $CDCl_3$) (δ, ppm) 181.0, 152.8, 141.9, 140.8, 137.0, 135.3, 132.4, 130.9, 130.54, 130.47, 128.8, 128.2, 127.9, 127.7, 127.2, 126.9, 125.7, 118.1, 111.1, 99.4, 77.6, 68.3, 19.1, 15.4; HRMS (TOF MS ESI$^+$) calculated for $C_{29}H_{23}NO_4Na$ $[M+Na]^+$: 472.1519, found 472.1514.

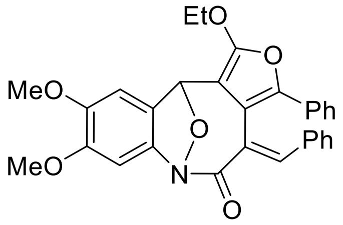

(*E*)-4-Benzylidene-1-ethoxy-8,9-dimethoxy-3-phenyl-11*H*-6,11-epoxybenzo[*b*]furo[3,4-*e*]azocin-5(4*H*)-one (7k). Yellow solid, m.p. = 194.3 - 195.7 °C; 41.1 mg, 83% yield; ^{1}H NMR (500 MHz, $CDCl_3$) (δ, ppm) 7.22 - 7.21 (m, 2H), 7.18 - 7.17 (m, 2H), 7.13 (s, 1H), 6.98 - 6.95 (comp, 6H), 6.92 (s, 1H), 6.90 (s, 1H), 6.21 (s, 1H), 4.40 - 4.33 (m, 1H), 4.32 - 4.27 (m, 1H), 3.94 (s, 3H), 3.88 (s, 3H), 1.46 (t, *J* = 7.1 Hz, 3H); ^{13}C NMR (126 MHz, $CDCl_3$) (δ, ppm) 180.5, 152.4, 149.6, 148.0, 141.1, 136.0, 135.2, 133.5, 130.4, 130.2, 128.8, 128.2, 127.9, 127.4, 127.3, 125.8, 110.7, 104.3, 100.6, 100.1, 77.8, 68.1, 56.6, 56.6, 15.5; HRMS (TOF MS ESI$^+$) calculated for $C_{30}H_{25}NO_6Na$ $[M+Na]^+$: 518.1574, found 518.1573.

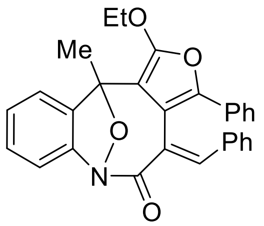

(*E*)-4-Benzylidene-1-ethoxy-11-methyl-3-phenyl-11*H*-6,11-epoxybenzo[*b*]furo[3,4-*e*]azocin-5(4*H*)-one (7l). Yellow solid, m.p. = 209.6 - 210.7 °C; 40.0 mg, 89% yield; ^{1}H NMR (500 MHz, $CDCl_3$) (δ, ppm) 7.49 (d, *J* = 7.7 Hz, 1H), 7.32 - 7.29 (m, 2H), 7.22 - 7.18 (comp, 5H), 6.98 - 6.93 (comp, 6H), 6.90 (s, 1H), 4.36 - 4.30 (m, 1H), 4.28 - 4.22 (m, 1H), 2.11 (s, 3H), 1.45 (t, *J* = 7.1 Hz, 3H); ^{13}C NMR (126 MHz, $CDCl_3$) (δ, ppm) 179.2, 152.4, 142.9, 140.5, 139.6, 135.3, 133.1, 130.4, 130.3, 128.8, 128.5, 128.2, 127.92, 127.90, 127.3, 126.7, 125.8, 119.7, 115.7, 111.0, 103.6, 84.8, 68.1, 21.1, 15.4; HRMS (TOF MS ESI^+) calculated for $C_{29}H_{23}NO_4Na$ $[M+Na]^+$: 472.1519, found 472.1516.

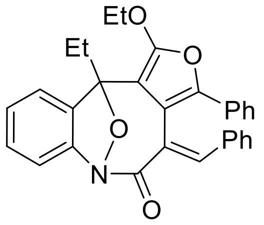

(*E*)-4-Benzylidene-1-ethoxy-11-ethyl-3-phenyl-11*H*-6,11-epoxybenzo[*b*]furo[3,4-*e*] azocin-5(4*H*)-one (7m). Yellow solid, m.p. = 207.9 - 209.2 °C; 43.1 mg, 93% yield; ^{1}H NMR (400 MHz, $CDCl_3$) (δ, ppm) 7.49 (d, *J* = 7.6 Hz, 1H), 7.30 (t, *J* = 7.3 Hz, 1H), 7.26 - 7.16 (comp, 6H), 6.99 - 6.96 (comp, 6H), 6.90 (s, 1H), 4.36 - 4.20 (m, 2H), 2.68 - 2.59 (m, 1H), 2.49 - 2.41 (m, 1H), 1.43 (t, *J* = 7.0 Hz, 3H), 1.12 (t, *J* = 7.2 Hz, 3H); ^{13}C NMR (101 MHz, $CDCl_3$) (δ, ppm) 179.2, 152.1, 143.2, 140.6, 138.4, 135.4, 132.9, 130.6, 130.4, 128.7, 128.4, 128.1, 127.9, 127.2, 126.6, 125.8, 119.9, 115.7, 111.6, 102.2, 88.1, 68.0, 26.8, 15.5, 8.2; HRMS (TOF MS ESI^+) calculated for $C_{30}H_{25}NO_4Na$ $[M+Na]^+$: 486.1676, found 486.1679.

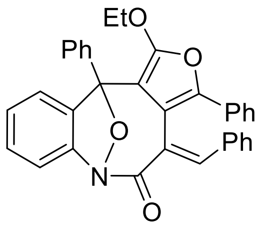

(*E*)-4-Benzylidene-1-ethoxy-3,11-diphenyl-11*H*-6,11-epoxybenzo[*b*]furo[3,4-*e*]azocin-5(4*H*)-one (7n). Yellow solid, m.p. = 197.2 - 198.5 °C; 43.5 mg, 85% yield; ^{1}H NMR (400 MHz, $CDCl_3$) (δ, ppm) 7.71 - 7.70 (m, 2H), 7.57 - 7.55 (m, 1H), 7.46 - 7.38 (comp, 3H), 7.36 (t, *J* = 7.5 Hz, 1H), 7.30 - 7.23 (comp, 6H), 7.00 - 6.96 (comp, 6H), 6.93 (s, 1H), 4.21 - 4.07 (m, 2H), 1.19 (t, *J* = 7.0 Hz, 3H); ^{13}C NMR (101 MHz, $CDCl_3$) (δ, ppm) 178.7, 152.7, 144.1, 140.6, 138.2, 136.3, 135.3, 133.0, 130.4, 130.3, 129.8, 129.3, 128.8, 128.7, 128.5, 128.3, 128.0, 127.3, 126.7, 125.7, 121.8, 115.4, 111.9, 101.7, 90.0, 67.6, 15.3; HRMS (TOF MS ESI$^+$) calculated for $C_{34}H_{25}NO_4Na$ $[M+Na]^+$: 534.1676, found 534.1674.

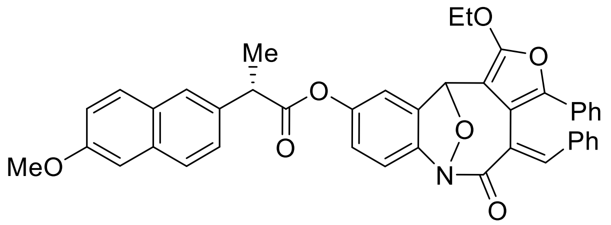

4-((*E*)-Benzylidene)-1-ethoxy-5-*oxo*-3-phenyl-4,5-dihydro-11*H*-6,11-epoxybenzo[*b*]furo[3,4-*e*]azocin-9-yl (2*S*)-2-(6-methoxynaphthalen-2-yl)propanoate (7o). Yellow solid, m.p. = 116.3 - 117.5 °C; 57.1 mg, 86% yield, 1:1 *dr*; ^{1}H NMR (500 MHz, $CDCl_3$) (δ, ppm) 7.75 - 7.71 (comp, 3H), 7.48 (d, *J* = 8.4 Hz, 1H), 7.44 (d, *J* = 8.4 Hz, 1H), 7.24 (s, 1H) 7.20 - 7.13 (comp, 6H), 7.01 - 6.99 (m, 1H), 6.94 - 6.91 (comp, 6H), 6.88 (s, 1H), 6.19 (s, 1H), 4.30 - 4.12 (m, 2H), 4.11 - 4.06 (m, 1H), 3.91 (s, 3H), 1.68 (d, *J* = 7.1 Hz, 3H), 1.28 - 1.24 (m, 3H); ^{13}C NMR (126 MHz, $CDCl_3$) (δ, ppm) 179.61, 179.60, 173.2, 157.9, 152.7, 149.32, 149.30, 141.19, 141.16, 139.87, 139.86, 137.70, 137.68, 135.1, 135.01, 134.99, 134.0, 133.8, 130.3, 129.8, 129.4, 129.1, 128.8, 128.3, 127.9, 127.5, 127.3, 126.3, 126.2, 126.1, 125.82, 125.80, 121.5, 121.4, 119.31, 119.29, 116.27, 116.25, 114.5, 110.6, 105.72, 105.71, 99.2, 99.1, 77.4, 77.3, 68.32, 68.27, 55.5, 45.6, 18.5, 15.3, 15.2; HRMS (TOF MS ESI$^+$) calculated for $C_{42}H_{33}NO_7Na$ $[M+Na]^+$: 686.2149, found 686.2145.

General Procedure for Scale Up and Synthetic Applications

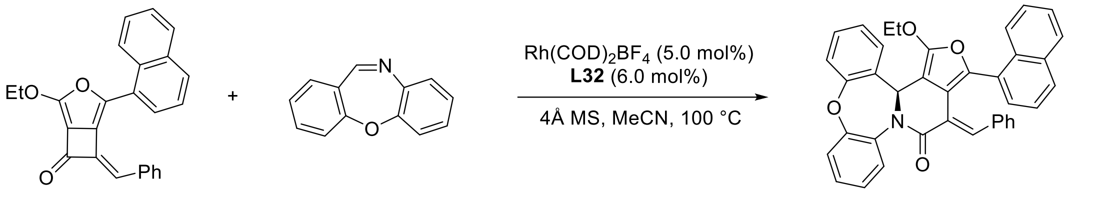

1n, 2.4 mmol **2a**, 2.0 mmol **4n**, 0.95 g, 85% yield, 95% *ee*

<u>Scale-up for the synthesis 4n</u>: To a 50-mL oven-dried vial containing a magnetic stirring bar, cyclobutanone **1n** (879.4 mg, 2.4 mmol, 1.2 equiv.), imine **2a** (390.2 mg, 2.0 mmol, 1.0 equiv.), 4Å MS (1.0 g), $Rh(COD)_2BF_4$ (40.0 mg, 5.0 mol%), and chiral ligand **L32** (46.0 mg, 6.0 mol%) in 10 mL MeCN under argon atmosphere at 100 ºC. After addition, the reaction mixture was stirred overnight under these conditions until consumption of the material (monitored by TLC). Then the solvent was evaporated under reduced pressure. The residues was purified by column chromatography on silica gel without any additional treatment (Hexanes : EtOAc = 50:1 to 20:1) to give 0.95 g pure product **4n** in 85% yield with 95% *ee*.

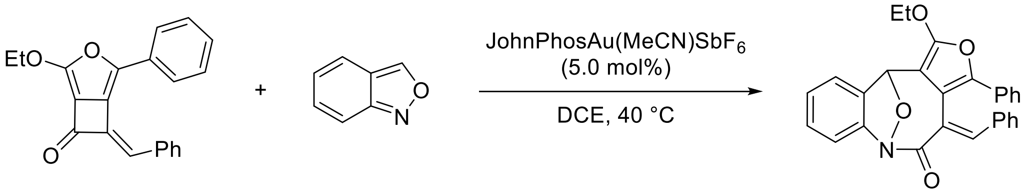

1a, 1.5 mmol **5a**, 1.65 mmol **6a**, 581 mg, 89% yield

<u>Scale up for the synthesis 6a</u>: To a 25-mL oven-dried vial containing a magnetic stirring bar, cyclobutanone **1a** (474.5 mg, 1.5 mmol, 1.0 equiv.), and anthranil **5a** (196.6 mg, 1.65 mmol, 1.1 equiv.) in DCE (5.0 mL), was added a solution of $JohnPhosAu(MeCN)SbF_6$ (57.9 mg, 5.0 mol%) in dry DCE (5.0 mL) slowly *via* a syringe under argon atmosphere at 40 ºC. After addition, the reaction mixture was stirred overnight under these conditions until consumption of the material (monitored by TLC). Then the solvent was evaporated under reduced pressure. The residues was purified by column chromatography on silica gel without any additional treatment (Hexanes : EtOAc = 30:1 to 10:1) to give 581 mg pure product **6a** in 89% yield.

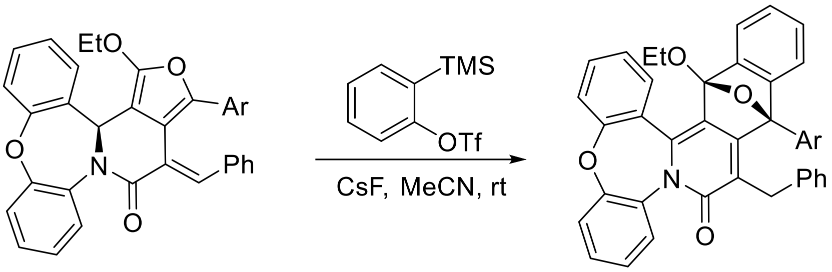

Ar = 1-naphthyl
4n, 95% *ee*

8, 82% yield
95% *ee*, *dr* > 20:1

Synthesis of 8: To a 10-mL oven-dried round bottom flask containing a magnetic stirring bar, 2-(trimethylsilyl)phenyltrifluoromethanesulfonate (89.4 mg, 0.3 mmol, 1.5 equiv.) in dry MeCN (1.0 mL), was added CsF (91.1 mg, 0.6 mmol, 3.0 equiv.) at room temperature, and the reaction mixture was stirred for 15 min under these conditions. Then a solution of **4n** (112.2 mg, 0.2 mmol, 1.0 equiv.) in dry MeCN (1.0 mL) was added slowly *via* a syringe at room temperature. After stirring for additional 3.0 h, the reaction was quenched with saturated aqueous NH_4Cl solution (3.0 mL) and the organic phase was separated, and the aqueous phase was extracted with EtOAc (3 × 5.0 mL). The combined organic phase was dried over anhydrous $MgSO_4$ and concentrated under reduced pressure after filtration. The obtained residues was purified by column chromatography on silica gel (Hexane/EtOAc = 20/1 - 10/1) to give the pure product **8** as white solid (104.5 mg, 82% yield). m.p. = 210. 5 - 212.2 °C; *dr* > 20:1, 95% *ee*; $[\alpha]_D^{20}$ = 27.00 (c = 0.033, CH_2Cl_2); ^{1}H NMR (400 MHz, $CDCl_3$) (δ, ppm) 8.30 (d, *J* = 7.2 Hz, 1H), 8.22 (d, *J* = 8.3 Hz, 1H), 8.01 (d, *J* = 8.2 Hz, 1H), 7.92 (d, *J* = 8.0 Hz, 1H), 7.65 (d, *J* = 7.2 Hz, 1H), 7.56 - 7.48 (comp, 4H), 7.46 - 7.41 (m, 1H), 7.35 - 7.28 (comp, 4H), 7.23 (s, 1H), 7.19 (t, *J* = 7.5 Hz, 2H), 7.15 - 7.08 (comp, 5H), 6.62 - 6.60 (m, 2H), 3.88 - 3.84 (m, 1H), 3.73 (d, *J* = 14.8 Hz, 1H), 3.17 - 3.13 (m, 1H), 2.61 (d, *J* = 14.8 Hz, 1H), 0.80 (t, *J* = 7.0 Hz, 3H); ^{13}C NMR (101 MHz, $CDCl_3$) (δ, ppm) 162.4, 162.2, 157.9, 155.8, 146.7, 145.7, 139.7, 135.1, 134.4, 132.5, 131.8, 131.2, 131.0, 129.7, 129.0, 128.92, 128.85, 128.0, 127.6, 127.2, 126.9, 126.3, 125.9, 125.8, 125.1, 124.9, 124.9, 124.8, 124.7, 124.6, 123.5, 121.1, 120.3, 119.7, 119.3, 110.6, 86.8, 62.4, 31.2, 14.8; HRMS (TOF MS ESI^+) calculated for $C_{44}H_{31}NO_4Na$ $[M+Na]^+$: 660.2145, found: 660.2140; HPLC conditions for determination of enantiomeric excess: Chiralpak IC, λ = 254 nm, hexane : 2-propanol

= 90:10, flow rate = 1.0 mL/min, t_{minor} = 15.4 min, t_{major} = 22.5min.

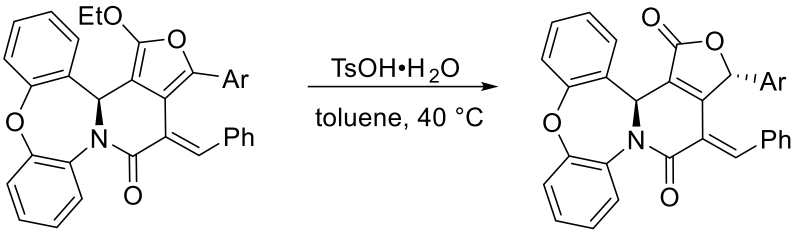

Ar = 1-naphthyl
4n, 95% *ee*

9, 91% yield
92% *ee*, *dr* > 20:1

Synthesis of 9: To a 10-mL oven-dried vial containing a magnetic stirring bar, *p*-toluenesulfonic acid ($TsOH{\cdot}H_2O$, 38.0 mg, 0.2 mmol, 2.0 equiv.), **4n** (56.1 mg, 0.1 mmol), and toluene (2.0 mL) were added in sequence, and the reaction mixture was stirred at 40 ºC for 1.0 h. After the reaction was completed (reaction monitored by TLC), the reaction mixture was diluted with H_2O (3.0 mL) and extracted with ethyl acetate (3 × 5.0 mL). The combined organic phase was dried over anhydrous $MgSO_4$ and concentrated under reduced pressure. The residues was purified by flash chromatography on silica gel without any additional treatment (Hexanes : EtOAc = 10:1 to 5:1) to afford pure product **9** as white solid (48.5 mg, 91% yield). m.p. = 258. 9 - 260.2 ℃; *dr* > 20:1, 92% *ee*; $[\alpha]_D^{20}$ = 27.00 (c = 0.033, CH_2Cl_2); ^1H NMR (500 MHz, $CDCl_3$) (δ, ppm) 8.12 (s, 1H), 7.75 - 7.71 (m, 2H), 7.52 - 7.48 (m, 2H), 7.44 - 7.42 (m, 1H), 7.40 - 7.37 (m, 1H), 7.35 - 7.21 (comp, 6H), 7.13 - 7.10 (m, 1H), 6.92 - 6.91 (m, 2H), 6.84 - 6.79 (m, 3H), 6.71 - 6.69 (m, 2H), 6.23 (s, 1H); ^{13}C NMR (126 MHz, $CDCl_3$) (δ, ppm) 169.7, 163.4, 157.1, 154.6, 152.2, 146.2, 134.6, 133.5, 132.3, 131.5, 130.6, 130.0, 129.4, 129.3, 128.58, 128.55, 128.5, 128.4, 128.2, 127.6, 127.0, 126.5, 126.0, 125.8, 124.9, 124.5, 124.3, 123.1, 122.4, 121.8, 121.7, 79.1, 56.5; HRMS (TOF MS ESI$^+$) calculated for $C_{36}H_{23}NO_4Na$ $[M+Na]^+$: 556.1519, found 556.1523; HPLC conditions for determination of enantiomeric excess: Chiralpak IC, λ = 254 nm, hexane : 2-propanol = 70:30, flow rate = 1.0 mL/min, t_{minor} = 21.3 min, t_{major} = 16.7min.

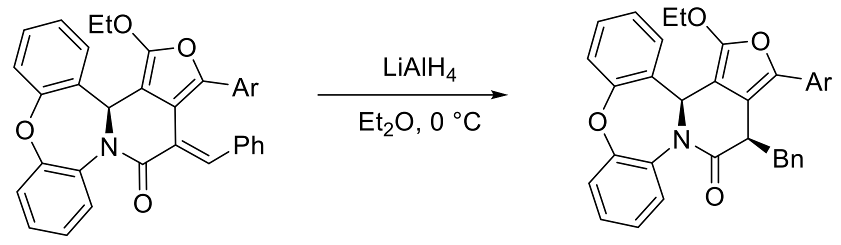

Ar = 1-naphthyl
4n, 95% *ee*

10, 80% yield
92% *ee*, *dr* > 20:1

Synthesis of 10: To a 10-mL oven-dried round-bottom flask with a magnetic stirring bar, and **4n** (56.1 mg, 0.1 mmol) in dry Et_2O (2.0 mL), was added $LiAlH_4$ (3.8 mg, 0.1 mmol, 1.0 equiv.) at 0 °C, and the reaction mixture was stirred for 1.0 h under these conditions. After the reaction was completed (reaction monitored by TLC), the reaction mixture was quenched with saturated aqueous ammonium chloride (3.0 mL) and extracted with ethyl acetate (3 × 5.0 mL). The combined organic phase was dried over anhydrous $MgSO_4$ and concentrated under reduced pressure. The residues was purified by flash chromatography on silica gel without any additional treatment (Hexanes : EtOAc = 15:1) to give pure product **10** as colorless oil (45.1 mg, 80% yield). *dr* > 20:1, 92% *ee*; $[\alpha]_D^{20}$ = 162.11 (c = 0.033, CH_2Cl_2); ^{1}H NMR (500 MHz, $CDCl_3$) (δ, ppm) 7.73 - 7.69 (m, 2H), 7.63 (d, *J* = 8.2 Hz, 1H), 7.40 - 7.34 (m, 3H), 7.30 (d, *J* = 7.7 Hz, 1H), 7.27 - 7.22 (m, 3H), 7.19 - 7.07 (comp, 5H), 6.85 (d, *J* = 7.0 Hz, 1H), 6.48 - 6.45 (m, 2H), 6.40 - 6.39 (m, 2H), 6.00 (s, 1H), 4.35 - 4.22 (m, 2H), 4.03 (dd, *J* = 11.3, 5.2 Hz, 1H), 2.87 (dd, *J* = 13.0, 5.1 Hz, 1H), 2.22 - 2.11 (m, 1H), 1.33 (t, *J* = 7.1 Hz, 3H); ^{13}C NMR (126 MHz, $CDCl_3$) (δ, ppm) 171.8, 154.3, 153.7, 152.3, 139.3, 137.2, 134.3, 133.8, 131.5, 129.7, 129.1, 129.0, 128.9, 128.8, 128.72, 128.71, 128.2, 127.6, 127.5, 127.3, 126.2, 126.1, 125.8, 125.7, 125.1, 124.4, 123.2, 121.8, 121.3, 119.9, 94.8, 68.2, 57.1, 44.6, 39.5, 15.4.; HRMS (TOF MS ESI$^+$) calculated for $C_{38}H_{29}NO_4Na$ $[M+Na]^+$: 586.1989, found 586.1990; HPLC conditions for determination of enantiomeric excess: Chiralpak IC, λ = 254 nm, hexane : 2-propanol = 90:10, flow rate = 1.0 mL/min, t_{minor} = 14.9 min, t_{major} = 19.3 min.

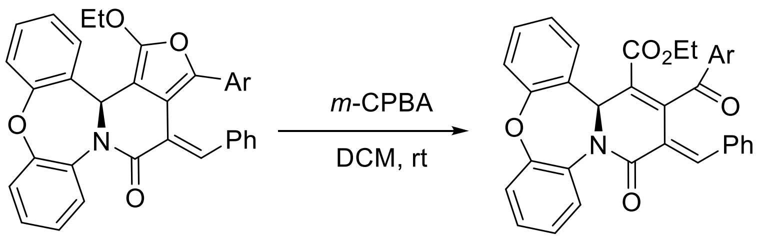

Ar = 1-naphthyl
4n, 95% *ee*

11, 86% yield, 95% *ee*

Synthesis of 11: To a 10-mL oven-dried vial containing a magnetic stirring bar, and **4n** (56.1 mg, 0.1 mmol) in DCM (2.0 mL), was added *m*-CPBA (25.9 mg, 0.15 mmol, 1.5 equiv.) at room temperature. The reaction mixture was stirred for 0.5 h under these conditions, After the reaction was completed (reaction monitored by TLC), the reaction mixture was treated with saturated aqueous $NaHCO_3$ (3.0 mL) and $Na_2S_2O_3$ (3.0 mL). The reaction mixture was extracted with EtOAc (3 × 5.0 mL). The combined organic extract was washed with brine, dried with anhydrous Na_2SO_4, and concentrated under reduced pressure after filtration. Then, the residue was purified by flash chromatography on silica gel (Hexanes : EtOAc = 5:1) to give 49.6 mg of pure product **11** as colorless oil (61.3 mg, 72% yield). 95% *ee*; $[\alpha]_D^{20}$ = 156.01 (c = 0.033, CH_2Cl_2); ^{1}H NMR (500 MHz, $CDCl_3$) (δ, ppm) 8.04 (d, *J* = 8.6 Hz, 1H), 7.93 (d, *J* = 8.1 Hz, 1H), 7.84 (s, 1H), 7.75 (d, *J* = 8.1 Hz, 1H), 7.66 (d, *J* = 7.2 Hz, 1H), 7.62 - 7.58 (m, 2H), 7.43 - 7.37 (m, 2H), 7.35 - 7.28 (comp, 4H), 7.26 - 7.22 (m, 2H), 7.19 (t, *J* = 7.6 Hz, 1H), 7.15 - 7.13 (m, 1H), 7.08 (t, *J* = 7.1 Hz, 2H), 6.92 (d, *J* = 7.4 Hz, 2H), 6.26 (s, 1H), 4.12 - 4.08 (m, 2H), 0.99 - 0.96 (m, 3H); ^{13}C NMR (126 MHz, $CDCl_3$) (δ, ppm) 193.6, 165.0, 164.5, 155.1, 149.6, 148.1, 145.7, 134.3, 133.8, 133.6, 131.8, 131.6, 130.7, 130.5, 130.3, 130.1, 129.9, 129.1, 128.8, 128.2, 128.14, 128.11, 128.05, 126.4, 126.3, 125.9, 125.7, 124.2, 124.0, 122.4, 121.5, 121.2, 62.2, 58.8, 13.6; HRMS (TOF MS ESI$^+$) calculated for $C_{38}H_{27}NO_5Na$ $[M+Na]^+$: 600.1781, found: 600.1783; HPLC conditions for determination of enantiomeric excess: Chiralpak IC, λ = 254 nm, hexane : 2-propanol = 90:10, flow rate = 1.0 mL/min, t_{minor} = 18.5 min, t_{major} = 29.5 min.

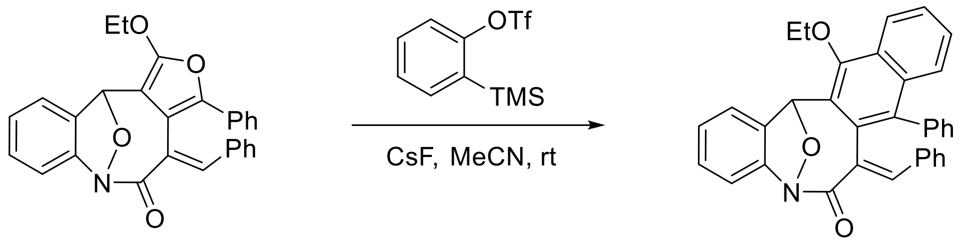

6a   **12**, 90% yield

Synthesis of 12: To a 10-mL oven-dried round bottom flask containing a magnetic stirring bar, and 2-(trimethylsilyl)phenyltrifluoromethanesulfonate (44.7 mg, 0.15 mmol, 1.5 equiv.) in dry MeCN (1.0 mL), was added CsF (45.6 mg, 0.3 mmol, 3.0 equiv.) at room temperature, and the reaction mixture was stirred for 15 min under these conditions. Then a solution of **6a** (43.5 mg, 0.1 mmol, 1.0 equiv.) in dry MeCN (1.0 mL) was added slowly *via* a syringe at room temperature. After stirring for additional 3.0 h, the reaction was quenched with saturated aqueous NH_4Cl solution (5.0 mL) and the organic phase was separated, and the aqueous phase was extracted with EtOAc (3 × 5.0 mL). The combined organic phase was dried over anhydrous $MgSO_4$ and concentrated under reduced pressure after filtration. The obtained residues was purified by column chromatography on silica gel (Hexane/EtOAc = 20/1 - 10/1) to give the pure product **12** as white solid (44.6 mg, 90% yield). m.p. = 184.8. 5 - 185.8 °C; ^{1}H NMR (500 MHz, $CDCl_3$) (δ, ppm) 7.49 (d, J = 6.2 Hz, 1H), 7.39 (d, J = 7.8 Hz, 2H), 7.19 - 7.12 (comp, 5H), 6.98 - 6.93 (comp, 6H), 6.91 - 6.86 (m, 2H), 6.75 (d, J = 7.1 Hz, 1H), 6.68 (t, J = 7.5 Hz, 1H), 6.60 (t, J = 7.4 Hz, 1H), 6.25 (s, 1H), 4.22 - 4.15 (m, 2H), 1.57 (t, J = 7.0 Hz, 3H); ^{13}C NMR (126 MHz, $CDCl_3$) (δ, ppm) 175.4, 150.3, 149.6, 145.1, 143.7, 143.5, 137.5, 134.9, 134.0, 129.8, 129.4, 128.9, 128.7, 128.3, 128.0, 127.7, 127.6, 125.9, 125.7, 125.3, 124.1, 122.4, 120.6, 119.4, 115.6, 113.0, 89.5, 80.5, 63.4, 16.1; HRMS (TOF MS ESI$^+$) calculated for $C_{34}H_{25}NO_3Na$ [M+Na]$^+$: 518.1727, found: 518.1725.

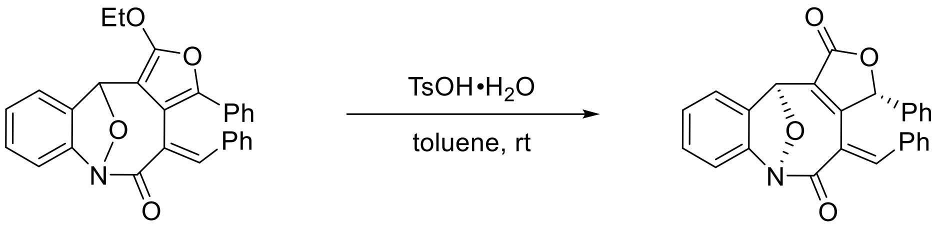

6a | **13**, 83% yield, *dr* > 20:1

Synthesis of 13: To a 10-mL oven-dried vial containing a magnetic stirring bar, *p*-toluenesulfonic acid ($TsOH\cdot H_2O$, 38.0 mg, 0.2 mmol, 2.0 equiv.), **6a** (43.5 mg, 0.1 mmol), and toluene (2.0 mL) were added in sequence, and the reaction mixture was stirred under 40 °C for 1.0 h. After the reaction was completed (reaction monitored by TLC), the reaction mixture was diluted with H_2O (3.0 mL) and extracted with ethyl acetate (3 × 5.0 mL). The combined organic phase was dried over anhydrous $MgSO_4$ and concentrated under reduced pressure after filtration. The residues was purified by flash chromatography on silica gel without any additional treatment (Hexanes : EtOAc = 10:1 to 5:1) to afford pure product **13** as white solid (33.8 mg, 83% yield). m.p. = 119.1 - 120.2 °C; > 20:1 *dr*; ^{1}H NMR (500 MHz, $CDCl_3$) (δ, ppm) 7.63 (d, *J* = 7.4 Hz, 1H), 7.53 - 7.46 (comp, 4H), 7.41 (t, *J* = 7.7 Hz, 1H), 7.38 (s, 1H), 7.34 (d, *J* = 7.2 Hz, 2H), 7.30 (t, *J* = 7.5 Hz, 1H), 7.19 (t, *J* = 7.4 Hz, 1H), 7.04 (t, *J* = 7.7 Hz, 2H), 6.19 - 6.16 (m, 3H), 5.81 (d, *J* = 1.8 Hz, 1H); ^{13}C NMR (126 MHz, $CDCl_3$) (δ, ppm) 176.2, 169.6, 152.3, 143.9, 142.7, 134.2, 133.1, 133.0, 131.9, 130.8, 130.5, 130.0, 129.9, 129.7, 129.2, 128.8, 127.14, 127.06, 122.1, 115.9, 83.2, 78.7; HRMS (TOF MS ESI$^+$) calculated for $C_{26}H_{17}NO_4Na$ $[M+Na]^+$: 430.1050, found 430.1050.

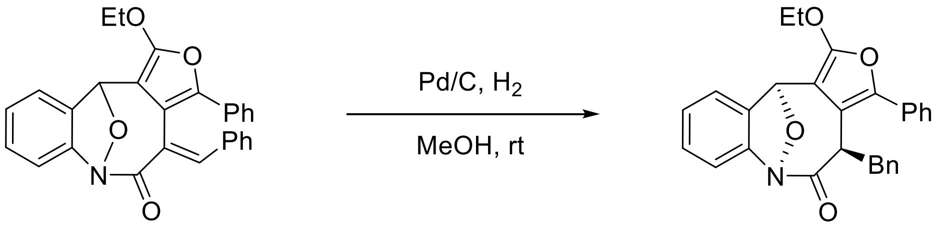

6a | **14**, 61% yield, *dr* > 20:1

Synthesis of 14: To a 10-mL hydrogenation reactor containing a magnetic stirring bar, and **6a** (43.5 mg, 0.1 mmol) in 1.5 mL of MeOH, was added wet 10% Pd/C (10 mg, 20 %). The heterogeneous mixture was placed under 40-60 psi H_2 atmosphere

and stirred overnight at room temperature. After the reaction was completed, as indicated by LC-MS analysis. The mixture was filtered through a pad of Celite to remove Pd/C, and the solid was washed with MeOH. The combined filtrate was concentrated under vacuum. Then, the residues was purified by flash chromatography on silica gel without any additional treatment (Hexanes : EtOAc = 15:1 to 5:1) to afford pure product **14** as white solid (26.7 mg, 61% yield). m.p. = 155.5 - 156.5 °C; > 20:1 *dr*; ^{1}H NMR (500 MHz, $CDCl_3$) (δ, ppm) 7.42 (d, *J* = 7.7 Hz, 1H), 7.38 - 7.32 (comp, 6H), 7.30 - 7.27 (m, 1H), 7.24 - 7.19 (comp, 3H), 7.16 - 7.13 (comp, 3H), 6.19 (s, 1H), 5.20 - 5.17 (m, 1H), 4.27 - 4.17 (m, 2H), 3.00 - 2.95 (m, 1H), 2.78 - 2.74 (m, 1H), 1.40 (t, *J* = 7.1 Hz, 3H); ^{13}C NMR (126 MHz, $CDCl_3$) (δ, ppm) 181.5, 152.9, 143.0, 140.8, 140.1, 137.2, 131.8, 131.4, 129.2, 129.1, 128.4, 128.3, 128.0, 126.8, 126.3, 120.4, 117.2, 114.6, 97.8, 78.0, 67.7, 44.6, 33.5, 15.4; HRMS (TOF MS ESI$^+$) calculated for $C_{28}H_{23}NO_4Na$ $[M+Na]^+$: 460.1519, found 460.1521.

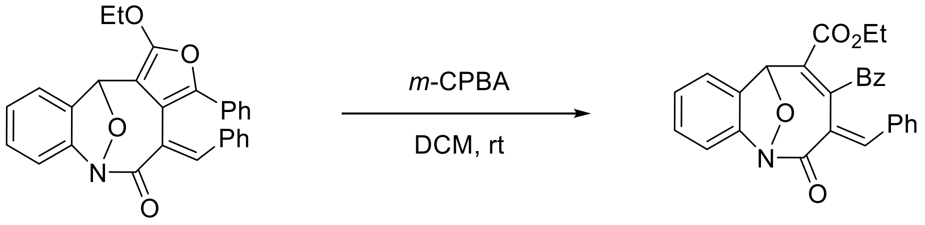

6a **15**, 92% yield

Synthesis of 15: To a 10-mL oven-dried vial containing a magnetic stirring bar, and **6a** (56.1 mg, 0.1 mmol) in DCM (2.0 mL), was added *m*-CPBA (25.9 mg, 0.15 mmol, 1.5 equiv.) at room temperature. The reaction mixture was stirred for 0.5 h under these conditions, After the reaction was completed (reaction monitored by TLC), the reaction mixture was treated with saturated aqueous $NaHCO_3$ (3.0 mL) and $Na_2S_2O_3$ (3.0 mL) in sequence. The reaction mixture was extracted with EtOAc (3 × 5.0 mL). The combined organic extract was washed with brine, dried with anhydrous Na_2SO_4, and concentrated under reduced pressure after filtration. Then, the residues was purified by flash chromatography on silica gel (Hexanes : EtOAc = 5:1) to afford pure product **15** as white solid (41.8 mg, 92% yield). m.p. = 227.4 - 228.5 °C; ^{1}H NMR (500 MHz, $CDCl_3$) (δ, ppm) 7.62 (d, *J* = 7.8 Hz, 1H), 7.43 (t, *J* = 7.6 Hz, 1H), 7.37 (t,

J = 7.3 Hz, 1H), 7.31 - 7.27 (m, 2H), 7.25 - 7.20 (comp, 4H), 7.17 (t, *J* = 7.7 Hz, 2H), 7.13 - 7.10 (comp, 4H), 6.43 (s, 1H), 4.05 - 3.99 (m, 2H), 0.95 (t, *J* = 7.1 Hz, 3H); ^{13}C NMR (126 MHz, $CDCl_3$) (δ, ppm) 193.1, 178.2, 164.7, 142.4, 142.0, 139.4, 138.9, 135.6, 134.0, 133.2, 132.9, 130.7, 130.0, 129.7, 128.8, 128.1, 128.0, 127.1, 121.7, 116.7, 82.1, 62.2, 13.4; HRMS (TOF MS ESI$^+$) calculated for $C_{28}H_{21}NO_5Na$ $[M+Na]^+$: 474.1312, found 474.1313.

1D-NOE NMR Analysis of 10 and 13

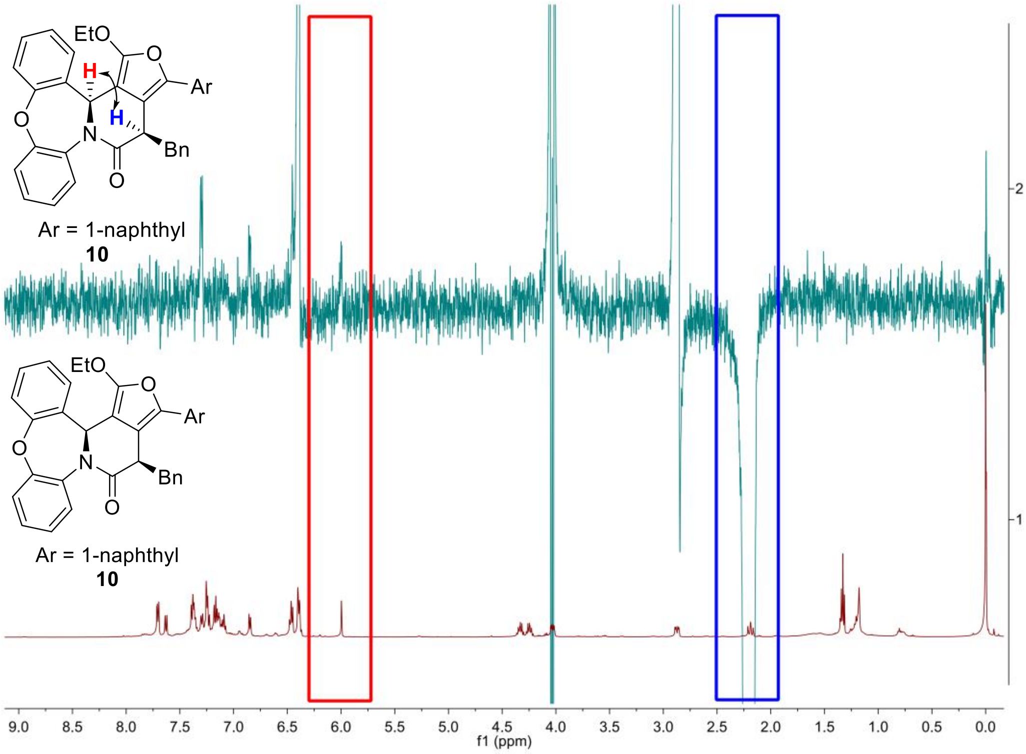

Supplementary Fig. 1 | NOE NMR Spectra of 10.

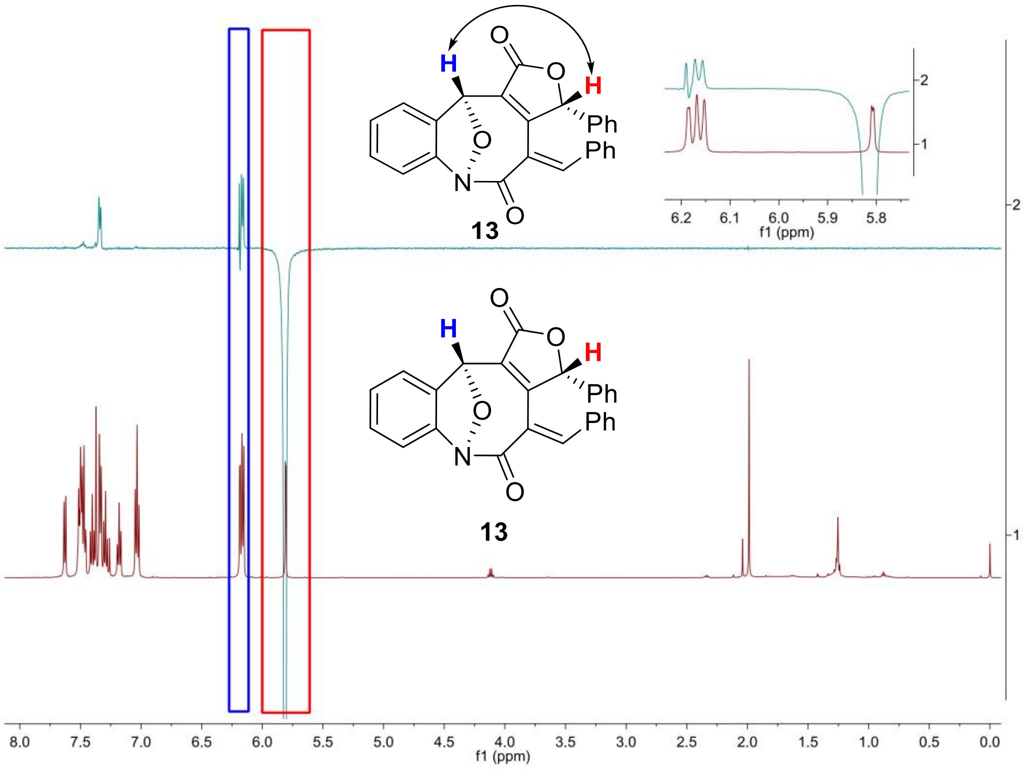

Supplementary Fig. 2 | NOE NMR Spectra of 13.

Control Experiments

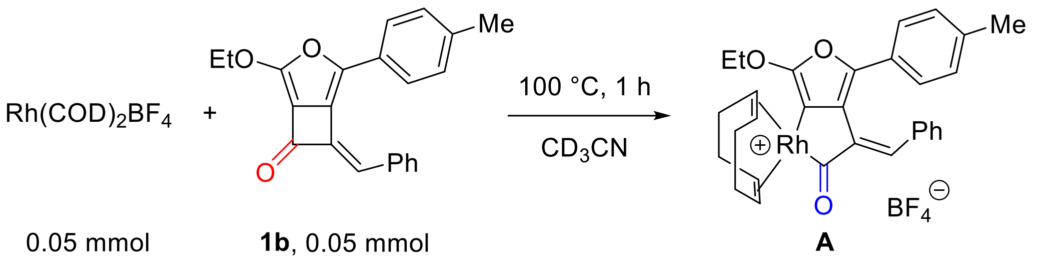

A solution of **1b** (16.5 mg, 0.05 mmol) in CD_3CN (0.7 mL) was added to an oven-dried NMR tube, and the mixture was subjected to ^{13}C NMR analysis (Supplementary Fig 3, bottom spectrum). The carbonyl signal of **1b** was observed in 174.7 ppm. Then, $Rh(COD)_2BF_4$ (20.3 mg, 0.05 mmol) was added to the above NMR tube, and the NMR tube was subjected to a 100 °C oil bath for 1 h. Then, the reaction mixture was subjected to ^{13}C NMR analysis (Supplementary Fig 3, up spectrum). The carbonyl signal of possible C-C bond activation intermediate **A** was observed in 202.7 ppm. This intermediate is relatively stable under these conditons.

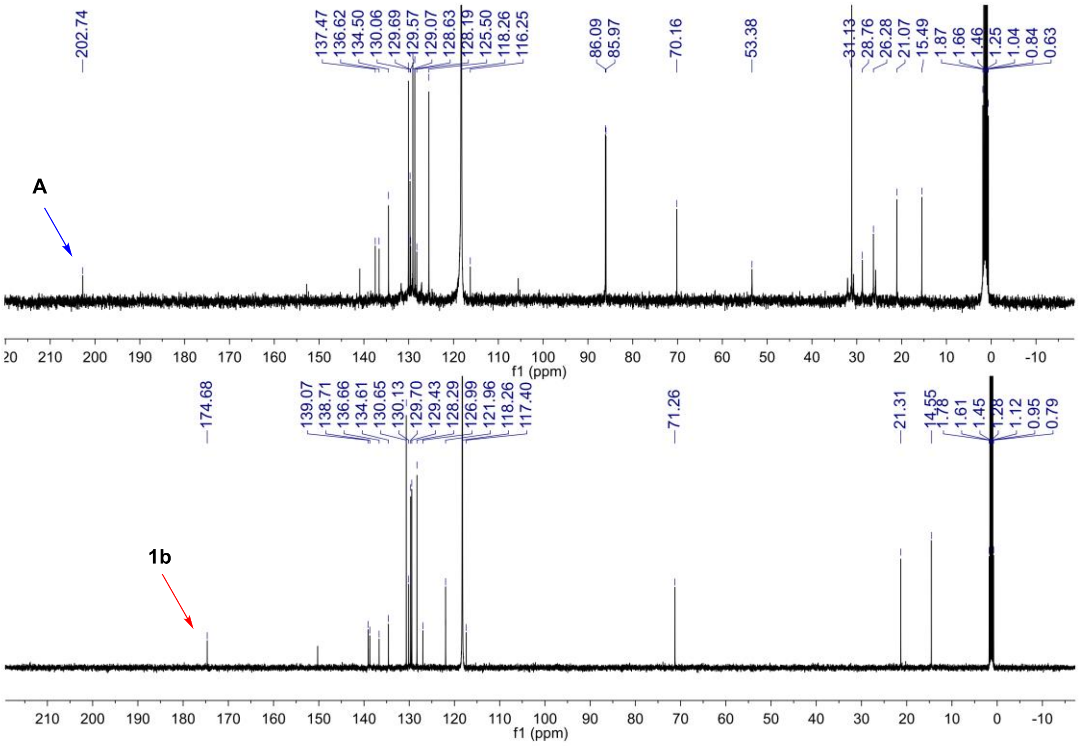

Supplementary Fig. 3 | ^{13}C NMR Observation of compound **1b** and **1b** treated with rhodium complex in CD_3CN.

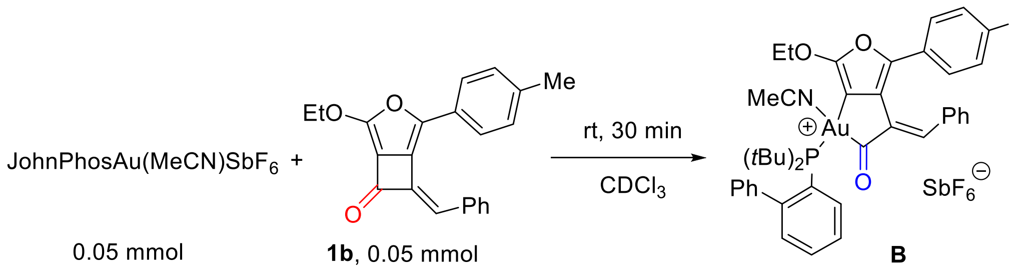

A solution of **1b** (16.5 mg, 0.05 mmol) in $CDCl_3$ (0.7 mL) was added to an oven-dried NMR tube, and the mixture was subjected to ^{13}C NMR analysis (Supplementary Fig 4, bottom spectrum). The carbonyl signal of **1b** was observed in 174.7 ppm. Then, JohnPhosAu(MeCN)SbF_6 (38.6 mg, 0.05 mmol) was added to the above NMR tube at room temperature. The reaction mixture was subjected to ^{13}C NMR analysis after 30 mins (Supplementary Fig 4, up spectrum). The carbonyl signal of possible C-C bond activation intermediate **B** was observed in 194.3 ppm. The material **1b** decomposed to complex mixture under these conditions within 5.0 mins.

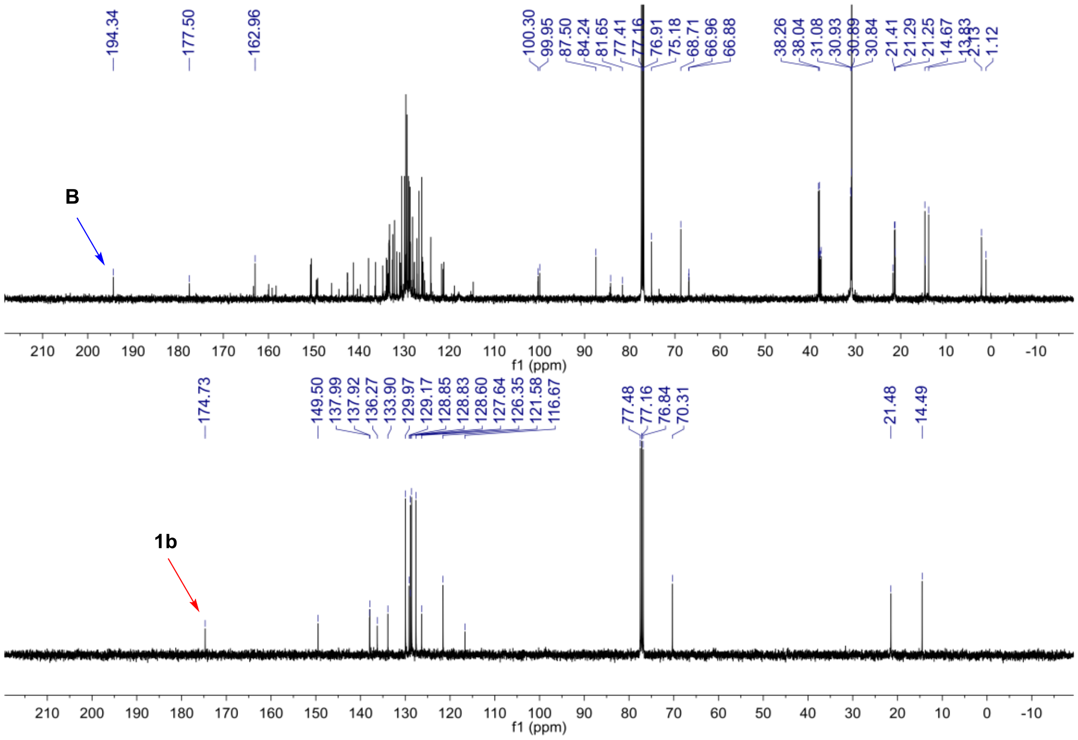

Supplementary Fig. 4 | ^{13}C NMR Observation of compound **1b** and **1b** treated with gold complex in $CDCl_3$.

NMR Spectra of New Compounds 3, 4, and 6-15

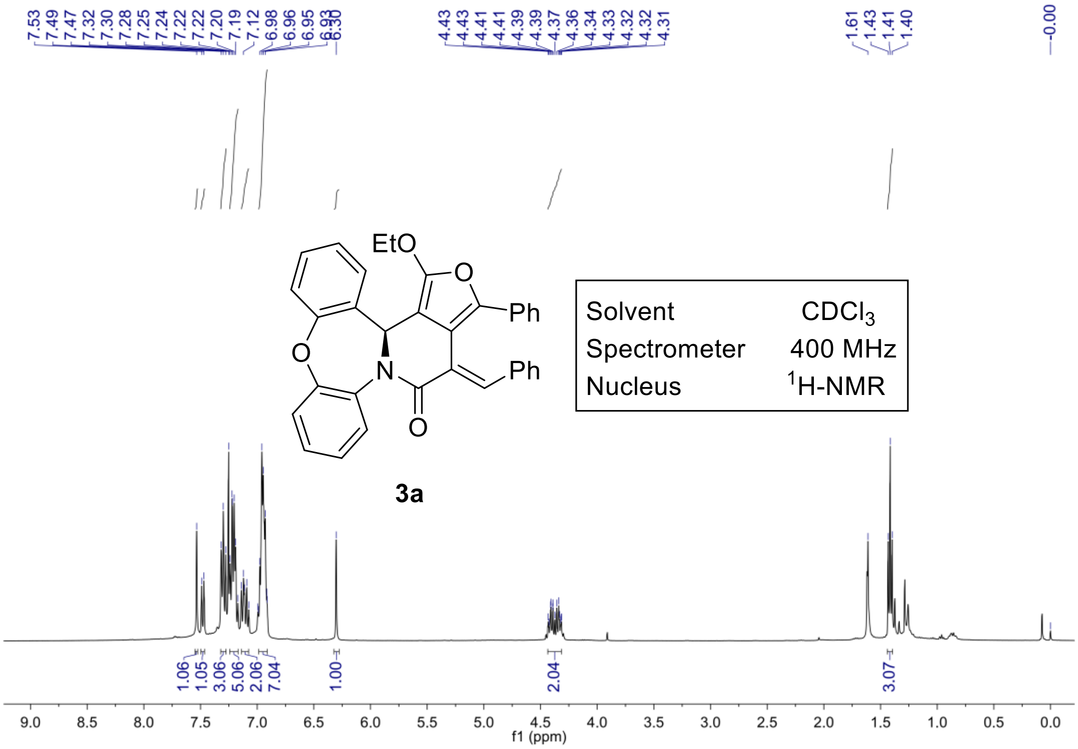

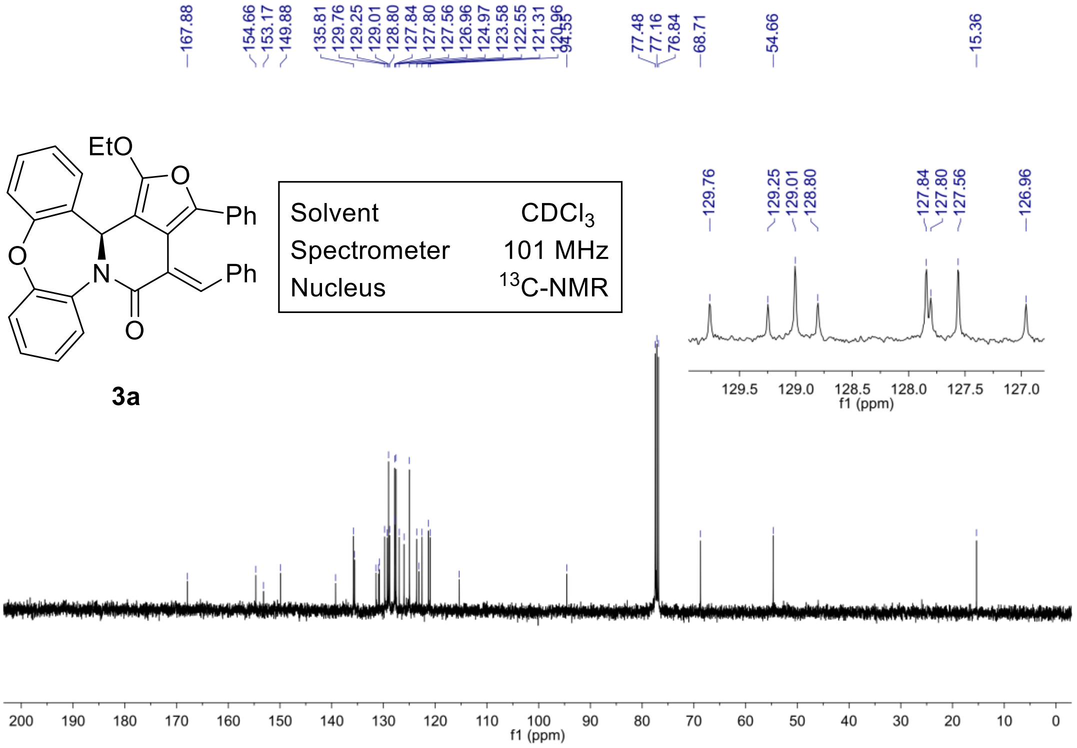

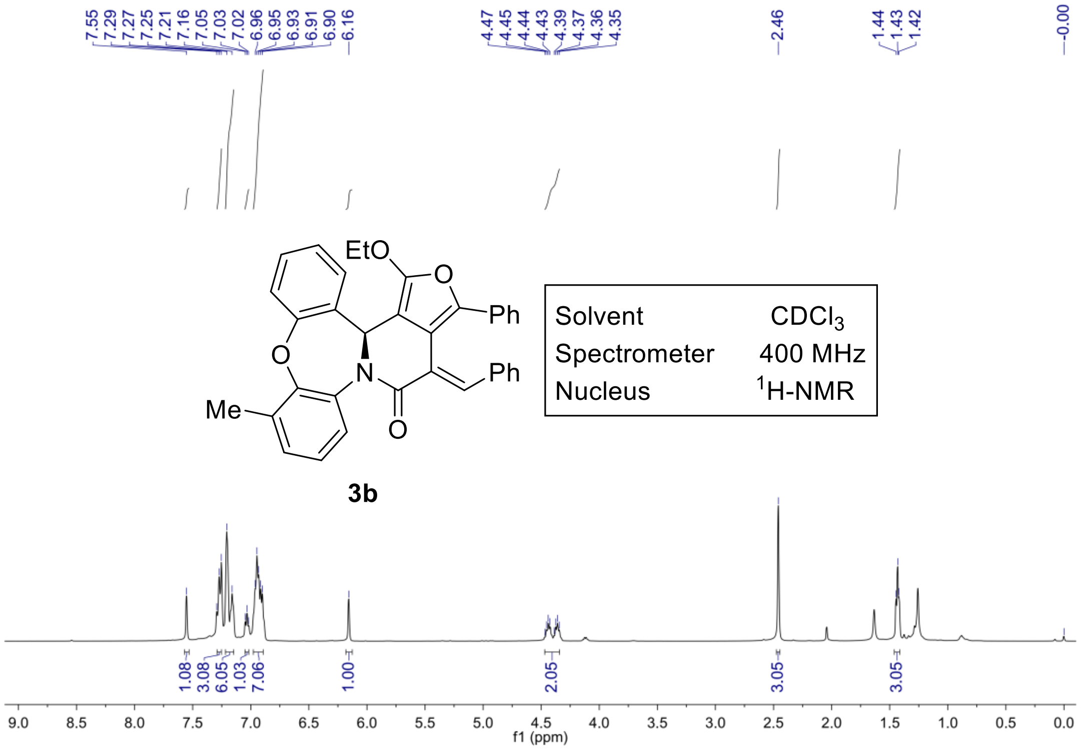
7.55 7.29 7.27 7.25 7.21 7.16 7.05 7.03 7.02 6.96 6.95 6.93 6.91 6.90 6.16
4.47 4.45 4.44 4.43 4.39 4.37 4.36 4.35
2.46
1.44 1.43 1.42
0.00
EtO
O
Ph
O
N
Ph
Me
O
3b
Solvent CDCl3
Spectrometer 400 MHz
Nucleus 1H-NMR
1.08 3.08 6.05 1.03 7.06 1.00 2.05 3.05 3.05
f1 (ppm)

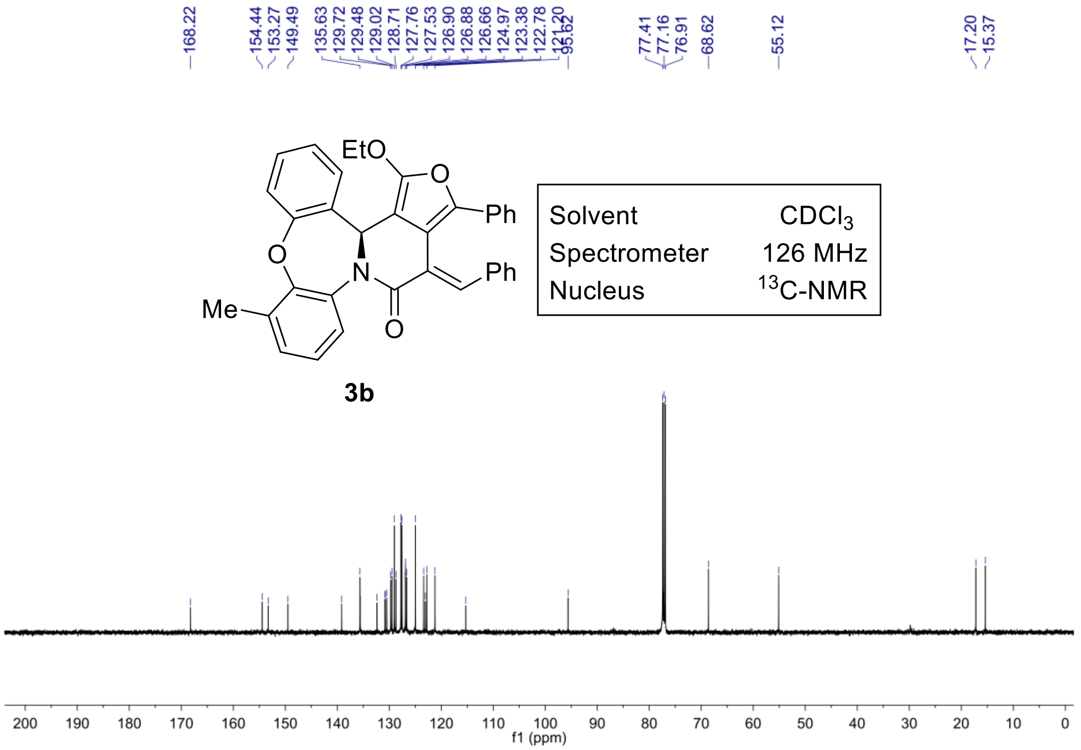
168.22
154.44 153.27 149.49
135.63 129.72 129.48 129.02 128.71 127.76 127.53 126.90 126.88 126.66 124.97 123.38 122.78 121.20 95.62
77.41 77.16 76.91 68.62
55.12
17.20 15.37
EtO
O
Ph
O
N
Ph
Me
O
3b
Solvent CDCl3
Spectrometer 126 MHz
Nucleus 13C-NMR
f1 (ppm)

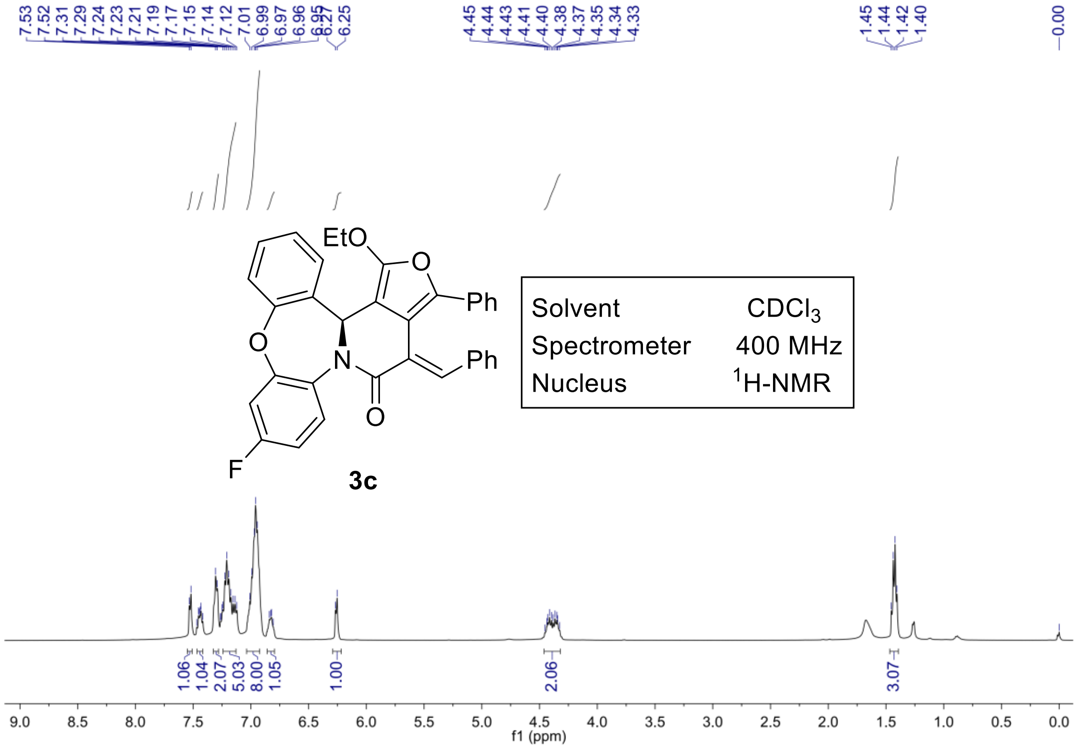
EtO
O
Ph
O
N
Ph
O
F
3c
Solvent CDCl3
Spectrometer 400 MHz
Nucleus 1H-NMR
f1 (ppm)

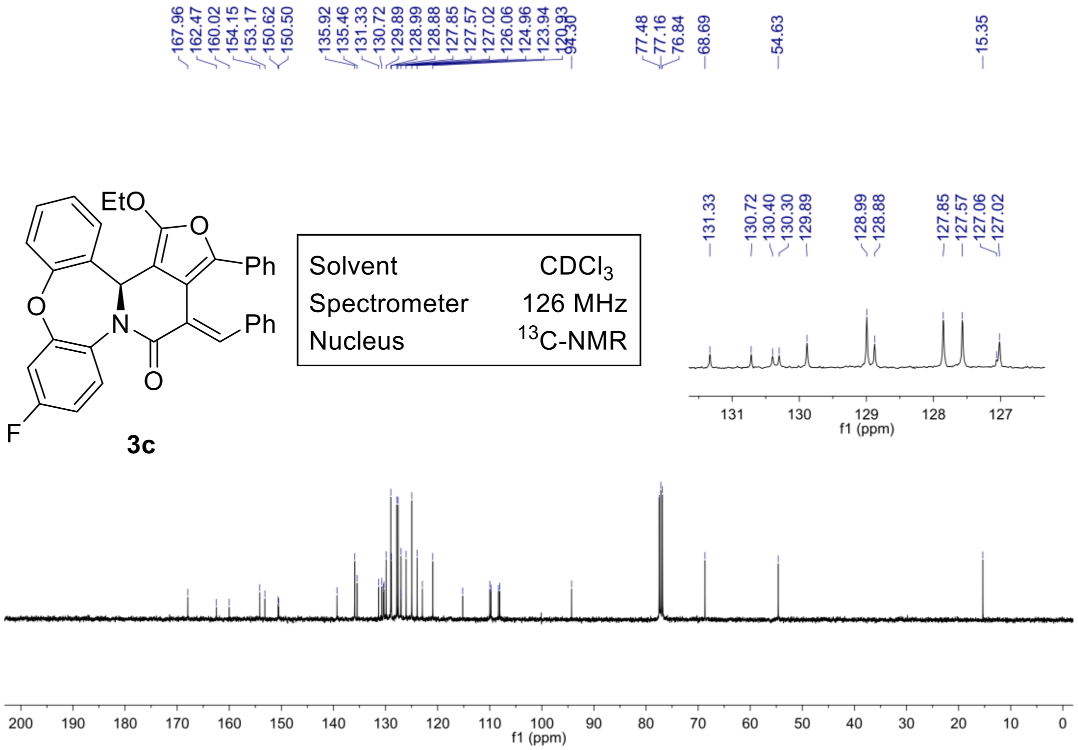
EtO
O
Ph
O
N
Ph
O
F
3c
Solvent CDCl3
Spectrometer 126 MHz
Nucleus 13C-NMR
f1 (ppm)

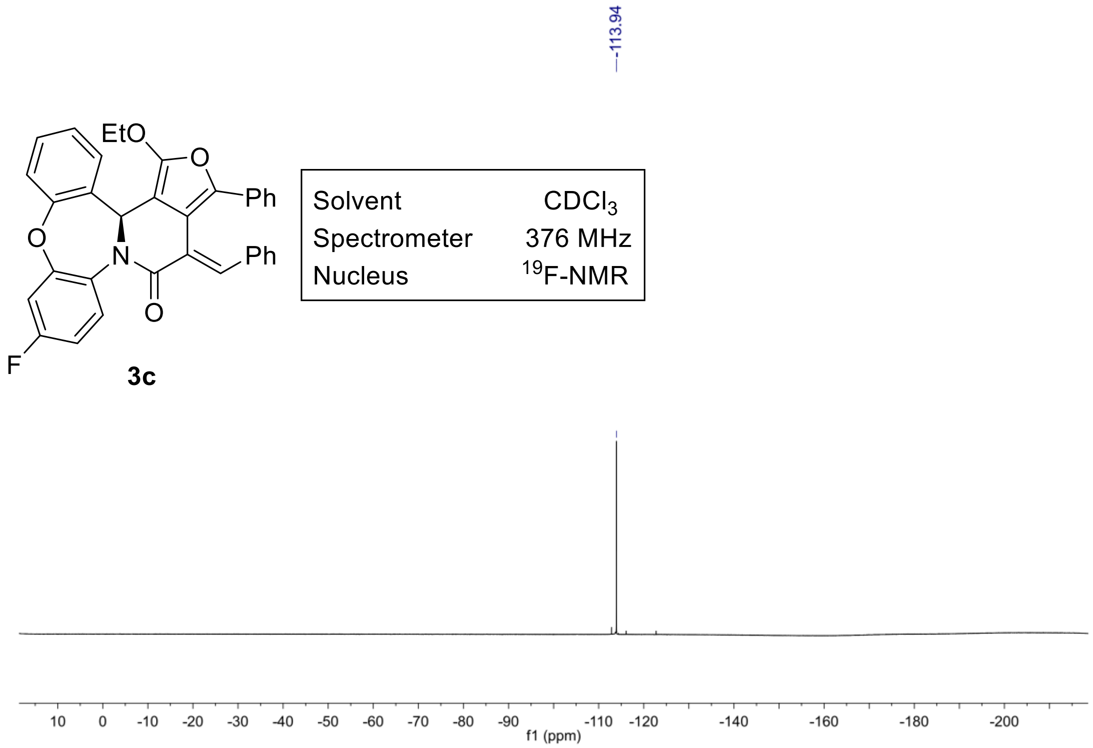
-113.94
EtO
O
Ph
Ph
N
O
O
F
3c
Solvent CDCl3
Spectrometer 376 MHz
Nucleus 19F-NMR
10 0 -10 -20 -30 -40 -50 -60 -70 -80 -90 -110 -120 -140 -160 -180 -200
f1 (ppm)

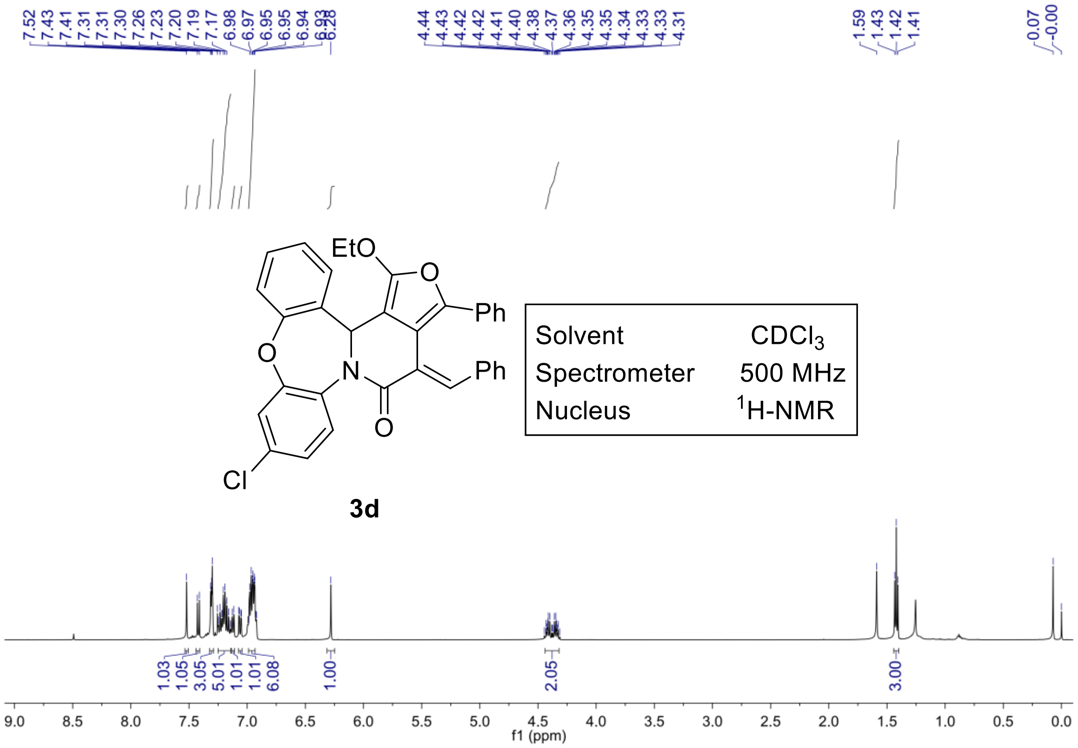
7.52 7.43 7.41 7.31 7.31 7.30 7.26 7.23 7.20 7.19 7.17 6.98 6.97 6.95 6.95 6.94 6.93 6.28
4.44 4.43 4.42 4.42 4.41 4.40 4.38 4.37 4.36 4.35 4.35 4.34 4.33 4.33 4.31
1.59 1.43 1.42 1.41
0.07 -0.00
EtO
O
Ph
Ph
N
O
O
Cl
3d
Solvent CDCl3
Spectrometer 500 MHz
Nucleus 1H-NMR
1.03 1.05 3.05 5.01 1.01 1.01 6.08 1.00 2.05 3.00
9.0 8.5 8.0 7.5 7.0 6.5 6.0 5.5 5.0 4.5 4.0 3.5 3.0 2.5 2.0 1.5 1.0 0.5 0.0
f1 (ppm)

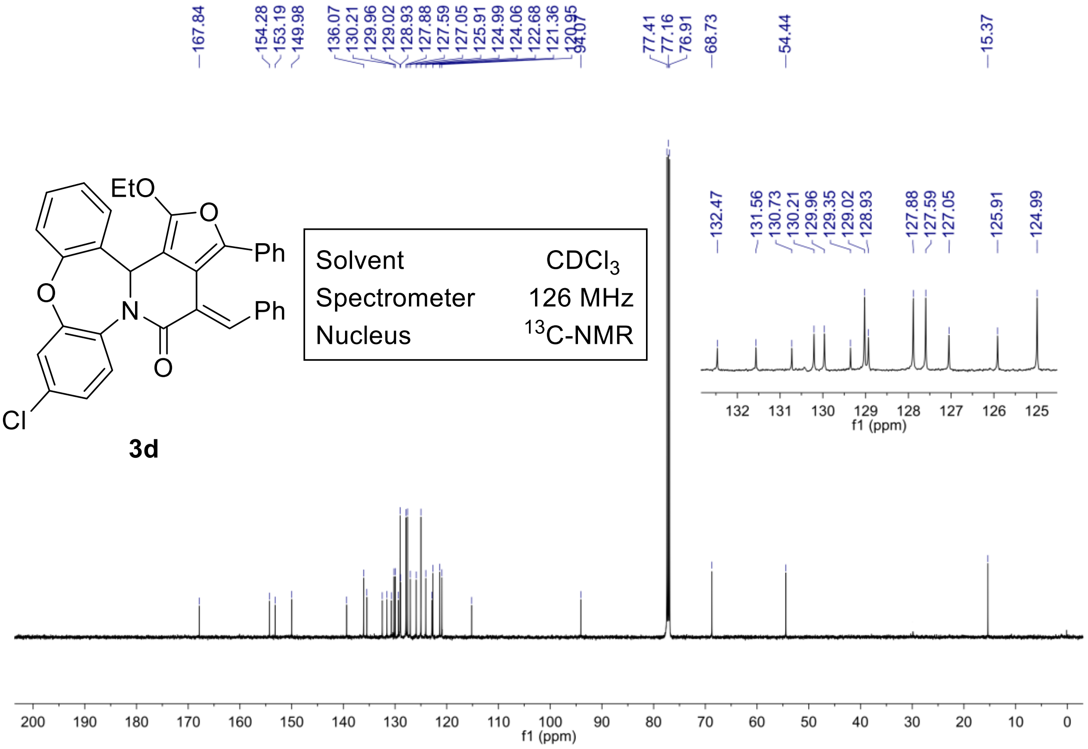
EtO
Ph
Ph
O
N
O
Cl
3d
Solvent CDCl3
Spectrometer 126 MHz
Nucleus 13C-NMR
f1 (ppm)

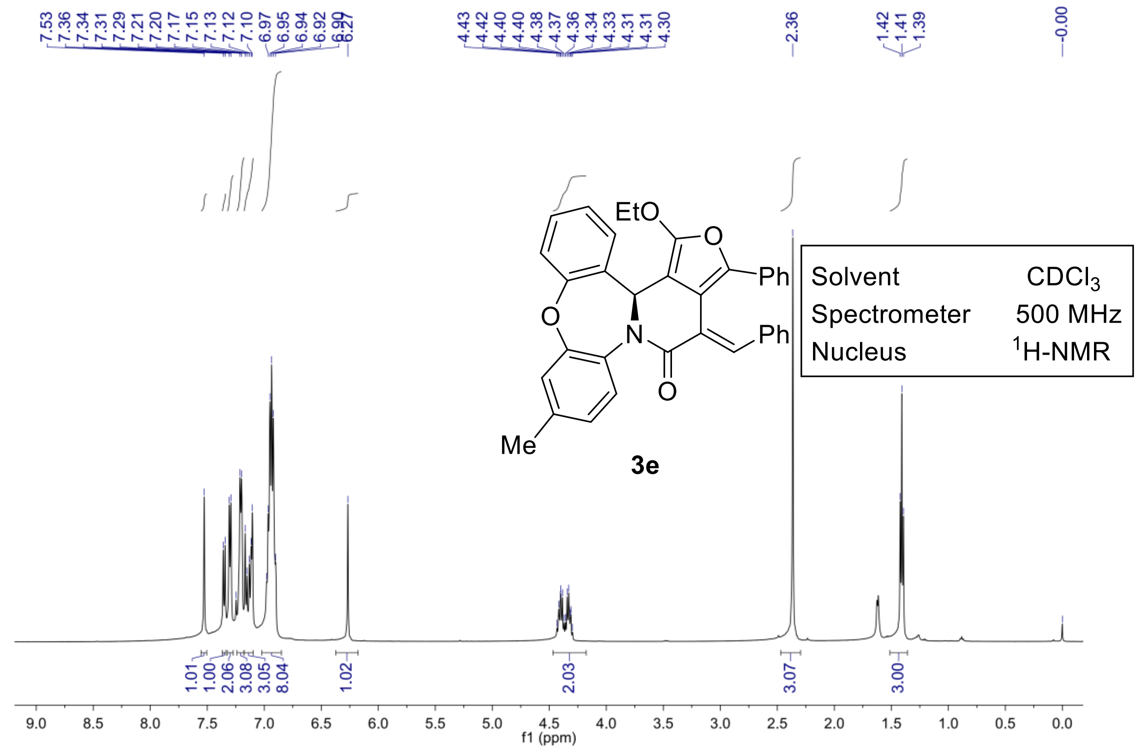
EtO
Ph
Ph
O
N
O
Me
3e
Solvent CDCl3
Spectrometer 500 MHz
Nucleus 1H-NMR
f1 (ppm)

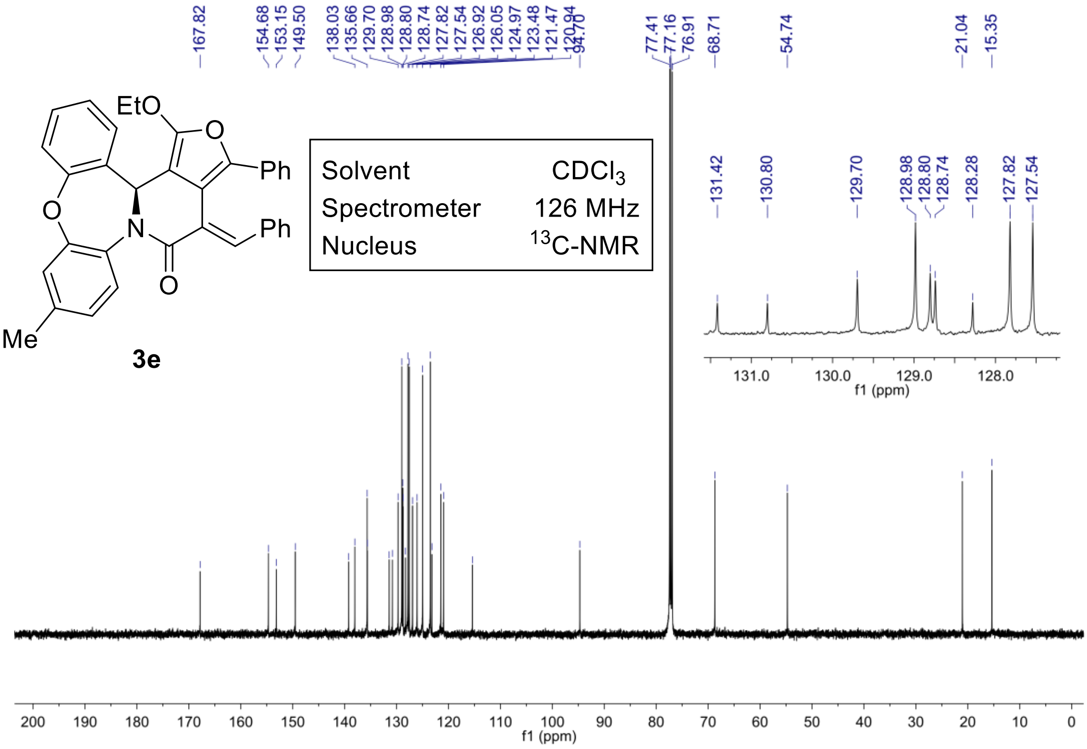
EtO
Ph
Ph
O
N
O
Me
3e
Solvent CDCl3
Spectrometer 126 MHz
Nucleus 13C-NMR
167.82
154.68
153.15
149.50
138.03
135.66
129.70
128.98
128.80
128.74
127.82
127.54
126.92
126.05
124.97
123.48
121.47
120.94
94.70
77.41
77.16
76.91
68.71
54.74
21.04
15.35
131.42
130.80
129.70
128.98
128.80
128.74
128.28
127.82
127.54
f1 (ppm)
f1 (ppm)

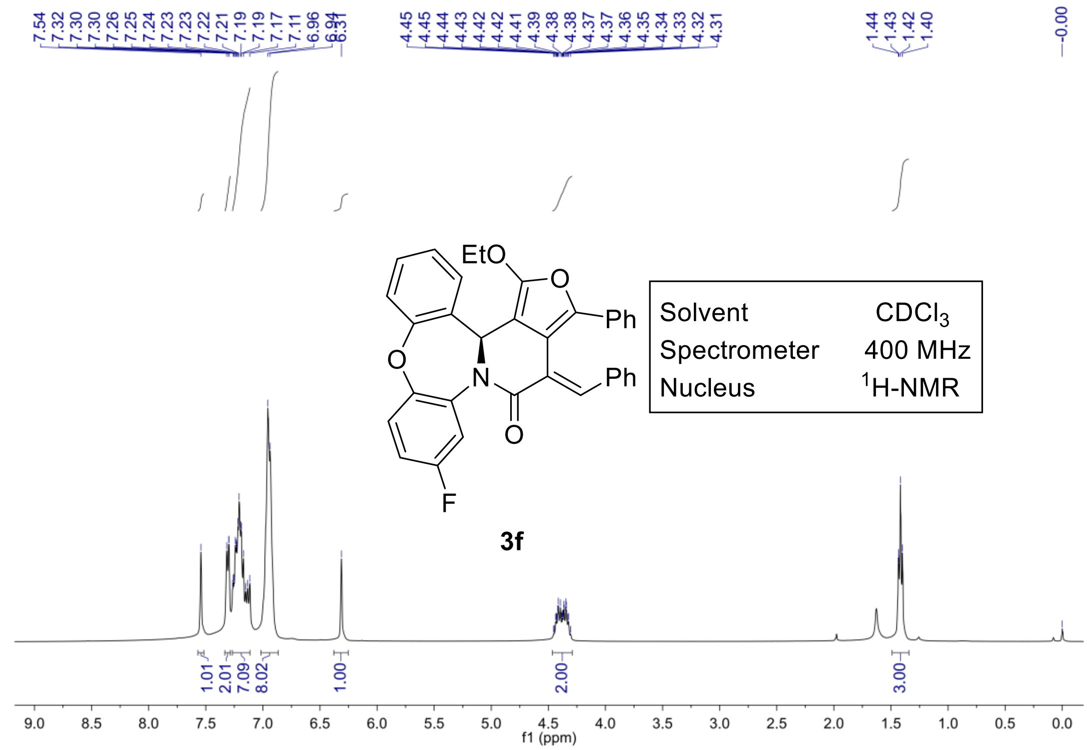
7.54
7.32
7.30
7.26
7.25
7.24
7.23
7.23
7.22
7.21
7.19
7.19
7.17
7.11
6.96
6.94
6.31
4.45
4.45
4.44
4.43
4.42
4.42
4.41
4.39
4.38
4.38
4.37
4.37
4.36
4.35
4.34
4.33
4.32
4.31
1.44
1.43
1.42
1.40
-0.00
EtO
Ph
Ph
O
N
O
F
3f
Solvent CDCl3
Spectrometer 400 MHz
Nucleus 1H-NMR
1.01
2.01
7.09
8.02
1.00
2.00
3.00
f1 (ppm)

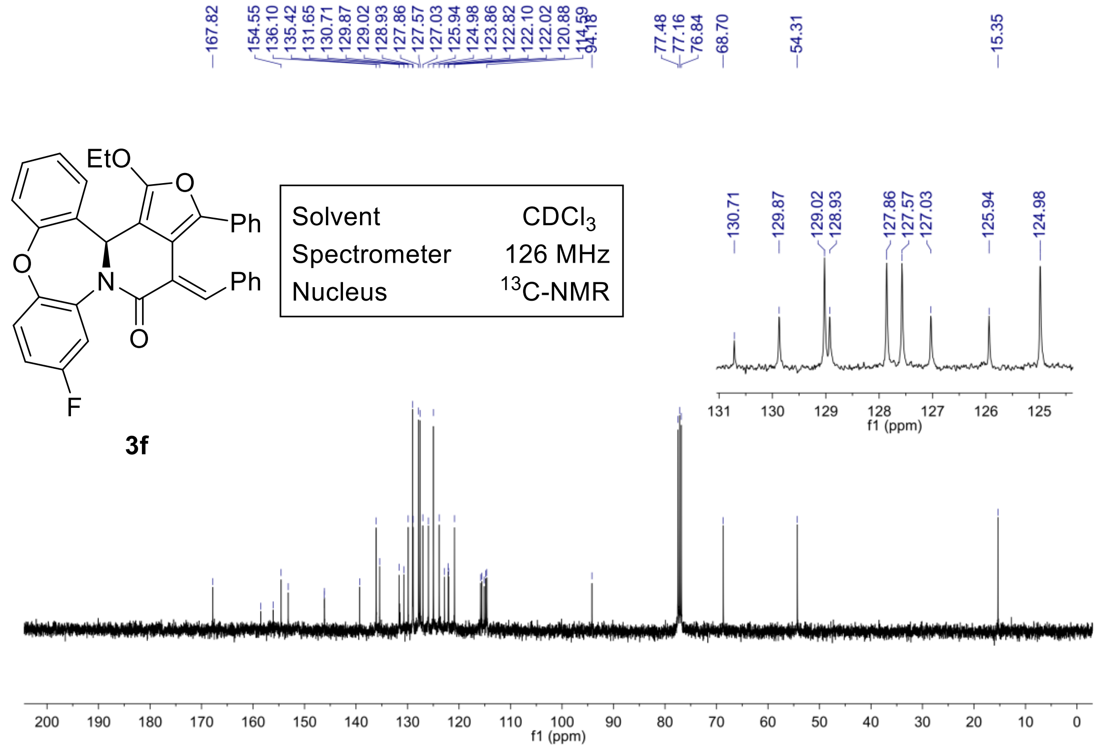
167.82
154.55
136.10
135.42
131.65
130.71
129.87
129.02
128.93
127.86
127.57
127.03
125.94
124.98
123.86
122.82
122.10
122.02
120.88
114.59
94.18
77.48
77.16
76.84
68.70
54.31
15.35
EtO
O
Ph
O
N
Ph
O
F
Solvent CDCl3
Spectrometer 126 MHz
Nucleus 13C-NMR
f1 (ppm)

3f

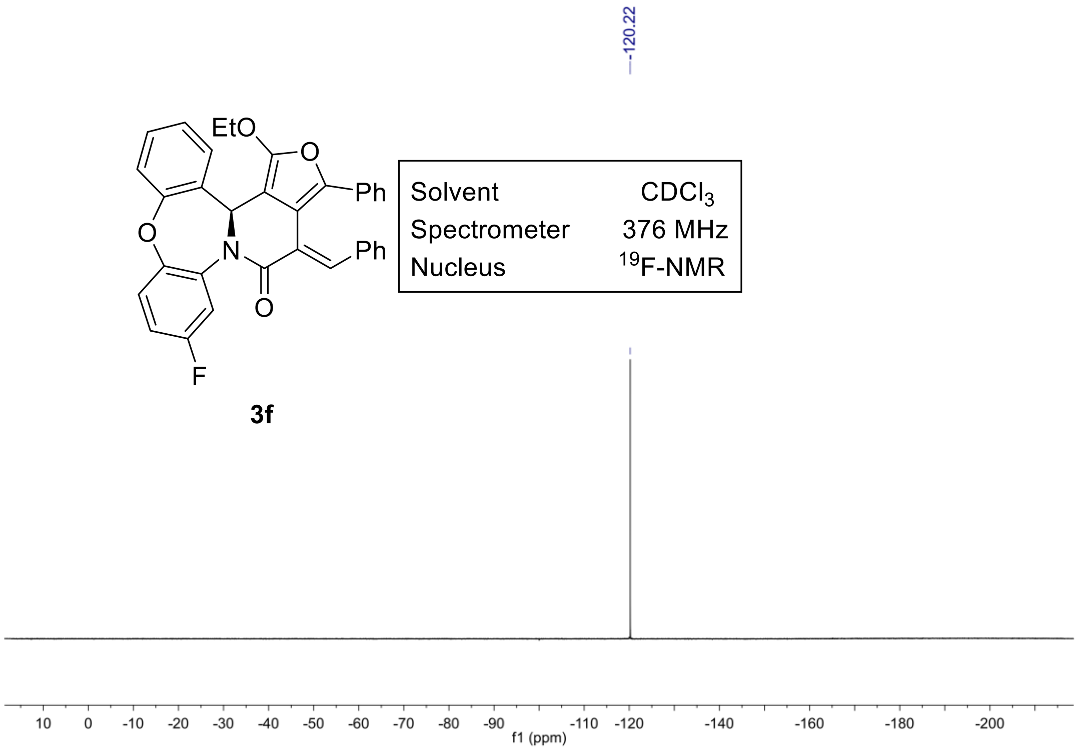
-120.22
EtO
O
Ph
O
N
Ph
O
F
Solvent CDCl3
Spectrometer 376 MHz
Nucleus 19F-NMR
f1 (ppm)

3f

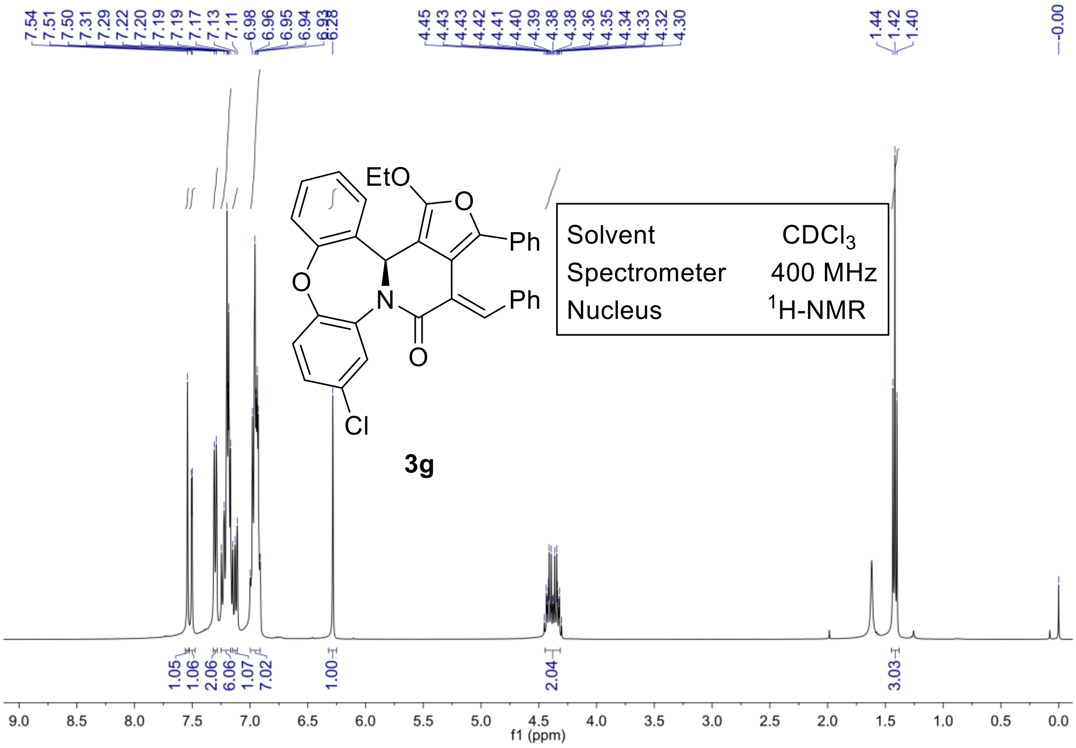

EtO
O
Ph
O
N
Ph
O
Cl
3g
Solvent CDCl3
Spectrometer 400 MHz
Nucleus 1H-NMR
f1 (ppm)

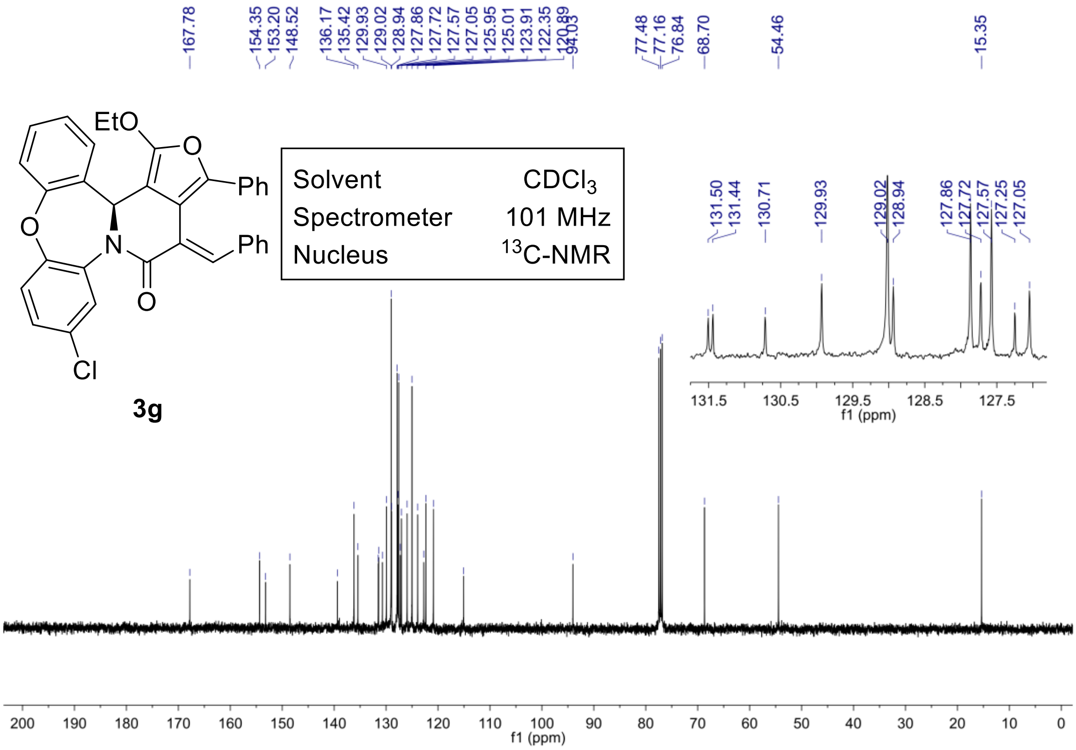

EtO
O
Ph
O
N
Ph
O
Cl
3g
Solvent CDCl3
Spectrometer 101 MHz
Nucleus 13C-NMR
f1 (ppm)

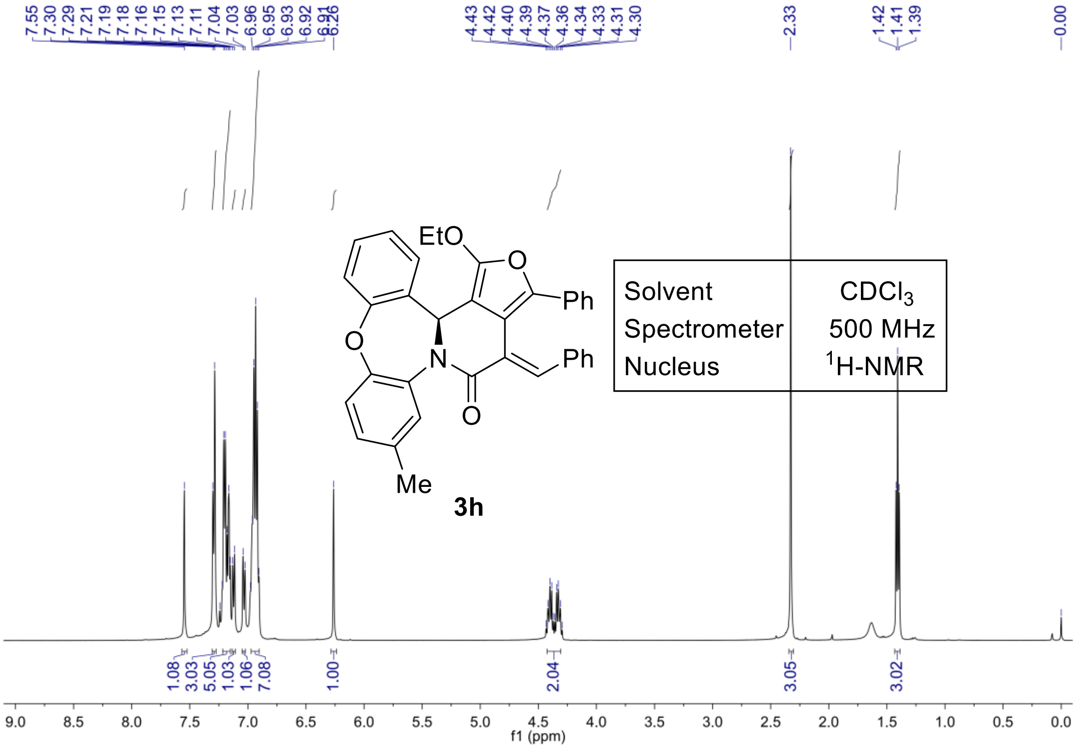

Solvent	$CDCl_3$
Spectrometer	500 MHz
Nucleus	^{1}H-NMR

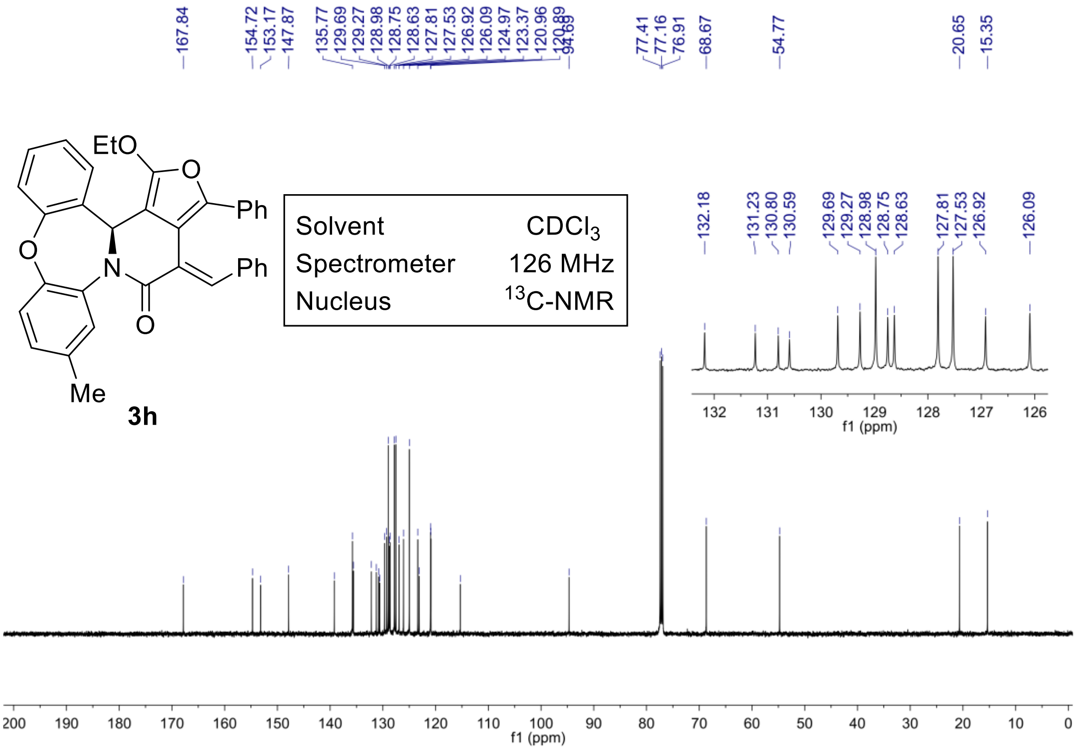

Solvent	$CDCl_3$
Spectrometer	126 MHz
Nucleus	^{13}C-NMR

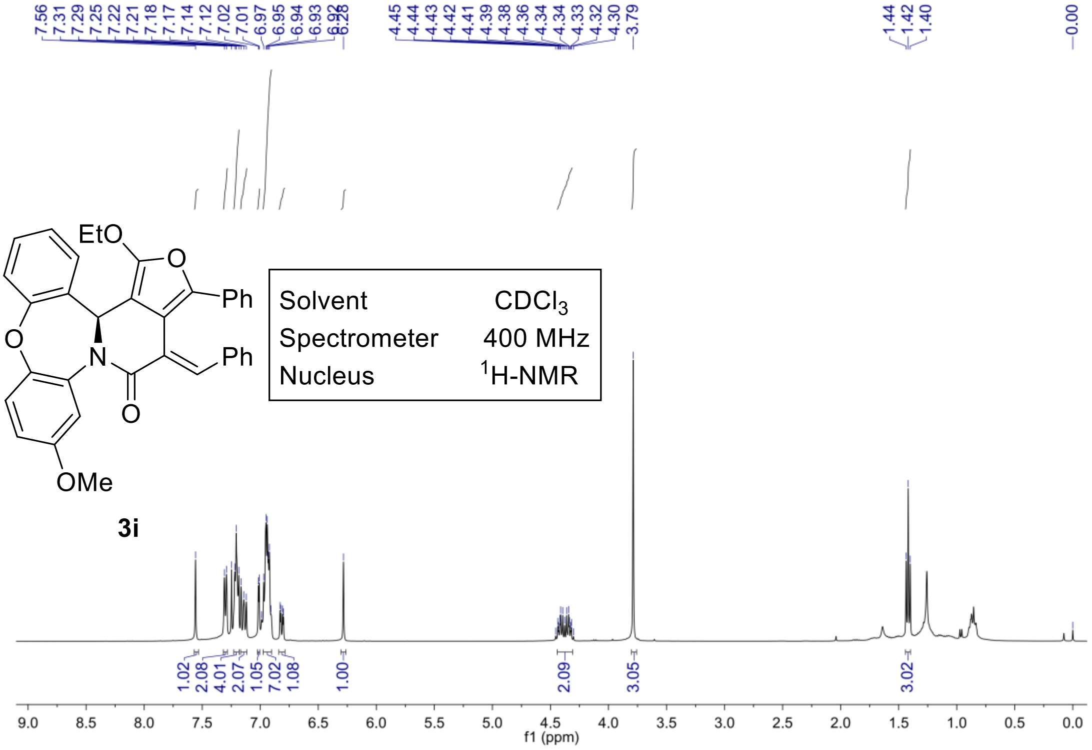

7.56 7.31 7.29 7.25 7.22 7.21 7.18 7.17 7.14 7.12 7.02 7.01 6.97 6.95 6.94 6.93 6.92 6.28
4.45 4.44 4.43 4.42 4.41 4.39 4.38 4.36 4.34 4.34 4.33 4.32 4.30 3.79
1.44 1.42 1.40
0.00
EtO
O
Ph
Ph
O
N
O
OMe
3i
Solvent CDCl$_3$
Spectrometer 400 MHz
Nucleus ^{1}H-NMR
1.02 2.08 4.01 2.07 1.05 7.02 1.08 1.00 2.09 3.05 3.02
9.0 8.5 8.0 7.5 7.0 6.5 6.0 5.5 5.0 4.5 4.0 3.5 3.0 2.5 2.0 1.5 1.0 0.5 0.0
f1 (ppm)

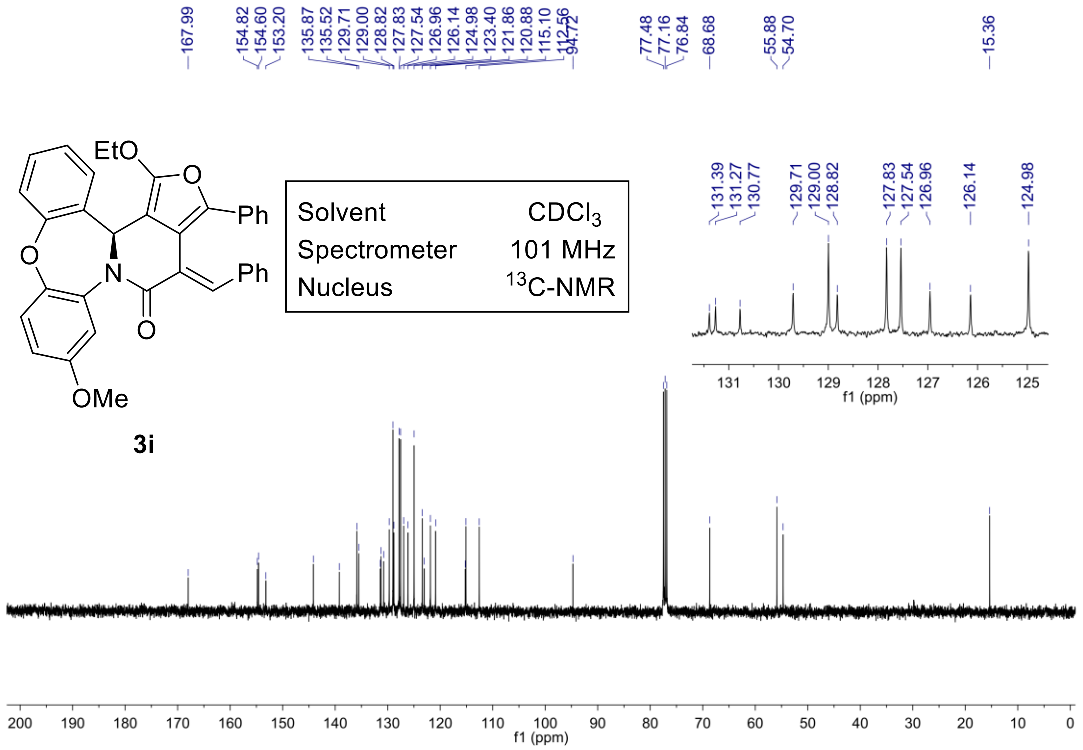

167.99
154.82 154.60 153.20
135.87 135.52 129.71 129.00 128.82 127.83 127.54 126.96 126.14 124.98 123.40 121.86 120.88 115.10 112.56 94.72
77.48 77.16 76.84 68.68
55.88 54.70
15.36
EtO
O
Ph
Ph
O
N
O
OMe
3i
Solvent CDCl$_3$
Spectrometer 101 MHz
Nucleus ^{13}C-NMR
131.39 131.27 130.77 129.71 129.00 128.82 127.83 127.54 126.96 126.14 124.98
131 130 129 128 127 126 125
f1 (ppm)
200 190 180 170 160 150 140 130 120 110 100 90 80 70 60 50 40 30 20 10 0
f1 (ppm)

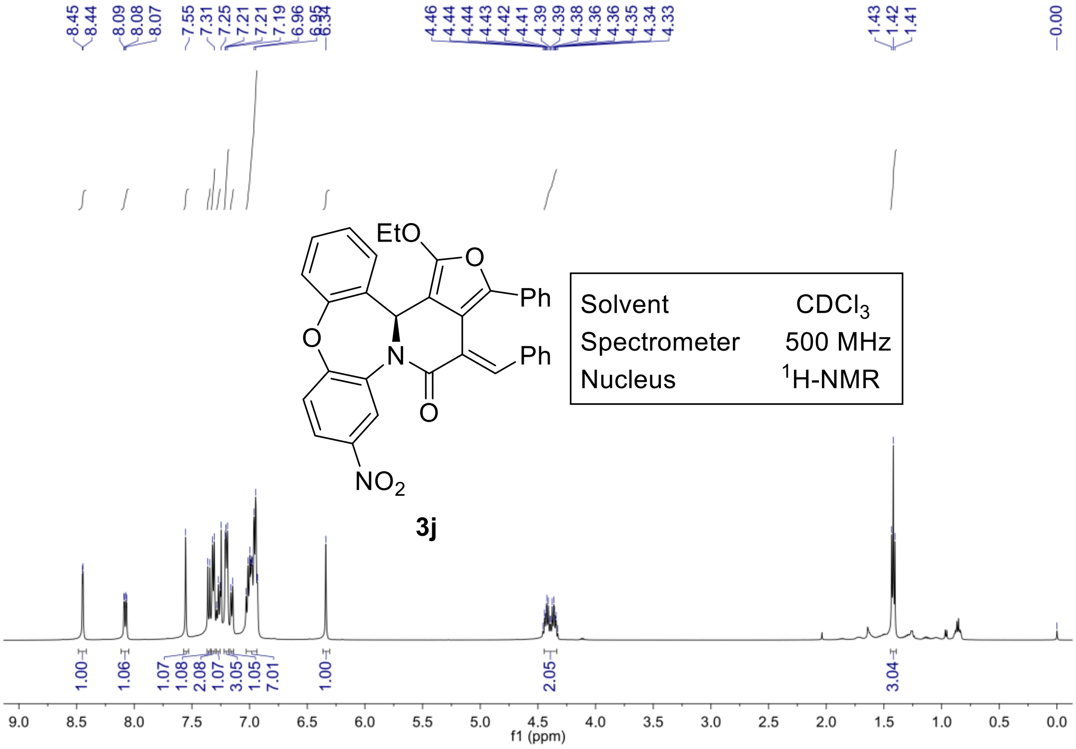
EtO
O
Ph
N
Ph
O
NO2
3j
Solvent CDCl3
Spectrometer 500 MHz
Nucleus 1H-NMR
f1 (ppm)

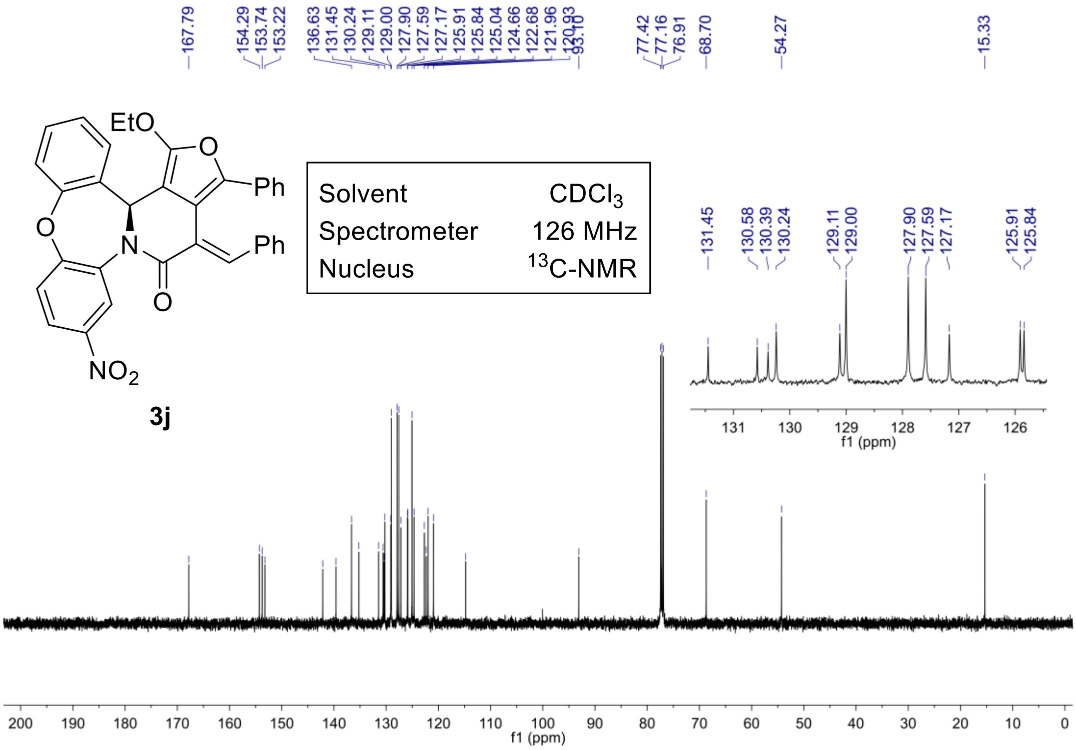
EtO
O
Ph
N
Ph
O
NO2
3j
Solvent CDCl3
Spectrometer 126 MHz
Nucleus 13C-NMR
f1 (ppm)

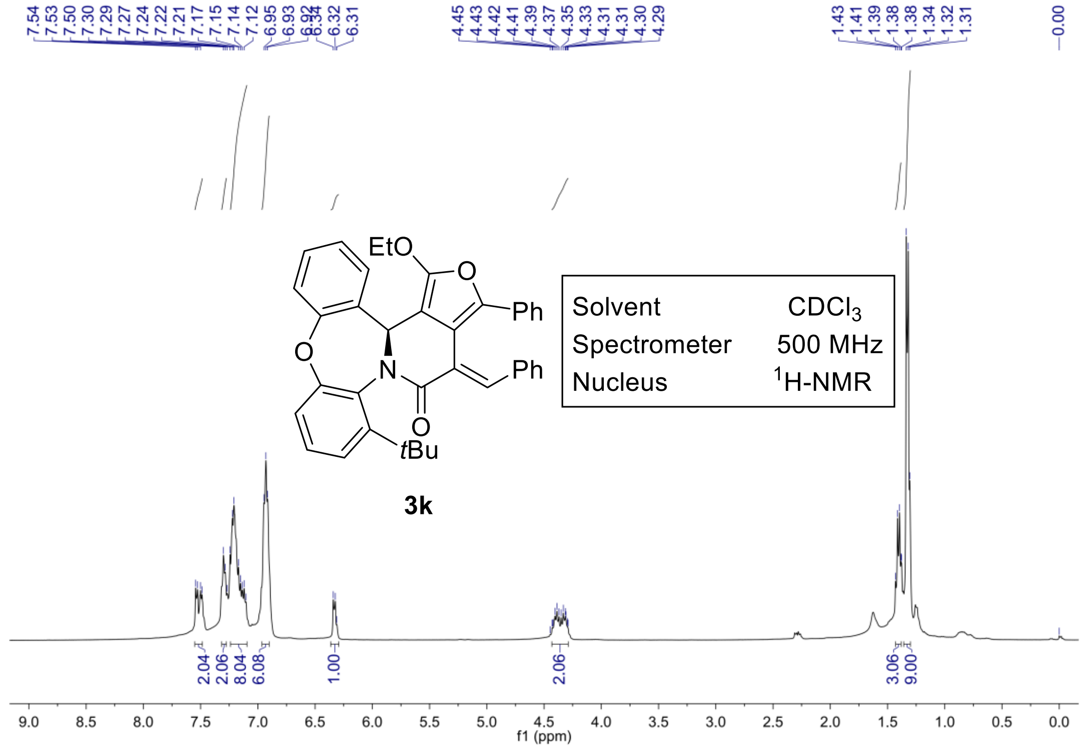
7.54 7.53 7.50 7.30 7.29 7.27 7.24 7.22 7.21 7.17 7.15 7.14 7.12 6.95 6.93 6.92 6.34 6.32 6.31
4.45 4.43 4.42 4.41 4.39 4.37 4.35 4.33 4.31 4.31 4.30 4.29
1.43 1.41 1.39 1.38 1.38 1.34 1.32 1.31
0.00
EtO
O
Ph
Ph
N
O
tBu
3k
Solvent CDCl$_3$
Spectrometer 500 MHz
Nucleus ^{1}H-NMR
2.04 2.06 8.04 6.08 1.00 2.06 3.06 9.00
f1 (ppm)

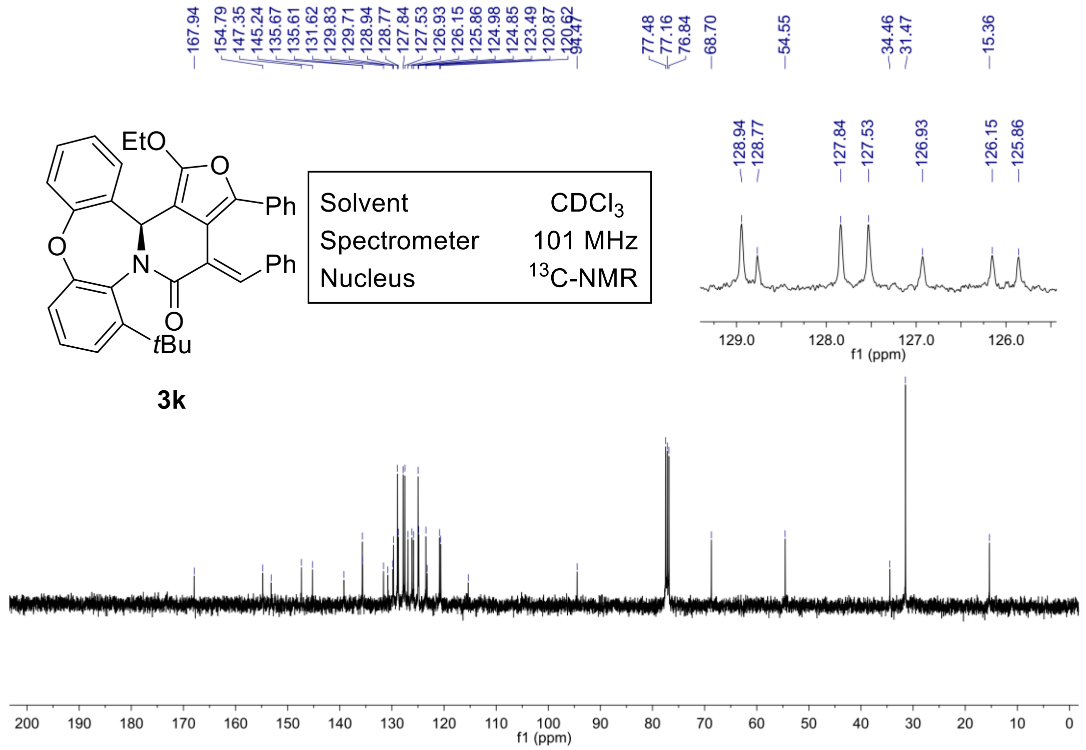
167.94 154.79 147.35 145.24 135.67 135.61 131.62 129.83 129.71 128.94 128.77 127.84 127.53 126.93 126.15 125.86 124.98 124.85 123.49 120.87 120.62 94.47
77.48 77.16 76.84 68.70 54.55 34.46 31.47 15.36
EtO
O
Ph
Ph
N
O
tBu
3k
Solvent CDCl$_3$
Spectrometer 101 MHz
Nucleus ^{13}C-NMR
128.94 128.77 127.84 127.53 126.93 126.15 125.86
f1 (ppm)

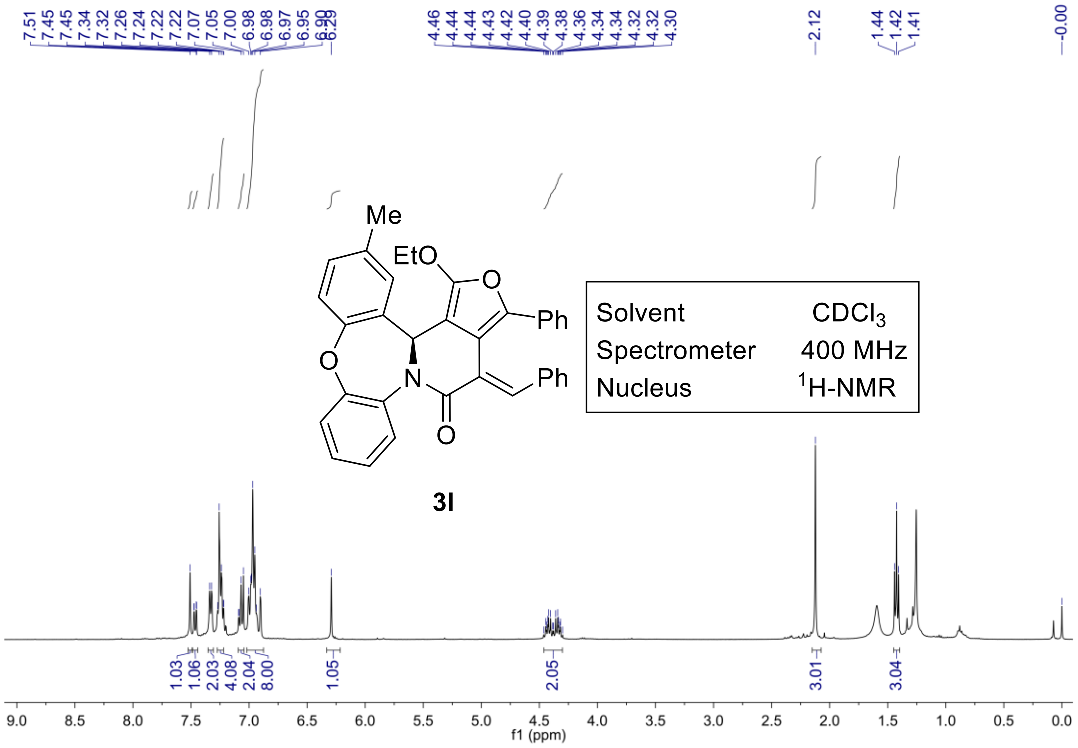

Me
EtO
O
Ph
O
N
Ph
O
3l
Solvent CDCl$_3$
Spectrometer 400 MHz
Nucleus ^{1}H-NMR
f1 (ppm)

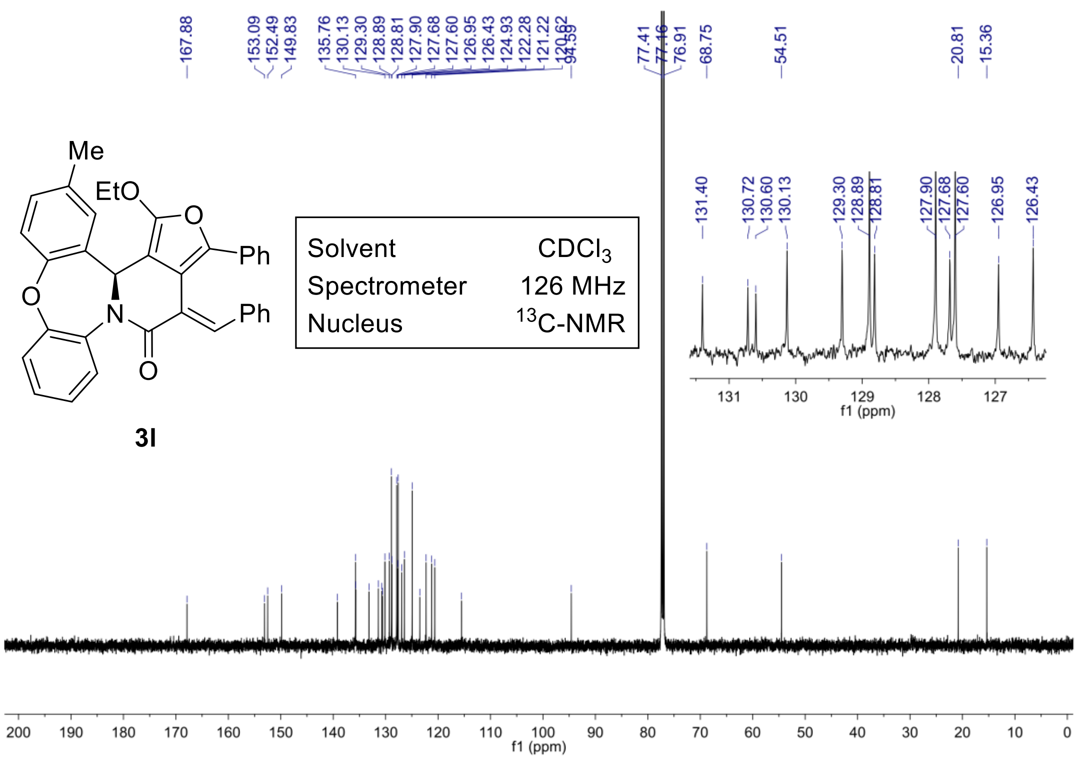

Me
EtO
O
Ph
O
N
Ph
O
3l
Solvent CDCl$_3$
Spectrometer 126 MHz
Nucleus ^{13}C-NMR
f1 (ppm)

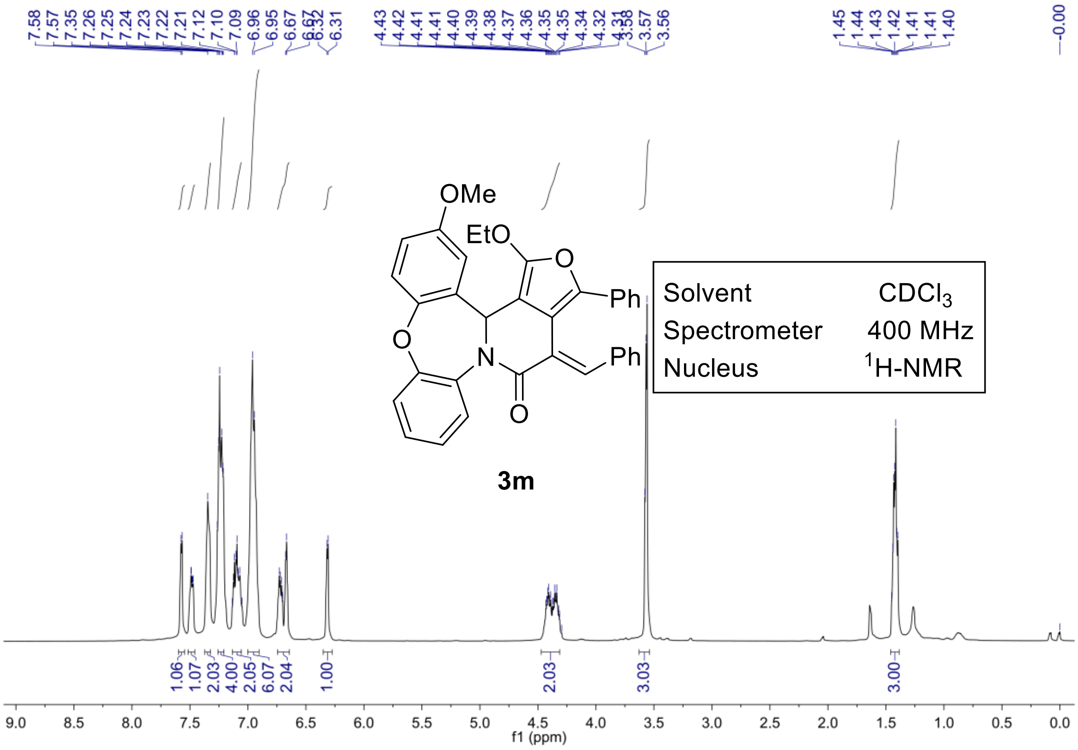
OMe
EtO
O
Ph
Ph
O
N
O
3m
Solvent CDCl3
Spectrometer 400 MHz
Nucleus 1H-NMR
f1 (ppm)

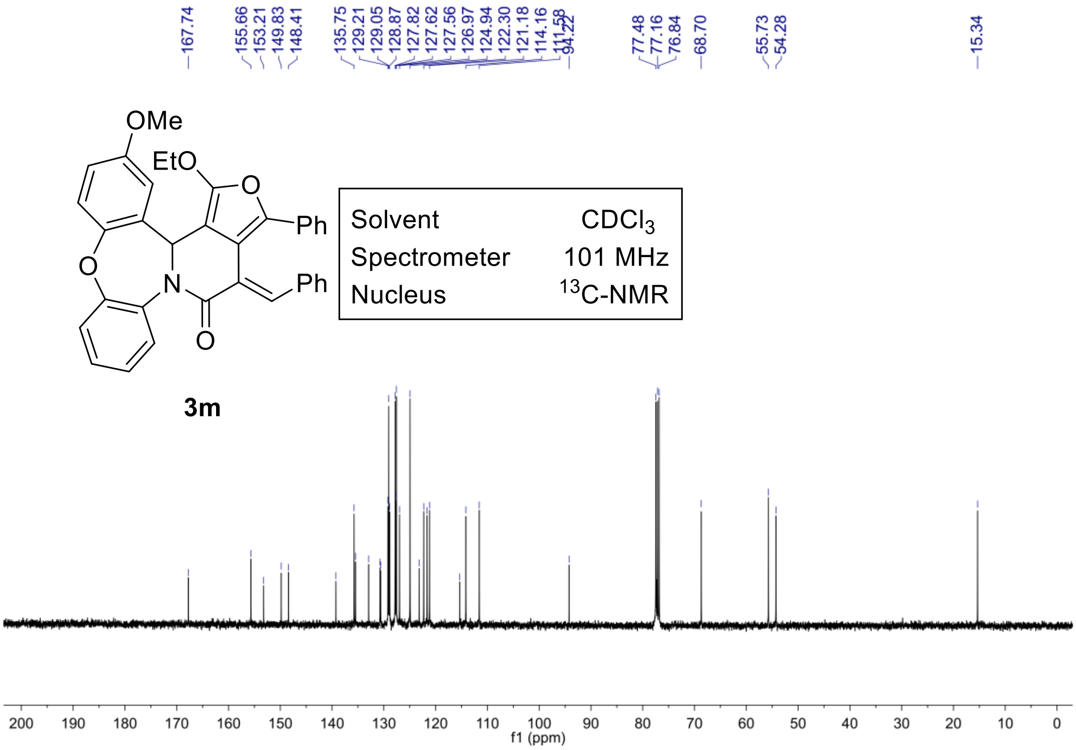
OMe
EtO
O
Ph
Ph
O
N
O
3m
Solvent CDCl3
Spectrometer 101 MHz
Nucleus 13C-NMR
f1 (ppm)

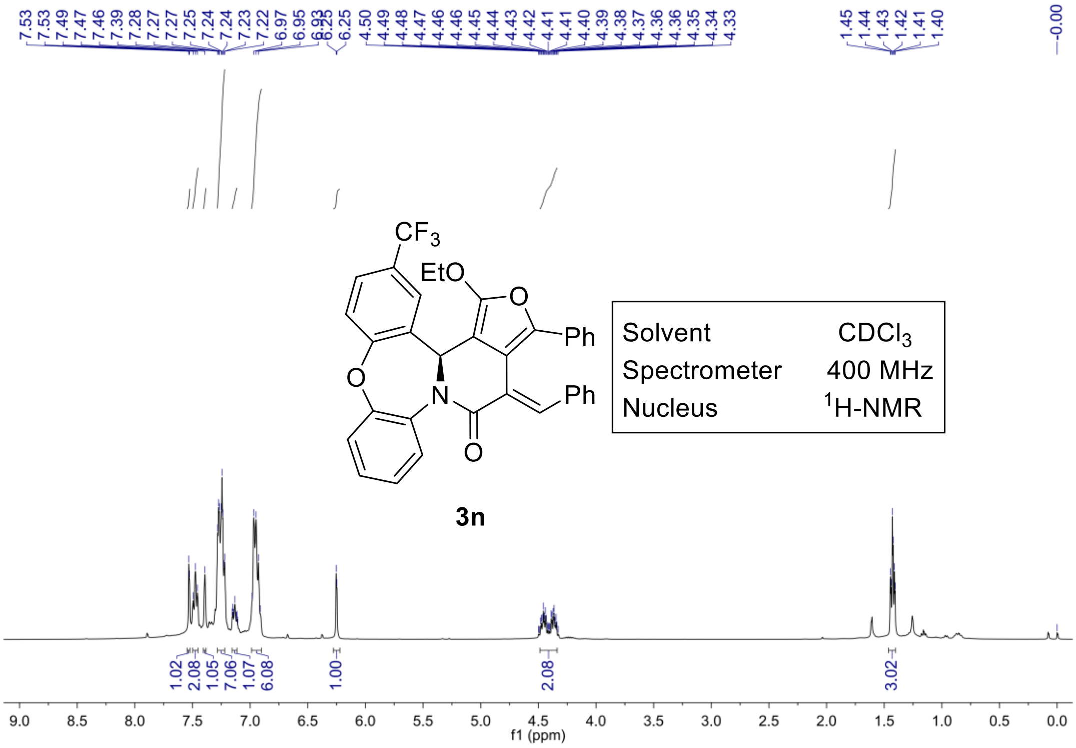
7.53 7.53 7.49 7.47 7.46 7.39 7.28 7.27 7.27 7.25 7.24 7.24 7.23 7.22 6.97 6.95 6.93 6.25 6.25 4.50 4.49 4.48 4.47 4.46 4.46 4.45 4.44 4.43 4.42 4.41 4.41 4.40 4.39 4.38 4.37 4.36 4.36 4.35 4.34 4.33 1.45 1.44 1.43 1.42 1.41 1.40 -0.00
CF3
EtO
O
Ph
N
Ph
O
O
Solvent CDCl3
Spectrometer 400 MHz
Nucleus 1H-NMR
3n
1.02 2.08 1.05 7.06 1.07 6.08 1.00 2.08 3.02
f1 (ppm)

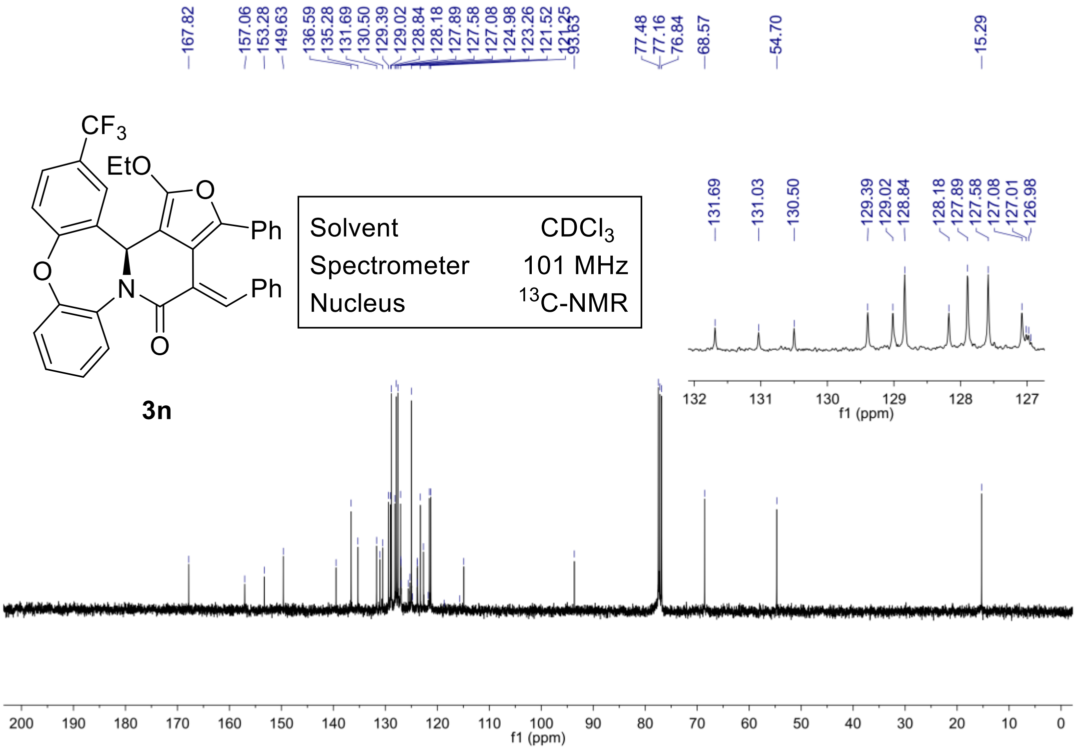
167.82 157.06 153.28 149.63 136.59 135.28 131.69 130.50 129.39 129.02 128.84 128.18 127.89 127.58 127.08 124.98 123.26 121.52 121.25 93.63 77.48 77.16 76.84 68.57 54.70 15.29
CF3
EtO
O
Ph
N
Ph
O
O
Solvent CDCl3
Spectrometer 101 MHz
Nucleus 13C-NMR
3n
131.69 131.03 130.50 129.39 129.02 128.84 128.18 127.89 127.58 127.08 127.01 126.98
f1 (ppm)

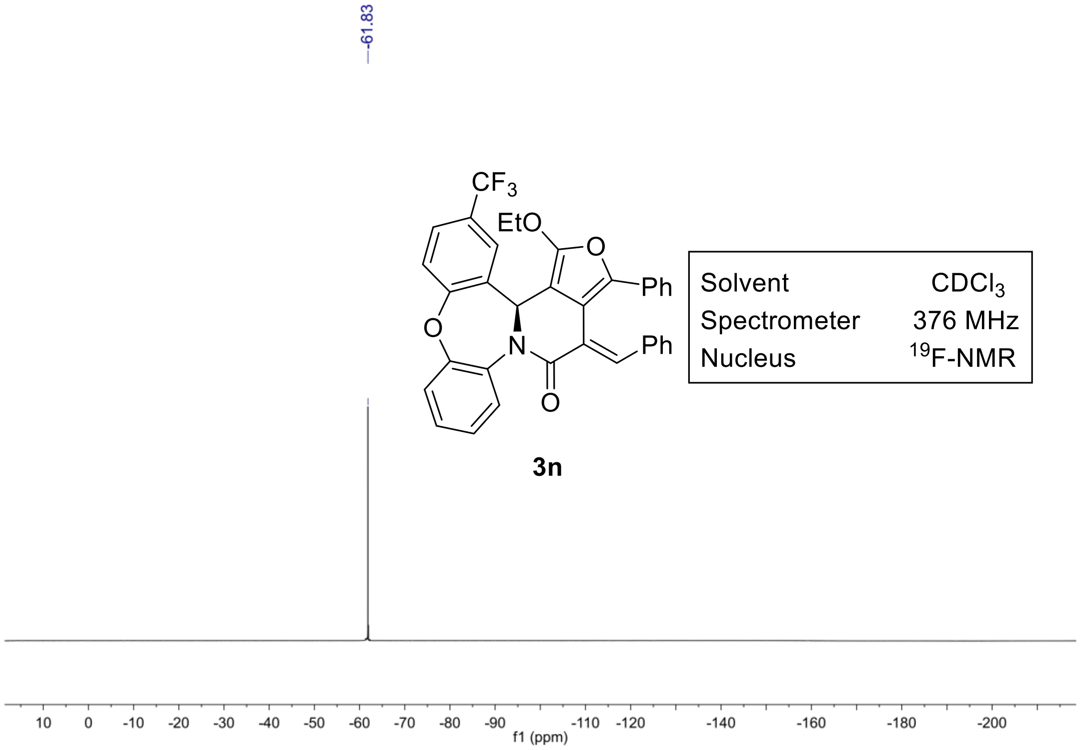

-61.83
CF3
EtO
O
Ph
Ph
N
O
3n
Solvent CDCl3
Spectrometer 376 MHz
Nucleus 19F-NMR
f1 (ppm)

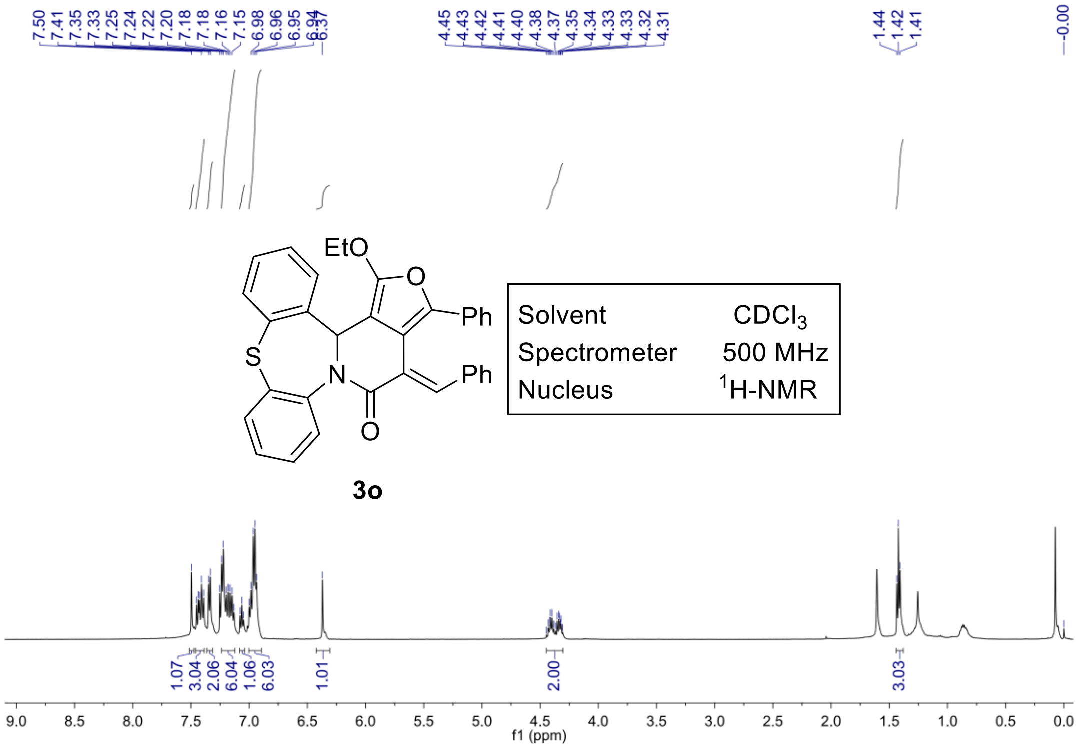

EtO
O
Ph
S
N
Ph
O
3o
Solvent CDCl3
Spectrometer 500 MHz
Nucleus 1H-NMR
f1 (ppm)

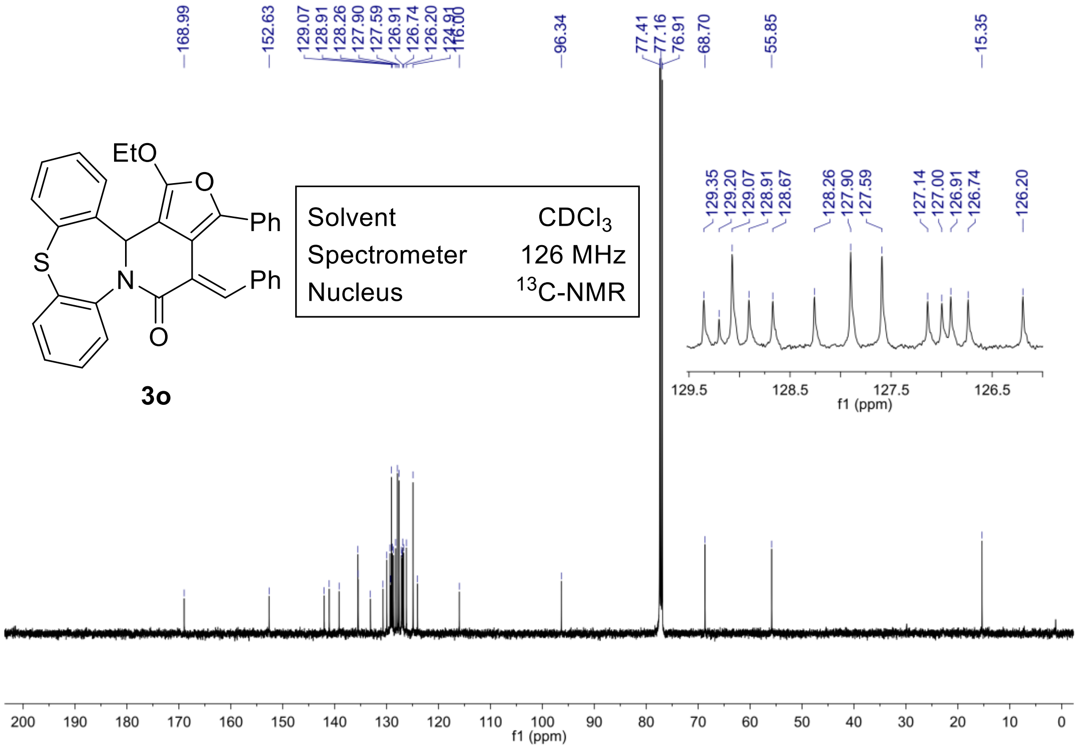
EtO
O
Ph
S
N
Ph
O
3o
Solvent CDCl3
Spectrometer 126 MHz
Nucleus 13C-NMR
168.99
152.63
129.07
128.91
128.26
127.90
127.59
126.91
126.74
126.20
124.91
116.00
96.34
77.41
77.16
76.91
68.70
55.85
15.35
129.35
129.20
129.07
128.91
128.67
128.26
127.90
127.59
127.14
127.00
126.91
126.74
126.20
129.5
128.5
127.5
126.5
f1 (ppm)
200
190
180
170
160
150
140
130
120
110
100
90
80
70
60
50
40
30
20
10
0
f1 (ppm)

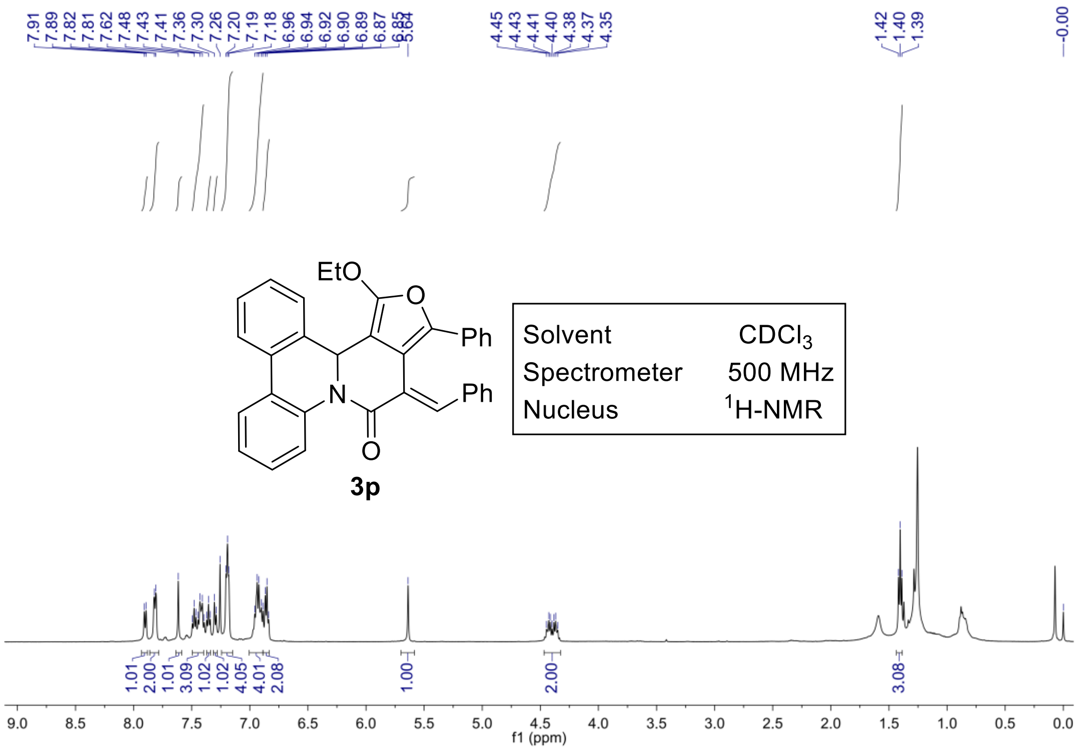
7.91
7.89
7.82
7.81
7.62
7.48
7.43
7.41
7.36
7.30
7.26
7.20
7.19
7.18
6.96
6.94
6.92
6.90
6.89
6.87
6.85
5.64
4.45
4.43
4.41
4.40
4.38
4.37
4.35
1.42
1.40
1.39
-0.00
EtO
O
Ph
N
Ph
O
3p
Solvent CDCl3
Spectrometer 500 MHz
Nucleus 1H-NMR
1.01
2.00
1.01
3.09
1.02
1.02
4.05
4.01
2.08
1.00
2.00
3.08
9.0
8.5
8.0
7.5
7.0
6.5
6.0
5.5
5.0
4.5
4.0
3.5
3.0
2.5
2.0
1.5
1.0
0.5
0.0
f1 (ppm)

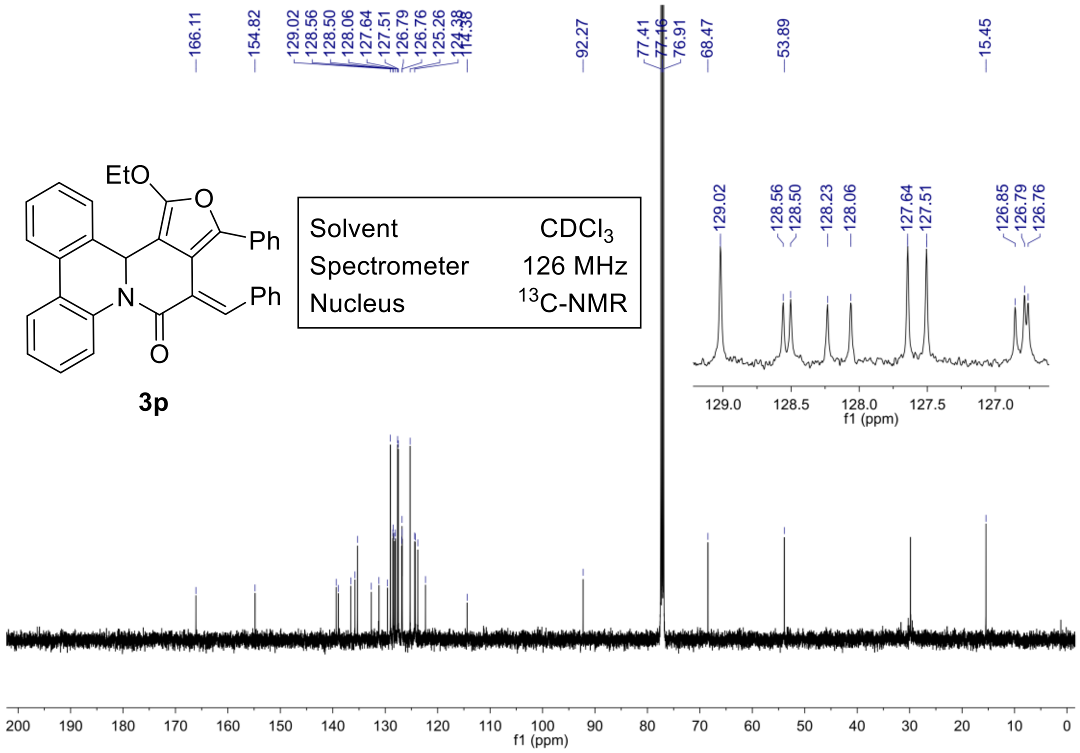
166.11
154.82
129.02
128.56
128.50
128.06
127.64
127.51
126.79
126.76
125.26
124.38
114.38
92.27
77.41
77.16
76.91
68.47
53.89
15.45
EtO
O
Ph
N
Ph
O
3p
Solvent CDCl3
Spectrometer 126 MHz
Nucleus 13C-NMR
129.02
128.56
128.50
128.23
128.06
127.64
127.51
126.85
126.79
126.76
f1 (ppm)
f1 (ppm)

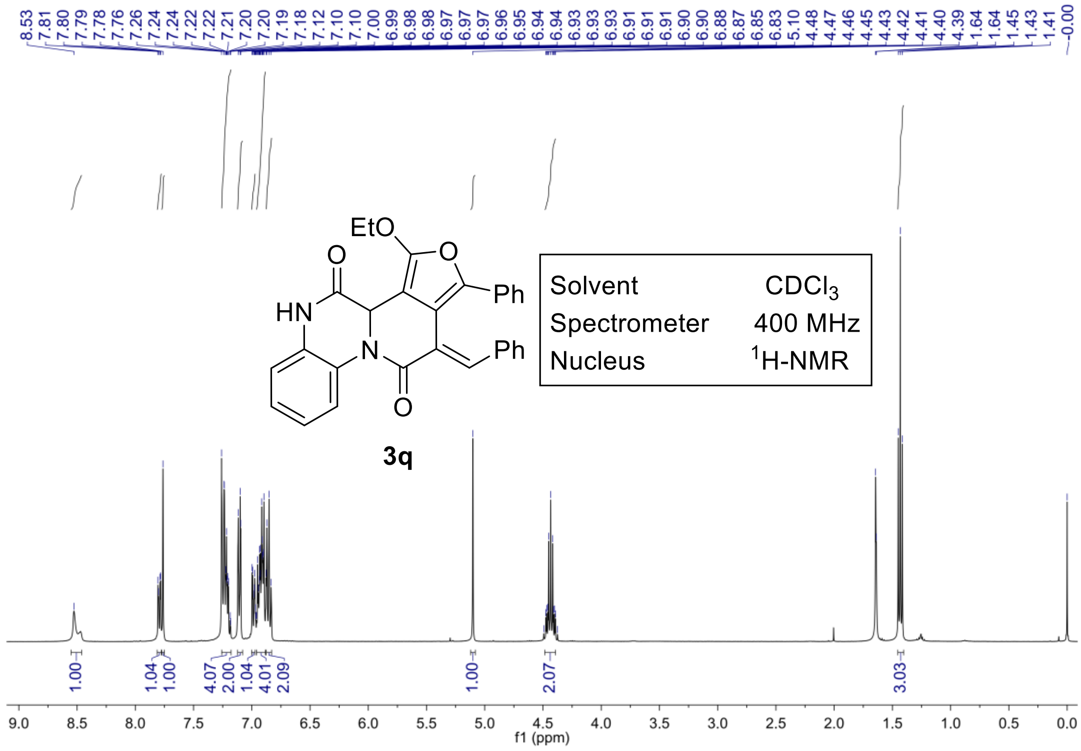
8.53
7.81
7.80
7.79
7.76
7.26
7.24
7.24
7.22
7.22
7.21
7.20
7.20
7.19
7.18
7.12
7.10
7.10
7.00
6.99
6.98
6.98
6.97
6.97
6.96
6.95
6.94
6.94
6.93
6.93
6.93
6.91
6.91
6.91
6.90
6.90
6.88
6.87
6.85
6.83
5.10
4.48
4.47
4.46
4.45
4.43
4.42
4.41
4.40
4.39
1.64
1.64
1.45
1.43
1.41
-0.00
EtO
O
O
HN
Ph
N
Ph
O
3q
Solvent CDCl3
Spectrometer 400 MHz
Nucleus 1H-NMR
1.00
1.04
1.00
4.07
2.00
1.04
4.01
2.09
1.00
2.07
3.03
f1 (ppm)

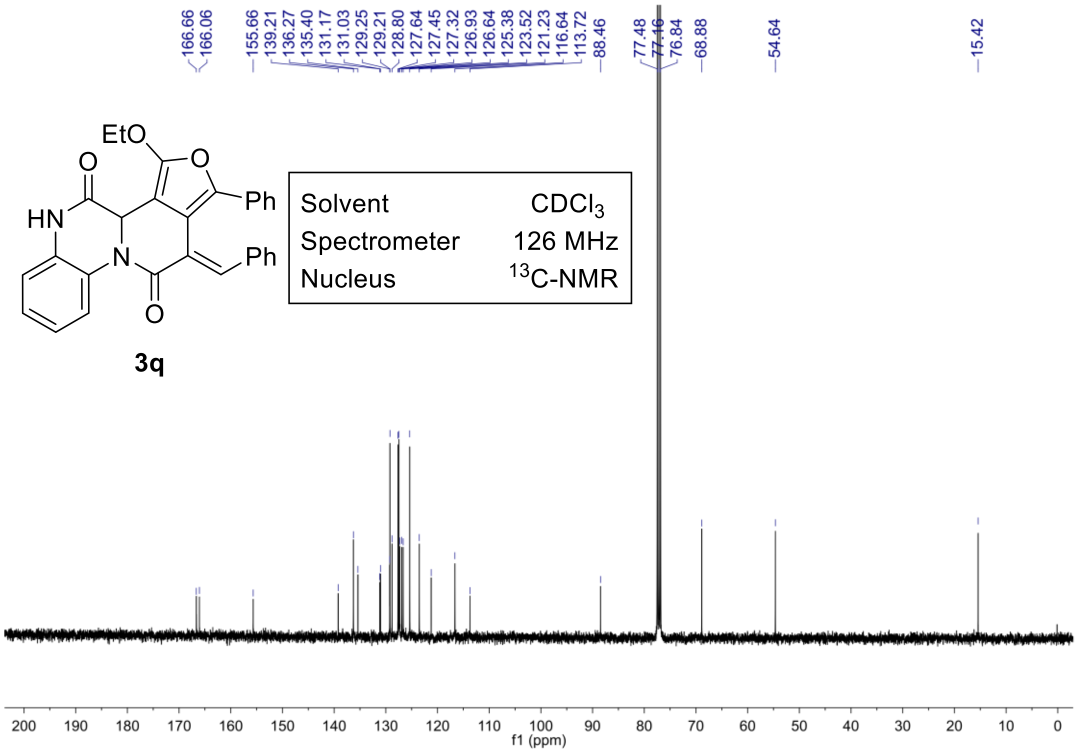
EtO
HN
N
O
Ph
Ph
3q
Solvent CDCl3
Spectrometer 126 MHz
Nucleus 13C-NMR
166.66
166.06
155.66
139.21
136.27
135.40
131.17
131.03
129.25
129.21
128.80
127.64
127.45
127.32
126.93
126.64
125.38
123.52
121.23
116.64
113.72
88.46
77.48
77.16
76.84
68.88
54.64
15.42
f1 (ppm)

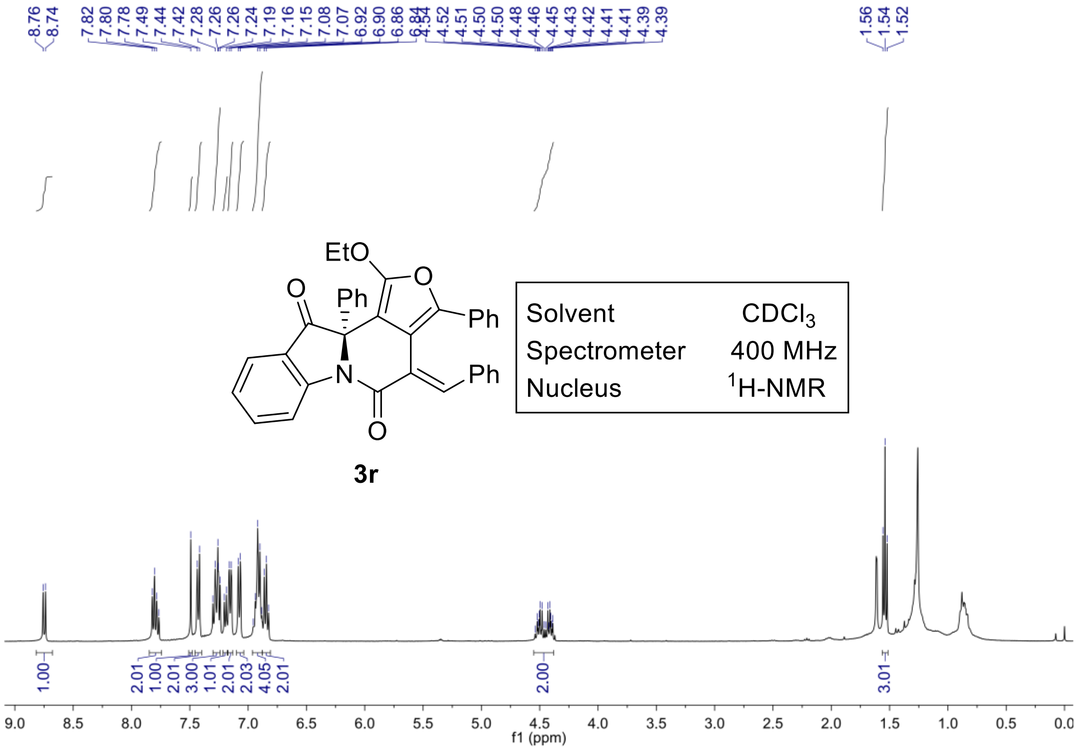
EtO
Ph
N
O
Ph
Ph
3r
Solvent CDCl3
Spectrometer 400 MHz
Nucleus 1H-NMR
8.76
8.74
7.82
7.80
7.78
7.49
7.44
7.42
7.28
7.26
7.26
7.24
7.19
7.16
7.15
7.08
7.07
6.92
6.90
6.86
6.84
4.54
4.52
4.51
4.50
4.50
4.48
4.46
4.45
4.43
4.42
4.41
4.41
4.39
4.39
1.56
1.54
1.52
1.00
2.01
1.00
2.01
3.00
1.01
2.01
2.03
4.05
2.01
2.00
3.01
f1 (ppm)

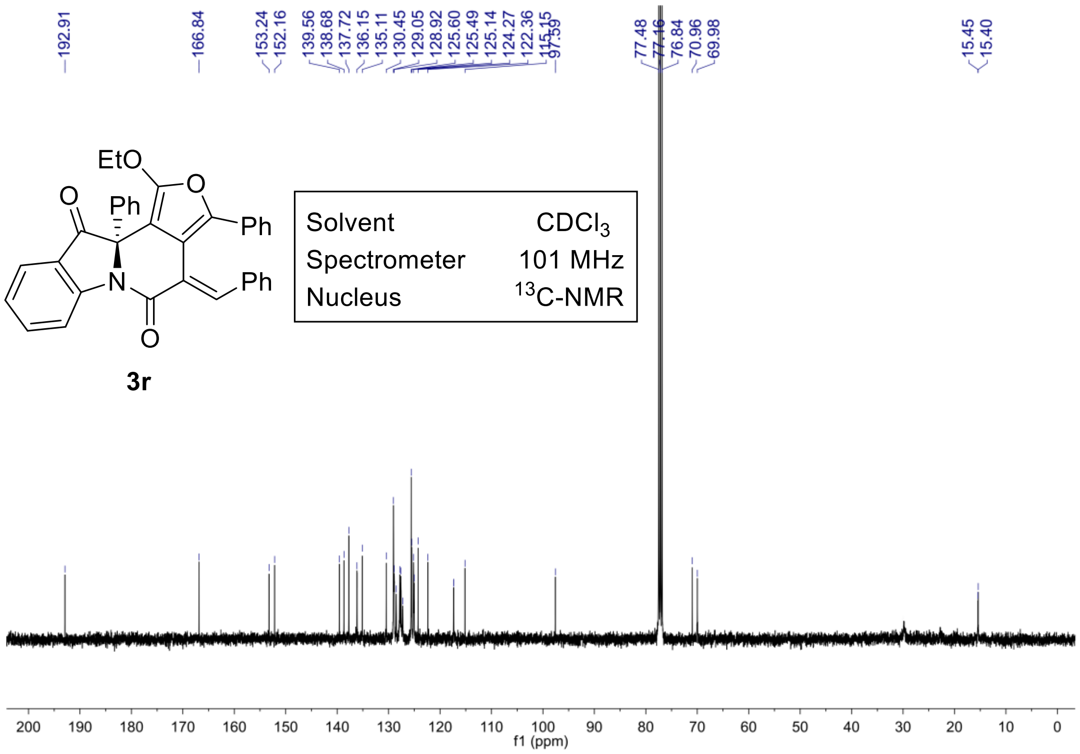
EtO
O
Ph
Ph
N
Ph
O
3r
Solvent CDCl3
Spectrometer 101 MHz
Nucleus 13C-NMR
f1 (ppm)

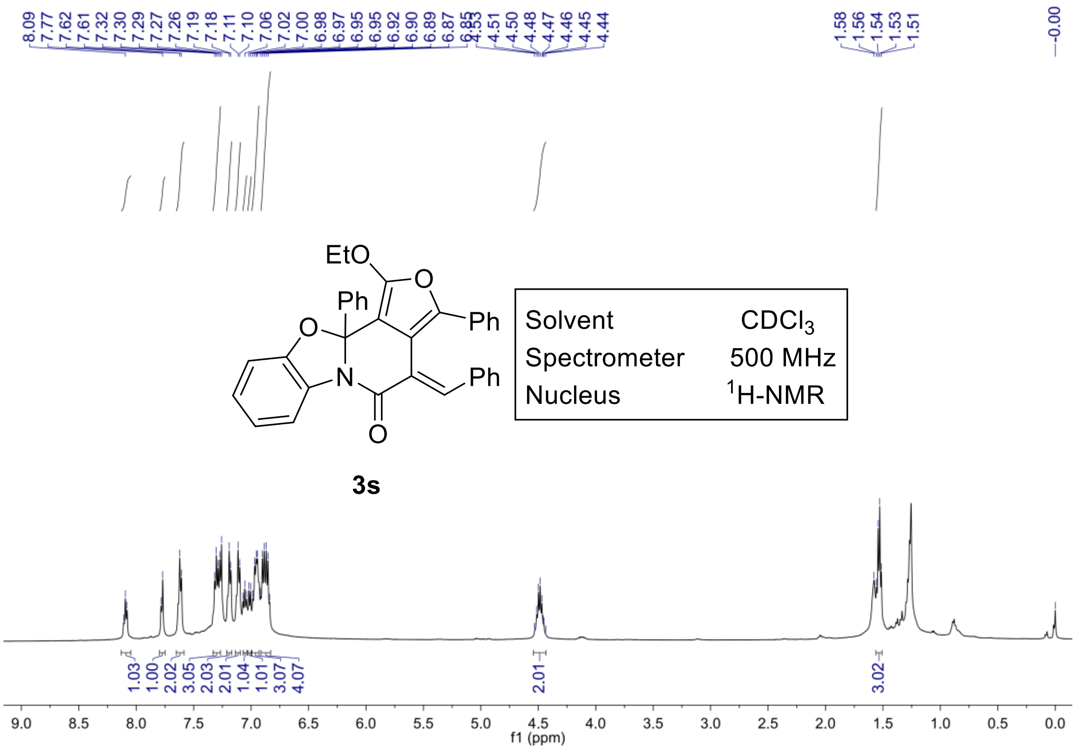
EtO
O
Ph
O
Ph
N
Ph
O
3s
Solvent CDCl3
Spectrometer 500 MHz
Nucleus 1H-NMR
f1 (ppm)

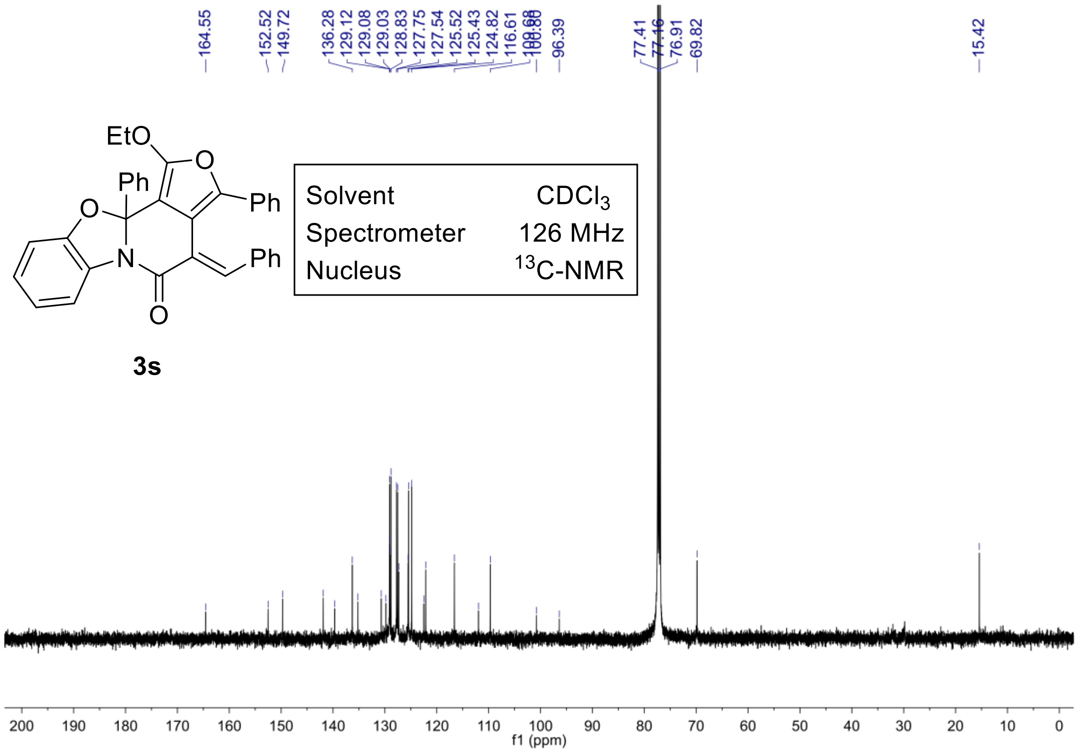
EtO
O
Ph
Ph
O
N
Ph
O
3s
Solvent CDCl3
Spectrometer 126 MHz
Nucleus 13C-NMR
164.55
152.52
149.72
136.28
129.12
129.08
129.03
128.83
127.75
127.54
125.52
125.43
124.82
116.61
96.39
77.41
77.16
76.91
69.82
15.42
f1 (ppm)

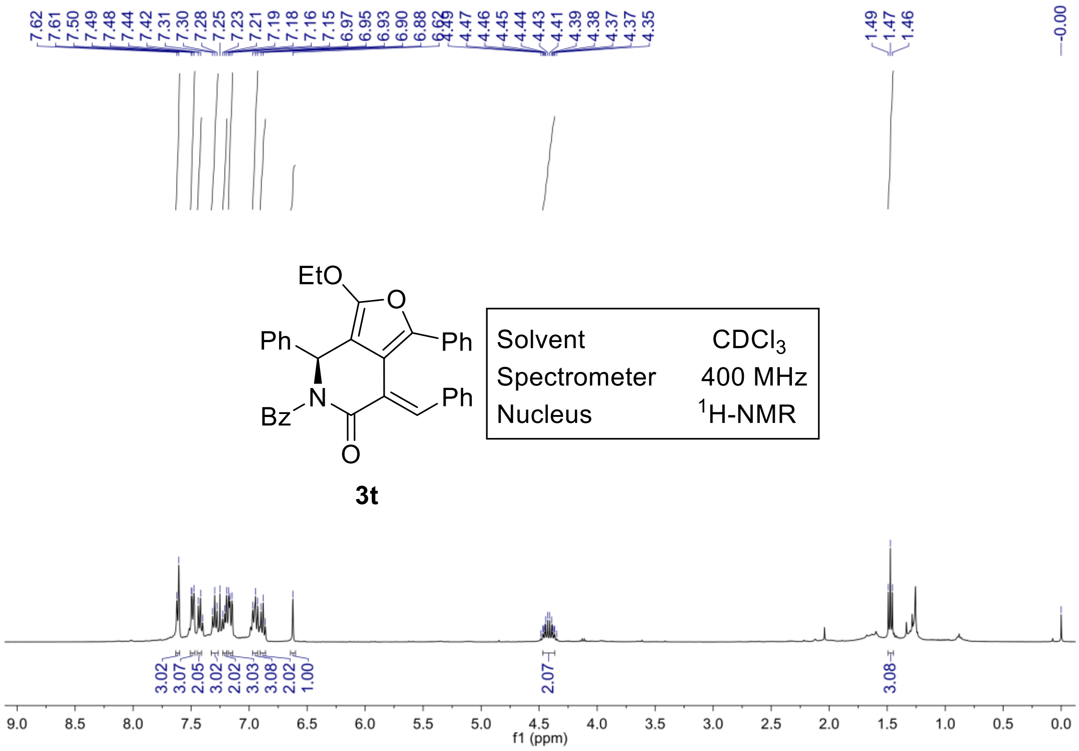
EtO
O
Ph
Ph
N
Bz
Ph
O
3t
Solvent CDCl3
Spectrometer 400 MHz
Nucleus 1H-NMR
7.62
7.61
7.50
7.49
7.48
7.44
7.42
7.31
7.30
7.28
7.25
7.23
7.21
7.19
7.18
7.16
7.15
6.97
6.95
6.93
6.90
6.88
4.47
4.46
4.45
4.44
4.43
4.41
4.39
4.38
4.37
4.35
1.49
1.47
1.46
-0.00
3.02
3.07
2.05
3.02
2.02
3.03
3.08
2.02
1.00
2.07
3.08
f1 (ppm)

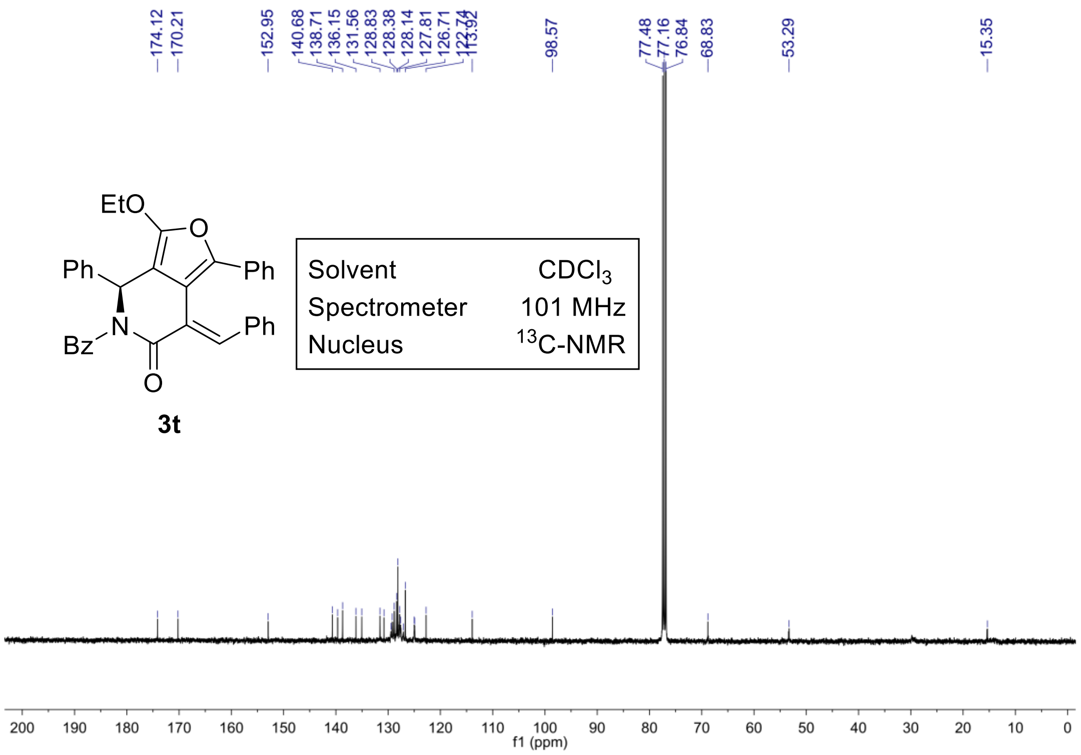

174.12
170.21
152.95
140.68
138.71
136.15
131.56
128.83
128.38
128.14
127.81
126.71
122.74
113.92
98.57
77.48
77.16
76.84
68.83
53.29
15.35
EtO
O
Ph
Ph
N
Bz
Ph
O
3t
Solvent CDCl3
Spectrometer 101 MHz
Nucleus 13C-NMR
f1 (ppm)

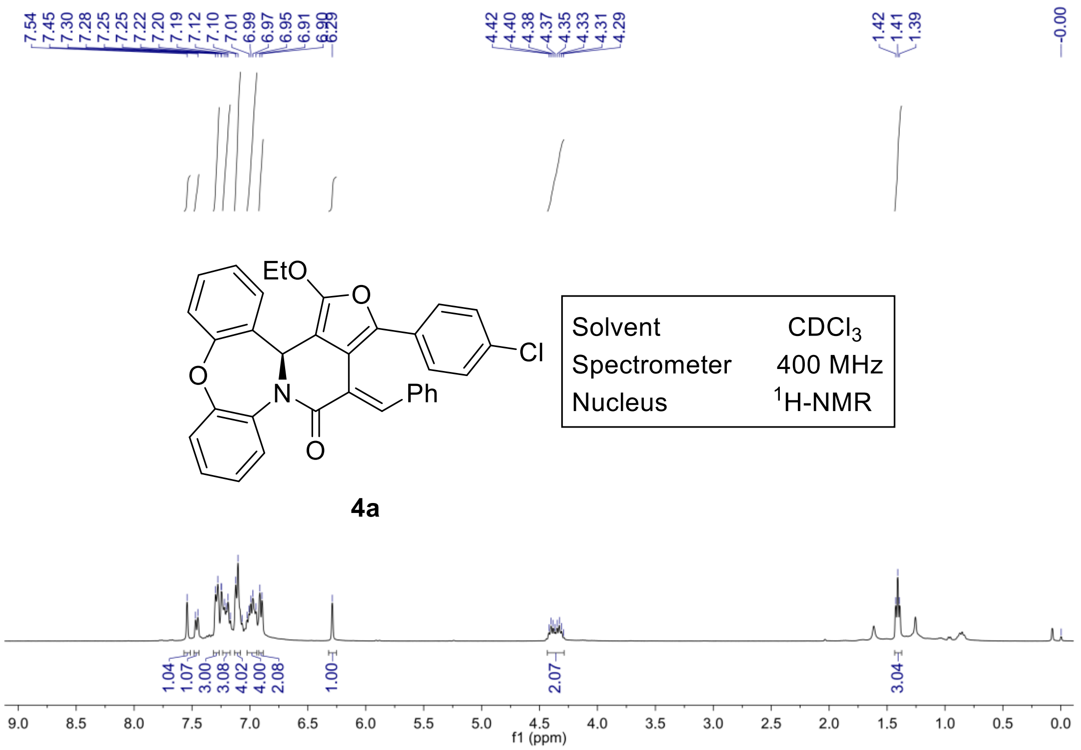

7.54
7.45
7.30
7.28
7.25
7.25
7.22
7.20
7.19
7.12
7.10
7.01
6.99
6.97
6.95
6.91
6.30
6.29
4.42
4.40
4.38
4.37
4.35
4.33
4.31
4.29
1.42
1.41
1.39
0.00
EtO
O
Cl
O
N
Ph
O
4a
Solvent CDCl3
Spectrometer 400 MHz
Nucleus 1H-NMR
1.04
1.07
3.00
3.08
4.02
4.00
2.08
1.00
2.07
3.04
f1 (ppm)

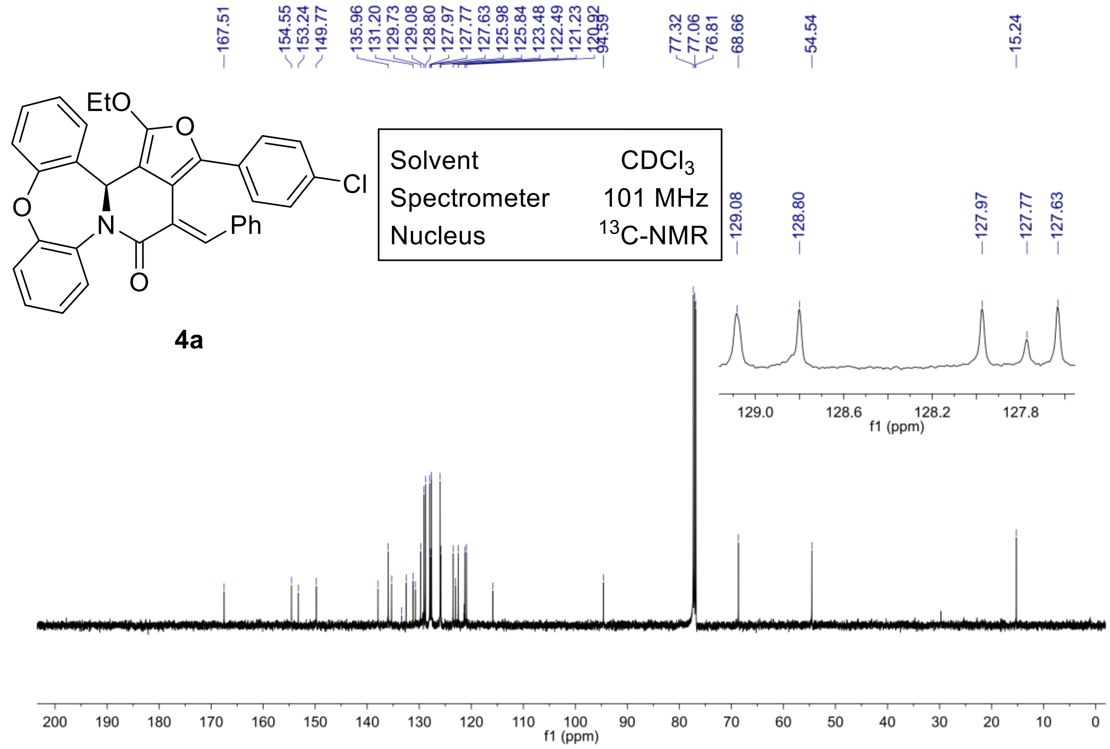
EtO
O
Cl
O
N
Ph
O
Solvent CDCl3
Spectrometer 101 MHz
Nucleus 13C-NMR
f1 (ppm)

4a

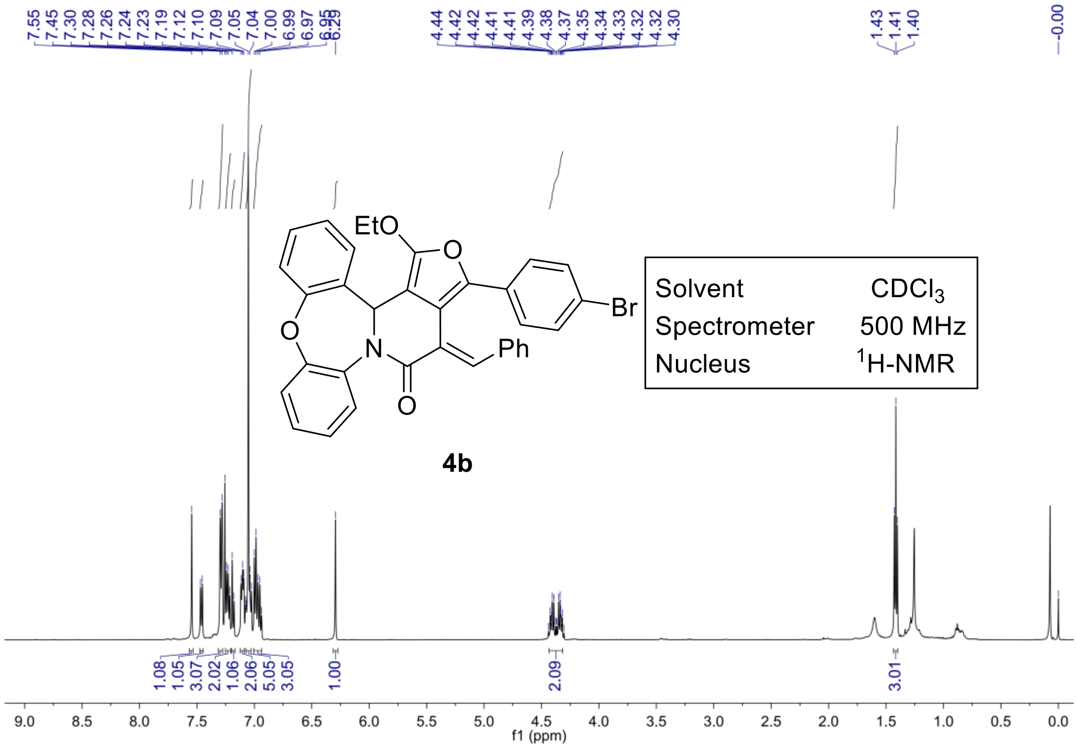
EtO
O
Br
O
N
Ph
O
Solvent CDCl3
Spectrometer 500 MHz
Nucleus 1H-NMR
f1 (ppm)

4b

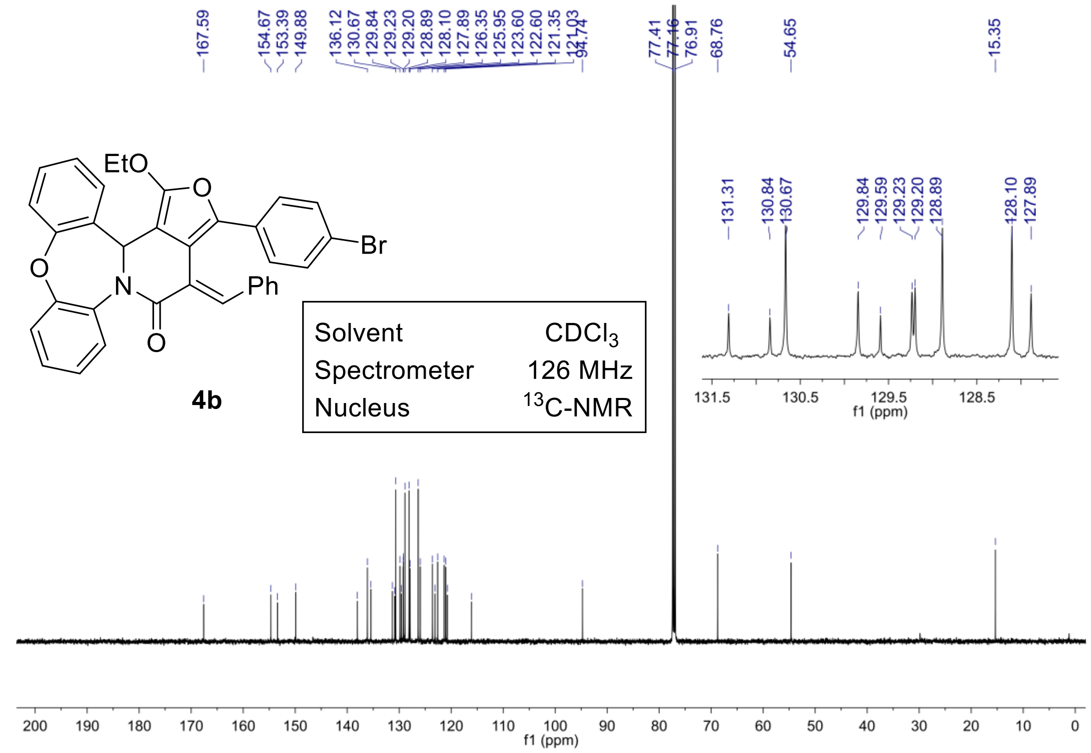
EtO
O
Br
N
Ph
O
O
4b
Solvent CDCl3
Spectrometer 126 MHz
Nucleus 13C-NMR
f1 (ppm)

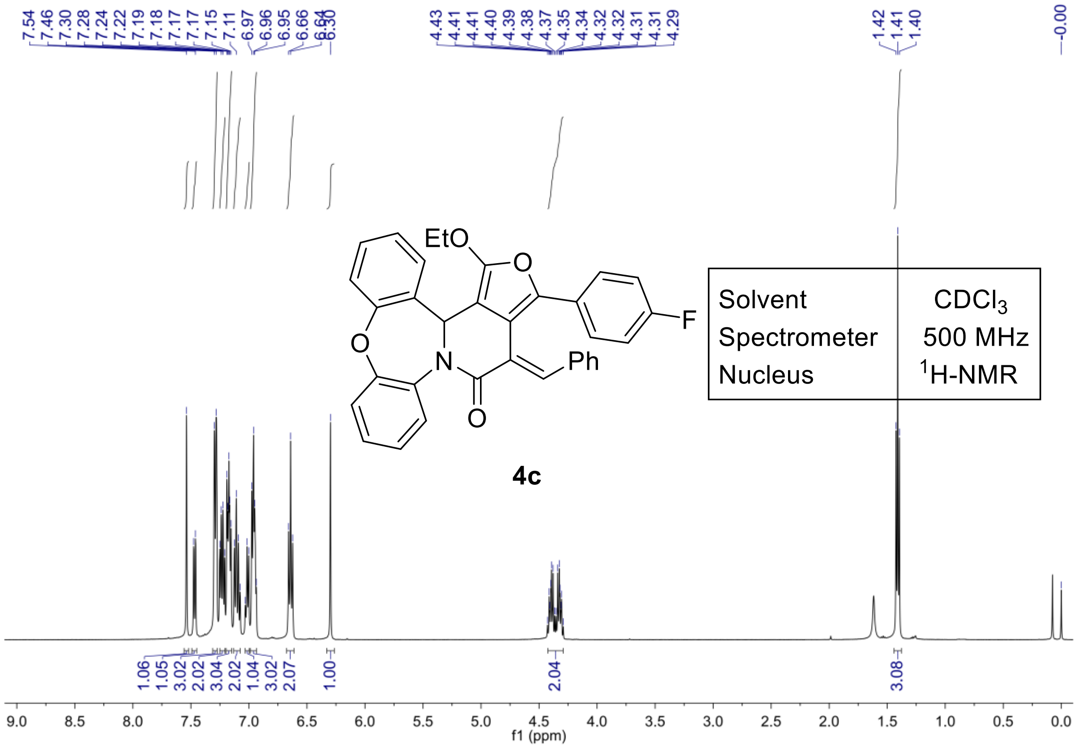
EtO
O
F
N
Ph
O
O
4c
Solvent CDCl3
Spectrometer 500 MHz
Nucleus 1H-NMR
f1 (ppm)

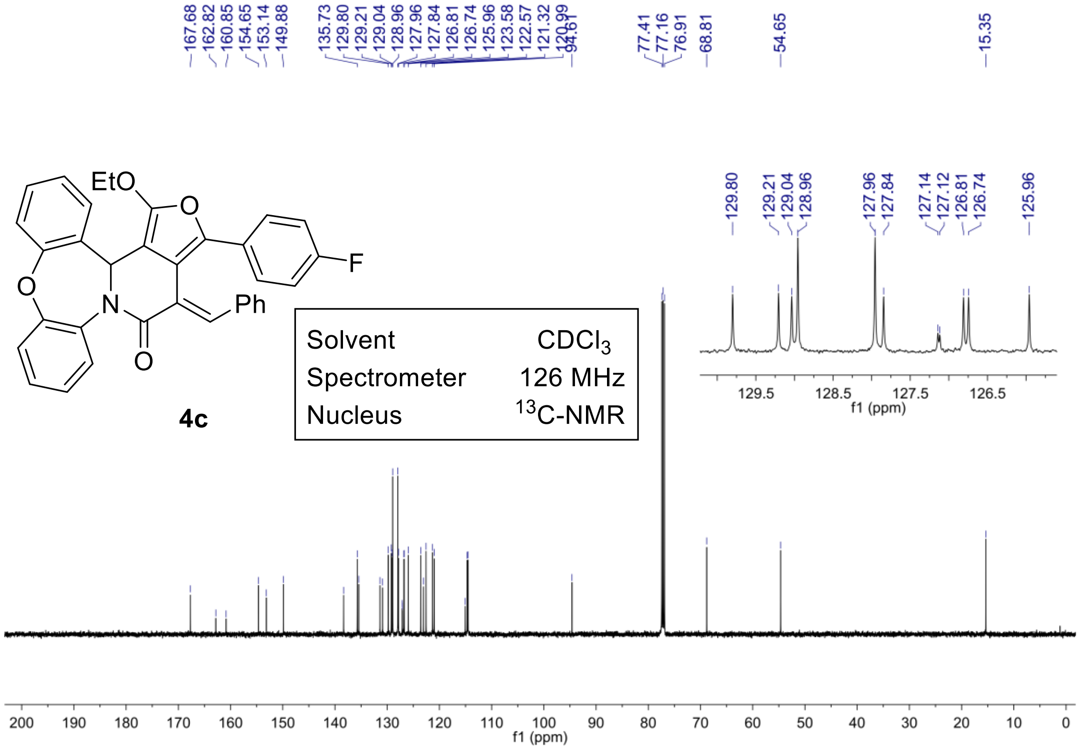
167.68
162.82
160.85
154.65
153.14
149.88
135.73
129.80
129.21
129.04
128.96
127.96
127.84
126.81
126.74
125.96
123.58
122.57
121.32
120.99
94.61
77.41
77.16
76.91
68.81
54.65
15.35
EtO
O
F
Ph
N
4c
Solvent CDCl3
Spectrometer 126 MHz
Nucleus 13C-NMR
129.80
129.21
129.04
128.96
127.96
127.84
127.14
127.12
126.81
126.74
125.96
f1 (ppm)
f1 (ppm)

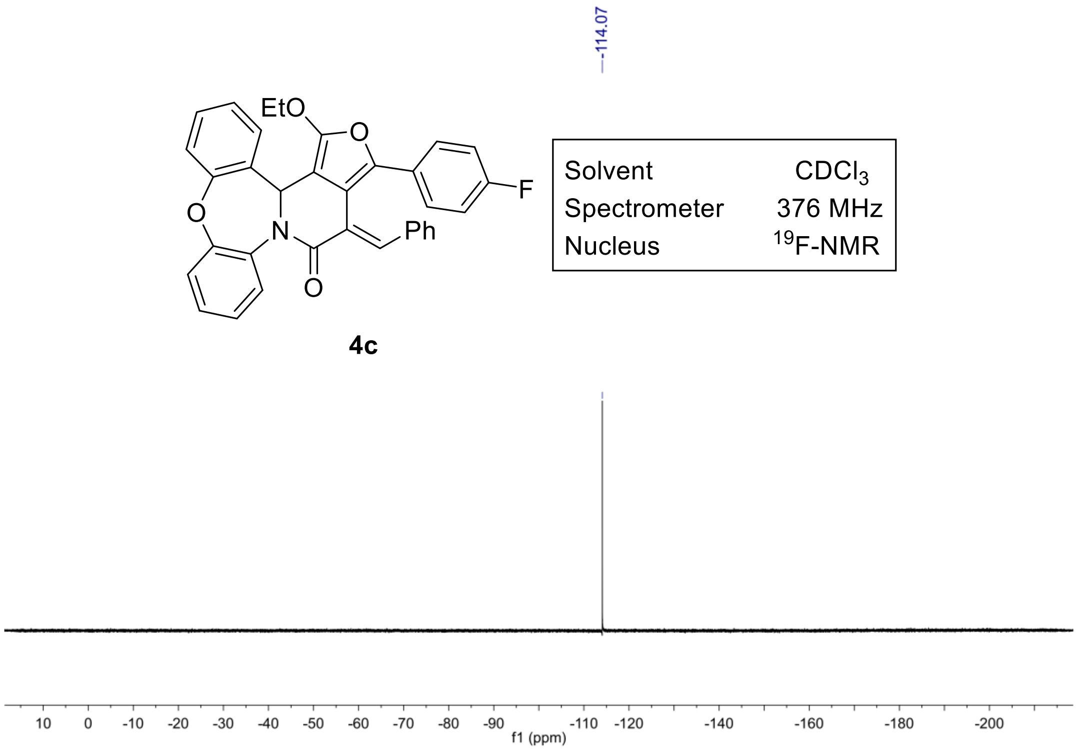
-114.07
EtO
O
F
Ph
N
4c
Solvent CDCl3
Spectrometer 376 MHz
Nucleus 19F-NMR
f1 (ppm)

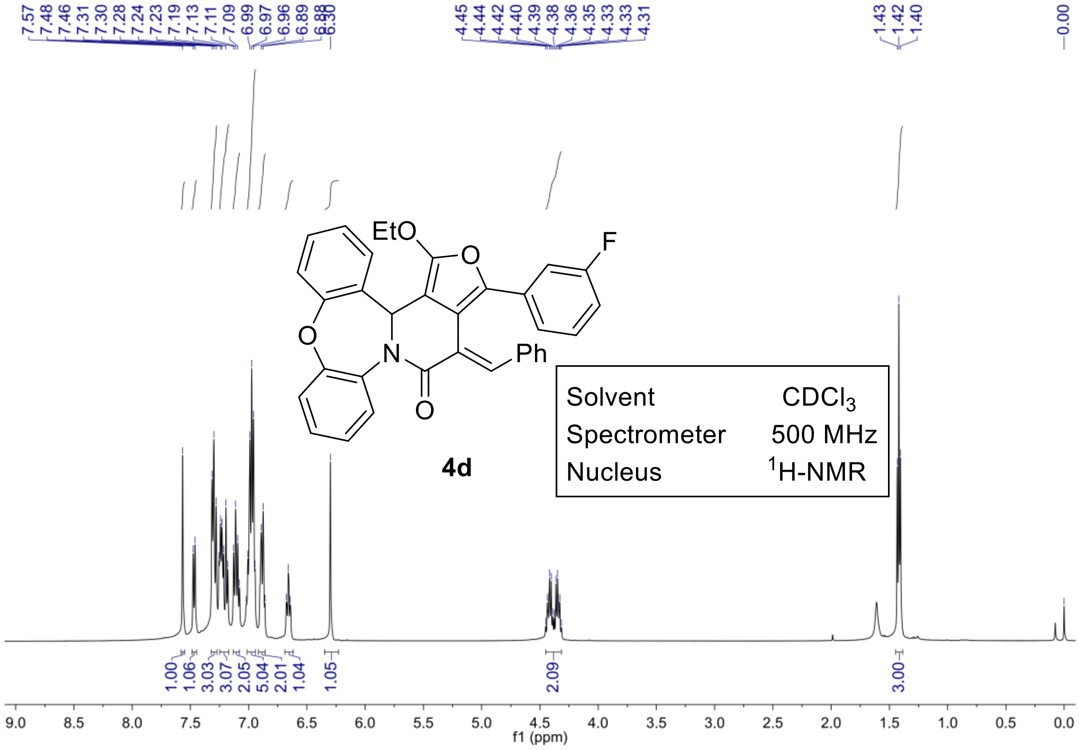

EtO
O
F
O
N
Ph
O
4d
Solvent CDCl3
Spectrometer 500 MHz
Nucleus 1H-NMR
f1 (ppm)

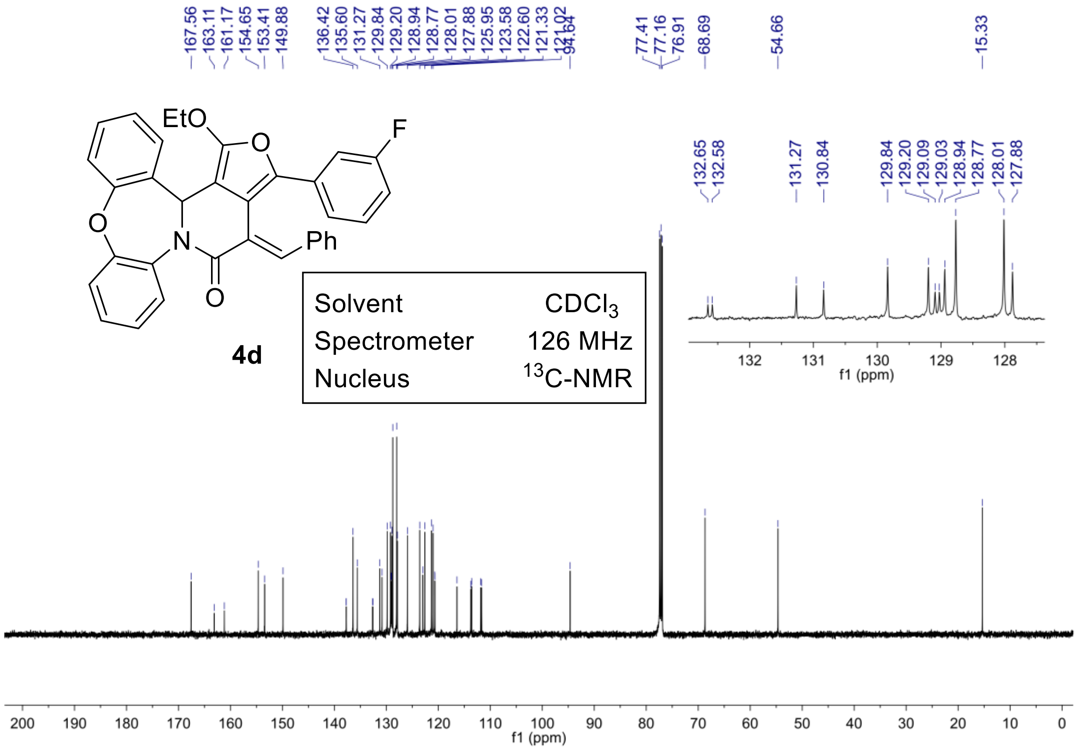

EtO
O
F
O
N
Ph
O
4d
Solvent CDCl3
Spectrometer 126 MHz
Nucleus 13C-NMR
f1 (ppm)

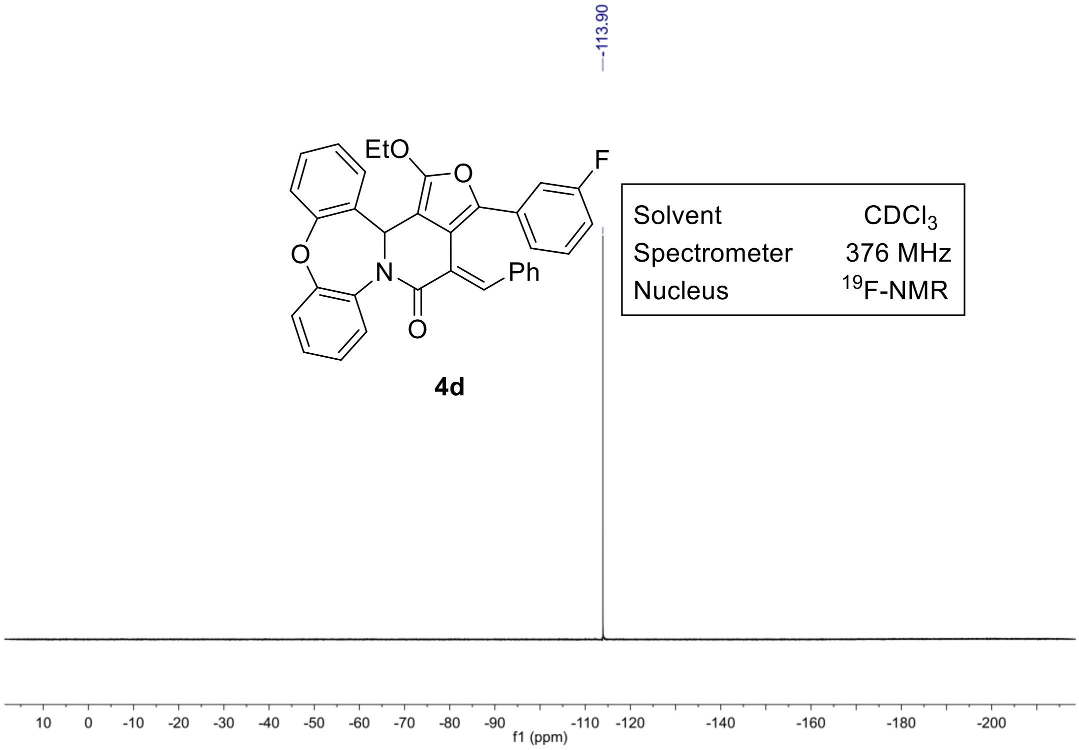
-113.90
EtO
F
Ph
O
N
O
4d
Solvent CDCl3
Spectrometer 376 MHz
Nucleus 19F-NMR
f1 (ppm)

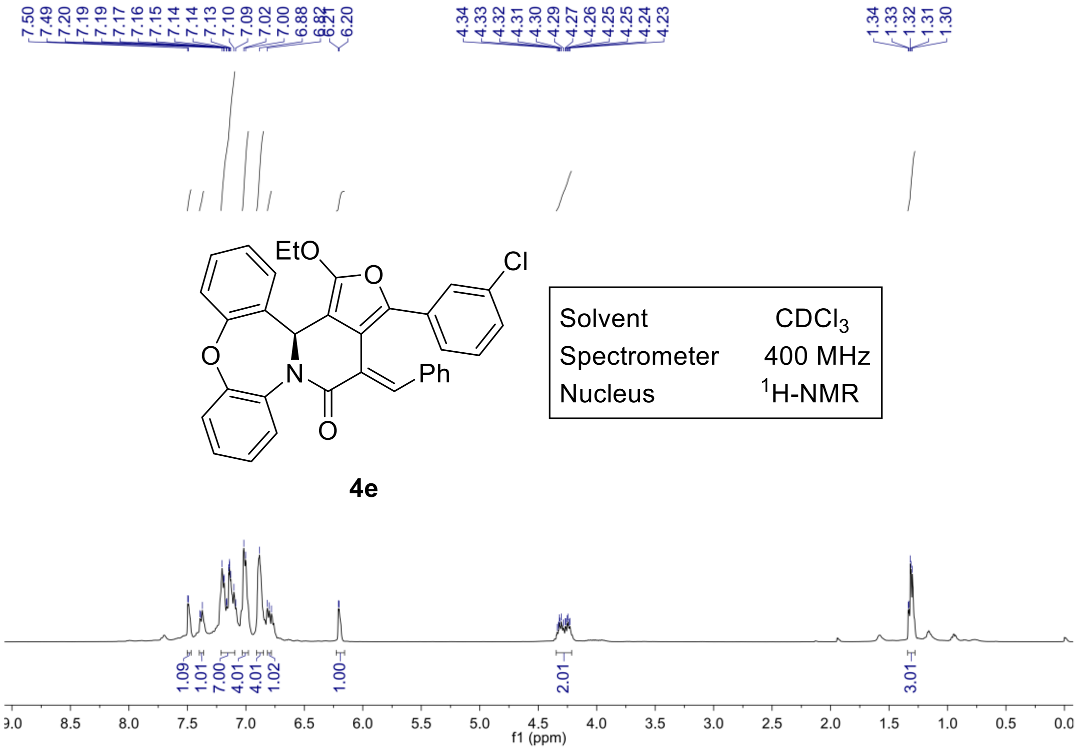
EtO
Cl
Ph
O
N
O
4e
Solvent CDCl3
Spectrometer 400 MHz
Nucleus 1H-NMR
f1 (ppm)

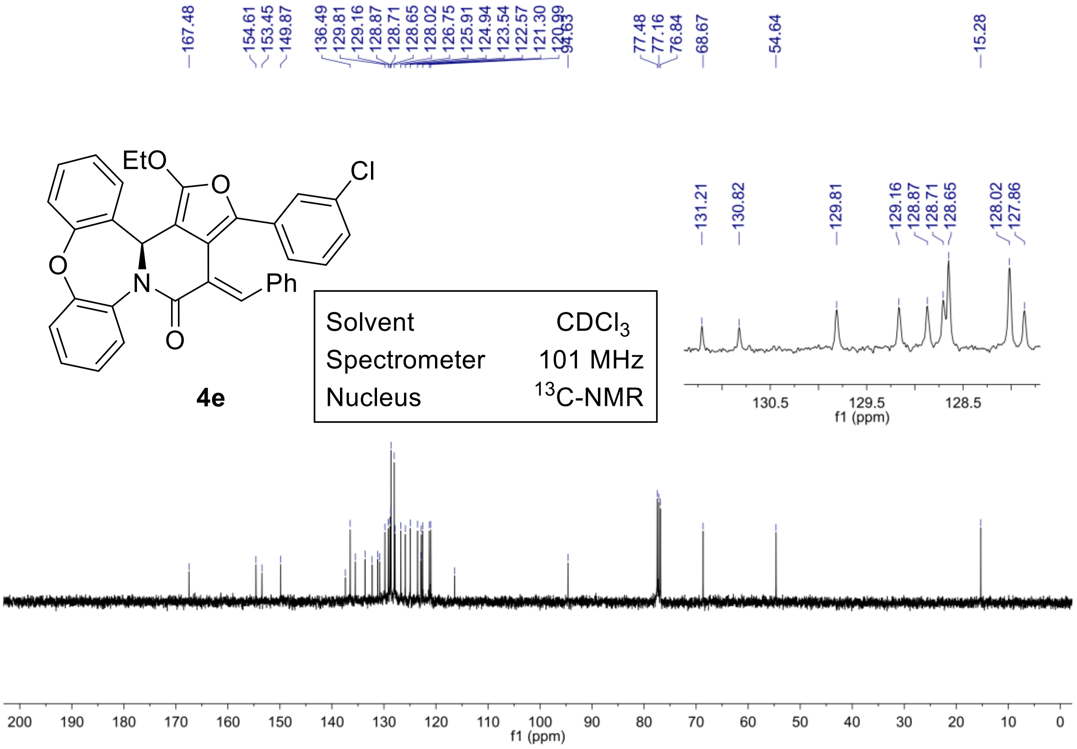
EtO
O
Cl
O
N
Ph
O
4e
Solvent CDCl3
Spectrometer 101 MHz
Nucleus 13C-NMR
f1 (ppm)

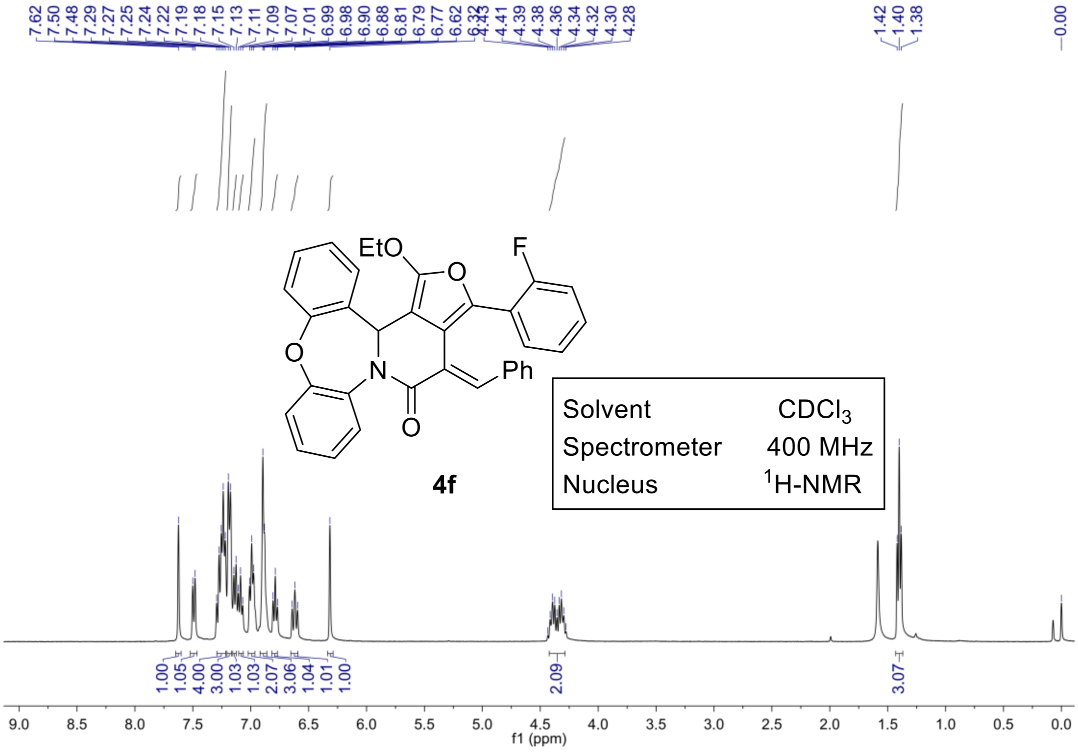
EtO
O
F
O
N
Ph
O
4f
Solvent CDCl3
Spectrometer 400 MHz
Nucleus 1H-NMR
f1 (ppm)

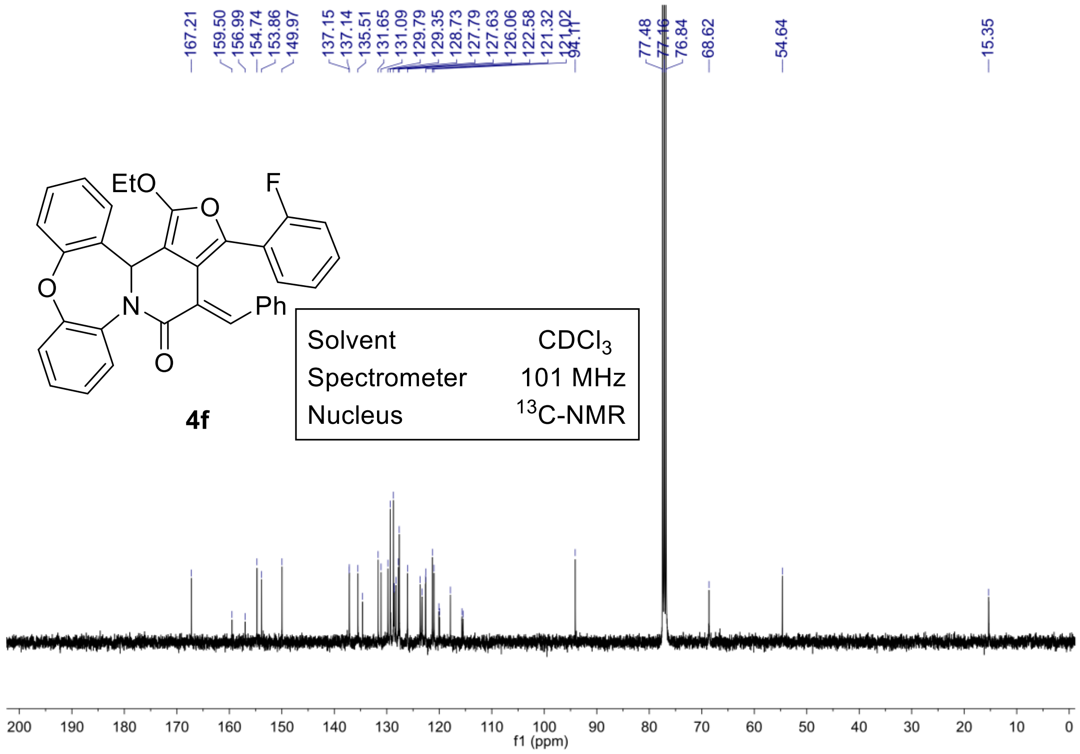
167.21
159.50
156.99
154.74
153.86
149.97
137.15
137.14
135.51
131.65
131.09
129.79
129.35
128.73
127.79
127.63
126.06
122.58
121.32
121.02
94.11
77.48
77.16
76.84
68.62
54.64
15.35
EtO
F
O
N
Ph
4f
Solvent CDCl3
Spectrometer 101 MHz
Nucleus 13C-NMR
f1 (ppm)

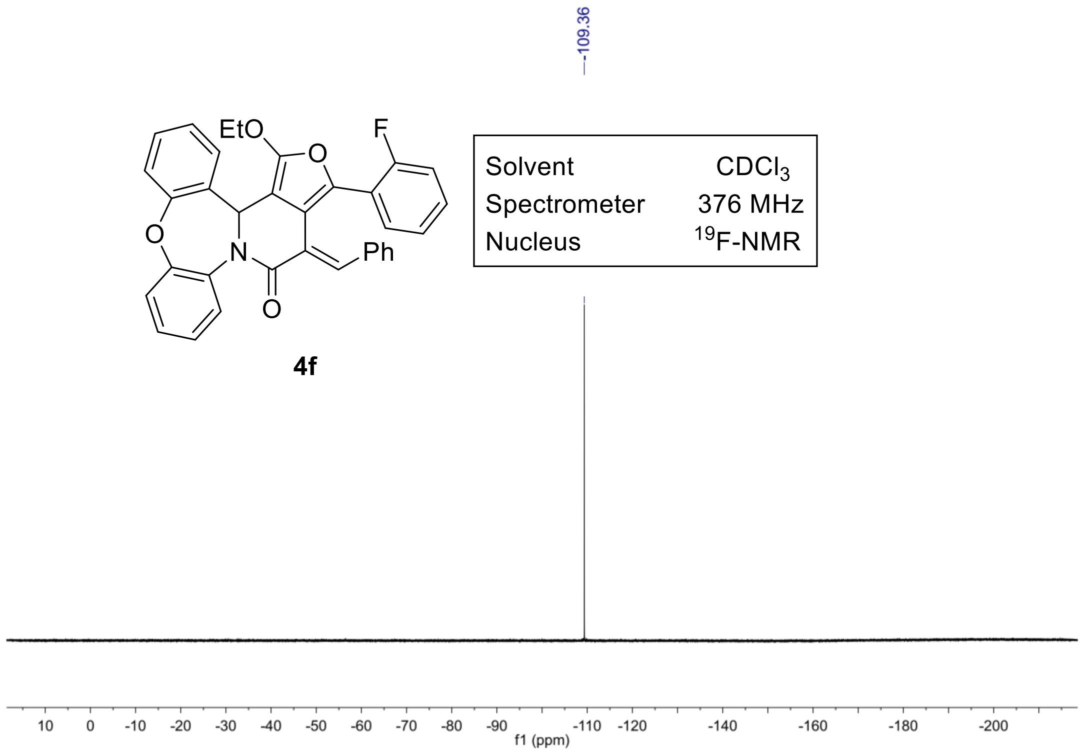
-109.36
EtO
F
O
N
Ph
4f
Solvent CDCl3
Spectrometer 376 MHz
Nucleus 19F-NMR
f1 (ppm)

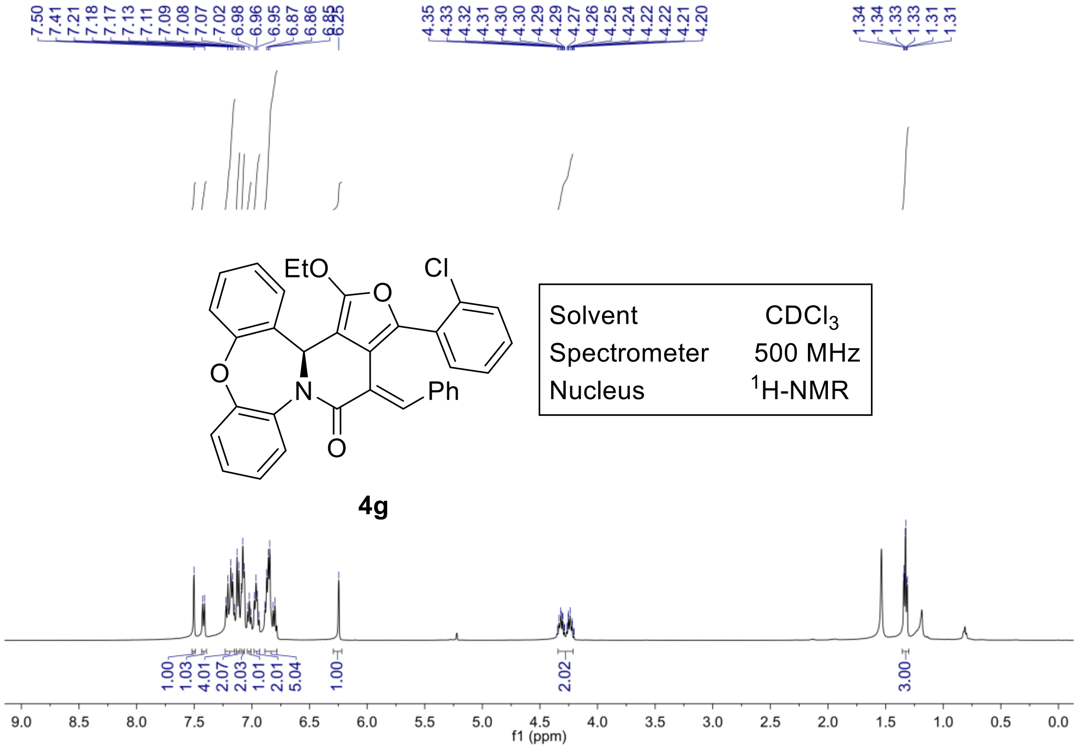
7.50 7.41 7.21 7.18 7.17 7.13 7.11 7.09 7.08 7.07 7.02 6.98 6.96 6.95 6.87 6.86 6.85 6.25
4.35 4.33 4.32 4.31 4.30 4.30 4.29 4.29 4.27 4.26 4.25 4.24 4.22 4.22 4.21 4.20
1.34 1.34 1.33 1.33 1.31 1.31
EtO
Cl
O
N
Ph
O
O
4g
Solvent CDCl3
Spectrometer 500 MHz
Nucleus 1H-NMR
1.00 1.03 4.01 2.07 2.03 1.01 2.01 5.04 1.00 2.02 3.00
9.0 8.5 8.0 7.5 7.0 6.5 6.0 5.5 5.0 4.5 4.0 3.5 3.0 2.5 2.0 1.5 1.0 0.5 0.0
f1 (ppm)

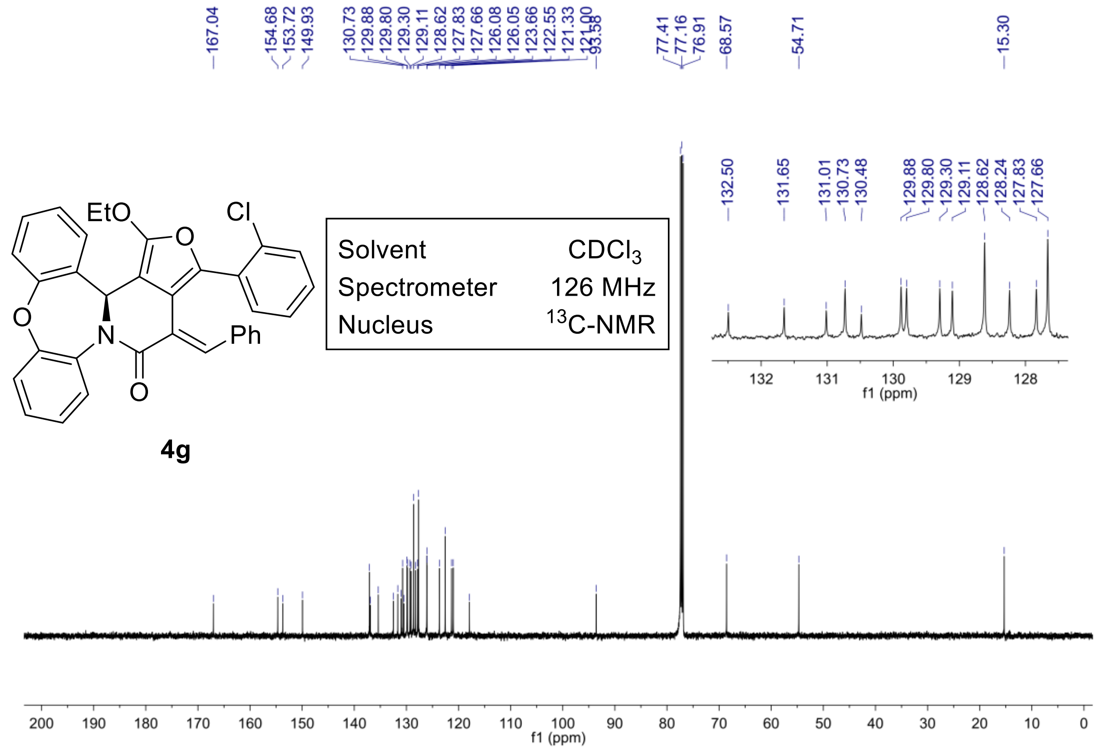
167.04 154.68 153.72 149.93 130.73 129.88 129.80 129.30 129.11 128.62 127.83 127.66 126.08 126.05 123.66 122.55 121.33 121.00 93.58 77.41 77.16 76.91 68.57 54.71 15.30
132.50 131.65 131.01 130.73 130.48 129.88 129.80 129.30 129.11 128.62 128.24 127.83 127.66
132 131 130 129 128
f1 (ppm)
EtO
Cl
O
N
Ph
O
O
4g
Solvent CDCl3
Spectrometer 126 MHz
Nucleus 13C-NMR
200 190 180 170 160 150 140 130 120 110 100 90 80 70 60 50 40 30 20 10 0
f1 (ppm)

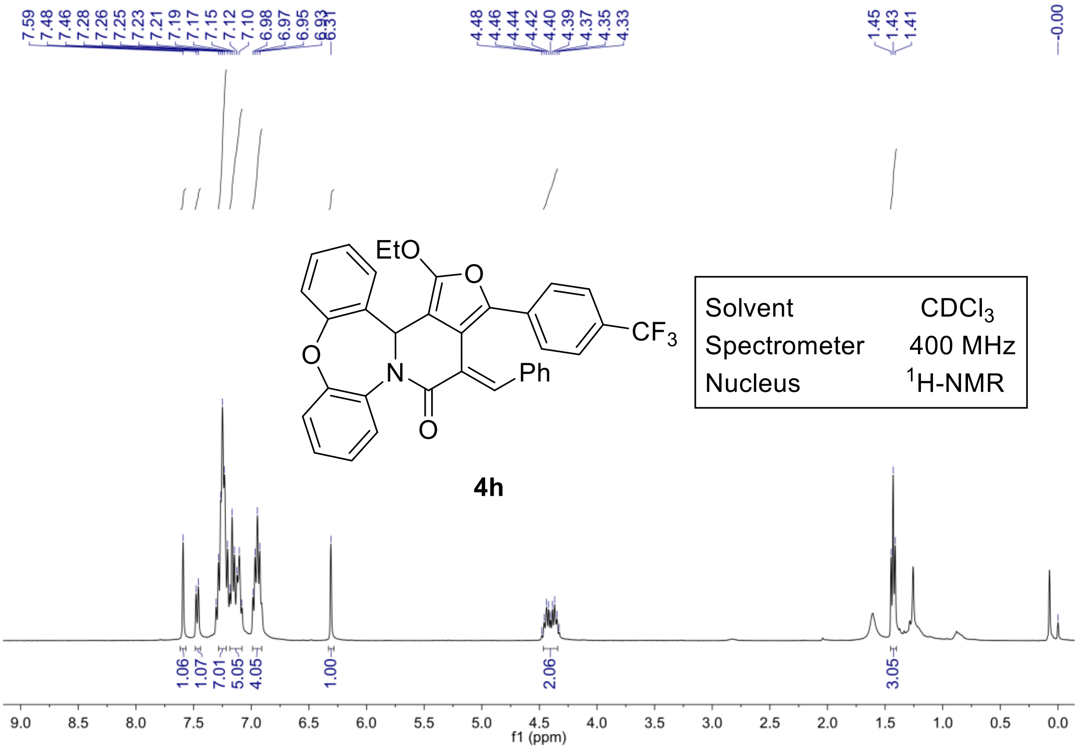

7.59 7.48 7.46 7.28 7.26 7.25 7.23 7.21 7.19 7.17 7.15 7.12 7.10 6.98 6.97 6.95 6.93 6.31
4.48 4.46 4.44 4.42 4.40 4.39 4.37 4.35 4.33
1.45 1.43 1.41
-0.00
EtO
O
CF3
O
N
Ph
O
4h
Solvent CDCl3
Spectrometer 400 MHz
Nucleus 1H-NMR
1.06 1.07 7.01 5.05 4.05 1.00 2.06 3.05
f1 (ppm)

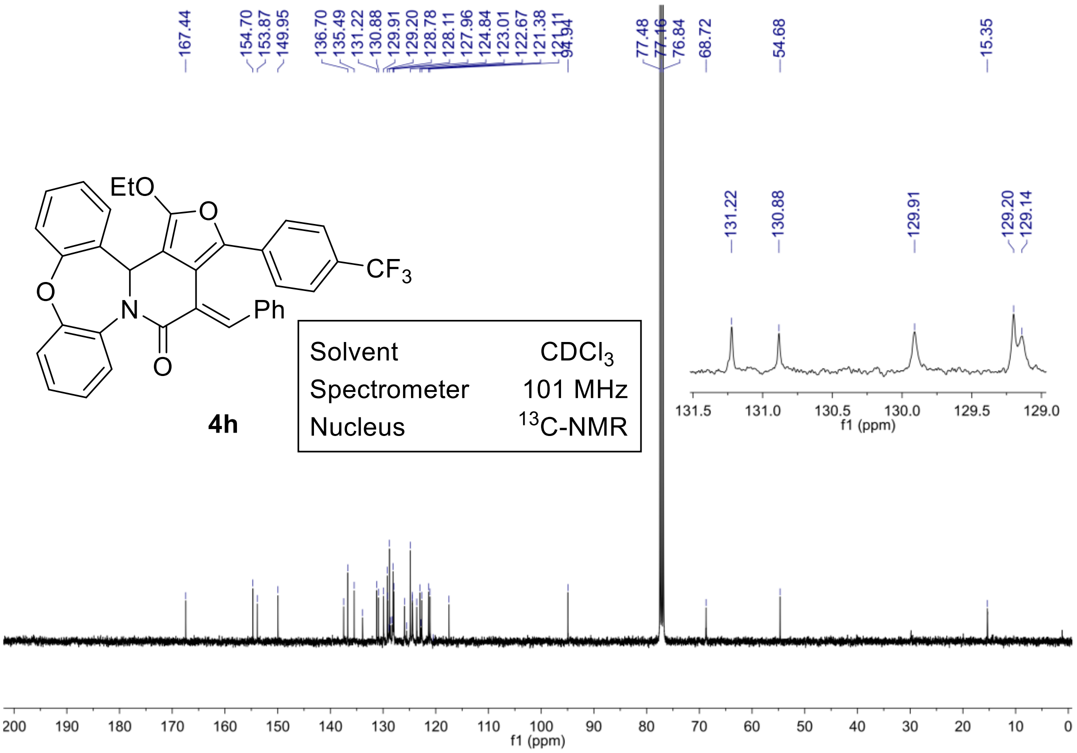

167.44
154.70 153.87 149.95
136.70 135.49 131.22 130.88 129.91 129.20 128.78 128.11 127.96 124.84 123.01 122.67 121.38 121.11 94.94
77.48 77.16 76.84 68.72
54.68
15.35
EtO
O
CF3
O
N
Ph
O
4h
Solvent CDCl3
Spectrometer 101 MHz
Nucleus 13C-NMR
131.22 130.88 129.91 129.20 129.14
f1 (ppm)

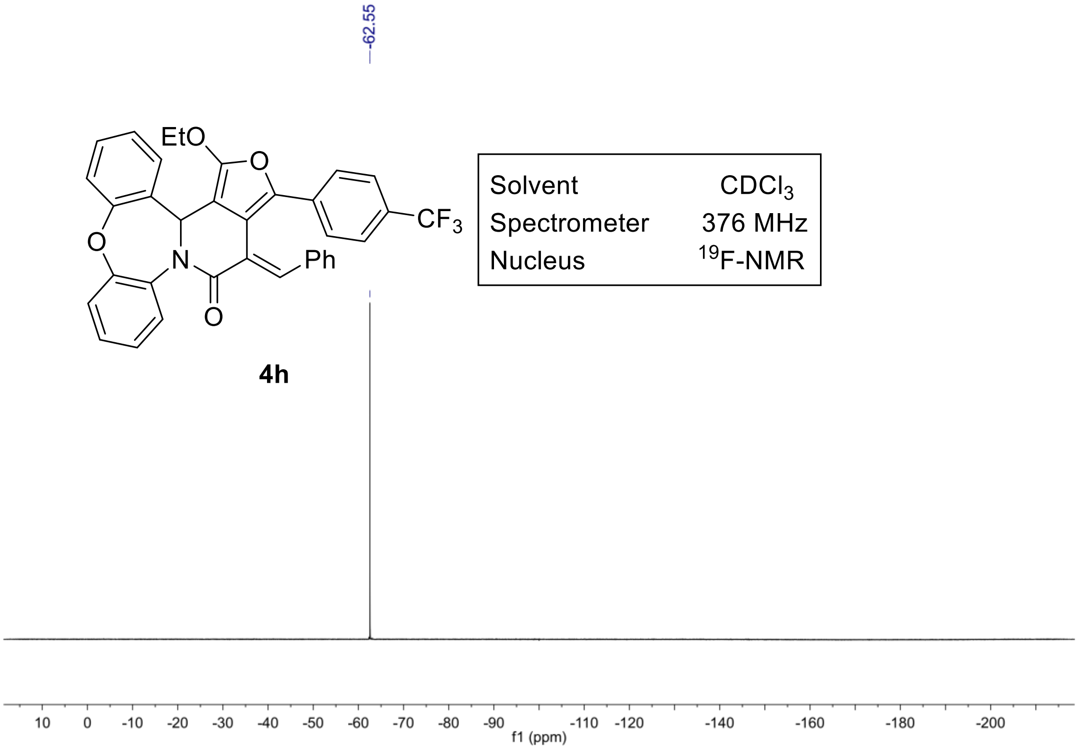

-62.55
EtO
O
N
O
CF3
Ph
4h
Solvent CDCl3
Spectrometer 376 MHz
Nucleus 19F-NMR
10 0 -10 -20 -30 -40 -50 -60 -70 -80 -90 -110 -120 -140 -160 -180 -200
f1 (ppm)

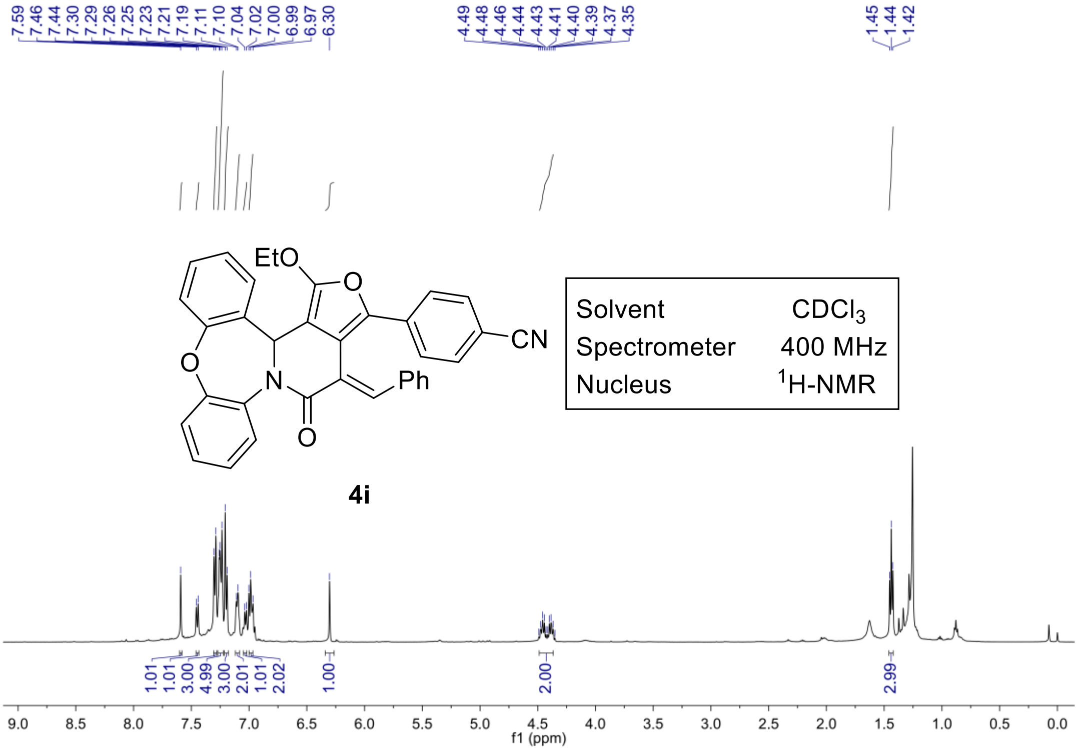

7.59 7.46 7.44 7.30 7.29 7.26 7.25 7.23 7.21 7.19 7.11 7.10 7.04 7.02 7.00 6.99 6.97 6.30
4.49 4.48 4.46 4.44 4.43 4.41 4.40 4.39 4.37 4.35
1.45 1.44 1.42
EtO
O
N
O
CN
Ph
4i
Solvent CDCl3
Spectrometer 400 MHz
Nucleus 1H-NMR
1.01 1.01 3.00 4.99 3.00 2.01 1.01 2.02
1.00
2.00
2.99
9.0 8.5 8.0 7.5 7.0 6.5 6.0 5.5 5.0 4.5 4.0 3.5 3.0 2.5 2.0 1.5 1.0 0.5 0.0
f1 (ppm)

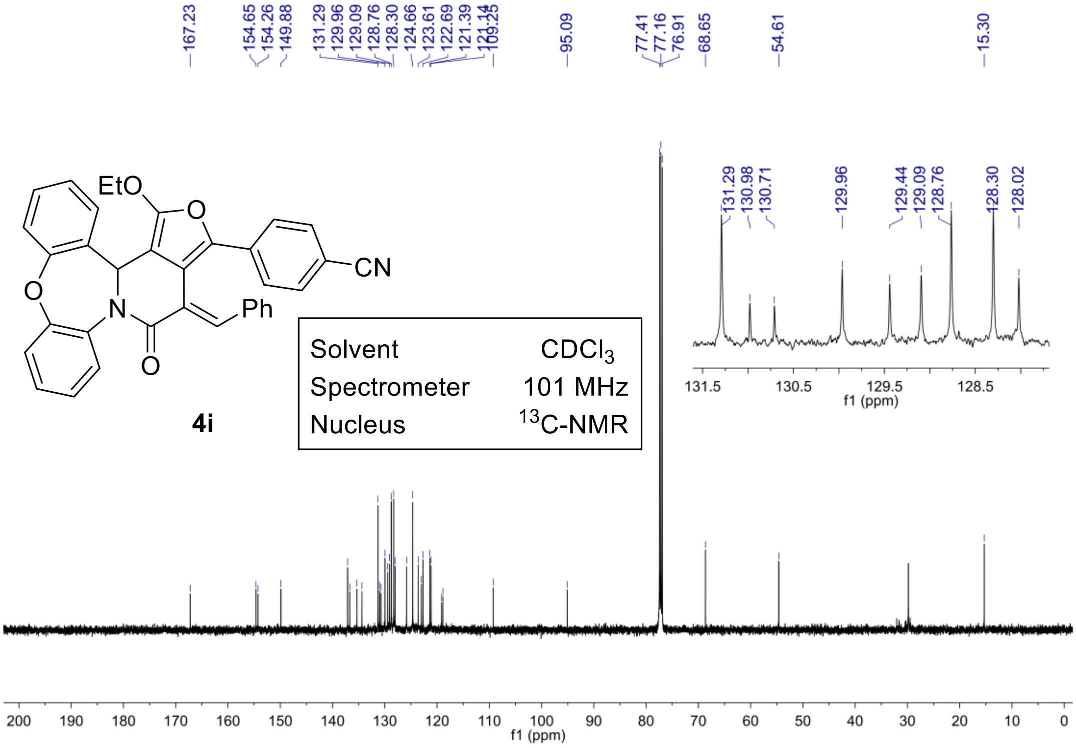
167.23
154.65
154.26
149.88
131.29
129.96
129.09
128.76
128.30
124.66
123.61
122.69
121.39
95.09
77.41
77.16
76.91
68.65
54.61
15.30
EtO
O
CN
Ph
N
4i
Solvent CDCl$_3$
Spectrometer 101 MHz
Nucleus ^{13}C-NMR
131.29
130.98
130.71
129.96
129.44
129.09
128.76
128.30
128.02
f1 (ppm)

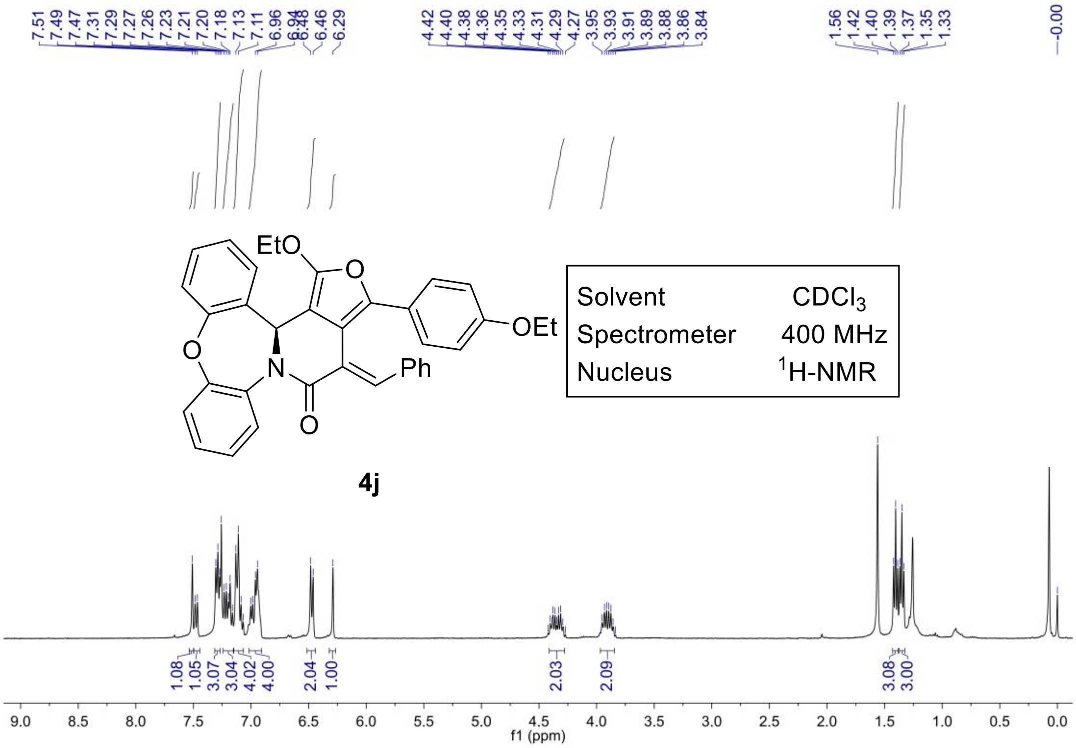
7.51
7.49
7.47
7.31
7.29
7.27
7.26
7.23
7.21
7.20
7.18
7.13
7.11
6.96
6.46
6.29
4.42
4.40
4.38
4.36
4.35
4.33
4.31
4.29
4.27
3.95
3.93
3.91
3.89
3.88
3.86
3.84
1.56
1.42
1.40
1.39
1.37
1.35
1.33
-0.00
EtO
O
OEt
Ph
N
4j
Solvent CDCl$_3$
Spectrometer 400 MHz
Nucleus ^{1}H-NMR
1.08
1.05
3.07
3.04
4.02
4.00
2.04
1.00
2.03
2.09
3.08
3.00
f1 (ppm)

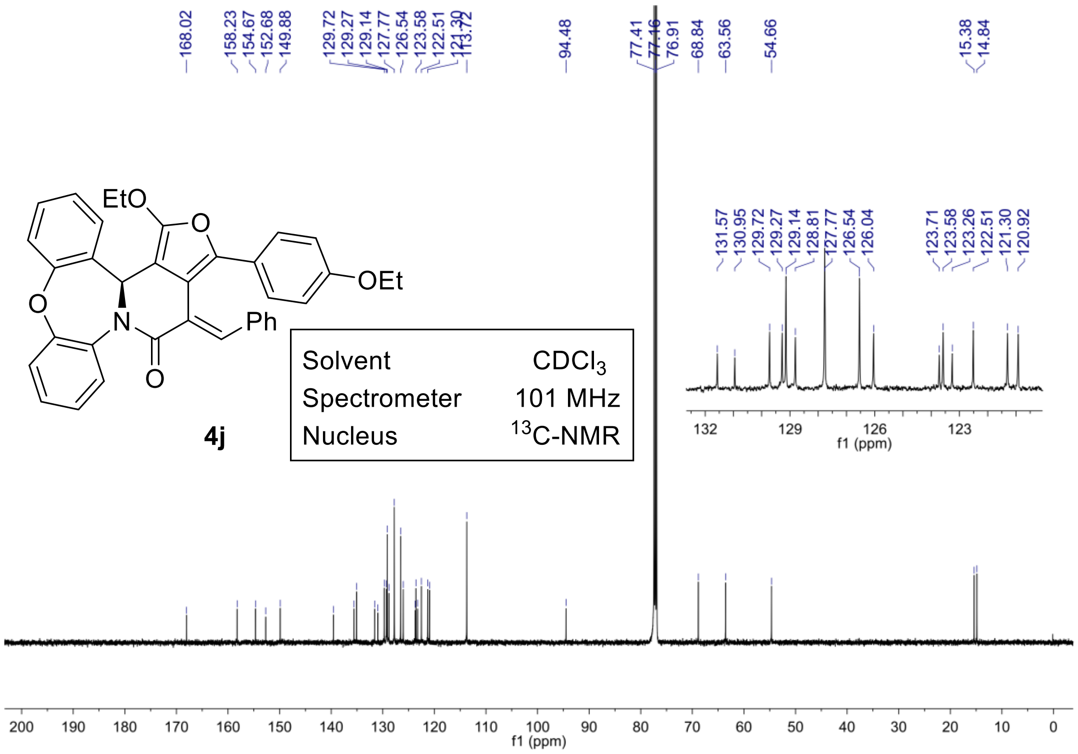

168.02
158.23
154.67
152.68
149.88
129.72
129.27
129.14
127.77
126.54
123.58
122.51
94.48
77.41
76.91
68.84
63.56
54.66
15.38
14.84
EtO
O
OEt
N
Ph
O
4j
131.57
130.95
129.72
129.27
129.14
128.81
127.77
126.54
126.04
123.71
123.58
123.26
122.51
121.30
120.92
Solvent CDCl3
Spectrometer 101 MHz
Nucleus 13C-NMR
f1 (ppm)

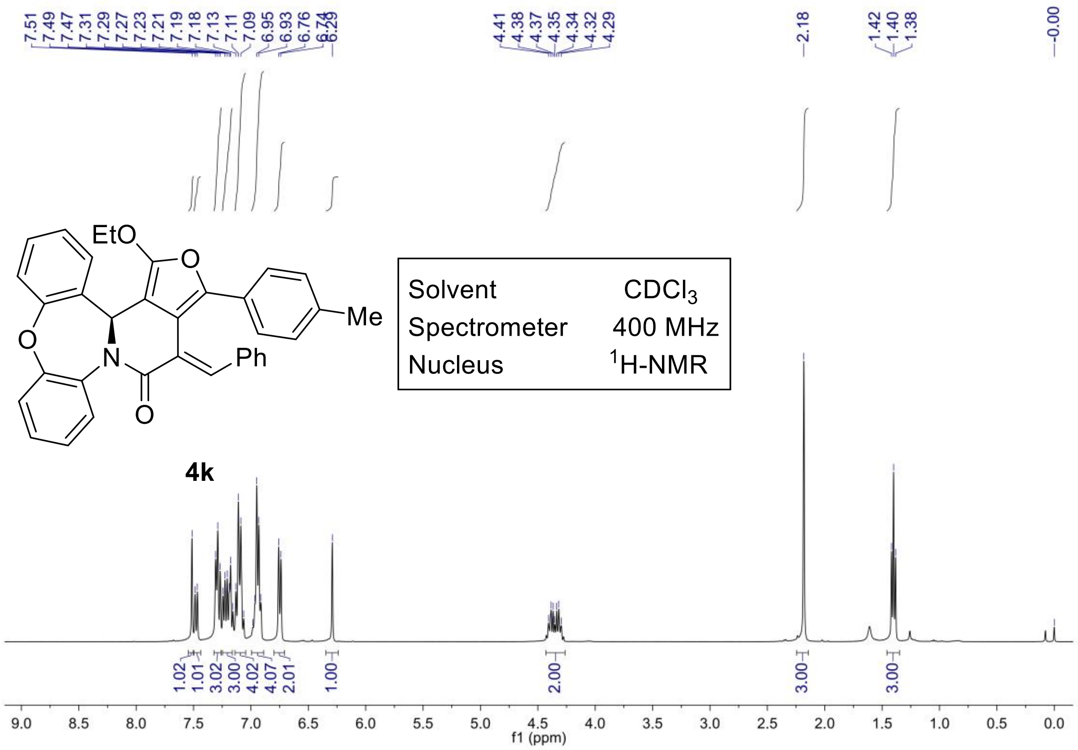

7.51
7.49
7.47
7.31
7.29
7.27
7.23
7.21
7.19
7.18
7.13
7.11
7.09
6.95
6.93
6.76
4.41
4.38
4.37
4.35
4.34
4.32
4.29
2.18
1.42
1.40
1.38
-0.00
EtO
O
Me
N
Ph
O
4k
Solvent CDCl3
Spectrometer 400 MHz
Nucleus 1H-NMR
f1 (ppm)

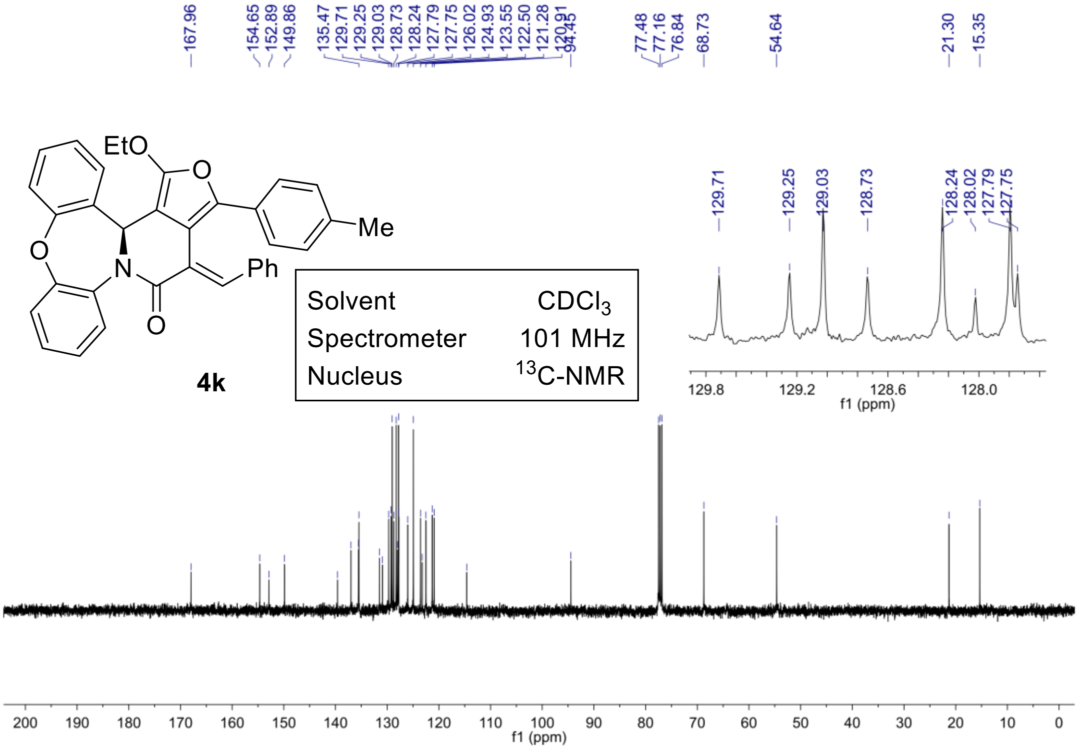
167.96
154.65
152.89
149.86
135.47
129.71
129.25
129.03
128.73
128.24
127.79
127.75
126.02
124.93
123.55
122.50
121.28
120.91
94.45
77.48
77.16
76.84
68.73
54.64
21.30
15.35
EtO
O
Me
N
Ph
O
4k
Solvent CDCl3
Spectrometer 101 MHz
Nucleus 13C-NMR
129.71
129.25
129.03
128.73
128.24
128.02
127.79
127.75
129.8
129.2
128.6
128.0
f1 (ppm)
200
190
180
170
160
150
140
130
120
110
100
90
80
70
60
50
40
30
20
10
0
f1 (ppm)

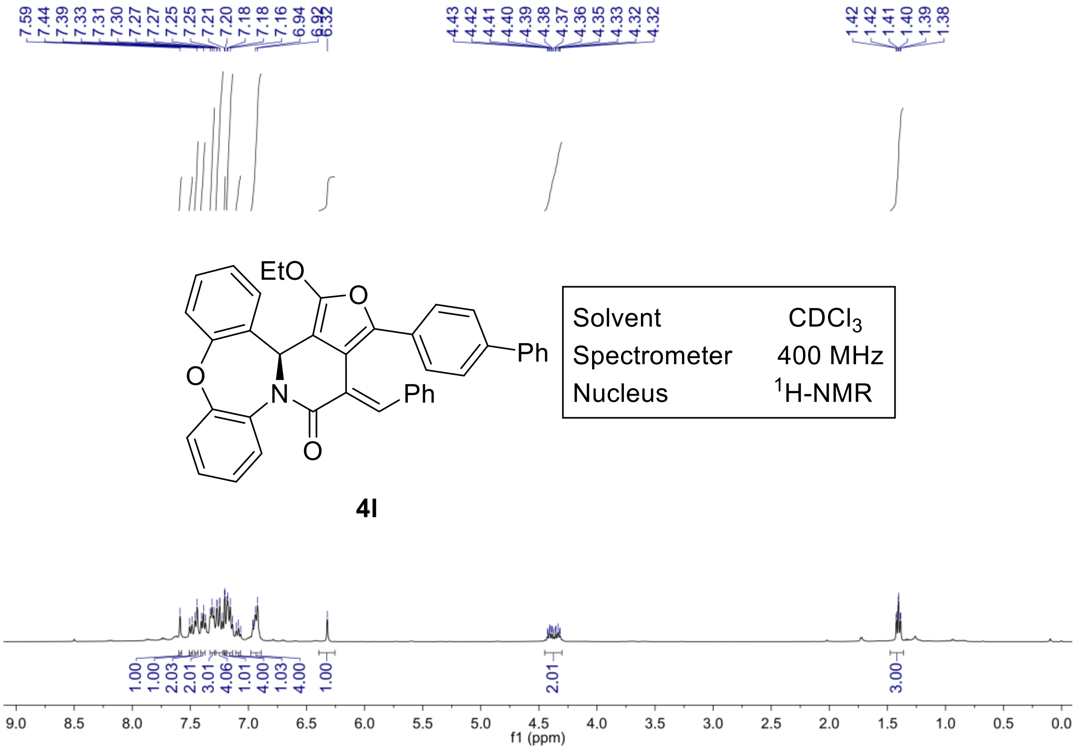
7.59
7.44
7.39
7.33
7.31
7.30
7.27
7.27
7.25
7.25
7.21
7.20
7.18
7.16
6.94
6.92
6.32
4.43
4.42
4.41
4.40
4.39
4.38
4.37
4.36
4.35
4.33
4.32
4.32
1.42
1.42
1.41
1.40
1.39
1.38
EtO
O
Ph
N
Ph
O
4l
Solvent CDCl3
Spectrometer 400 MHz
Nucleus 1H-NMR
1.00
1.00
2.03
2.01
3.01
4.06
1.01
4.00
1.03
4.00
1.00
2.01
3.00
9.0
8.5
8.0
7.5
7.0
6.5
6.0
5.5
5.0
4.5
4.0
3.5
3.0
2.5
2.0
1.5
1.0
0.5
0.0
f1 (ppm)

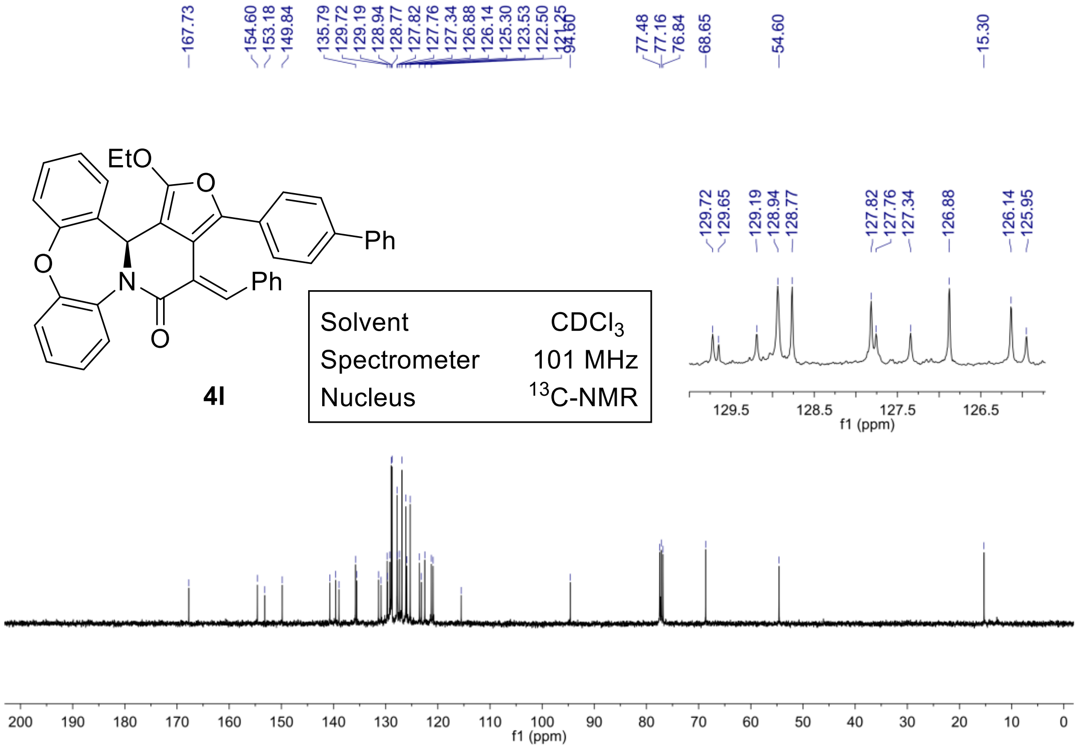
167.73
154.60
153.18
149.84
135.79
129.72
129.19
128.94
128.77
127.82
127.76
127.34
126.88
126.14
125.30
123.53
122.50
121.25
94.60
77.48
77.16
76.84
68.65
54.60
15.30
EtO
O
Ph
Ph
N
O
O
4l
Solvent CDCl3
Spectrometer 101 MHz
Nucleus 13C-NMR
129.72
129.65
129.19
128.94
128.77
127.82
127.76
127.34
126.88
126.14
125.95
129.5 128.5 127.5 126.5
f1 (ppm)
200 190 180 170 160 150 140 130 120 110 100 90 80 70 60 50 40 30 20 10 0
f1 (ppm)

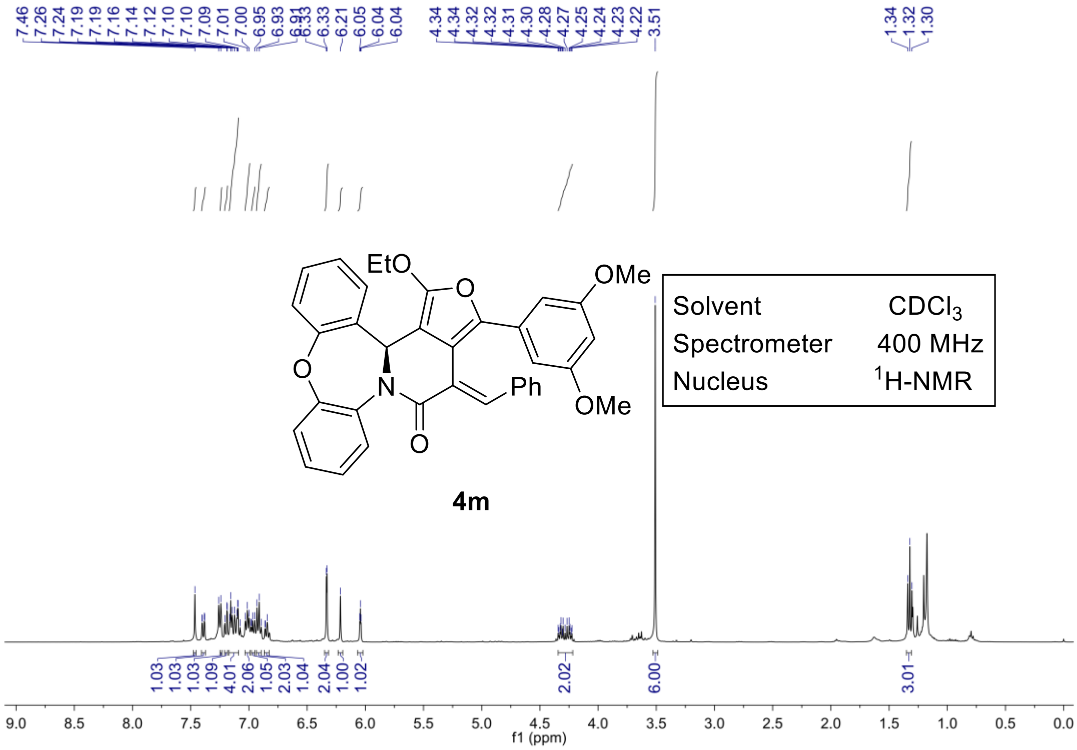
7.46
7.26
7.24
7.19
7.16
7.14
7.12
7.10
7.09
7.01
7.00
6.95
6.93
6.91
6.33
6.21
6.05
6.04
6.04
4.34
4.34
4.32
4.32
4.31
4.30
4.28
4.27
4.25
4.24
4.23
4.22
3.51
1.34
1.32
1.30
EtO
O
OMe
O
N
Ph
OMe
O
4m
Solvent CDCl3
Spectrometer 400 MHz
Nucleus 1H-NMR
1.03
1.03
1.03
1.09
4.01
2.06
1.05
2.03
1.04
2.04
1.00
1.02
2.02
6.00
3.01
9.0 8.5 8.0 7.5 7.0 6.5 6.0 5.5 5.0 4.5 4.0 3.5 3.0 2.5 2.0 1.5 1.0 0.5 0.0
f1 (ppm)

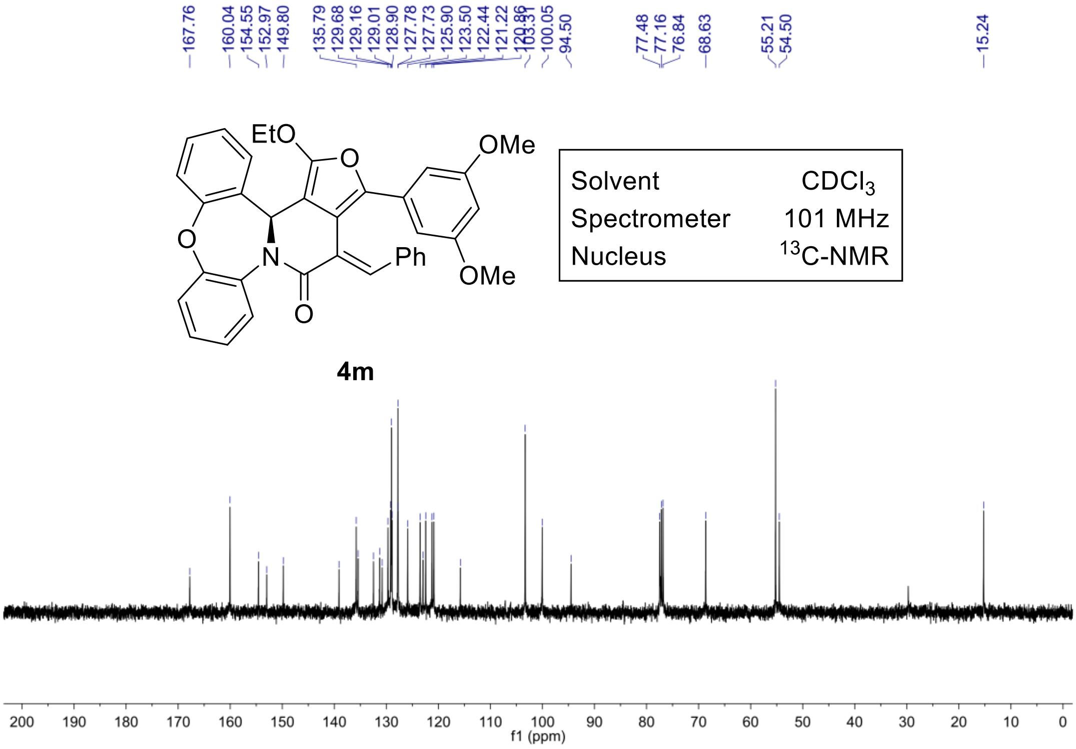

167.76
160.04
154.55
152.97
149.80
135.79
129.68
129.16
129.01
128.90
127.78
127.73
125.90
123.50
122.44
121.22
100.05
94.50
77.48
77.16
76.84
68.63
55.21
54.50
15.24
EtO
O
OMe
OMe
N
O
Ph
4m
Solvent CDCl3
Spectrometer 101 MHz
Nucleus 13C-NMR
f1 (ppm)

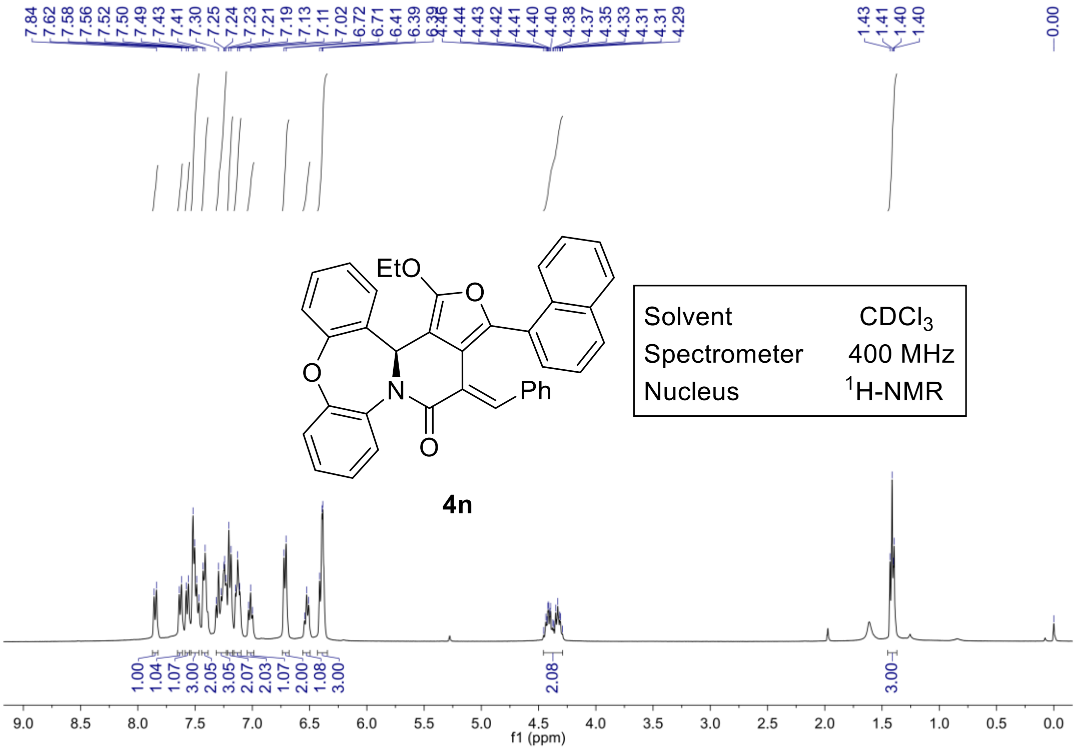

7.84
7.62
7.58
7.56
7.52
7.50
7.49
7.43
7.41
7.30
7.25
7.24
7.23
7.21
7.19
7.13
7.11
7.02
6.72
6.71
6.41
6.39
4.44
4.43
4.42
4.41
4.40
4.40
4.38
4.37
4.35
4.33
4.31
4.31
4.29
1.43
1.41
1.40
1.40
0.00
EtO
O
N
O
Ph
4n
Solvent CDCl3
Spectrometer 400 MHz
Nucleus 1H-NMR
1.00
1.04
1.07
3.00
2.05
3.05
2.07
2.03
1.07
2.00
1.08
3.00
2.08
3.00
f1 (ppm)

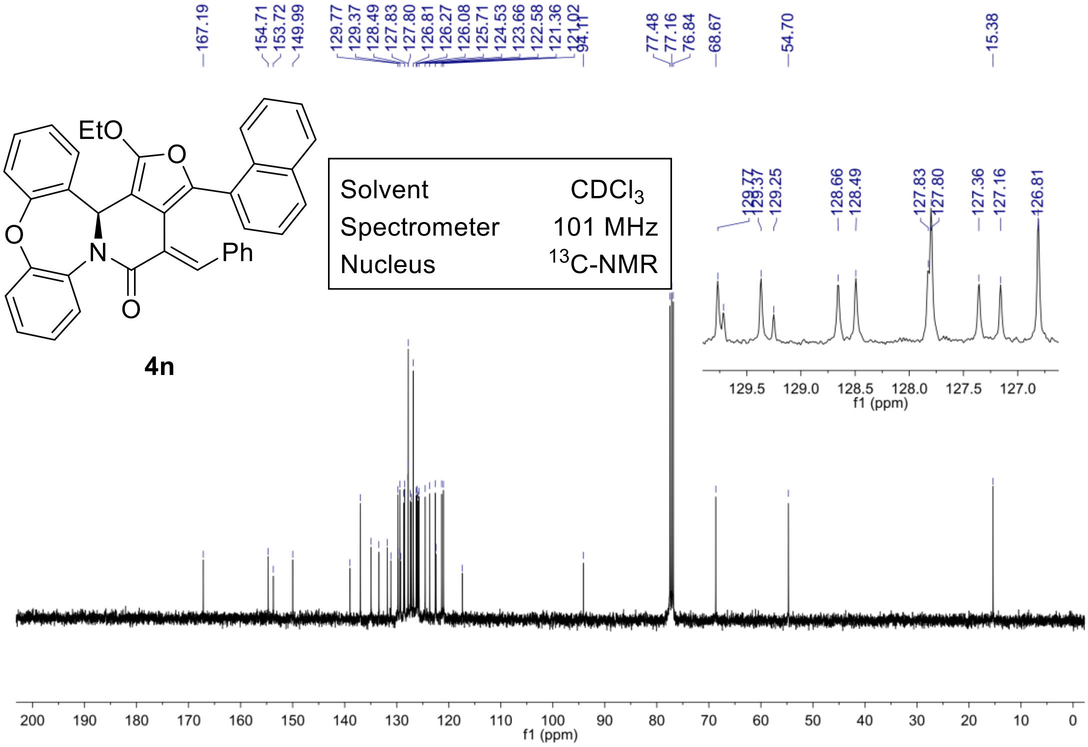
167.19
154.71
153.72
149.99
129.77
129.37
128.49
127.83
127.80
126.81
126.27
126.08
125.71
124.53
123.66
122.58
121.36
121.02
94.11
77.48
77.16
76.84
68.67
54.70
15.38
EtO
O
N
O
Ph
O
4n
Solvent CDCl3
Spectrometer 101 MHz
Nucleus 13C-NMR
129.77
129.37
129.25
128.66
128.49
127.83
127.80
127.36
127.16
126.81
129.5 129.0 128.5 128.0 127.5 127.0
f1 (ppm)
200 190 180 170 160 150 140 130 120 110 100 90 80 70 60 50 40 30 20 10 0
f1 (ppm)

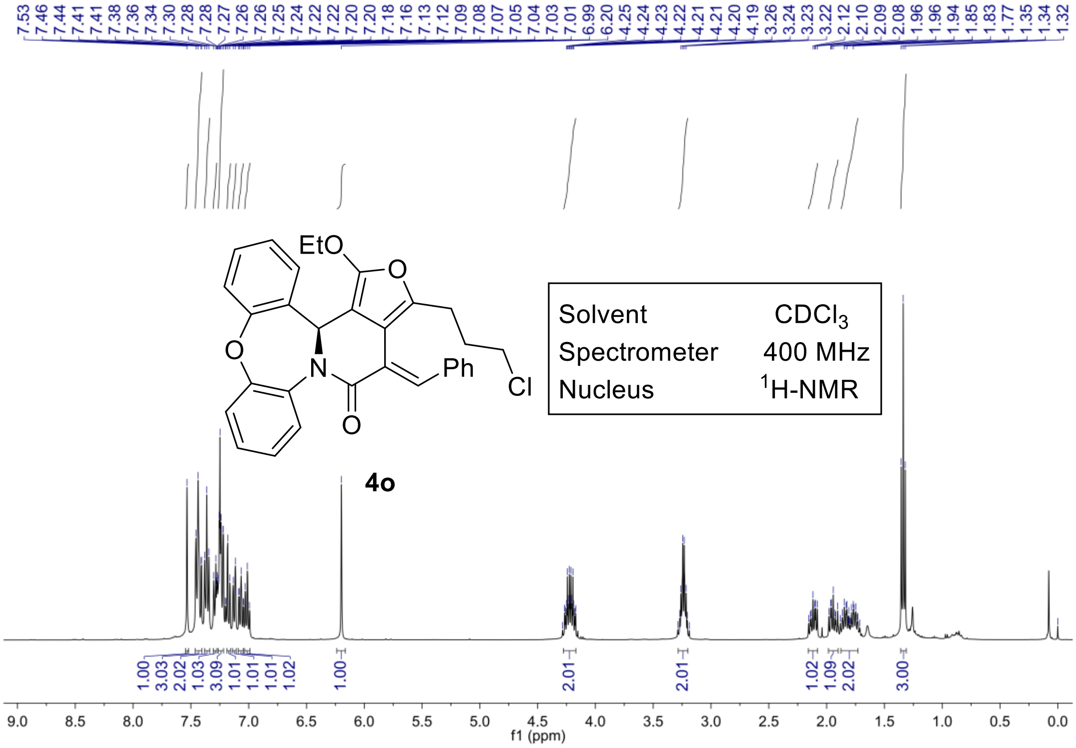
7.53
7.46
7.44
7.41
7.38
7.36
7.34
7.30
7.28
7.28
7.27
7.26
7.26
7.25
7.24
7.22
7.22
7.20
7.20
7.18
7.16
7.13
7.12
7.09
7.08
7.07
7.05
7.04
7.03
7.01
6.99
6.20
4.25
4.24
4.23
4.22
4.21
4.21
4.20
4.19
3.26
3.24
3.23
3.22
2.12
2.10
2.09
2.08
1.96
1.94
1.85
1.83
1.77
1.35
1.34
1.32
EtO
O
O
N
O
Ph
Cl
4o
Solvent CDCl3
Spectrometer 400 MHz
Nucleus 1H-NMR
1.00
3.03
2.02
1.03
3.09
1.01
1.01
1.01
1.02
1.00
2.01
2.01
1.02
1.09
2.02
3.00
9.0 8.5 8.0 7.5 7.0 6.5 6.0 5.5 5.0 4.5 4.0 3.5 3.0 2.5 2.0 1.5 1.0 0.5 0.0
f1 (ppm)

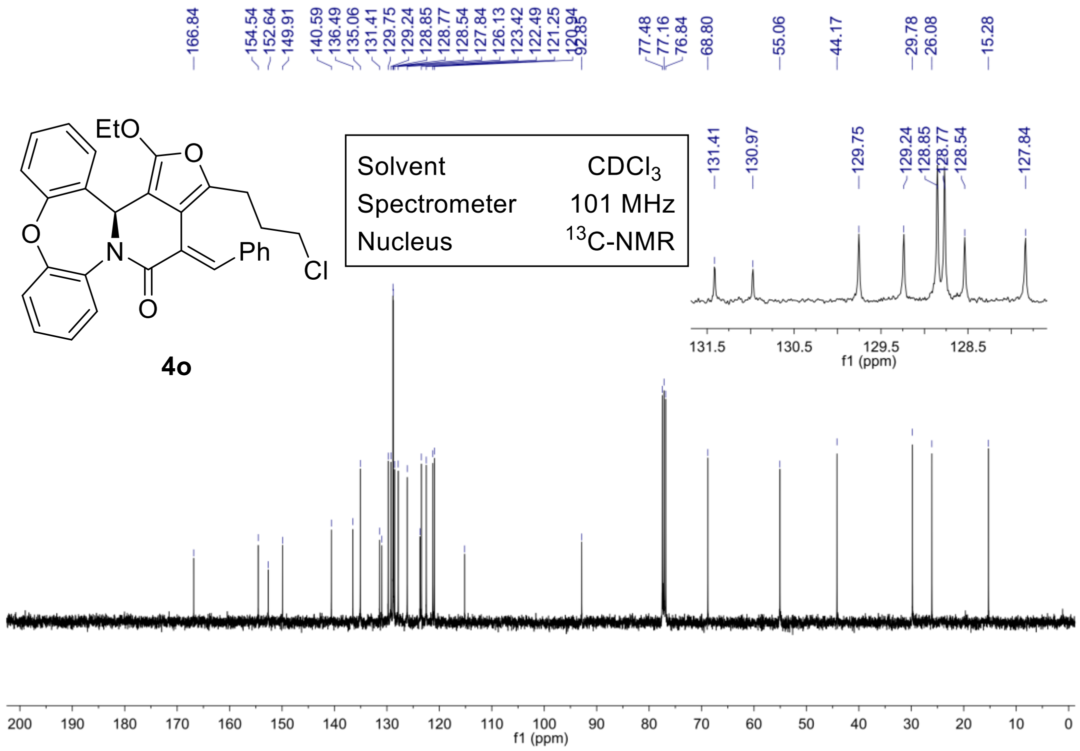
Solvent CDCl3
Spectrometer 101 MHz
Nucleus 13C-NMR
4o
f1 (ppm)

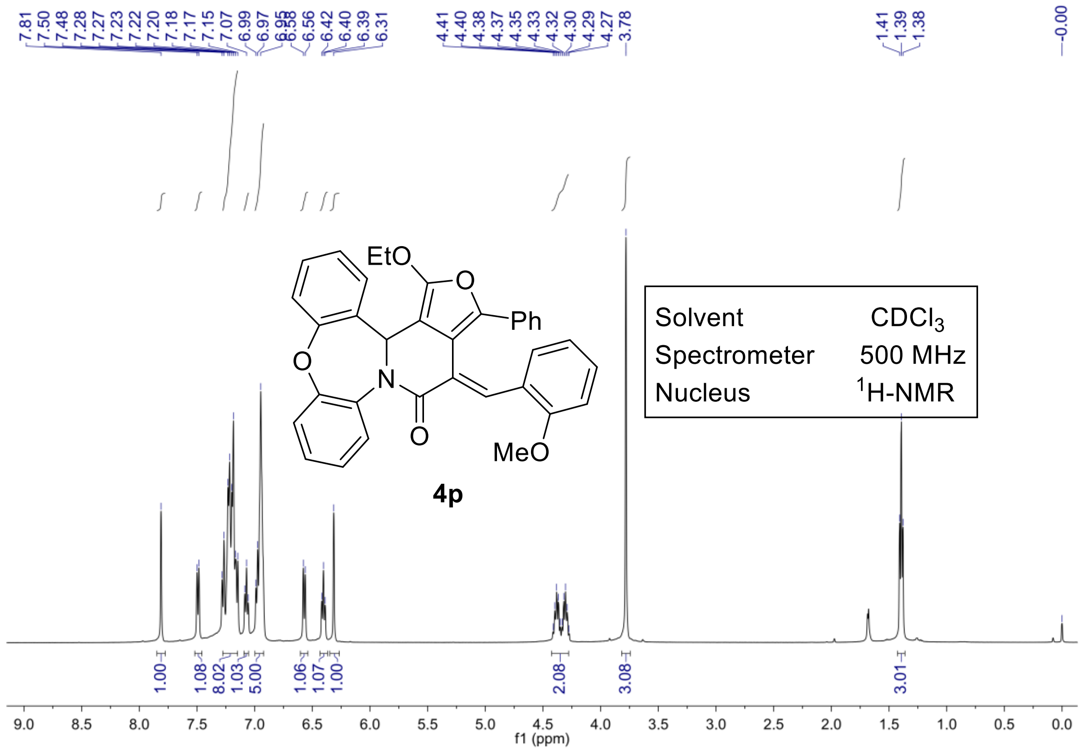
Solvent CDCl3
Spectrometer 500 MHz
Nucleus 1H-NMR
4p
f1 (ppm)

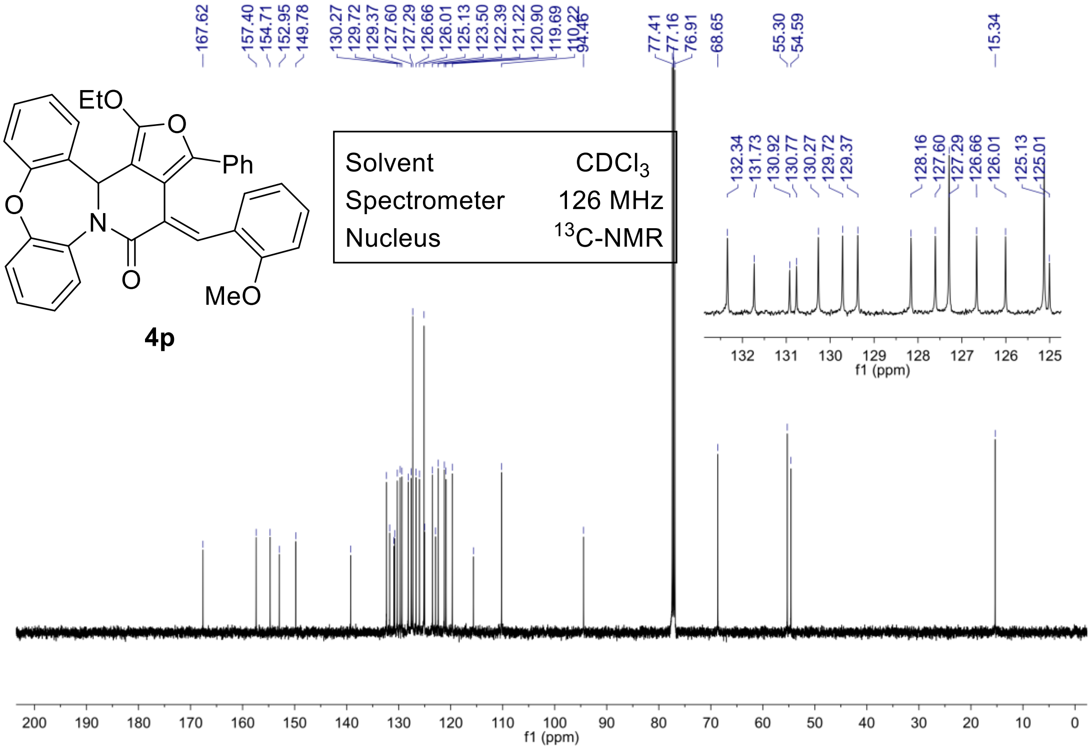
EtO
Ph
MeO
4p
Solvent CDCl$_3$
Spectrometer 126 MHz
Nucleus ^{13}C-NMR
f1 (ppm)

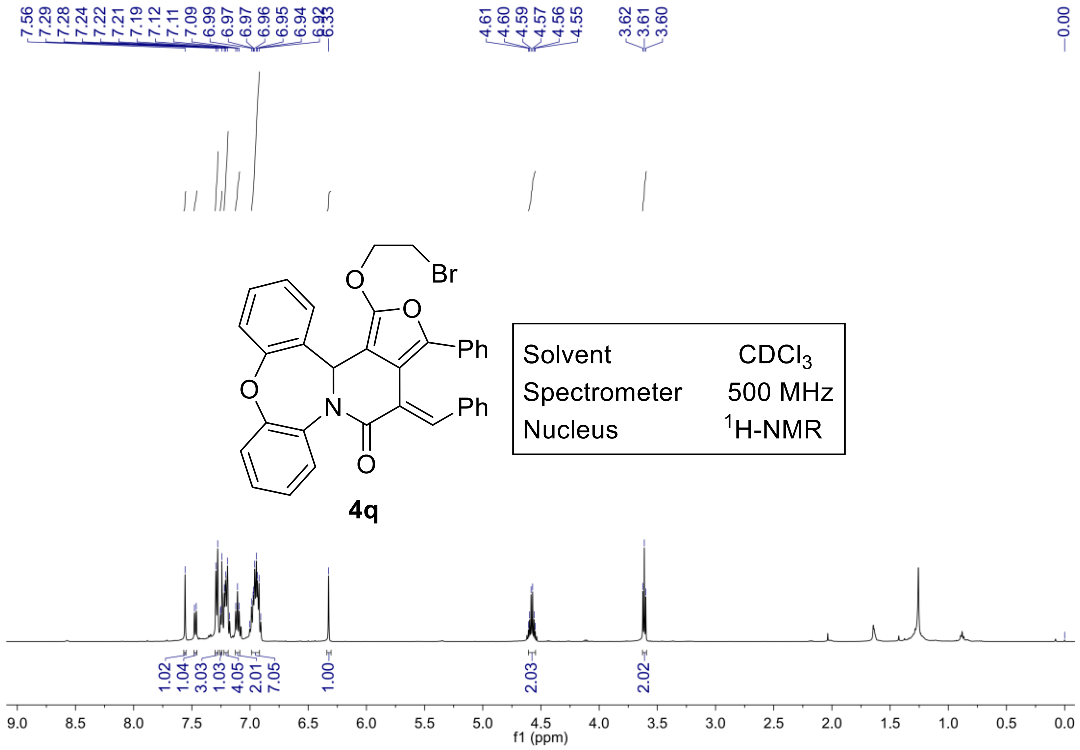
Br
Ph
Ph
4q
Solvent CDCl$_3$
Spectrometer 500 MHz
Nucleus ^{1}H-NMR
f1 (ppm)

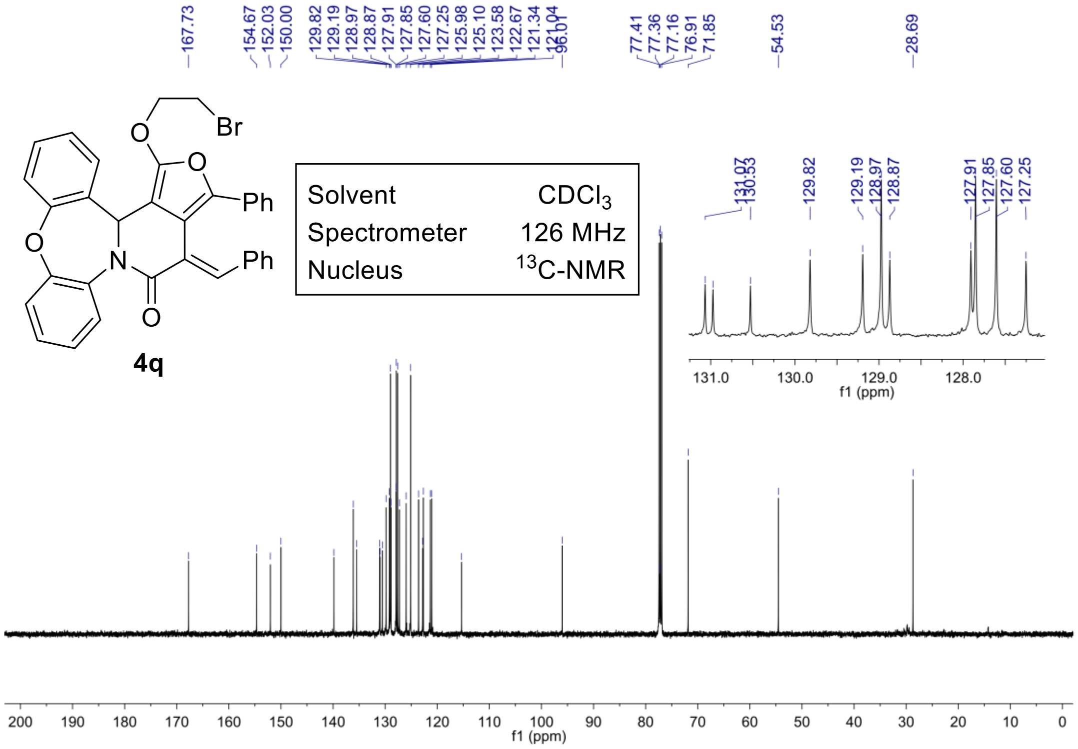
Br
O
Ph
Ph
4q
Solvent CDCl$_3$
Spectrometer 126 MHz
Nucleus ^{13}C-NMR
f1 (ppm)

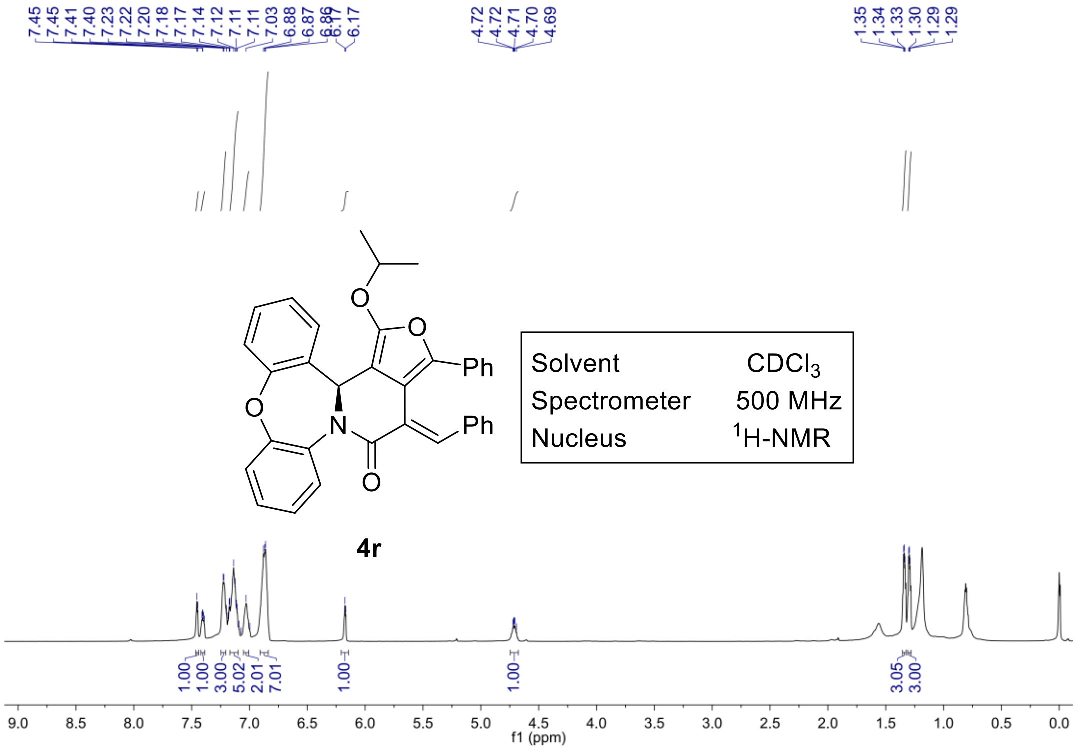
Ph
Ph
4r
Solvent CDCl$_3$
Spectrometer 500 MHz
Nucleus ^{1}H-NMR
f1 (ppm)

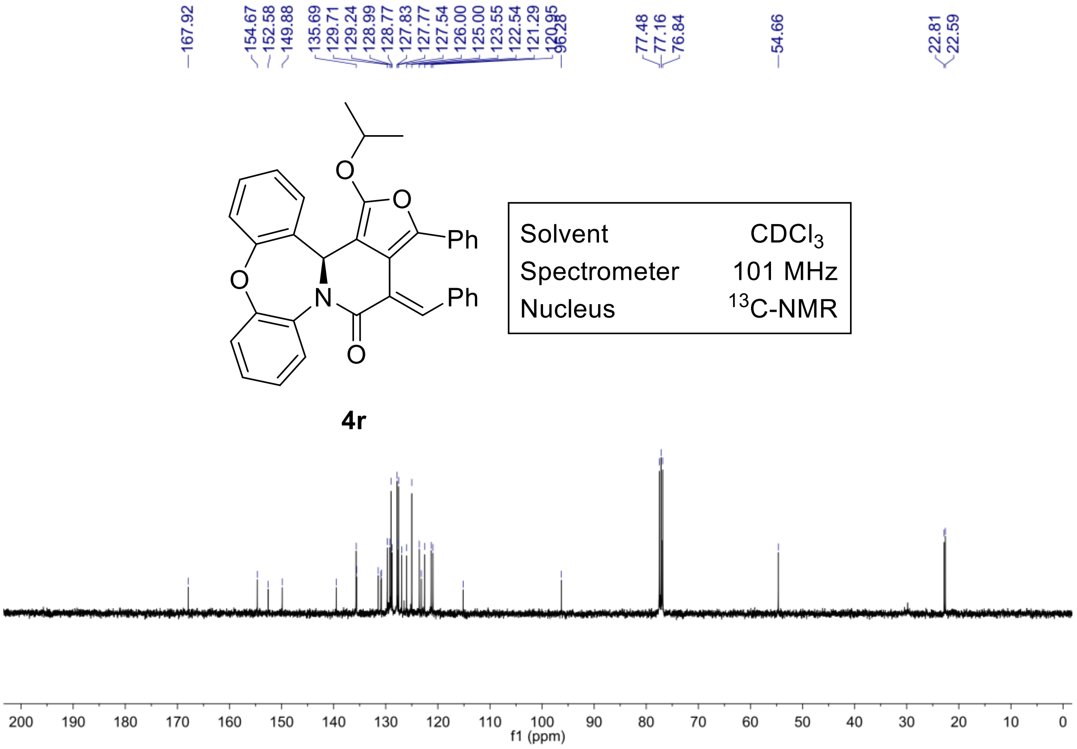
167.92
154.67
152.58
149.88
135.69
129.71
129.24
128.99
128.77
127.83
127.77
127.54
126.00
125.00
123.55
122.54
121.29
120.95
96.28
77.48
77.16
76.84
54.66
22.81
22.59
O
O
Ph
O
N
Ph
O
4r
Solvent CDCl3
Spectrometer 101 MHz
Nucleus 13C-NMR
200 190 180 170 160 150 140 130 120 110 100 90 80 70 60 50 40 30 20 10 0
f1 (ppm)

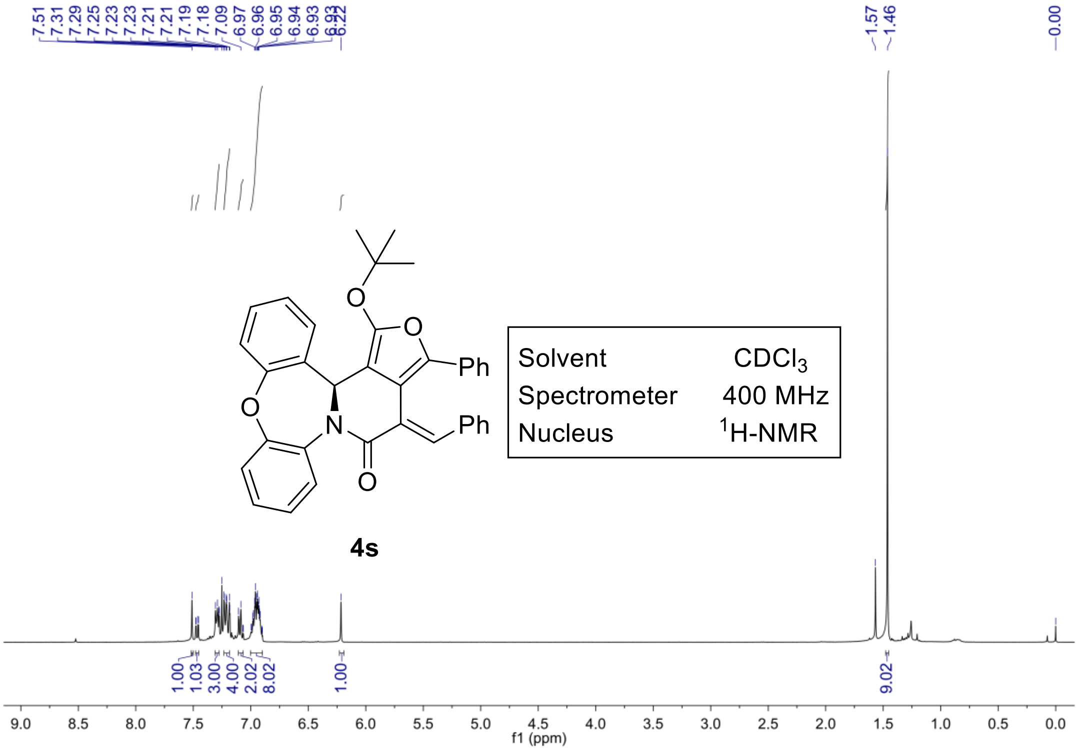
7.51
7.31
7.29
7.25
7.23
7.23
7.21
7.21
7.19
7.18
7.09
6.97
6.96
6.95
6.94
6.93
6.92
6.22
1.57
1.46
0.00
O
O
Ph
O
N
Ph
O
4s
Solvent CDCl3
Spectrometer 400 MHz
Nucleus 1H-NMR
1.00
1.03
3.00
4.00
2.02
8.02
1.00
9.02
9.0 8.5 8.0 7.5 7.0 6.5 6.0 5.5 5.0 4.5 4.0 3.5 3.0 2.5 2.0 1.5 1.0 0.5 0.0
f1 (ppm)

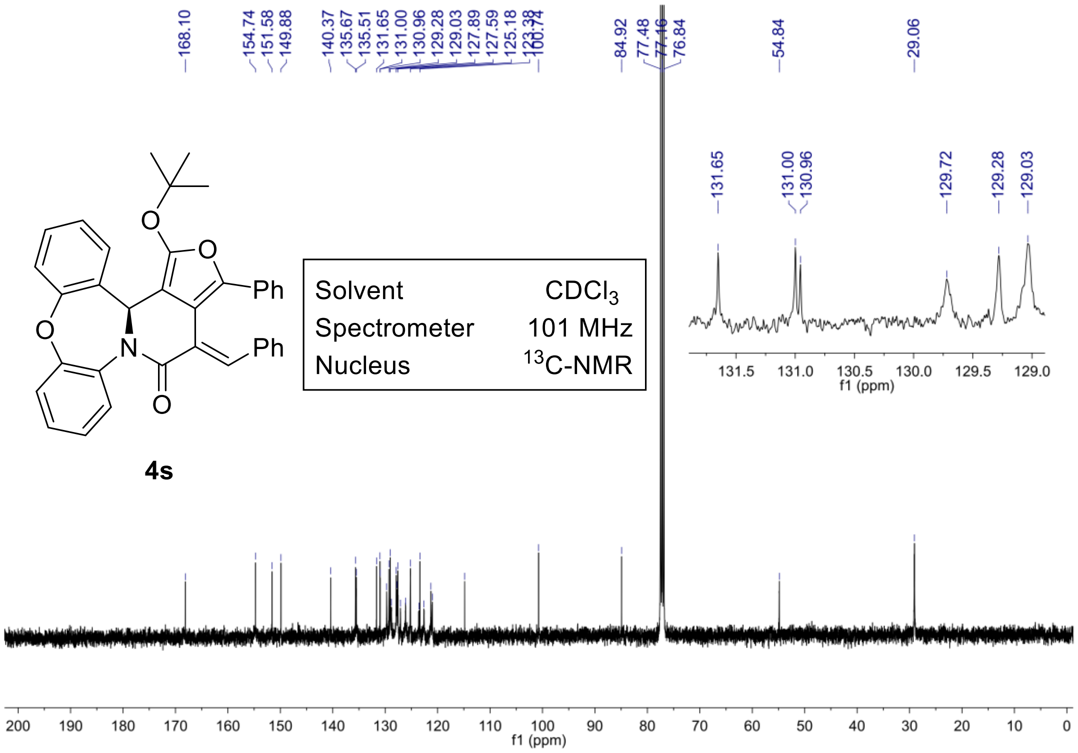
4s
Solvent CDCl$_3$
Spectrometer 101 MHz
Nucleus ^{13}C-NMR
168.10
154.74
151.58
149.88
140.37
135.67
135.51
131.65
131.00
130.96
129.28
129.03
127.89
127.59
125.18
100.74
84.92
77.48
76.84
54.84
29.06
129.72
f1 (ppm)

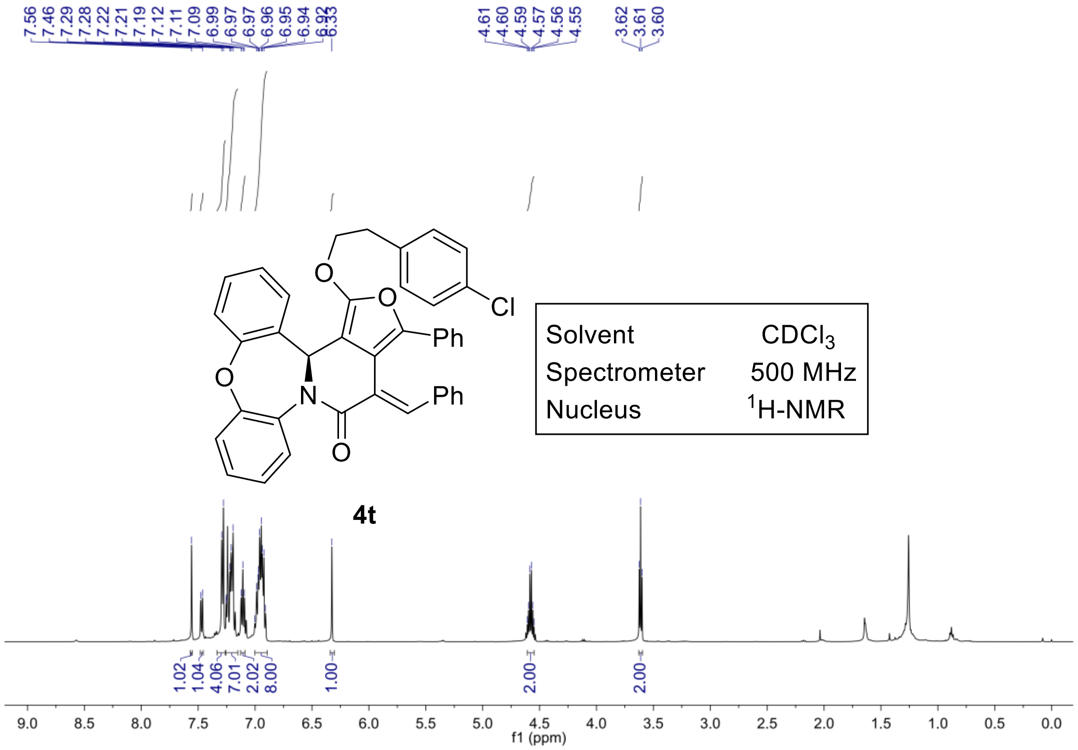
4t
Solvent CDCl$_3$
Spectrometer 500 MHz
Nucleus ^{1}H-NMR
7.56
7.46
7.29
7.28
7.22
7.21
7.19
7.12
7.11
7.09
6.99
6.97
6.97
6.96
6.95
6.94
6.33
4.61
4.60
4.59
4.57
4.56
4.55
3.62
3.61
3.60
1.02
1.04
4.06
7.01
2.02
8.00
1.00
2.00
2.00
f1 (ppm)

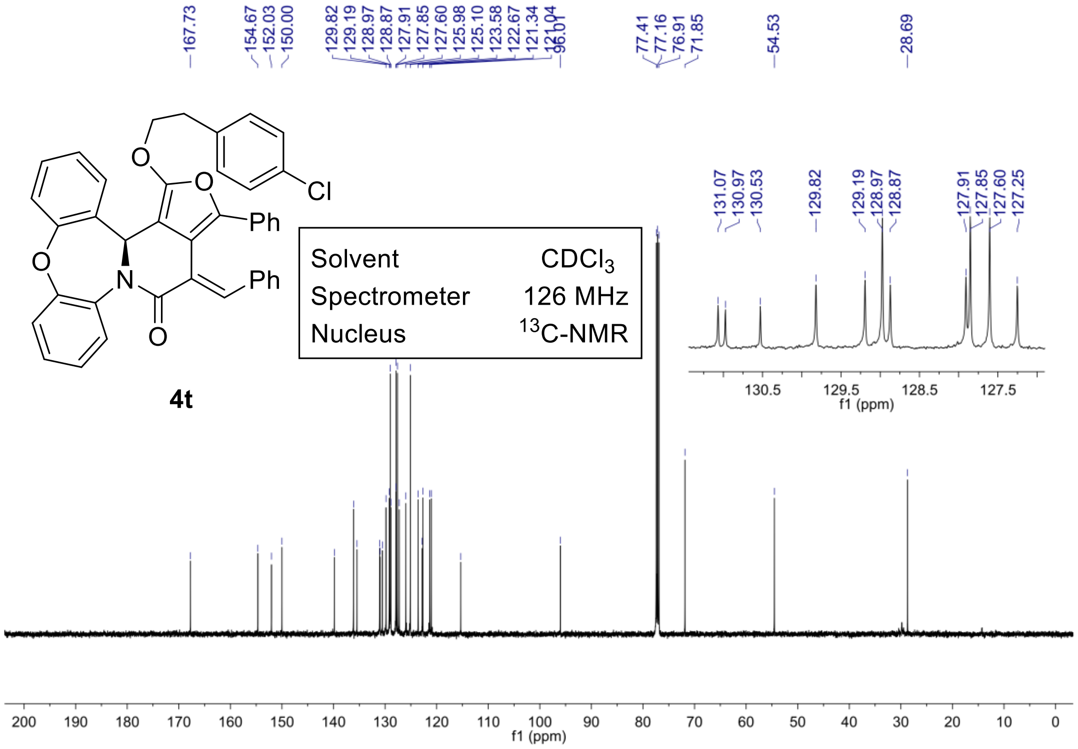
Solvent CDCl3
Spectrometer 126 MHz
Nucleus 13C-NMR
4t

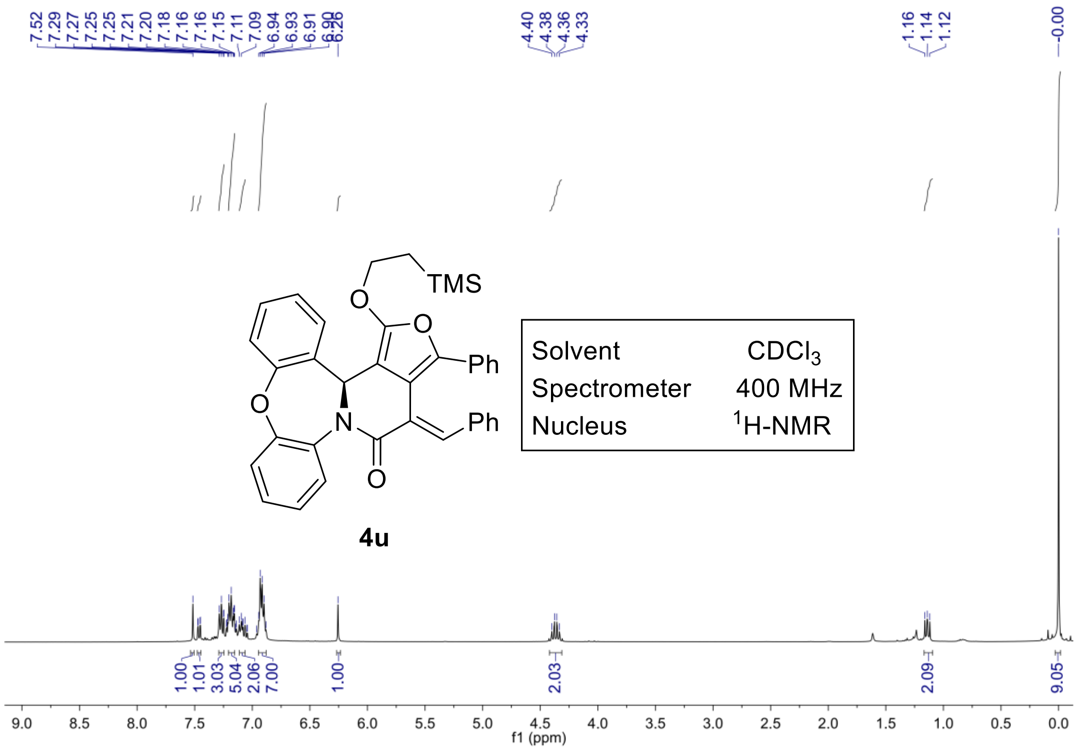
Solvent CDCl3
Spectrometer 400 MHz
Nucleus 1H-NMR
4u

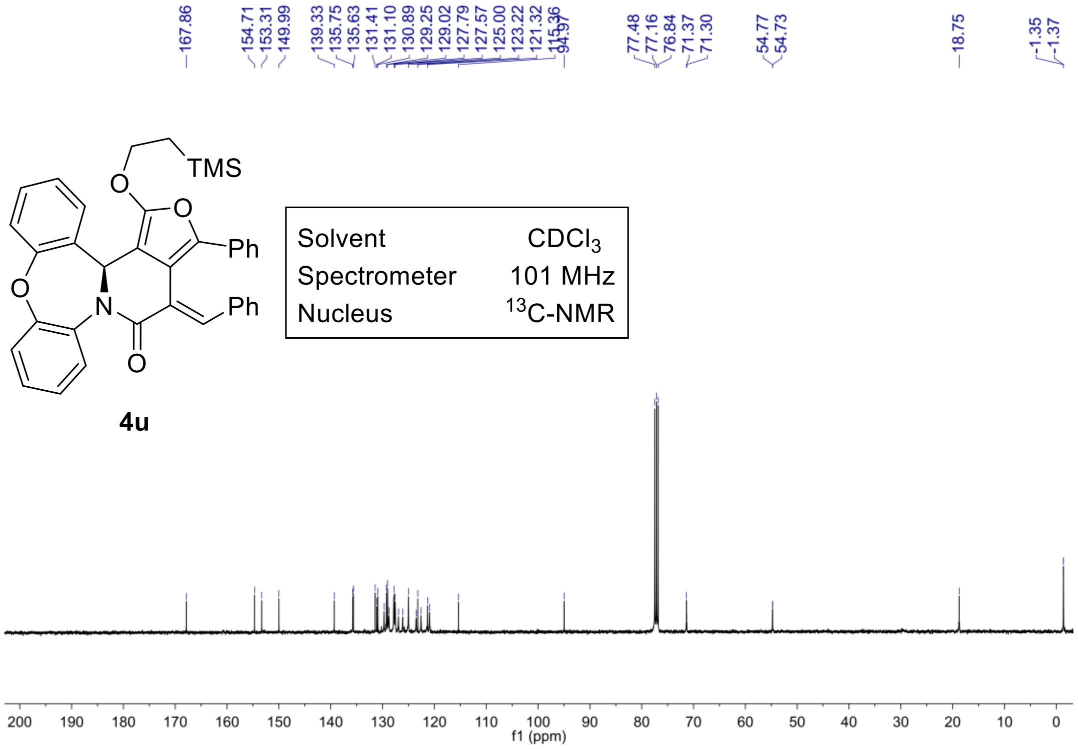
167.86
154.71
153.31
149.99
139.33
135.75
135.63
131.41
131.10
130.89
129.25
129.02
127.79
127.57
125.00
123.22
121.32
115.36
94.97
77.48
77.16
76.84
71.37
71.30
54.77
54.73
18.75
-1.35
-1.37
TMS
O
Ph
N
4u
Solvent CDCl3
Spectrometer 101 MHz
Nucleus 13C-NMR
f1 (ppm)

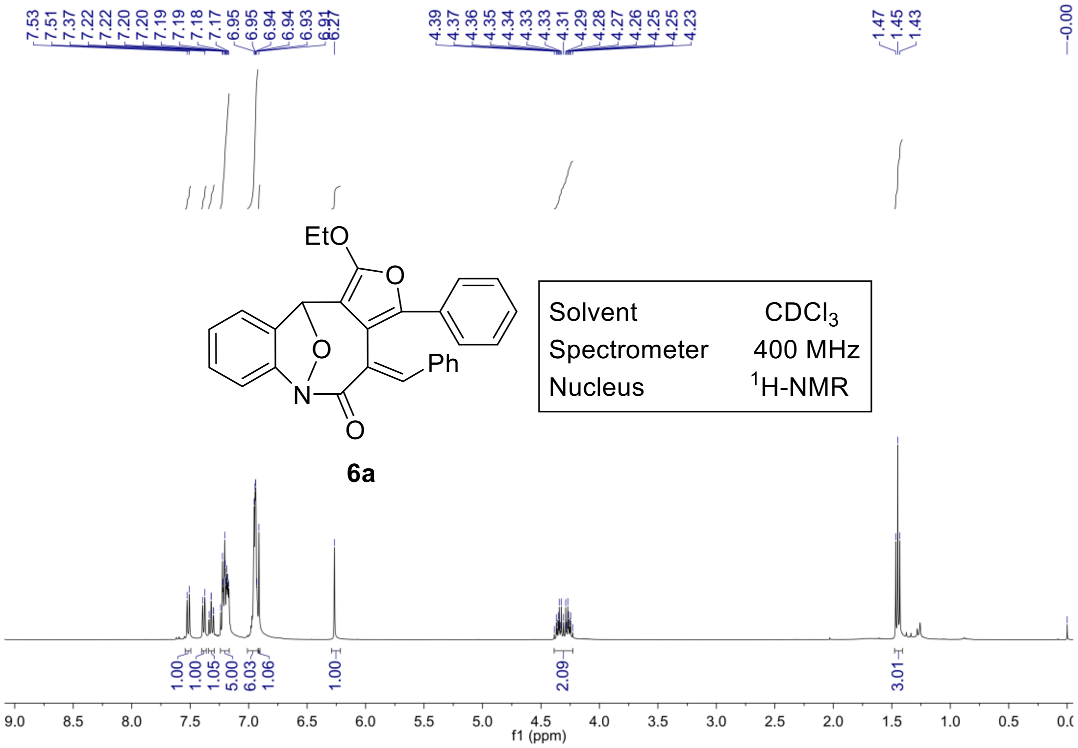
7.53
7.51
7.37
7.22
7.22
7.20
7.20
7.19
7.18
7.17
6.95
6.95
6.94
6.94
6.93
6.91
6.27
4.39
4.37
4.36
4.35
4.34
4.33
4.33
4.31
4.29
4.28
4.27
4.26
4.25
4.25
4.23
1.47
1.45
1.43
-0.00
EtO
O
Ph
N
6a
Solvent CDCl3
Spectrometer 400 MHz
Nucleus 1H-NMR
1.00
1.00
1.05
5.00
6.03
1.06
1.00
2.09
3.01
f1 (ppm)

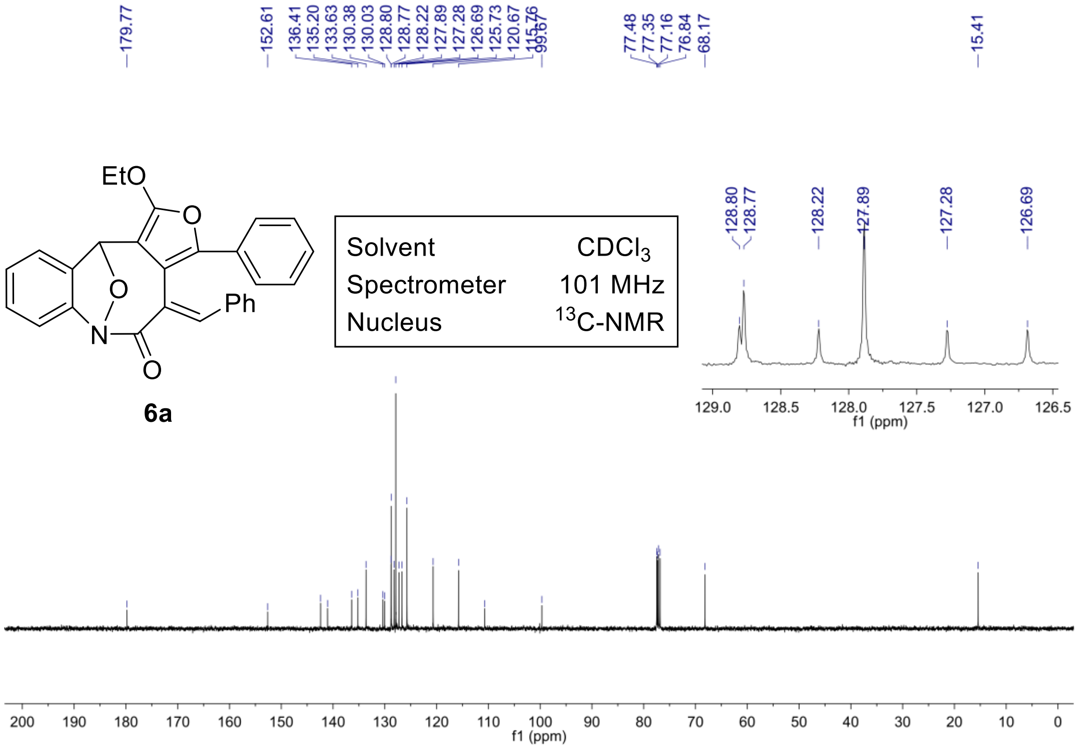
179.77
152.61
136.41
135.20
133.63
130.38
130.03
128.80
128.77
128.22
127.89
127.28
126.69
125.73
120.67
115.76
99.67
77.48
77.35
77.16
76.84
68.17
15.41
EtO
O
Ph
N
O
O
6a
Solvent CDCl$_3$
Spectrometer 101 MHz
Nucleus ^{13}C-NMR
f1 (ppm)

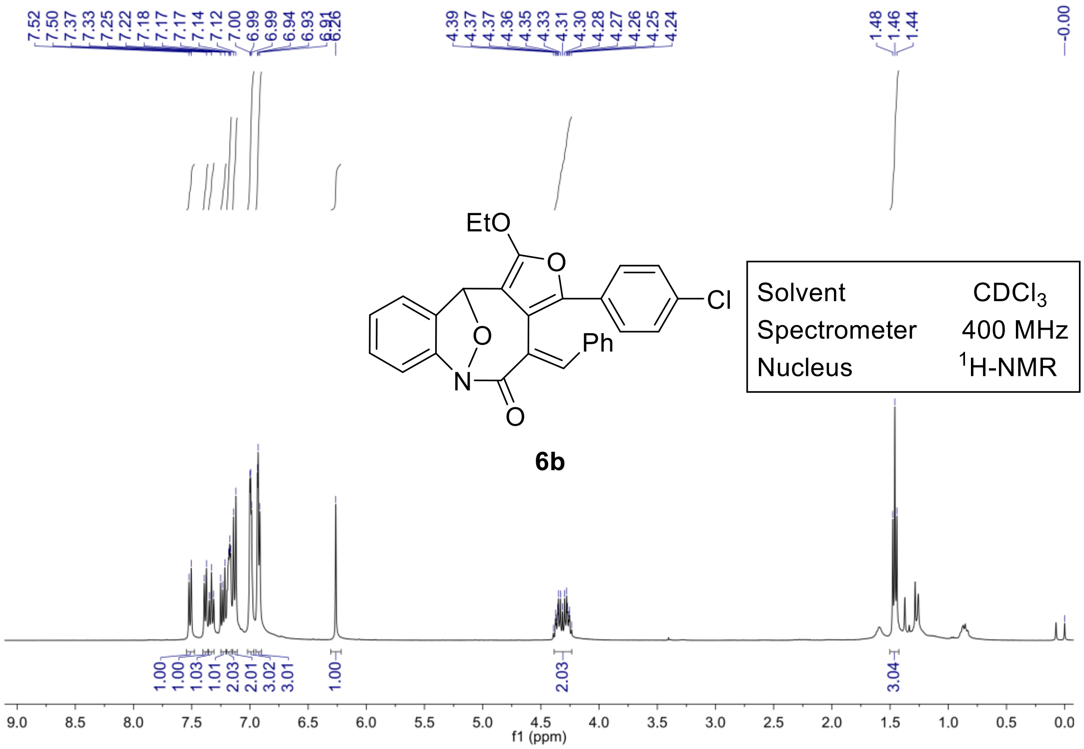
7.52
7.50
7.37
7.33
7.25
7.22
7.18
7.17
7.14
7.12
7.00
6.99
6.99
6.94
6.93
6.91
6.26
4.39
4.37
4.37
4.36
4.35
4.33
4.31
4.30
4.28
4.27
4.26
4.25
4.24
1.48
1.46
1.44
-0.00
EtO
O
Cl
Ph
N
O
O
6b
Solvent CDCl$_3$
Spectrometer 400 MHz
Nucleus ^{1}H-NMR
1.00
1.00
1.03
1.01
2.03
2.01
3.02
3.01
1.00
2.03
3.04
f1 (ppm)

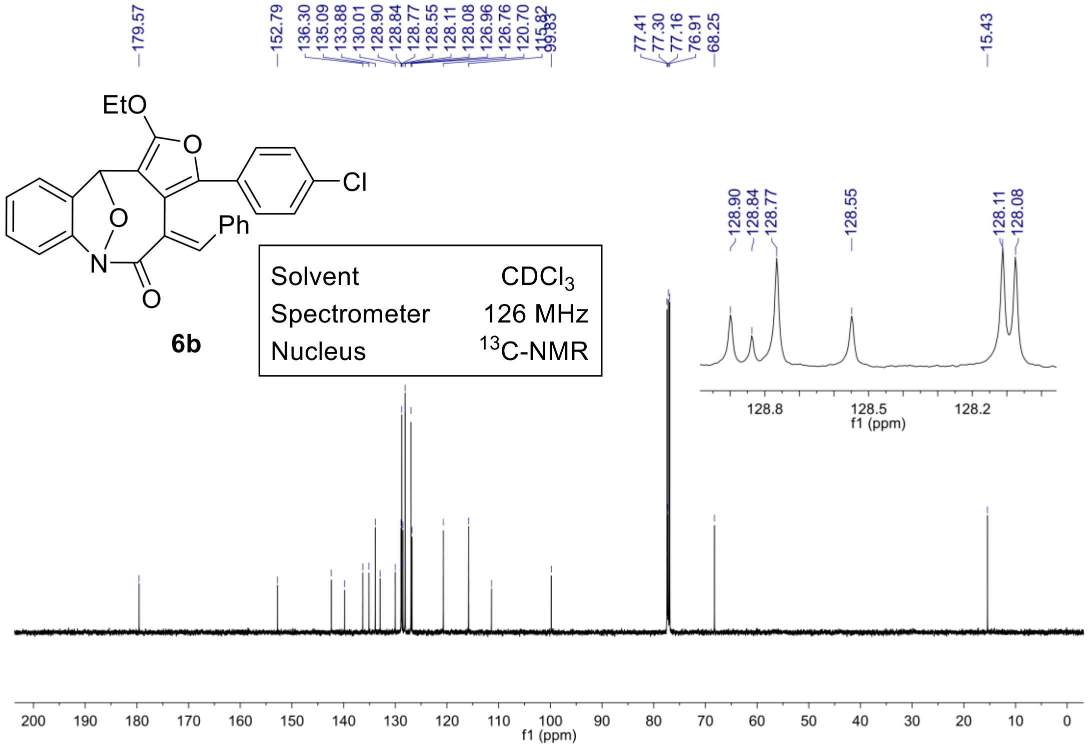
179.57
152.79
136.30
135.09
133.88
130.01
128.90
128.84
128.77
128.55
128.11
128.08
126.96
126.76
120.70
115.82
99.83
77.41
77.30
77.16
76.91
68.25
15.43
EtO
O
Cl
O
Ph
N
O
6b
Solvent CDCl3
Spectrometer 126 MHz
Nucleus 13C-NMR
128.90
128.84
128.77
128.55
128.11
128.08
128.8
128.5
128.2
f1 (ppm)
200 190 180 170 160 150 140 130 120 110 100 90 80 70 60 50 40 30 20 10 0
f1 (ppm)

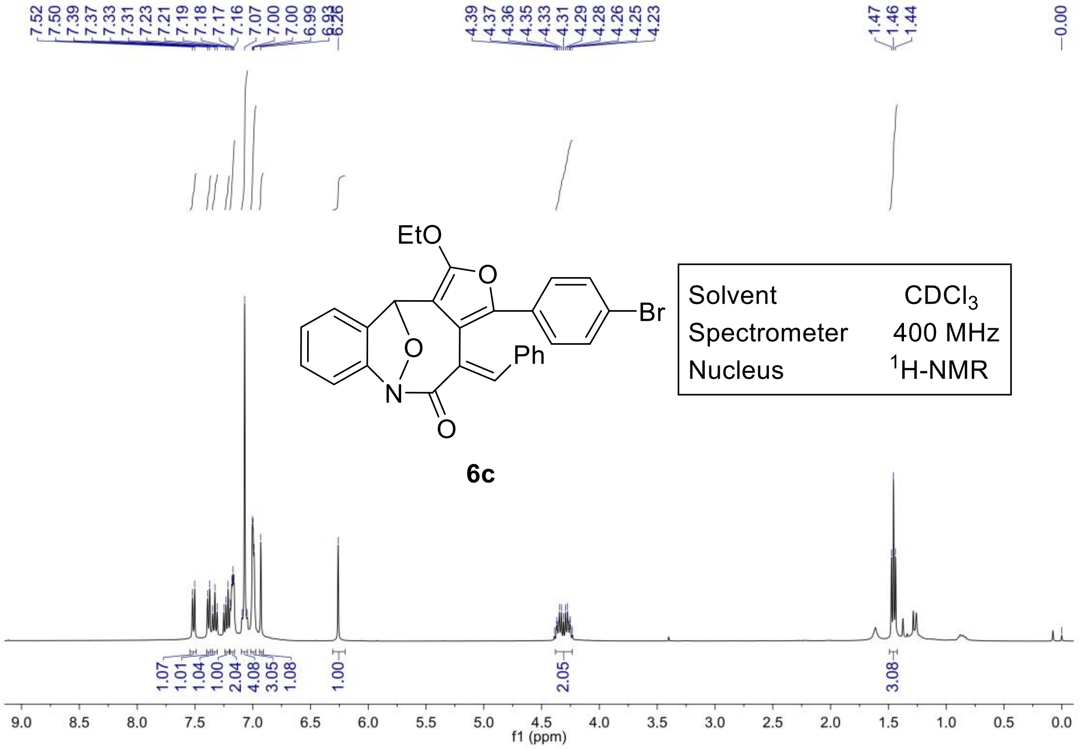
7.52
7.50
7.39
7.37
7.33
7.31
7.23
7.21
7.19
7.18
7.17
7.16
7.07
7.00
7.00
6.99
6.93
6.26
4.39
4.37
4.36
4.35
4.33
4.31
4.29
4.28
4.26
4.25
4.23
1.47
1.46
1.44
0.00
EtO
O
Br
O
Ph
N
O
6c
Solvent CDCl3
Spectrometer 400 MHz
Nucleus 1H-NMR
1.07
1.01
1.04
1.00
2.04
4.08
3.05
1.08
1.00
2.05
3.08
9.0 8.5 8.0 7.5 7.0 6.5 6.0 5.5 5.0 4.5 4.0 3.5 3.0 2.5 2.0 1.5 1.0 0.5 0.0
f1 (ppm)

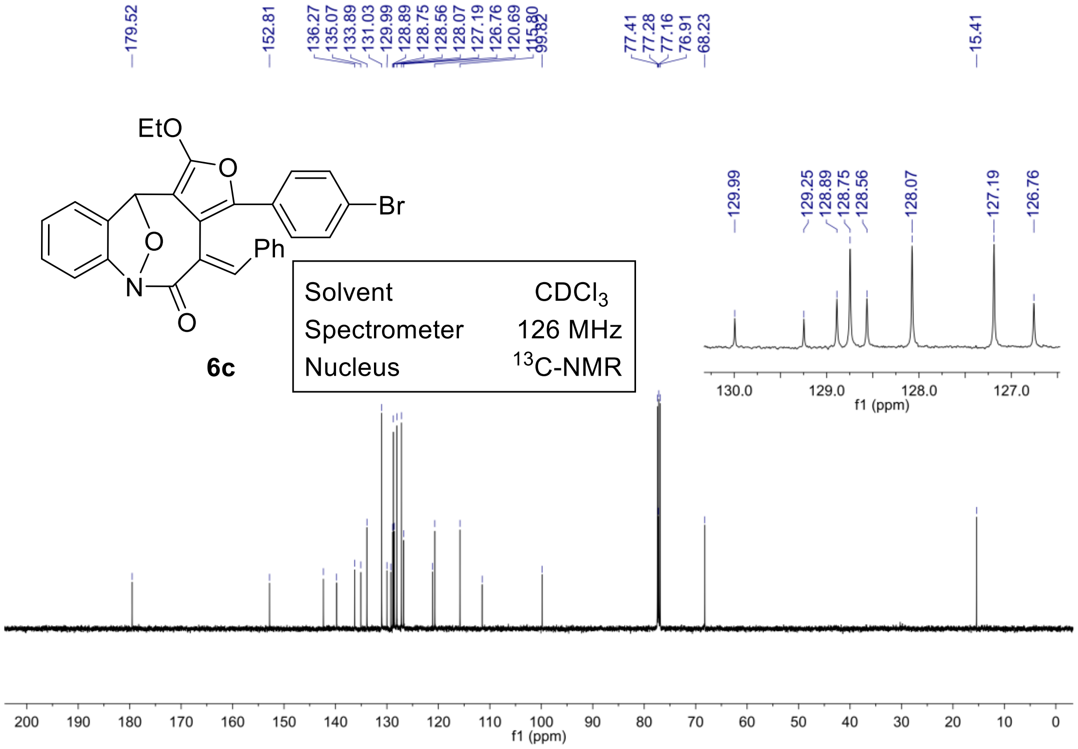
Solvent CDCl3
Spectrometer 126 MHz
Nucleus 13C-NMR
6c
f1 (ppm)

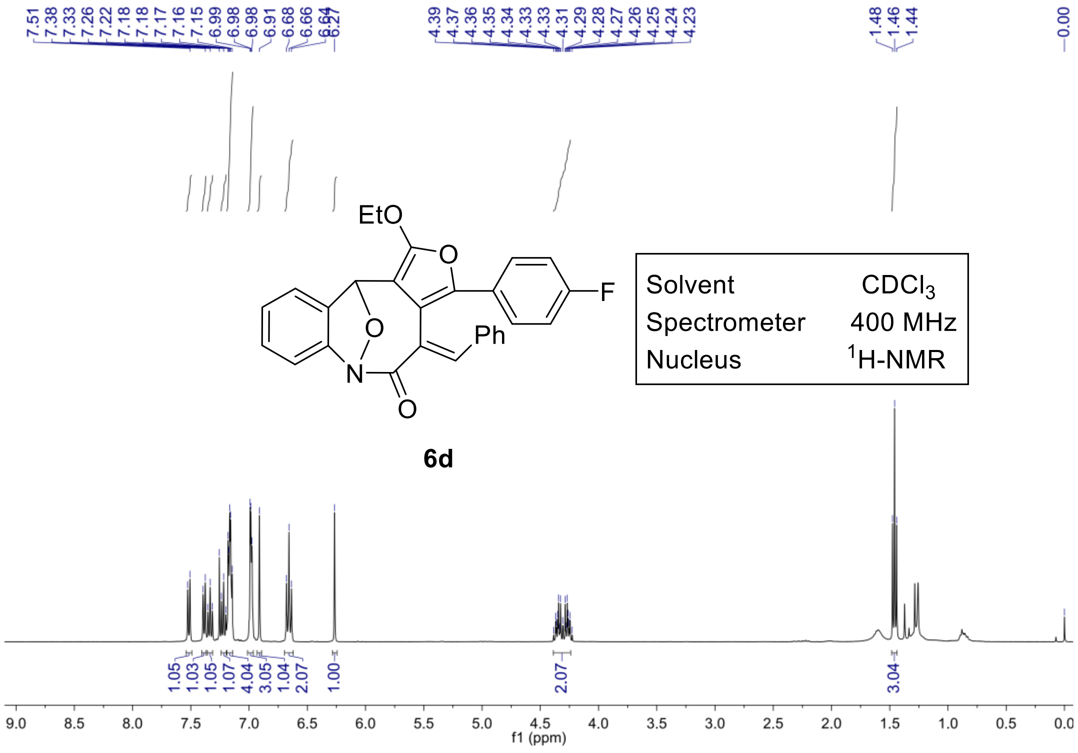
Solvent CDCl3
Spectrometer 400 MHz
Nucleus 1H-NMR
6d
f1 (ppm)

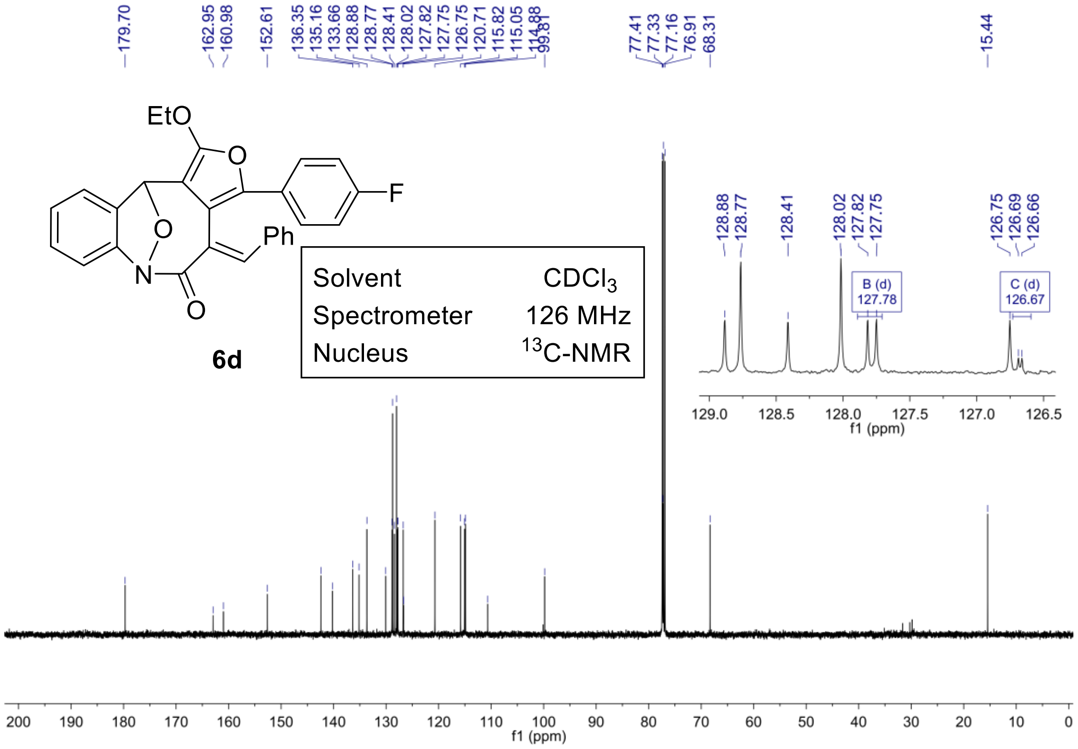
179.70
162.95
160.98
152.61
136.35
135.16
133.66
128.88
128.77
128.41
128.02
127.82
127.75
126.75
120.71
115.82
115.05
114.88
99.81
77.41
77.33
77.16
76.91
68.31
15.44
EtO
O
F
Ph
O
N
O
6d
Solvent CDCl3
Spectrometer 126 MHz
Nucleus 13C-NMR
B (d) 127.78
C (d) 126.67
126.69
126.66
f1 (ppm)

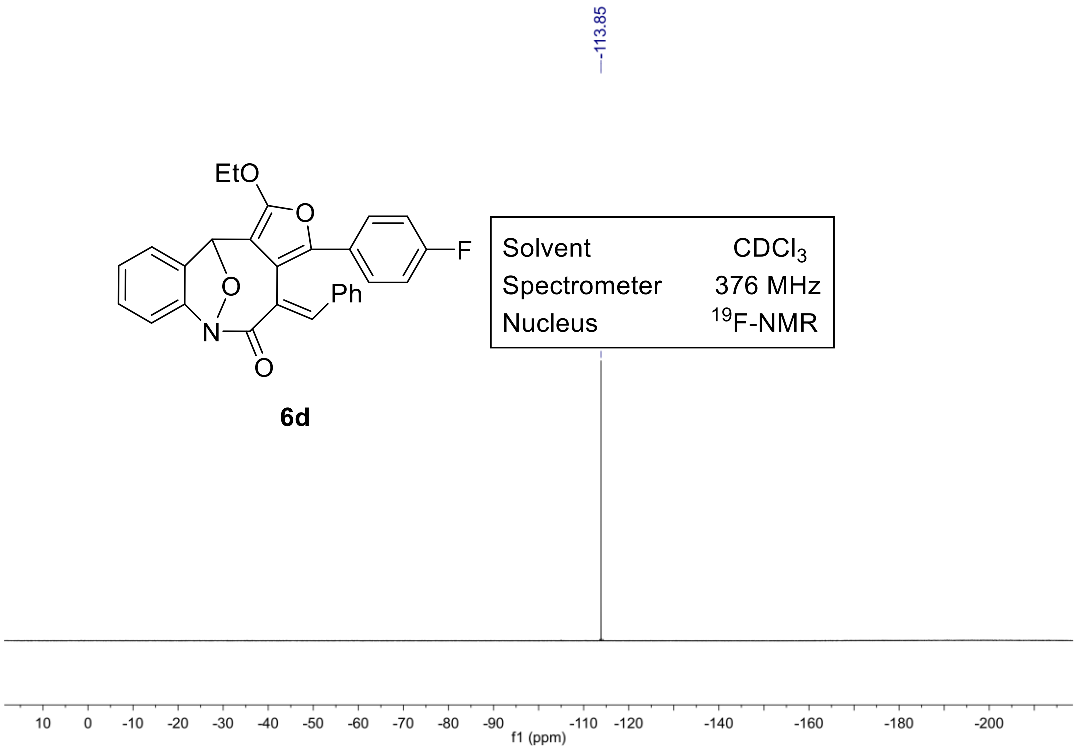
-113.85
EtO
O
F
Ph
O
N
O
6d
Solvent CDCl3
Spectrometer 376 MHz
Nucleus 19F-NMR
f1 (ppm)

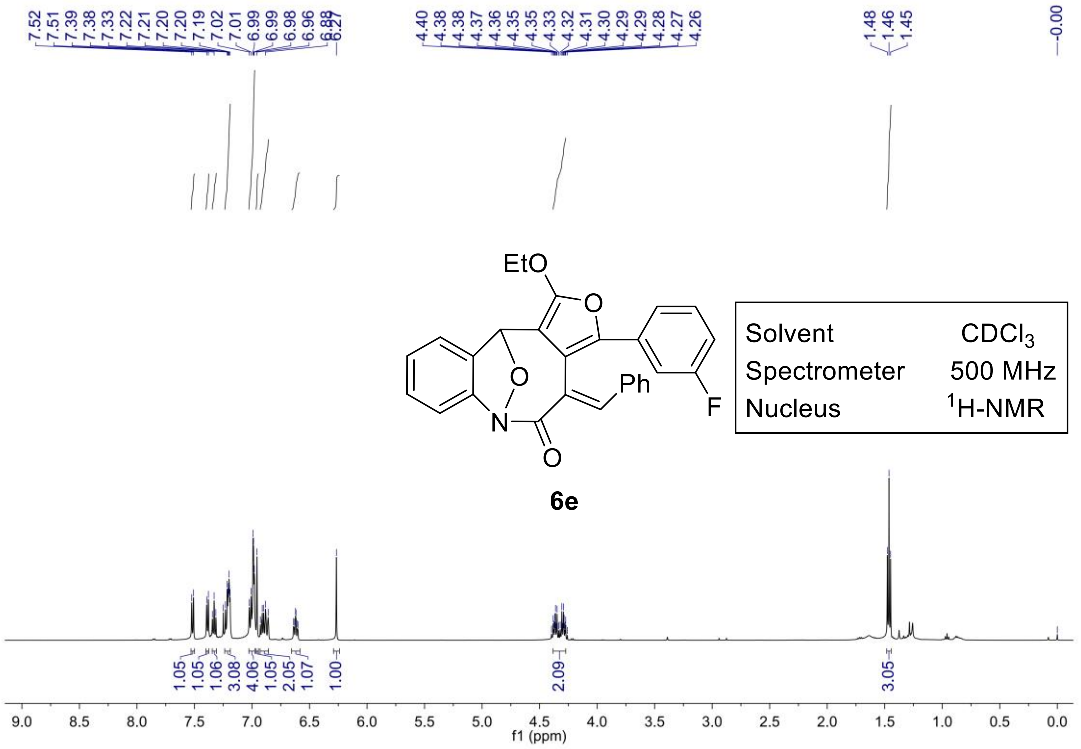
7.52
7.51
7.39
7.38
7.33
7.22
7.21
7.20
7.20
7.19
7.02
7.01
6.99
6.99
6.98
6.96
6.88
6.27
4.40
4.38
4.38
4.37
4.36
4.35
4.35
4.33
4.32
4.31
4.30
4.29
4.29
4.28
4.27
4.26
1.48
1.46
1.45
-0.00
EtO
O
O
Ph
F
N
O
6e
Solvent CDCl3
Spectrometer 500 MHz
Nucleus 1H-NMR
1.05
1.05
1.06
3.08
4.06
1.05
2.05
1.07
1.00
2.09
3.05
f1 (ppm)

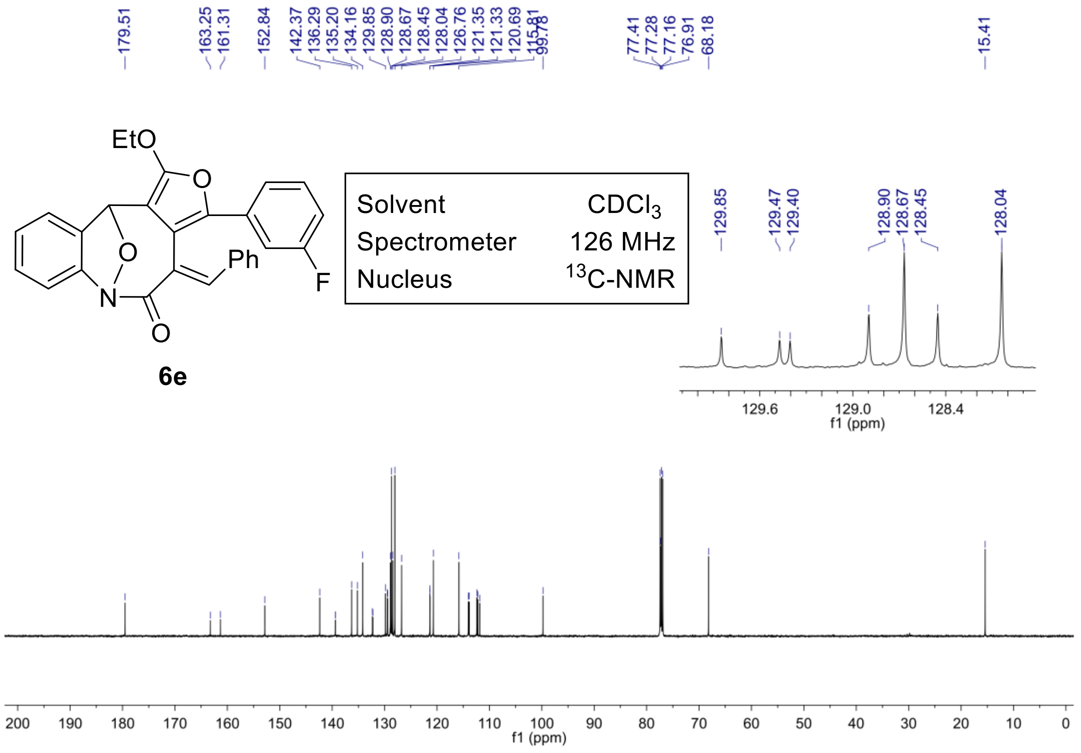
179.51
163.25
161.31
152.84
142.37
136.29
135.20
134.16
129.85
128.90
128.67
128.45
128.04
126.76
121.35
121.33
120.69
115.81
99.78
77.41
77.28
77.16
76.91
68.18
15.41
EtO
O
O
Ph
F
N
O
6e
Solvent CDCl3
Spectrometer 126 MHz
Nucleus 13C-NMR
129.85
129.47
129.40
128.90
128.67
128.45
128.04
f1 (ppm)
f1 (ppm)

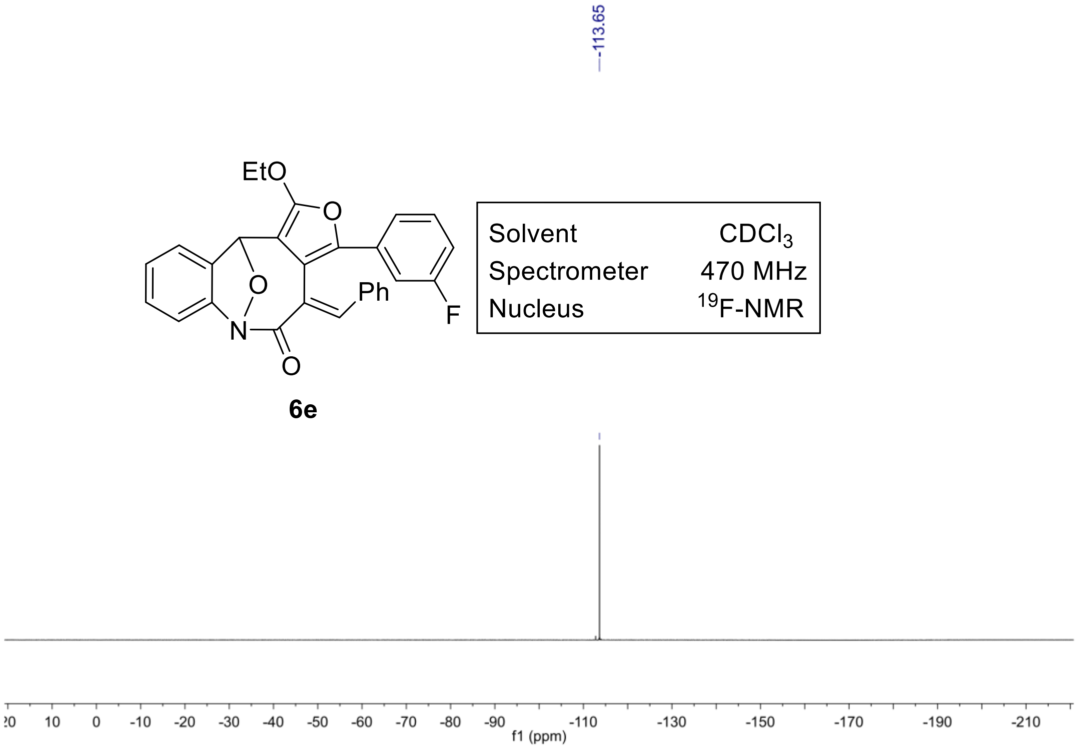
-113.65
EtO
O
O
N
O
Ph
F
6e
Solvent CDCl$_3$
Spectrometer 470 MHz
Nucleus ^{19}F-NMR
f1 (ppm)

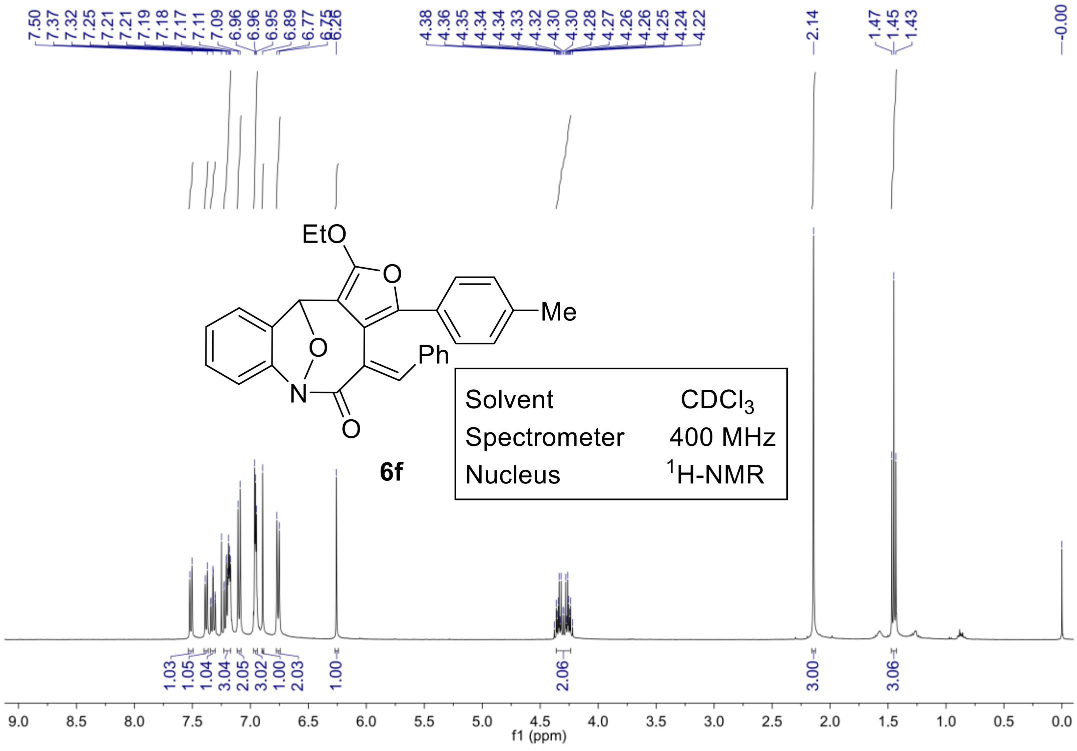
EtO
O
O
N
O
Ph
Me
6f
Solvent CDCl$_3$
Spectrometer 400 MHz
Nucleus ^{1}H-NMR
f1 (ppm)

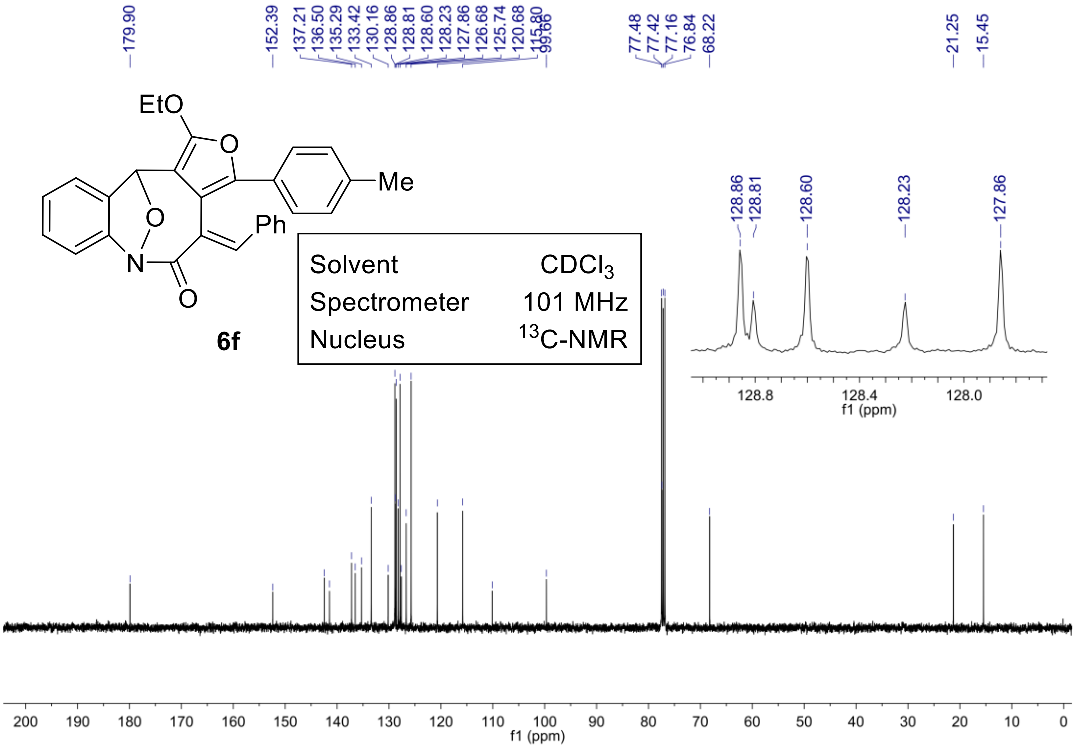
EtO
O
Me
O
Ph
N
O
6f
Solvent CDCl3
Spectrometer 101 MHz
Nucleus 13C-NMR
179.90
152.39
137.21
136.50
135.29
133.42
130.16
128.86
128.81
128.60
128.23
127.86
126.68
125.74
120.68
115.80
99.66
77.48
77.42
77.16
76.84
68.22
21.25
15.45
f1 (ppm)

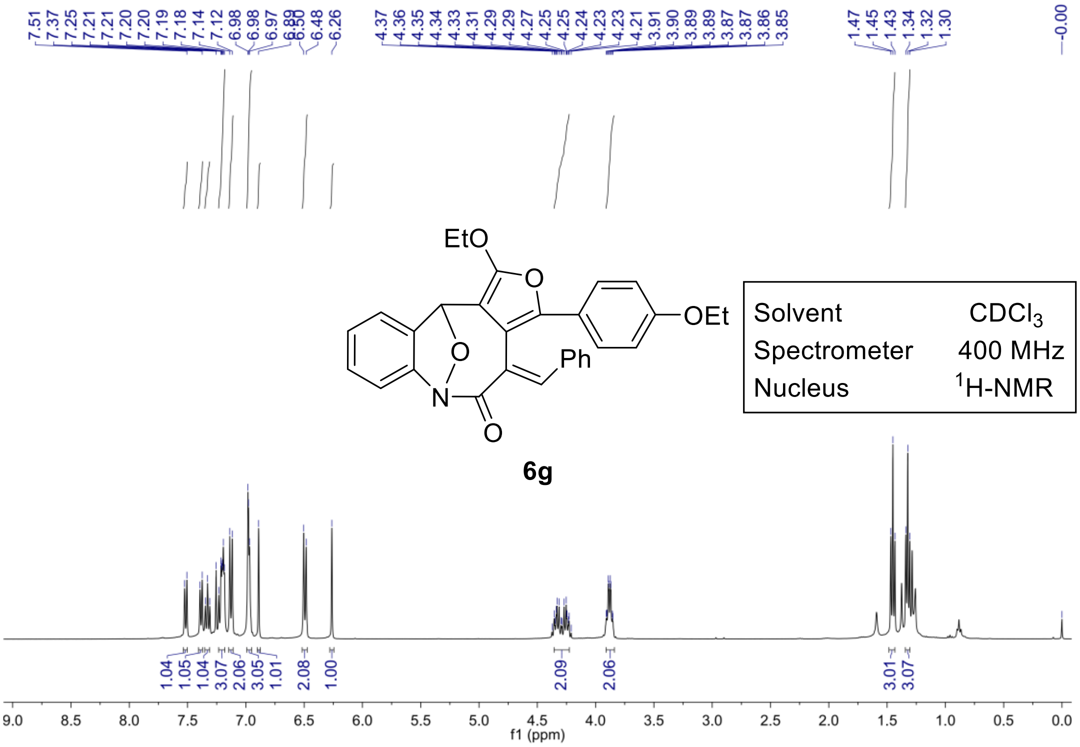
EtO
O
OEt
O
Ph
N
O
6g
Solvent CDCl3
Spectrometer 400 MHz
Nucleus 1H-NMR
7.51
7.37
7.25
7.21
7.21
7.20
7.20
7.19
7.18
7.14
7.12
6.98
6.98
6.97
6.89
6.50
6.48
6.26
4.37
4.36
4.35
4.34
4.33
4.31
4.29
4.29
4.27
4.25
4.25
4.24
4.23
4.23
4.21
3.91
3.90
3.89
3.89
3.87
3.87
3.86
3.85
1.47
1.45
1.43
1.34
1.32
1.30
-0.00
1.04
1.05
1.04
3.07
2.06
3.05
1.01
2.08
1.00
2.09
2.06
3.01
3.07
f1 (ppm)

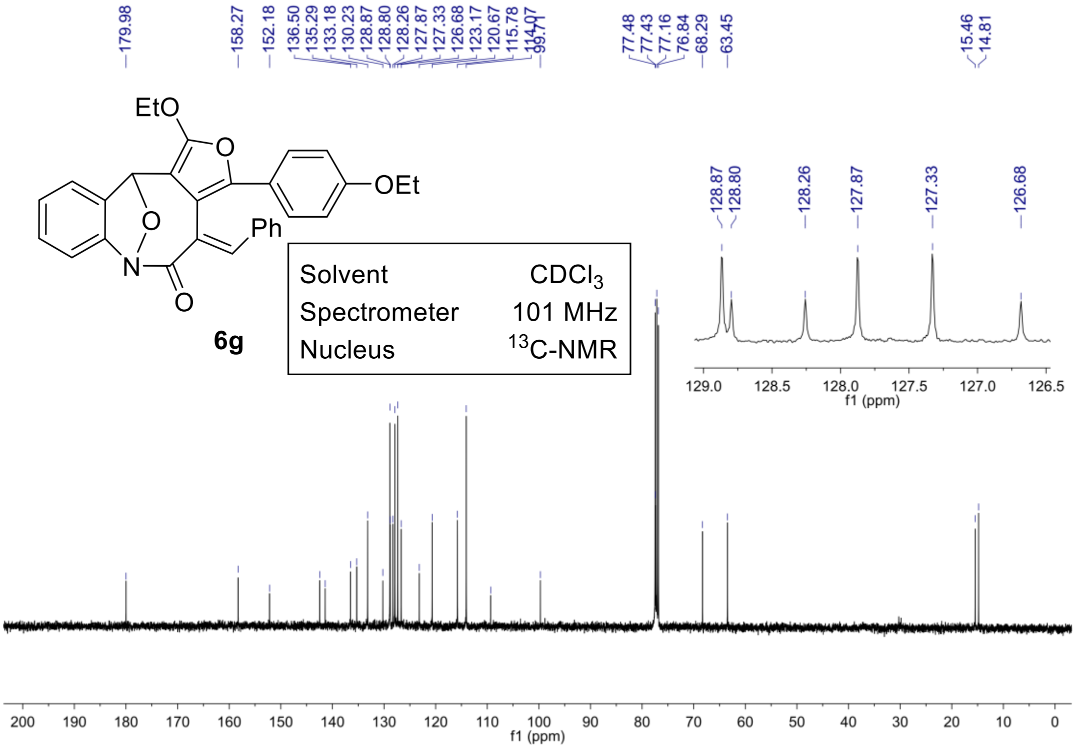
179.98
158.27
152.18
136.50
135.29
133.18
130.23
128.87
128.80
128.26
127.87
127.33
126.68
123.17
120.67
115.78
114.07
99.71
77.48
77.43
77.16
76.84
68.29
63.45
15.46
14.81
EtO
O
OEt
O
Ph
N
O
6g
Solvent CDCl3
Spectrometer 101 MHz
Nucleus 13C-NMR
f1 (ppm)

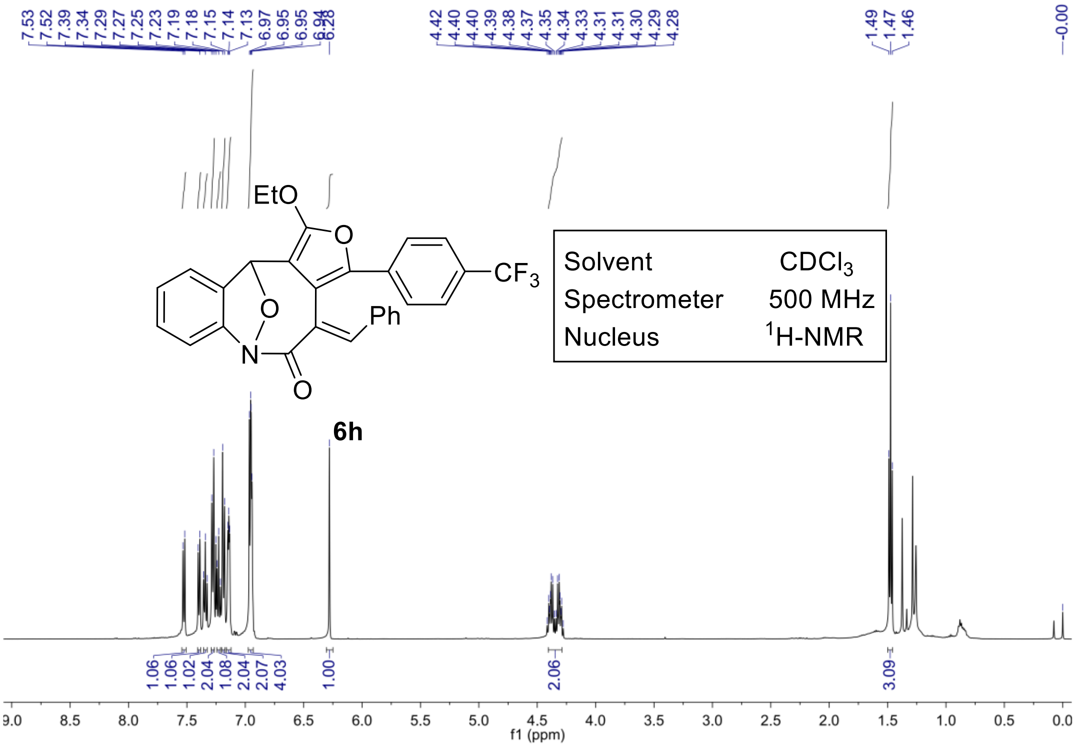
7.53
7.52
7.39
7.34
7.29
7.27
7.25
7.23
7.19
7.18
7.15
7.14
7.13
6.97
6.95
6.95
6.94
6.28
4.42
4.40
4.40
4.39
4.38
4.37
4.35
4.34
4.33
4.31
4.31
4.30
4.29
4.28
1.49
1.47
1.46
-0.00
EtO
O
CF3
O
Ph
N
O
6h
Solvent CDCl3
Spectrometer 500 MHz
Nucleus 1H-NMR
1.06
1.06
1.02
2.04
1.08
2.04
2.07
4.03
1.00
2.06
3.09
f1 (ppm)

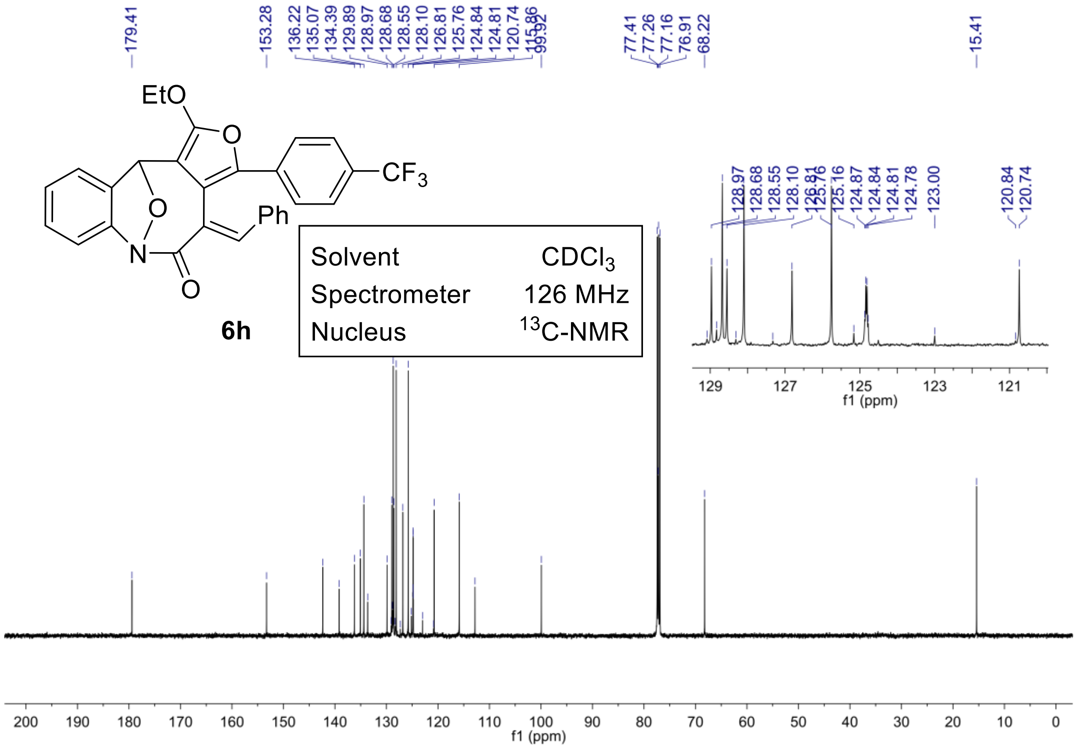
EtO
O
CF3
Ph
N
6h
Solvent CDCl3
Spectrometer 126 MHz
Nucleus 13C-NMR
f1 (ppm)

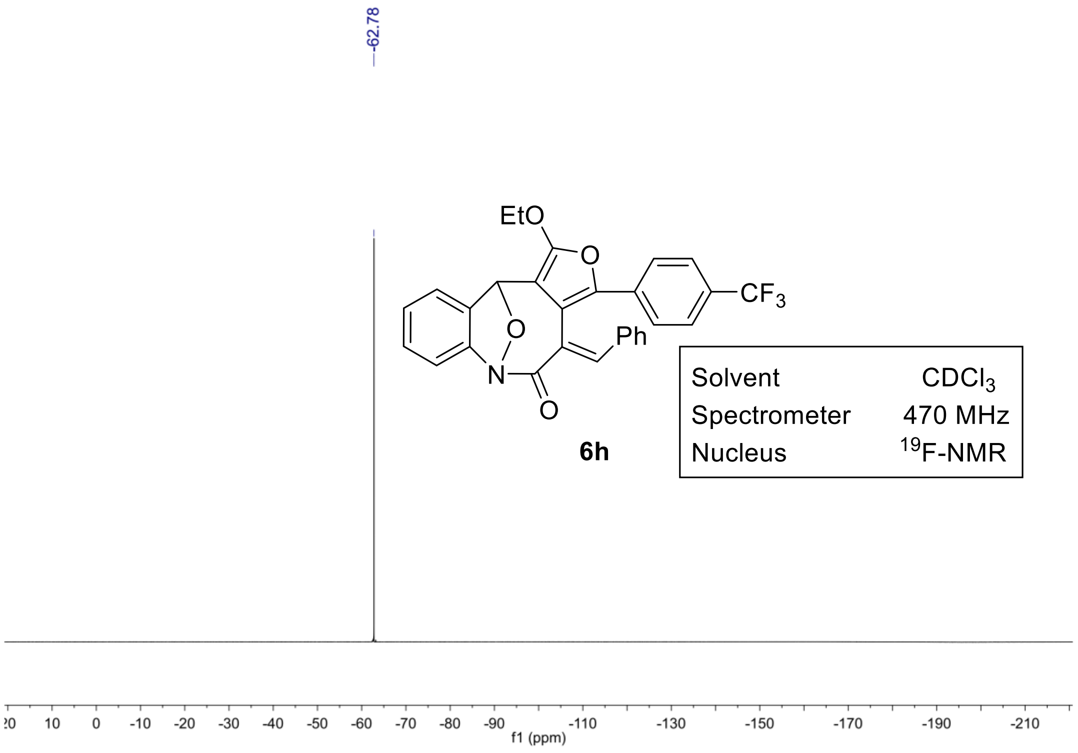
-62.78
EtO
O
CF3
Ph
N
6h
Solvent CDCl3
Spectrometer 470 MHz
Nucleus 19F-NMR
f1 (ppm)

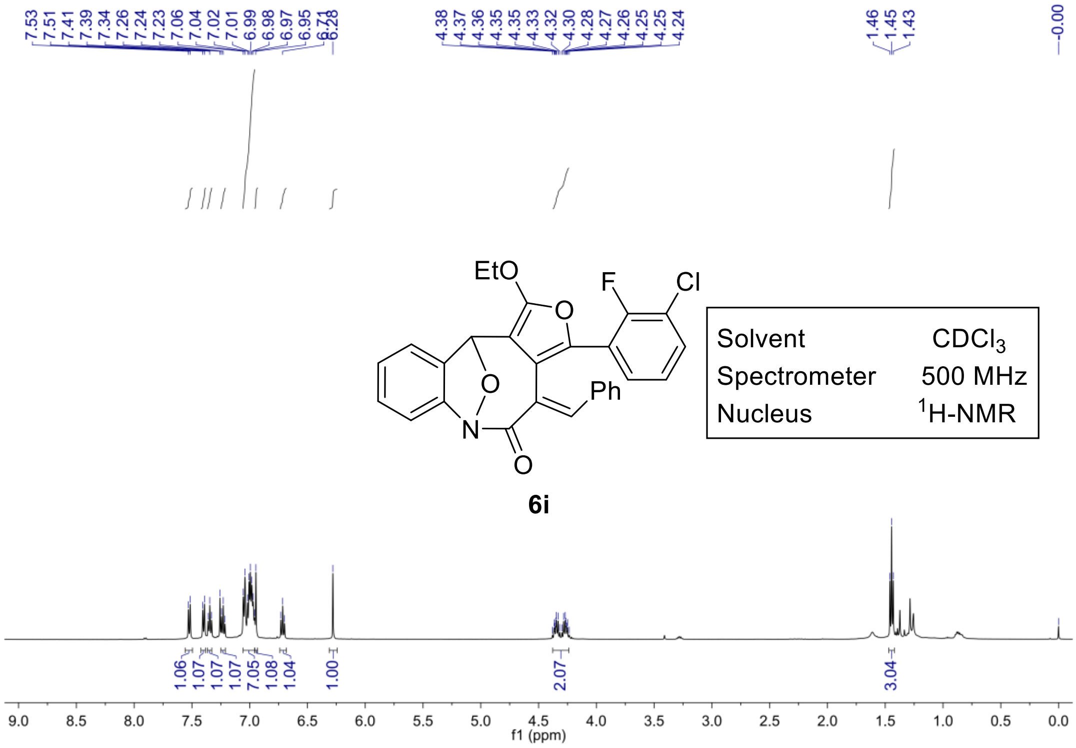
7.53 7.51 7.41 7.39 7.34 7.26 7.24 7.23 7.06 7.04 7.02 7.01 6.99 6.98 6.97 6.95 6.74 6.28
4.38 4.37 4.36 4.35 4.35 4.33 4.32 4.30 4.28 4.27 4.26 4.25 4.25 4.24
1.46 1.45 1.43
0.00
EtO
F
Cl
O
Ph
O
N
O
6i
Solvent CDCl$_3$
Spectrometer 500 MHz
Nucleus ^{1}H-NMR
1.06 1.07 1.07 1.07 7.05 1.08 1.04 1.00 2.07 3.04
9.0 8.5 8.0 7.5 7.0 6.5 6.0 5.5 5.0 4.5 4.0 3.5 3.0 2.5 2.0 1.5 1.0 0.5 0.0
f1 (ppm)

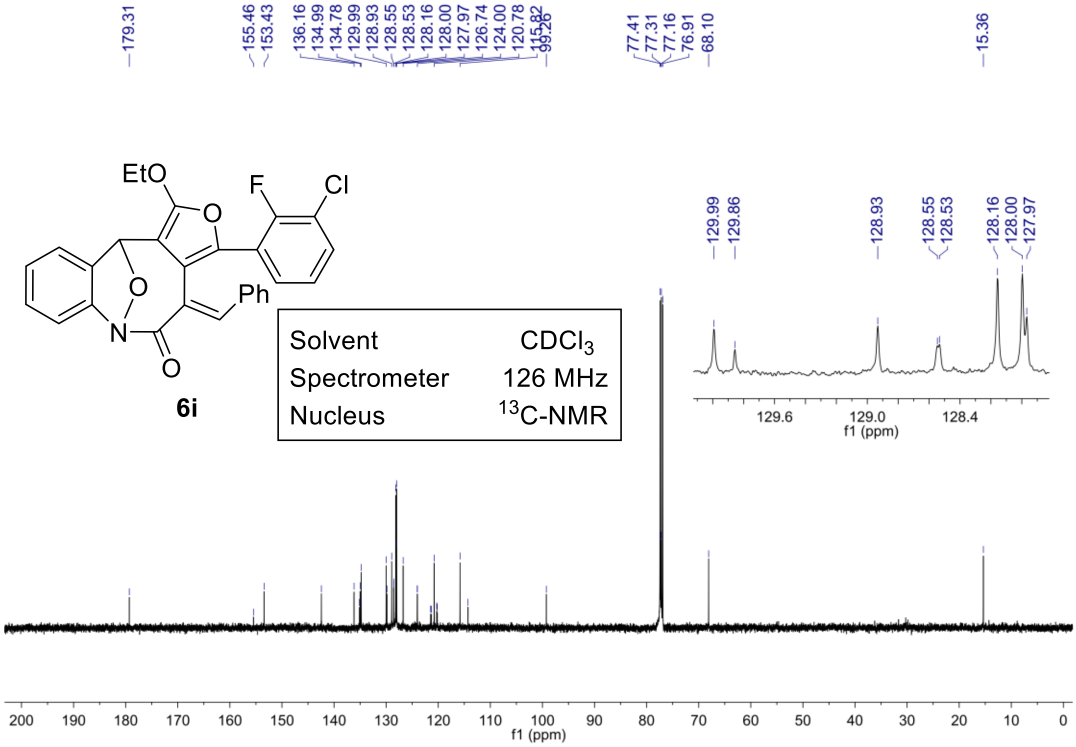
179.31
155.46 153.43
136.16 134.99 134.78 129.99 128.93 128.55 128.53 128.16 128.00 127.97 126.74 124.00 120.78 115.82 99.26
77.41 77.31 77.16 76.91 68.10
15.36
EtO
F
Cl
O
Ph
O
N
O
6i
Solvent CDCl$_3$
Spectrometer 126 MHz
Nucleus ^{13}C-NMR
129.99 129.86 128.93 128.55 128.53 128.16 128.00 127.97
129.6 129.0 128.4
f1 (ppm)
200 190 180 170 160 150 140 130 120 110 100 90 80 70 60 50 40 30 20 10 0
f1 (ppm)

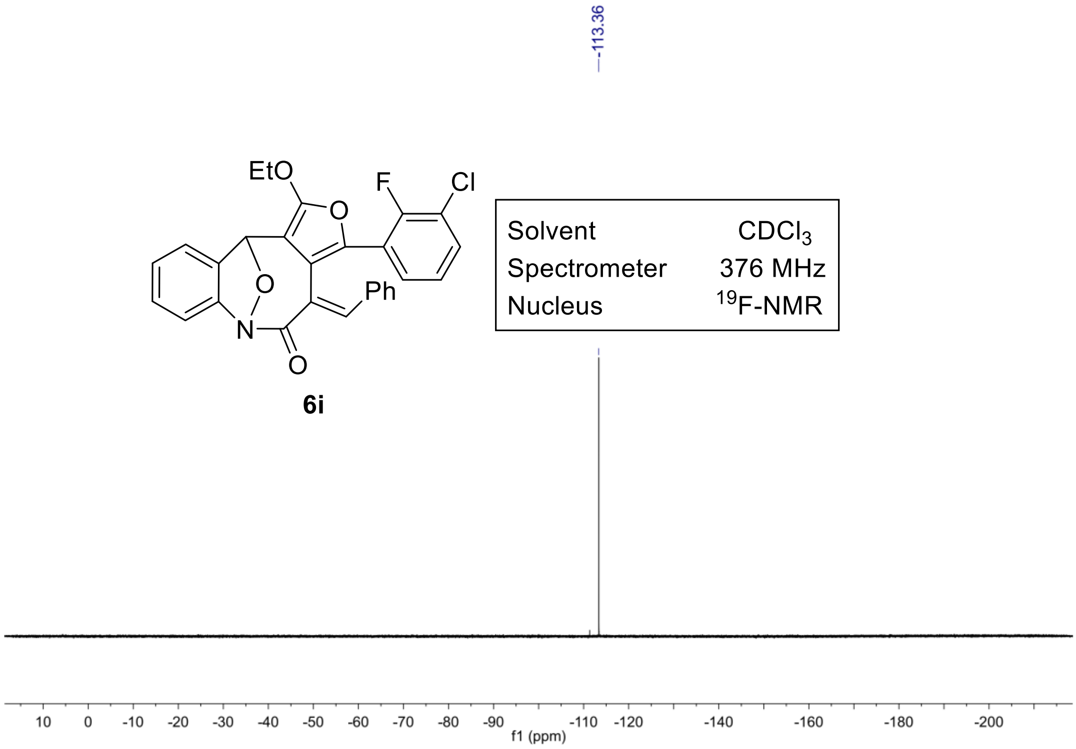

-113.36
EtO
F
Cl
O
O
N
O
Ph
6i
Solvent CDCl$_3$
Spectrometer 376 MHz
Nucleus ^{19}F-NMR
10 0 -10 -20 -30 -40 -50 -60 -70 -80 -90 -110 -120 -140 -160 -180 -200
f1 (ppm)

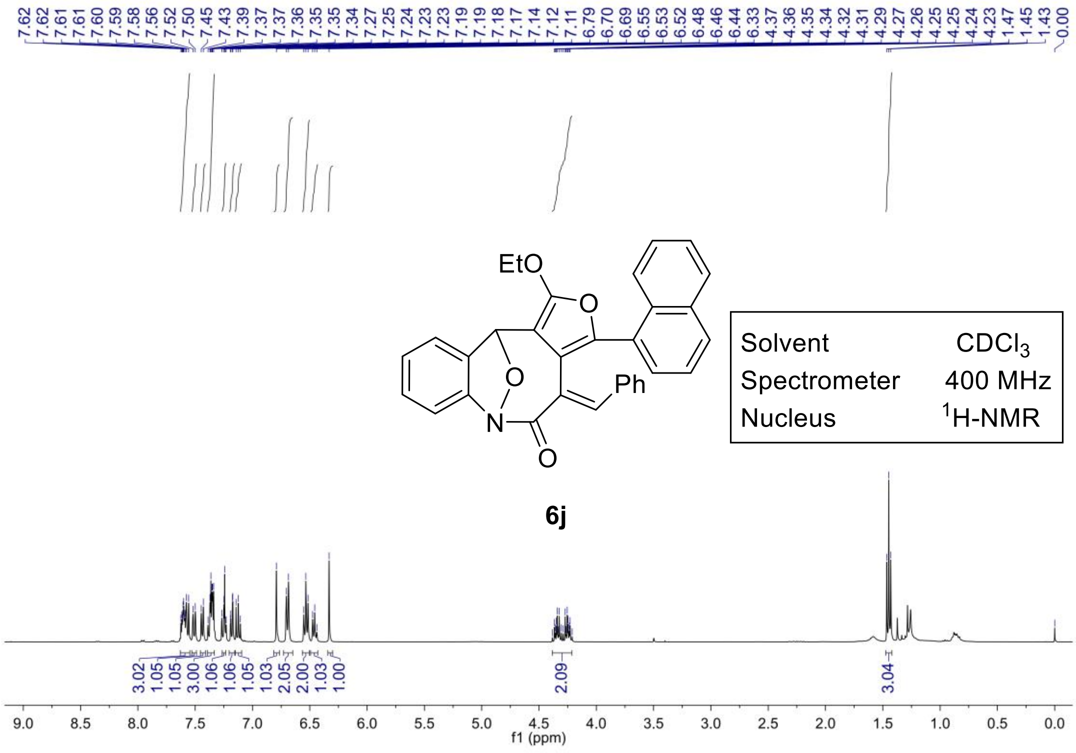

7.62 7.62 7.61 7.61 7.60 7.59 7.58 7.56 7.52 7.50 7.45 7.43 7.39 7.37 7.37 7.36 7.35 7.35 7.34 7.27 7.25 7.24 7.23 7.23 7.19 7.19 7.18 7.17 7.14 7.12 7.11 6.79 6.70 6.69 6.55 6.53 6.52 6.48 6.46 6.44 6.33 4.37 4.36 4.35 4.34 4.32 4.31 4.29 4.27 4.26 4.25 4.25 4.24 4.23 1.47 1.45 1.43 0.00
EtO
O
O
N
O
Ph
6j
Solvent CDCl$_3$
Spectrometer 400 MHz
Nucleus ^{1}H-NMR
3.02 1.05 3.00 1.06 1.06 1.05 1.03 2.05 2.00 1.03 1.00
2.09
3.04
9.0 8.5 8.0 7.5 7.0 6.5 6.0 5.5 5.0 4.5 4.0 3.5 3.0 2.5 2.0 1.5 1.0 0.5 0.0
f1 (ppm)

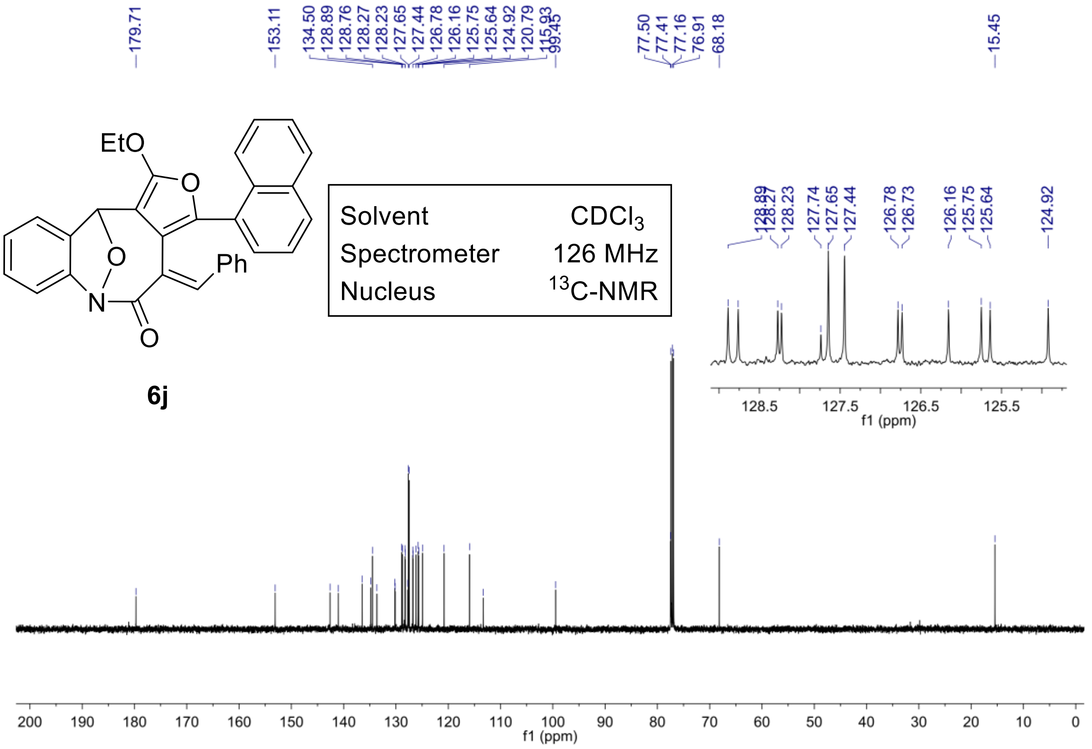
EtO
Ph
6j
Solvent CDCl$_3$
Spectrometer 126 MHz
Nucleus ^{13}C-NMR
f1 (ppm)

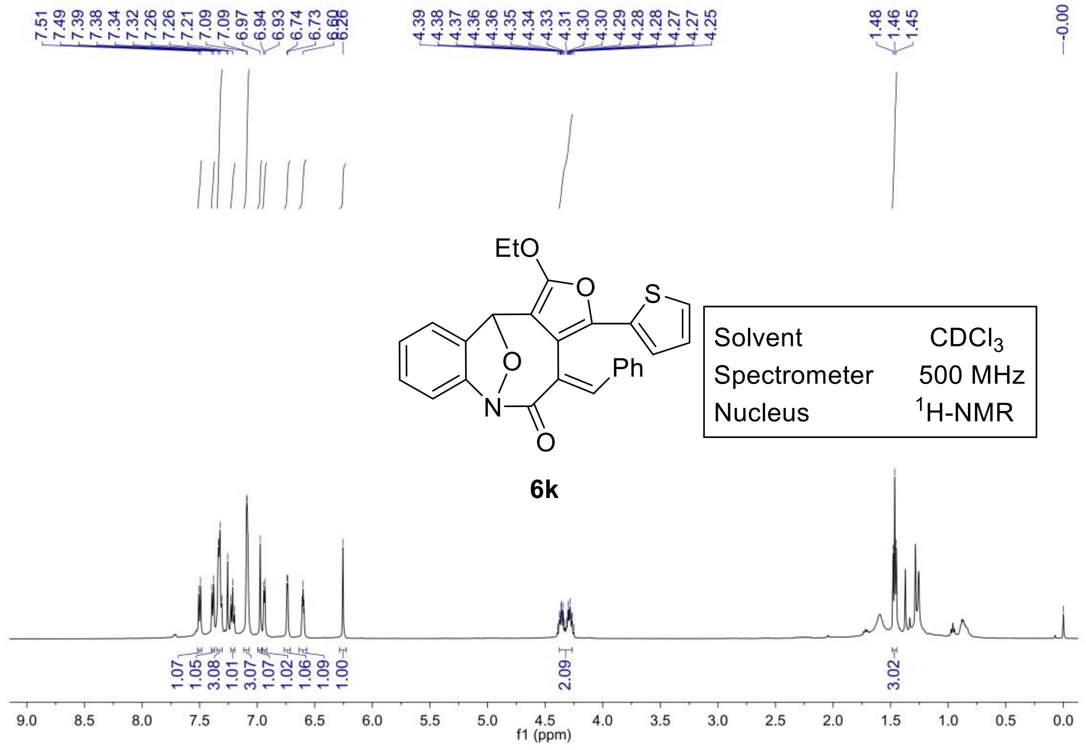
EtO
S
Ph
6k
Solvent CDCl$_3$
Spectrometer 500 MHz
Nucleus ^{1}H-NMR
f1 (ppm)

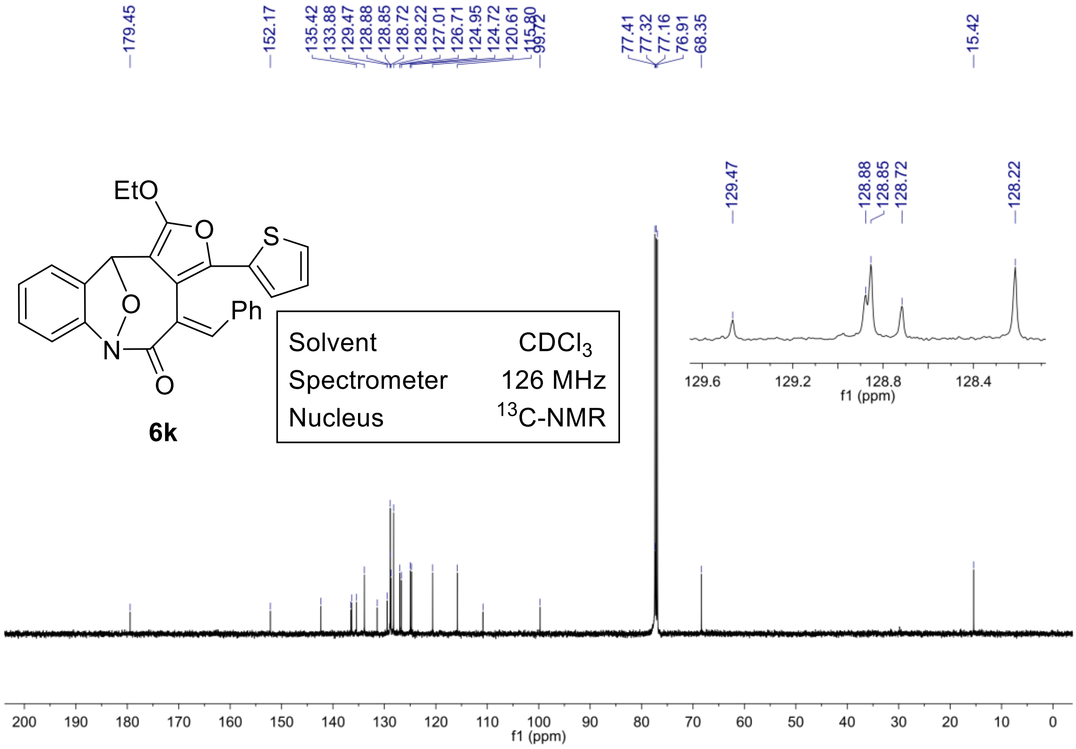
Solvent CDCl3
Spectrometer 126 MHz
Nucleus 13C-NMR
6k

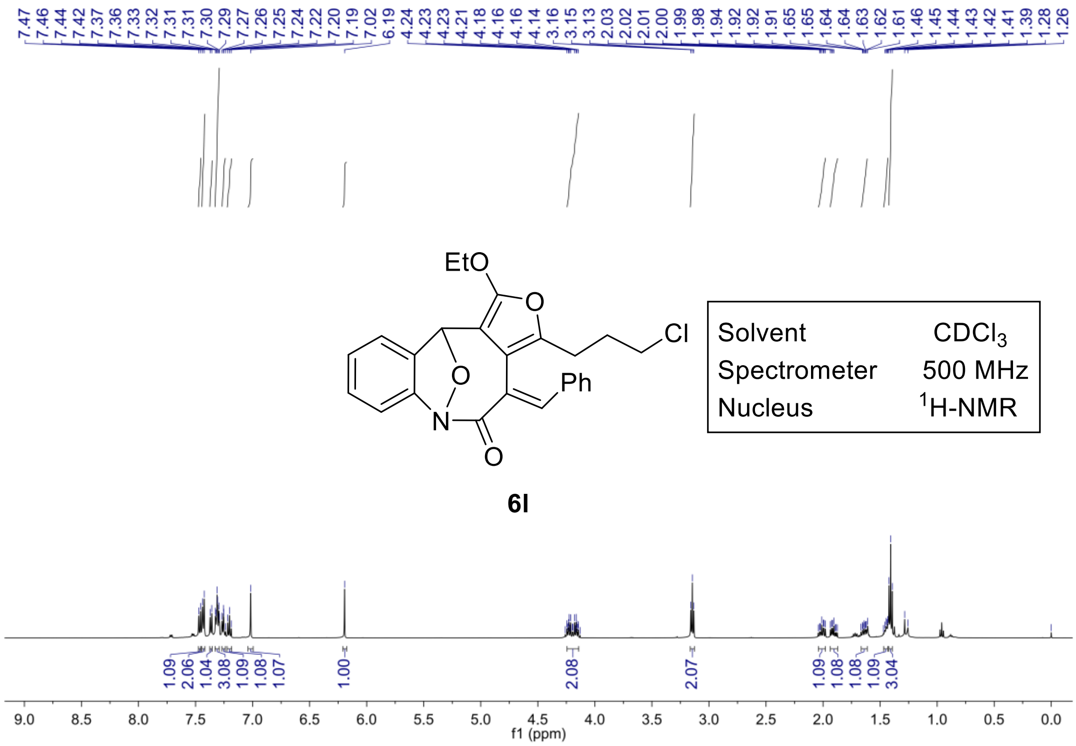
Solvent CDCl3
Spectrometer 500 MHz
Nucleus 1H-NMR
6l

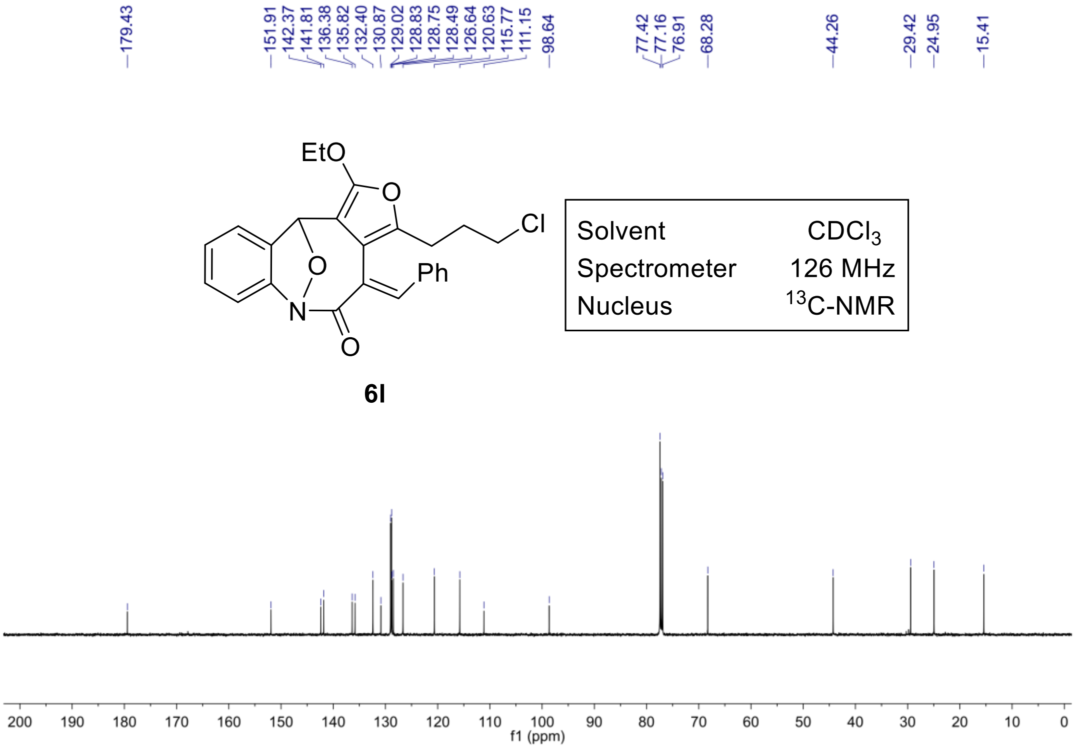
179.43
151.91
142.37
141.81
136.38
135.82
132.40
130.87
129.02
128.83
128.75
128.49
126.64
120.63
115.77
111.15
98.64
77.42
77.16
76.91
68.28
44.26
29.42
24.95
15.41
EtO
O
Cl
O
Ph
N
O
Solvent CDCl3
Spectrometer 126 MHz
Nucleus 13C-NMR
f1 (ppm)

6l

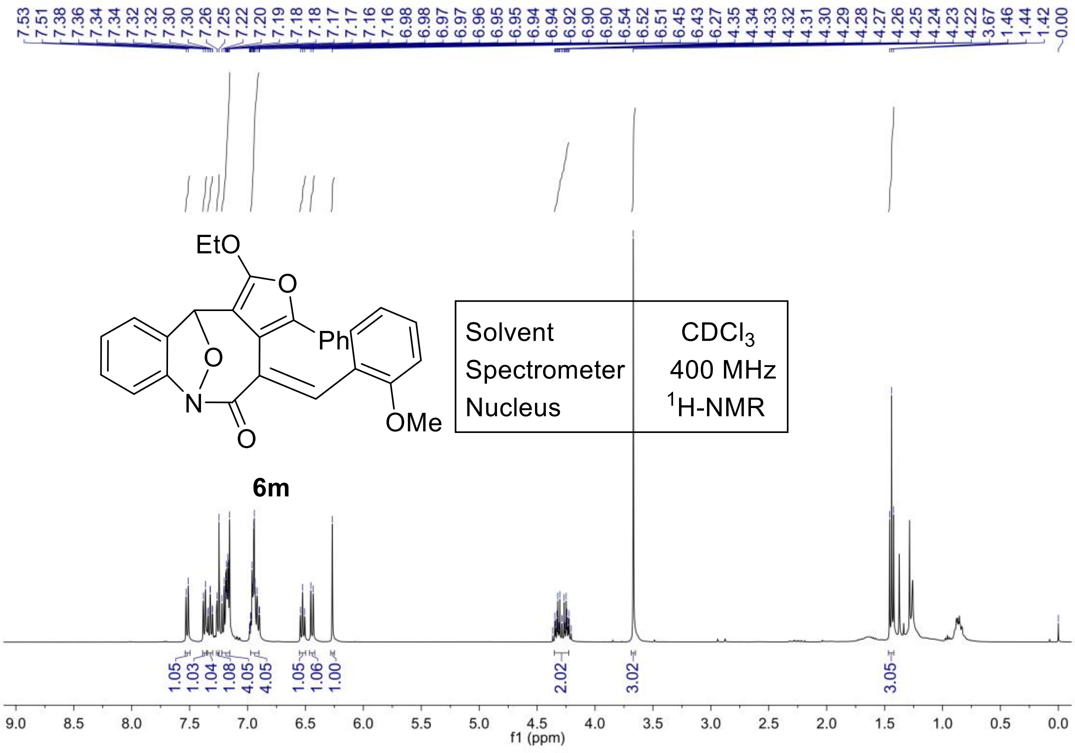
EtO
O
Ph
O
N
O
OMe
Solvent CDCl3
Spectrometer 400 MHz
Nucleus 1H-NMR
f1 (ppm)

6m

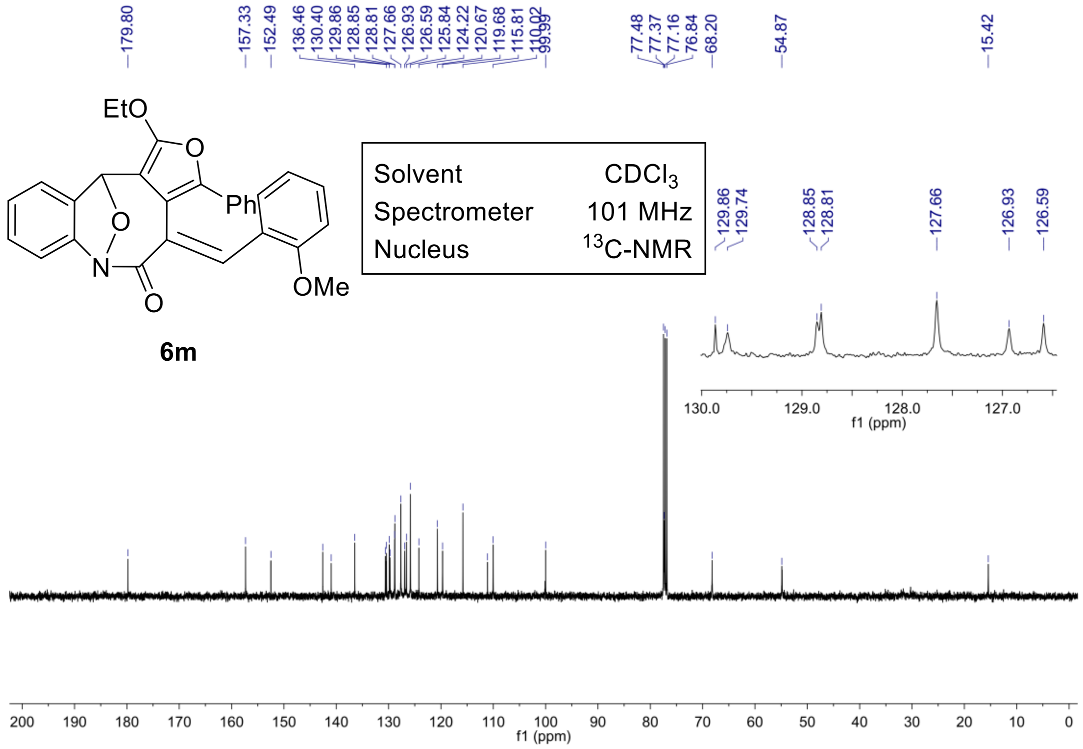
Solvent CDCl3
Spectrometer 101 MHz
Nucleus 13C-NMR
6m
f1 (ppm)

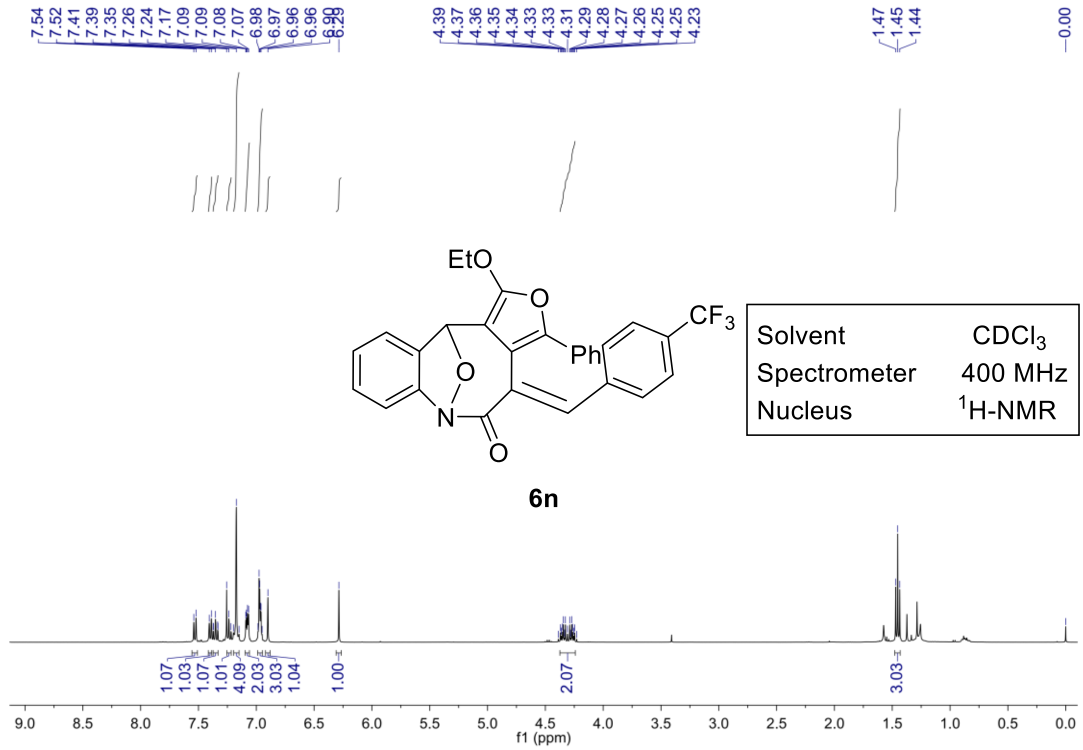
Solvent CDCl3
Spectrometer 400 MHz
Nucleus 1H-NMR
6n
f1 (ppm)

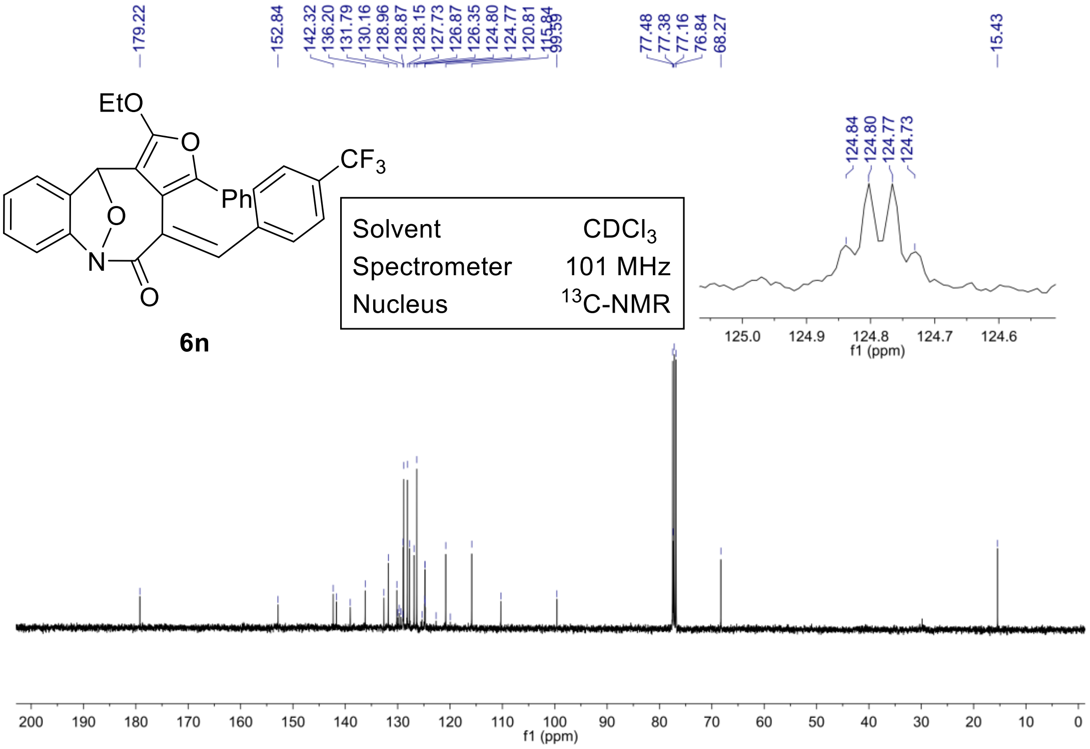
EtO
O
CF3
Ph
O
N
O
6n
Solvent CDCl3
Spectrometer 101 MHz
Nucleus 13C-NMR
179.22
152.84
142.32
136.20
131.79
130.16
128.96
128.87
128.15
127.73
126.87
126.35
124.80
124.77
120.81
115.84
99.59
77.48
77.38
77.16
76.84
68.27
15.43
124.84
124.80
124.77
124.73
f1 (ppm)

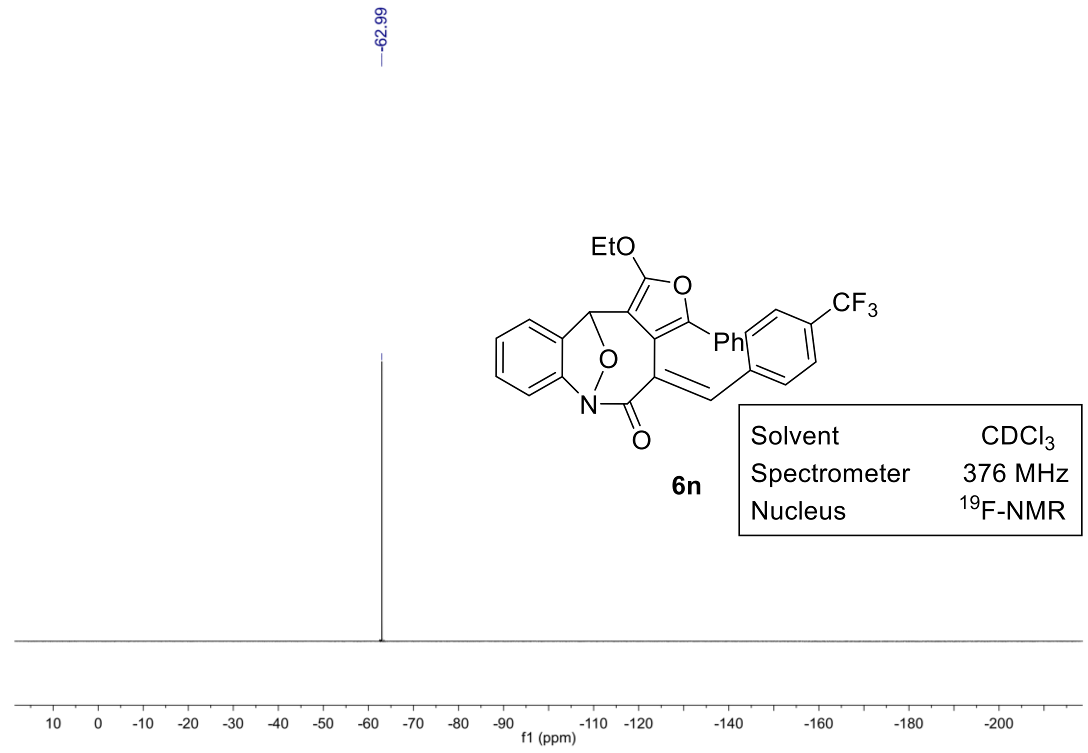
-62.99
EtO
O
CF3
Ph
O
N
O
6n
Solvent CDCl3
Spectrometer 376 MHz
Nucleus 19F-NMR
f1 (ppm)

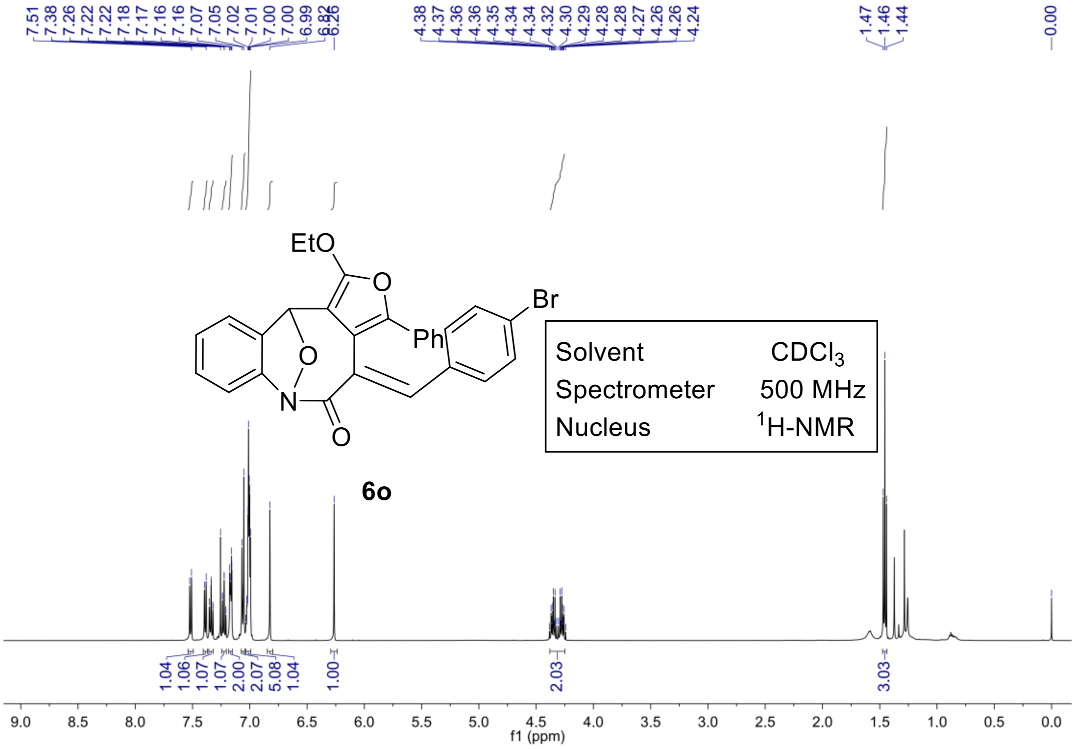
EtO
O
Br
Ph
O
N
O
6o
Solvent CDCl3
Spectrometer 500 MHz
Nucleus 1H-NMR
f1 (ppm)

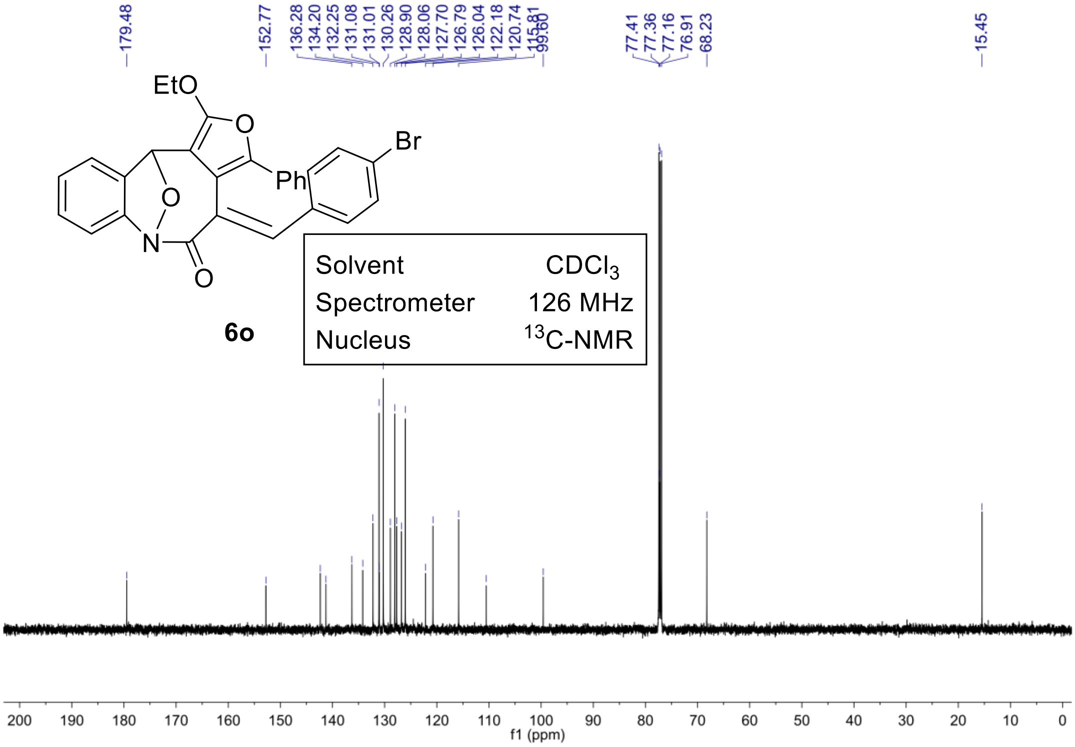
EtO
O
Br
Ph
O
N
O
6o
Solvent CDCl3
Spectrometer 126 MHz
Nucleus 13C-NMR
f1 (ppm)

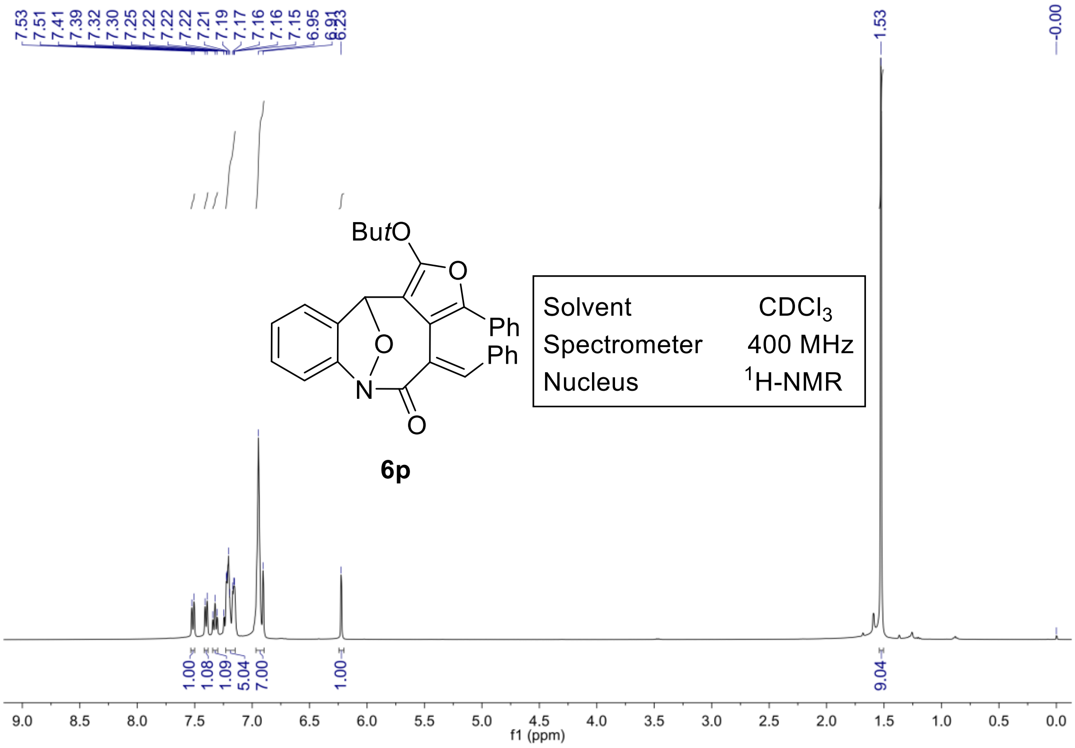
7.53
7.51
7.41
7.39
7.32
7.30
7.25
7.22
7.22
7.22
7.21
7.19
7.17
7.16
7.16
7.15
6.95
6.91
6.23
1.53
-0.00
ButO
O
Ph
Ph
O
N
O
Solvent CDCl3
Spectrometer 400 MHz
Nucleus 1H-NMR
1.00
1.08
1.09
5.04
7.00
1.00
9.04
f1 (ppm)

6p

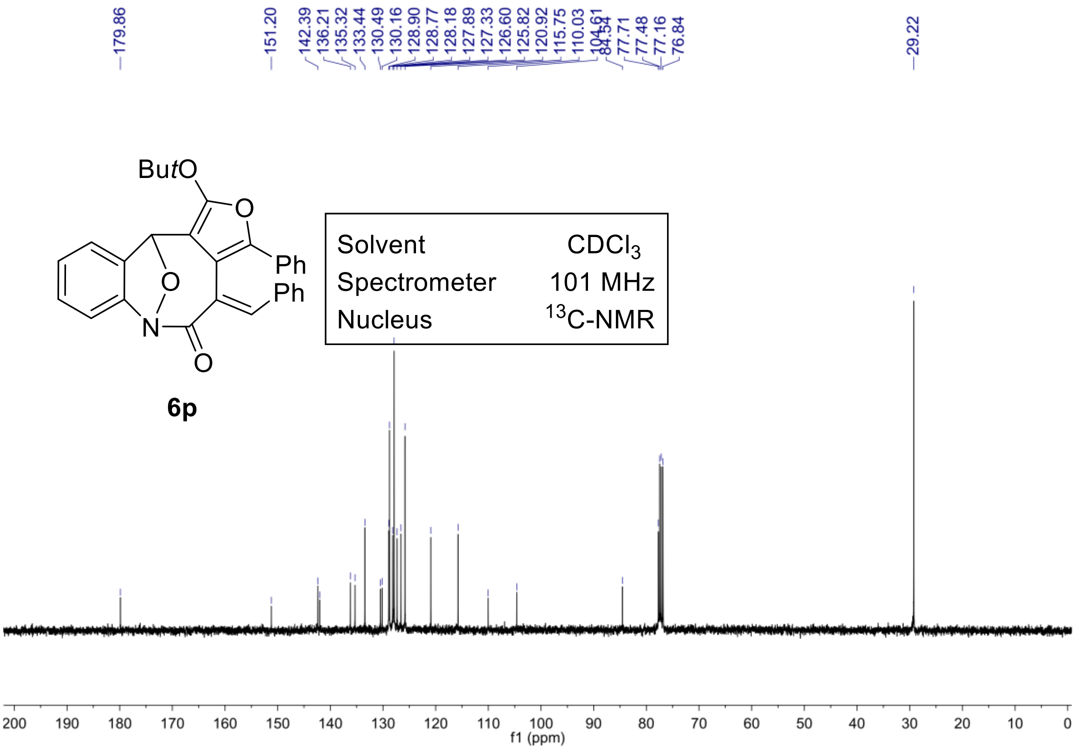
179.86
151.20
142.39
136.21
135.32
133.44
130.49
130.16
128.90
128.77
128.18
127.89
127.33
126.60
125.82
120.92
115.75
110.03
104.61
84.54
77.71
77.48
77.16
76.84
29.22
ButO
O
Ph
Ph
O
N
O
Solvent CDCl3
Spectrometer 101 MHz
Nucleus 13C-NMR
f1 (ppm)

6p

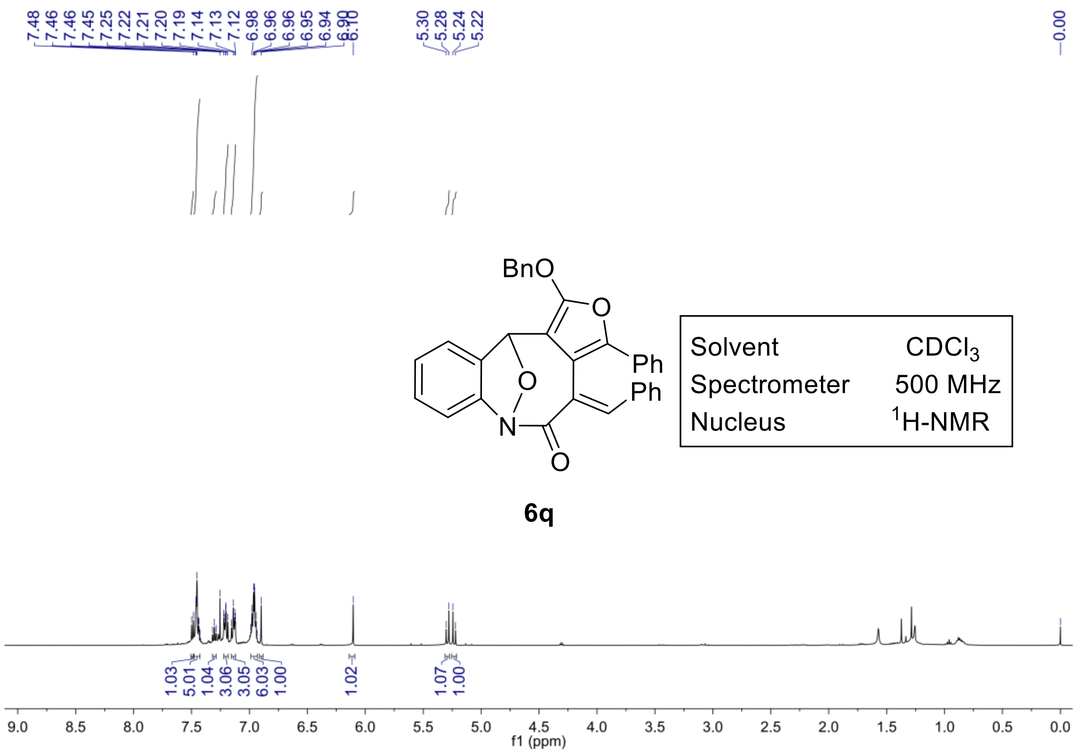
7.48 7.46 7.46 7.45 7.25 7.22 7.21 7.20 7.19 7.14 7.13 7.12 6.98 6.96 6.96 6.95 6.94 6.90 6.10
5.30 5.28 5.24 5.22
0.00
BnO
O
Ph
Ph
O
N
O
6q
Solvent CDCl3
Spectrometer 500 MHz
Nucleus 1H-NMR
1.03 5.01 1.04 3.06 3.05 6.03 1.00 1.02 1.07 1.00
f1 (ppm)

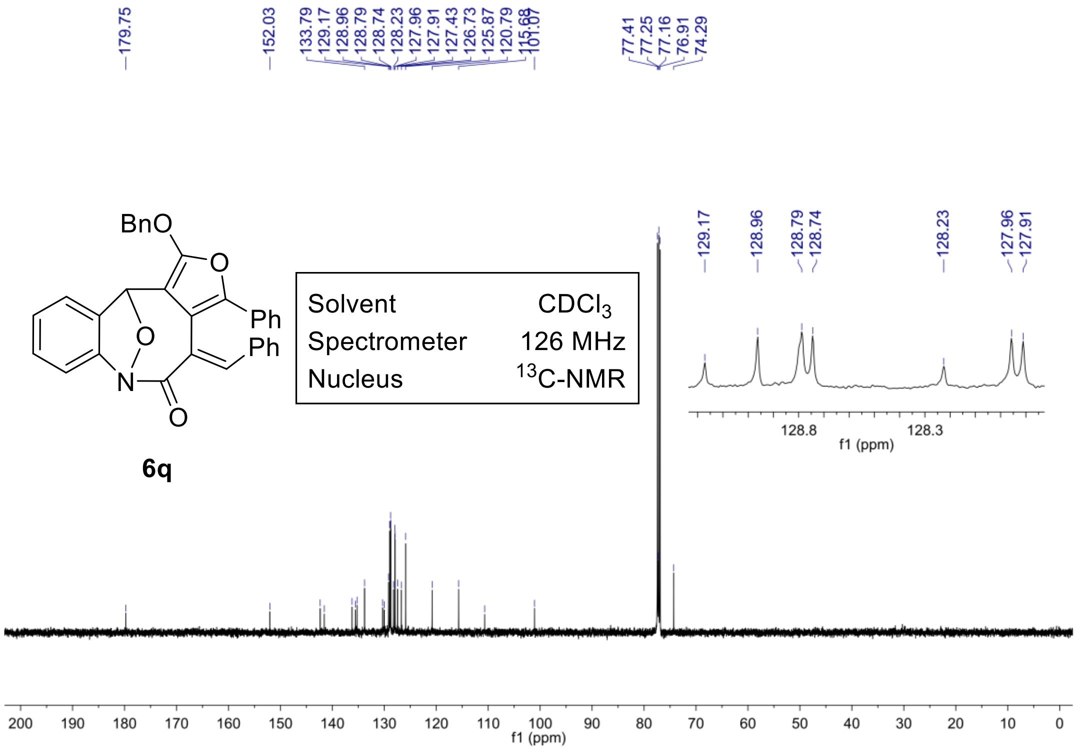
179.75
152.03
133.79 129.17 128.96 128.79 128.74 128.23 127.96 127.91 127.43 126.73 125.87 120.79 115.68 101.07
77.41 77.25 77.16 76.91 74.29
129.17 128.96 128.79 128.74 128.23 127.96 127.91
128.8 128.3
BnO
O
Ph
Ph
O
N
O
6q
Solvent CDCl3
Spectrometer 126 MHz
Nucleus 13C-NMR
f1 (ppm)

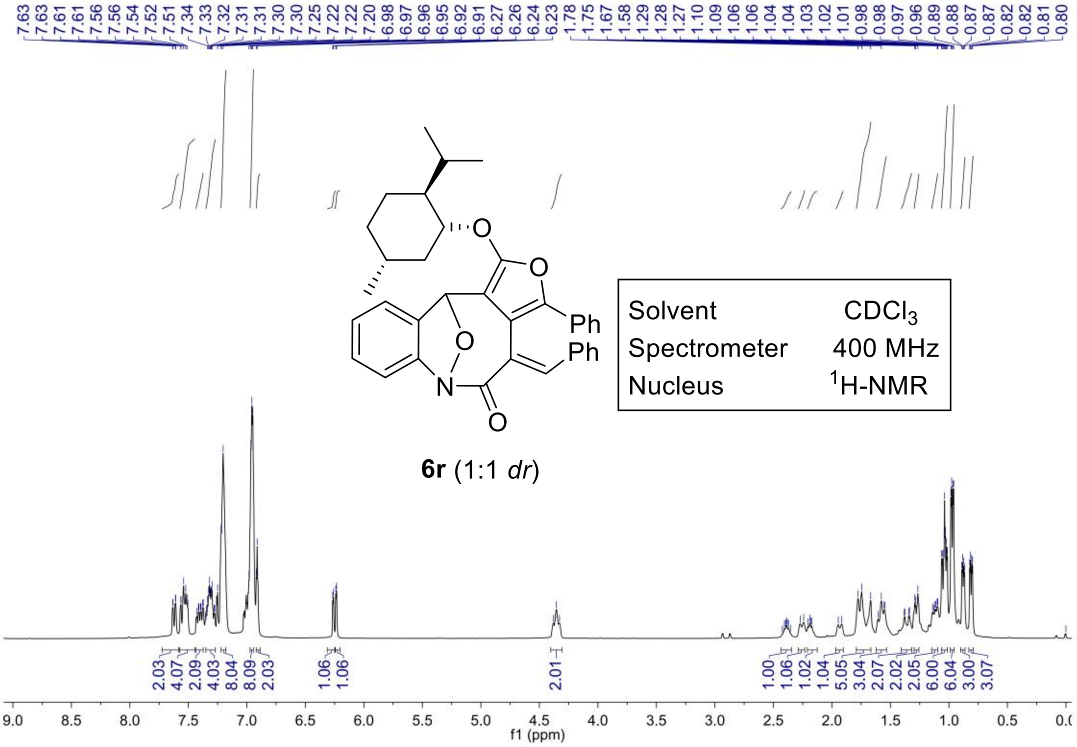
Solvent CDCl3
Spectrometer 400 MHz
Nucleus 1H-NMR
6r (1:1 dr)
f1 (ppm)

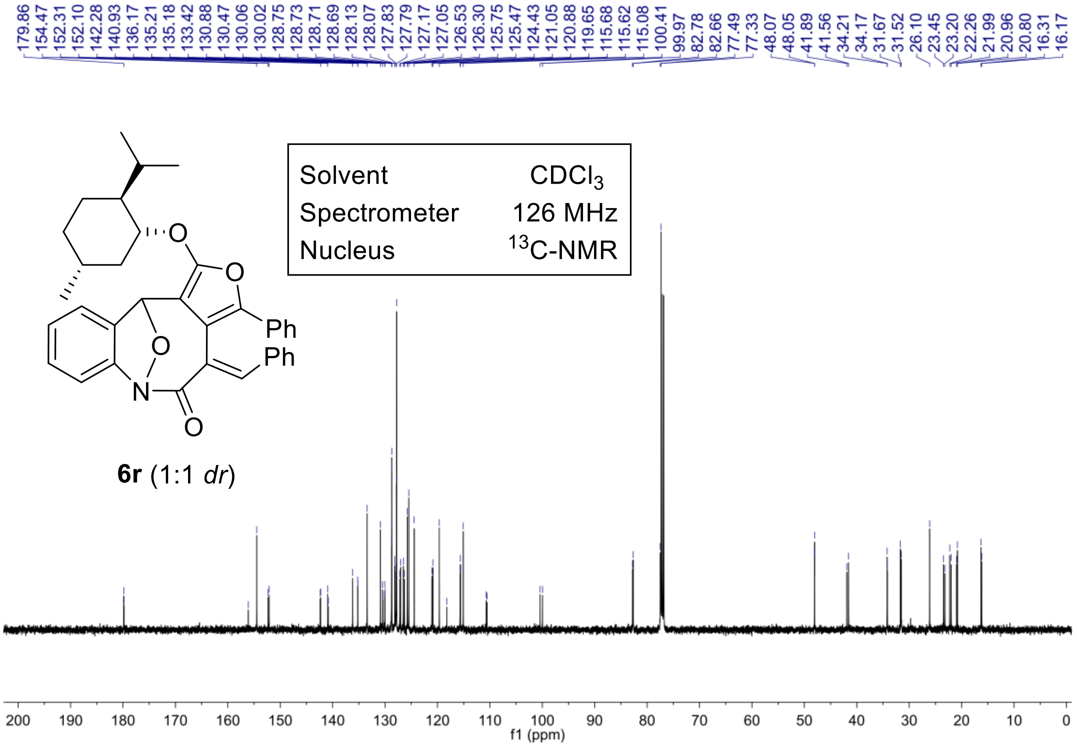
Solvent CDCl3
Spectrometer 126 MHz
Nucleus 13C-NMR
6r (1:1 dr)
f1 (ppm)

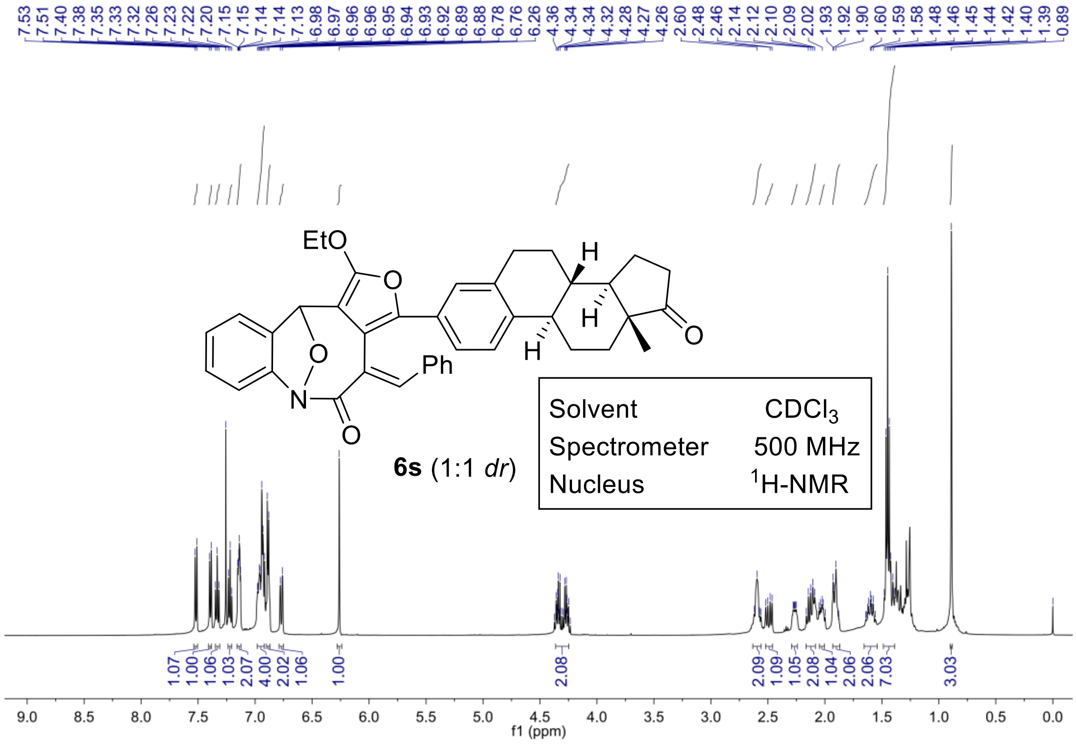
6s (1:1 dr)
Solvent CDCl3
Spectrometer 500 MHz
Nucleus 1H-NMR
f1 (ppm)

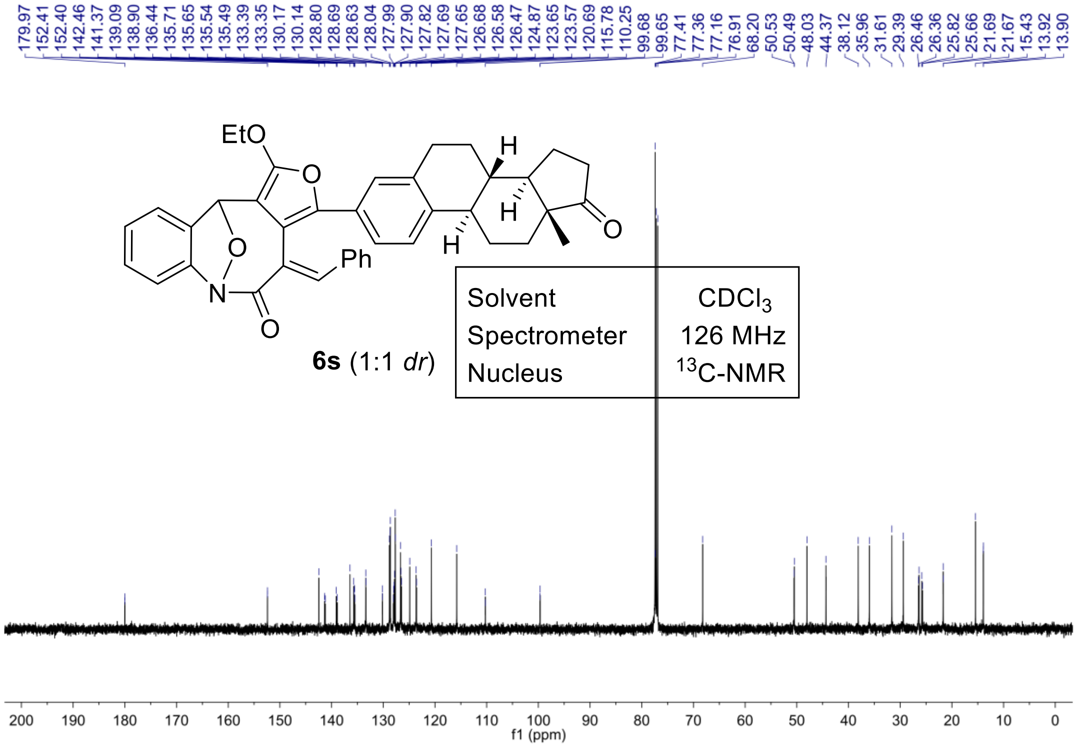
6s (1:1 dr)
Solvent CDCl3
Spectrometer 126 MHz
Nucleus 13C-NMR
f1 (ppm)

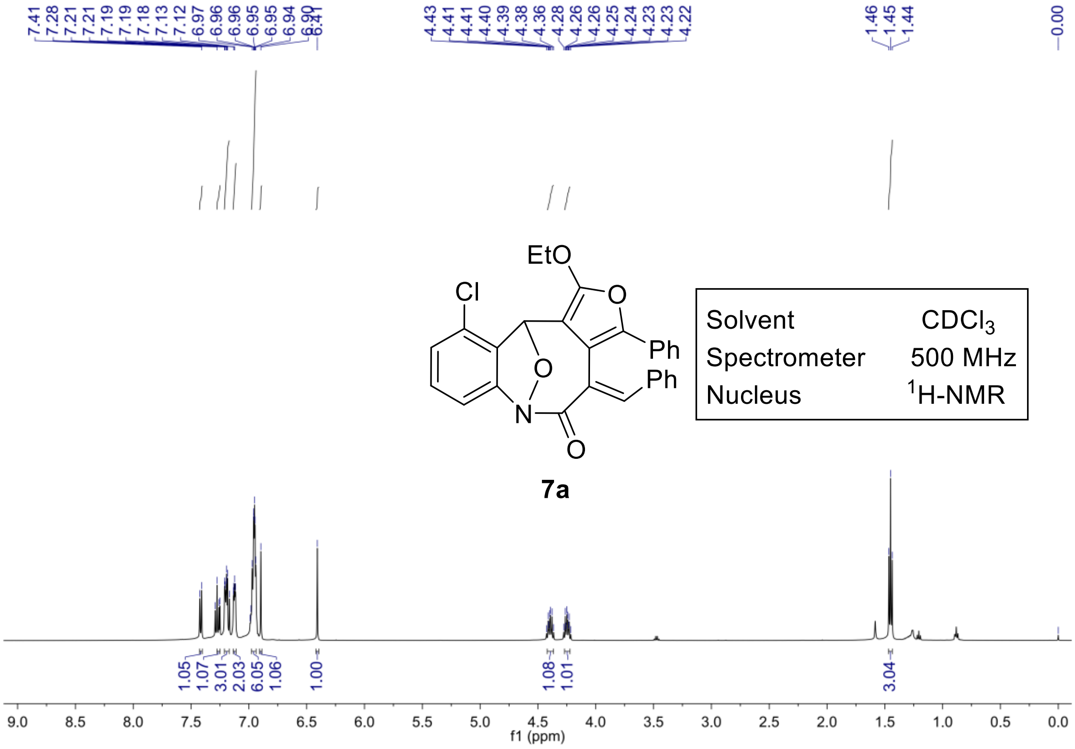
Solvent CDCl3
Spectrometer 500 MHz
Nucleus 1H-NMR
7a
f1 (ppm)

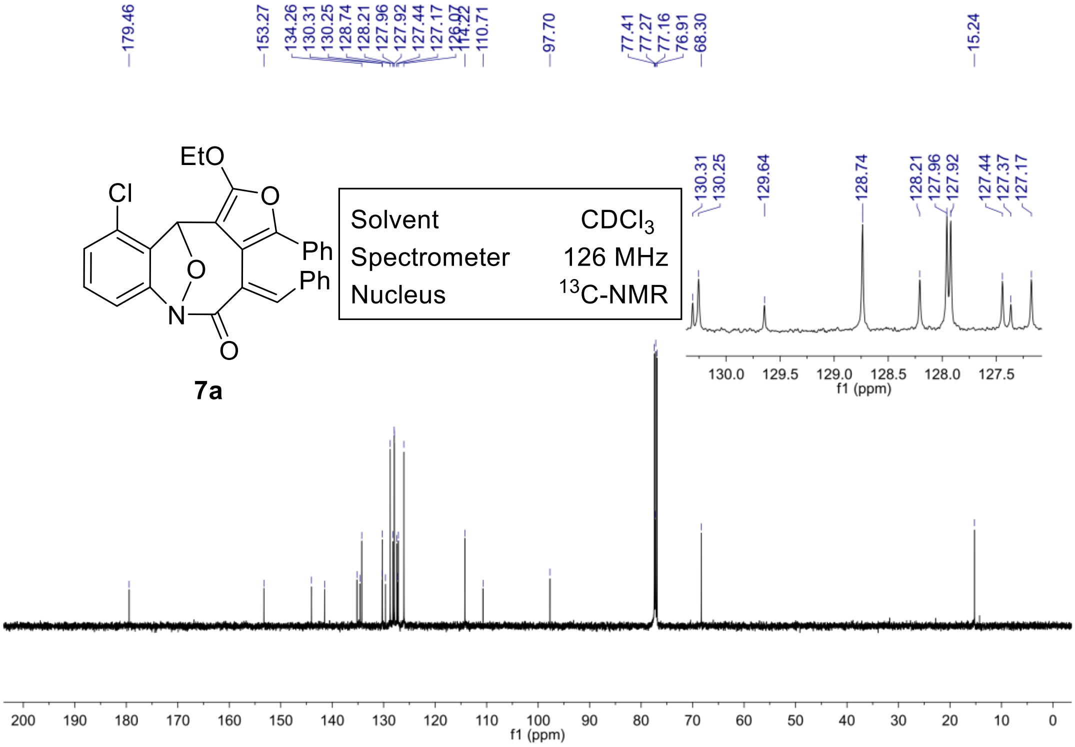
Solvent CDCl3
Spectrometer 126 MHz
Nucleus 13C-NMR
7a
f1 (ppm)

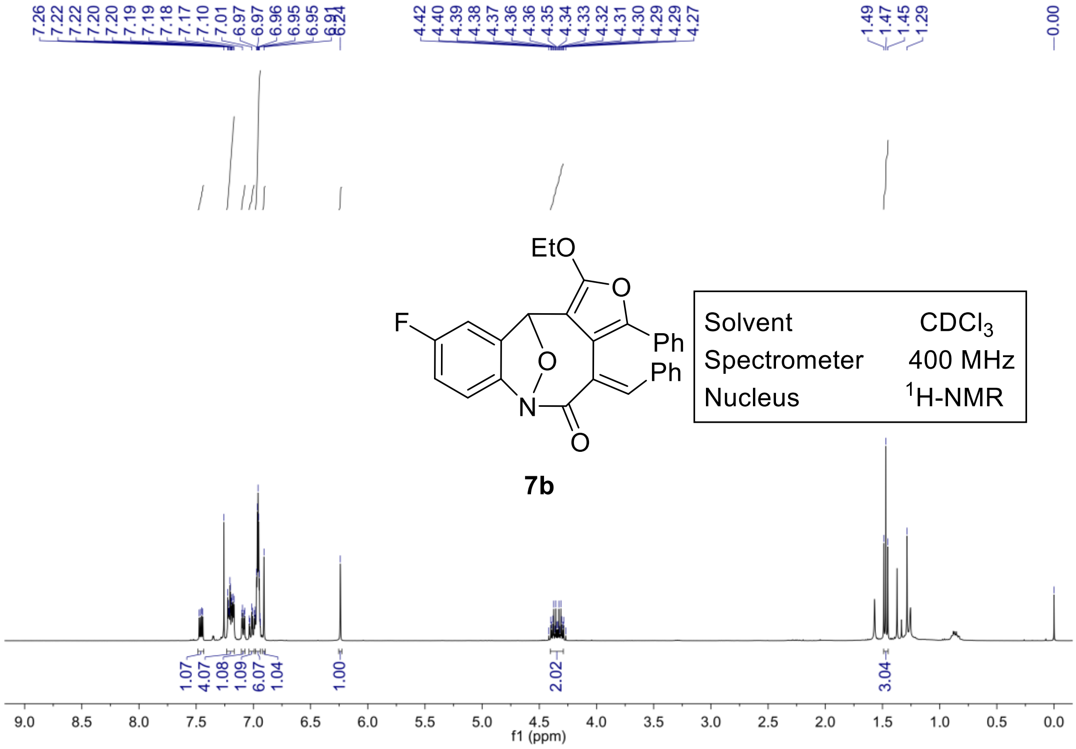
EtO
O
F
Ph
O
Ph
N
O
7b
Solvent CDCl3
Spectrometer 400 MHz
Nucleus 1H-NMR
f1 (ppm)

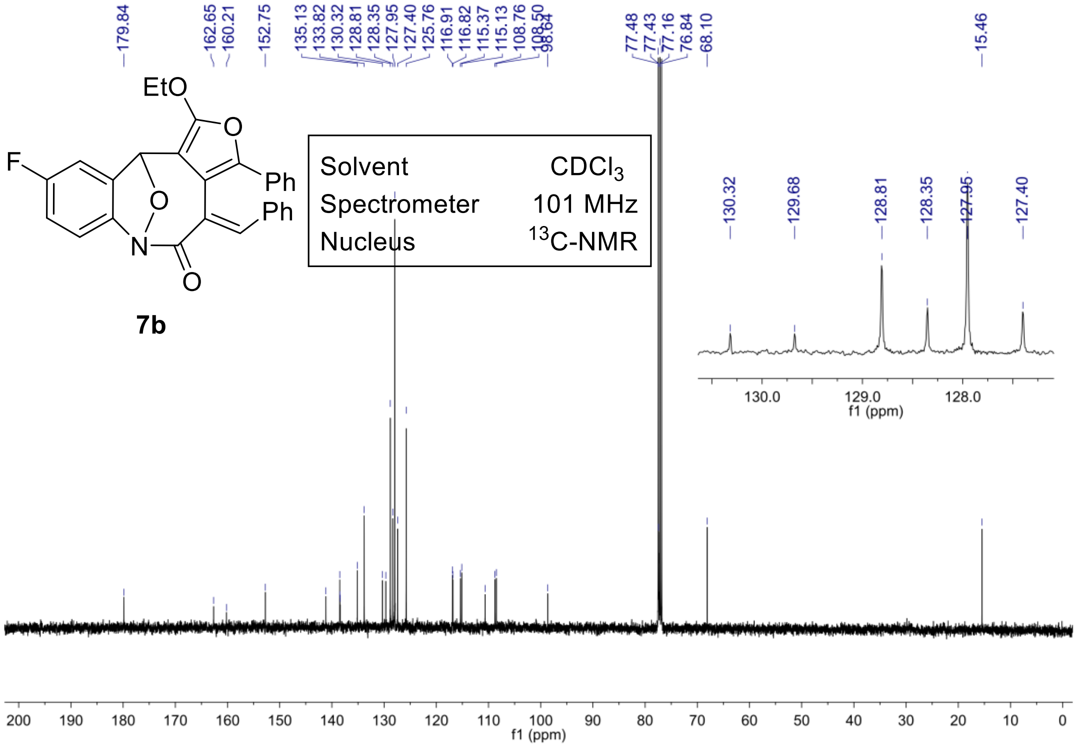
EtO
O
F
Ph
O
Ph
N
O
7b
Solvent CDCl3
Spectrometer 101 MHz
Nucleus 13C-NMR
f1 (ppm)

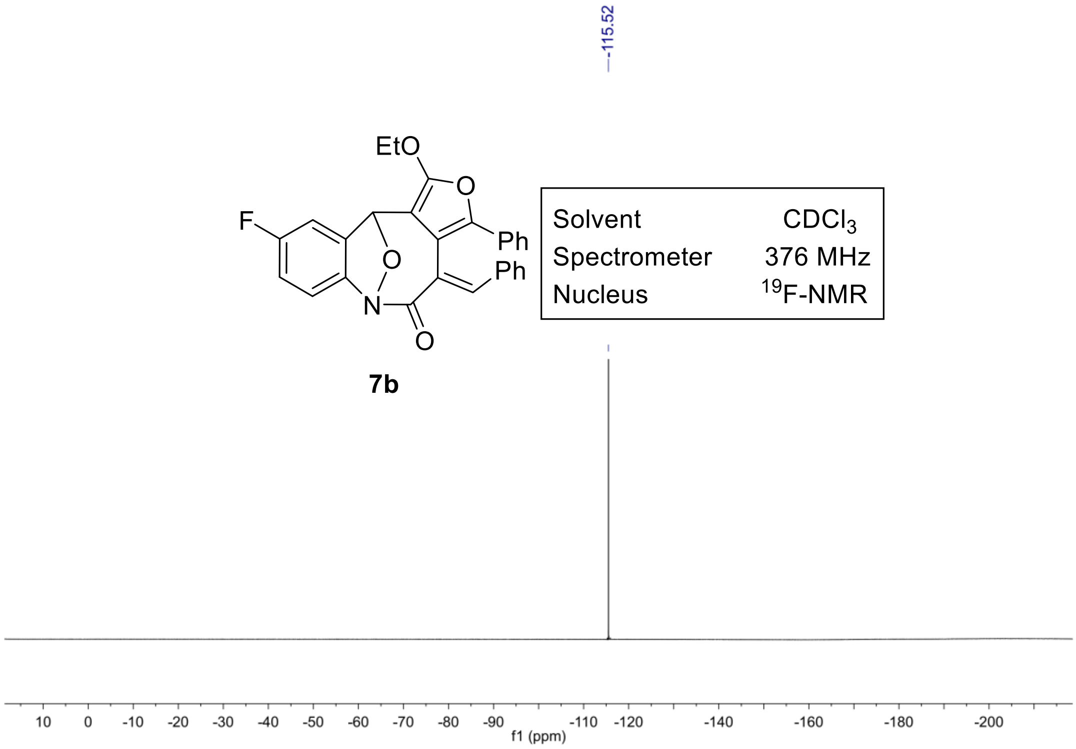
-115.52
EtO
O
F
Ph
Ph
O
N
O
7b
Solvent CDCl$_3$
Spectrometer 376 MHz
Nucleus ^{19}F-NMR
10 0 -10 -20 -30 -40 -50 -60 -70 -80 -90 -110 -120 -140 -160 -180 -200
f1 (ppm)

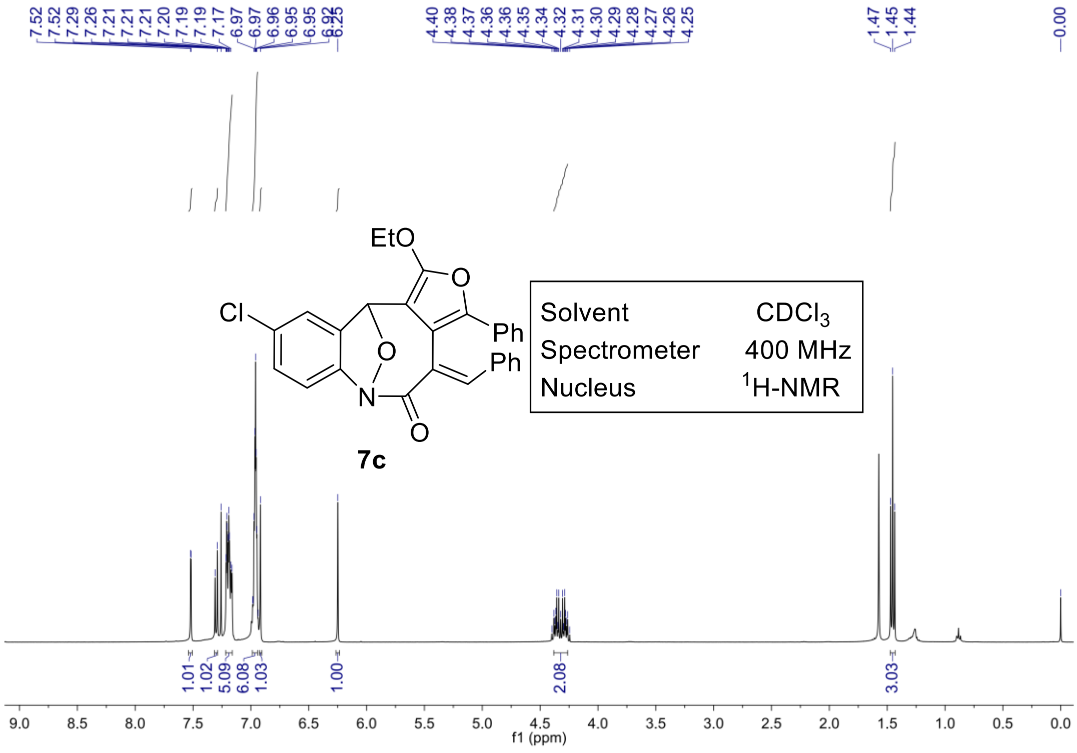
7.52 7.52 7.29 7.26 7.21 7.21 7.21 7.20 7.19 7.19 7.17 6.97 6.97 6.96 6.95 6.95 6.92 6.25
4.40 4.38 4.37 4.36 4.36 4.35 4.34 4.32 4.31 4.30 4.29 4.28 4.27 4.26 4.25
1.47 1.45 1.44
0.00
EtO
O
Cl
Ph
Ph
O
N
O
7c
Solvent CDCl$_3$
Spectrometer 400 MHz
Nucleus ^{1}H-NMR
1.01 1.02 5.09 6.08 1.03
1.00
2.08
3.03
9.0 8.5 8.0 7.5 7.0 6.5 6.0 5.5 5.0 4.5 4.0 3.5 3.0 2.5 2.0 1.5 1.0 0.5 0.0
f1 (ppm)

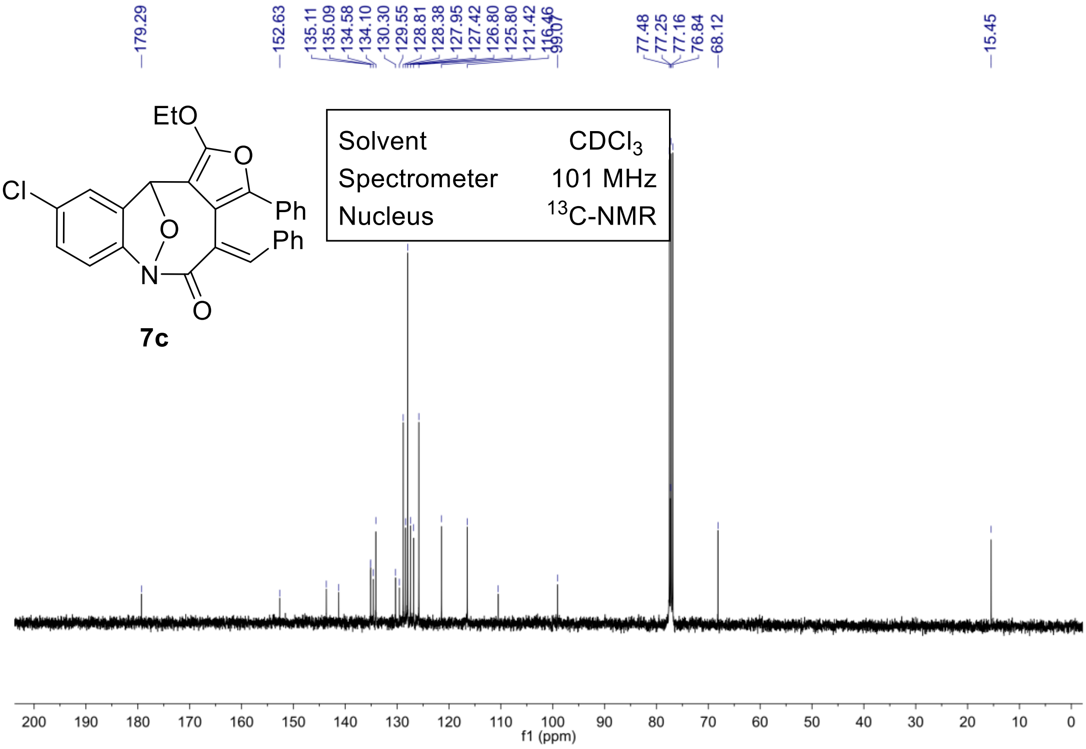
179.29
152.63
135.11
135.09
134.58
134.10
130.30
129.55
128.81
128.38
127.95
127.42
126.80
125.80
121.42
116.46
99.07
77.48
77.25
77.16
76.84
68.12
15.45
EtO
O
Cl
Ph
Ph
O
N
O
7c
Solvent CDCl3
Spectrometer 101 MHz
Nucleus 13C-NMR
f1 (ppm)

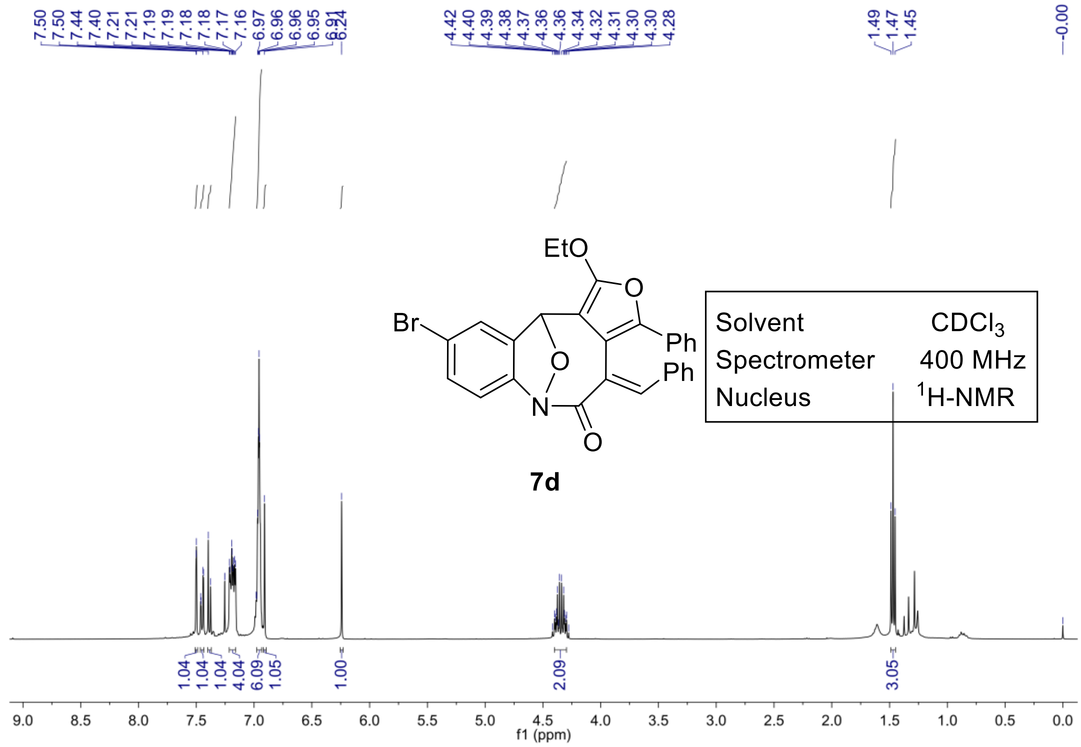
7.50
7.50
7.44
7.40
7.21
7.21
7.19
7.19
7.18
7.18
7.17
7.16
6.97
6.96
6.96
6.95
6.91
6.24
4.42
4.40
4.39
4.38
4.37
4.36
4.36
4.34
4.32
4.31
4.30
4.30
4.28
1.49
1.47
1.45
-0.00
EtO
O
Br
Ph
Ph
O
N
O
7d
Solvent CDCl3
Spectrometer 400 MHz
Nucleus 1H-NMR
1.04
1.04
1.04
4.04
6.09
1.05
1.00
2.09
3.05
f1 (ppm)

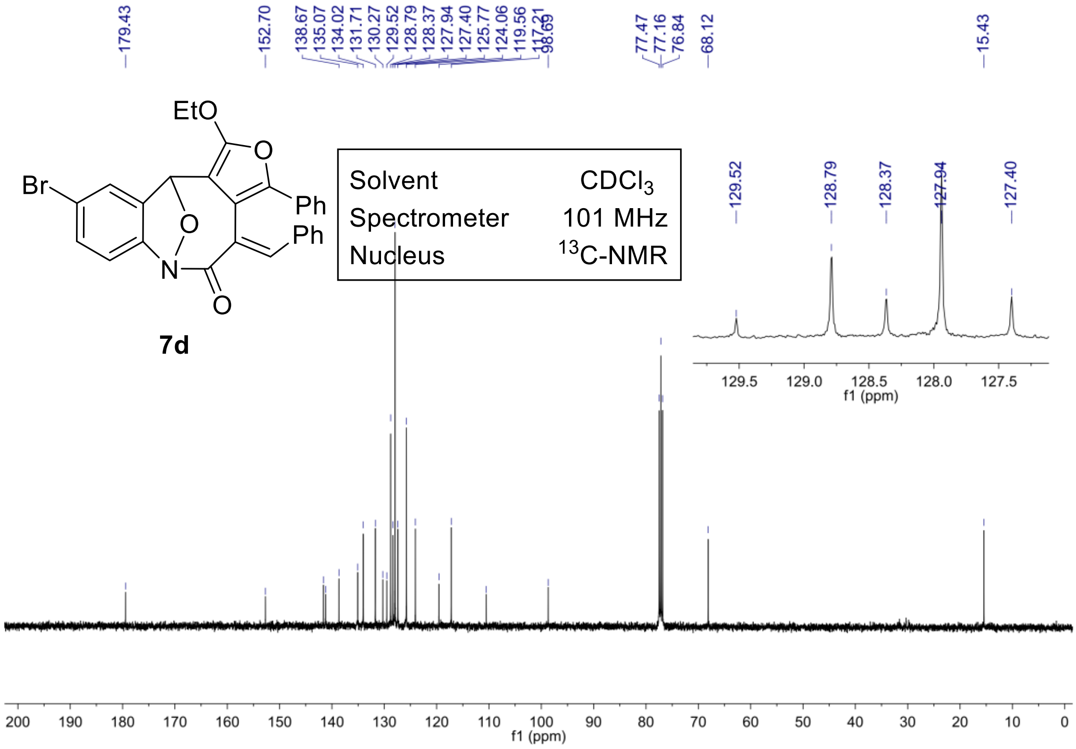
Solvent CDCl3
Spectrometer 101 MHz
Nucleus 13C-NMR
7d
f1 (ppm)

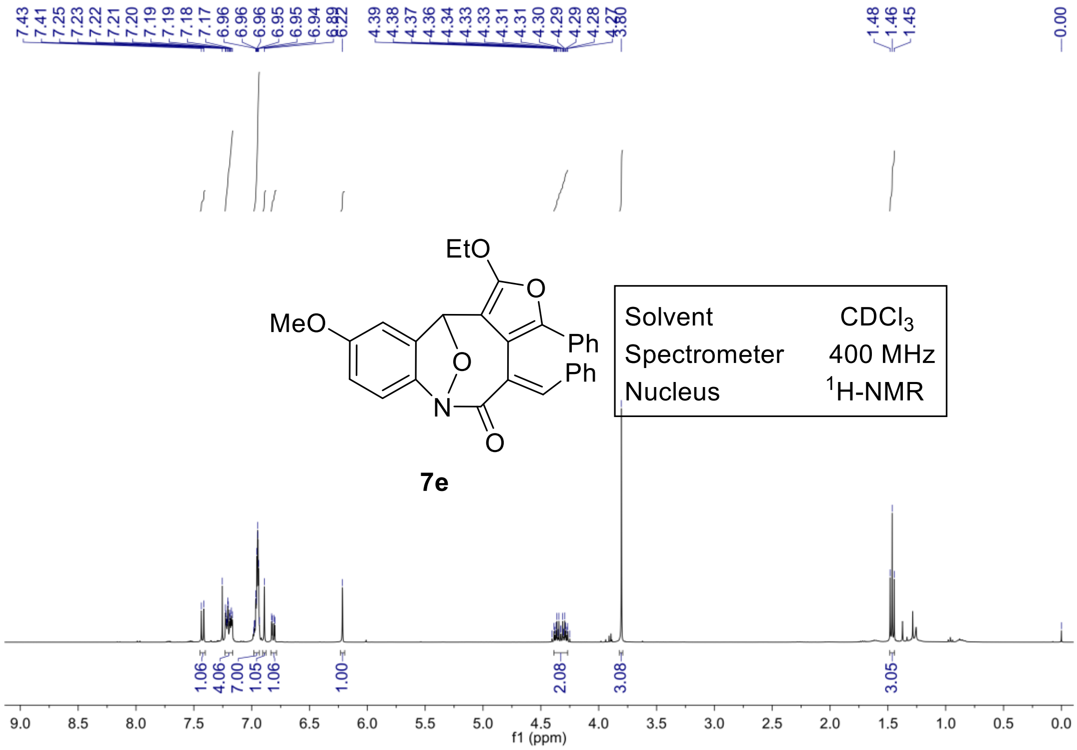
Solvent CDCl3
Spectrometer 400 MHz
Nucleus 1H-NMR
7e
f1 (ppm)

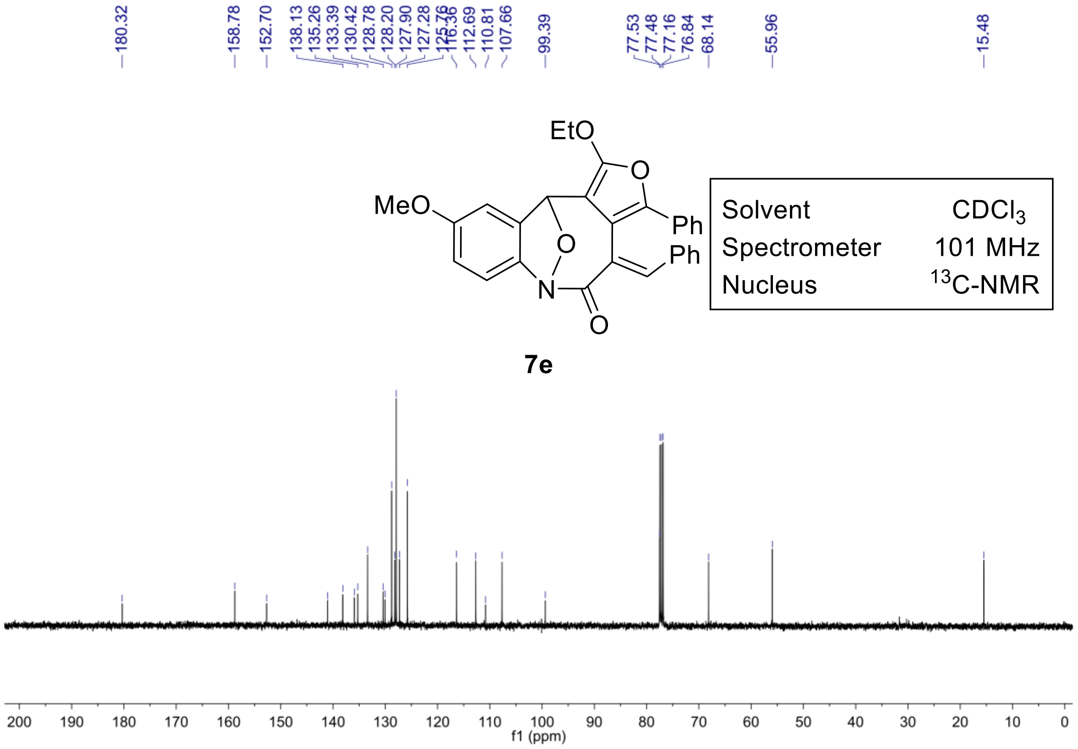
EtO
MeO
O
N
O
Ph
Ph
7e
Solvent CDCl3
Spectrometer 101 MHz
Nucleus 13C-NMR
f1 (ppm)

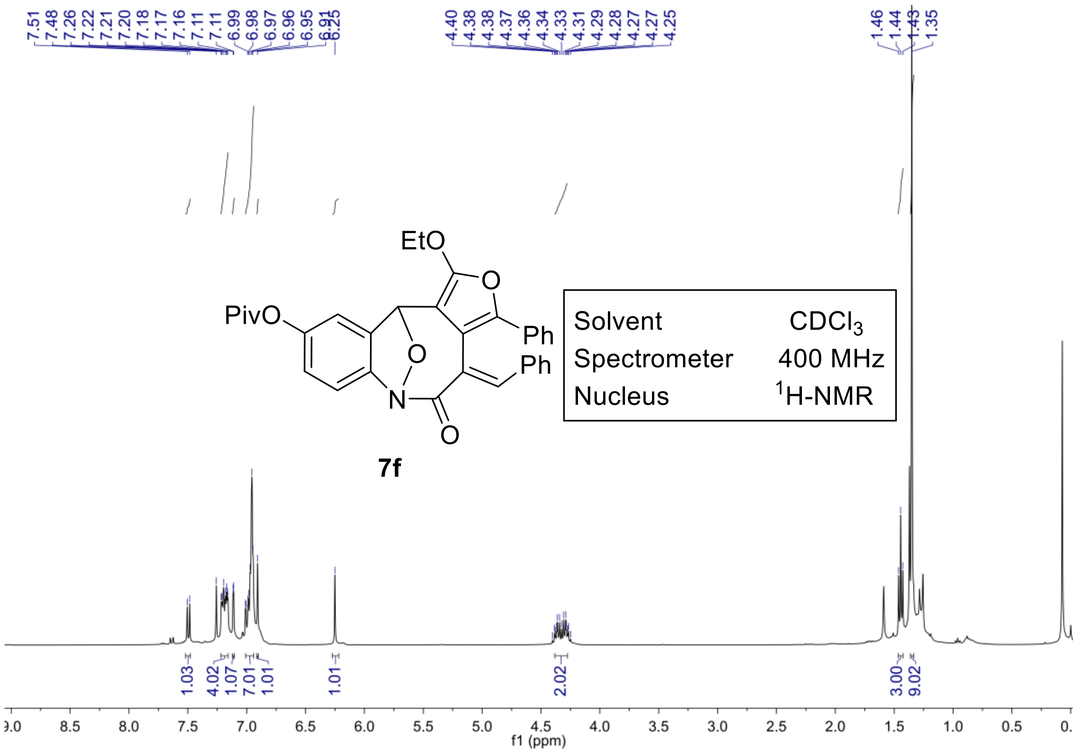
EtO
PivO
O
N
O
Ph
Ph
7f
Solvent CDCl3
Spectrometer 400 MHz
Nucleus 1H-NMR
f1 (ppm)

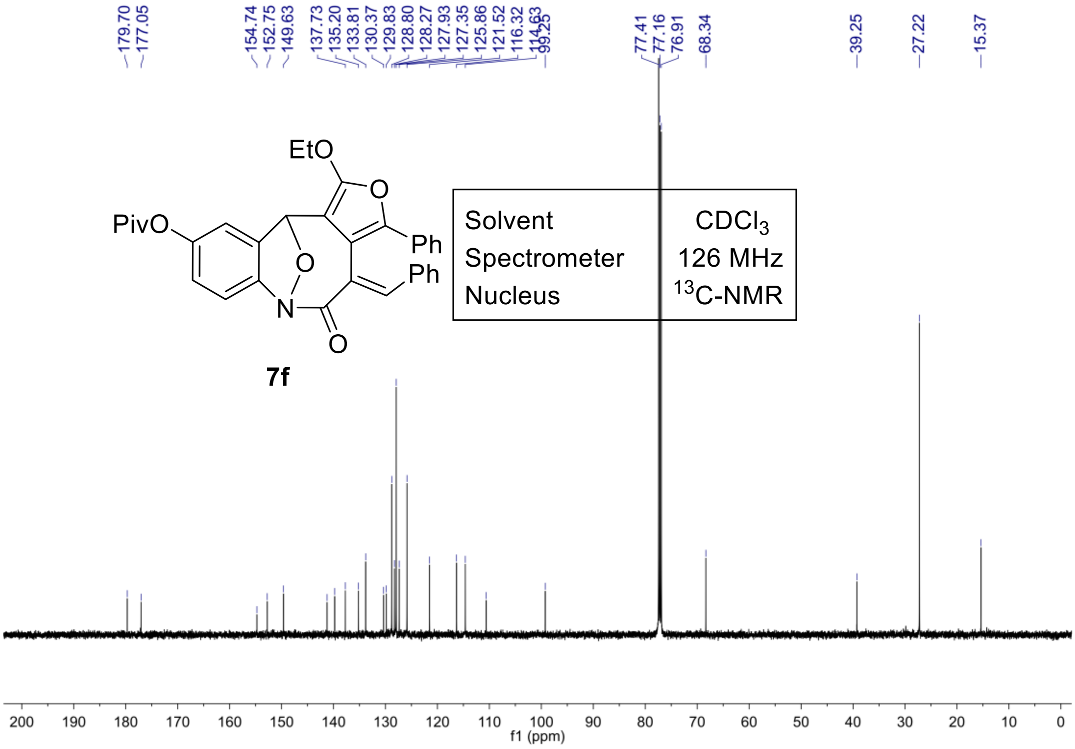
EtO
PivO
O
N
O
Ph
Ph
7f
Solvent CDCl3
Spectrometer 126 MHz
Nucleus 13C-NMR
f1 (ppm)

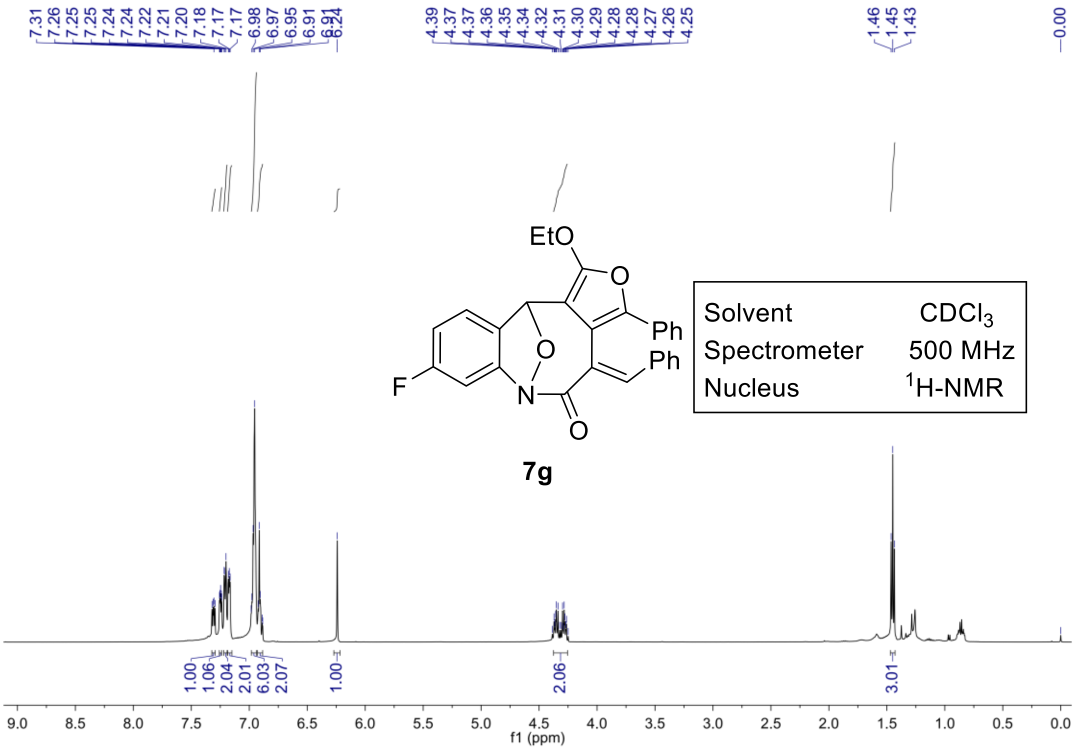
EtO
F
O
N
O
Ph
Ph
7g
Solvent CDCl3
Spectrometer 500 MHz
Nucleus 1H-NMR
f1 (ppm)

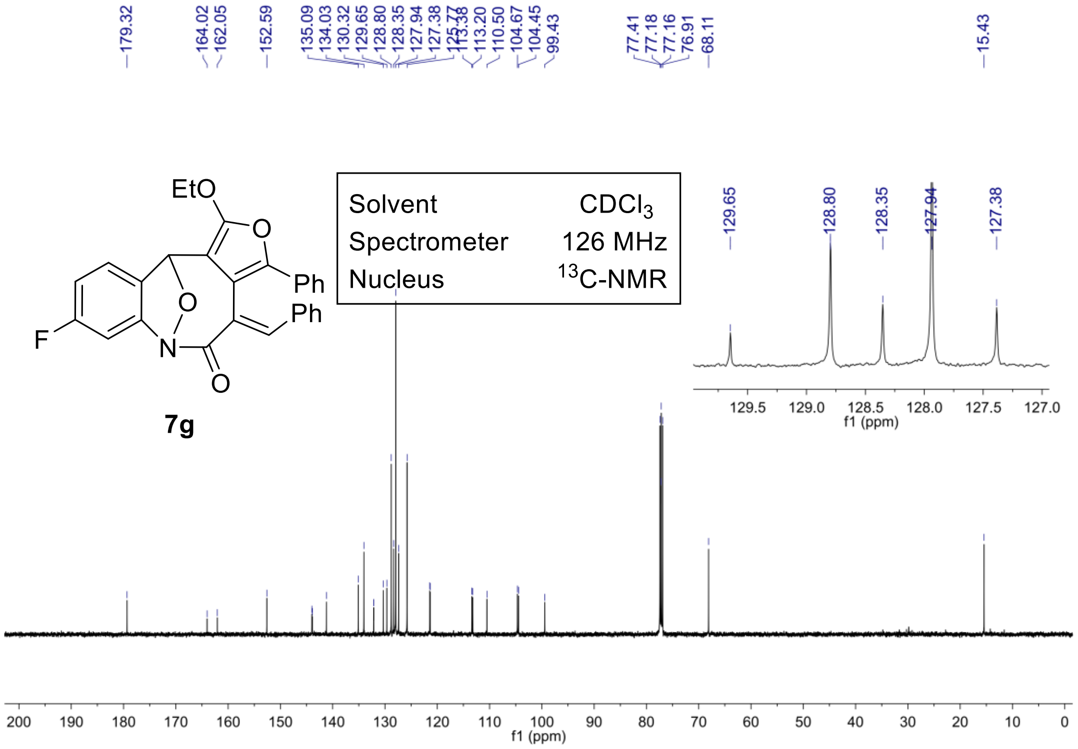
179.32
164.02
162.05
152.59
135.09
134.03
130.32
129.65
128.80
128.35
127.94
127.38
125.77
113.38
113.20
110.50
104.67
104.45
99.43
77.41
77.18
77.16
76.91
68.11
15.43
EtO
O
Ph
Ph
F
O
N
O
7g
Solvent CDCl$_3$
Spectrometer 126 MHz
Nucleus ^{13}C-NMR
f1 (ppm)

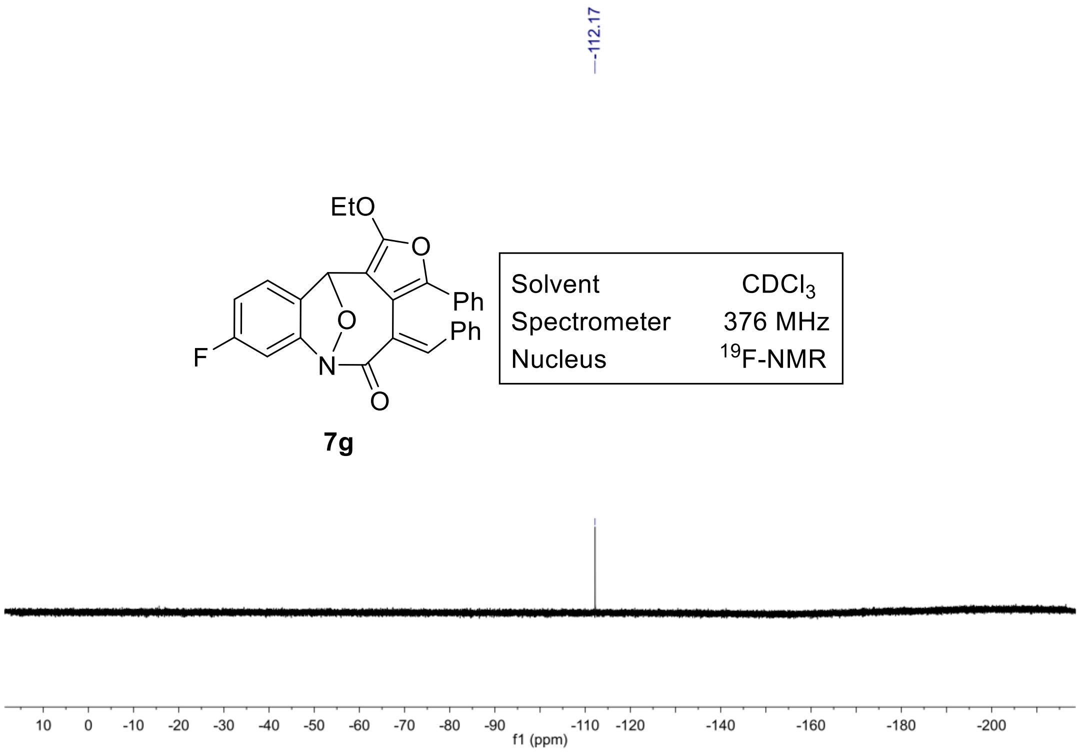
-112.17
EtO
O
Ph
Ph
F
O
N
O
7g
Solvent CDCl$_3$
Spectrometer 376 MHz
Nucleus ^{19}F-NMR
f1 (ppm)

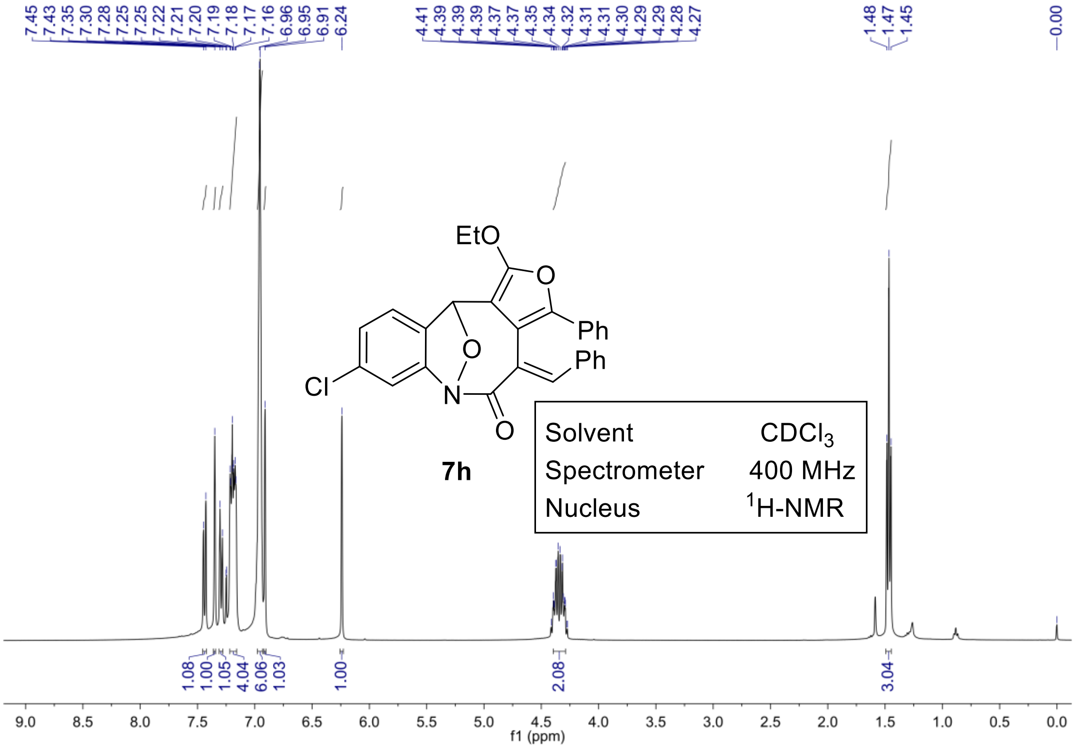

EtO
O
Ph
Ph
O
Cl
N
O
7h
Solvent CDCl3
Spectrometer 400 MHz
Nucleus 1H-NMR
f1 (ppm)

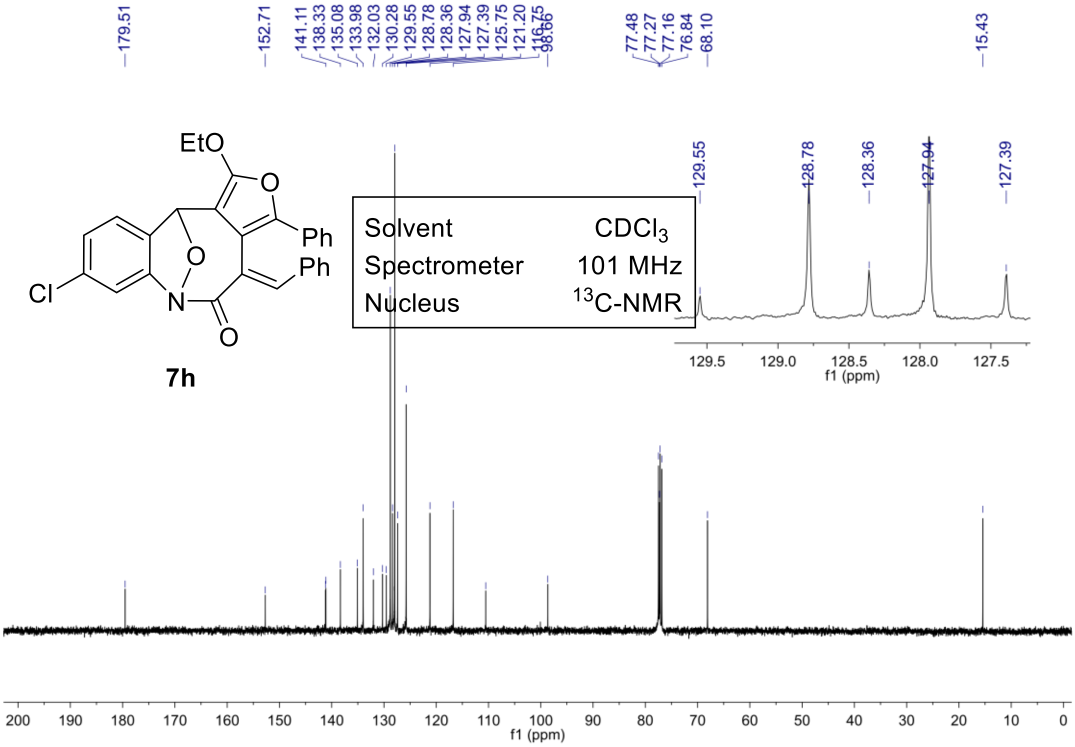

EtO
O
Ph
Ph
O
Cl
N
O
7h
Solvent CDCl3
Spectrometer 101 MHz
Nucleus 13C-NMR
f1 (ppm)

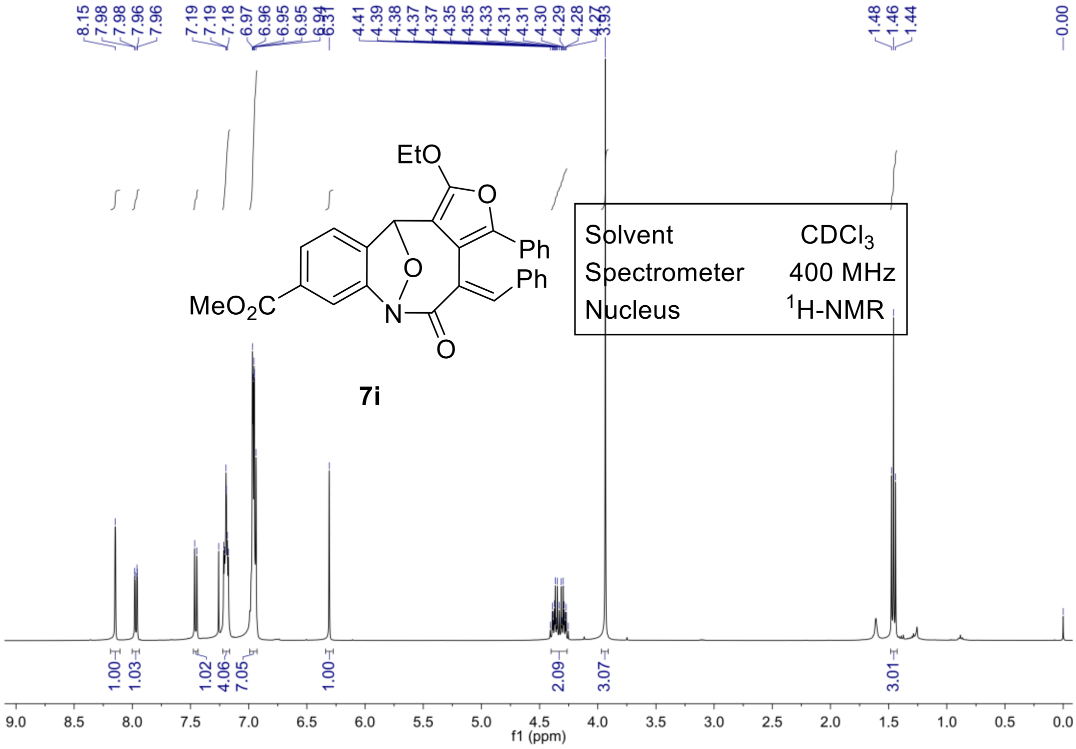
EtO
O
Ph
Ph
O
MeO2C
N
O
7i
Solvent CDCl3
Spectrometer 400 MHz
Nucleus 1H-NMR
f1 (ppm)

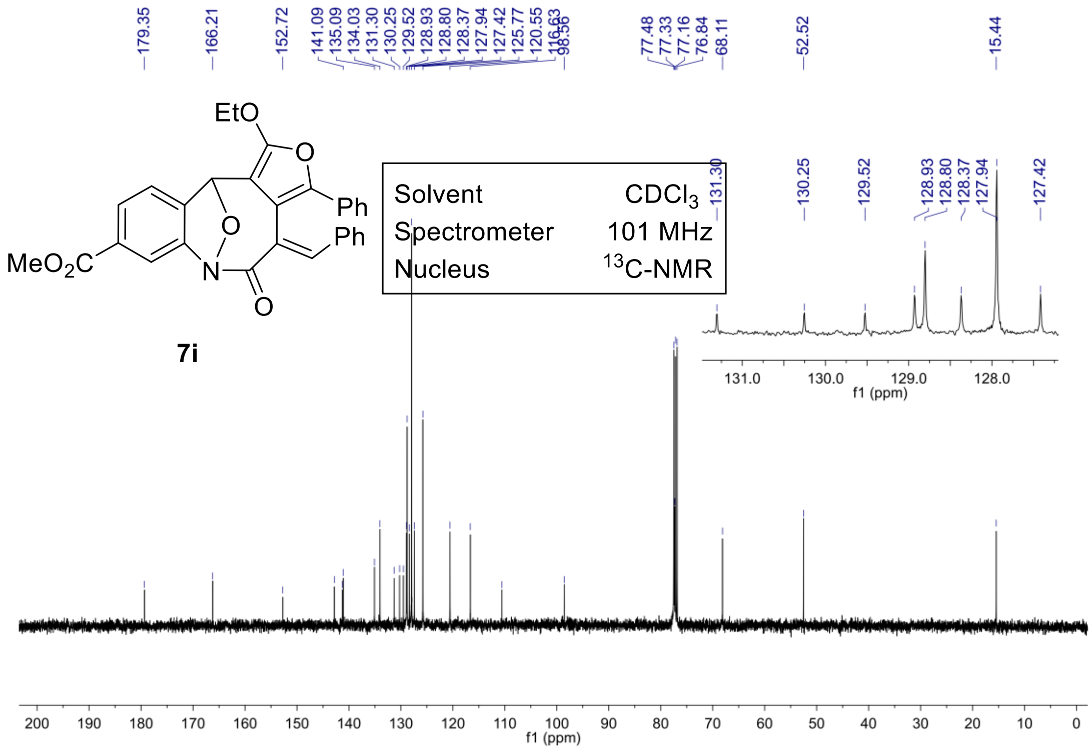
EtO
O
Ph
Ph
O
MeO2C
N
O
7i
Solvent CDCl3
Spectrometer 101 MHz
Nucleus 13C-NMR
f1 (ppm)

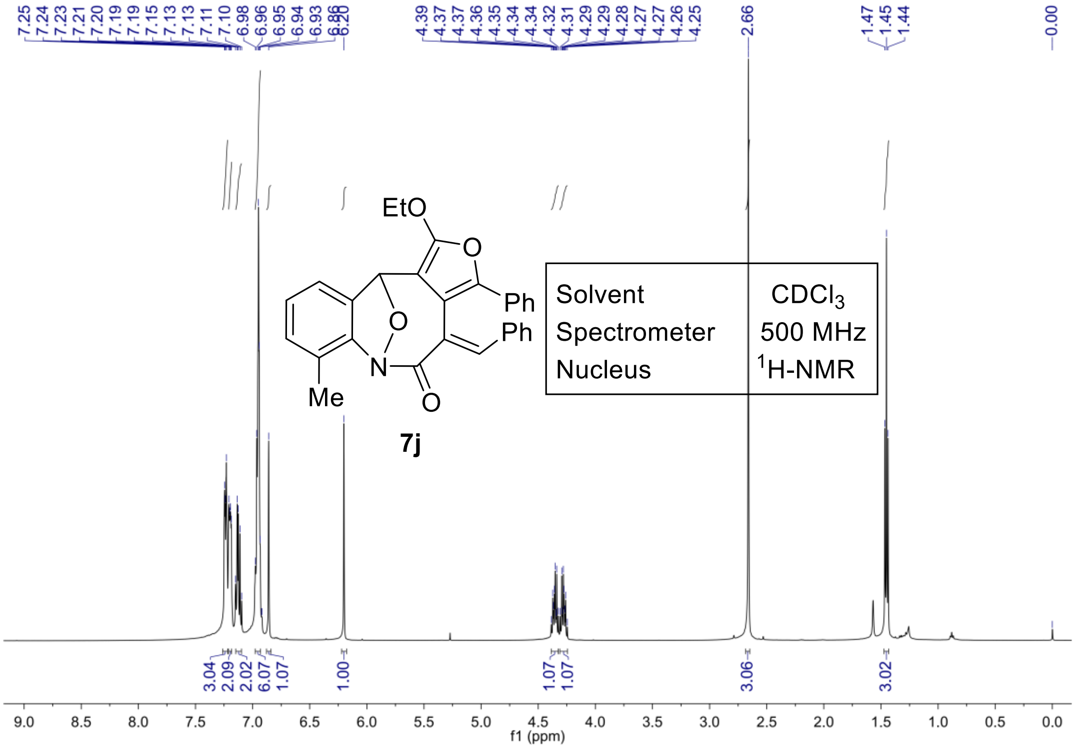
7.25 7.24 7.23 7.21 7.20 7.19 7.19 7.15 7.13 7.13 7.11 7.10 6.98 6.96 6.95 6.94 6.93
4.39 4.37 4.37 4.36 4.35 4.34 4.32 4.31 4.29 4.29 4.28 4.27 4.27 4.26 4.25
2.66
1.47 1.45 1.44
0.00
EtO
O
Ph
Ph
O
N
Me
O
7j
Solvent CDCl3
Spectrometer 500 MHz
Nucleus 1H-NMR
3.04 2.09 2.02 6.07 1.07
1.00
1.07 1.07
3.06
3.02
9.0 8.5 8.0 7.5 7.0 6.5 6.0 5.5 5.0 4.5 4.0 3.5 3.0 2.5 2.0 1.5 1.0 0.5 0.0
f1 (ppm)

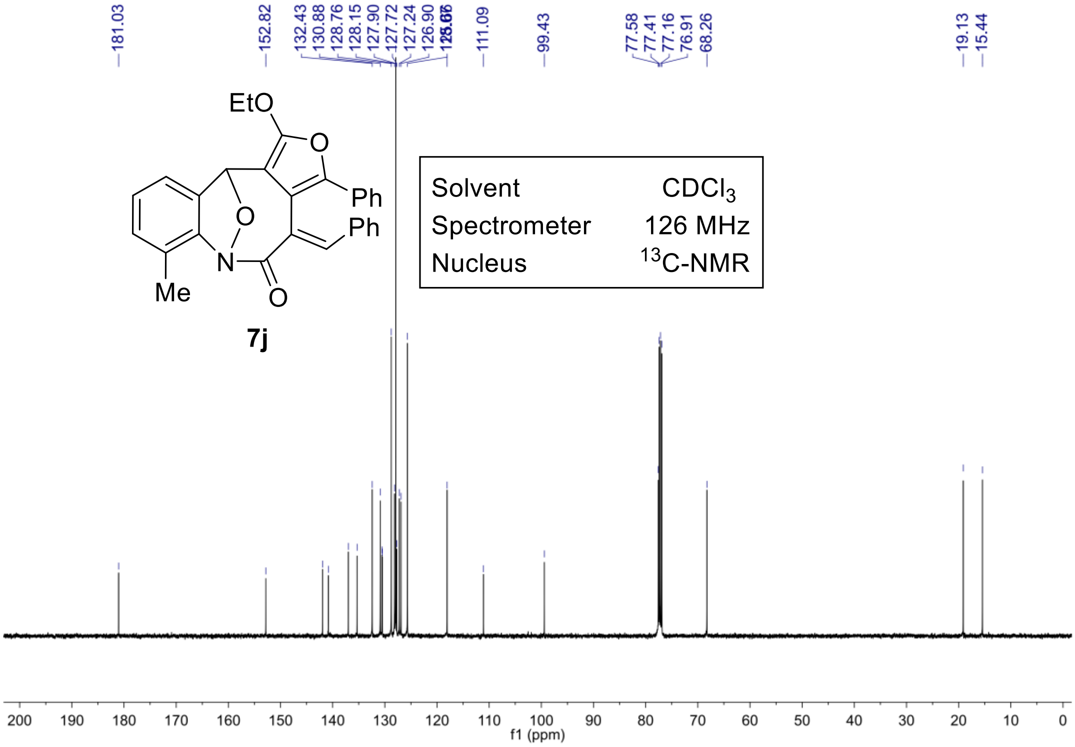
181.03
152.82
132.43 130.88 128.76 128.15 127.90 127.72 127.24 126.90
111.09
99.43
77.58 77.41 77.16 76.91 68.26
19.13 15.44
EtO
O
Ph
Ph
O
N
Me
O
7j
Solvent CDCl3
Spectrometer 126 MHz
Nucleus 13C-NMR
200 190 180 170 160 150 140 130 120 110 100 90 80 70 60 50 40 30 20 10 0
f1 (ppm)

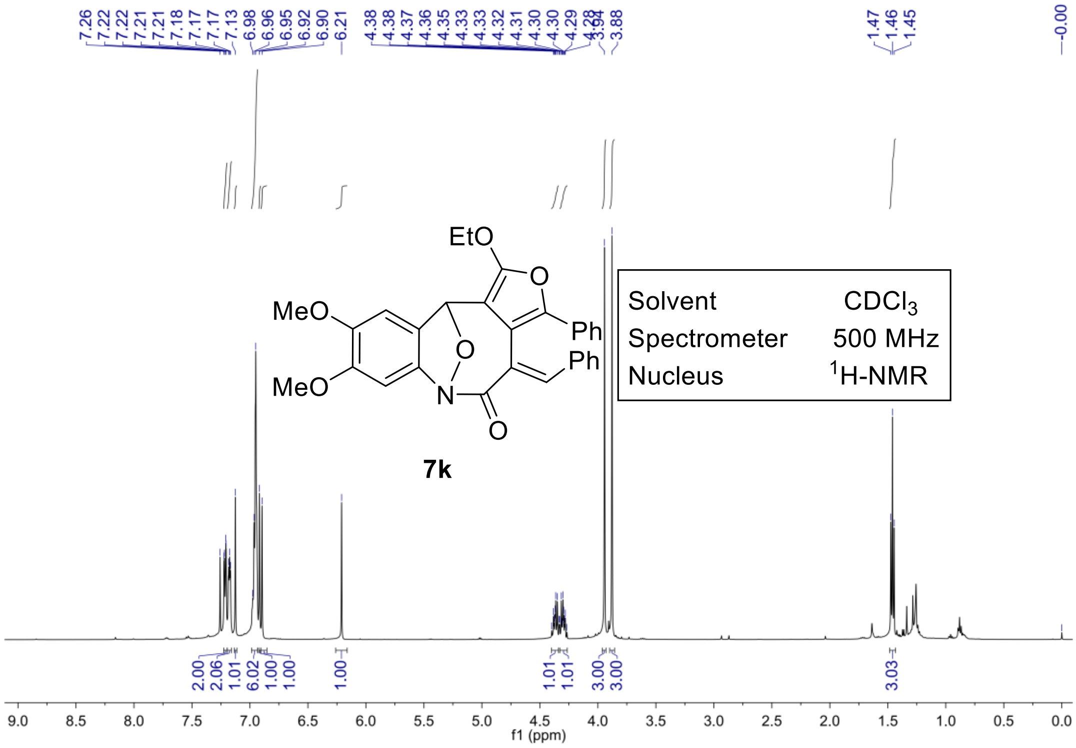
7.26 7.22 7.22 7.21 7.21 7.18 7.17 7.17 7.13 6.98 6.96 6.95 6.92 6.90 6.21 4.38 4.38 4.37 4.36 4.35 4.33 4.33 4.32 4.31 4.30 4.30 4.29 4.28 3.94 3.88 1.47 1.46 1.45 0.00
EtO
O
MeO
MeO
O
N
Ph
Ph
O
7k
Solvent CDCl3
Spectrometer 500 MHz
Nucleus 1H-NMR
2.00 2.06 1.01 6.02 1.00 1.00 1.00 1.01 1.01 3.00 3.00 3.03
9.0 8.5 8.0 7.5 7.0 6.5 6.0 5.5 5.0 4.5 4.0 3.5 3.0 2.5 2.0 1.5 1.0 0.5 0.0
f1 (ppm)

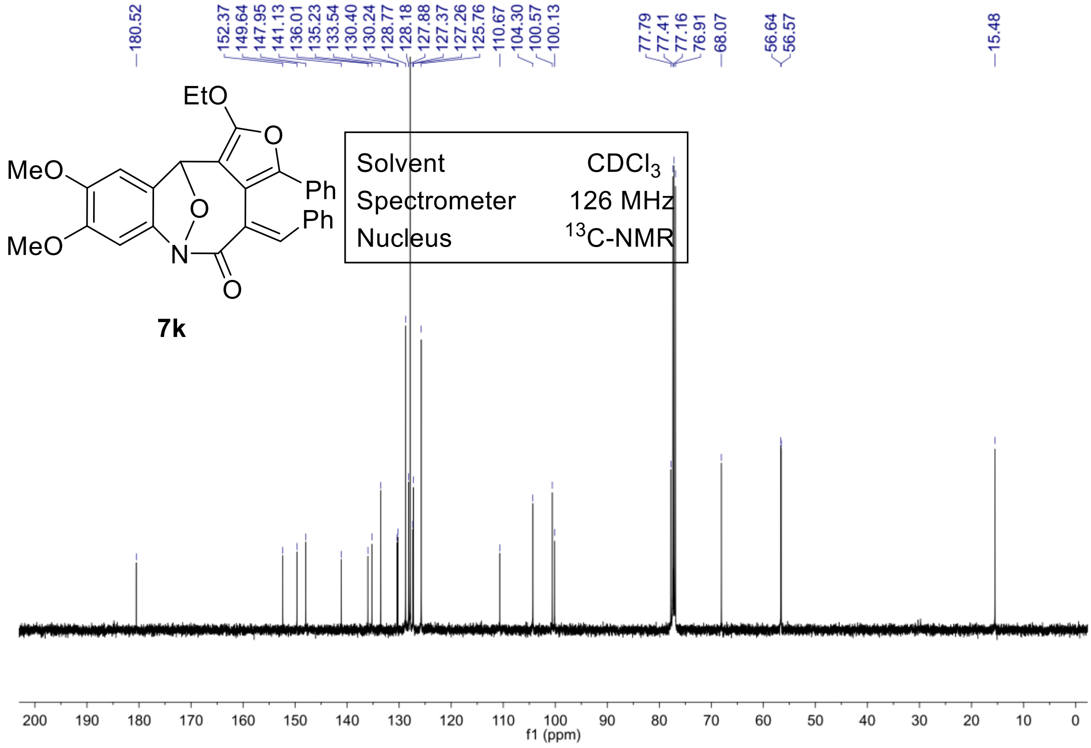
180.52 152.37 149.64 147.95 141.13 136.01 135.23 133.54 130.40 130.24 128.77 128.18 127.88 127.37 127.26 125.76 110.67 104.30 100.57 100.13 77.79 77.41 77.16 76.91 68.07 56.64 56.57 15.48
EtO
O
MeO
MeO
O
N
Ph
Ph
O
7k
Solvent CDCl3
Spectrometer 126 MHz
Nucleus 13C-NMR
200 190 180 170 160 150 140 130 120 110 100 90 80 70 60 50 40 30 20 10 0
f1 (ppm)

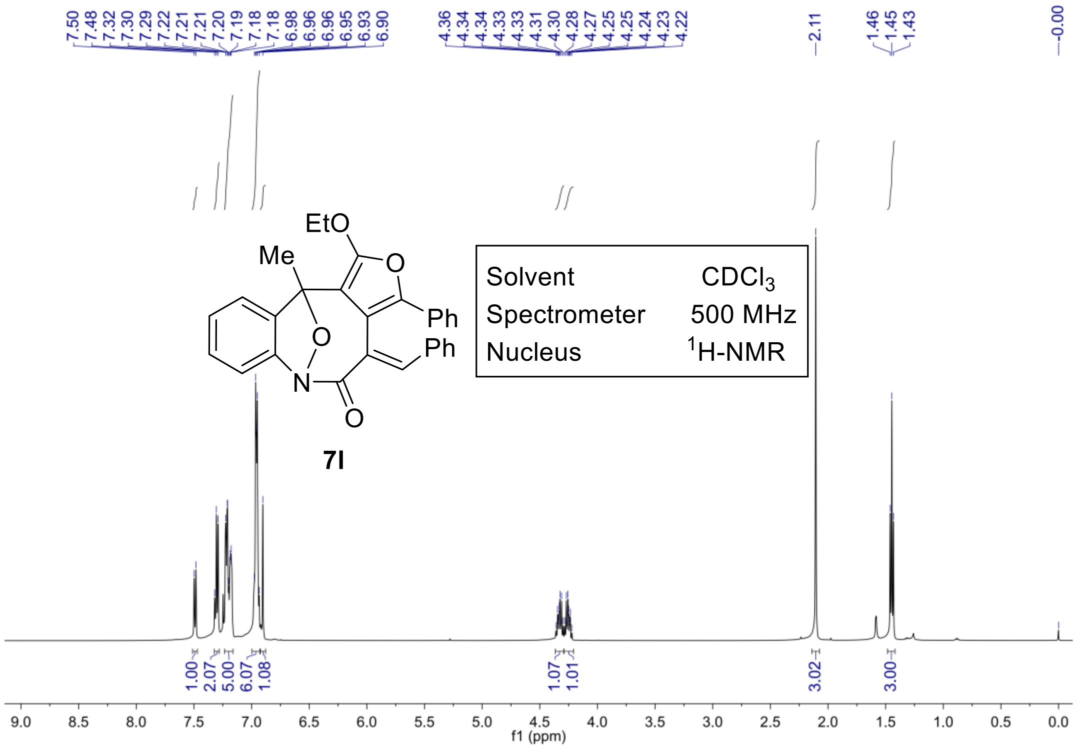

EtO
Me
O
Ph
Ph
O
N
O
7l
Solvent CDCl3
Spectrometer 500 MHz
Nucleus 1H-NMR
f1 (ppm)

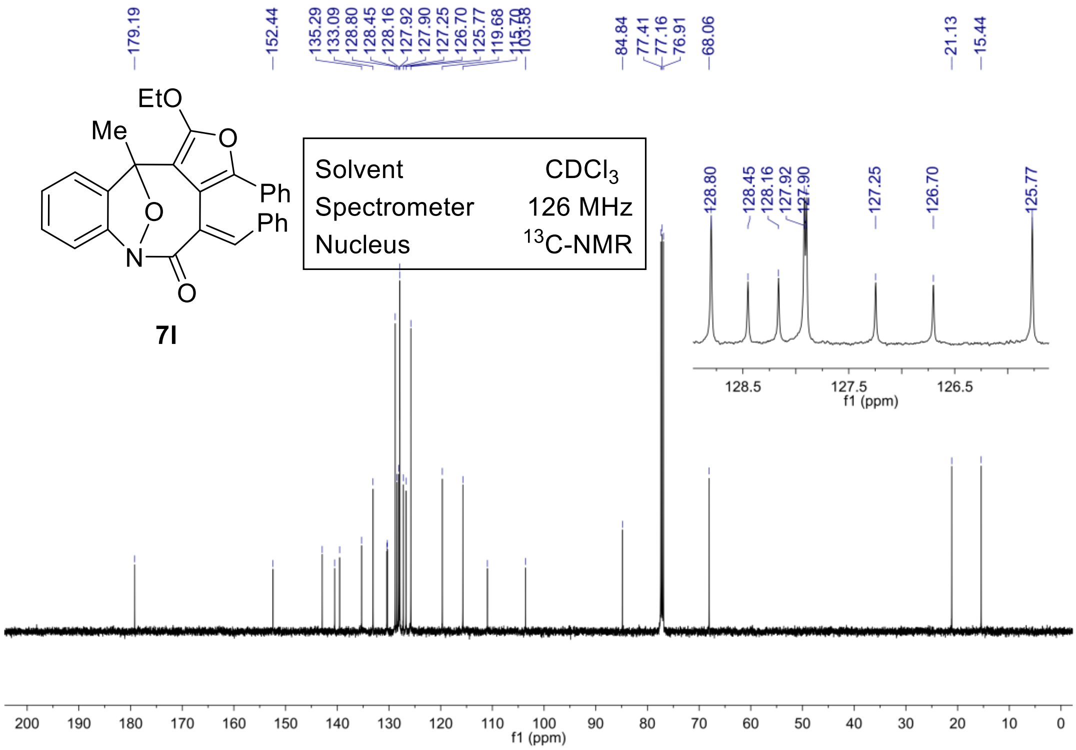

EtO
Me
O
Ph
Ph
O
N
O
7l
Solvent CDCl3
Spectrometer 126 MHz
Nucleus 13C-NMR
f1 (ppm)

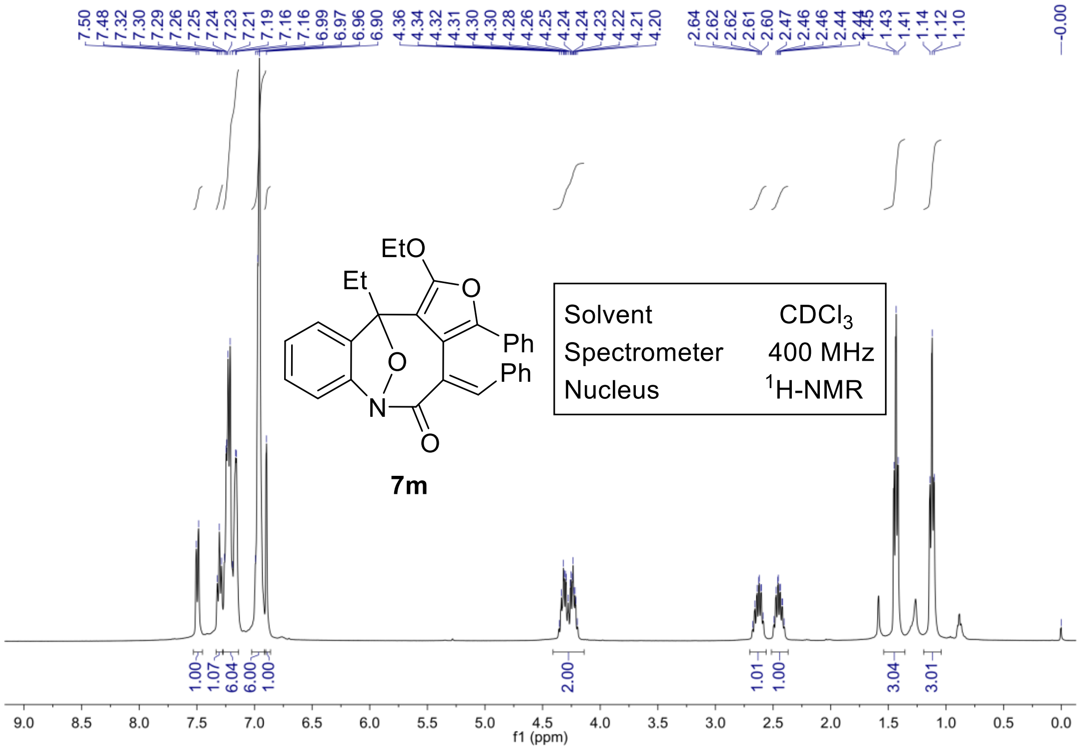

EtO
Et
O
Ph
Ph
O
N
O
7m
Solvent CDCl3
Spectrometer 400 MHz
Nucleus 1H-NMR
f1 (ppm)

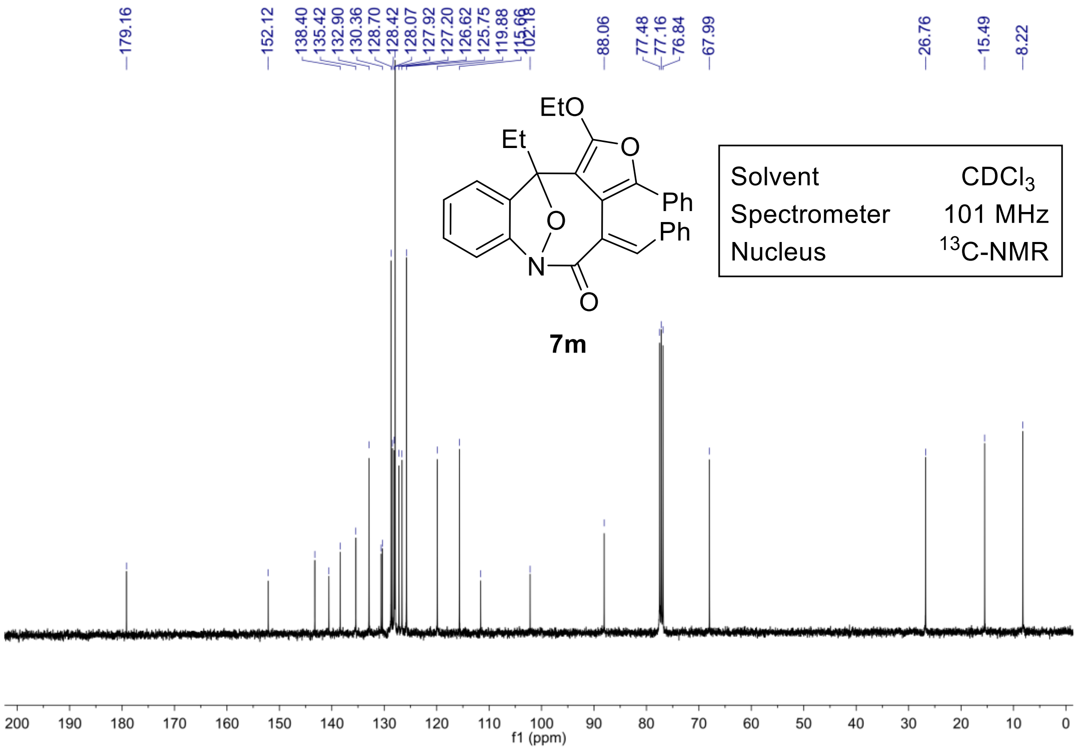

EtO
Et
O
Ph
Ph
O
N
O
7m
Solvent CDCl3
Spectrometer 101 MHz
Nucleus 13C-NMR
f1 (ppm)

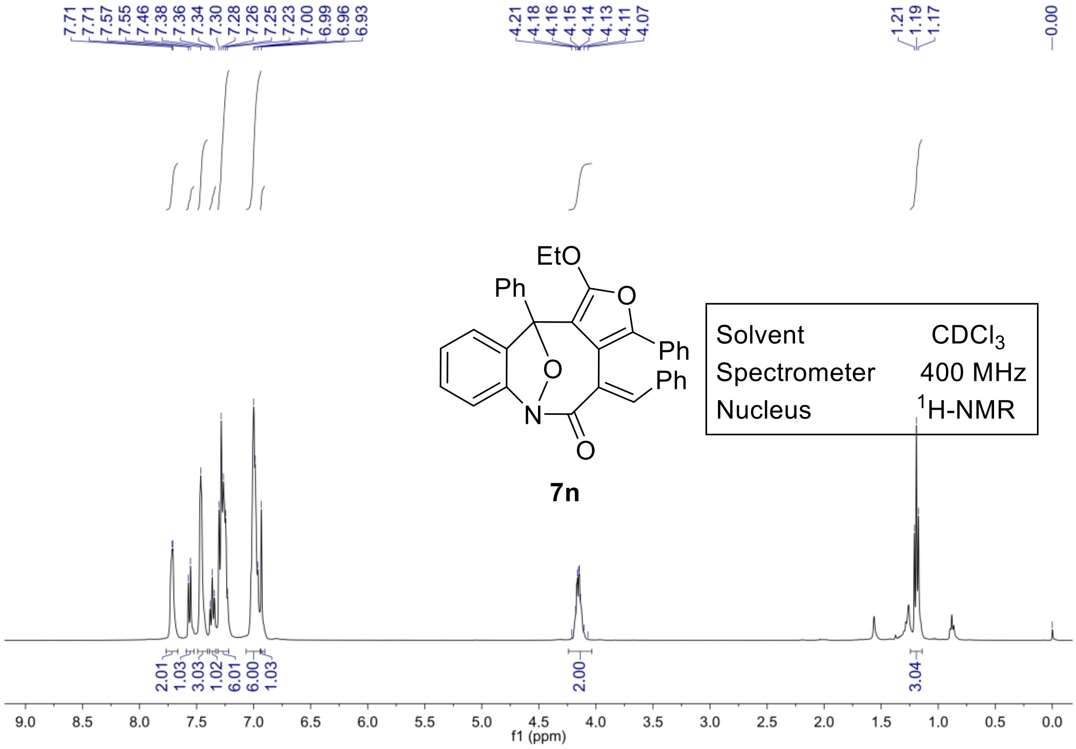

7.71
7.71
7.57
7.55
7.46
7.38
7.36
7.34
7.30
7.28
7.26
7.25
7.23
7.00
6.99
6.96
6.93
4.21
4.18
4.16
4.15
4.14
4.13
4.11
4.07
1.21
1.19
1.17
0.00
EtO
Ph
O
Ph
Ph
O
N
O
7n
Solvent CDCl3
Spectrometer 400 MHz
Nucleus 1H-NMR
2.01
1.03
3.03
1.02
6.01
6.00
1.03
2.00
3.04
f1 (ppm)

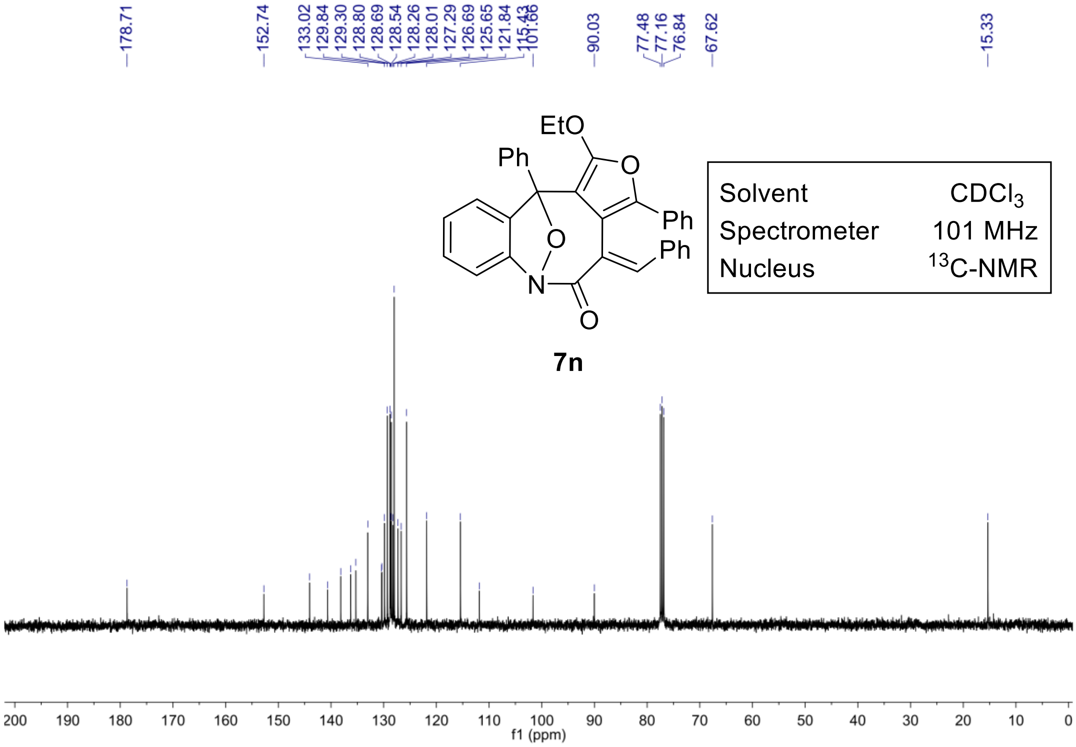

178.71
152.74
133.02
129.84
129.30
128.80
128.69
128.54
128.26
128.01
127.29
126.69
125.65
121.84
115.43
101.66
90.03
77.48
77.16
76.84
67.62
15.33
EtO
Ph
O
Ph
Ph
O
N
O
7n
Solvent CDCl3
Spectrometer 101 MHz
Nucleus 13C-NMR
f1 (ppm)

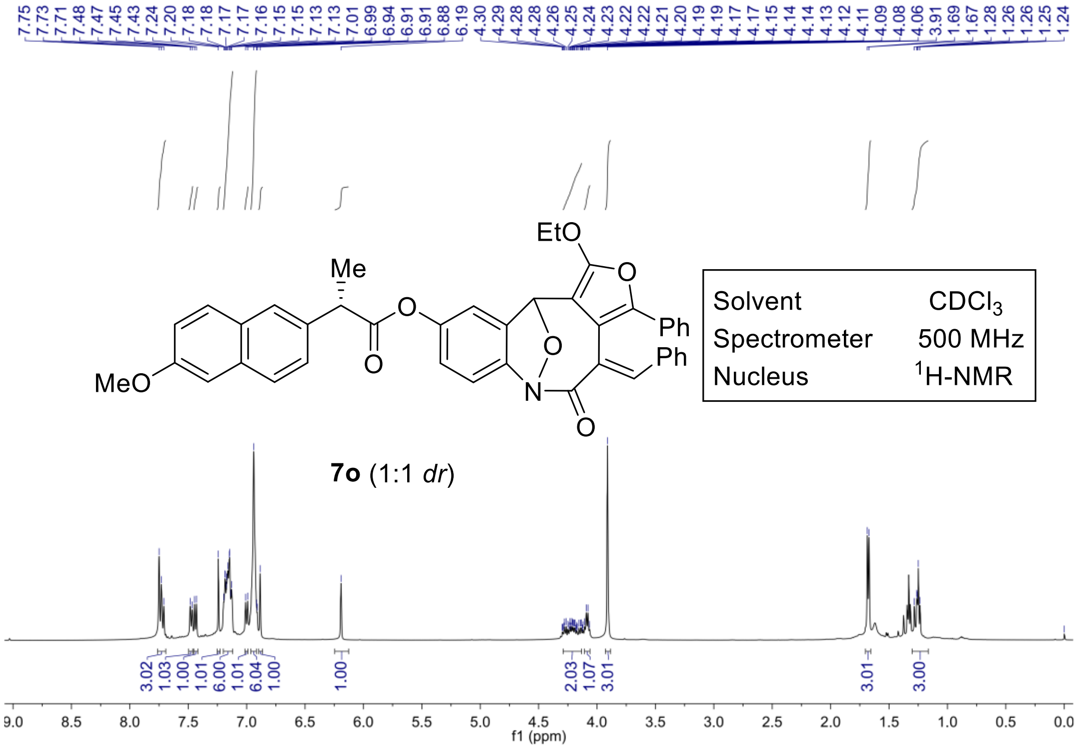
EtO
Me
O
O
O
O
Ph
Ph
MeO
N
O
7o (1:1 dr)
Solvent CDCl3
Spectrometer 500 MHz
Nucleus 1H-NMR
f1 (ppm)

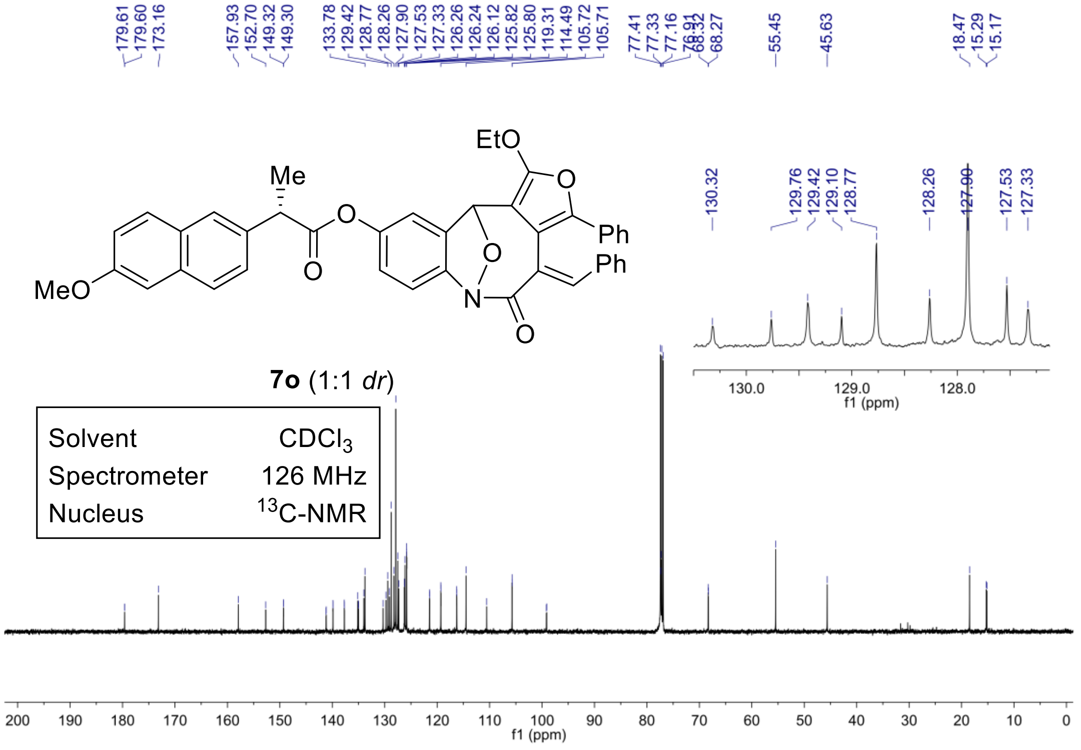
EtO
Me
MeO
Ph
Ph
N
7o (1:1 dr)
Solvent CDCl3
Spectrometer 126 MHz
Nucleus 13C-NMR
f1 (ppm)

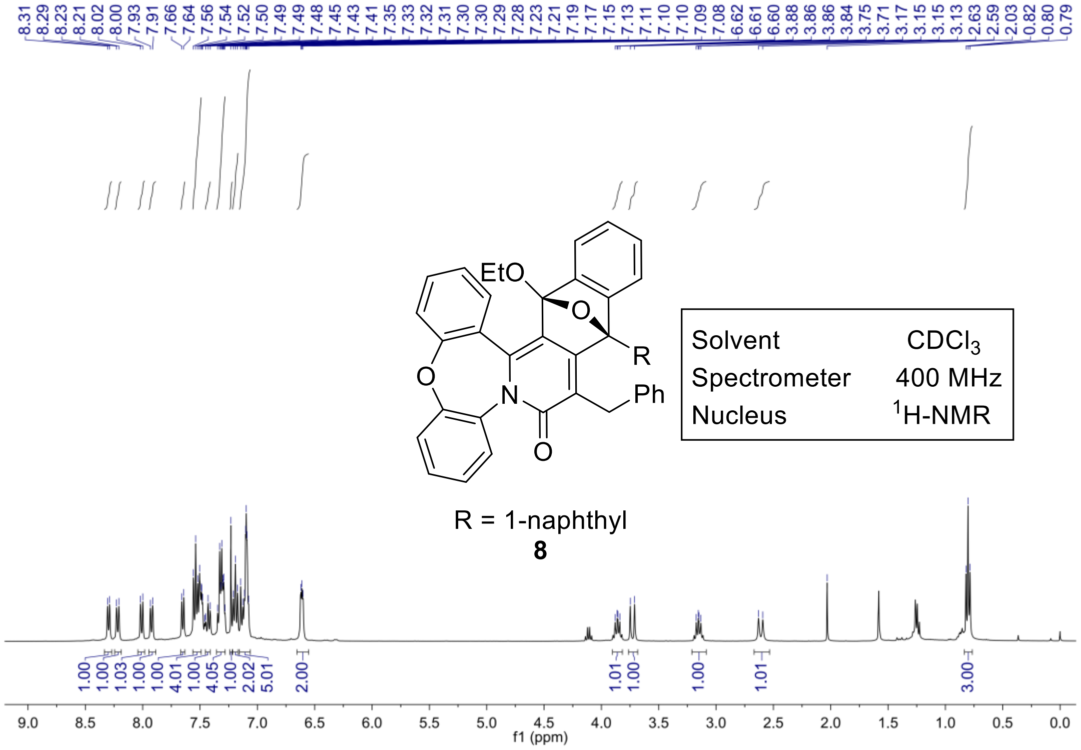
EtO
O
R
Ph
N
O
Solvent CDCl3
Spectrometer 400 MHz
Nucleus 1H-NMR
R = 1-naphthyl
8
f1 (ppm)

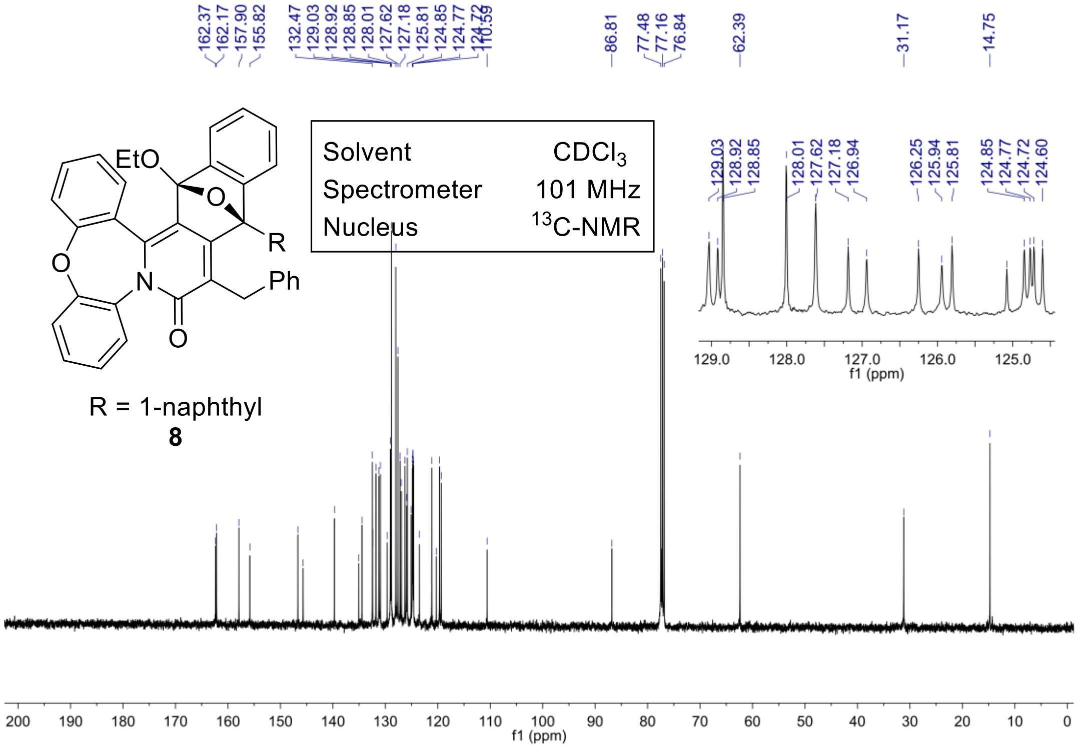
EtO
O
R
Ph
N
O
Solvent CDCl3
Spectrometer 101 MHz
Nucleus 13C-NMR
R = 1-naphthyl
8
f1 (ppm)

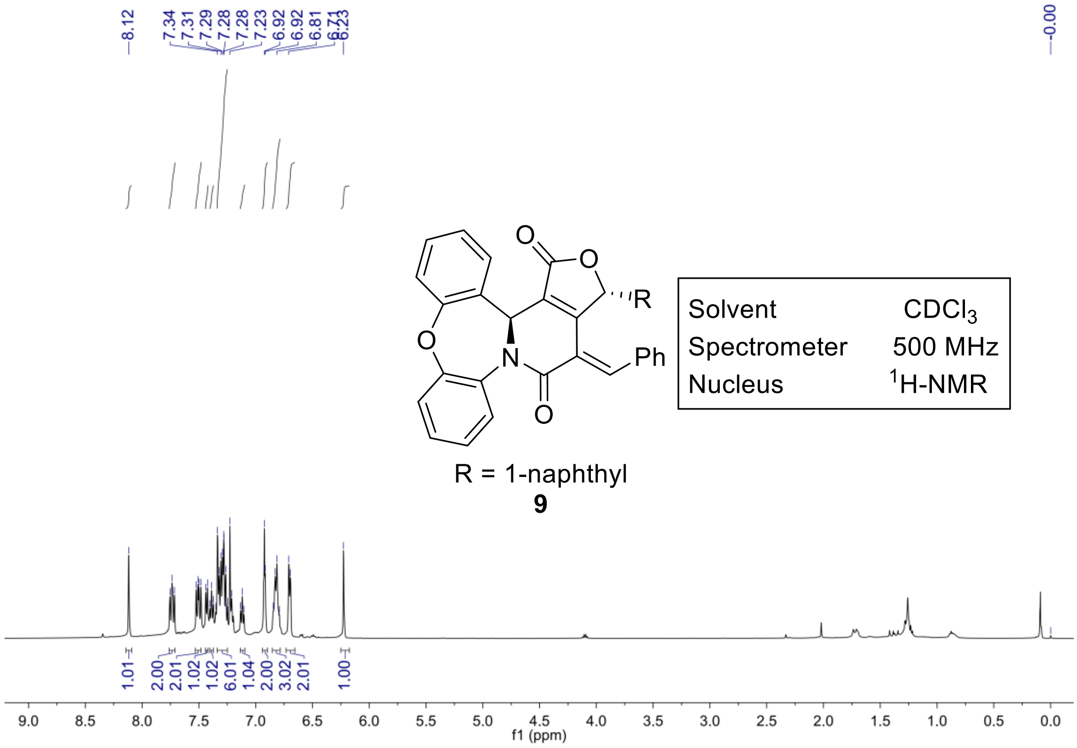
O
O
R
O
N
Ph
O
Solvent CDCl3
Spectrometer 500 MHz
Nucleus 1H-NMR
R = 1-naphthyl
9
f1 (ppm)

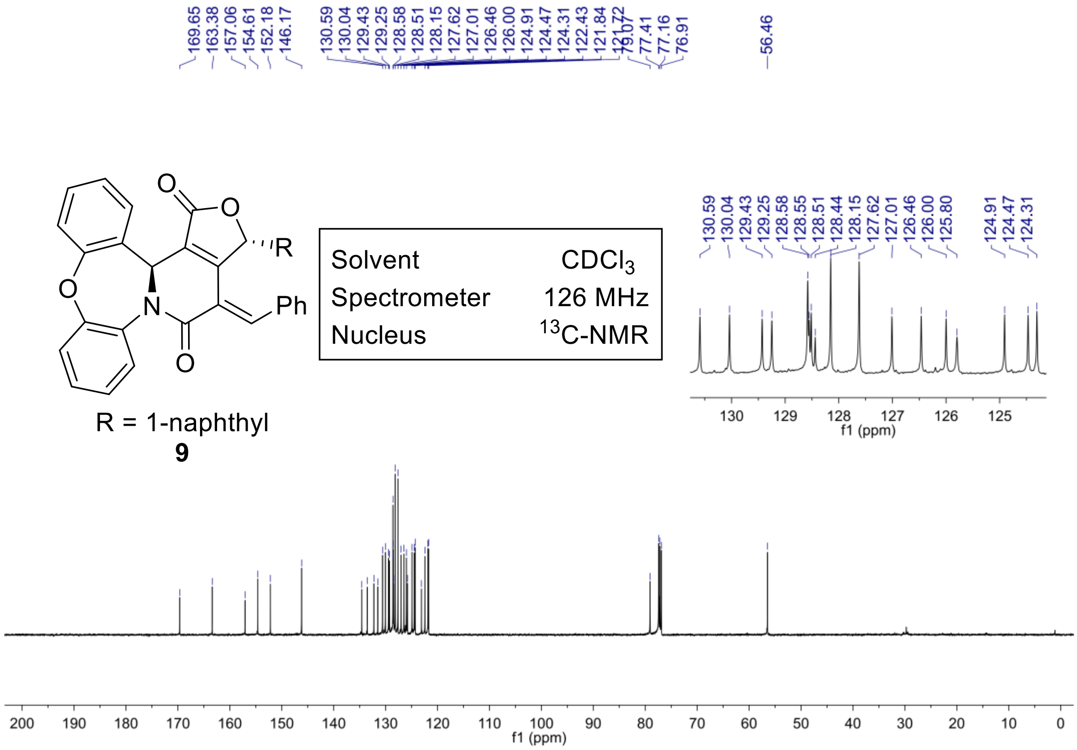
O
O
R
O
N
Ph
O
Solvent CDCl3
Spectrometer 126 MHz
Nucleus 13C-NMR
R = 1-naphthyl
9
f1 (ppm)

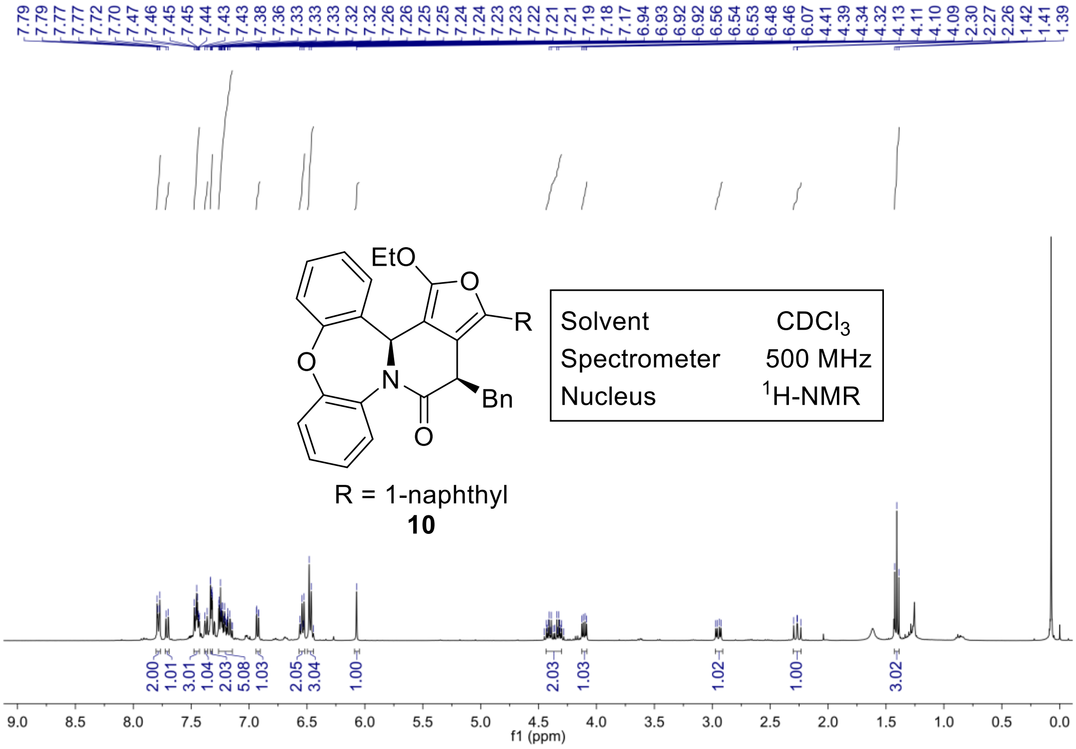
7.79 7.79 7.77 7.77 7.72 7.70 7.47 7.46 7.45 7.45 7.44 7.43 7.43 7.38 7.36 7.33 7.33 7.33 7.32 7.32 7.26 7.26 7.25 7.25 7.24 7.24 7.23 7.23 7.22 7.21 7.21 7.19 7.18 7.17 6.94 6.93 6.92 6.92 6.56 6.54 6.53 6.48 6.46 6.07 4.41 4.39 4.34 4.32 4.13 4.11 4.10 4.09 2.30 2.27 2.26 1.42 1.41 1.39
EtO
O
R
N
Bn
O
Solvent CDCl3
Spectrometer 500 MHz
Nucleus 1H-NMR
R = 1-naphthyl
10
2.00 1.01 3.01 1.04 2.03 5.08 1.03 2.05 3.04 1.00 2.03 1.03 1.02 1.00 3.02
f1 (ppm)

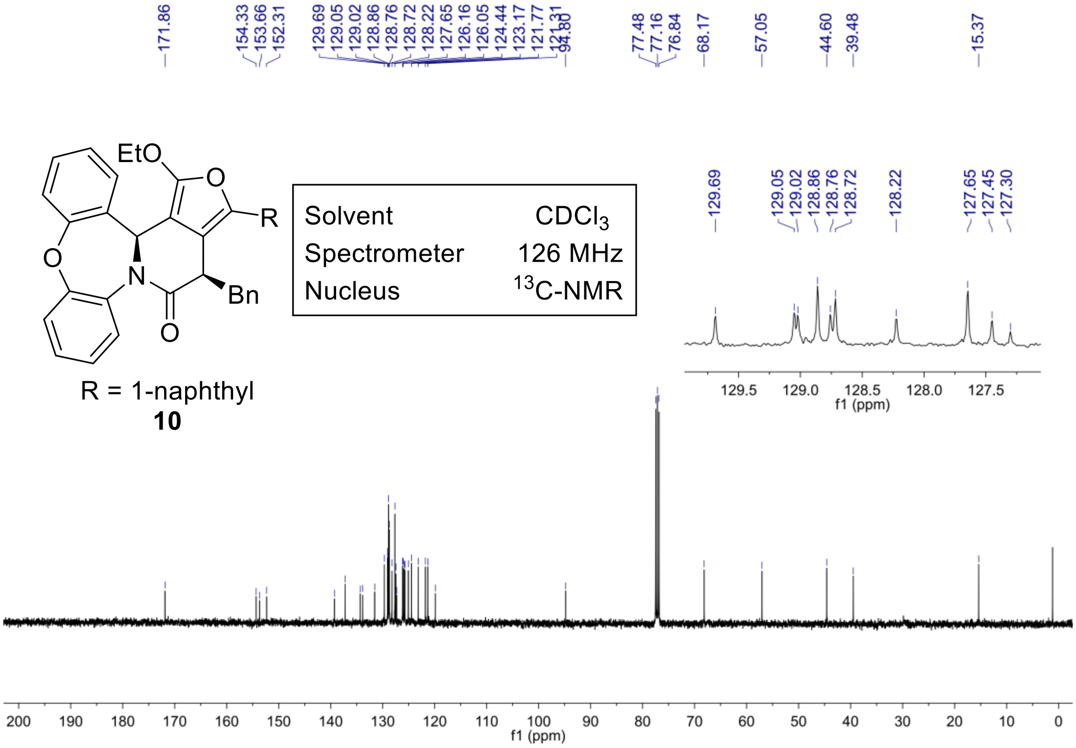
171.86 154.33 153.66 152.31 129.69 129.05 129.02 128.86 128.76 128.72 128.22 127.65 126.16 126.05 124.44 123.17 121.77 121.31 94.80 77.48 77.16 76.84 68.17 57.05 44.60 39.48 15.37
EtO
O
R
N
Bn
O
Solvent CDCl3
Spectrometer 126 MHz
Nucleus 13C-NMR
R = 1-naphthyl
10
129.69 129.05 129.02 128.86 128.76 128.72 128.22 127.65 127.45 127.30
f1 (ppm)

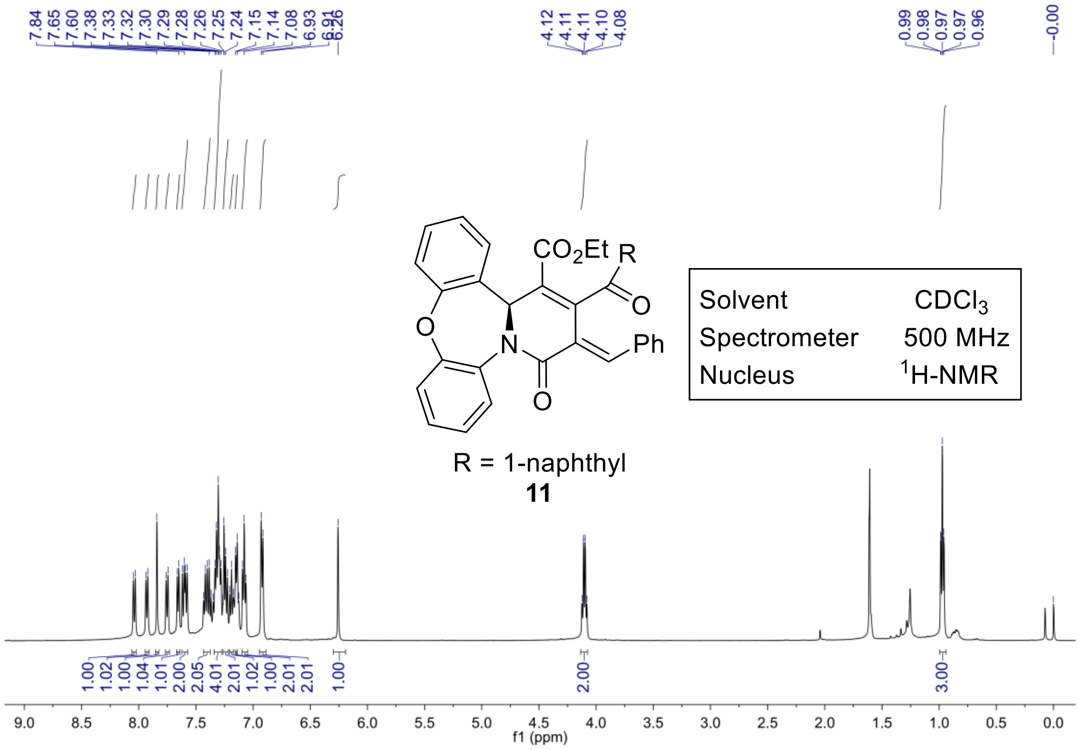

CO2Et
R
O
N
Ph
R = 1-naphthyl
11
Solvent CDCl3
Spectrometer 500 MHz
Nucleus 1H-NMR
f1 (ppm)

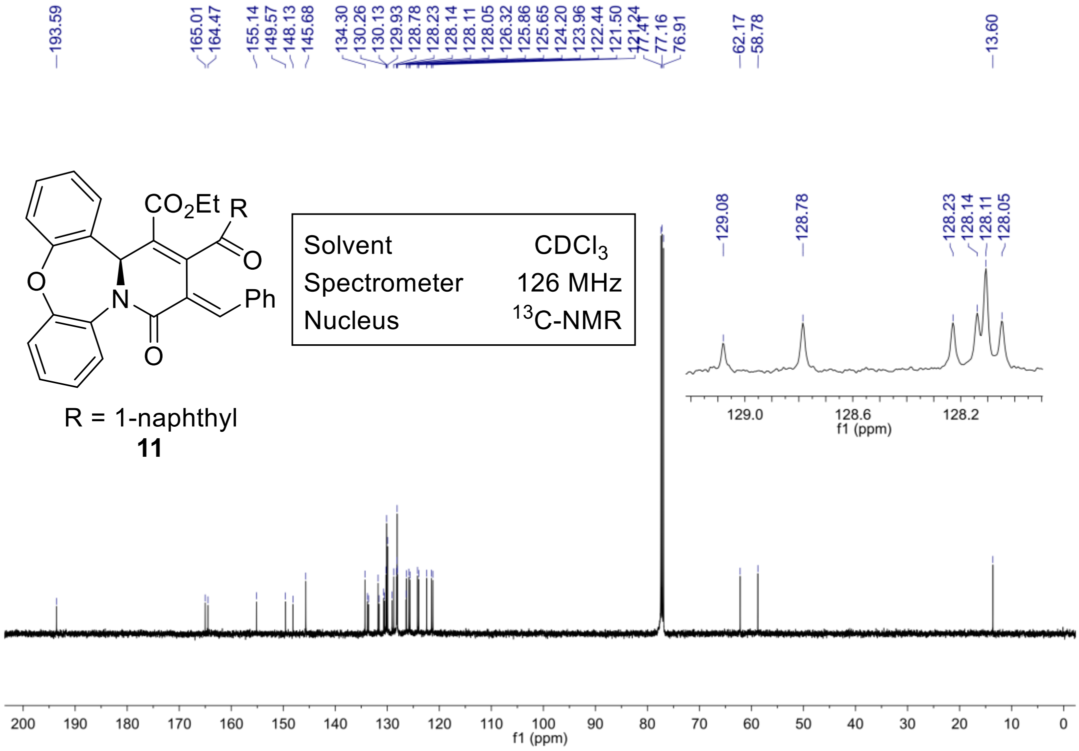

CO2Et
R
O
N
Ph
R = 1-naphthyl
11
Solvent CDCl3
Spectrometer 126 MHz
Nucleus 13C-NMR
f1 (ppm)

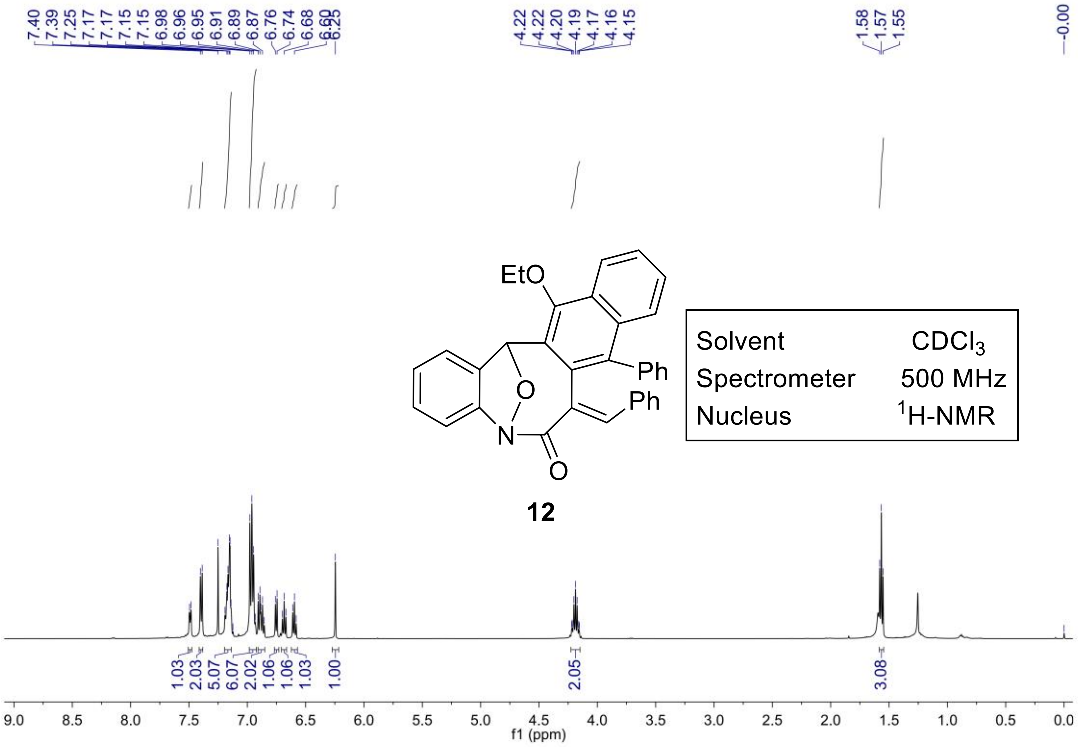
7.40 7.39 7.25 7.17 7.17 7.15 7.15 6.98 6.96 6.95 6.91 6.89 6.87 6.76 6.74 6.68 6.60 6.25
4.22 4.22 4.20 4.19 4.17 4.16 4.15
1.58 1.57 1.55
-0.00
EtO
Ph
Ph
O
N
O
12
Solvent CDCl3
Spectrometer 500 MHz
Nucleus 1H-NMR
1.03 2.03 5.07 6.07 2.02 1.06 1.06 1.03 1.00
2.05
3.08
f1 (ppm)

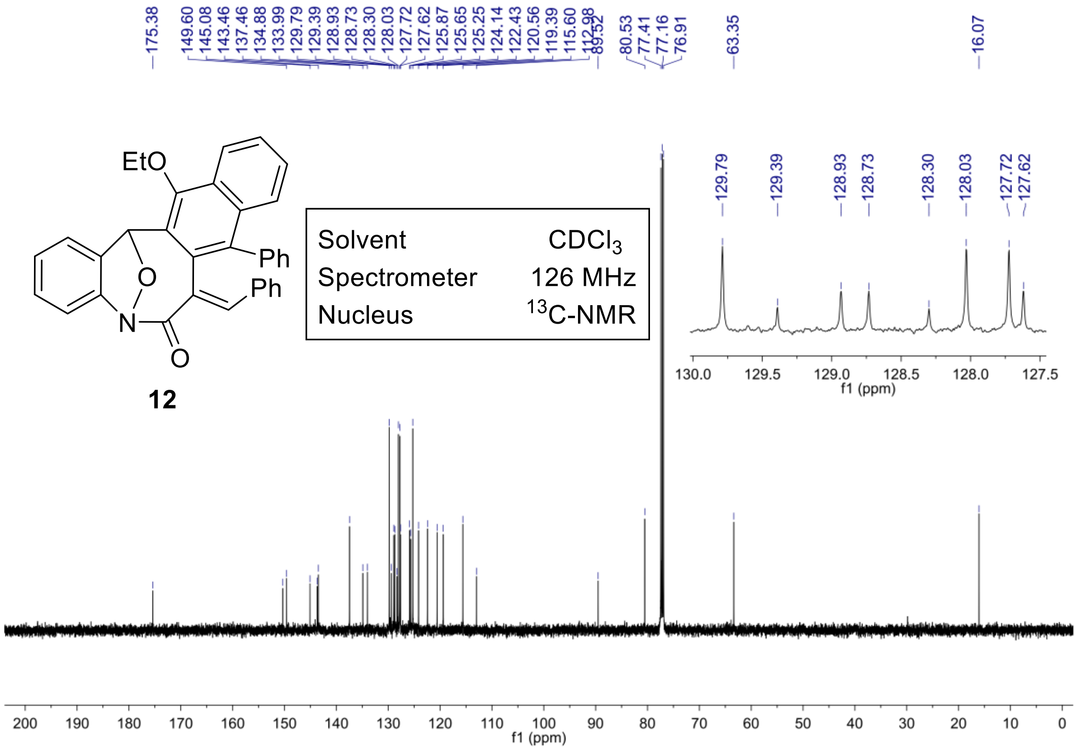
175.38
149.60 145.08 143.46 137.46 134.88 133.99 129.79 129.39 128.93 128.73 128.30 128.03 127.72 127.62 125.87 125.65 125.25 124.14 122.43 120.56 119.39 115.60 112.98 89.52
80.53 77.41 77.16 76.91
63.35
16.07
EtO
Ph
Ph
O
N
O
12
Solvent CDCl3
Spectrometer 126 MHz
Nucleus 13C-NMR
129.79 129.39 128.93 128.73 128.30 128.03 127.72 127.62
f1 (ppm)

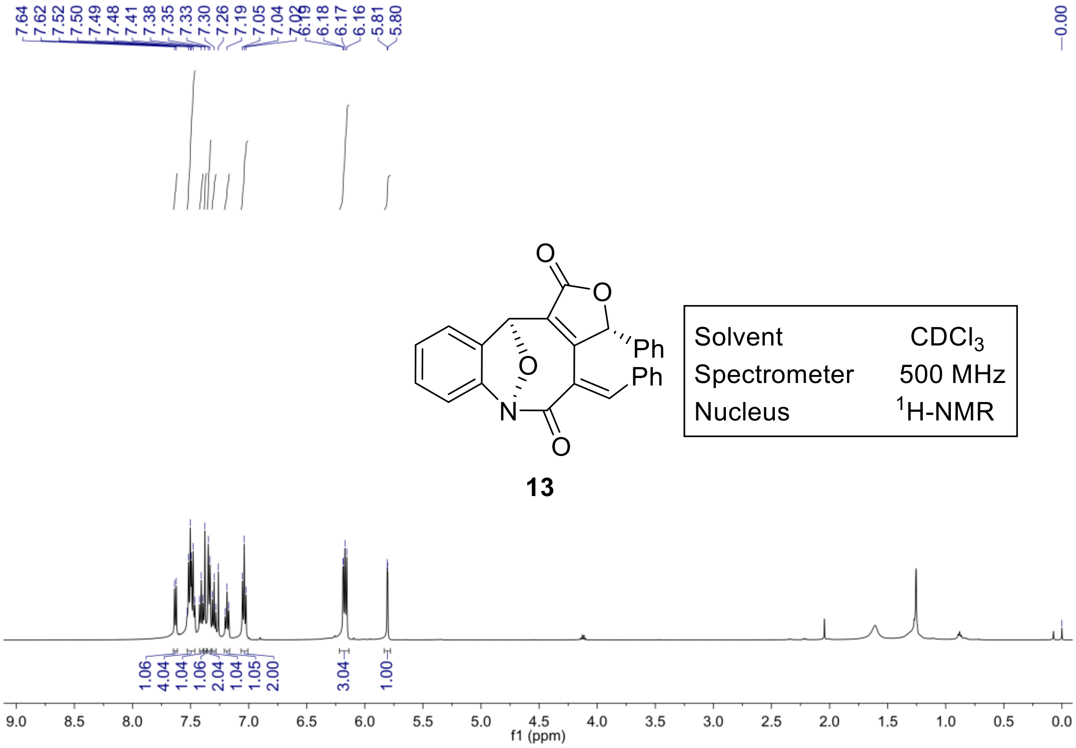
7.64
7.62
7.52
7.50
7.49
7.48
7.41
7.38
7.35
7.33
7.30
7.26
7.19
7.05
7.04
7.02
6.19
6.18
6.17
6.16
5.81
5.80
0.00
Ph
Ph
O
N
13
Solvent CDCl3
Spectrometer 500 MHz
Nucleus 1H-NMR
1.06
4.04
1.04
1.06
2.04
1.04
1.05
2.00
3.04
1.00
f1 (ppm)

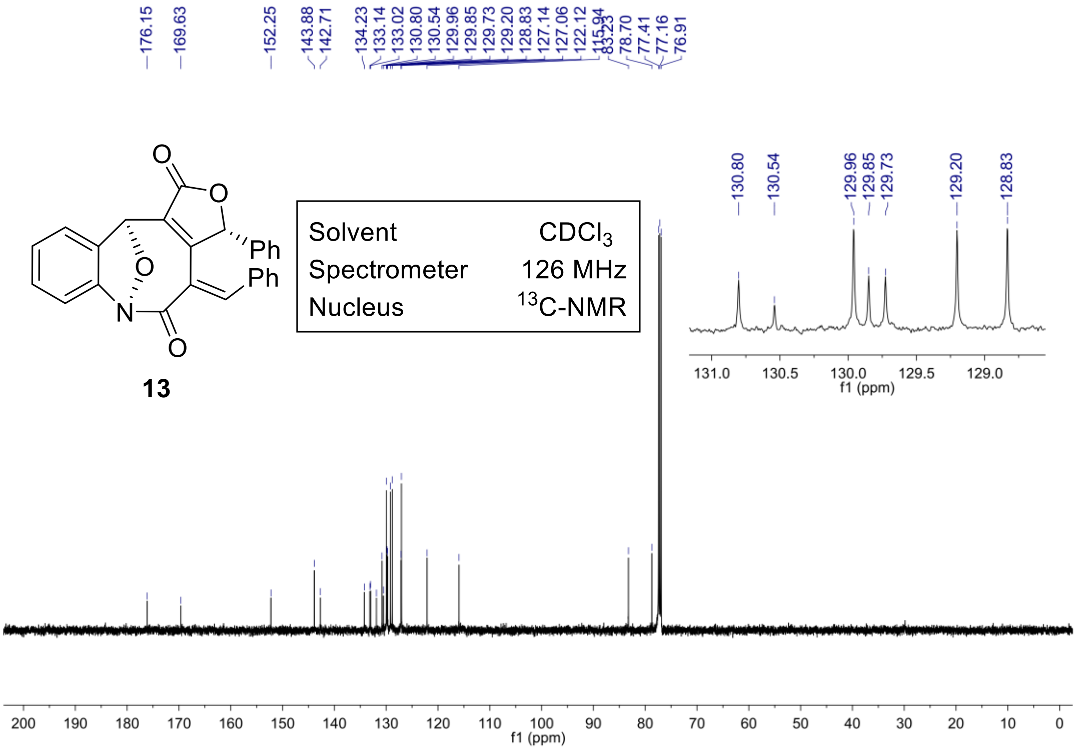
176.15
169.63
152.25
143.88
142.71
134.23
133.14
133.02
130.80
130.54
129.96
129.85
129.73
129.20
128.83
127.14
127.06
122.12
115.94
83.23
78.70
77.41
77.16
76.91
Ph
Ph
O
N
13
Solvent CDCl3
Spectrometer 126 MHz
Nucleus 13C-NMR
f1 (ppm)

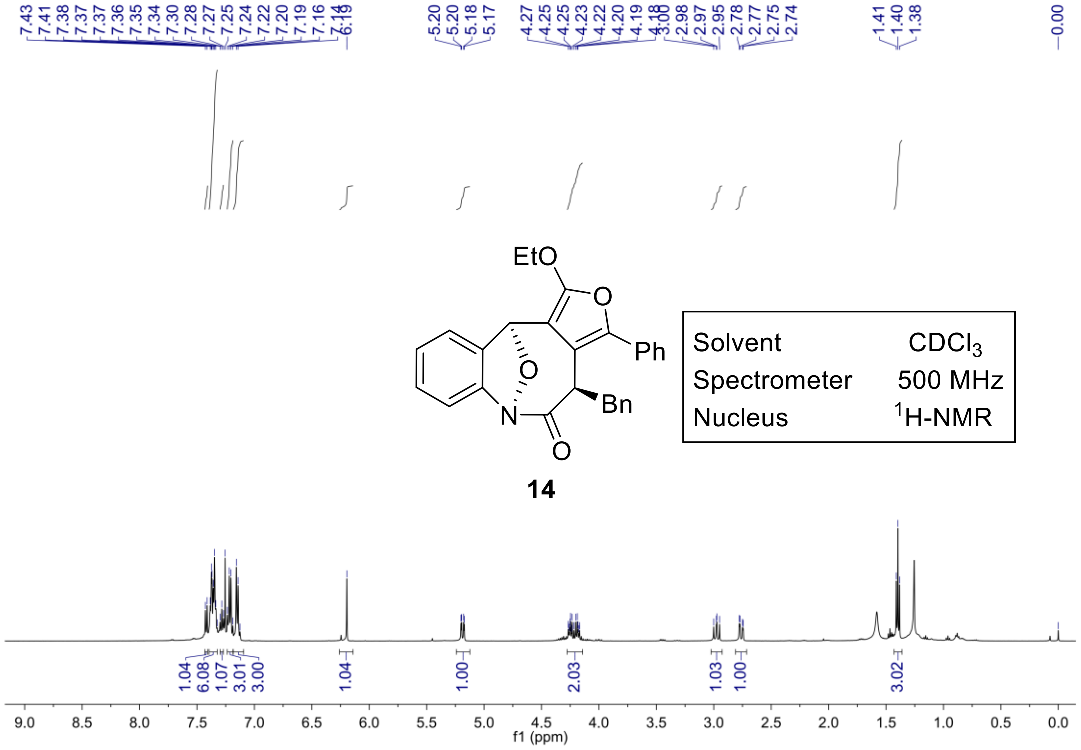
EtO
O
Ph
O
N
Bn
O
14
Solvent CDCl$_3$
Spectrometer 500 MHz
Nucleus ^{1}H-NMR
f1 (ppm)

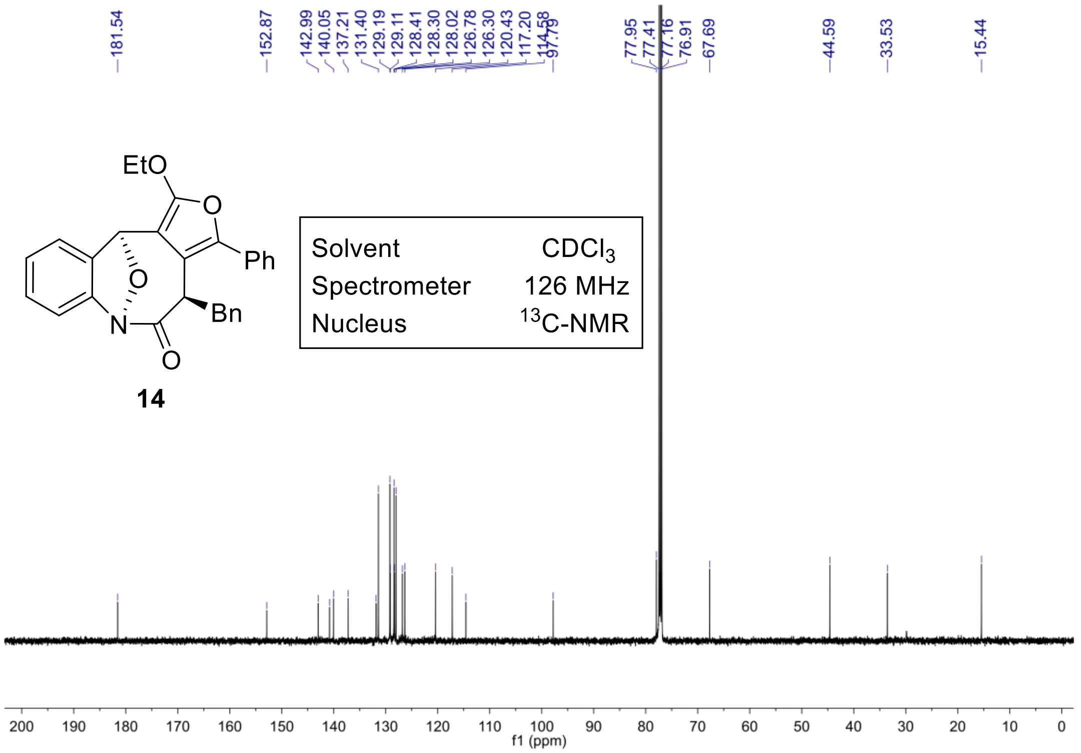
EtO
O
Ph
O
N
Bn
O
14
Solvent CDCl$_3$
Spectrometer 126 MHz
Nucleus ^{13}C-NMR
f1 (ppm)

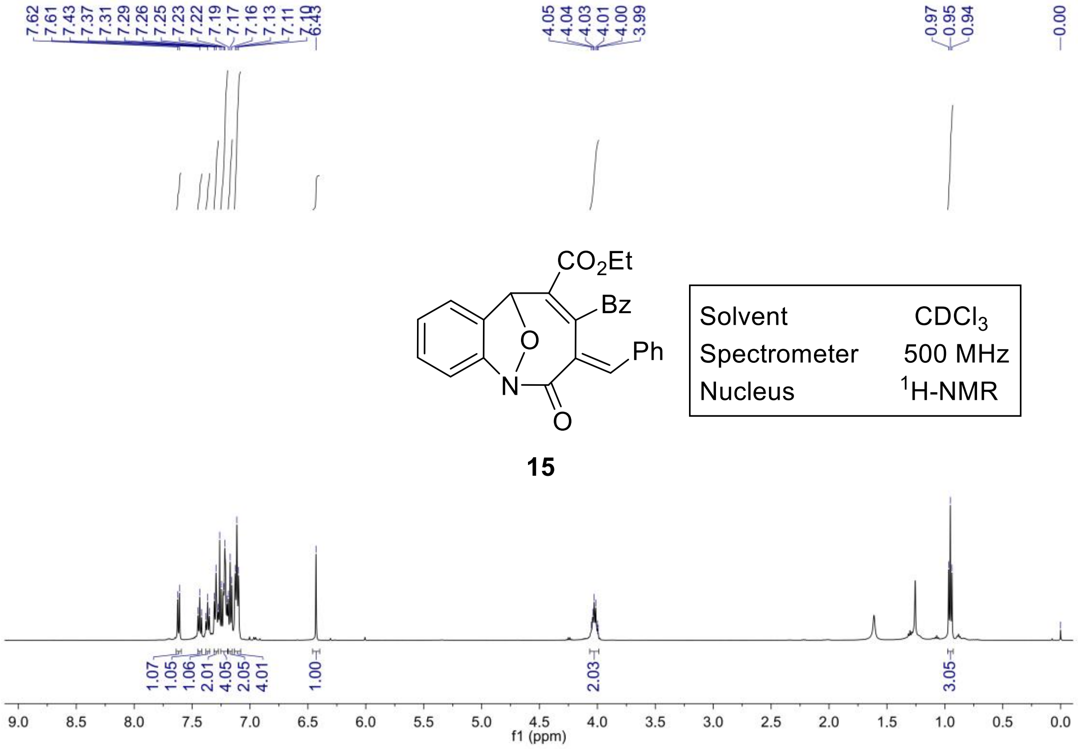
CO2Et
Bz
Ph
O
N
O
15
Solvent CDCl3
Spectrometer 500 MHz
Nucleus 1H-NMR
f1 (ppm)

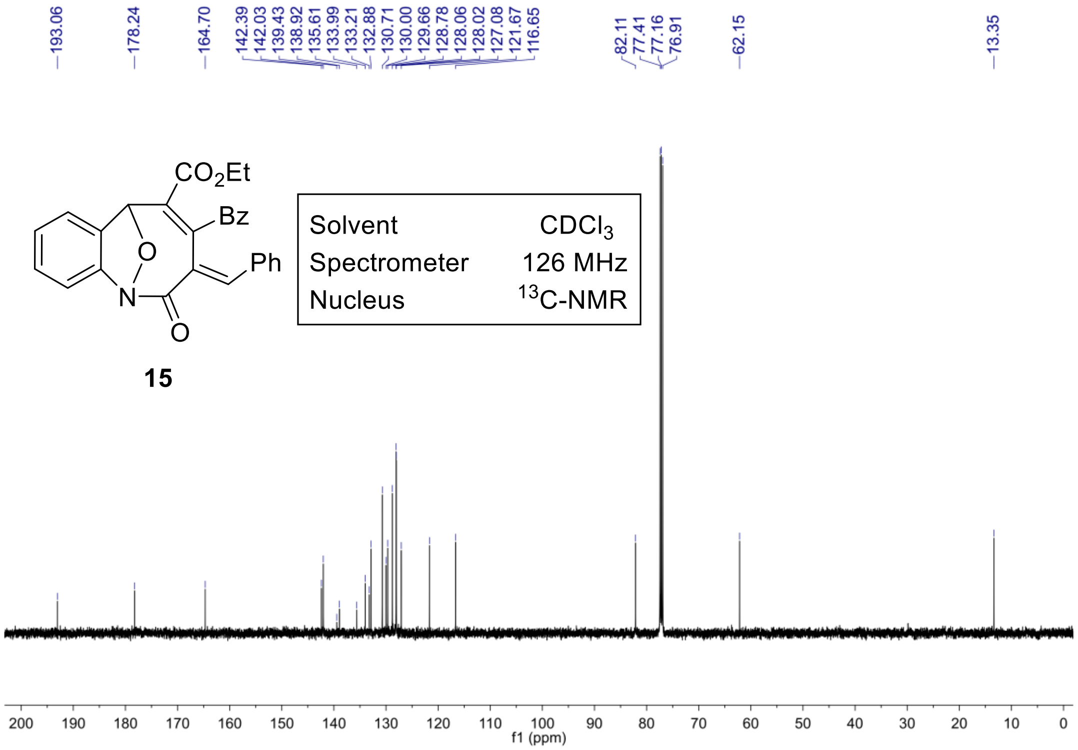
CO2Et
Bz
Ph
O
N
O
15
Solvent CDCl3
Spectrometer 126 MHz
Nucleus 13C-NMR
f1 (ppm)

HPLC Analyses Figures of Compounds 3, 4, and 8-11

Condition: Daicel Chiralpak IC, λ = 254 nm, hexane/2-propanol = 90:10

flow rate = 1.0 mL/min

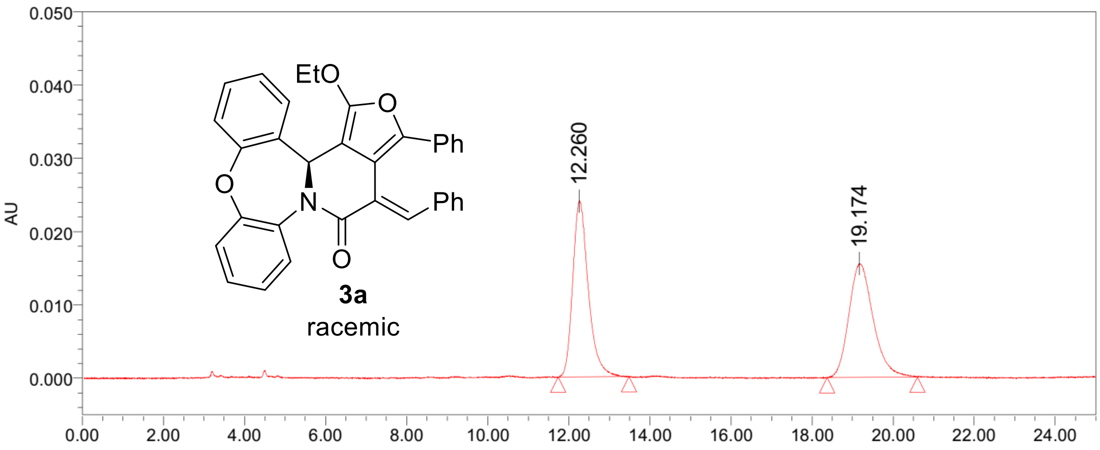

Entry	RT min	Height mV	Area mV.sec	% Area %
1	12.260	648403	23985	49.99
2	19.174	648687	15513	50.01

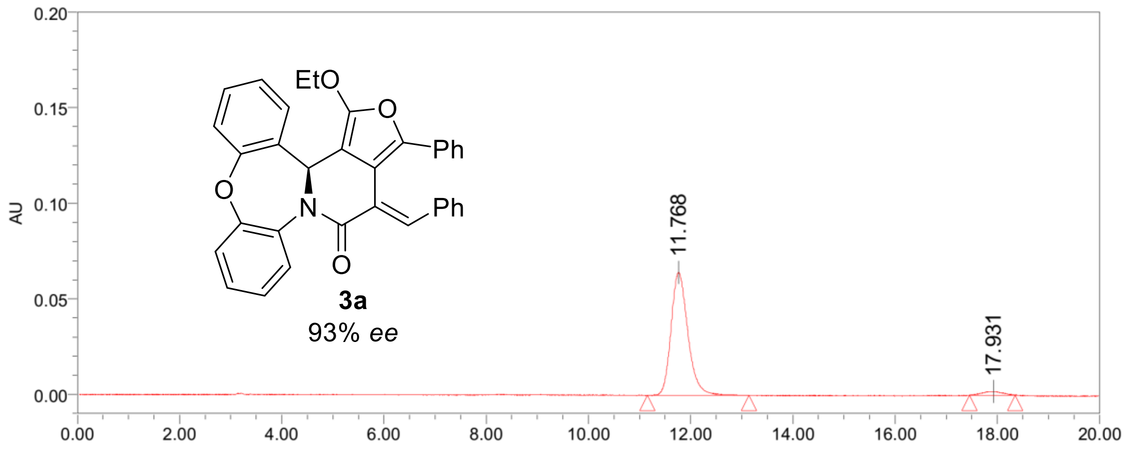

Entry	RT min	Height mV	Area mV.sec	% Area %
1	11.768	1479995	64378	96.27
2	17.931	57289	1991	3.73

Condition：Daicel Chiralpak IC, λ = 254 nm, hexane/2-propanol = 90:10

flow rate = 1.0 mL/min

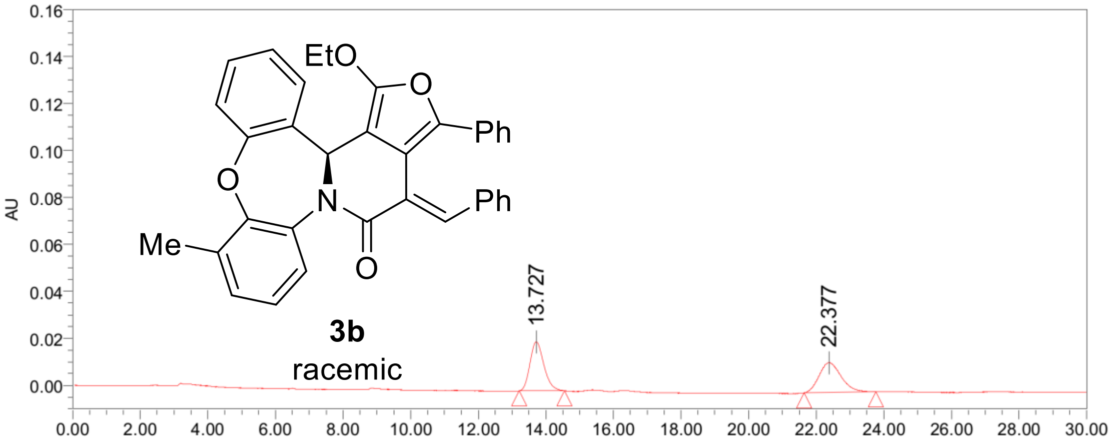

Entry	RT<br>min	Height<br>mV	Area<br>mV.sec	% Area<br>%
1	13.727	597058	20945	50.78
2	22.377	578710	12688	49.22

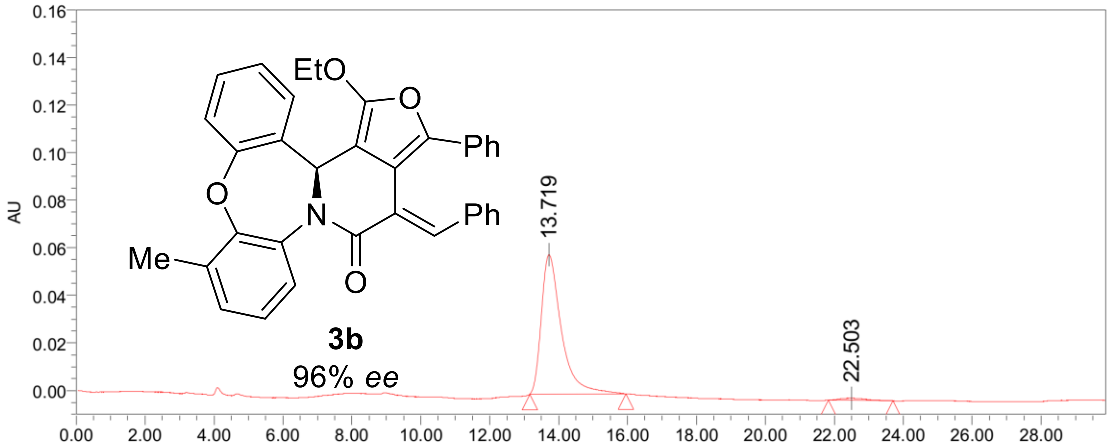

Entry	RT<br>min	Height<br>mV	Area<br>mV.sec	% Area<br>%
1	13.719	2361257	58608	97.84
2	22.503	52211	992	2.16

Condition: Daicel Chiralpak IC, λ = 254 nm, hexane/2-propanol = 90:10

flow rate = 1.0 mL/min

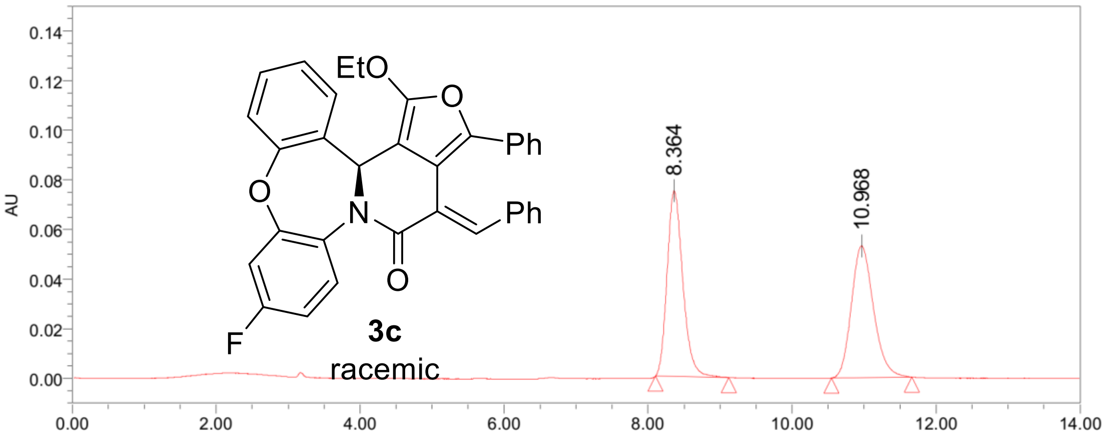

Entry	RT min	Height mV	Area mV.sec	% Area %
1	8.364	1135345	74812	50.00
2	10.968	1135518	53151	50.00

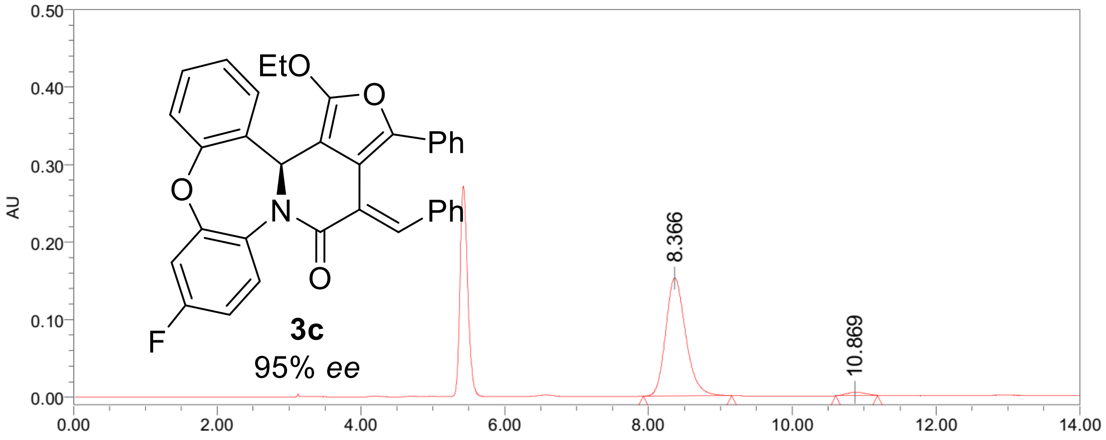

Entry	RT min	Height mV	Area mV.sec	% Area %
1	8.366	2951147	152297	97.47
2	10.869	76522	4092	2.53

Condition: Daicel Chiralpak IC, λ = 254 nm, hexane/2-propanol = 90:10

flow rate = 1.0 mL/min

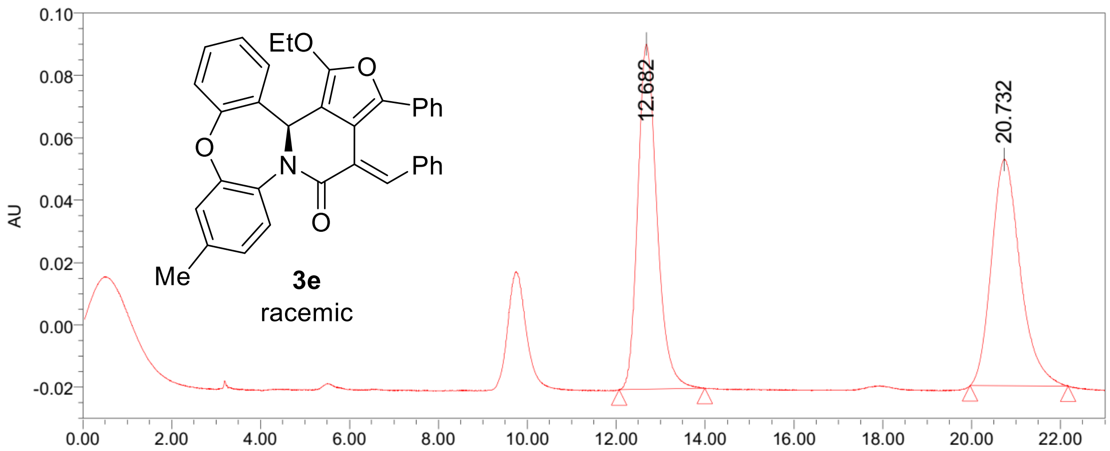

Entry	RT<br>min	Height<br>mV	Area<br>mV.sec	% Area<br>%
1	12.682	3292371	110634	50.12
2	20.732	3276205	72592	49.88

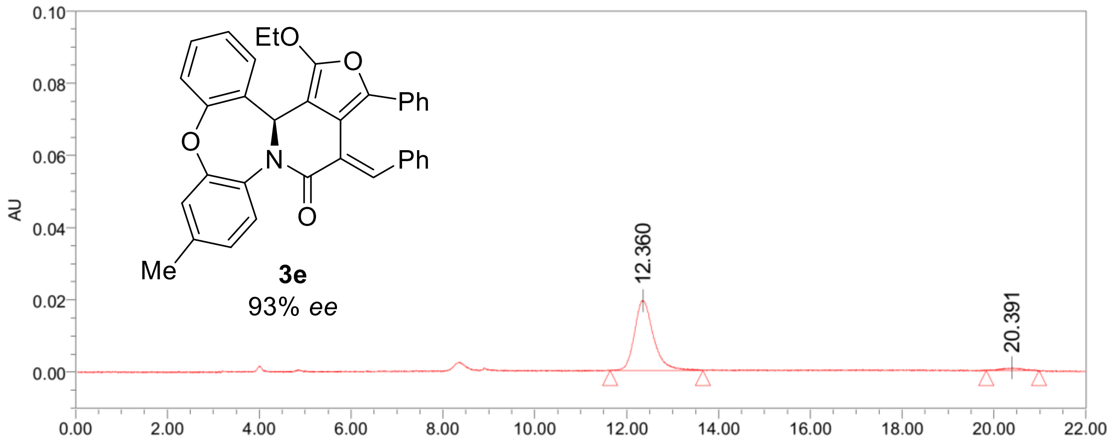

Entry	RT<br>min	Height<br>mV	Area<br>mV.sec	% Area<br>%
1	12.360	558796	19403	96.52
2	20.391	20128	748	3.48

Condition：Daicel Chiralpak IC, λ = 254 nm, hexane/2-propanol = 90:10

flow rate = 1.0 mL/min

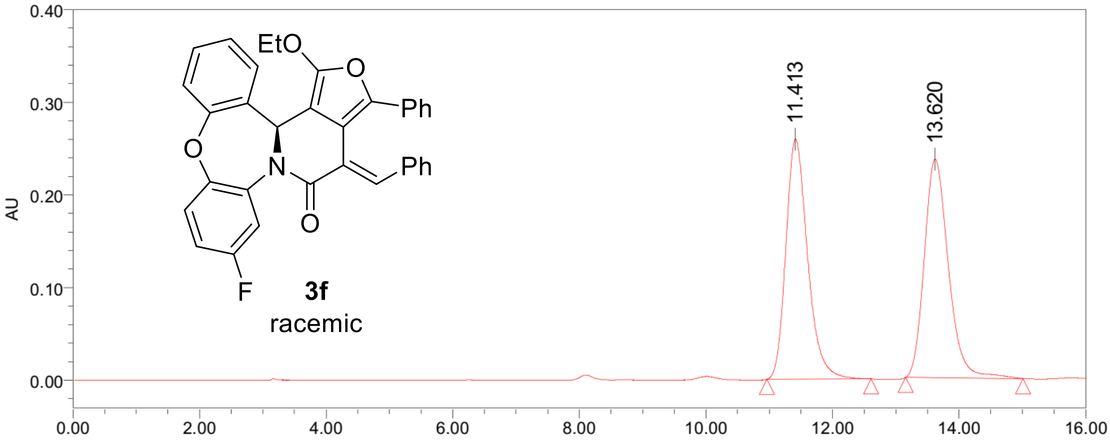

Entry	RT<br>min	Height<br>mV	Area<br>mV.sec	% Area<br>%
1	11.413	6183908	259100	49.85
2	13.620	6221258	235868	50.15

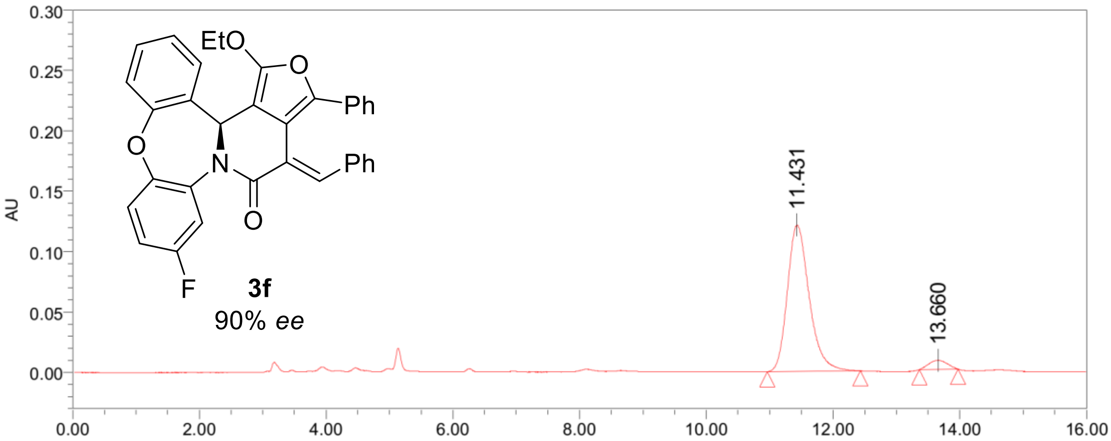

Entry	RT<br>min	Height<br>mV	Area<br>mV.sec	% Area<br>%
1	11.431	2904457	121170	95.07
2	13.660	150587	7474	4.93

Condition: Daicel Chiralpak IC, λ = 254 nm, hexane/2-propanol = 90:10

flow rate = 1.0 mL/min

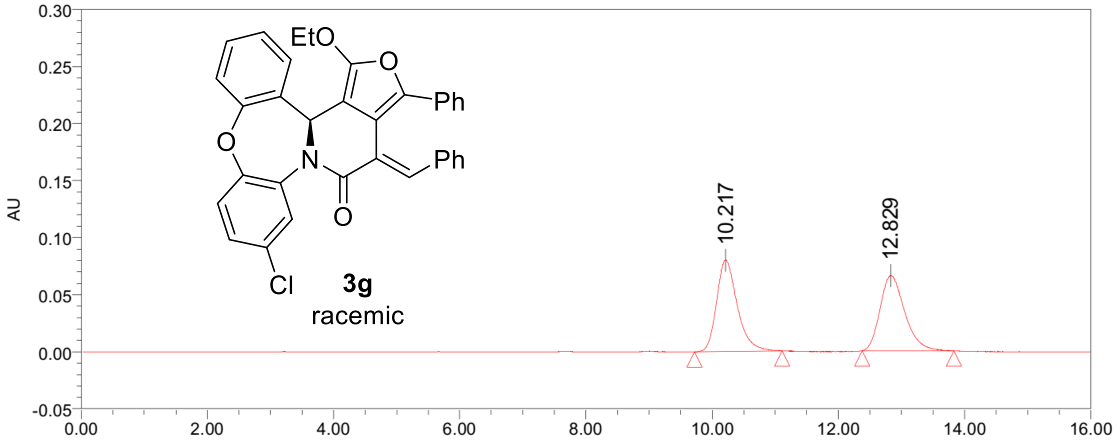

Entry	RT<br>min	Height<br>mV	Area<br>mV.sec	% Area<br>%
1	10.217	1836621	80072	50.45
2	12.829	1803558	66009	49.55

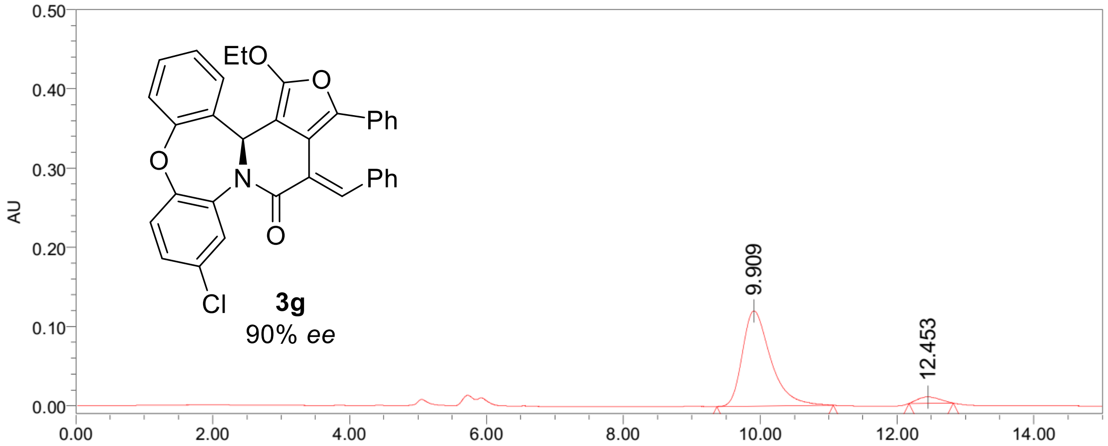

Entry	RT<br>min	Height<br>mV	Area<br>mV.sec	% Area<br>%
1	9.909	3438322	120178	95.02
2	12.453	180191	8085	4.98

Condition: Daicel Chiralpak IC, λ = 254 nm, hexane/2-propanol = 90:10

flow rate = 1.0 mL/min

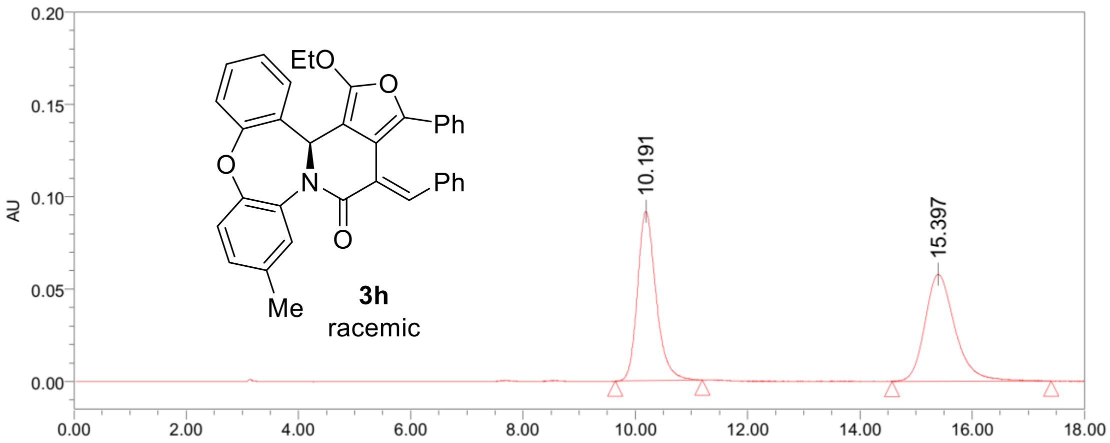

Entry	RT<br>min	Height<br>mV	Area<br>mV.sec	% Area<br>%
1	10.191	2114177	91730	49.66
2	15.397	2143216	57826	50.34

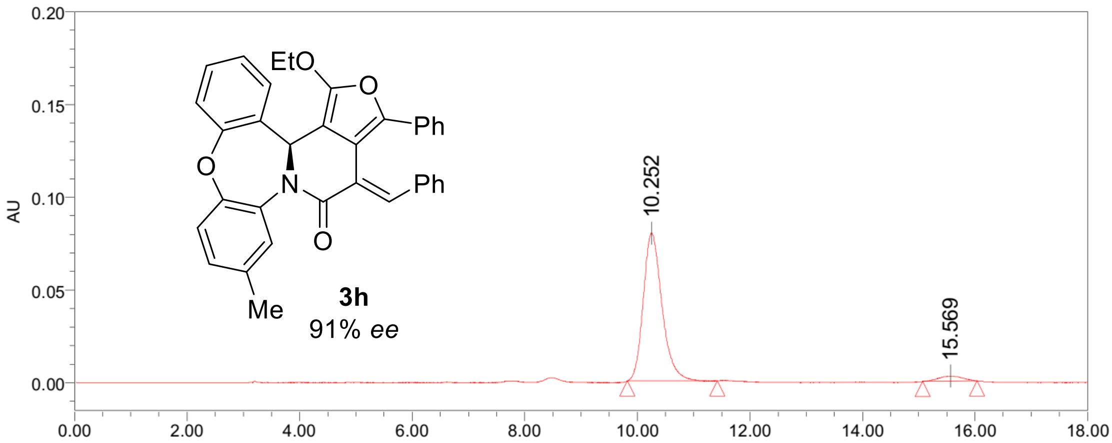

Entry	RT<br>min	Height<br>mV	Area<br>mV.sec	% Area<br>%
1	10.252	1849267	79607	95.67
2	15.569	83642	2791	4.33

Condition: Daicel Chiralpak IC, λ = 254 nm, hexane/2-propanol = 90:10

flow rate = 1.0 mL/min

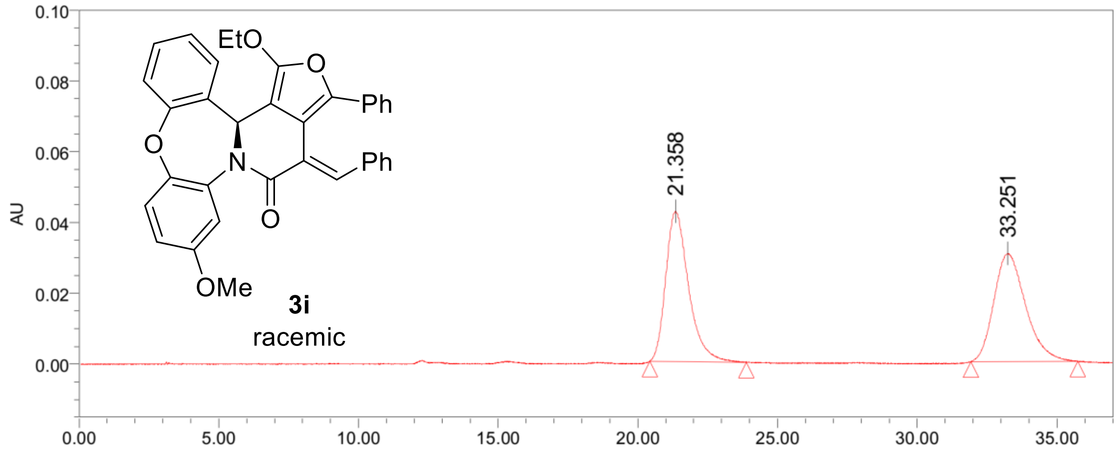

Entry	RT<br>min	Height<br>mV	Area<br>mV.sec	% Area<br>%
1	21.358	2417378	42369	50.00
2	33.251	2417164	30510	50.00

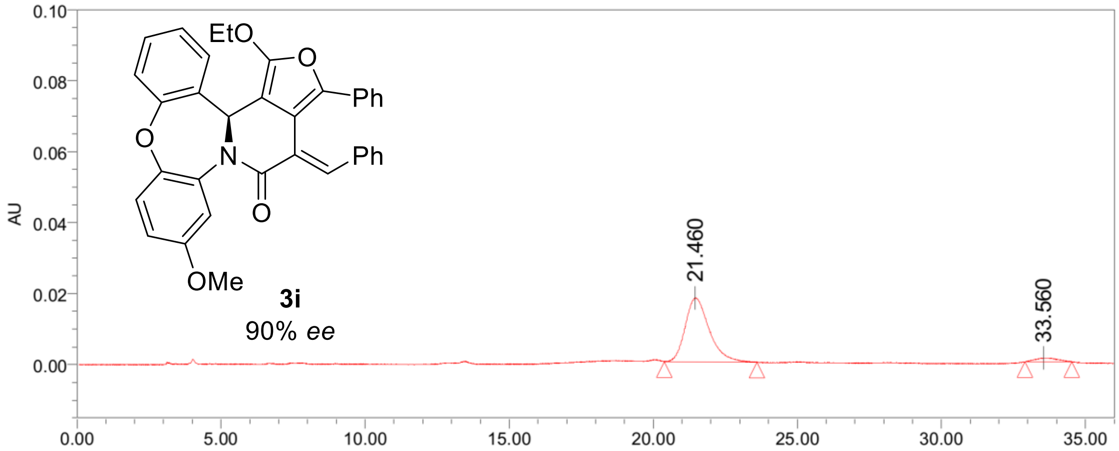

Entry	RT<br>min	Height<br>mV	Area<br>mV.sec	% Area<br>%
1	21.460	1044566	18033	95.01
2	33.560	54853	1070	4.99

Condition: Daicel Chiralpak IC, λ = 254 nm, hexane/2-propanol = 80:20

flow rate = 1.0 mL/min

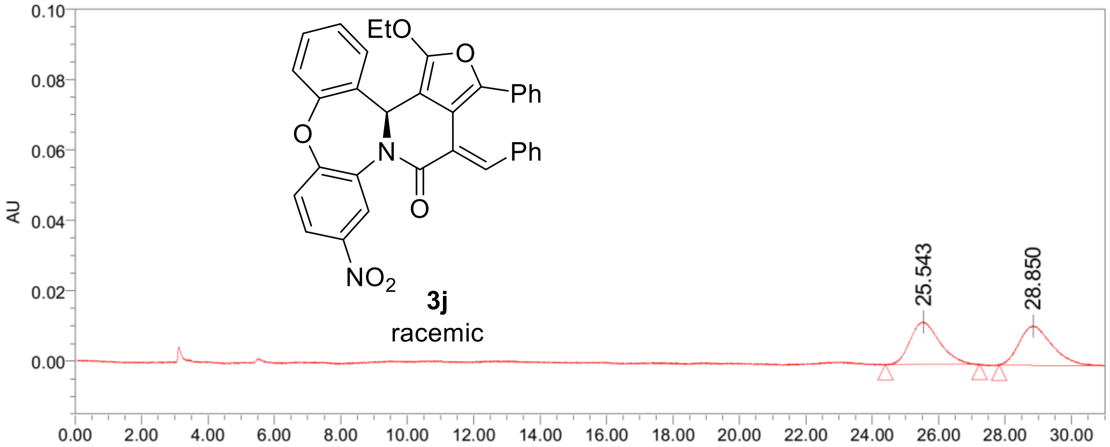

Entry	RT<br>min	Height<br>mV	Area<br>mV.sec	% Area<br>%
1	25.543	748184	12105	49.36
2	28.850	767736	11228	50.64

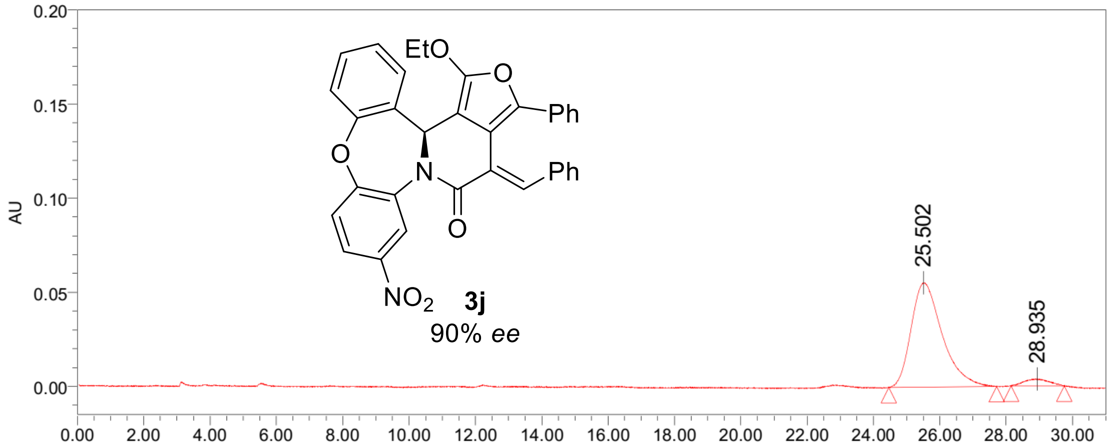

Entry	RT<br>min	Height<br>mV	Area<br>mV.sec	% Area<br>%
1	25.502	3592847	55368	95.06
2	28.935	186624	3654	4.94

Condition: Daicel Chiralpak IC, λ = 254 nm, hexane/2-propanol = 90:10

flow rate = 1.0 mL/min

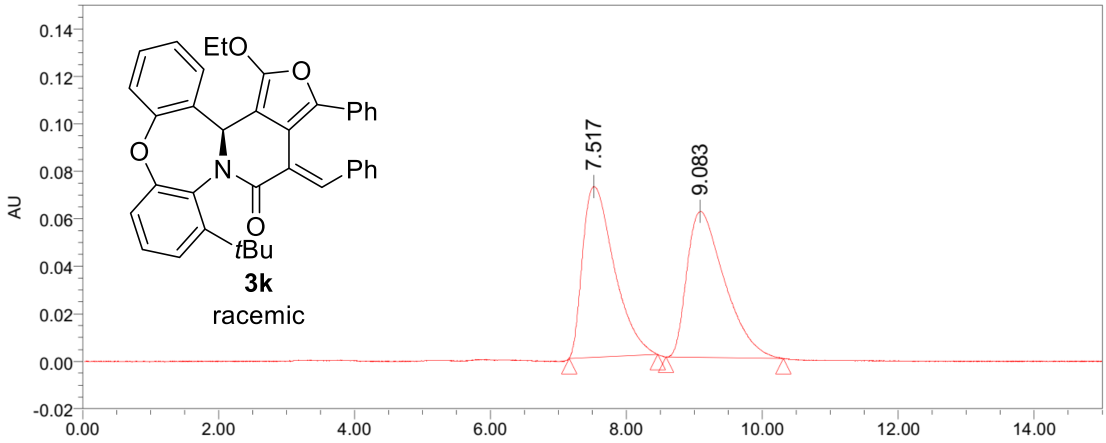

Entry	RT<br>min	Height<br>mV	Area<br>mV.sec	% Area<br>%
1	7.517	2329296	71949	49.53
2	9.083	2373674	61578	50.47

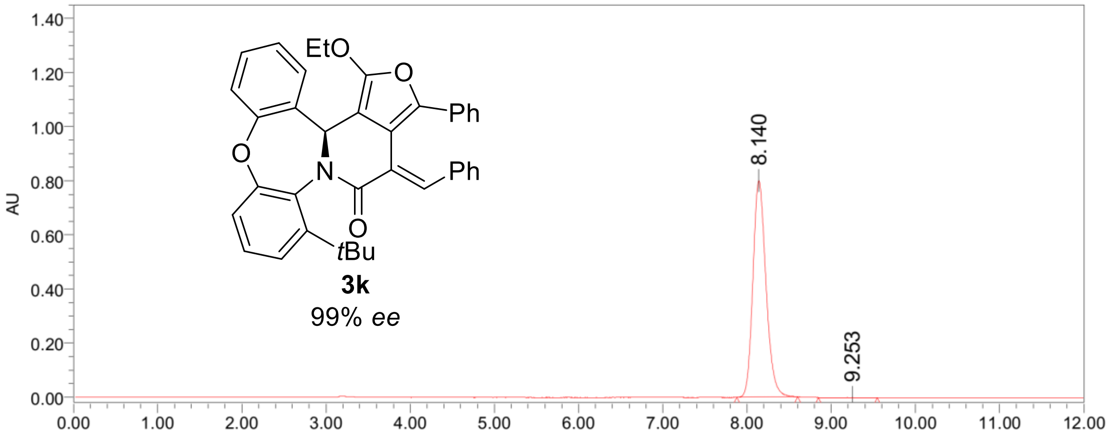

Entry	RT<br>min	Height<br>mV	Area<br>mV.sec	% Area<br>%
1	8.140	8613877	800077	99.96
2	9.253	3683	342	0.04

Condition：Daicel Chiralpak IC, λ = 254 nm, hexane/2-propanol = 90:10

flow rate = 1.0 mL/min

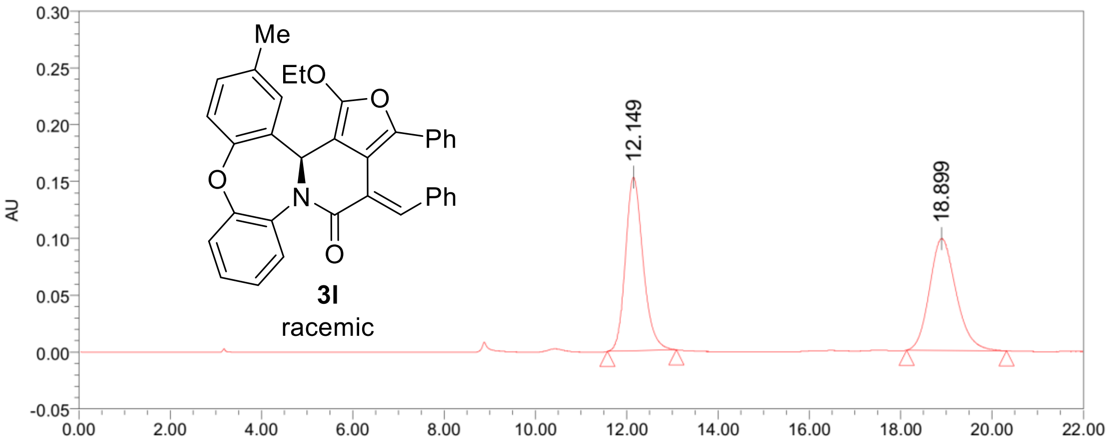

Entry	RT<br>min	Height<br>mV	Area<br>mV.sec	% Area<br>%
1	12.149	4070848	152667	50.41
2	18.899	4004064	98322	49.59

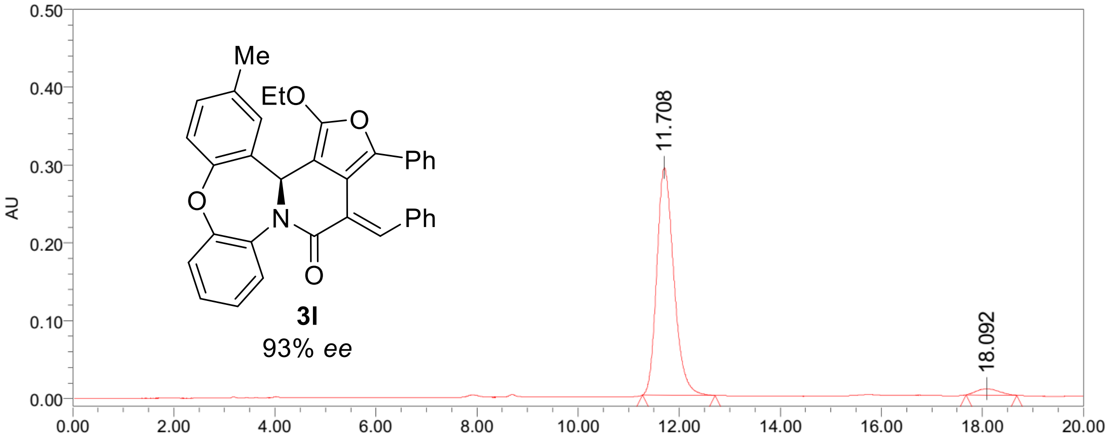

Entry	RT<br>min	Height<br>mV	Area<br>mV.sec	% Area<br>%
1	11.708	6873575	292048	96.55
2	18.092	245816	8004	3.45

Condition：Daicel Chiralpak IC, λ = 254 nm, hexane/2-propanol = 90:10

flow rate = 1.0 mL/min

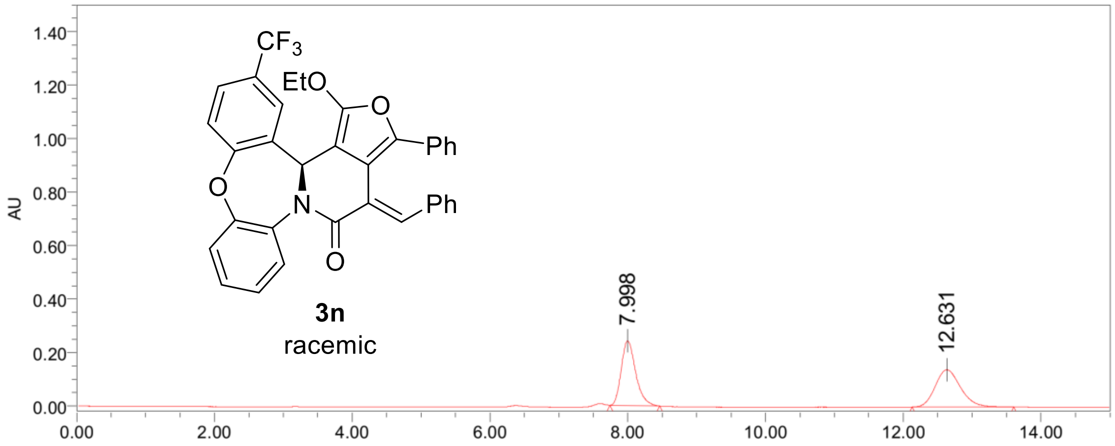

Entry	RT<br>min	Height<br>mV	Area<br>mV.sec	% Area<br>%
1	7.998	3528471	242351	49.67
2	12.631	3575101	139879	50.33

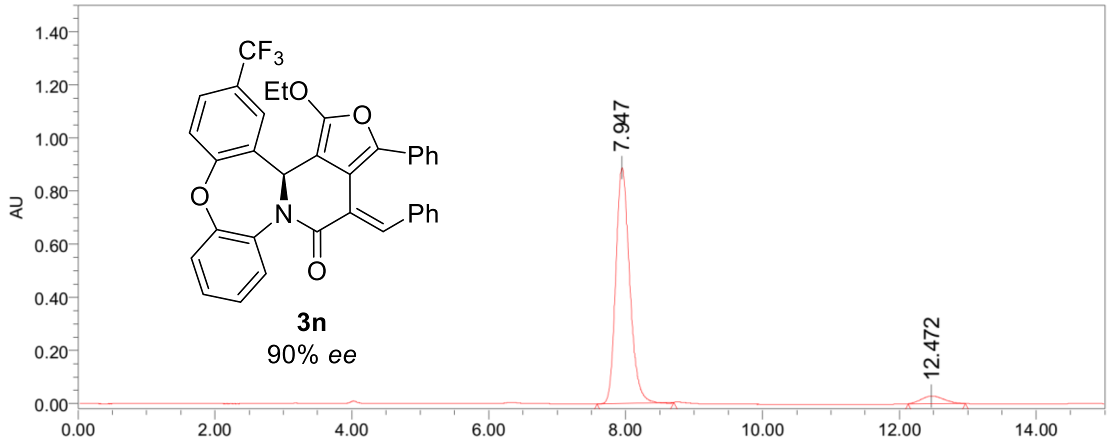

Entry	RT<br>min	Height<br>mV	Area<br>mV.sec	% Area<br>%
1	7.947	12497165	887654	95.01
2	12.472	656335	28586	4.99

Condition：Daicel Chiralpak IC, λ = 254 nm, hexane/2-propanol = 95:5

flow rate = 1.0 mL/min

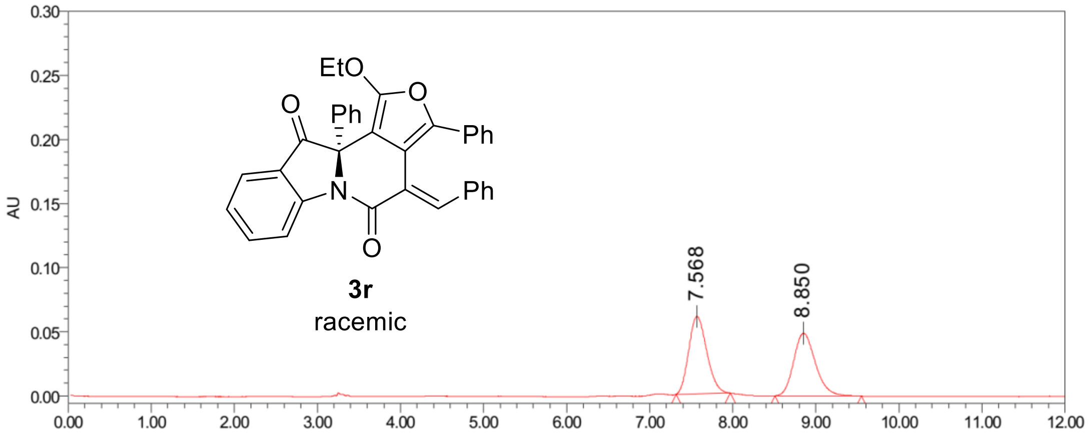

Entry	RT min	Height mV	Area mV.sec	% Area %
1	7.568	60436	908081	50.69
2	8.850	48926	883207	49.31

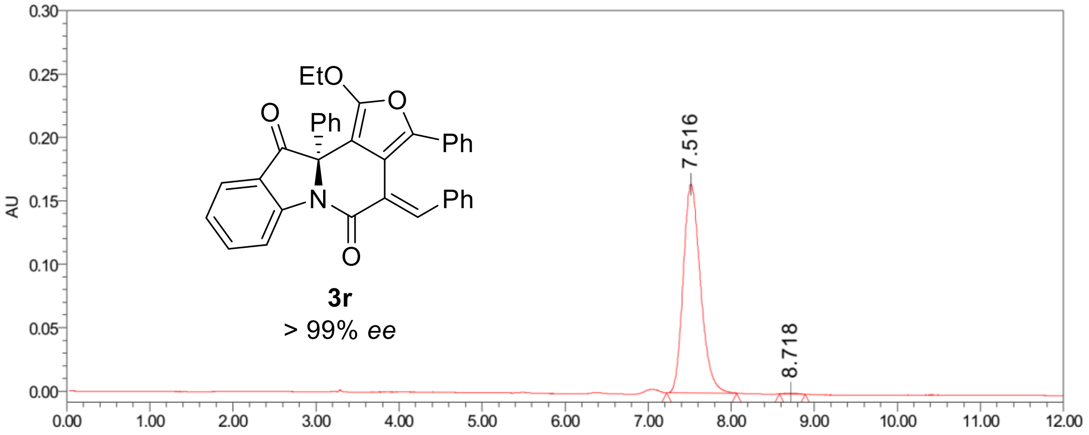

Entry	RT min	Height mV	Area mV.sec	% Area %
1	7.516	164736	2371204	99.71
2	8.718	645	6817	0.29

Condition：Daicel Chiralpak IC, λ = 254 nm, hexane/2-propanol = 95:5

flow rate = 1.0 mL/min

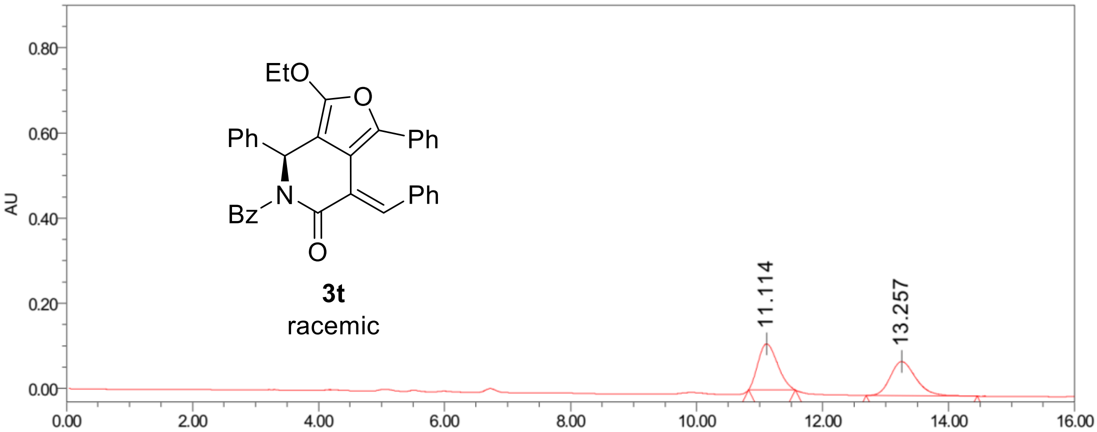

Entry	RT min	Height mV	Area mV.sec	% Area %
1	11.114	108268	2328510	50.87
2	13.257	79867	2249122	49.13

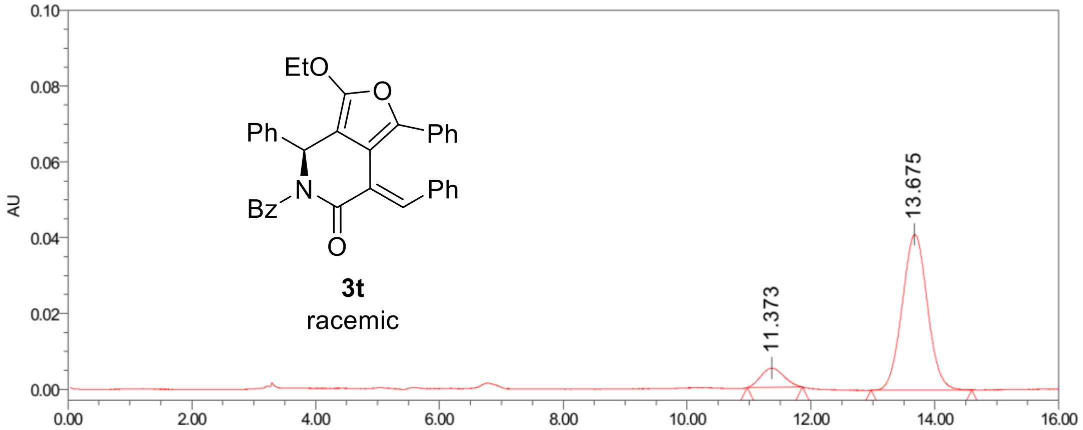

Entry	RT min	Height mV	Area mV.sec	% Area %
1	11.373	5064	130423	10.15
2	13.675	41059	1155008	89.95

Condition： Daicel Chiralpak IC, λ = 254 nm, hexane/2-propanol = 90:10

flow rate = 1.0 mL/min

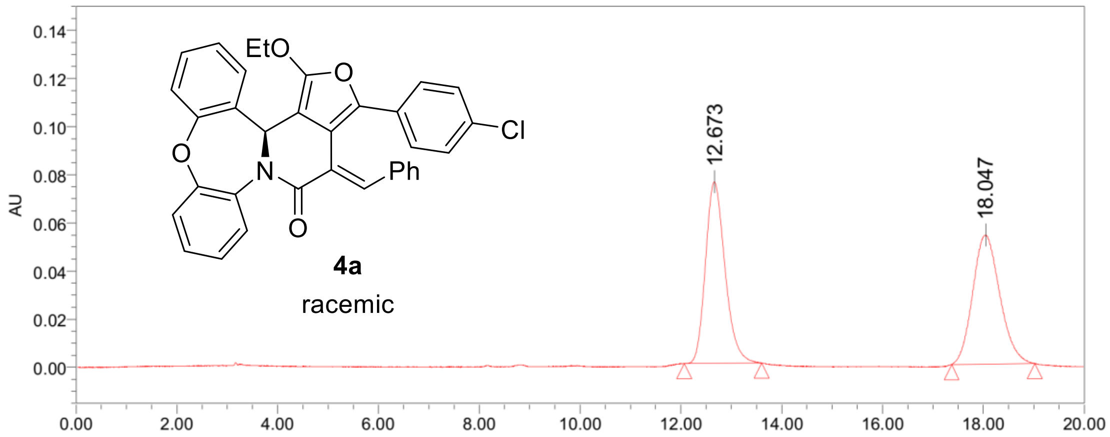

Entry	RT<br>min	Height<br>mV	Area<br>mV.sec	% Area<br>%
1	12.673	2026397	75422	50.16
2	18.047	2013535	53644	49.84

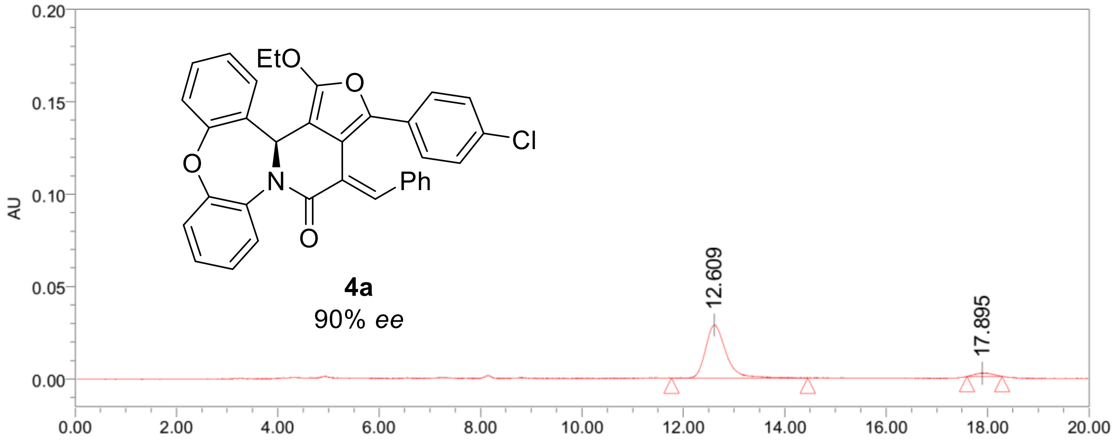

Entry	RT<br>min	Height<br>mV	Area<br>mV.sec	% Area<br>%
1	12.609	796689	28697	94.82
2	17.895	43491	1786	5.18

Condition：Daicel Chiralpak IC, λ = 254 nm, hexane/2-propanol = 90:10

flow rate = 1.0 mL/min

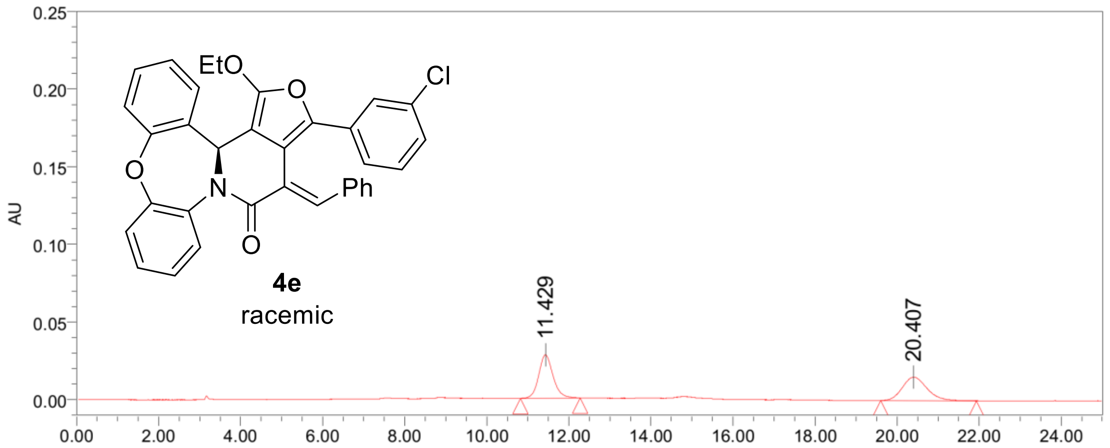

Entry	RT<br>min	Height<br>mV	Area<br>mV.sec	% Area<br>%
1	11.429	672583	27881	51.61
2	20.407	630724	15159	48.39

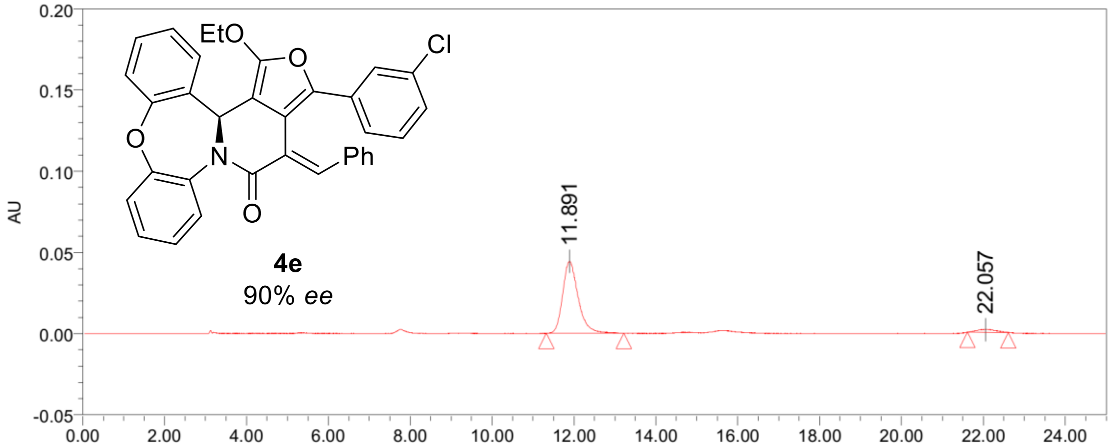

Entry	RT<br>min	Height<br>mV	Area<br>mV.sec	% Area<br>%
1	11.891	1171564	44222	95.00
2	22.057	61640	1837	5.00

Condition：Daicel Chiralpak IC, λ = 254 nm, hexane/2-propanol = 90:10

flow rate = 1.0 mL/min

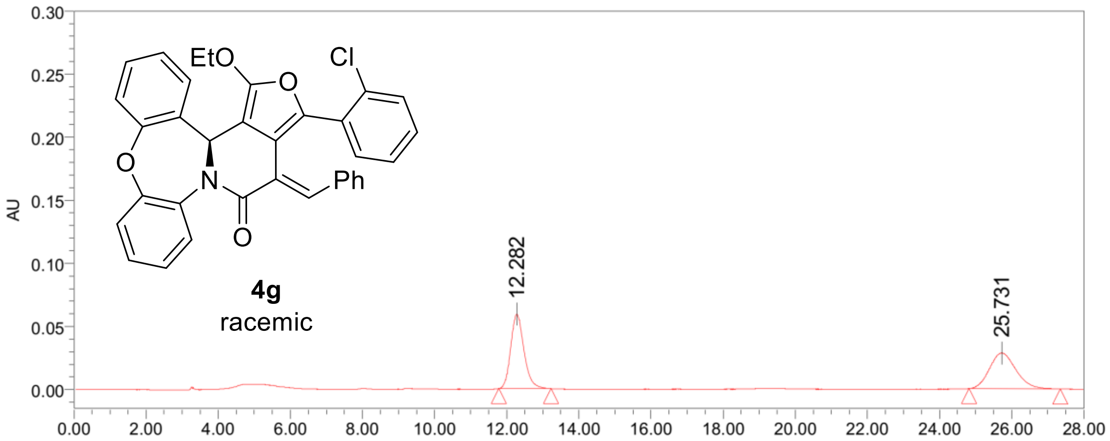

Entry	RT<br>min	Height<br>mV	Area<br>mV.sec	% Area<br>%
1	12.282	1504896	58945	50.91
2	25.731	1450853	28377	49.09

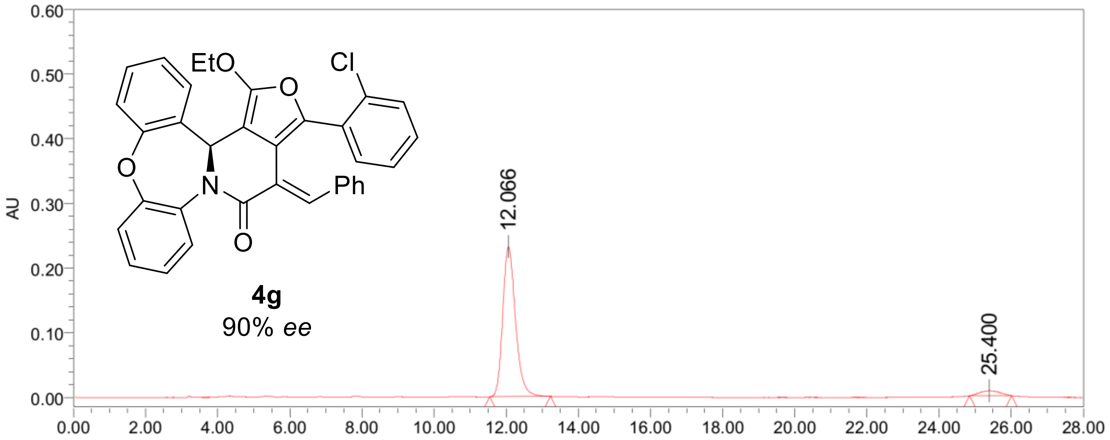

Entry	RT<br>min	Height<br>mV	Area<br>mV.sec	% Area<br>%
1	12.066	5708977	232318	95.11
2	25.400	293834	7522	4.89

Condition：Daicel Chiralpak IC, λ = 254 nm, hexane/2-propanol = 90:10

flow rate = 1.0 mL/min

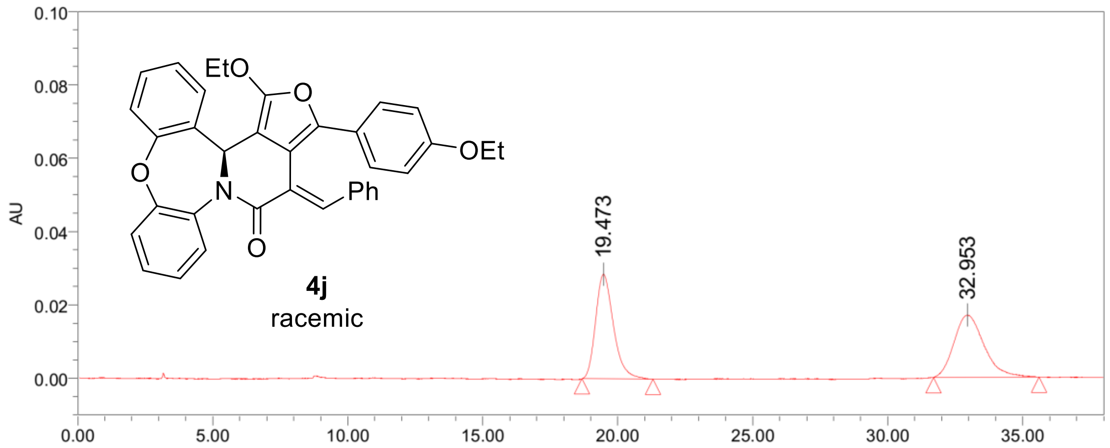

Entry	RT<br>min	Height<br>mV	Area<br>mV.sec	% Area<br>%
1	19.473	1309496	28540	49.97
2	32.953	1311163	16969	50.03

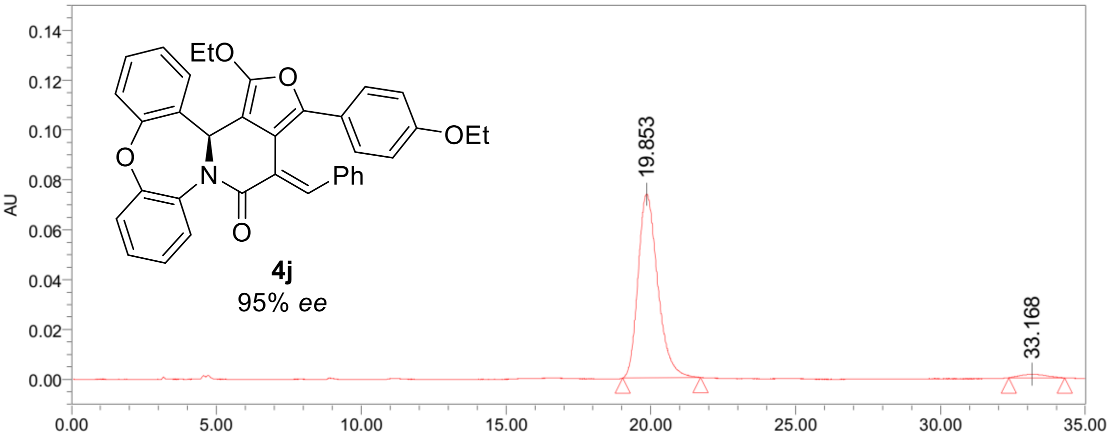

Entry	RT<br>min	Height<br>mV	Area<br>mV.sec	% Area<br>%
1	19.853	3487963	73671	97.59
2	33.168	86290	1415	2.41

Condition：Daicel Chiralpak IC, λ = 254 nm, hexane/2-propanol = 90:10

flow rate = 1.0 mL/min

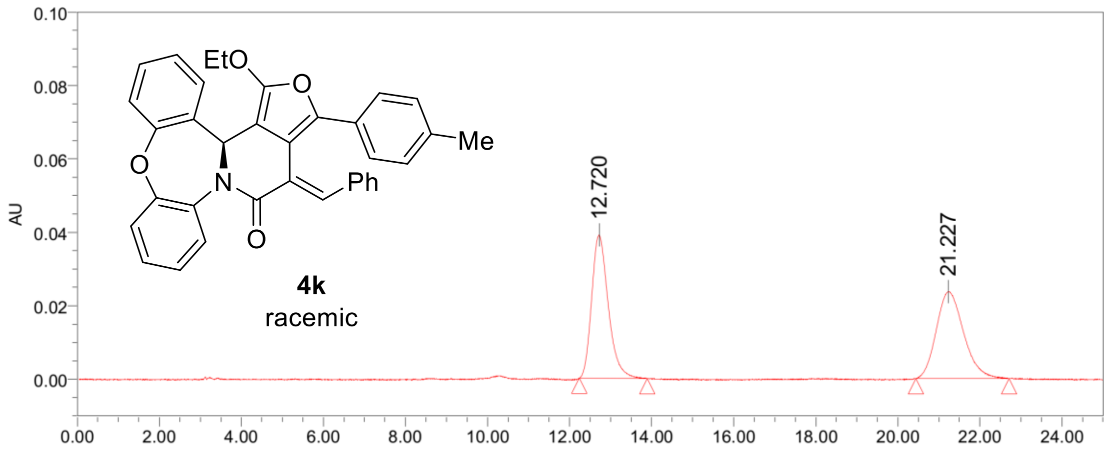

Entry	RT<br>min	Height<br>mV	Area<br>mV.sec	% Area<br>%
1	12.720	1093966	38996	50.17
2	21.227	1086568	23621	49.83

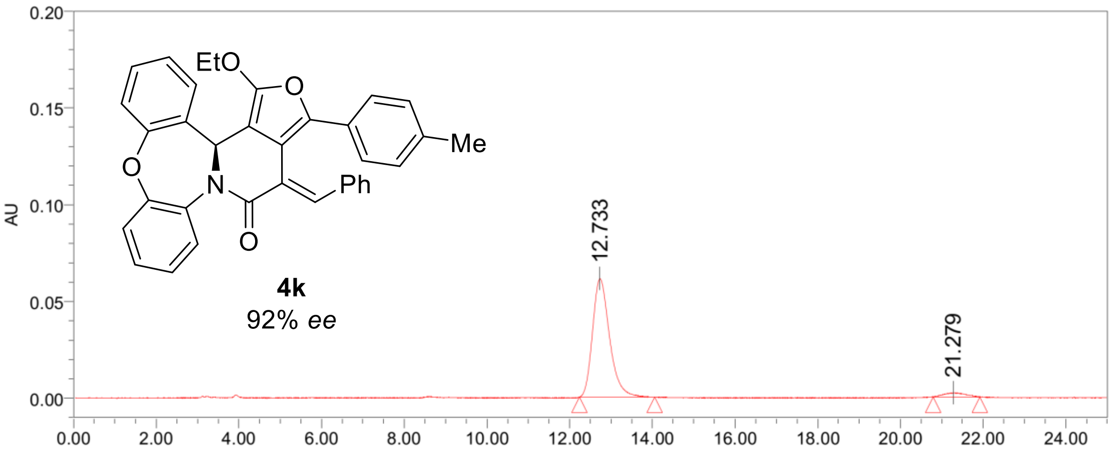

Entry	RT<br>min	Height<br>mV	Area<br>mV.sec	% Area<br>%
1	12.733	1746114	61286	96.05
2	21.279	71894	2043	3.95

Condition: Daicel Chiralpak IC, λ = 254 nm, hexane/2-propanol = 90:10

flow rate = 1.0 mL/min

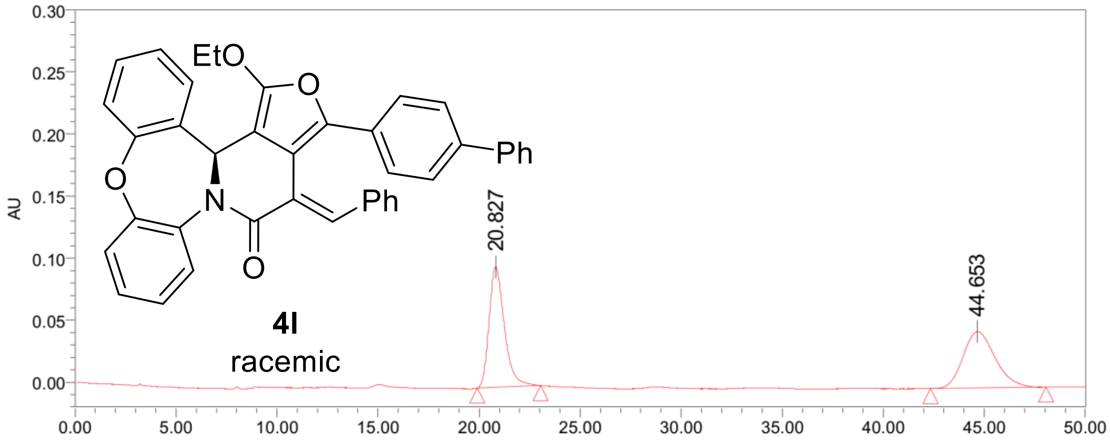

Entry	RT<br>min	Height<br>mV	Area<br>mV.sec	% Area<br>%
1	20.827	5141941	97075	50.51
2	44.653	5037317	45419	49.49

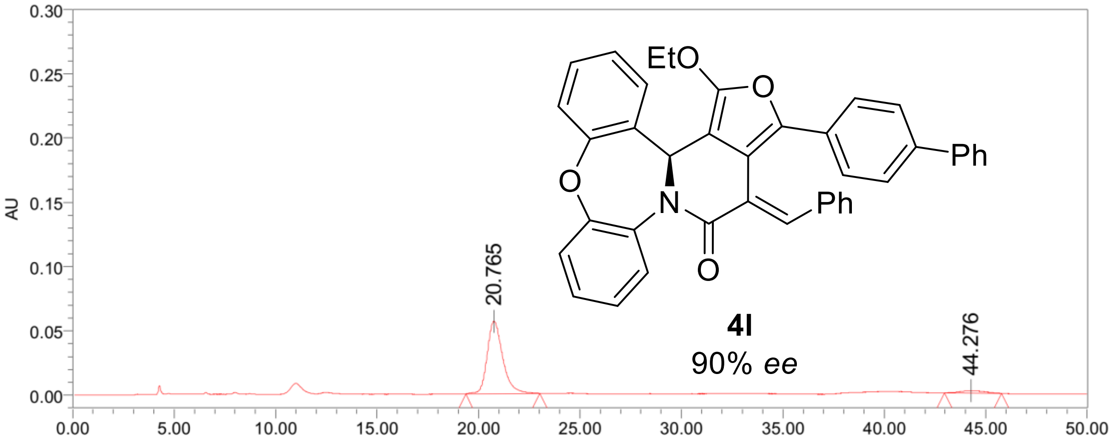

Entry	RT<br>min	Height<br>mV	Area<br>mV.sec	% Area<br>%
1	20.765	2994879	56318	95.02
2	44.276	157013	1771	4.98

Condition：Daicel Chiralpak IC, λ = 254 nm, hexane/2-propanol = 85:15

flow rate = 1.0 mL/min

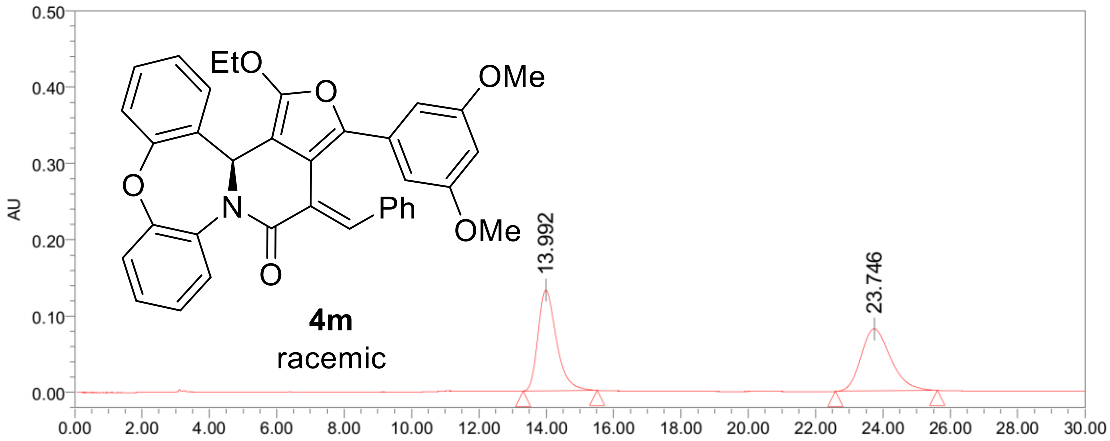

Entry	RT min	Height mV	Area mV.sec	% Area %
1	13.992	5031551	132139	50.32
2	23.746	4967807	81373	49.68

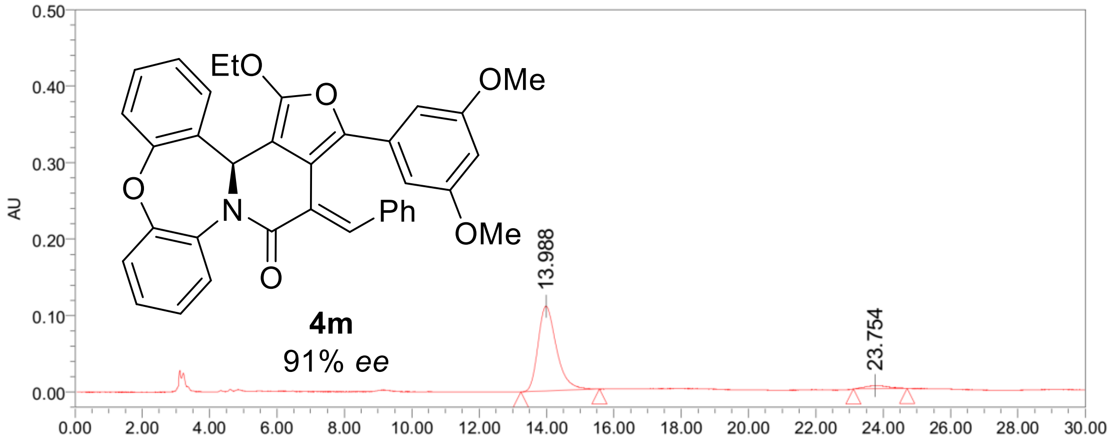

Entry	RT min	Height mV	Area mV.sec	% Area %
1	13.988	4228489	111042	95.61
2	23.754	194161	4028	4.39

Condition：Daicel Chiralpak IC, λ = 254 nm, hexane/2-propanol = 90:10

flow rate = 1.0 mL/min

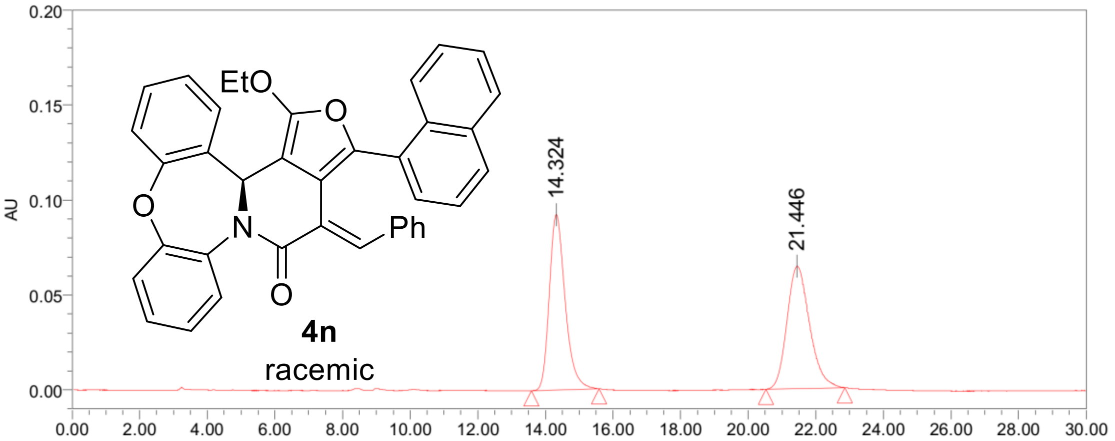

Entry	RT min	Height mV	Area mV.sec	% Area %
1	14.324	2998082	92426	49.48
2	21.446	2997939	64407	50.52

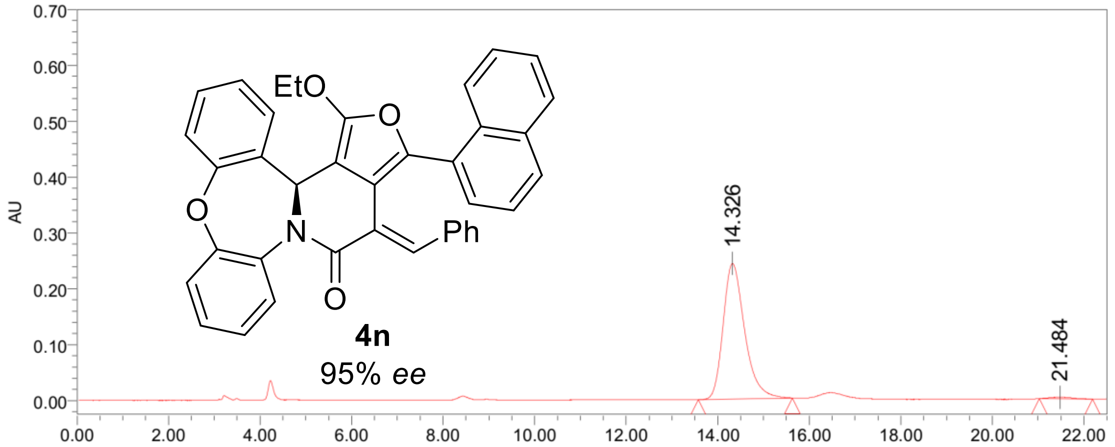

Entry	RT min	Height mV	Area mV.sec	% Area %
1	14.326	7889330	242801	97.53
2	21.484	104297	2912	2.47

Condition: Daicel Chiralpak IC, λ = 254 nm, hexane/2-propanol = 90:10

flow rate = 1.0 mL/min

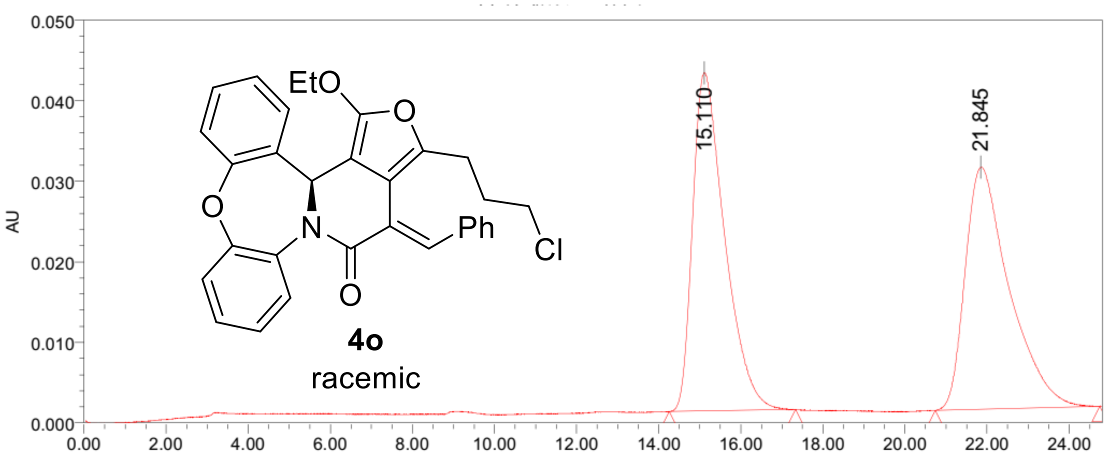

Entry	RT<br>min	Height<br>mV	Area<br>mV.sec	% Area<br>%
1	15.110	2281437	41966	50.47
2	21.845	2238651	30028	49.53

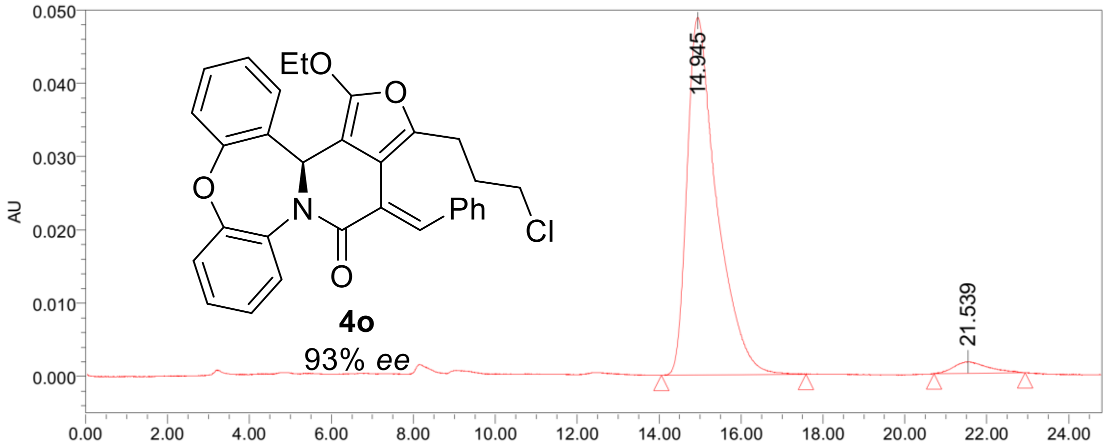

Entry	RT<br>min	Height<br>mV	Area<br>mV.sec	% Area<br>%
1	14.945	2448375	48760	96.45
2	21.539	90202	1568	3.55

Condition：Daicel Chiralpak IC, λ = 254 nm, hexane/2-propanol = 90:10

flow rate = 1.0 mL/min

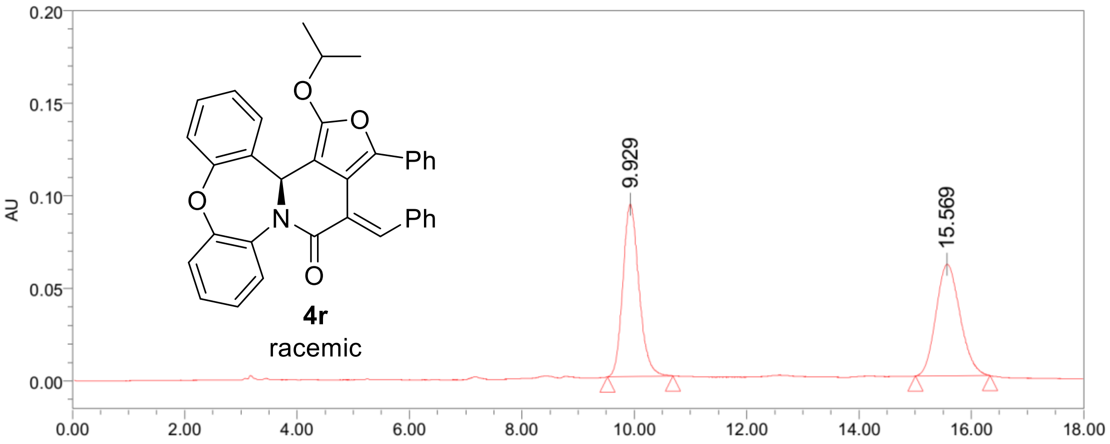

Entry	RT<br>min	Height<br>mV	Area<br>mV.sec	% Area<br>%
1	9.929	1861311	92999	50.84
2	15.569	1799573	60188	49.16

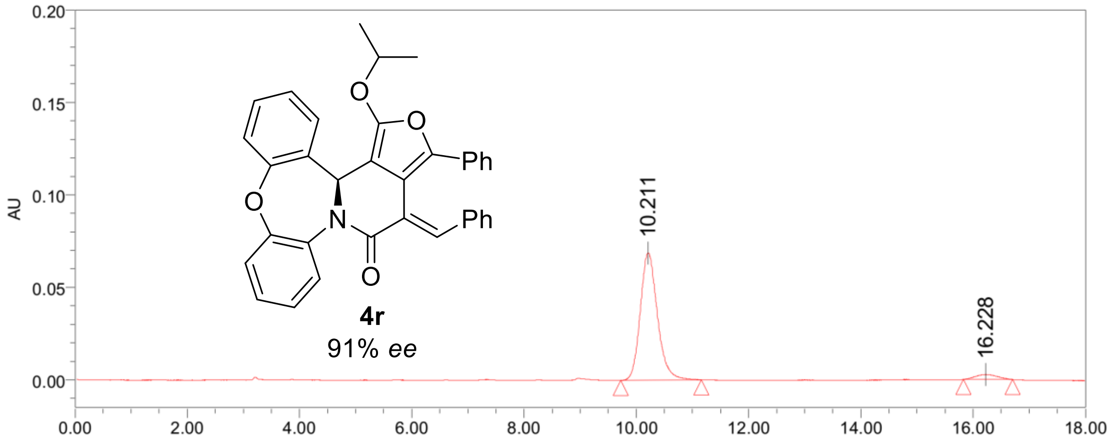

Entry	RT<br>min	Height<br>mV	Area<br>mV.sec	% Area<br>%
1	10.211	1463814	68812	95.33
2	16.228	71767	2665	4.67

Condition： Daicel Chiralpak IC, λ = 254 nm, hexane/2-propanol = 85:15

flow rate = 1.0 mL/min

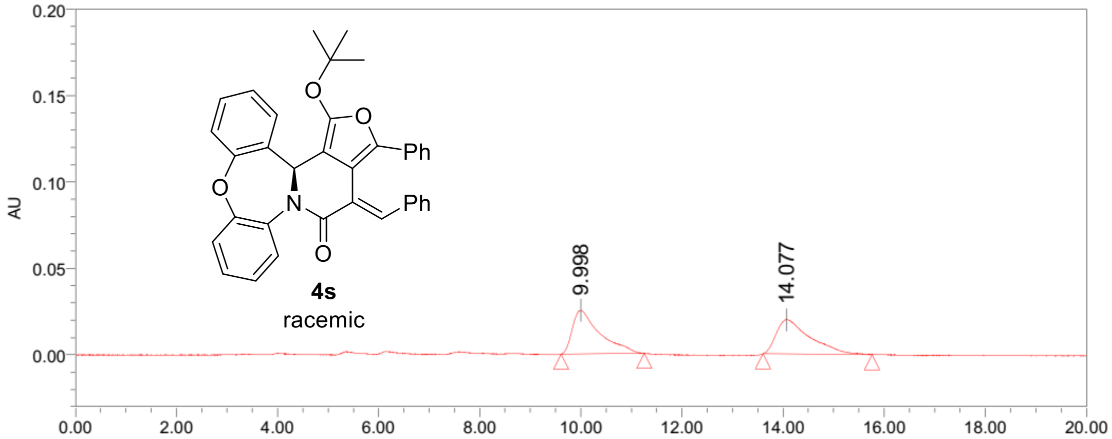

Entry	RT<br>min	Height<br>mV	Area<br>mV.sec	% Area<br>%
1	9.998	950869	25144	50.79
2	14.077	921302	19829	49.21

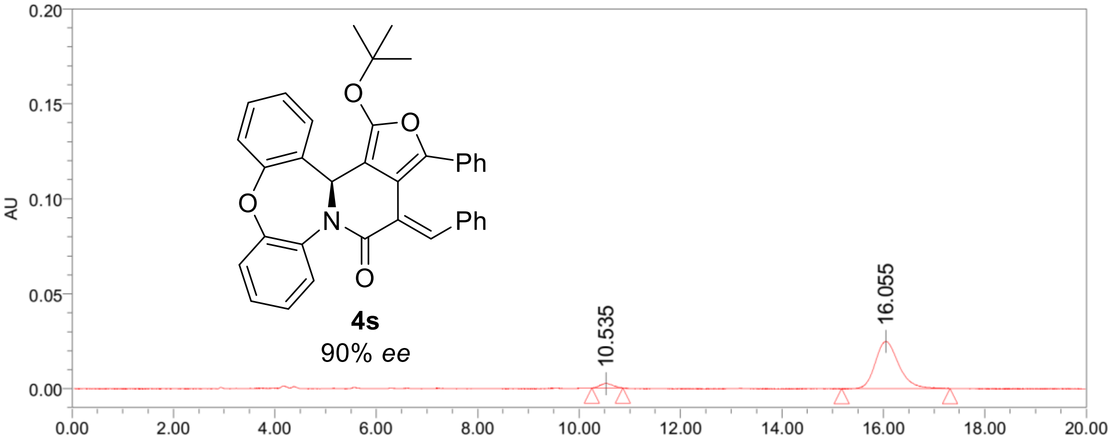

Entry	RT<br>min	Height<br>mV	Area<br>mV.sec	% Area<br>%
1	10.535	40761	2374	4.86
2	16.055	797235	24968	95.14

Condition: Daicel Chiralpak IC, λ = 254 nm, hexane/2-propanol = 90:10

flow rate = 1.0 mL/min

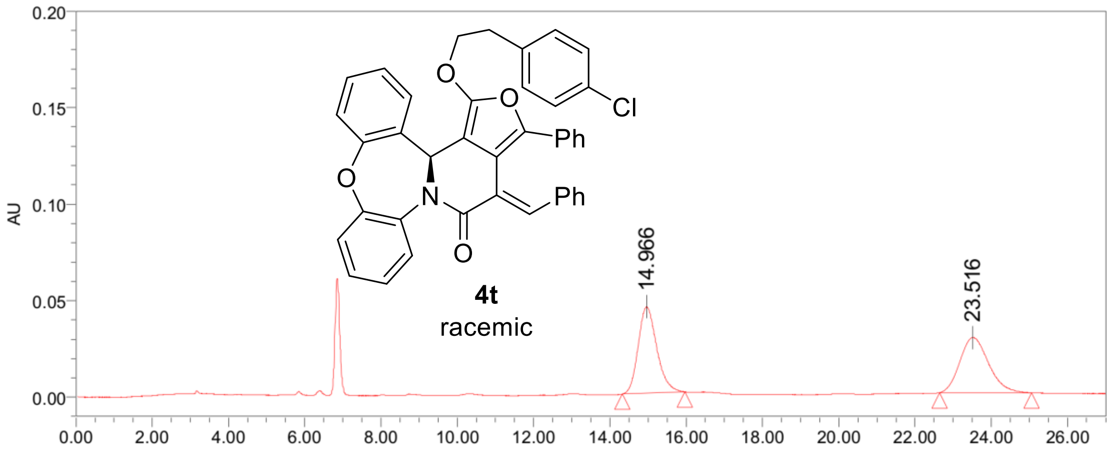

Entry	RT<br>min	Height<br>mV	Area<br>mV.sec	% Area<br>%
1	14.966	1520906	44740	50.48
2	23.516	1491785	28570	49.52

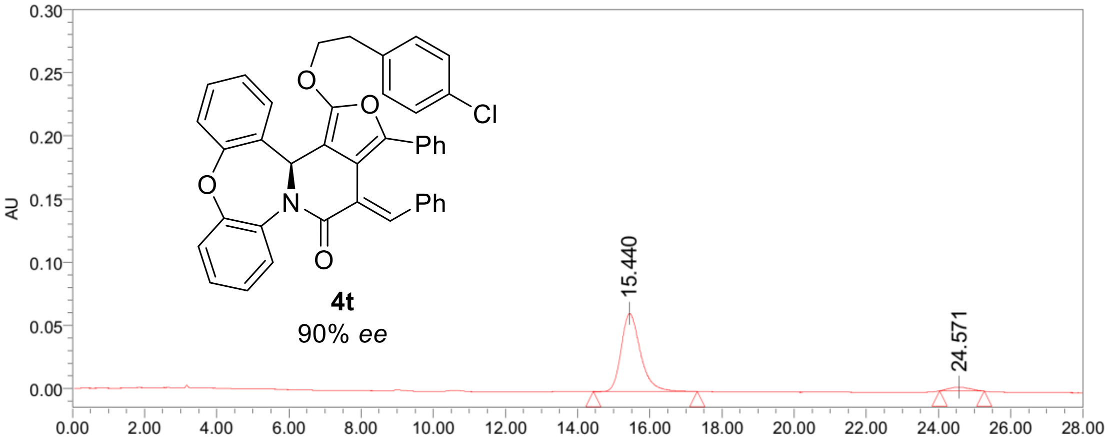

Entry	RT<br>min	Height<br>mV	Area<br>mV.sec	% Area<br>%
1	15.440	2308811	61750	95.09
2	24.571	119142	2869	4.91

Condition：Daicel Chiralpak IC, λ = 254 nm, hexane/2-propanol = 90:10

flow rate = 1.0 mL/min

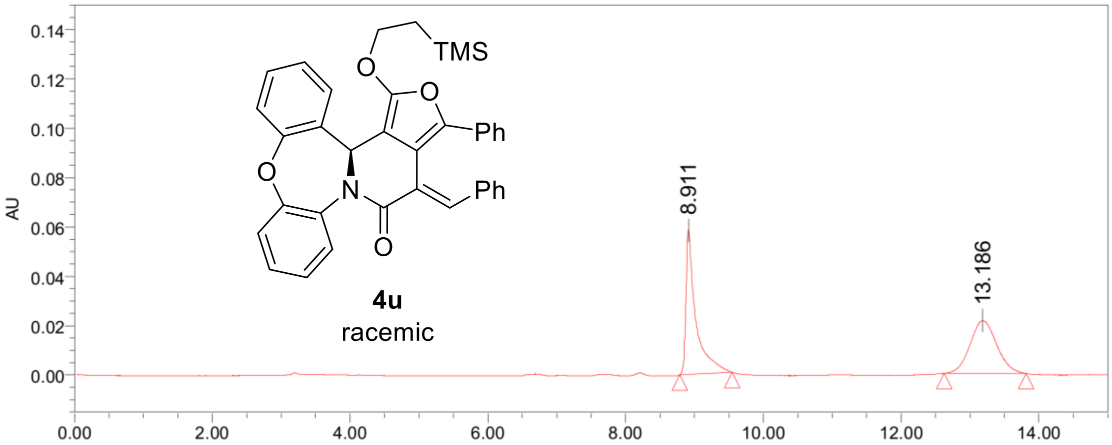

Entry	RT<br>min	Height<br>mV	Area<br>mV.sec	% Area<br>%
1	8.911	588359	58343	50.50
2	13.186	576653	21351	49.50

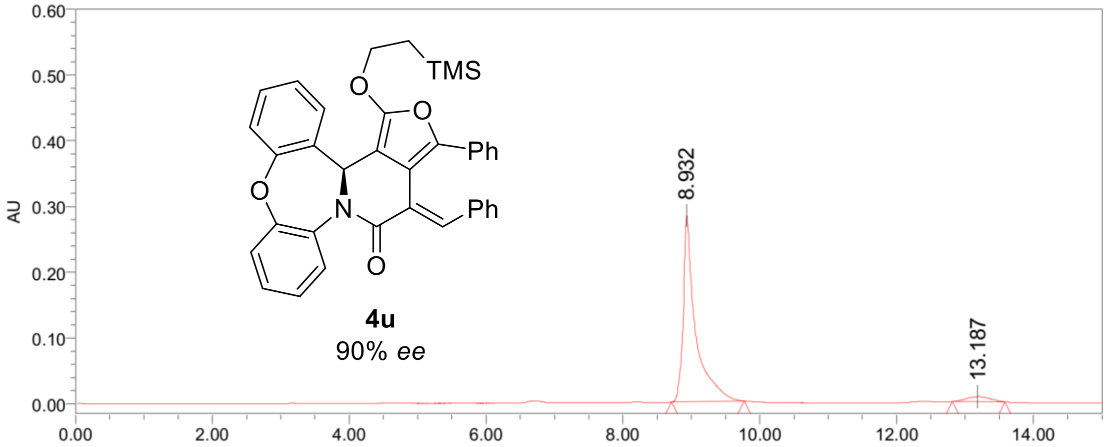

Entry	RT<br>min	Height<br>mV	Area<br>mV.sec	% Area<br>%
1	8.932	3581315	283032	95.02
2	13.187	187670	7769	4.98

Condition: Daicel Chiralpak IA, λ = 254 nm, hexane/2-propanol = 90:10

flow rate = 1.0 mL/min

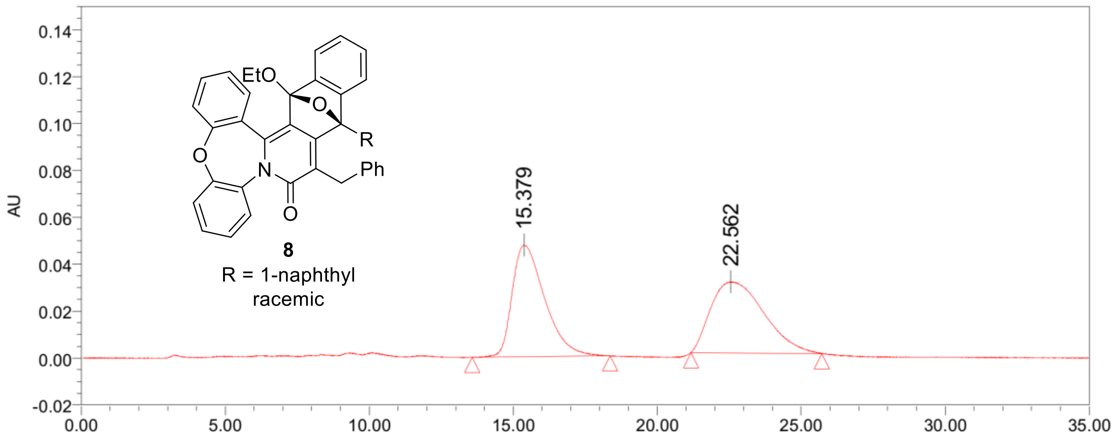

Entry	RT min	Height mV	Area mV.sec	% Area %
1	15.379	3750389	47631	49.04
2	22.562	3897353	30238	50.96

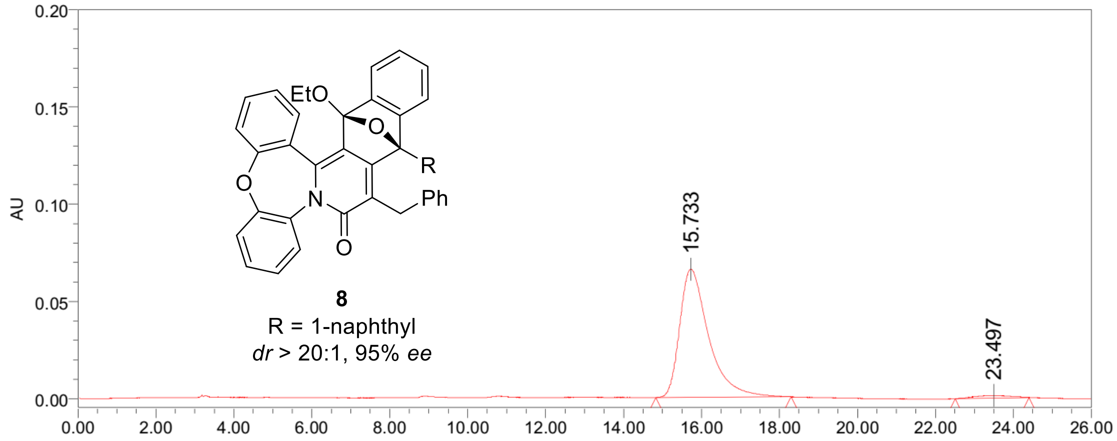

Entry	RT min	Height mV	Area mV.sec	% Area %
1	15.733	3406398	65869	97.61
2	23.497	83464	1307	2.39

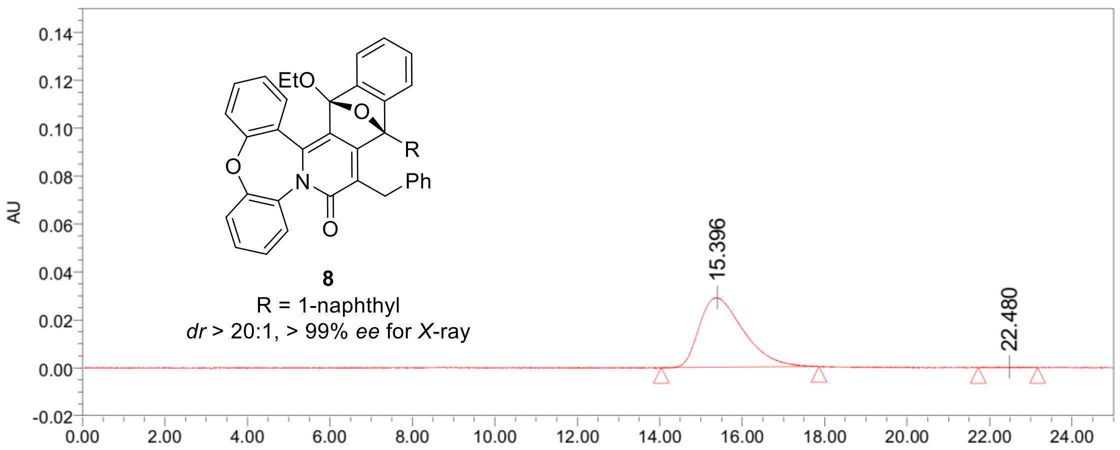

Entry	RT<br>min	Height<br>mV	Area<br>mV.sec	% Area<br>%
1	15.396	2153981	29061	99.79
2	22.480	4527	226	0.21

Condition: Daicel Chiralpak IA, λ = 254 nm, hexane/2-propanol = 70:30

flow rate = 1.0 mL/min

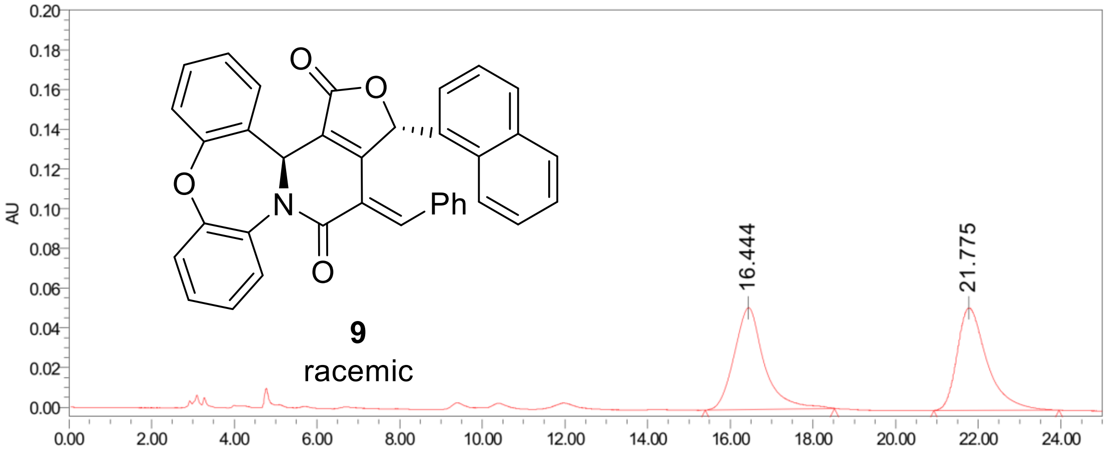

Entry	RT<br>min	Height<br>mV	Area<br>mV.sec	% Area<br>%
1	16.444	2592524	51258	49.94
2	21.775	2598485	51586	50.06

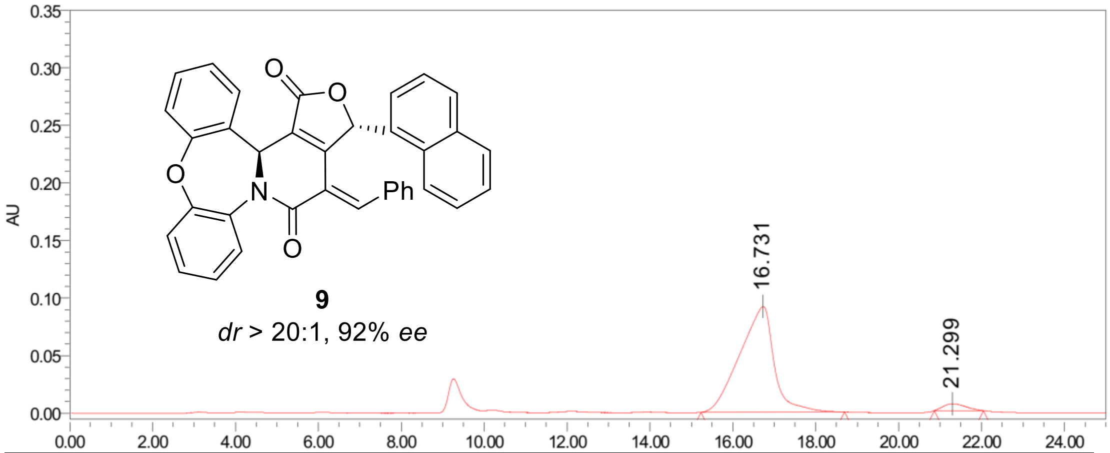

Entry	RT<br>min	Height<br>mV	Area<br>mV.sec	% Area<br>%
1	16.731	5303064	91934	95.94
2	21.299	224137	5974	4.06

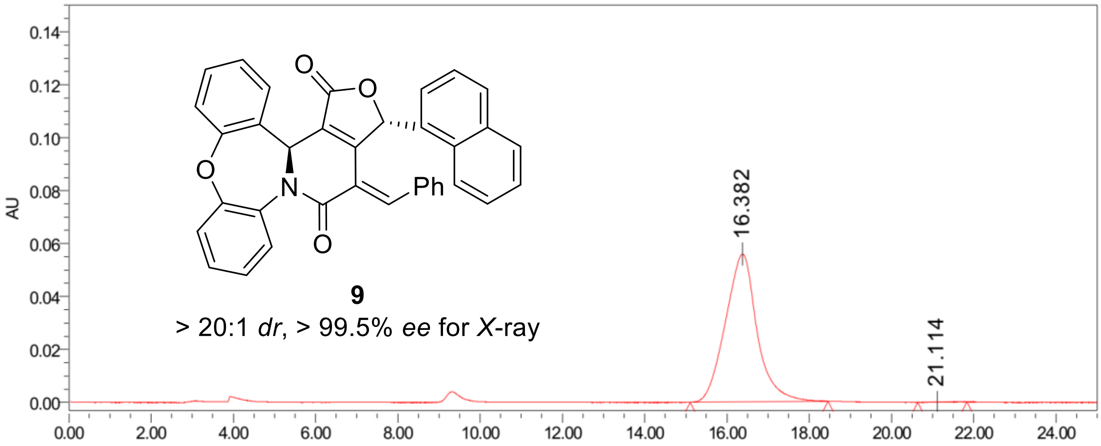

Entry	RT<br>min	Height<br>mV	Area<br>mV.sec	% Area<br>%
1	16.382	2978370	55741	99.94
2	21.114	1811	-62	0.06

Condition: Daicel Chiralpak IC, λ = 254 nm, hexane/2-propanol = 90:10

flow rate = 1.0 mL/min

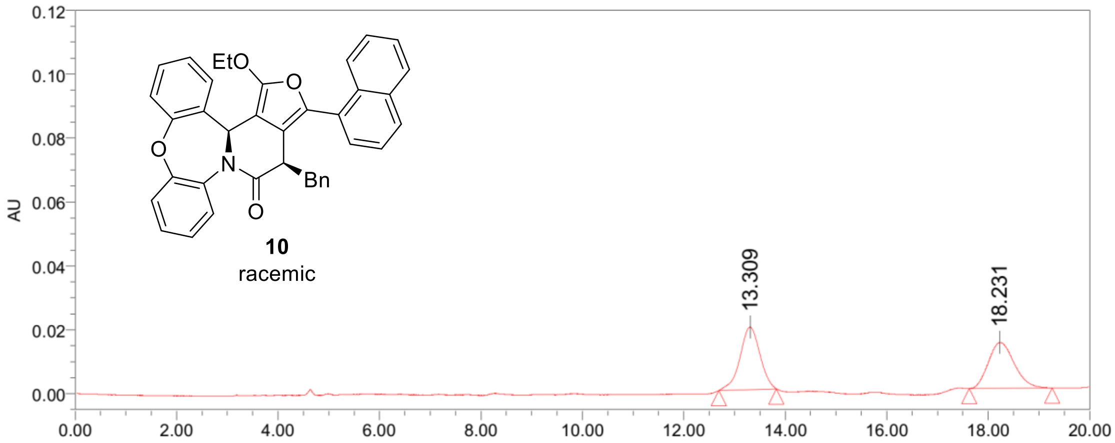

Entry	RT<br>min	Height<br>mV	Area<br>mV.sec	% Area<br>%
1	13.309	532967	19573	50.94
2	18.231	513295	14389	49.06

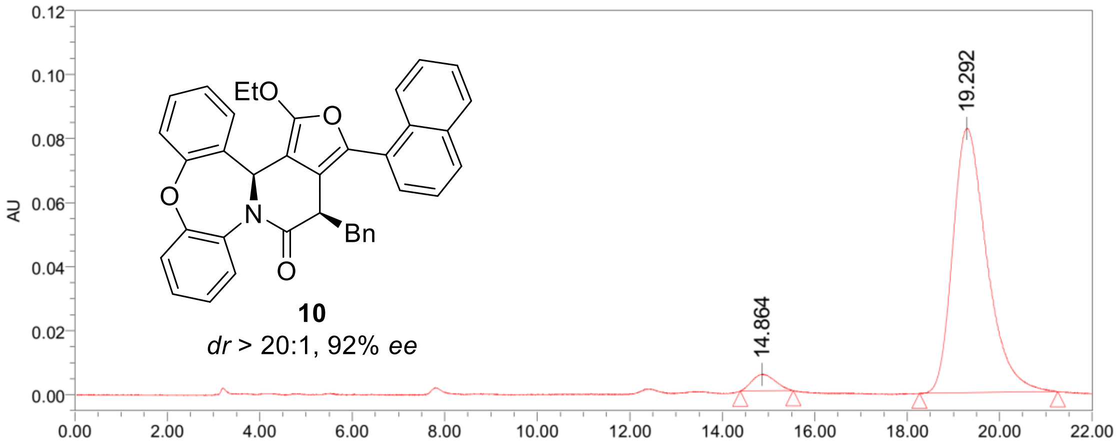

Entry	RT<br>min	Height<br>mV	Area<br>mV.sec	% Area<br>%
1	14.864	182582	5239	4.14
2	19.292	4228196	82562	95.86

Condition：Daicel Chiralpak IC, λ = 254 nm, hexane/2-propanol = 90:10

flow rate = 1.0 mL/min

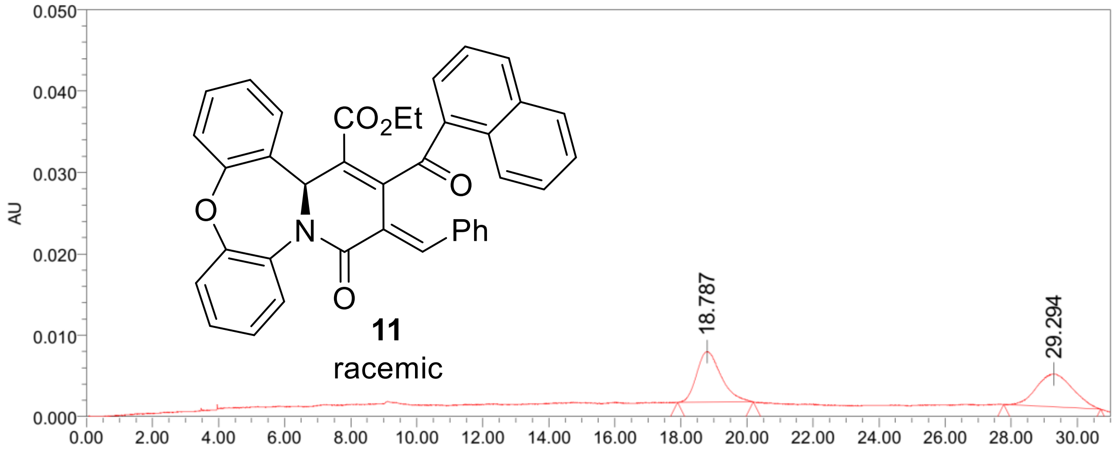

Entry	RT<br>min	Height<br>mV	Area<br>mV.sec	% Area<br>%
1	18.787	320074	6232	50.73
2	29.294	310823	4047	49.27

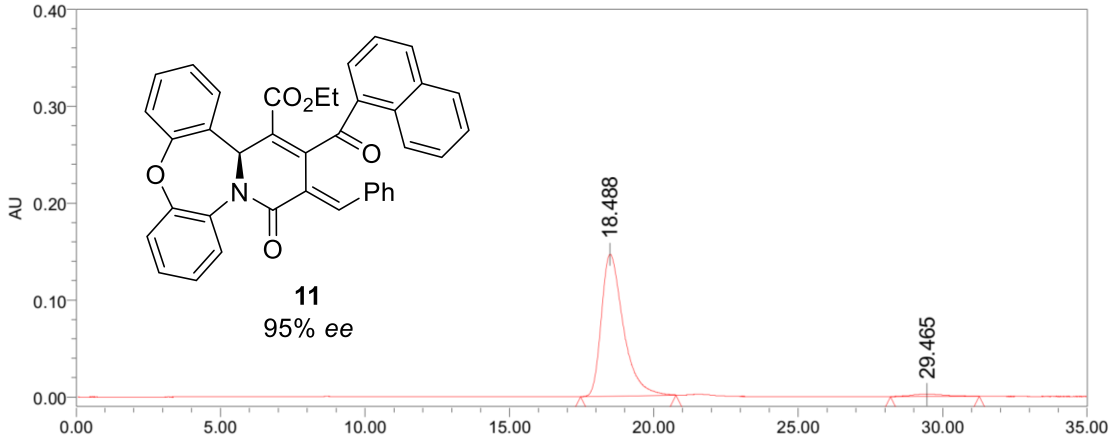

Entry	RT<br>min	Height<br>mV	Area<br>mV.sec	% Area<br>%
1	18.488	7708688	146423	97.64
2	29.465	186640	2289	2.36

Supplementary Fig. 5 | Single-Crystal X-ray Diffraction of 4k

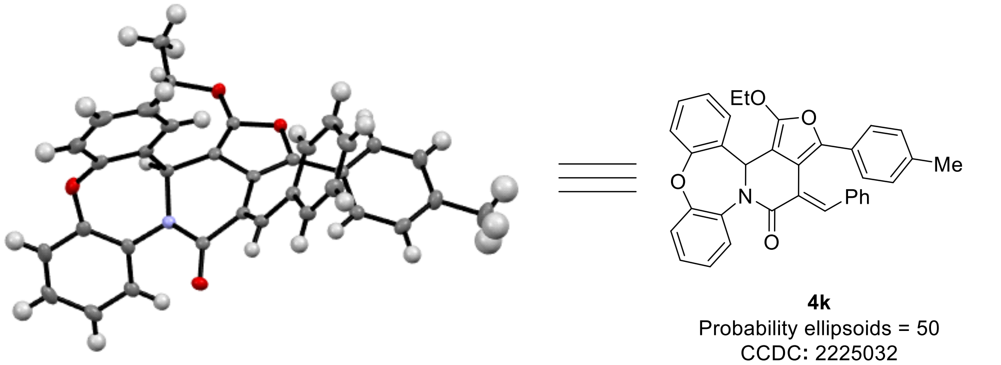

4k
Probability ellipsoids = 50
CCDC: 2225032

Bond precision: C–C = 0.0019 A Wavelength=1.54184

Cell:	a=12.9766(2)	b=14.1296(2)	c=16.5741(2)
	alpha=104.808(1)	beta=94.218(1)	gamma=111.857(1)
Temperature:	100 K		

	Calculated	Reported
Volume	2678.18(7)	2678.17(7)
Space group	P -1	P -1
Hall group	-P 1	-P 1
Moiety formula	C35 H27 N O4	C35 H27 N O4
Sum formula	C35 H27 N O4	C35 H27 N O4
Mr	525.58	525.57
Dx,g cm-3	1.304	1.303
Z	4	4
Mu (mm-1)	0.680	0.680
F000	1104.0	1104.0
F000’	1107.30	
h,k,lmax	16,17,20	16,17,20
Nref	11129	10733
Tmin,Tmax	0.783,0.903	0.484,1.000
Tmin’	0.712	

Correction method= # Reported T Limits: Tmin=0.484 Tmax=1.000
AbsCorr = MULTI-SCAN

Data completeness= 0.964 Theta(max)= 75.570

R(reflections)= 0.0403(10396) wR2(reflections)= 0.1029(10733)

S = 1.053 Npar= 725

Supplementary Fig. 6 | Single-Crystal X-ray Diffraction of 6f

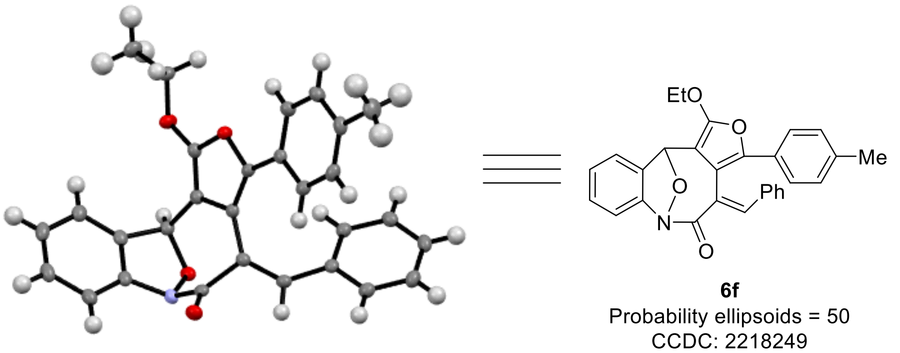

6f
Probability ellipsoids = 50
CCDC: 2218249

Bond precision: C-C = 0.0017 A   Wavelength=1.54184

Cell:	a=7.9086(2)	b=11.3545(3)	c=13.6401(3)
	alpha=70.485(2)	beta=87.467(2)	gamma=77.723(2)
Temperature:	100 K		

	Calculated	Reported
Volume	1127.61(5)	1127.61(5)
Space group	P -1	P -1
Hall group	-P 1	-P 1
Moiety formula	C29 H23 N O4	C29 H23 N O4
Sum formula	C29 H23 N O4	C29 H23 N O4
Mr	449.48	449.48
Dx,g cm-3	1.324	1.324
Z	2	2
Mu (mm-1)	0.711	0.711
F000	472.0	472.0
F000’	473.44	
h,k,lmax	9,14,17	9,14,17
Nref	4688	4453
Tmin,Tmax	0.774,0.808	0.964,1.000
Tmin’	0.752	

Correction method= # Reported T Limits: Tmin=0.964 Tmax=1.000
AbsCorr = MULTI-SCAN

Data completeness= 0.950   Theta(max)= 75.876

R(reflections)= 0.0342(4144)   wR2(reflections)= 0.0882(4453)

S = 1.045   Npar= 310

Supplementary Fig. 7 | Single-Crystal X-ray Diffraction of 8

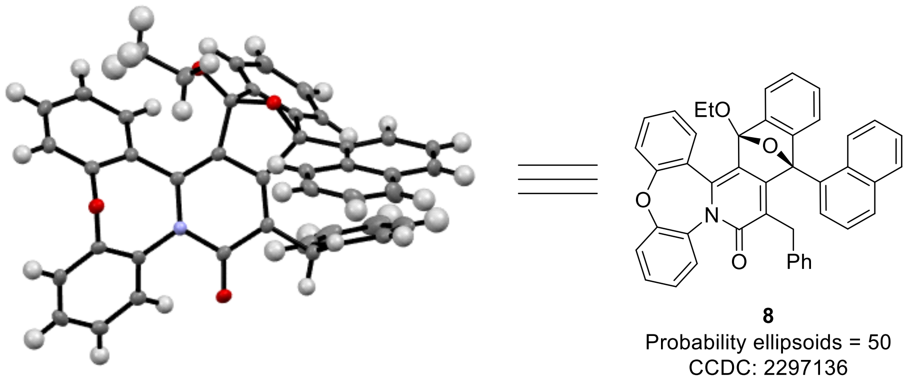

8
Probability ellipsoids = 50
CCDC: 2297136

Bond precision: C-C = 0.0040 A Wavelength=1.54184

Cell:	a=10.2322(1)	b=16.3070(1)	c=19.7132(1)
	alpha=90	beta=99.641(1)	gamma=90
Temperature:	100 K		

	Calculated	Reported
Volume	3242.82(4)	3242.82(4)
Space group	P 21	P 1 21 1
Hall group	P 2yb	P 2yb
Moiety formula	C44 H31 N O4	2(C44 H31 N O4)
Sum formula	C44 H31 N O4	C88 H62 N2 O8
Mr	637.70	1275.39
Dx,g cm-3	1.306	1.306
Z	4	2
Mu (mm-1)	0.662	0.662
F000	1336.0	1336.0
F000’	1339.92	
h,k,lmax	12,20,24	12,20,24
Nref	13440[6965]	12773
Tmin,Tmax	0.888,0.936	0.766,1.000
Tmin’	0.820	

Correction method= # Reported T Limits: Tmin=0.766 Tmax=1.000
AbsCorr = MULTI-SCAN

Data completeness= 1.83/0.95 Theta(max)= 75.453

R(reflections)= 0.0352(12487) wR2(reflections)= 0.0843(12773)

S = 1.108 Npar= 886

Supplementary Fig. 8 | Single-Crystal X-ray Diffraction of 9

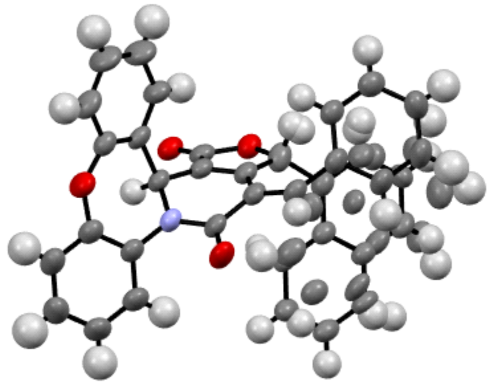

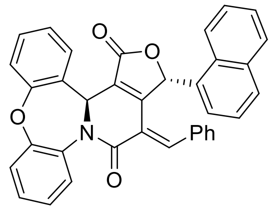

9
Probability ellipsoids = 50
CCDC: 2309002

Bond precision:	C-C = 0.0110 A		Wavelength=1.54184
Cell:	a=10.2145(4)	b=9.5543(4)	c=13.9690(4)
	alpha=90	beta=103.071(4)	gamma=90
Temperature:	100 K		

	Calculated	Reported
Volume	1327.95(9)	1327.95(9)
Space group	P 21	P 1 21 1
Hall group	P 2yb	P 2yb
Moiety formula	C36 H23 N O4	C36 H23 N O4
Sum formula	C36 H23 N O4	C36 H23 N O4
Mr	533.55	533.55
Dx,g cm-3	1.334	1.334
Z	2	2
Mu (mm-1)	0.699	0.699
F000	556.0	556.0
F000'	557.68	
h,k,lmax	12,11,16	12,11,16
Nref	4697[2504]	4571
Tmin,Tmax	0.920,0.946	0.268,1.000
Tmin'	0.900	

Correction method= # Reported T Limits: Tmin=0.268 Tmax=1.000
AbsCorr = MULTI-SCAN

Data completeness= 1.83/0.97 Theta(max)= 66.580

R(reflections)= 0.1077(3906) wR2(reflections)= 0.2596(4571)

S = 0.883 Npar= 389

Alert level B

PLAT340_ALERT_3_B Low Bond Precision on C-C Bonds .... 0.011 Ang.

Author Response: The structure is badly disordered in both the framework and the guest molecules, which causes the low bond precision on C-C bonds.

Supplementary Fig. 9 | Single-Crystal X-ray Diffraction of 14

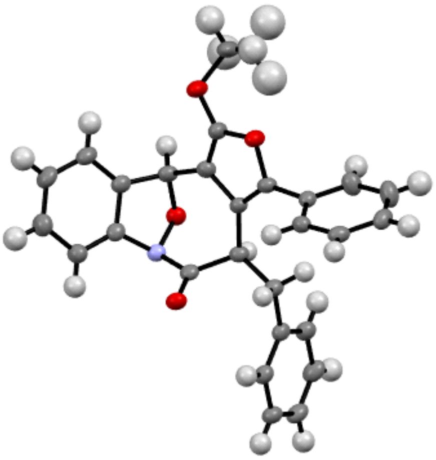

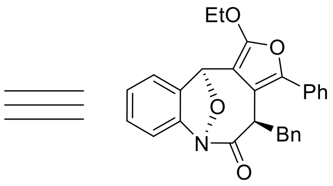

14
Probability ellipsoids = 50
CCDC: 2348279

Bond precision: C-C = 0.0042 A Wavelength=1.54184

Cell: a=11.5066(2) b=17.6790(3) c=23.5765(4)
alpha=84.220(2) beta=79.147(2) gamma=71.731(2)

Temperature: 100 K

	Calculated	Reported
Volume	4468.38(14)	4468.38(15)
Space group	P -1	P -1
Hall group	-P 1	-P 1
Moiety formula	C28 H23 N O4	C28 H23 N O4
Sum formula	C28 H23 N O4	C28 H23 N O4
Mr	437.47	437.47
Dx,g cm-3	1.301	1.301
Z	8	8
Mu (mm-1)	0.702	0.702
F000	1840.0	1840.0
F000’	1845.63	
h,k,lmax	14,22,29	14,22,29
Nref	18582	17583
Tmin,Tmax	0.810,0.869	0.740,1.000
Tmin’	0.810	

Correction method= # Reported T Limits: Tmin=0.740 Tmax=1.000
AbsCorr = MULTI-SCAN

Data completeness= 0.946 Theta(max)= 75.579

R(reflections)= 0.0687(11961) wR2(reflections)= 0.1889(17583)

S = 1.036 Npar= 1193

General Procedure for the *in vitro* Anti-tumor Activity Study

Cell viability was measured by CCK-8 assay

Human cancer cell lines HCT116, A549, and KYSE-520 were obtained from Cell Cook. Cells were cultured in RPMI1640 medium containing 10% fetal bovine serum and 1% penicillin/streptomycin (Gibco) in a humidified incubator containing 5% CO_2 at 37 °C. Human cancer cell lines MCF-7 was obtained from Procell and cells were cultured in MEM medium containing 10% fetal bovine serum, 1% penicillin/streptomycin (Gibco) and 0.01 mg/mL insulin (Procell) in a humidified incubator containing 5% CO_2 at 37 °C. For cell viability, cells were seeded in 96-well plates at 5000 cells per well. After 24 hours, serially diluted compounds were added and cells were cultured for another 48 hours. Cell viability was measured using a Cell Counting Kit-8 (CCK-8) assay according to the manufacturer's instructions (Yeasen Biotechnology, China).

These representative products **6b - 6o**, **6s**, **7b - 7c**, **7e**, **7g - 7i**, and **7l - 7m** on cell viability was evaluated *via* CCK8 assay in HCT116 (colon cancer), MCF-7 (breast cancer), A549 (lung adenocarcinoma), and KYSE-520 (esophageal squamous cell carcinoma) human cancer cell lines, and the *in vitro* anti-tumor activity results are listed in **Supplementary Table 12-13**.

Most of these poly-substituted furan-fused eight-membered lactams, such as **6e**, **6f**, **6h**, and **6m** showed significant anti-cancer activity in comparison to the Paclitaxel (**Supplementary Fig. 10-14**), The results demonstrated that compound **6e** shows the highest anticancer potency against human colon cancer cells (HCT-116 cells, IC_{50} = 0.50 ± 0.05 μM), compound **6f** possesses the highest anticancer potency against human esophageal squamous cell carcinoma cells (KYSE-520 cells, IC_{50} = 0.89 ± 0.13 μM). These results were presented as percentages and vehicle-treated cells set at 5000. All data were presented as mean values ± SD, n = 3.

Supplementary Table 12 | Anti-tumor activities of compounds 6b - 6o, 6s, 7b - 7c, 7e, 7g - 7i, and 7l - 7m (inhibition rate at 20 μM).

Compound	HCT-116 (%)	MCF-7 (%)	A549 (%)	KYSE-520 (%)
**6b**	97.34±0.22	62.14±2.77	53.19±0.31	96.89±0.75
**6c**	97.80±0.43	65.23±3.05	60.66±2.57	98.03±0.55
**6d**	90.14±1.38	58.58±3.78	55.30±5.05	92.29±2.70
**6e**	91.53±0.66	55.48±2.46	60.38±2.00	90.98±1.27
**6f**	96.40±0.41	68.24±0.30	62.86±3.89	96.30±1.40
**6g**	98.89±0.25	89.16±1.91	91.01±2.20	99.05±0.48
**6h**	92.05±0.63	53.63±6.02	59.68±3.08	96.04±0.75
**6i**	97.73±0.44	56.33±1.39	73.64±2.24	93.16±1.12
**6j**	97.44±0.96	61.57±3.15	51.09±0.39	96.16±0.10
**6k**	96.43±2.14	69.21±1.49	86.25±2.14	98.41±0.60
**6l**	80.04±3.16	49.54±2.68	37.76±7.13	99.28±0.73
**6m**	93.26±1.12	72.59±2.64	69.54±2.35	98.06±0.17
**6n**	98.52±0.42	75.28±4.57	76.32±1.51	98.42±0.49
**6o**	99.25±0.24	78.98±5.11	84.25±3.57	97.54±0.16
**6s**	77.14±1.40	53.30±3.42	48.82±0.39	73.00±1.98
**7b**	91.09±4.67	52.14±5.40	34.49±3.34	94.79±0.78
**7c**	98.03±0.77	72.28±3.47	65.95±1.10	99.05±0.27
**7e**	99.11±0.49	79.23±5.02	86.87±2.76	98.14±0.55
**7g**	91.03±0.61	59.55±1.37	38.90±3.54	97.91±0.46
**7h**	97.01±0.36	82.43±0.50	81.40±1.82	98.65±0.66
**7i**	96.91±0.51	69.24±4.45	66.90±2.48	98.57±0.09
**7l**	75.97±1.57	56.02±2.84	38.13±4.59	92.09±1.25
**7m**	69.85±4.21	43.67±3.16	32.93±2.45	79.34±4.51
**Taxol**	73.85±1.57	89.20±1.08	85.52±5.20	90.46±2.11

Supplementary Table 13 | Anti-tumor activities of compounds 6b - 6o, 7b - 7c, 7e, 7g - 7i, and 7l (IC_{50}, μM)[a]

Compound	HCT-116	MCF-7	A549	KYSE-520
**6b**	0.99±0.31	-	-	
**6c**	0.89±0.07	-	-	
**6d**	3.11±0.73	-	-	2.62±0.41
**6e**	0.50±0.05	-	-	2.03±0.31
**6f**	2.09±0.31	-	-	0.89±0.13
**6g**	1.00±0.07		-	
**6h**	2.92±0.39	-	-	0.95±0.10
**6i**	0.77±0.07	-	-	
**6j**	1.07±0.06	-	-	1.46±0.36
**6k**	1.18±0.11	-	-	
**6l**	-	-	-	2.25±0.24
**6m**	1.34±0.13	-	-	1.53±0.30
**6n**	1.23±0.05	-	-	
**6o**	0.69±0.09	-	-	
**7b**	0.61±0.09	-	-	1.69±0.22
**7c**	2.81±0.58	-	-	
**7e**	1.13±0.16	-	-	
**7g**	1.86±0.12	-	-	1.76±0.21
**7h**	0.86±0.08	-	-	
**7i**	0.93±0.07	-	-	1.19±0.21
**7l**	-	-	-	1.81±0.26

[a]IC_{50} is the half maximal inhibitory concentration. All data were presented as mean values ± SD, n = 3.

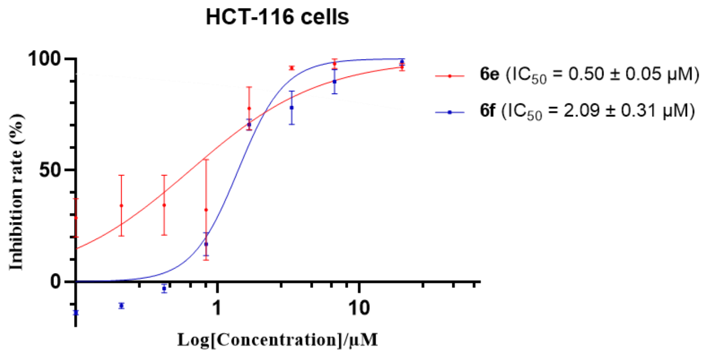

Supplementary Fig. 10 | Compounds 6e and 6f on the inhibition of HCT-116 cells, data were presented as mean values ± SD, n = 3.

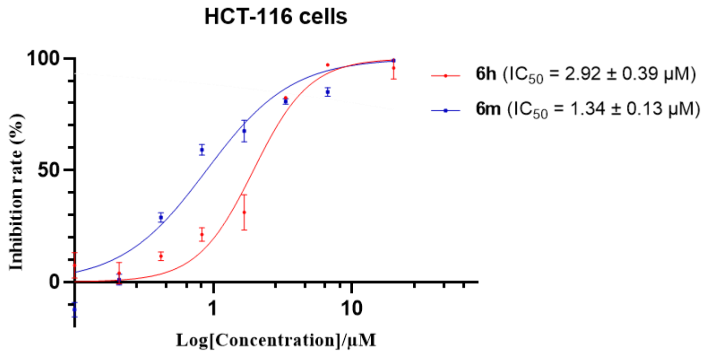

Supplementary Fig. 11 | Compounds 6h and 6m on the inhibition of HCT-116 cells, data were presented as mean values ± SD, n = 3.

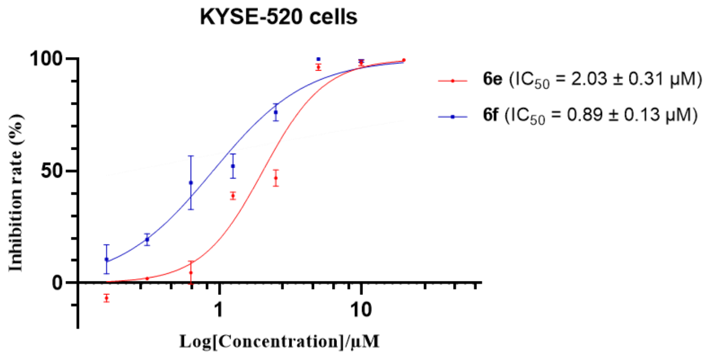

Supplementary Fig. 12 | Compounds 6e and 6f on the inhibition of KYSE-520 cells, data were presented as mean values ± SD, n = 3.

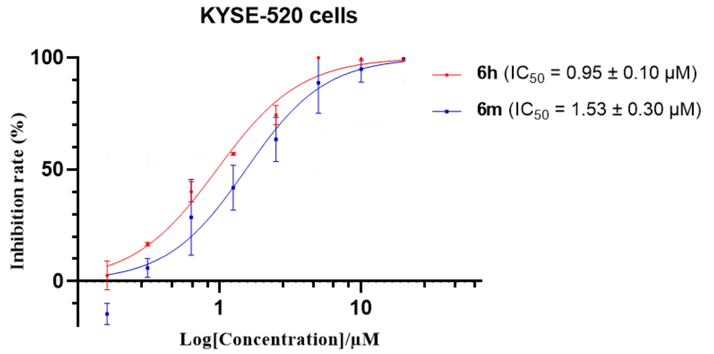

Supplementary Fig. 13 | Compounds 6h and 6m on the inhibition of KYSE-520 cells, data were presented as mean values ± SD, n = 3.

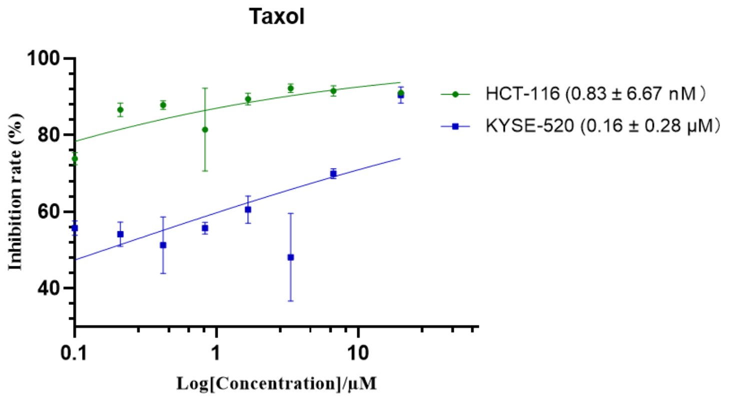

Supplementary Fig. 14 | Taxol on the inhibition of HCT-116 and KYSE-520 cells, data were presented as mean values ± SD, n = 3.

Human normal colon mucosal epithelial cell line NCM460 was obtained from Cell Cook. Cells were cultured in RPMI1640 medium containing 10% fetal bovine serum and 1% penicillin/streptomycin (Gibco) in a humidified incubator containing 5% CO_2 at 37 °C. For cell viability, cells were seeded in 96-well plates at 5000 cells per well. After 24 hours, serially diluted compounds were added and cells were cultured for another 48 hours. Cell viability was measured using a Cell Counting Kit-8 (CCK-8) assay according to the manufacturer's instructions (Yeasen Biotechnology, China).

These representative products **6e** and **6f** on cell viability were evaluated *via* CCK8 assay in NCM460 (colon mucosal epithelial) human normal cell line. The results show that compounds **6e** and **6f** exhibit no obvious cytotoxicity against human colon mucosal epithelial cells (NCM460 cells, **6e**: IC_{50} = 32.30 ± 1.23 μM; **6f**: IC_{50} = 23.89 ± 1.01 μM). The results were presented as percentages and vehicle-treated cells set at 100 (Supplementary Fig. 15).

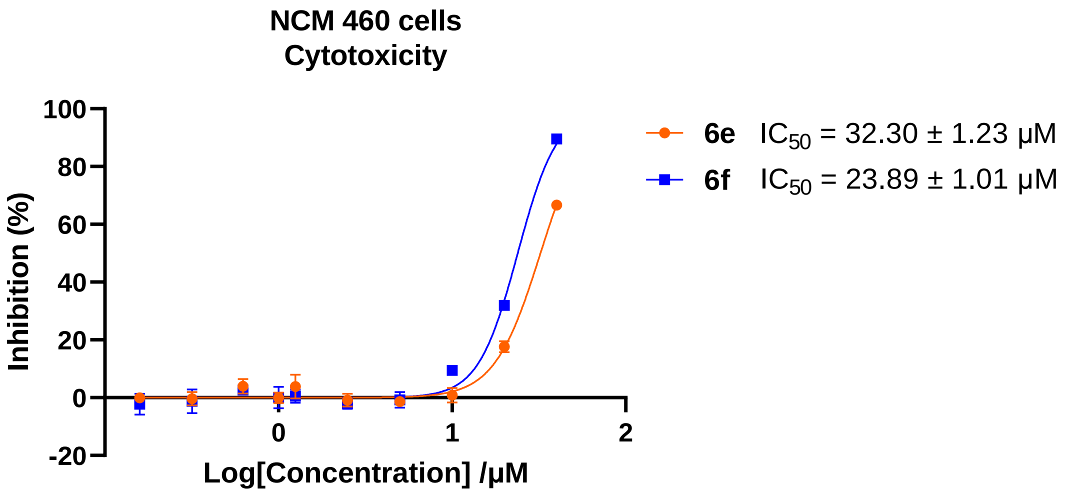

Supplementary Fig. 15 | Compounds 6e and 6f on the inhibition of NCM460 cells, data were presented as mean values ± SD, n = 3.

Supplementary References

1. Hong, K. et al. Catalytic 4-*exo-dig* carbocyclization for the construction of furan-fused cyclobutanones and synthetic applications. *Nat. Commun.* **14**, 6378 (2023).

2. Xie, X., Bao, M., Chen, K., Xu, X. & Hu, W. An asymmetric three-component reaction of a diazo compound with an alcohol and a seven-membered imine. *Org. Chem. Front.* **9**, 2102-2108 (2022).

3. Bao, M., Xie, X., Hu, W. & Xu, X. Gold-catalyzed carbocyclization/C=N bond formation cascade of alkyne-tethered diazo compounds with benzo[*c*]isoxazoles for the assembly of 4-iminonaphthalenones and indenes. *Adv. Synth. Catal.* **363**, 4018-4023 (2021).

4. Breuil, P.-A. R., Patureau, F. W. & Reek, J. N. H. Singly hydrogen bonded supramolecular ligands for highly selective rhodium-catalyzed hydrogenation reactions. *Angew. Chem. Int. Ed.* **48**, 2162-2165 (2009).
