# Supplementary material for: Catalytic [4+2]- and [4+4]-cycloaddition using furan-fused cyclobutanone as a privileged C4 synthon
Source: Nat Commun. 2024 Jun 26;15:5407. doi: 10.1038/s41467-024-49664-5 (PMC11208666; doi:10.1038/s41467-024-49664-5)
Supplement: Supplementary file 3 — Reporting Summary [file 41467_2024_49664_MOESM3_ESM.pdf]

nature portfolio

Corresponding author(s): Xinfang Xu

Last updated by author(s): Apr 24, 2024

Reporting Summary

Nature Portfolio wishes to improve the reproducibility of the work that we publish. This form provides structure for consistency and transparency in reporting. For further information on Nature Portfolio policies, see our Editorial Policies and the Editorial Policy Checklist.

Statistics

For all statistical analyses, confirm that the following items are present in the figure legend, table legend, main text, or Methods section.

n/a	Confirmed	
☐	☒	The exact sample size (n) for each experimental group/condition, given as a discrete number and unit of measurement
☐	☒	A statement on whether measurements were taken from distinct samples or whether the same sample was measured repeatedly
☐	☒	The statistical test(s) used AND whether they are one- or two-sided<br>*Only common tests should be described solely by name; describe more complex techniques in the Methods section.*
☐	☒	A description of all covariates tested
☐	☒	A description of any assumptions or corrections, such as tests of normality and adjustment for multiple comparisons
☐	☒	A full description of the statistical parameters including central tendency (e.g. means) or other basic estimates (e.g. regression coefficient) AND variation (e.g. standard deviation) or associated estimates of uncertainty (e.g. confidence intervals)
☒	☐	For null hypothesis testing, the test statistic (e.g. F, t, r) with confidence intervals, effect sizes, degrees of freedom and P value noted<br>*Give P values as exact values whenever suitable.*
☒	☐	For Bayesian analysis, information on the choice of priors and Markov chain Monte Carlo settings
☒	☐	For hierarchical and complex designs, identification of the appropriate level for tests and full reporting of outcomes
☒	☐	Estimates of effect sizes (e.g. Cohen's d, Pearson's r), indicating how they were calculated

Our web collection on statistics for biologists contains articles on many of the points above.

Software and code

Policy information about availability of computer code

Data collection	n/a
Data analysis	n/a

For manuscripts utilizing custom algorithms or software that are central to the research but not yet described in published literature, software must be made available to editors and reviewers. We strongly encourage code deposition in a community repository (e.g. GitHub). See the Nature Portfolio guidelines for submitting code & software for further information.

Data

Policy information about availability of data

All manuscripts must include a data availability statement. This statement should provide the following information, where applicable:

- Accession codes, unique identifiers, or web links for publicly available datasets
- A description of any restrictions on data availability
- For clinical datasets or third party data, please ensure that the statement adheres to our policy

All data are available from the corresponding author upon request. The data supporting the findings of this study are available within the paper and its Supplementary Information. Crystallographic data for the structures reported in this article have been deposited at the Cambridge Crystallographic Data Centre, under deposition numbers CCDC 2225032 (4k), 2218249 (6f), 2297136 (8), 2309002 (9), and 2348279 (14). Copies of the data can be obtained free of charge via https://www.ccdc.cam.ac.uk/structures/.

Research involving human participants, their data, or biological material

Policy information about studies with human participants or human data. See also policy information about sex, gender (identity/presentation), and sexual orientation and race, ethnicity and racism.

Reporting on sex and gender	n/a
Reporting on race, ethnicity, or other socially relevant groupings	n/a
Population characteristics	n/a
Recruitment	n/a
Ethics oversight	n/a

Note that full information on the approval of the study protocol must also be provided in the manuscript.

Field-specific reporting

Please select the one below that is the best fit for your research. If you are not sure, read the appropriate sections before making your selection.

[x] Life sciences [ ] Behavioural & social sciences [ ] Ecological, evolutionary & environmental sciences

For a reference copy of the document with all sections, see nature.com/documents/nr-reporting-summary-flat.pdf

Life sciences study design

All studies must disclose on these points even when the disclosure is negative.

Sample size	The IC50 values of each cell were tested in at least three parallel groups and eight concentrations were measured in each group of cells. In case that a fold difference in the IC50 value bigger than 10-fold was obtained between replicates, an additional replicate was performed. In addition, the complete dose-effect curve can be accurately fitted with 5-8 concentration points. In general, the obtained IC50 values from three parallel experiments met that criteria and hence we consider that sample size accurate enough to report the result.
Data exclusions	No data was excluded.
Replication	To ensure robust reproducibility: All bioassay data presented in this manuscript were repeated twice times at least. And all attempts to repeat the experiment were successful.
Randomization	Cells were randomly divided into experimental groups, all other samples collected were used for this study without any discrimination.
Blinding	The evaluation of bioactivity of furan-fused eight-membered lactams derivatives were conducted in blinding. The structures and resources information of the tested compounds were unknown to the investigator who conducted bioactivity test, to ensure the objective data collection and analysis.

Reporting for specific materials, systems and methods

We require information from authors about some types of materials, experimental systems and methods used in many studies. Here, indicate whether each material, system or method listed is relevant to your study. If you are not sure if a list item applies to your research, read the appropriate section before selecting a response.

Materials & experimental systems

n/a	Involved in the study	
[x]	[ ]	Antibodies
[ ]	[x]	Eukaryotic cell lines
[x]	[ ]	Palaeontology and archaeology
[x]	[ ]	Animals and other organisms
[x]	[ ]	Clinical data
[x]	[ ]	Dual use research of concern
[x]	[ ]	Plants

Methods

n/a	Involved in the study	
[x]	[ ]	ChIP-seq
[x]	[ ]	Flow cytometry
[x]	[ ]	MRI-based neuroimaging

Eukaryotic cell lines

Policy information about cell lines and Sex and Gender in Research

Cell line source(s)	The human cancer cell lines HCT-116 (CCL-228), MCF-7 (HTB-22), and A549 (CCL-185) were obtained from the American Type Culture Collection (ATCC, Manassas, VA), KYSE-520 cell line was purchased from BioVector NTCC Inc. (Beijing, China). The human normal colonic epithelial cell (NCM460) was purchased from China Center for Type Culture Collection (CCTCC, Shanghai, China). All cells were cultured in RPMI1640 medium containing 10% fetal bovine serum and 1% penicillin/streptomycin (Gibco) in a humidified incubator containing 5% CO_2 at 37 °C.
Authentication	The original cell lines were purchased from vendors as mentioned above, and all cells were cultured in RPMI1640 medium containing 10% fetal bovine serum and 1% penicillin/streptomycin (Gibco) in a humidified incubator containing 5% CO_2 at 37 °C. Then used for the testing study without authentication.
Mycoplasma contamination	Negative for the mycoplasma contamination
Commonly misidentified lines (See ICLAC register)	n/a

Plants

Seed stocks	n/a
Novel plant genotypes	n/a
Authentication	n/a
